# Isothiourea‐Catalysed Acylative Kinetic and Dynamic Kinetic Resolution of Planar Chiral Paracyclophanols

**DOI:** 10.1002/anie.202507126

**Published:** 2025-06-23

**Authors:** Zhanyu Zhou, Kevin Kasten, Aidan P. McKay, David B. Cordes, Andrew D. Smith

**Affiliations:** ^1^ EaStCHEM, School of Chemistry University of St Andrews St Andrews Fife KY16 9ST UK

**Keywords:** Dynamic kinetic resolution, Isothiourea, Kinetic resolution, Macrocycles, Paracyclophane

## Abstract

The development of synthetic methods for the catalytic enantioselective preparation of planar chiral paracyclophane derivatives is of considerable interest to the synthetic community. To date, relatively few successful and effective organocatalytic approaches to this molecular class have been reported. This manuscript describes effective isothiourea catalysed acylative kinetic (KR) and dynamic kinetic resolution (DKR) approaches to the generation of a range of planar chiral paracyclophane macrocycles with excellent levels of enantioselectivity. Effective KR of configurationally stable planar chiral paracyclophanols with 12‐ and 13‐membered *ansa*‐chains is demonstrated (6 examples, *s* = up to 50) using 5 mol% of the isothiourea (*R*)‐BTM and isobutyric anhydride. Application to configurationally labile macrocyclic phenols with 14 to 18‐membered *ansa*‐chains allows their effective acylative DKR, generating the desired products with excellent enantioselectivity (25 examples, up to 95% yield and 98:2 er).

## Graphical abstract

The highly selective acylative (dynamic) kinetic resolution of racemic paracyclophanols catalysed by a chiral isothiourea (5 mol%) is described (up to *s* = 50 at 49% conversion or up to 98:2 er and 96% yield in 30 examples). Rotational barriers were analysed for paracyclophanols and selected ester products.

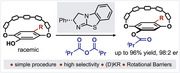

## Introduction

**Figure 1:**
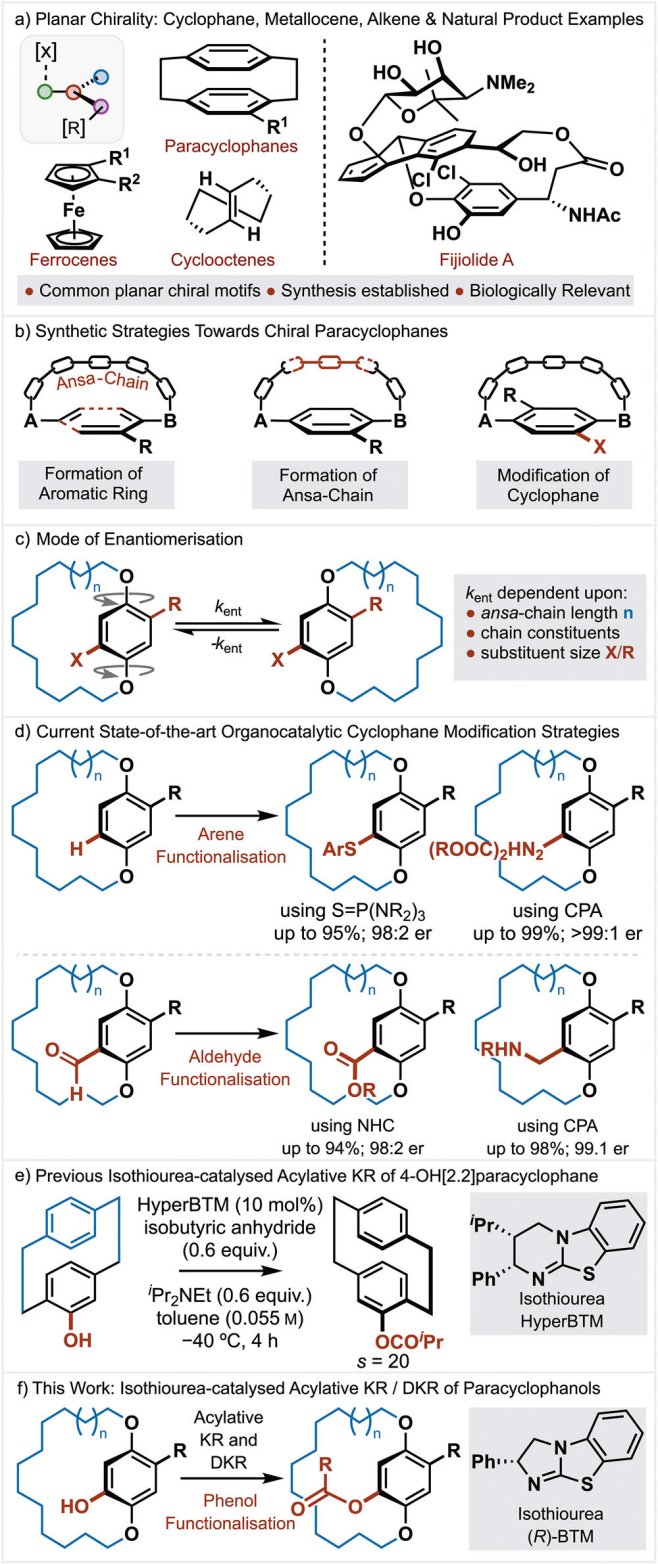
a) Planar chirality: cyclophane, metallocene, alkene, and natural product. b) Synthetic strategies towards chiral paracyclophanes. c) Mode of enantiomerization. d) Current state‐of‐the‐art organocatalytic cyclophane modification strategies. e) Previous isothiourea‐catalysed KR of [2.2]paracyclophan‐4‐ol. f) This work: isothiourea‐catalysed acylative KR/DKR of paracyclophanols.

Planar chirality describes stereoisomerism that results from the arrangement of out‐of‐plane groups in relation to a reference plane (Figure 1a).^[^ ^1^, ^2^ ^]^ A range of molecular types that encompass macrocycles, such as cyclophanes and bridged annulenes, as well as cycloalkenes and metallocenes, have been prepared that demonstrate this configurational phenomenon. Such species are of significant interest from a synthetic,^[^ ^3^, ^4^, ^5^, ^6^, ^7^, ^8^, ^9^, ^10^, ^11^, ^12^, ^13^, ^14^, ^15^, ^16^, ^17^ ^]^ structural,^[^ ^18^, ^19^, ^20^, ^21^, ^22^, ^23^, ^24^, ^25^, ^26^, ^27^, ^28^ ^]^ and chiroptical perspective.^[^ ^29^ ^]^ In addition, planar chiral macrocycles are found within natural products^[^ ^30^, ^31^, ^32^ ^]^ and bioactive species^[^ ^7^, ^13^, ^14^, ^33^, ^34^, ^35^, ^36^, ^37^, ^38^, ^39^, ^40^, ^41^, ^42^, ^43^, ^44^, ^45^, ^46^, ^47^, ^48^, ^49^, ^50^, ^51^, ^52^, ^53^, ^54^, ^55^, ^56^, ^57^, ^58^, ^59^, ^60^, ^61^, ^62^, ^63^, ^64^, ^65^, ^66^, ^67^, ^68^ ^]^ such as Fijiolides.^[^ ^69^ ^]^ Within this class, a cyclophane can be defined as a macrocycle that is made up of aromatic units connected by chain linkers.^[^ ^70^ ^]^ In mancude‐ring systems (such as benzene) that are bridged by a linker, the so‐called *ansa*‐chain is constrained to lie over one face of the ring, with asymmetric substitution of the arene leading to the formation of chiral products. In such structures, configurational stability arises due to restricted rotation of the aromatic ring, with substituents needing to pass through the *ansa*‐chain to interconvert enantiomers. Because of their structural interest and dynamic behaviour, several methods have been developed for the asymmetric synthesis of planar chiral cyclophanes (Figure 1b).^[^ ^5^, ^6^, ^10^, ^16^, ^17^, ^21^ ^]^ These include elegant methods for the construction of either the *ansa*‐chain,^[^ ^71^, ^72^, ^73^, ^74^, ^75^, ^76^, ^77^, ^78^, ^79^, ^80^, ^81^, ^82^ ^]^ the arene within the chiral plane,^[^ ^83^, ^84^, ^85^, ^86^, ^87^ ^]^ or stereoselective transformation of the chiral plane employing chiral auxiliaries^[^ ^42^, ^88^, ^89^, ^90^, ^91^, ^92^, ^93^, ^94^, ^95^ ^]^ and reagents,^[^ ^96^, ^97^ ^]^ as well as enantioselective desymmetrisation^[^ ^98^, ^99^, ^100^ ^]^ or (dynamic) kinetic resolution [(D)KR] processes.^[^ ^98^, ^101^, ^102^, ^103^, ^104^, ^105^, ^106^, ^107^, ^108^, ^109^, ^110^, ^111^, ^112^, ^113^, ^114^, ^115^ ^]^ In the latter area, the rate of enantiomerization is dependent upon size and position of arene substituents within the plane, as well as *ansa*‐chain length and its constitution (Figure 1c).^[^ ^5^, ^16^, ^17^, ^21^, ^116^, ^117^, ^118^ ^]^ Either reducing the size of the *ansa*‐chain or increasing the size of the substituents within the chiral plane can lead to configurationally stable products. Several methods that allow modification of the arene in this manner encompass asymmetric Sonagashira coupling,^[^ ^101^ ^]^ or lithiation‐trapping approaches,^[^ ^98^ ^]^ as well as C─H‐activation‐olefination or ‐arylation using transition metal based catalysts.^[^ ^105^, ^112^ ^]^ To date, limited organocatalytic (D)KR approaches have been demonstrated in this area to generate chiral paracyclophanes.^[^ ^102^, ^103^, ^107^, ^115^ ^]^ The current state‐of‐the‐art methods utilise a direct electrophilic arene substitution process via either CPA‐promoted electrophilic amination^[^ ^104^ ^]^ or Lewis base‐catalysed electrophilic sulfenylation of arenes.^[^ ^111^ ^]^ Alternatively, functionalisation of a macrocyclic aldehyde via either NHC‐catalysed oxidative esterification (that requires a stoichiometric oxidant)^[^ ^109^ ^]^ or chiral phosphoric acid (CPA) catalysed transfer hydrogenation to promote reductive amination^[^ ^106^ ^]^ has been achieved (Figure 1d).

The acylative KR and DKR of racemic alcohols is a strategy that has been widely adopted by both industry and academia to deliver enantiomerically pure products.^[^ ^119^, ^120^ ^]^ The use of nitrogen‐centred Lewis base catalysts has been widely described in this area,^[^ ^121^, ^122^, ^123^, ^124^ ^]^ with the use of isothiourea catalysts having been applied to the KR of a range of primary,^[^ ^125^ ^]^ secondary,^[^ ^126^, ^127^, ^128^, ^129^, ^130^, ^131^ ^]^ and tertiary^[^ ^132^ ^]^ point chiral alcohols since the first demonstration of their utility in KR by Birman and co‐workers.^[^ ^133^ ^]^ Application to the KR of 2,6‐dihydroxybiaryls,^[^ ^134^ ^]^ as well as desymmetrisation of 2‐hydroxybiaryls^[^ ^135^ ^]^ has extended their utility to the development of molecules with a chiral axis. Deployment of isothiourea‐catalysed enantioselective acylation to planar chiral molecules is in its infancy, with Waser and co‐workers having demonstrated a single example of an acylative KR of [2.2]paracyclophan‐4‐ol with promising but modest selectivity (up to *s* = 20, Figure 1e).^[^ ^115^ ^]^ In this context, the catalytic acylative DKR of planar chiral alcohols represents an underdeveloped area in the field. This manuscript describes the isothiourea‐catalysed acylative KR and DKR of a range of planar chiral paracyclophanols (Figure 1f). At the onset of this study, the following key challenges were recognised. 1) For an effective DKR, the rate of enantioselective acylation must couple with enantiomerization of the racemic planar chiral paracyclophanol, with the assumption that the increased steric hinderance generated upon esterification would prohibit product racemisation. 2) Differentiation of the two paracyclophanol enantiomers using an isothiourea catalyst is a previously underexplored challenge that would require distinguishing between the two potential ether linkage recognition motifs within the substrate. 3) Variation of the *ansa*‐chain length in such macrocycles is known to markedly affect their configurational stability,^[^ ^5^, ^16^, ^17^, ^21^, ^116^, ^117^, ^118^ ^]^ potentially allowing for both KR and DKR processes to be developed. Herein, we describe the successful realisation of this strategy that utilises a commercially available isothiourea catalyst (*R*)‐BTM and common anhydride reagents to promote effective catalytic acylative KR and DKR of a range of planar chiral paracyclophanols (KR, *s* = 50 at 49% conversion; DKR up to 96% yield and 98:2 er).^[^ ^136^ ^]^

## Results and Discussion

### Acylative Kinetic Resolution of Configurationally Stable Planar Chiral Paracyclophanols

To initially verify the feasibility of this enantioselective acylation process, the kinetic resolution (KR) of a configurationally stable racemic macrocyclic phenol **1** with a 12‐membered *ansa*‐chain was explored as a model substrate. Using 5 mol% of the chiral isothiourea catalyst (2*S*,3*R*)‐HyperBTM **3** and isobutyric anhydride as the acyl source in CHCl_3_ or toluene with *^i^* Pr_2_NEt as the co‐base led to effective acylation but moderate selectivity (*s *= 5) (entries 1 and 2, Table 1). Changing to the alternative isothiourea catalyst (*R*)‐BTM **4** in CHCl_3_ led to increased selectivity (*s* = 14, entry 3). Exploration of several co‐bases showed that the use of 1,8‐diazabicyclo[5.4.0]undec‐7‐ene (DBU) led to poor selectivity (*s* = 2, entry 4), while lutidine, 2,6‐di‐*tert*‐butyl pyridine (2,6‐DTBP) and quinaldine all lead to useful levels of selectivity (*s* = 16) at 47%–52% conversion (entries 5–7). Using quinaldine, decreasing the catalyst loading showed that both 5% and 2.5 mol% of (*R*)‐BTM **4** gave equivalent conversion and selectivity (entries 8–9). Optimal selectivity was observed using toluene as the reaction solvent and quinaldine as the base, leading to effective KR of **1** (c = 49, *s* = 50), allowing isolation of alcohol (*S*_p_)‐**1** in 38% yield and 93:7 er, as well as the generation of ester (*R*_p_)‐**2** in 48% yield and 95:5 er (entry 10).

**Table 1:** Optimisation of Acylative Kinetic Resolution Process.

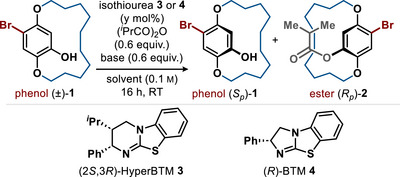					
Entry	Catalyst (y mol%)	Base	Solvent	c^a)^	*s* ^a)^
1	**3** (10)	*^i^* Pr_2_NEt	PhMe	51	5
2	**3** (10)	*^i^* Pr_2_NEt	CHCl_3_	56	3
3	**4** (10)	*^i^* Pr_2_NEt	CHCl_3_	56	14
4	**4** (10)	DBU	CHCl_3_	57	2
5	**4** (10)	Lutidine	CHCl_3_	48	16
6	**4** (10)	2,6‐DTBP	CHCl_3_	47	16
7	**4** (10)	Quinaldine	CHCl_3_	52	16
8	**4** (5)	Quinaldine	CHCl_3_	55	16
9	**4** (2.5)	Quinaldine	CHCl_3_	52	16
10	**4** (5)	Quinaldine	PhMe	49	50

^a)^ Conversion (c) and selectivity factor (*s*) calculated using the enantiomeric ratios of alcohol and ester as measured by HPLC analysis on a chiral stationary phase.^[^ ^119^, ^120^, ^137^ ^]^

*s* values rounded according to estimated errors.^[^ ^138^ ^]^

**Scheme 1:**
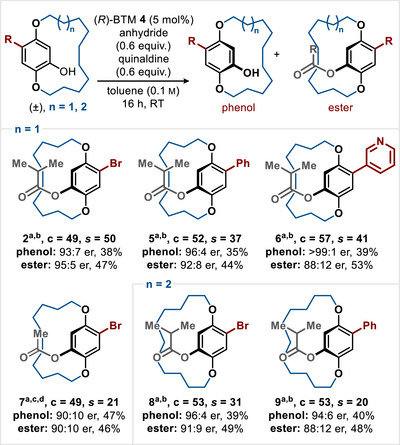
a) Conversion (c) and selectivity factor (*s*) calculated using the enantiomeric ratios of recovered alcohol and ester as measured by HPLC analysis on a chiral stationary phase.^[^ ^119^, ^120^, ^137^ ^]^ *s* values rounded according to estimated errors.^[^ ^138^ ^]^ b) Isobutyric anhydride used; c) Acetic anhydride used; d) Following KR, the phenol and ester products could not be separated, but were resolved on HPLC, yield determined by NMR ratio of the mixture.

With optimised conditions for the KR developed, the scope and limitations of this process were investigated through variation of the aryl substitution pattern and macrocycle ring size (Scheme 1). With a 12‐membered *ansa*‐chain (*n* = 1), incorporation of bromine, phenyl, and 3‐pyridyl substituents led to consistently excellent selectivity in this acylative KR (**2**, **5**, **6**, *s* = 50, 37, and 41, respectively) at 49%–57% conversion. Employing sterically less encumbered acetic anhydride was also feasible, albeit with decreased selectivity (**7**, *s* = 21 at 49% conversion). Further extension to a 13‐membered *ansa*‐chain (*n* = 2) also gave efficient KR, giving bromine‐bearing macrocycle **8** (c = 53, *s* = 31) and phenyl‐substituted analogue **9** (c = 53, *s* = 20). The configurational stability of paracyclophanol **8** (96:4 er) was probed via heating in toluene at 60 °C for >24 h, but no change of the enantiomeric ratio was observed. However, upon heating in toluene at 100 °C, a linear decrease in er with time was observed (to 82:18 er after 43 h), consistent with *k_rac_* = 2.0 × 10^−6^ s^−1^, ΔG_373_ = 32.6 kcal mol^−1^ (see SI for details).^[^ ^136^ ^]^

### Acylative Dynamic Kinetic Resolution of Configurationally Labile Planar Chiral Paracyclophanols

Further investigation considered that increasing the *ansa*‐chain length would allow starting material enantiomerization to occur on a timescale compatible with enantioselective acylation, facilitating an acylative DKR process (Table 2). This process was predicated on the increased steric encumbrance of the ester effectively increasing the barrier to enantiomerization under the reaction conditions. Using paracyclophanol **10** with a 14‐membered *ansa*‐chain as a model system, screening for the potential DKR process was followed. Using 10 mol% of the chiral isothiourea catalyst (2*S*,3*R*)‐HyperBTM **3** and isobutyric anhydride as the acyl source in toluene with *^i^* Pr_2_NEt as the co‐base led to effective acylation but poor selectivity (96% yield, 63:37 er, entry 1, Table 1). Using isothiourea catalyst (*R*)‐BTM **4** under these conditions led to significantly increased selectivity (96% yield, 96:4 er, entry 2). Using (*R*)‐BTM **4**, variation in solvent was tested (entries 3–5), with CHCl_3_ and Et_2_O giving reduced but acceptable selectivity and MeCN leading to significantly reduced product enantiocontrol (54:46 er). Variation of the base was also trialled, with Na_2_CO_3_, quinaldine, 2,6‐lutidine, and 1,4‐diazabicylo[2.2.2]octane (DABCO) all leading to good to excellent product enantiocontrol but reduced product yields (entries 6–9). As the use of *^i^* Pr_2_NEt in toluene led to optimal balance between yield and enantiocontrol, reduction in catalyst loading to 5 and 2.5 mol% using this combination was also tested (entries 10 and 11). While good conversion and high product er were observed using 2.5 mol% catalyst, the optimal reaction conditions were considered to use 5 mol% catalyst, leading to **11** in 92% yield and 97:3 er. Under these reaction conditions, the use of isobutyryl chloride as acylating agent also proved successful, giving (*R*_P_)‐**11** in 82% yield and 92:8 er (entry 12). The use of propionic and benzoic anhydrides also worked well, giving the corresponding esters in good yields and enantioselectivity (88% yield, 92:8 er; 83% yield, 97:3 er, respectively; see SI for further information).

**Table 2:** Optimisation of Acylative Dynamic Kinetic Resolution Process.

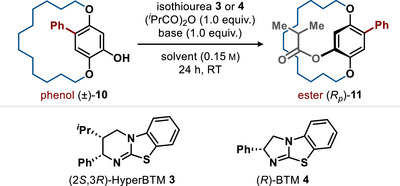					
Entry	Catalyst (mol%)	Base	Solvent	Yield^a)^	er^b)^
1	**3** (10)	*^i^* Pr_2_NEt	PhMe	96	63:37
2	**4** (10)	*^i^* Pr_2_NEt	PhMe	96	96:4
3	**4** (10)	*^i^* Pr_2_NEt	CHCl_3_	92	88:12
4	**4** (10)	*^i^* Pr_2_NEt	Et_2_O	80	93:7
5	**4** (10)	*^i^* Pr_2_NEt	MeCN	92	54:46
6	**4** (10)	Na_2_CO_3_	PhMe	81	95:5
7	**4** (10)	quinaldine	PhMe	78	98:2
8	**4** (10)	2,6‐lutidine	PhMe	80	97:3
9	**4** (10)	DABCO	PhMe	85	93:7
10	**4** (5)	*^i^* Pr_2_NEt	PhMe	92	97:3
11	**4** (2.5)	*^i^* Pr_2_NEt	PhMe	87	96:4
12^c)^	**4** (5)	*^i^* Pr_2_NEt	PhMe	82	92:8

^a)^ Isolated yield.

^b)^ Measured by HPLC analysis on a chiral stationary phase.

^c)^ Isobutyryl chloride used as acylating agent.

**Scheme 2:**
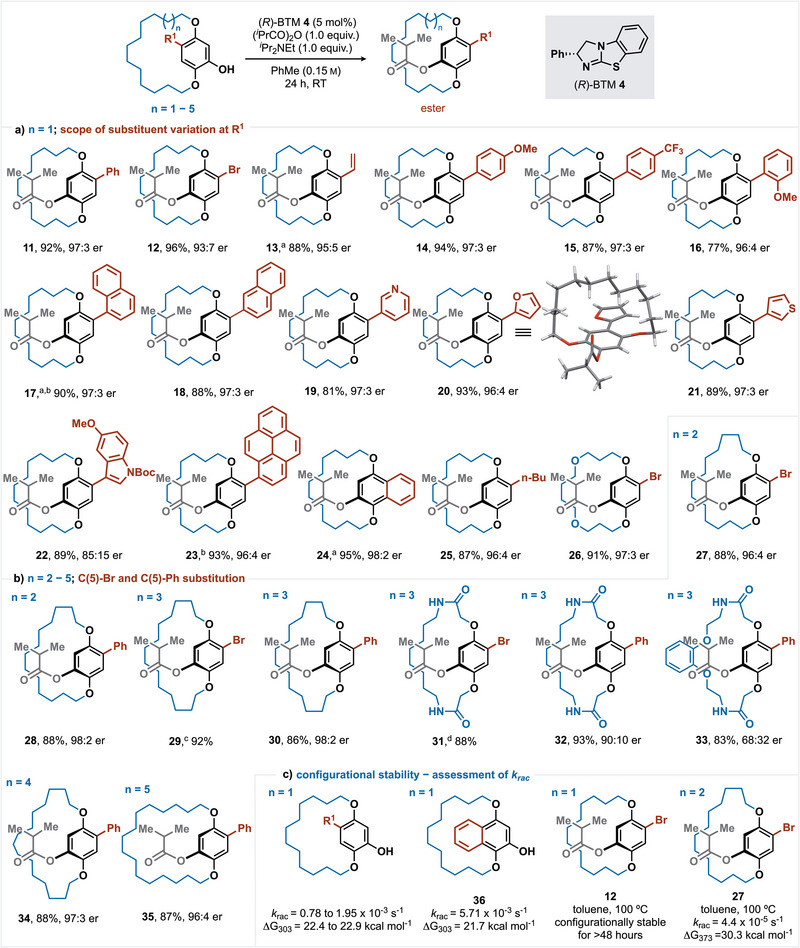
All quoted yields are isolated yields; all ers determined by HPLC analysis on a chiral stationary phase. ^a)^ Reaction performed under an N_2_ atmosphere, ^b)^ Rotamers were observed for **17** and **23** by ^1^H NMR spectroscopy at RT in CDCl_3_ and were confirmed by 1D‐NOESY; product er was assessed by HPLC analysis at 30 °C (see SI for further information). ^c)^ Enantiomers were not resolved by HPLC at 30 °C due to fast interconversion within the HPLC timescale. ^d)^ Dynamic HPLC observed consistent with enantiomerization within the HPLC timescale (*k*_rac_ = 3.92 × 10^−3^ s^−1^, ΔG_303_ = 21.9 kcal mol^−1^).

Under the developed DKR conditions, the scope and limitations of this process were identified. With a 14‐membered *ansa*‐chain, variation of the R^1^‐substituent was probed (Scheme 2a). Incorporation of a bromine substituent was tolerated, giving **12** in 96% yield and 93:7 er. The incorporation of a vinyl substituent was also tested, giving **13** with excellent stereoselectivity (88%, 95:5 er) with the reaction conditions requiring a N_2_ atmosphere to avoid competitive oxidation of the paracyclophanol starting material to the quinone (see SI for further information). The incorporation of aryl and heteroaryl substituents at R^1^ was extensively tested, with the incorporation of electron‐rich 4‐MeOC_6_H_4_ and electron‐deficient 4‐F_3_CC_6_H_4_ substituents tolerated, giving **14** and **15** in good yield and selectivity (94% yield, 97:3 er and 87% yield, 97:3 er, respectively). Incorporation of a 2‐MeOC_6_H_4_ substituent gave **16** in 77% yield (96:4 er). The incorporation of 1‐ and 2‐naphthyl substituents led to **17** and **18** with excellent stereocontrol (90% yield, 95:5 er and 88% yield, 97:3 er, respectively), although for optimal yield and selectivity, **17** had to be generated under N_2_ to avoid competitive paracyclophanol oxidation. Rotamers for ester **17** were observed on the NMR time scale at 20 °C, but product er could be assessed by HPLC at 30 °C (see SI for details), emphasising the dynamic complexity of this molecular class.

Heteroaromatic substituent variation was also trialled, with 3‐pyridyl, 2‐furyl, and 3‐thienyl **19**–**21** generated with excellent stereoselectivity (81% yield, 97:3 er;93% yield, 96:4 er; 89% yield, 97:3 er, respectively). The absolute configuration within 2‐furyl derivative **20** was unambiguously assigned as (*R*_P_) by single crystal X‐ray diffraction,^[^ ^139^ ^]^ with all other structures assigned by analogy. The functionalised N‐Boc‐protected indole variant was also tolerated, giving **22** with reduced stereocontrol (89%, 85:15 er), while the incorporation of a pyrene substituent gave **23** in 93% yield (96:4 er). Similarly to **17**, rotamers were also observed for **23** on the NMR time scale at 20 °C but not via HPLC at 30 °C. The incorporation of a naphthoquinol substituent derivative was also tested, giving **24** with excellent stereoselectivity (95%, 98:2 er), with a protective N_2_ atmosphere again required to minimise alcohol oxidation and achieve high product yield. The incorporation of an alkyl R^1^‐substituent was also explored, giving *^n^* Bu substituted ester **25** with excellent enantioselectivity (87% yield, 96:4 er). Maintaining the length of the *ansa*‐chain but incorporating two oxygen atoms within the linkage was also tolerated, giving **26** in 91% yield and 97:3 er. Interestingly, the paracyclophanol precursor to **26** was not resolved at 30 °C, consistent with enantiomerization being faster than the HPLC timescale. As a comparison, the corresponding starting material to **12** bearing an all hydrocarbon CH_2_ linker showed enantiomer interconversion through dynamic HPLC at 30 °C, consistent with enantiomerization occurring at the HPLC timescale. This emphasises the importance of chain constituents with regard to conformational flexibility and configurational stability. Further variation probed the effect of extension to the *ansa*‐chain length (from 15 to 18 atoms) as well as the incorporation of amide linkages within the chain (Scheme 2b). Chain extension to a 15‐membered *ansa*‐chain with the inclusion of either a bromo‐ or phenyl substituent proved successful, giving **27** (88% yield, 96:4 er) and **28** (88% yield, 98:2 er), respectively. Notably, a further single carbon extension to give a 16‐membered *ansa*‐chain showed that incorporation of a bromo‐substituent gave product **29** in 92% yield that could not be resolved on the HPLC timescale, consistent with configurational instability as observed in other literature examples.^[^ ^76^, ^81^, ^104^, ^105^, ^109^, ^111^ ^]^ However, with a 16‐membered *ansa*‐chain chain length and the incorporation of a phenyl substituent, effective DKR was observed, giving ester **30** in 86% yield and 98:2 er. The incorporation of amide linkages within a 16‐membered *ansa*‐chain length was probed, with a bromo‐substituent giving ester product **31**. Notably, ester **31** showed enantiomer interconversion through dynamic HPLC, in contrast to that observed for **29** (only a single broad peak displayed, consistent with enantiomerization being faster than the HPLC timescale) despite containing the same *ansa*‐chain length and Br substituent. This is consistent with an increased barrier to rotation and enantiomerization for **31** over **29**, consistent with the inclusion of partial double bond character increasing the conformational rigidity of the *ansa*‐chain (*k*_rac_ = 3.92 × 10^−3^ s^−1^, ΔG_303_ = 21.9 kcal mol^−1^). With R^1^ = phenyl effective acylative DKR gave **32** in 93% yield and 90:10 er, while the incorporation of a catechol‐containing linkage gave **33** in 83% yield but moderate enantioselectivity (68:32 er). Further chain extension to the incorporation of 17‐ and 18‐membered *ansa*‐chains with a R^1^ phenyl substituent also showed effective DKR, generating **34** and **35** with high stereoselectivity (88% yield, 97:3 er and 87% yield, 96:4 er, respectively). The configurational stability of a range of paracyclophanol starting materials with a 14‐membered *ansa*‐chain were assessed using dynamic HPLC with the corresponding phenols of esters **11**–**23** giving *k_rac_* values in the range of 0.78–1.95 × 10^−3^ s^−1^, equating to ΔG_303_ = 22.4–22.9 kcal mol^−1^ (see SI for full details).^[^ ^136^ ^]^ Notably, the incorporation of a naphthoquinol substituent derivative within **36** led to increased *k_rac_* (5.71 × 10^−3^ s^−1^, ΔG_303_ = 21.4 kcal mol^−1^). Interestingly, for the ester product (*R*_p_)‐**12** with a 14‐membered *ansa*‐chain, no enantiomerization was observed after heating in toluene at 100 °C for 2 days, indicating that the rotational barrier within **12** is sufficiently large to prevent racemization. Increasing the *ansa*‐chain to a 15‐atom linkage as within **27** led to enantiomerization upon heating in toluene at 100 °C, consistent with *k_rac_* = 4.4 × 10^−5^ s^−1^, ΔG_373_ = 30.3 kcal mol^−1^.

**Scheme 3:**
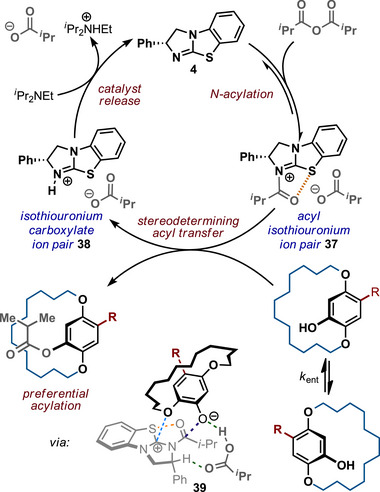
Plausible mechanism for the isothiourea‐catalysed acylative (dynamic) kinetic resolution of paracyclophanols.

A postulated mechanistic scheme for this acylative DKR process is indicated in Scheme 3. Acylation of the isothiourea (*R*)‐BTM **4** with isobutyric anhydride generates an intermediate acyl isothiouronium carboxylate ion pair **37**. For the configurationally labile macrocyclic phenols with 14 to 18‐membered *ansa*‐chains, substrate enantiomerization is fast with respect to acylation, resulting in selective DKR upon acylation. Following the established model developed in previous KR work by ourselves and others,^[^ ^134^, ^140^, ^141^, ^142^, ^143^, ^144^, ^145^, ^146^, ^147^, ^148^, ^149^, ^150^, ^151^, ^152^ ^]^ preferential reaction of this acyl isothiouronium intermediate with the (*R*_P_)‐enantiomer of the paracyclophanol in the stereodetermining step will preferentially generate the enantioenriched ester products. Subsequent reaction of **38** with *^i^* Pr_2_NEt regenerates the free isothiourea BTM catalyst.

By analogy to previous computational studies, the observed selectivity in this acylation can be rationalised using the proposed transition state assembly **39**. This is predicated upon the inclusion of a 1,5‐chalcogen bond (n_O_ → σ*_S‐C_) O•••S interaction between the acyl oxygen and the isothiourea catalyst sulphur that acts as a conformational lock.^[^ ^153^, ^154^, ^155^ ^]^ The isobutyrate counterion deprotonates the phenol, activating it to acylation, while participating in non‐classical H‐bonding to the acylated catalyst's benzylic hydrogen.^[^ ^143^, ^144^, ^156^, ^157^, ^158^, ^159^, ^160^ ^]^ To deliver high enantioselectivity, a donor substrate motif is needed to promote enantiorecognition through interaction with the positively charged acylated isothiouronium intermediate **38**.^[^ ^70^ ^]^ Recognised enantiorecognition motifs in isothiourea‐catalyzed acylations include aryl,^[^ ^161^, ^162^, ^163^, ^164^, ^165^ ^]^ heteroaryl,^[^ ^141^ ^]^ alkenyl,^[^ ^163^ ^]^ alkynyl,^[^ ^164^ ^]^ heteroatom,^[^ ^151^ ^]^ C═O,^[^ ^145^, ^166^ ^]^ CF_2_,^[^ ^149^ ^]^ and P═O substituents.^[^ ^146^ ^]^ Utilising the O‐atom adjacent to the phenol within the cyclophane in this capacity (blue dotted line) directs the *ansa*‐chain away from the stereodirecting unit of the acylated catalyst, leading to the observed selectivity.

## Conclusion

In this manuscript, an effective isothiourea [(*R*)‐BTM)] catalysed acylative kinetic (KR) and dynamic kinetic resolution (DKR) approach that leads to the efficient synthesis of a range of planar chiral paracyclophanols with excellent levels of enantioselectivity is demonstrated. Efficient KR of stable planar chiral macrocyclic paracyclophanols with 12‐ and 13‐membered *ansa*‐chains is demonstrated (6 examples, *s* = up to 50). Extension of the *ansa*‐chain to include 14 to 18‐membered substituted substrates allows their effective acylative DKR, generating the desired products with excellent enantioselectivity (25 examples, up to 95% yield and 98:2 er). In a wider context, the configurational lability of the starting materials and products has been systematically evaluated, with the effect of *ansa*‐chain length and constitution investigated. Furthermore, esterification of the paracyclophanol starting material leads to a significant increase in the barrier to enantiomerization resulting in enhanced configurational stability of the ester products. The simplicity of this synthetic approach significantly broadens the methods available of the preparation of these valuable materials. The further application of this methodology to alternative planar chiral substrates and bioactive natural products is currently underway within our laboratory.

## Supporting Information

The authors have cited additional references within the Supporting Information.^[^ ^167^, ^168^, ^169^, ^170^, ^171^, ^172^, ^173^, ^174^, ^175^, ^176^, ^177^, ^178^, ^179^, ^180^, ^181^, ^182^, ^183^, ^184^, ^185^, ^186^, ^187^, ^188^, ^189^, ^190^ ^]^

## Conflict of Interests

The authors declare no conflict of interest.

## Supporting information



Supporting Information

Supporting Information

## Data Availability

The research data supporting this publication can be accessed from “Isothiourea‐Catalysed Acylative Kinetic and Dynamic Resolution of Planar Chiral Macrocycles”. Pure ID: 316 046 332. University of St Andrews Research Portal: https://doi.org/10.17630/70b47d2f‐941b‐40b6‐9645‐036d99456577.
