# Supplementary material for: Isothiourea‐Catalysed Acylative Kinetic and Dynamic Kinetic Resolution of Planar Chiral Paracyclophanols
Source: Angew Chem Int Ed Engl. 2025 Jun 23;64(31):e202507126. doi: 10.1002/anie.202507126 (PMC12304874; doi:10.1002/anie.202507126)

Isothiourea-Catalysed Acylative Kinetic and Dynamic Kinetic Resolution of Planar Chiral Paracyclophanols

Zhanyu Zhou,[a] Kevin Kasten,[a] Aidan P. McKay,[a] David B. Cordes,[a] Andrew D. Smith*[a]

[a] Mr. Z. Zhou, Dr. K. Kasten, Dr. A. P. McKay, Dr. D. B. Cordes, Prof. Dr. A. D. Smith
EaStCHEM, School of Chemistry, University of St Andrews
St Andrews, Fife, KY16 9ST, (UK)
E-mail: ads10@st-andrews.ac.uk

References cited in published manuscript: [1],[2],[3],[4],[5],[6],[7],[8],[9],[10],[11],[12],[13],[14],[15],[16],[17],[18],[19],[20],[21],[22],[23],[24],[25],[26],[27],[28],[29],[30],[31],[32],[33],[34],[35],[36],[37],[38],[39],[40],[41],[42],[43],[44],[45]

Contents

1 General Experimental 3
2 Syntheses 6
2.1 Synthesis of 2,5-dihydroxybenzaldehyde derivatives 6
Synthesis of 2,5-dihydroxy-[1,1'-biphenyl]-4-carbaldehyde **S2** 6
2.2 Synthesis of dibromide derivatives **S3-S9** 7
Synthesis of di-*tert*-butyl 3,3'-(butane-1,4-diylbis(oxy))dipropionate **S3** 7
Synthesis of 3,3'-(butane-1,4-diylbis(oxy))bis(propan-1-ol) **S4** 7
Synthesis of 1,4-bis(3-bromopropoxy)butane **S5** 7
Synthesis of 1,8-bis(α-bromoacetylamino)octane **S6** 8
Synthesis of *O,O'*-bis(2-(*tert*-butoxycarbonylamino)ethyl)catechol **S7** 8
Synthesis of *O,O'*-bis(2-aminoethyl)catechol **S8** 9
Synthesis of *O,O'*-bis(2-(α-bromoacetylamino)ethyl)catechol **S9** 9
2.3 General Procedure A: Synthesis of macrocycle 1,4-dioxa-aldehyde derivatives **S10-S17** 10
2.4 General Procedure B: Synthesis of macrocycle 2,5-dioxa-aldehyde derivatives **S20-S37** 16
Synthesis of methyl 1,4-dihydroxy-2-naphthoate **S40** 29
Synthesis of methyl 2,15-dioxa-1(1,4)-naphthalenacyclopentadecaphane-1^2-carboxylate **S41** 29
Synthesis of 2,15-dioxa-1(1,4)-naphthalenacyclopentadecaphane-1^2-ylmethanol **S42** 30
Synthesis of 2,15-dioxa-1(1,4)-naphthalenacyclopentadecaphane-1^2-carbaldehyde **S43** 30
2.5 General Procedure C: Synthesis of macrocycle 2,5-dioxa-phenol derivatives **1, 10, S44-S52, S57-S67, S69** 31
2.6 General Procedure D: Synthesis of macrocycle 2,5-dioxa-phenol derivatives **36, S53-S56** 31
2.7 General Procedure E: Kinetic resolution of macrocycle phenol derivatives 52
2.8 General Procedure F: Dynamic kinetic resolution of macrocycle phenol derivatives 52
2.8.1 Macrocycle phenols products and macrocycle esters products 53
3 Racemization experiment 89
3.1 Dynamic HPLC 89
3.2 Monitoring the rate of racemization 90
Table S1. Racemization Kinetics of (*Sp*)-**S45**. 91
Table S2. Racemization Kinetics of (*Rp*)-**12**. 92
Table S3. Racemization Kinetics of (*Rp*)-**27**. 93
4 Single crystal X-ray diffraction data 95
5 Reference 97
Appendix I: ^{1}H, ^{13}C{^{1}H}, 2D ^{1}H COSY, 2D ^{1}H–^{13}C HSQC and 2D ^{1}H–^{13}C HMBC, NOESY, NMR Spectra 102

1 General Experimental

Reactions involving moisture sensitive reagents were carried out in flame-dried glassware under an inert atmosphere (Ar or N_2) using standard vacuum line techniques. Anhydrous solvents (Et_2O, CH_2Cl_2, THF and PhMe) were obtained after passing through an alumina column (MBraun SPS-800) or purchased in a sealed bottled under inert atmosphere. Organometallic reagents were titrated before use according to literature procedures.[46] Room temperature (r.t.) refers to 18 ± 3 °C, Petrol refers to petroleum ether with the boiling range of 40 – 60 °C, brine refers to saturated aqueous sodium chloride solution, ether refers to diethylether (Et_2O). All chemicals and solvents used were purchased by pertinent brands (Sigma Aldrich, Alfa Aesar, Acros, Apollo Scientific, TCI, STREM) and used without further purification unless stated. For reactions conducted during the day following cooling baths were applied: 0 °C (ice/water), −10 °C (ice/acetone), −20 °C (ice/NaCl), −45 °C (CO_2(s) or N_2(l)/MeCN), −60 °C (CO_2(s) or N_2(l)/$CHCl_3$) and −78 °C (CO_2(s)/acetone). Temperatures of 0 °C to −78 °C for overnight reactions were obtained using an immersion cooler (HAAKE EK 90). Reactions involving heating were performed using DrySyn blocks or oil baths and a contact thermocouple. Under reduced pressure refers to the use of either a Büchi Rotavapor R-200 with a Büchi V491 heating Bath and Büchi V-800 vacuum controller, a Büchi Rotavapor R-210 with a Büchi V-491 heating bath and Büchi V-850 vacuum controller, a Heidolph Laborota 4001 with vacuum controller, an IKA RV10 rotary evaporator with an IKA HB10 heating bath and ILMVAC vacuum controller, or an IKA RV10 rotary evaporator with an IKA HB10 heating bath and Vacuubrand CVC3000 vacuum controller. Rotary evaporator condensers are fitted to Julabo FL601 Recirculating Coolers filled with ethylene glycol and set to −6 °C.

Analytical thin layer chromatography (TLC)[47] was performed on pre-coated aluminium plates (Kieselgel 60 F_{254} silica) plates purchased from Merck. Visualisation was achieved using ultraviolet light (254 nm) and staining with aqueous $KMnO_4$ or ethanolic vanillin solution followed by heating. Flash column chromatography was performed in glass columns fitted with porosity 3 sintered discs over Silica gel 60 (0.043 – 0.060 mm) using standard techniques as reported in literature with the solvent system stated.[48] Automated chromatography was performed on a Biotage® Selekt™ SEL-2SV with a 200 – 400 nm UV-detector using the method stated and Biotage® Sfär™ Silica HC D or Biotage® Sfär™ Silica D columns.

HPLC analyses were obtained on either a Shimadzu HPLC consisting of a DGU-20A5 degassing unit, LC-20AT liquid chromatography pump, SIL-20AHT autosampler, CMB-20A communications bus module, SPD-M20A diode array detector and a CTO-20A column oven or a Shimadzu HPLC consisting of a DGU-20A5R degassing unit, LC-20AD liquid chromatography pump, SIL-20AHT autosampler, SPD-20A UV/Vis detector

and a CTO-20A column oven. Separation was achieved using either DAICEL CHIRALCEL OD-H and OJ-H columns or DAICEL CHIRALPAK AD-H, AS-H, IA, IB, IC and ID columns using the method stated. HPLC traces of enantiomerically enriched compounds were compared with authentic racemic spectra. Racemic compounds were synthesised under analogous reaction conditions using achiral or racemic catalysts where necessary.

Optical rotations were determined using a Perkin Elmer Precisely/Model-341 Polarimeter with a Na/Hal lamp (Na D line, 589 nm) at 20 °C.[49]

Infrared spectra were recorded on a Shimadzu IRAffinity-1 Fourier transform IR spectrophotometer fitted with a Specac Quest ATR accessory (diamond puck). Spectra were recorded of either thin films or solids, with characteristic absorption wavenumbers (ν_{max}) reported in cm^{-1}.[50]

^{1}H, ^{13}C, ^{19}F and ^{32}P nuclear magnetic resonance (NMR) spectra were recorded with Bruker Avance™ 300 Cryomagnet with a BBFO probe, Bruker Avance II™ 400 Ultrashield with a BBFO probe, Bruker Avance™ 500 Ultrashield with a SmartProbe BBFO+ probe or Bruker Avance III™ 500 Ascend™ with a CryoProbe Prodigy BBO probe using deuterated solvents ($CDCl_3$, CD_2Cl_2, D_2O, CD_3OD, CD_3CN, $(CD_3)_2SO$, $(CD_3)_2CO$, $C_6D_5CD_3$) purchased from Sigma-Aldrich. Chemical shifts (δ) are quoted in ppm and referenced to residual solvent signals reported in literature.[51] $^{13}C\{^{1}H\}$ and $^{19}F\{^{1}H\}$ spectra were acquired using a proton broadband decoupling sequence. ^{13}C were recorded with DEPTQ or UDEFT sequences. Couplings were indicated by the use of conventional agreed abbreviations: s (singlet), d (doublet), t (triplet), q (quartet), m (multiplet), dd (doublet of doublets), td (triplet of doublets), etc.[52] The abbreviation Ar denotes aromatic and app denotes apparent. NMR peak assignments were confirmed using 2D ^{1}H correlated spectroscopy (COSY), ^{1}D selective ^{1}H nuclear Overhauser effect spectroscopy (NOESY), 2D ^{1}H–^{13}C heteronuclear multiple-bond correlation spectroscopy (HMBC), and 2D ^{1}H–^{13}C heteronuclear single quantum coherence (HSQC) where necessary. For analysis of NMR-spectra MestReNova and tools therein were used.

Melting points were recorded on an Electrothermal 9100 melting point apparatus and are not corrected; (dec) refers to decomposition.

Mass spectrometry (m/z) data were acquired using ThermoFisher Exactive Orbitrap mass spectrometer or Micromass GCT (TOF) mass spectrometer with solids probe. Ionisation techniques used are indicated for each compound. Values are quoted as a ratio of mass to charge (m/z) in Daltons [Da].[53]

Common chemical abbreviations were used to indicate chemical groups or environments such as Ph (phenyl), *Ar* (aromatic, not confuse with Argon), Bn (benzyl), Et (ethyl), Me (methyl).[54] To indicate atoms

numbering schemes are displayed with the spectrum and deviate from IUPAC numbering for clarity. For names and numbering concerning stereodiscriptors IUPAC nomenclature was applied.[1, 49, 55]

Authentic racemic samples were prepared in an analogous fashion using 4-(Dimethylamino)pyridine.

Selectivity factors were calculated using the following equations, with all enantiomeric excesses (ee) determined by HPLC analysis on a chiral stationary phase.[38-39]

$$s = \frac{\ln[(1 - conv)(1 - ee_{alcohol})]}{\ln[(1 - conv)(1 + ee_{alcohol})]}$$

where both ee and conv are given as between 0 and 1

$$conv = \frac{ee_{alcohol}}{ee_{alcohol} + ee_{ester}}$$

2 Syntheses

2.1 Synthesis of 2,5-dihydroxybenzaldehyde derivatives

Synthesis of 1-bromo-2,5-dihydroxybenzaldehyde S1

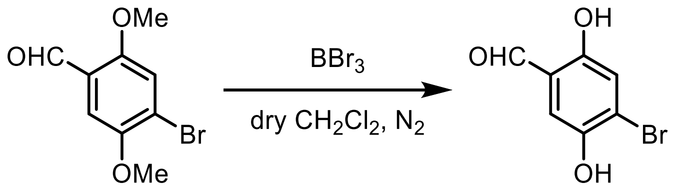

S1

Following the method described by Zhao *et al.*,[26f] to a solution of 4-bromo-2,5-dimethoxybenzaldehyde (4.90 g, 20.00 mmol) in dry CH_2Cl_2 (100 ml) was added BBr_3 (1 M, 24.00 mmol, 24 ml) dropwise at 0 °C. The reaction mixture was stirred for overnight and quenched carefully with water. The aqueous phase was extracted with CH_2Cl_2 (3 × 100 ml). The combined organic phase was washed with brine and dried over anhydrous Na_2SO_4. The solvent was removed under reduced pressure, and the residue was purified by column chromatography (10% EtOAc/Toluene) to afford **S1** as a yellow solid (3.69 g, 85%); mp 117–122 °C (EtOAc/Hexane); ν_{max} (film)/cm^{-1} 3358 (O–H), 2874 (C–H), 1645 (C=O), 1470 (C=C), 1140 (C–O), 866 (C–Br); ^{1}H NMR (500 MHz, $CDCl_3$) δ_H: 3.93 (1H, s, ArC(2)-O*H*), 7.21 (1H, s, ArC(6)*H*), 7.22 (1H, s, ArC(3)*H*), 9.85 (1H, s, ArC(5)-O*H*), 10.59 (1H, s, C*H*O). Data in agreement with the literature.[26f]

Synthesis of 2,5-dihydroxy-[1,1'-biphenyl]-4-carbaldehyde S2

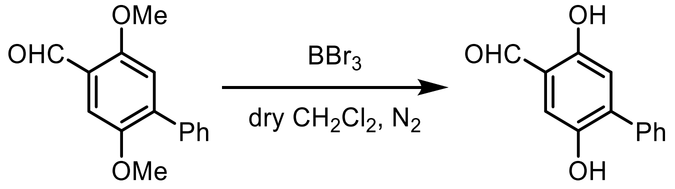

S2

Following the method described by Zhao *et al.*,[26f] to a solution of 2,5-dimethoxy-[1,1'-biphenyl]-4-carbaldehyde (0.93 g, 3.90 mmol) in dry CH_2Cl_2 (50 ml) was added BBr_3 (1 M, 6.90 mmol, 6.9 ml) dropwise at 0 °C. The reaction mixture was stirred for overnight and quenched carefully with water. The aqueous phase was extracted with CH_2Cl_2 (3 × 100 ml). The combined organic phase was washed with brine and dried over anhydrous Na_2SO_4. The solvent was removed under reduced pressure, and the residue was purified by column chromatography (10% EtOAc/Toluene) to afford **S2** as a yellow solid (0.76 g, 91%); mp 89–91 °C (EtOAc/Hexane); ν_{max} (film)/cm^{-1} 3379 (O–H), 1622 (C=O), 1362 (C=C), 1169 (C–O); ^{1}H NMR (500 MHz,

CDCl3) δ_H: 4.99 (1H, s, ArC(5)-O*H*), 6.94 (1H, s, ArC(3)*H*), 7.18 (1H, s, ArC(6)*H*), 7.44-7.59 (5H, s, Ar′C(2,3,4,5,6)*H*), 9.89 (1H, s, ArC(2)-O*H*), 10.61 (1H, s, C*H*O). Data in agreement with the literature.[26f]

2.2 Synthesis of dibromide derivatives **S3-S9**

Synthesis of di-*tert*-butyl 3,3'-(butane-1,4-diylbis(oxy))dipropionate S3

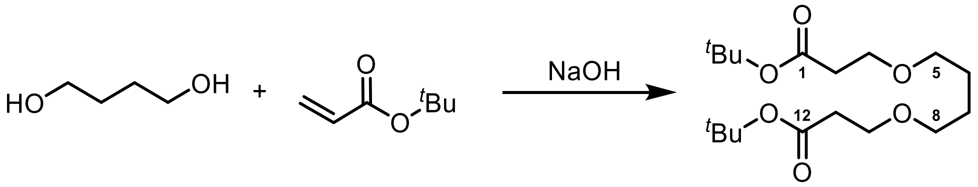

To a mixture of 1,4-butanediol (4.50 g, 50.00 mmol) and *tert*-butyl acrylate (19.23 g, 150.00 mmol) was slowly added NaOH (0.20 g, 5.00 mmol) at 0 °C. The mixture was stirred at this temperature for 10 min and then warmed to room temperature with stirring continued for 36 h. The solution was diluted with water (50 ml) and extracted with EtOAc (3 × 100 ml). The combined organic phase was washed with brine and dried over anhydrous Na_2SO_4. The solvent was removed under reduced pressure, and the residue was purified by column chromatography (10% EtOAc/Petrol ether) to afford colourless oil **S3** (6.30 g, 36%); ν_{max} (film)/cm^{-1} 2978 (C–H), 2931 (C–H), 2868 (C–H), 1730 (C=O), 1367 (C–H), 1159 (C–O), 1111 (C–O); ^{1}H NMR (500 MHz, CDCl3) δ_H: 1.45 (18H, s, 2 × C(C*H*3)3), 1.61 (4H, p, *J* 3.1, C(6,7)*H*2), 2.48 (4H, t, *J* 6.5, C(2,11)*H*2), 3.44 (4H, p, *J* 3.0, OC(5,8)*H*2, 3.65 (4H, t, *J* 6.5, C(3,10)*H*2). Data in agreement with the literature.[56]

Synthesis of 3,3'-(butane-1,4-diylbis(oxy))bis(propan-1-ol) S4

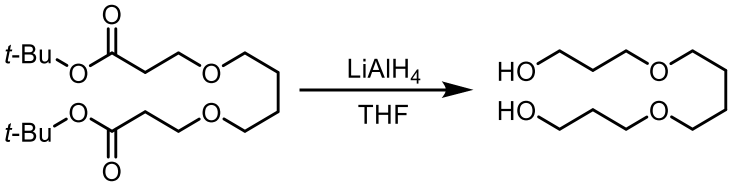

To a solution of **S3** (5.20 g, 15.00 mmol) in dry THF (100 ml) was slowly added $LiAlH_4$ (2.4 M, 15.6 ml, 37.50 mmol) at 0 °C. The mixture was stirred at this temperature for 10 min and was then warmed to room temperature with stirring continued for 6 h. The solution was quenched with saturated NH_4Cl (50 ml) and extracted with EtOAc (3 × 100 ml). The combined organic phase was washed with brine and dried over anhydrous Na_2SO_4, filtered, and concentrated in *vacuo*. The crude product was used for next step without any purification.

Synthesis of 1,4-bis(3-bromopropoxy)butane S5

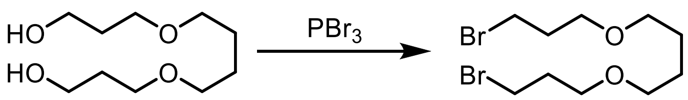

To **S4** (1.56 g, 7.60 mmol) was slowly added PBr_3 (1.4 ml, 15.20 mmol) at 0 °C under N_2 protective atmosphere. The mixture was stirred at this temperature for 10 min and was then heated to 60 °C with stirring continued for 16 h. The reaction was quenched with ice-cold water and the mixture was extracted with EtOAc (3 × 20 ml). The combined organic phases were washed with saturated $NaHCO_3$, dried over anhydrous Na_2SO_4, filtered, and concentrated in *vacuo*. The crude product was used for next step without any purification.

Synthesis of 1,8-bis(α-bromoacetylamino)octane S6

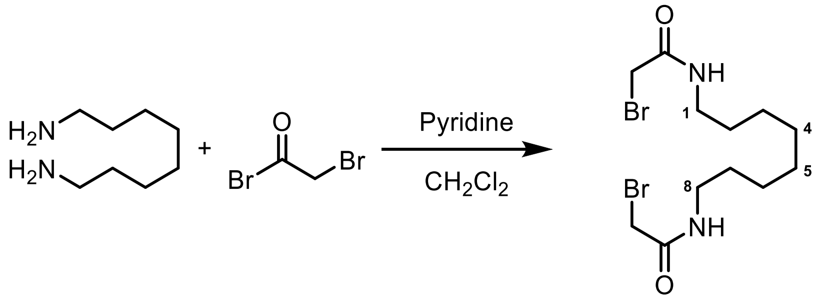

Following the method described by Beer *et al.*,[57] to a solution of 1,8-diaminooctane (0.84 ml, 5.00 mmol) and pyridine (1.0 ml, 12.5 mmol) in anhydrous CH_2Cl_2 (50 ml) was slowly added bromoacetyl bromide (1.3 ml, 15.00 mmol) under N_2 protective atmosphere at 0 °C. The mixture was stirred at this temperature for 40 min and was then warmed to room temperature with stirring continued for 1 h. The reaction was quenched with water and extracted with CH_2Cl_2 (3 × 20 ml). The combined organic phases were washed with brine, dried over anhydrous Na_2SO_4, filtered, and concentrated in *vacuo*. The residue was purified by column chromatography (30% EtOAc/Petrol ether) to afford **S6** as a brown solid (1.00 g, 54%); mp 78–82 °C (EtOAc/Hexane); ν_{max} (film)/cm^{-1} 3294 (N–H), 2932 (C–H), 2855 (C–H), 1647 (C=O), 1541 (C=O), 1202 (C–N), 945 (C–Br); ^{1}H NMR (500 MHz, $CDCl_3$) δ_H: 1.35 (8H, m, C(3,4,5,6)H_2), 1.56 (4H, p, *J* 7.2, C(2,7)H_2), 3.31 (4H, td, *J* 7.4, *J* 5.9, C(1,8)H_2), 3.91 (4H, s, 2 × CH_2Br), 6.51 (2H, s (br), 2 × N*H*). Data in agreement with the literature.[58]

Synthesis of *O,O′*-bis(2-(*tert*-butoxycarbonylamino)ethyl)catechol S7

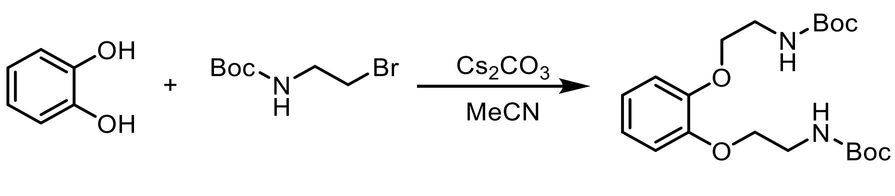

Catechol (1.65 g, 15.00 mmol) and Cs_2CO_3 (12.22 g, 37.50 mmol) in CH_3CN (80 ml) were stirred at room temperature for 30 min. 2-(*tert*-Butyloxycarbonylamino)ethyl bromide (1.3 ml, 15.00 mmol) was slowly added to the solution. After the addition the solution was heated to 80 °C for 8 h. After cooling to room temperature, EtOAc (100 ml) and water (150 ml) were added and the mixture was extracted with EtOAc (3 × 50 ml). The combined organic phases were washed with brine, dried over anhydrous Na_2SO_4, filtered, and

concentrated in *vacuo*. The mixture of the bis and mono alkylated products was stirred with 5% NaOH (100 ml) overnight. White precipitate was formed which was filtered and washed with H_2O, the crude product was used for next step without purification.

Synthesis of *O,O′*-bis(2-aminoethyl)catechol S8

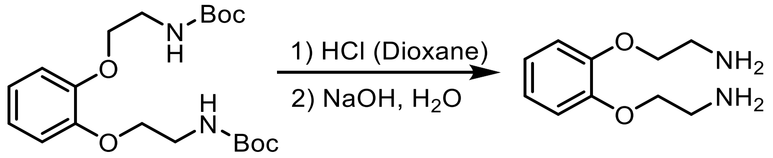

To a solution of **S7** (4.9 g, 12.36 mmol) in 30 ml of dioxane was slowly added hydrogen chloride solution (4 M in 1,4-dioxane, 98.90 mmol, 24.7 ml) at 0 °C over 25 minutes. The reaction mixture was allowed to warm to room temperature and stirring was continued for 24 hours. After all the starting material had been consumed, reaction mixture was concentrated in *vacuo*. The residual HCl was removed by dissolving the residue in MeOH and subsequent rotary evaporation (3 × 50 ml). The residual white solid was triturated with Et_2O and stirred for 3 hours and the HCl salt was collected by filtration. To obtain the free base, the HCl salt was dissolved in H_2O (30 ml) and the pH was set to 13 with solid NaOH. Water was subsequently evaporated. The solid was redissolved in CH_3CN, filtered, and concentrated in *vacuo*. The white solid crude product was used for next step without any purification.

Synthesis of *O,O′*-bis(2-(α-bromoacetylamino)ethyl)catechol S9

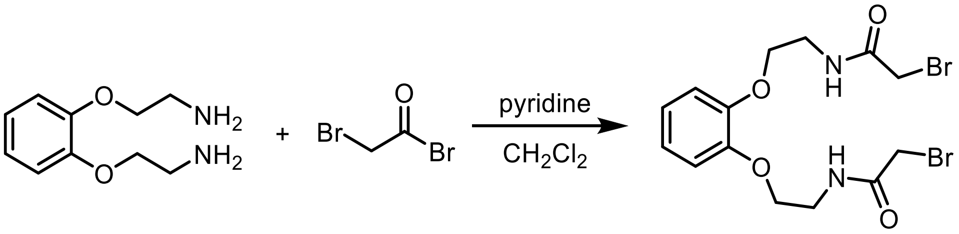

Following the method described by Beer *et al.*,[57] to a solution of **S8** (0.98 g, 5.00 mmol) and pyridine (1.0 ml, 12.5 mmol) in dry CH_2Cl_2 (50 ml) was slowly added bromoacetyl bromide (1.3 ml, 15.00 mmol) under N_2 protective atmosphere at 0 °C. The mixture was stirred at this temperature for 40 min and was then warm to room temperature with stirring continued for 1 h. The reaction was quenched with water and extracted with CH_2Cl_2 (3 × 20 ml). The combined organic phases were washed with brine, dried over anhydrous Na_2SO_4, filtered, and concentrated in *vacuo*. The residue was purified by column chromatography (30% EtOAc/Petrol ether) to afford **S9** as a white solid (1.66 g, 76%); mp 69–72 °C (EtOAc/Hexane); ν_{max} (film)/cm^{-1} 3298 (N–H), 3273 (N–H), 2926 (C–H), 2877 (C–H), 1651 (C=O), 1508 (C=C), 1209 (C–N), 1125 (C–O), 895 (C–Br); ^{1}H NMR (500 MHz, $CDCl_3$) δ_H: 3.74 (4H, q, *J* 5.4, 2 × C*H*$_2$), 3.94 (4H, s, 2 × C*H*$_2$Br), 4.14 (4H, t, *J* 5.1, 2 × C*H*$_2$), 6.90-7.05

(4H, m, ArC(3,4,5,6)*H*), 7.20 (2H, s (br), 2×N*H*); **^{13}C{1H} NMR** (126 MHz, $CDCl_3$) δ_C: 29.2 (2 × $BrCH_2$), 39.9 (2 × NCH_2), 68.1 (2 × OCH_2), 115.2 (2 × Ar*C*(2,5)H), 122.4 (2 × Ar*C*(3,4)H), 148.6 (2 × Ar*C*(1,2)), 166.1 (2 × CONH); HRMS (APCI$^+$) $C_{14}H_{18}Br_2N_2O_4$ ([M+Na]$^+$) requires 460.95051, found 460.9503 (–0.46 ppm).

2.3 General Procedure A: Synthesis of macrocycle 1,4-dioxa-aldehyde derivatives **S10-S17**

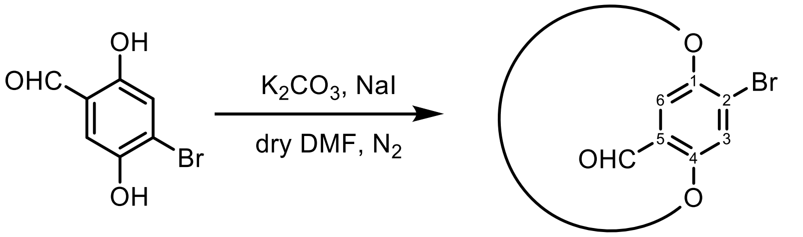

Following the method described by Zhao *et al.*,[26f] *C*-formylquinol derivatives (1.0 equiv.) and 1,ω-dibromide derivatives (1.0 equiv.) in DMF (0.4 M) were slowly added to a suspension of K_2CO_3 (2.5 equiv.) and NaI (0.11 equiv.) in DMF (0.05 M) at 140 °C by syringe pump over 30 minutes. The mixture was stirred overnight at 140 °C. The solvent was removed in *vacuo*. EtOAc (50 ml) was added to the residue which was then filtered and washed with EtOAc (100 ml). The combined organic layers were washed with water and brine and dried over Na_2SO_4, filtered, and concentrated in *vacuo*. The residue was purified by column chromatography (5% EtOAc/Petrol ether) to afford 1,4-dioxa-aldehyde derivatives **S10-S17**.

Synthesis of 1^2-bromo-1^5-formyl-2,13-dioxa-1(1,4)-benzenacyclotridecaphane S10

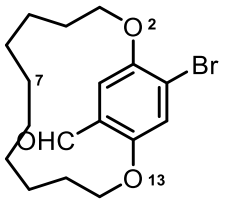

The title compound was prepared according to *General Procedure A* from **S1** (1.74 g, 8.00 mmol) and 1,10-dibromodecane (2.4 g, 8.00 mmol) in DMF (20 ml), K_2CO_3 (2.76 g, 20.00 mmol) and NaI (131.9 mg, 0.88 mmol) in DMF (150 ml) to afford **S10** as a yellow solid (1.31 g, 46%); mp 42–45 °C (EtOAc/Hexane); ν_{max} (film)/cm^{-1} 2922 (C–H), 2853 (C–H), 1676 (C=O), 1472 (C=C), 1190 (C–O), 887 (C–Br); ^{1}H NMR (500 MHz, $CDCl_3$) δ_H: 0.60-0.78 (4H, m, 2 × C*H*$_2$), 0.87-1.02 (2H, m, C*H*$_2$), 1.03-1.17 (4H, m, 2 × C*H*$_2$), 1.20-1.30 (2H, m, C*H*$_2$), 1.54-1.84 (4H, m, 2 × C*H*$_2$), 4.28 (2H, ddd, *J* 12.1, *J* 6.3, *J* 3.6, OC*H*$_2$), 4.47 (2H, ddd, *J* 12.1, *J* 5.9, *J* 3.9, OC*H*$_2$), 7.35 (1H, s, ArC(6)*H*), 7.45 (1H, s, ArC(3)*H*), 10.43 (1H, s, C*H*O). Data in agreement with the literature.[26f]

Synthesis of 1^2-bromo-1^5-formyl-2,14-dioxa-1(1,4)-benzenacyclotetradecaphane S11

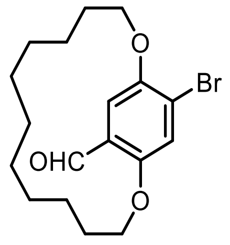

The title compound was prepared according to *General Procedure A* from **S1** (0.87 g, 4.00 mmol) and 1,11-dibromoundecane (1.26 g, 4.00 mmol) in DMF (10 ml), K_2CO_3 (1.38 g, 10.00 mmol) and NaI (65.9 mg, 0.44 mmol) in DMF (60 ml) to afford **S11** as a white solid (0.62 g, 42%); mp 40–43 °C (EtOAc/Hexane); ν_{max} (film)/cm^{-1} 2922 (C–H), 2853 (C–H), 1678 (C=O), 1474 (C=C), 1194 (C–O), 881 (C–Br); ^{1}H NMR (500 MHz, $CDCl_3$) δ_H: 0.66-0.81 (2H, m, C*H*$_2$), 0.84-1.09 (6H, m, 3 × C*H*$_2$), 1.12-1.30 (6H, m, 3 × C*H*$_2$), 1.60-1.83 (4H, m, 2 × C*H*$_2$), 4.22-4.51 (4H, m, 2 × OC*H*$_2$), 7.35 (1H, s, ArC(6)*H*), 7.46 (1H, s, ArC(3)*H*), 10.45 (1H, s, C*H*O). Data in agreement with the literature.[26f]

Synthesis of 1^2-bromo-1^5-formyl-2,15-dioxa-1(1,4)-benzenacyclotetradecaphane S12

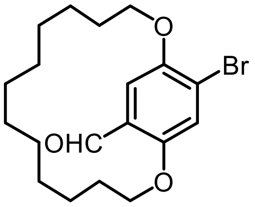

The title compound was prepared according to *General Procedure A* from **S1** (1.74 g, 8.00 mmol) and 1,12-dibromododecane (2.62 g, 8.00 mmol) in DMF (20 ml), K_2CO_3 (2.76 g, 20.00 mmol) and NaI (131.9 mg, 0.88 mmol) in DMF (150 ml) to afford **S12** as a white solid (1.59 g, 52%); mp 68–72 °C (EtOAc/Hexane); ν_{max} (film)/cm^{-1} 2924 (C–H), 2851 (C–H), 1678 (C=O), 1474 (C=C), 1198 (C–O), 1049 (C–O); ^{1}H NMR (500 MHz, $CDCl_3$) δ_H: 0.84-1.01 (8H, m, 4 × C*H*$_2$), 1.05-1.26 (4H, m, 2 × C*H*$_2$), 1.29-1.44 (4H, m, 2 × C*H*$_2$), 1.58-1.72 (2H, m, C*H*$_2$), 1.74-1.94 (2H, m, C*H*$_2$), 4.28 (2H, ddd, *J* 12.3, *J* 8.5, *J* 3.9, OC*H*$_2$), 4.43 (2H, ddd, *J* 12.3, *J* 6.2, *J* 4.2, OC*H*$_2$), 7.32 (1H, s, ArC(6)*H*), 7.42 (1H, s, ArC(3)*H*), 10.45 (1H, s, C*H*O). Data in agreement with the literature.[26i]

Synthesis of 1^2-bromo-1^5-formyl-2,6,11,15-tetraoxa-1(1,4)-benzenacyclopentadecaphane S13

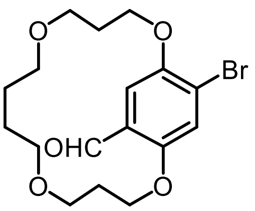

The title compound was prepared according to *General Procedure A* from **S1** (0.47 g, 2.16 mmol) and **S5** (0.72 g, 2.16 mmol) in DMF (5 ml), (0.75 g, 5.40 mmol) and NaI (35.6 mg, 0.24 mmol) in DMF (55 ml) to afford **S13** as a colourless oil (0.29 g, 35%); ν_{max} (film)/cm^{-1} 2916 (C–H), 2860 (C–H), 1678 (C=O), 1477 (C=C), 1198 (C–O), 1111 (C–O); ^{1}H NMR (500 MHz, $CDCl_3$) δ_H: 0.95-1.13 (4H, m, 2 × C(8,9)*H*$_2$), 1.87-2.01 (2H, m, C(4)*H*$_2$), 2.01-2.12 (2H, m, C(13)*H*$_2$), 2.98 (1H, ddd, *J* 9.7, *J* 6.9, OC*H*^{A}H^B), 3.07 (1H, ddd *J* 9.8, *J* 6.6, OC*H*$_2$), 3.29 (2H, m, OC*H*$_2$), 3.36-3.49 (3H, m, OC*H*$_2$, OC*H*^{A}H^B), 3.61 (1H, ddd, *J* 10.2, *J* 9.0, *J* 3.4, OCH^A*H*^{B}), 4.31-4.39 (2H, m, ArC(1)-OC*H*$_2$), 4.42 (1H, ddd, *J* 10.6, *J* 10.5, *J* 2.9, ArC(4)-OC*H*^{A}H^B), 4.49 (1H, ddd, *J* 10.7, *J* 10.6, *J* 2.7, ArC(4)-OCH^A*H*^{B}), 7.32 (1H, s, ArC(6)*H*), 7.41 (1H, s, ArC(3)*H*), 10.44 (C*H*O); ^{13}C{1H} NMR (126 MHz, $CDCl_3$) δ_C: 25.7 (*C*H$_2$), 25.8 (*C*H$_2$), 30.1 (*C*H$_2$), 30.6 (*C*H$_2$), 65.8 (O*C*H$_2$), 66.2 (O*C*H$_2$), 68.4 (ArC(4)-O*C*H$_2$), 68.4 (ArC(1)-O*C*H$_2$), 70.8 (O*C*H$_2$), 71.1 (O*C*H$_2$), 113.4 (Ar*C*(6)H), 121.0 (Ar*C*(3)H), 121.4 (Ar*C*(4)), 125.4 (Ar*C*(1)), 151.5 (Ar*C*(5)), 157.4 (Ar*C*(2)), 189.0 (*C*HO); HRMS (APCI$^+$) $C_{17}H_{24}O_5Br$ ([M]$^+$) requires 387.08016, found 387.0799 (–0.78 ppm).

Synthesis of 1^2-bromo-1^5-formyl-2,16-dioxa-1(1,4)-benzenacyclotetradecaphane S14

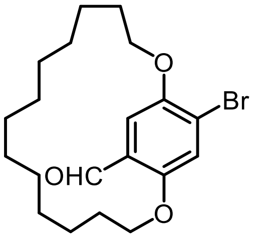

The title compound was prepared according to *General Procedure A* from **S1** (1.09 g, 5.00 mmol) and 1,13-dibromotridecane (1.71 g, 5.00 mmol) in DMF (15 ml), (1.73 g, 12.50 mmol) and NaI (82.3 mg, 0.55 mmol) in DMF (100 ml) to afford **S14** as an off-white solid (0.94 g, 47%); mp 53–55 °C (EtOAc/Hexane); ν_{max} (film)/cm^{-1} 2922 (C–H), 2849 (C–H), 1676 (C=O), 1464 (C=C), 1198 (C–O), 1042 (C–O); ^{1}H NMR (500 MHz, $CDCl_3$) δ_H: 0.92-1.16 (11H, m, 5 × C*H*$_2$, C*H*^{A}H^B), 1.25-1.46 (7H, m, 3 × C*H*$_2$, CH^A*H*^{B}), 1.60-1.88 (4H, m, 2 × C*H*$_2$), 4.25 (2H, m, OC*H*$_2$), 4.37 (2H, m, OC*H*$_2$), 7.31 (1H, s, ArC(6)*H*), 7.40 (1H, s, ArC(3)*H*), 10.45 (1H, s, C*H*O). Data in agreement with the literature.[26i]

Synthesis of 1^2-bromo-1^5-formyl-2,17-dioxa-1(1,4)-benzenacyclotetradecaphane S15

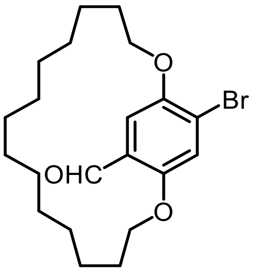

The title compound was prepared according to *General Procedure A* from **S1** (1.09 g, 5.00 mmol) and 1,14-dibromotetradecane (1.78 g, 5.00 mmol) in DMF (15 ml), K_2CO_3 (1.73 g, 12.50 mmol) and NaI (82.3 mg, 0.55 mmol) in DMF (100 ml) to afford **S15** as a white solid (0.92 g, 45%); mp 40–42 °C (EtOAc/Hexane); ν_{max} (film)/cm^{-1} 2922 (C–H), 2853 (C–H), 1682 (C=O), 1462 (C=C), 1200 (C–O), 1026 (C–O), 878 (C–Br); ^{1}H NMR (500 MHz, $CDCl_3$) δ_H: 0.96-1.15 (10H, m, 5 × C*H*$_2$), 1.21-1.41 (8H, m, 4 × C*H*$_2$), 1.44-1.62 (2H, m, C*H*$_2$), 1.69-1.85 (4H, m, 2 × C*H*$_2$), 4.17-4.35 (4H, m, 2 × OC*H*$_2$), 7.29 (1H, s, ArC(6)*H*), 7.38 (1H, s, ArC(6)*H*), 10.45 (1H, s, C*H*O). Data in agreement with the literature.[26i]

Synthesis of 1^2-bromo-1^5-formyl-2,18-dioxa-1(1,4)-benzenacyclotetradecaphane S16

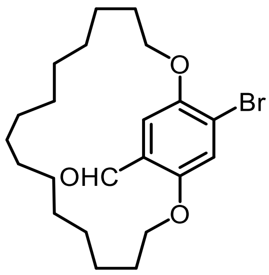

The title compound was prepared according to *General Procedure A* from **S1** (0.25 g, 1.16 mmol) and 1,15-dibromopentadecane (0.43 g, 1.16 mmol) in DMF (5 ml), K_2CO_3 (0.40 g, 2.90 mmol) and NaI (19.1 mg, 0.13 mmol) in DMF (30 ml) to afford **S16** as a white solid (0.18 g, 36%); mp 38–40 °C (EtOAc/Hexane); ν_{max} (film)/cm^{-1} 2922 (C–H), 2851 (C–H), 1680 (C=O), 1466 (C=C), 1196 (C–O), 714 (C–Br); ^{1}H NMR (500 MHz, $CDCl_3$) δ_H: 0.98-1.22 (14H, m, 7 × C*H*$_2$), 1.24-1.36 (4H, m, 2 × C*H*$_2$), 1.37-1.55 (4H, m, 2 × C*H*$_2$), 1.68-1.87 (4H, m, 2 × C*H*$_2$), 4.24 (4H, m, 2 × OC*H*$_2$), 7.29 (1H, s, ArC(6)*H*), 7.39 (1H, s, ArC(3)*H*), 10.45 (1H, s, C*H*O). Data in agreement with the literature.[26f]

Synthesis of 1^2-bromo-1^5-formyl-2,19-dioxa-1(1,4)-benzenacyclotetradecaphane S17

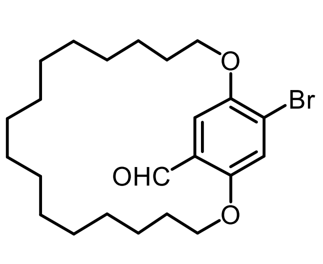

The title compound was prepared according to *General Procedure A* from **S1** (0.47 g, 2.16 mmol) and 1,16-dibromohexadecane (0.83 g, 2.16 mmol) in DMF (5 ml), (0.75 g, 5.40 mmol) and NaI (35.6 mg, 0.24 mmol) in DMF (55 ml) to afford **S17** as a white solid (0.37 g, 39%); mp 46–48 °C (EtOAc/Hexane); ν_{max} (film)/cm^{-1} 2920 (C–H), 2849 (C–H), 1676 (C=O), 1464 (C=C), 1204 (C–O), 1032 (C–O), 735 (C–Br); ^{1}H NMR (500 MHz, $CDCl_3$)

δ_H: 1.01-1.10 (7H, m, 3 × C*H*2, C*H*^{A}H^B), 1.13-1.37 (13H, m, 6 × C*H*2, CH^A*H*^{B}), 1.50 (4H, m, 2 × C*H*2), 1.79 (4H, m, 2 × C*H*2), 4.19 (4H, m, 2 × OC*H*2), 7.27 (1H, s, ArC(6)*H*), 7.36 (1H, s, ArC(3)*H*), 10.45 (1H, s, C*H*O). Data in agreement with the literature.[26i]

Synthesis of 1^2-bromo-1^5-formyl-4,15-dioxo-2,17-dioxa-5,14-diaza-1(1,4)-benzenacycloheptadecaphane S18

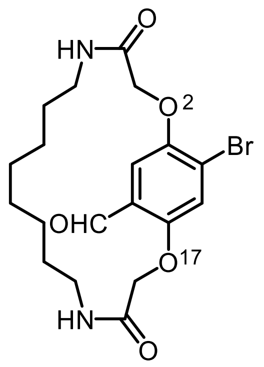

To a solution of 2,5-dihydroxybenzaldehyde **S1** (0.43 g, 2.00 mmol) and NaH (0.20 g, 5.00 mmol, 60% dispersion in mineral oil) in anhydrous THF (50 ml) was slowly added **S18** (0.77 g, 2.00 mmol) at 0 °C. The mixture was heated to 55 °C and stirred at this temperature overnight. After cooling to room temperature, saturated NH_4Cl (5 ml) was added, and the reaction mixture was diluted with water (30 ml) and extracted with EtOAc (3 × 50 ml). The combined organic layers were washed with water and brine, dried over Na_2SO_4, filtered, and concentrated in *vacuo*. The residue was purified by column chromatography (20%-35% Aceton/CH_2Cl_2) to afford **S18** as an off-white solid (0.18 g, 21%); mp 158–162 °C (EtOAc/Hexane); ν_{max} (film)/cm^{-1} 3283 (N–H), 2931 (C–H), 2853 (C–H), 1682 (C=O), 1647 (C=C), 1198 (C–O), 1043 (C–O); ^{1}H NMR (500 MHz, $CDCl_3$) δ_H: 0.88-1.04 (4H, m, 2 × C*H*2), 1.04-1.20 (4H, m, 2 × C*H*2), 1.35-1.47 (2H, m, C*H*2), 1.49-1.65 (2H, m, C*H*2), 2.97 (2H, m, NC*H*2), 3.65 (1H, dtd, *J* 13.4, *J* 8.3, *J* 3.5, NC*H*^{A}H^B), 3.79 (1H, dtd, *J* 13.5, *J* 8.1, *J* 3.5, NCH^A*H*^{B}), 4.59 (1H, d, *J* 16.2, ArC(1)-OC*H*^{A}H^B), 4.69-4.80 (2H, m, ArC(4)-OC*H*2), 4.82 (1H, d, *J* 16.2, ArC(1)-OCH^A*H*^{B}), 6.43 (1H, d, *J* 4.5, *C*(4)ONH), 6.54 (1H, d, *J* 4.7, (*C*(15)ONH), 7.25 (1H, s, ArC(6)*H*), 7.39 (1H, s, ArC(3)*H*), 10.35 (1H, s, C*H*O); ^{13}C{1H} NMR (126 MHz, $CDCl_3$) δ_C: 26.4 (*C*H2), 26.5 (*C*H2), 28.5 (*C*H2), 28.7 (*C*H2), 29.7 (*C*H2), 29.8 (*C*H2), 39.2 (NCH2), 39.3 (NCH2), 68.8 (ArC(1)-OCH2), 68.9 (ArC(4)-OCH2), 113.2 (Ar*C*H(6)), 118.9 (Ar*C*H(3)), 120.3 (ArC(4)), 124.8 (Ar*C*(1)), 149.3 (Ar*C*(5)), 153.9 (Ar*C*(2)), 167.4 (*C*(4)ONH), 167.4 (*C*(15)ONH), 187.7 (CHO); HRMS (APCI$^+$) $C_{19}H_{25}O_5N_2Br$ ([M+Na]$^+$) requires 463.08391, found 463.0836 (–0.62 ppm).

Synthesis of 1^2-formyl-4,14-dioxo-1^5-phenyl-5,13-diaza-2,8,10,16-tetraoxa-1(1,2),9(1,4)-dibenzena-cyclohexadecaphane S19

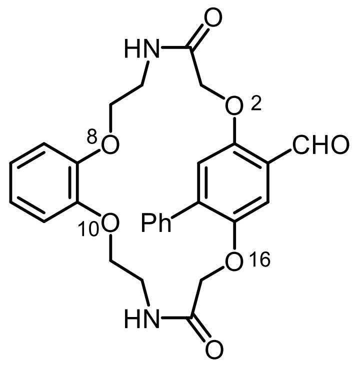

To a solution of **S2** (0.43 g, 2.00 mmol) and NaH (0.20 g, 5.00 mmol, 60% dispersion in mineral oil) in anyhdrous THF (50 ml) was slowly added **S19** (0.88 g, 2.00 mmol) at 0 °C. The mixture was heated to 55 °C and stirred at this temperature overnight. After cooling to room temperature, saturated NH_4Cl (5 ml) was added, the reaction mixture was diluted with water (30 ml) and extracted with EtOAc (3 × 50 ml). The combined organic layers were washed with water and brine, dried over Na_2SO_4, filtered, and concentrated in *vacuo*. The residue was purified by column chromatography (20%-35% Aceton/CH_2Cl_2) to afford **S19** as white solid (0.20 g, 20%); mp 170–174 °C (EtOAc/Hexane); ν_{max} (film)/cm^{-1} 3424 (N–H), 3375 (N–H), 2928 (C–H), 2876 (C–H), 1682 (C=O), 1661 (C=O), 1504 (C=O), 1252 (C–N), 1221 (C–N), 1194 (C–O), 1043 (C–O); ^{1}H NMR (500 MHz, $CDCl_3$) δ_H: 3.06-3.17 (1H, m, NC*H*^{A}H^B), 3.42-3.50 (1H, m, NCH^A*H*^{B}), 3.50-3.57 (1H, m, NC*H*^{A}H^B), 3.84 (1H, ddd, *J* 11.4, *J* 6.1 *J* 2.0, NCH^A*H*^{B}), 3.98-4.05 (1H, m, ArOC*H*^{A}H^B), 4.05-4.16 (2H, m, ArOC*H*$_2$), 4.41-4.50 (1H, m, ArOCH^A*H*^{B}), 4.62 (1H, d, *J* 16.8, OC*H*^{A}H^B), 4.73 (1H, d, *J* 16.9, OCH^A*H*^{B}), 4.85 (1H, d, *J* 16.7, OC*H*^{A}H^B), 4.89 (1H, d, *J* 17.0, CH^A*H*^{B}), 5.91 (1H, t, *J* 5.5, C(14)ON*H*), 6.70 (1H, dd, *J* 7.9, *J* 1.5, ArC*H*), 6.86 (1H, t, *J* 5.8, C(4)ON*H*), 6.88-7.04 (6H, m, 6×ArC*H*), 7.24 (1H, tt, *J* 7.5, *J* 1.2, ArC*H*), 7.36 (1H, dt, *J* 6.8, *J* 1.3, ArC(1^6)*H*), 7.63 (1H, s, ArC(1^3)*H*), 10.47 (1H, s, C*H*O); ^{13}C{1H} NMR (126 MHz, $CDCl_3$) δ_C: 38.6 (NCH_2), 39.8 (NCH_2), 66.5 (OCH_2), 67.0 (OCH_2), 67.7 (OCH_2), 69.3 (OCH_2), 112.1 (ArCH), 112.2 (ArCH), 114.4 (ArCH), 115.1 (ArCH), 121.3 (ArCH), 121.3 (ArCH), 124.6 (ArC), 128.3 (2×ArCH), 128.7 (ArCH), 129.7 (2×ArCH), 135.5 (ArC), 140.2 (ArC), 147.4 (ArC), 148.0 (ArC), 149.2 (ArC(ArC(1^2)), 154.1 (ArC(1^5)), 168.4 (C(14)ON*H*), 168.5 (C(4)ON*H*), 188.0 (*C*HO); HRMS (APCI$^+$) $C_{26}H_{27}O_{10}N$ ([M]$^+$) requires 513.16295, found 513.1628 (–0.26 ppm).

2.4 General Procedure B: Synthesis of macrocycle 2,5-dioxa-aldehyde derivatives **S20-S37**

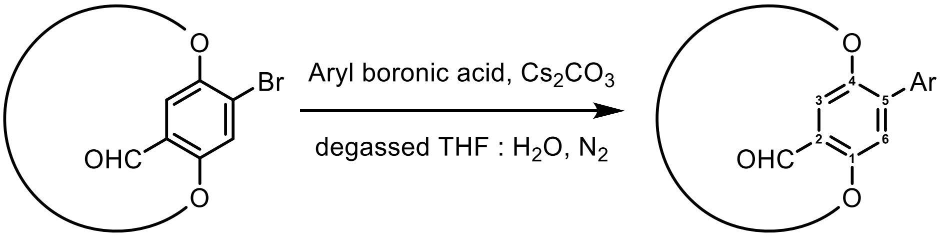

Following the method described by Zhao *et al.*,[26f] bromocyclophane derivatives (1.0 equiv.), aryl boronic acid (3.0 equiv.), $Pd(PPh_3)_4$ (0.05 equiv.) and Cs_2CO_3 (3.0 equiv.) were added to a dried flask. The flask was evacuated and backfilled with N_2 three times. Degassed THF : H_2O (5:1, 0.1 M) was added and the mixture was stirred at 50 °C for 24 h. After cooling to room temperature, the mixture was diluted with water (20 ml) and extracted with EtOAc (3 × 40 ml). The combined organic layers were washed with water and brine, dried over Na_2SO_4, filtered, and concentrated in *vacuo*. The residue was purified by column chromatography (2%-5% EtOAc/Petrol ether) to afford the cross-coupling product **S20-S37**.

Synthesis of 1^2-formyl-1^5-phenyl-2,13-dioxa-1(1,4)-benzenacyclotridecaphane S20

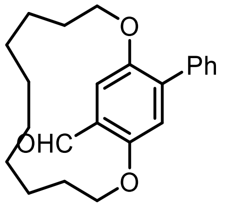

The title compound was prepared according to *General Procedure B* from **S10** (1.07 g, 3.00 mmol), phenyl boronic acid (1.10 g, 9.00 mmol), $Pd(PPh_3)_4$ (0.17 g, 0.15 mmol) and Cs_2CO_3 (2.93 g, 9.00 mmol) in degassed THF : H_2O (5:1, 30 ml) to afford **S20** as a colourless oil (1.02 g, 97%); ν_{max} (film)/cm^{-1} 2926 (C–H), 2855 (C–H), 1678 (C=O), 1477 (C=C), 1410 (C=C), 1180 (C–O); **^{1}H NMR** (500 MHz, $CDCl_3$) δ_H: 0.66-0.99 (7H, m, 3 × CH_2, CH^AH^B), 1.01-1.40 (6H, m, 3 × CH_2), 1.61-1.74 (2H, m, CH_2), 1.88-2.02 (1H, m, CH^AH^B), 3.92 (1H, ddd, *J* 12.3, *J* 8.7, *J* 3.8, ArC(4)-OCH^AH^B), 4.26 (1H, ddd, *J* 12.2, *J* 5.9, *J* 3.9, ArC(4)-OCH^AH^B), 4.32 (1H, ddd, *J* 12.3, *J* 8.7, *J* 3.7, ArC(1)-OCH^AH^B), 4.58 (1H, ddd, *J* 12.2, *J* 6.1, *J* 3.7, ArC(1)-OCH^AH^B), 7.11 (1H, s, ArC(6)*H*), 7.39-7.44 (1H, m, ArC(5)-PhC(4)*H*), 7.44-7.51 (2H, m, ArC(5)-PhC(3,5)*H*), 7.57 (1H, s, ArC(3)*H*), 7.58-7.62 (2H, m, ArC(5)-PhC(2,6)*H*), 10.51 (1H, s, C*H*O). Data in agreement with the literature.[26f]

Synthesis of 1^5-phenyl-2,14-dioxa-1(1,4)-benzenacyclotridecaphane-1^2-carbaldehyde S21

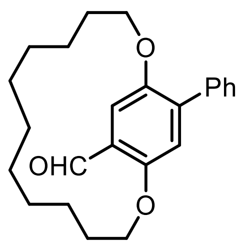

The title compound was prepared according to *General Procedure B* from **S11** (1.11 g, 3.00 mmol), phenyl boronic acid (1.10 g, 9.00 mmol), $Pd(PPh_3)_4$ (0.17 g, 0.15 mmol) and Cs_2CO_3 (2.93 g, 9.00 mmol) in degassed THF : H_2O (5:1, 30 ml) to afford **S21** as a colourless oil (1.08 g, 98%); ν_{max} (film)/cm^{-1} 2924 (C–H), 2853 (C–H), 1678 (C=O), 1477 (C=C), 1408 (C=C), 1184 (C–O); **^{1}H NMR** (500 MHz, $CDCl_3$) δ_H: 0.75-0.83 (2H, m, C*H*$_2$), 0.85-1.15 (9H, m, 4 × C*H*$_2$, C*H*^{A}H^B), 1.19-1.36 (3H, m, C*H*$_2$, CH^A*H*^{B}), 1.38-1.49 (1H, m, C*H*^{A}H^B), 1.50-1.62 (1H, m, CH^A*H*^{B}), 1.71-1.89 (2H, m, C*H*$_2$), 4.07 (1H, ddd, *J* 12.0, *J* 7.3, *J* 4.0, ArC(4)-OC*H*^{A}H^B), 4.33 (2H, ddt, *J* 10.7, *J* 7.4, *J* 3.8, ArC(4)-OCH^A*H*^{B}, ArC(1)-OC*H*^{A}H^B), 4.53 (1H, ddd, *J* 11.9, *J* 7.1, *J* 3.7, ArC(1)-OCH^A*H*^{B}), 7.09 (1H, s, ArC(6)*H*), 7.38-7.43 (1H, m, ArC(5)-PhC(4)*H*), 7.43-7.49 (2H, m, ArC(5)-PhC(3,5)*H*), 7.55 (1H, s, ArC(3)*H*), 7.55-7.61 (2H, m, ArC(5)-PhC(2,6)*H*), 10.53 (1H, s, C*H*O); **^{13}C{1H} NMR** (126 MHz, $CDCl_3$) δ_C: 24.1 (*C*H$_2$), 24.4 (*C*H$_2$), 27.2 (*C*H$_2$), 27.2 (*C*H$_2$), 27.3 (*C*H$_2$), 28.2 (*C*H$_2$), 28.4 (*C*H$_2$), 28.6 (2×*C*H$_2$), 69.5 (ArC(4)-O*C*H$_2$), 69.5 (ArC(5)-O*C*H$_2$), 115.2 (Ar*C*(6)H), 119.0 (Ar*C*(3)H), 125.8 (Ar*C*(2)), 127.9 (ArC(5)-Ph*C*(4)H), 128.1 (ArC(5)-Ph*C*(3,5)H), 129.4 (ArC(5)-Ph*C*(2,6)H), 137.7 (ArC(5)-Ph*C*(1)), 140.4 (Ar*C*(5), 150.3 (Ar*C*(1)), 156.3 (Ar*C*(4)), 189.5 (*C*HO); HRMS (APCI$^+$) $C_{24}H_{31}O_3$ ([M+Na]$^+$) requires 389.20872, found 389.2087 (–0.55 ppm).

Synthesis of 1^5-phenyl-2,15-dioxa-1(1,4)-benzenacyclotridecaphane-1^2-carbaldehyde S22

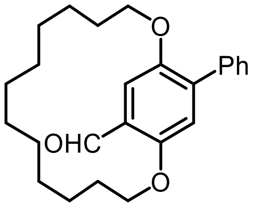

The title compound was prepared according to *General Procedure B* from **S12** (1.15 g, 3.00 mmol), phenyl boronic acid (1.10 g, 9.00 mmol), $Pd(PPh_3)_4$ (0.17 g, 0.15 mmol) and Cs_2CO_3 (2.93 g, 9.00 mmol) in degassed THF : H_2O (5:1, 30 ml) to afford **S22** as a colourless oil (1.12 g, 98%); ν_{max} (film)/cm^{-1} 2924 (C–H), 2853 (C–H), 1678 (C=O), 1477 (C=C), 1410 (C=C), 1190 (C–O); **^{1}H NMR** (500 MHz, $CDCl_3$) δ_H: 0.86-1.34 (17H, m, 7 × C*H*$_2$), 1.37-1.49 (3H, m, C*H*$_2$, C*H*^{A}H^B), 1.61-1.82 (2H, m, C*H*$_2$), 1.88-2.01 (1H, m, CH^A*H*^{B}), 4.04 (1H, ddd, *J* 11.9, *J* 8.6, *J* 4.1, ArC(4)-OC*H*^{A}H^B), 4.23-4.38 (2H, m, ArC(4)-OCH^A*H*^{B}, ArC(1)-OC*H*^{A}H^B), 4.51 (1H, ddd, *J* 11.8, *J* 6.0, *J* 4.0, ArC(1)-OCH^A*H*^{B}), 7.04 (1H, s, ArC(6)*H*), 7.40-7.44 (1H, m, ArC(5)-PhC(4)*H*), 7.45-7.49 (2H, m, ArC(5)-

PhC(3,5)*H*), 7.54-7.57 (3H, m, ArC(5)-PhC(2,6)*H*, ArC(3)*H*), 10.53 (1H, s, C*H*O). Data in agreement with the literature.[26i]

Synthesis of 1^5-(4-methoxyphenyl)-2,15-dioxa-1(1,4)-benzenacyclopentadecaphane-1^2-carbaldehyde S23

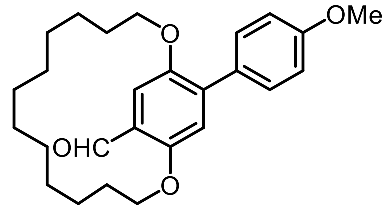

The title compound was prepared according to *General Procedure B* from **S12** (0.5 g, 1.31 mmol), 4-methoxypheny phenyl boronic acid (0.60 g, 3.93 mmol), $Pd(PPh_3)_4$ (75.7 mg, 0.065 mmol) and Cs_2CO_3 (1.28 g, 3.93 mmol) in degassed THF : H_2O (5:1, 12 ml) to afford **S23** as a colourless oil (0.52 g, 97%); ν_{max} (film)/cm^{-1} 2924 (C–H), 2853 (C–H), 1676 (C=O), 1481 (C=C), 1246 (C–O), 1177 (C–O); **^{1}H NMR** (500 MHz, $CDCl_3$) δ_H: 0.88-0.95 (5H, m, 2 × C*H*$_2$, C*H*^{A}H^B), 0.97-1.13 (5H, m, 2 × C*H*$_2$, CH^A*H*^{B}), 1.15-1.32 (4H, m, 2 × C*H*$_2$), 1.37-1.48 (3H, m, C*H*$_2$, C*H*^{A}H^B), 1.63-1.79 (2H, m, C*H*$_2$), 1.88-2.01 (1H, m, CH^A*H*^{B}), 3.89 (3H, s, OC*H*$_3$), 4.04 (1H, ddd, *J* 11.8, *J* 8.5, *J* 4.2, ArC(4)-OC*H*^{A}H^B), 4.27 (1H, ddd, *J* 12.1, *J* 8.6, *J* 3.9, ArC(4)-OCH^A*H*^{B}), 4.33 (1H, ddd, *J* 11.9, *J* 6.0, *J* 4.3, ArC(1)-OC*H*^{A}H^B), 4.50 (1H, ddd, *J* 11.7, *J* 6.0, *J* 4.0, ArC(1)-OCH^A*H*^{B}), 6.96-7.01 (2H, m, ArC(5)-ArC(3,5)*H*), 7.02 (1H, s, ArC(6)*H*), 7.48 (1H, s, ArC(3)*H*), 7.50-7.56 (2H, m, ArC(5)-ArC(2,6)*H*), 10.50 (1H, s, C*H*O); **^{13}C{1H} NMR** (126 MHz, $CDCl_3$) δ_C: 23.8 (*C*H$_2$), 24.1 (*C*H$_2$), 26.7 (*C*H$_2$), 27.1 (*C*H$_2$), 27.4 (*C*H$_2$), 27.6 (*C*H$_2$), 27.9 (*C*H$_2$), 28.1 (*C*H$_2$), 28.5 (*C*H$_2$), 28.6 (*C*H$_2$), 55.3 (O*C*H$_3$), 68.6 (ArC(4)-O*C*H$_2$), 68.7 (ArC(1)-O*C*H$_2$), 113.6 (Ar*C*(6)H), 113.6 (ArC(5)-Ar*C*(3,5)H), 117.5 (Ar*C*(3)H), 124.7 (Ar*C*(2)), 130.1 (ArC(5)-Ar*C*(1)), 130.6 (ArC(5)-Ar*C*(2,6)H), 139.6 (Ar*C*(5)), 149.2 (Ar*C*(1)), 155.3 (Ar*C*(4)), 159.4 (ArC(5)-Ar*C*(4)), 189.4 (*C*HO); HRMS (APCI$^+$) $C_{26}H_{34}O_4$ ([M+Na]$^+$) requires 433.23493, found 433.2345 (–0.89 ppm).

Synthesis of 1^5-(4-(trifluoromethyl)phenyl)-2,15-dioxa-1(1,4)-benzenacyclopentadecaphane-1^2-carbaldehyde S24

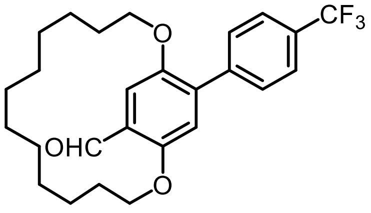

The title compound was prepared according to *General Procedure B* from **S12** (0.5 g, 1.31 mmol), 4-(trifluoromethyl) benzeneboronic acid (0.75 g, 3.93 mmol), $Pd(PPh_3)_4$ (75.7 mg, 0.065 mmol) and Cs_2CO_3 (1.28 g, 3.93 mmol) in degassed THF : H_2O (5:1, 12 ml) to afford **S24** as an off-white solid (0.56 g, 96%); mp 53–54 °C (EtOAc/Hexane); ν_{max} (film)/cm^{-1} 2924 (C–H), 2853 (C–H), 1674 (C=O), 1481 (C=C), 1109 (C–O), 839 (C–F); **^{1}H NMR** (500 MHz, $CDCl_3$) δ_H: 0.90-1.11 (10H, m, 2 × C*H*$_2$), 1.15-1.33 (4H, m, 2 × C*H*$_2$), 1.35-1.49 (3H, m, C*H*$_2$, C*H*^{A}H^B), 1.63-1.81 (2H, m, C*H*$_2$), 1.87-2.00 (1H, m, CH^A*H*^{B}), 4.06 (1H, ddd, *J* 12.2, *J* 8.8, *J* 4.0, ArC(1)-OC*H*^{A}H^B), 4.29 (1H, ddd, *J* 12.1, *J* 8.7, *J* 3.8, ArC(4)-OC*H*^{A}H^B), 4.38 (1H, ddd, *J* 12.0, *J* 5.9, *J* 4.1, ArC(1)-OCH^A*H*^{B}), 4.50 (1H, ddd, *J* 11.9, *J* 6.0, *J* 3.9, ArC(4)-OCH^A*H*^{B}), 7.03 (1H, s, ArC(6)*H*), 7.52 (1H, s, ArC(3)*H*), 7.67 (2H, d, *J* 8.2, ArC(5)-Ar(2,6)*H*), 7.72 (2H, d, *J* 8.4, ArC(5)-Ar(3,5)*H*), 10.53 (C*H*O); **^{13}C{1H} NMR** (126 MHz, $CDCl_3$) δ_C: 23.7 (*C*H$_2$), 24.1 (*C*H$_2$), 26.5 (*C*H$_2$), 27.1 (*C*H$_2$), 27.4 (*C*H$_2$), 27.6 (*C*H$_2$), 27.9 (*C*H$_2$), 28.1 (*C*H$_2$), 28.5 (*C*H$_2$), 28.6 (*C*H$_2$), 68.5 (ArC(1)-OCH$_2$), 68.8 (ArC(4)-OCH$_2$), 113.3 (Ar*C*(6)H), 117.9 (Ar*C*(3)H), 124.2 (q, $^1J_{CF}$ 272.3, ArC(5)-ArCF$_3$), 125.1 (q, $^3J_{CF}$ 4.0, 3.8, ArC(5)-Ar*C*(3,5)H), 125.7 (Ar*C*(2)), 129.7 (ArC(5)-Ar*C*(2,6)H), 129.9 (q, $^2J_{CF}$ 32.4, ArC(5)-Ar*C*(4)), 137.9 (Ar*C*(5)), 141.3 (ArC(5)-Ar*C*(1)), 149.0 (Ar*C*(1)), 155.1 (Ar*C*(4)), 189.3 (CHO); **^{19}F{1H} NMR** (470 MHz, $CDCl_3$) δ_F: –62.53 (CF$_3$); HRMS (APCI$^+$) $C_{26}H_{31}O_3F_3$ ([M+Na]$^+$) requires 471.21175, found 471.2112 (–1.25 ppm).

Synthesis of 1^5-(2-methoxyphenyl)-2,15-dioxa-1(1,4)-benzenacyclopentadecaphane-1^2-carbaldehyde S25

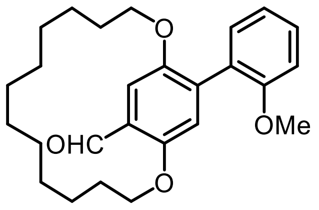

The title compound was prepared according to *General Procedure B* from **S12** (0.5 g, 1.31 mmol), 2-methoxypheny phenyl boronic acid (0.60 g, 3.93 mmol), $Pd(PPh_3)_4$ (75.7 mg, 0.065 mmol) and Cs_2CO_3 (1.28 g, 3.93 mmol) in degassed THF : H_2O (5:1, 12 ml) to afford **S25** as a yellow oil (0.53 g, 98%); ν_{max} (film)/cm^{-1} 2924 (C–H), 2855 (C–H), 1678 (C=O), 1477 (C=C), 1248 (C–O), 1196 (C–O), 1113 (C–O); **^{1}H NMR** (500 MHz, $CDCl_3$) δ_H: 0.94-1.31 (14H, m, 7 × C*H*$_2$), 1.35-1.48 (3H, m, C*H*$_2$, C*H*^{A}H^B), 1.54-1.63 (1H, m, CH^A*H*^{B}), 1.65-1.77 (1H, m, C*H*^{A}H^B), 1.93-2.06 (1H, m, CH^A*H*^{B}), 3.80 (3H, s, ArC(5)-ArC(2)-OC*H*$_3$), 3.94 (1H, ddd, *J* 15.9, *J* 8.5, *J* 4.3, ArC(1)-OC*H*^{A}H^B), 4.18-4.31 (2H, m, ArC(4)-OC*H*^{A}H^B, ArC(1)-OCH^A*H*^{B}), 4.46 (1H, ddd, *J* 11.9, *J* 5.4, *J* 3.9, ArC(4)-OCH^A*H*^{B}), 6.98-7.07 (3H, m, ArC(6)*H*, ArC(5)-ArC(3,6)*H*), 7.28 (1H, dd, *J* 7.0, *J* 2.3, ArC(5)-ArC(5)*H*), 7.39 (1H, ddd, *J* 8.3, *J* 7.4, *J* 7.8, ArC(5)-ArC(4)*H*), 7.48 (1H, s, ArC(3)*H*), 10.52 (1H, s, C*H*O); **^{13}C{1H} NMR** (126 MHz, $CDCl_3$) δ_C: 23.4 (*C*H$_2$), 24.2 (*C*H$_2$), 26.6 (*C*H$_2$), 26.7 (*C*H$_2$), 27.5 (*C*H$_2$), 27.7 (*C*H$_2$), 27.7 (*C*H$_2$), 27.9 (*C*H$_2$), 28.5

(CH_2), 28.6 (CH_2), 55.6 (OCH_3), 68.4 (ArC(1)-OCH_2), 68.5 (ArC(4)-OCH_2), 111.1 (ArC(5)-Ar*C*(5)H), 113.1 (Ar*C*(3)H), 119.1 (Ar*C*(6)H), 120.2 (ArC(5)-Ar*C*(3)H), 125.1 (Ar*C*(2)), 126.8 (ArC(5)-Ar*C*(1)), 129.3 (ArC(5)-Ar*C*(4)H), 131.1 (ArC(5)-Ar*C*(6)H), 137.3 (Ar*C*(5)), 149.6 (Ar*C*(1)), 154.5 (Ar*C*(4)), 156.7 (ArC(5)-Ar*C*(2)), 189.6 (*C*HO); HRMS (APCI$^+$) $C_{26}H_{34}O_4$ ([M+Na]$^+$) requires 433.23493, found 433.2346 (–0.69 ppm).

Synthesis of 1^5-(naphthalen-1-yl)-2,15-dioxa-1(1,4)-benzenacyclopentadecaphane-1^2-carbaldehyde S26

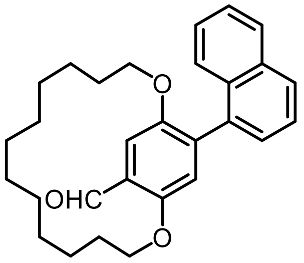

The title compound was prepared according to *General Procedure B* from **S12** (0.5 g, 1.31 mmol), 1-naphthylboronic acid (0.68 g, 3.93 mmol), Pd(PPh_3)$_4$ (75.7 mg, 0.065 mmol) and Cs_2CO_3 (1.28 g, 3.93 mmol) in degassed THF : H_2O (5:1, 12 ml) to afford **S26** as a white solid (0.52 g, 92%); mp 84–88 °C (EtOAc/Hexane); ν_{max} (film)/cm^{-1} 2924 (C–H), 2853 (C–H), 1674 (C=O), 1487 (C=C), 1198 (C–O), 1047 (C–O); **major diastereomer** (NMR time scale 62:38 dr, could not found by HPLC): **^{1}H NMR** (500 MHz, $CDCl_3$) δ_H: 0.94-1.24 (7.2H, m, 6 × C*H*$_2$), 1.26-1.38 (1.8H, m, C*H*$_2$, C*H*^{A}H^B), 1.41-1.54 (1.2H, m, C*H*$_2$), 1.58-1.78 (1.2H, m, C*H*$_2$), 1.90-2.03 (0.6H, m, CH^A*H*^{B}), 3.74-3.91 (0.6H, m, ArC(4)-OC*H*^{A}H^B), 7.18-4.34 (1.2H, m, ArC(4)-OCH^A*H*^{B}, ArC(1)-OC*H*^{A}H^B), 4.37-4.54 (0.6H, m, ArC(1)-OCH^A*H*^{B}), 7.04 (0.6H, s, ArC(3)*H*), 7.38-7.83 (3.6H, s, ArC(6)*H*, 5 × ArC(5)-ArC*H*), 7.89-7.99 (1.2H, 2×ArC(5)-ArC*H*), 10.60 (0.6H, s, C*H*O); **^{13}C{1H} NMR** (126 MHz, $CDCl_3$) δ_C: 23.5 (CH_2), 24.2 (CH_2), 26.5 (CH_2), 26.9 (CH_2), 27.5 (CH_2), 27.7 (CH_2), 27.9 (CH_2), 28.2 (CH_2), 28.7 (CH_2), 28.7 (CH_2), 68.0 (ArC(1)-OCH_2), 68.6 (ArC(4)-OCH_2), 112.6 (Ar*C*(3)H), 119.0 (Ar*C*(6)H), 125.1 (ArC(5)-ArCH), 125.6 (Ar*C*(2)), 125.7 (ArC(5)-ArCH), 126.0 (ArC(5)-ArCH), 126.3 (ArC(5)-ArCH), 128.1 (2 × ArC(5)-ArCH), 128.4 (ArC(5)-ArCH), 131.6 (ArC(5)-Ar*C*), 133.3 (ArC(5)-Ar*C*), 136.6 (ArC(5)-Ar*C*), 139.1 (Ar*C*(5)), 149.8 (Ar*C*(1)), 155.0 (Ar*C*(4)), 189.6 (CHO); HRMS (APCI$^+$) $C_{29}H_{34}O_3$ ([M+Na]$^+$) requires 453.24002, found 453.2400 (+0.01 ppm).

Minor diastereomer (NMR time scale 62:38 dr, could not found by HPLC): **^{1}H NMR** (500 MHz, $CDCl_3$) δ_H: 0.94-1.24 (2.4H, m, 6 × C*H*$_2$), 1.26-1.38 (1.2H, m, C*H*$_2$, C*H*^{A}H^B), 1.41-1.54 (0.8H, m, C*H*$_2$), 1.58-1.78 (0.8H, m, C*H*$_2$), 1.90-2.03 (0.4H, m, CH^A*H*^{B}), 3.74-3.91 (0.4H, m, ArC(4)-OC*H*^{A}H^B), 7.18-4.34 (0.8H, m, ArC(4)-OCH^A*H*^{B}, ArC(1)-OC*H*^{A}H^B), 4.37-4.54 (0.4H, m, ArC(1)-OCH^A*H*^{B}), 7.02 (0.4H, s, ArC(3)*H*), 7.38-7.83 (2H, s, ArC(6)*H*, 5 × ArC(5)-ArC*H*), 7.78-7.84 (0.4H, m, ArC(5)-ArC*H*), 7.89-7.99 (0.8H, 2 × ArC(5)-ArC*H*), 10.60 (0.4H, s, C*H*O); **^{13}C{1H} NMR** (126 MHz, $CDCl_3$) δ_C: **^{13}C{1H} NMR** (126 MHz, $CDCl_3$) δ_C: 23.6 (CH_2), 24.4 (CH_2), 26.9 (CH_2),

27.1 (CH_2), 27.5 (CH_2), 27.9 (CH_2), 27.9 (CH_2), 28.3 (CH_2), 28.7 (CH_2), 28.7 (CH_2), 68.2 (ArC(1)-OCH_2), 68.9 (ArC(4)-OCH_2), 113.7 (Ar*C*(3)H), 120.0 (Ar*C*(6)H), 125.3 (ArC(5)-ArCH), 125.6 (Ar*C*(2)), 125.7 (ArC(5)-ArCH), 125.9 (ArC(5)-ArCH), 126.2 (ArC(5)-ArCH), 126.4 (ArC(5)-ArCH), 127.7 (ArC(5)-ArCH), 128.6 (ArC(5)-ArCH), 131.6 (ArC(5)-Ar*C*), 133.8 (Ar*C*(1)), 133.9 (ArC(5)-Ar*C*), 135.3 (ArC(5)-Ar*C*), 138.6 (Ar*C*(5)), 150.2 (Ar*C*(1)), 154.3 (Ar*C*(4)), 189.5 (*C*HO).

Synthesis of 1⁵-(naphthalen-2-yl)-2,15-dioxa-1(1,4)-benzenacyclopentadecaphane-1²-carbaldehyde S27

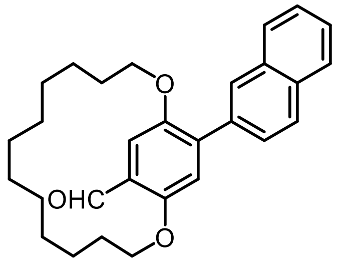

The title compound was prepared according to *General Procedure B* from **S12** (0.5 g, 1.31 mmol), 2-naphthylboronic acid (0.68 g, 3.93 mmol), $Pd(PPh_3)_4$ (75.7 mg, 0.065 mmol) and Cs_2CO_3 (1.28 g, 3.93 mmol) in degassed THF : H_2O (5:1, 12 ml) to afford **S27** as a white oil (0.54 g, 96%); ν_{max} (film)/cm^{-1} 2924 (C–H), 2853 (C–H), 1674 (C=O), 1412 (C=C), 1196 (C–O), 1179 (C–O); **^{1}H NMR** (500 MHz, $CDCl_3$) δ_H: 0.95-1.20 (11H, m, 5 × CH_2, CH^AH^B), 1.26-1.37 (3H, m, CH_2, CH^AH^B), 1.38-1.50 (3H, m, CH_2, CH^AH^B), 1.66-1.80 (2H, m, CH_2), 1.93-2.03 (1H, m, CH^AH^B), 4.04 (1H, ddd, *J* 12.2, *J* 8.5, *J* 4.1, ArC(4)-OCH^AH^B), 4.26-4.40 (2H, m, ArC(4)-OCH^AH^B, ArC(1)-OCH^AH^B), 4.54 (1H, ddd, *J* 11.8, *J* 6.1, *J* 3.9, ArC(1)-OCH^AH^B), 7.15 (1H, s, ArC(6)*H*), 7.52-7.58 (3H, m, ArC(3)*H*, 2 × ArC(5)-ArC*H*), 7.72 (1H, dd, *J* 8.5, *J* 1.8, ArC(5)-ArC*H*), 7.88-7.96 (3H, m, 3 × ArC(5)-ArC*H*), 8.01 (1H, d, *J* 1.7, ArC(5)-ArC*H*), 10.55 (1H, s, C*H*O); **^{13}C{1H} NMR** (126 MHz, $CDCl_3$) δ_C: 23.8 (CH_2), 24.2 (CH_2), 26.7 (CH_2), 27.2 (CH_2), 27.4 (CH_2), 27.6 (CH_2), 27.9 (CH_2), 28.1 (CH_2), 28.6 (CH_2), 28.6 (CH_2), 68.7 (ArC(4)-OCH_2), 68.8 (ArC(1)-OCH_2), 113.7 (Ar*C*(6)H), 118.2 (Ar*C*(3)H), 125.2 (Ar*C*(2)), 126.2 (ArC(5)-ArCH), 126.4 (ArC(5)-ArCH), 127.4 (ArC(5)-ArCH), 127.5 (ArC(5)-ArCH), 127.7 (ArC(5)-ArCH), 128.3 (ArC(5)-ArCH), 128.4 (ArC(5)-ArCH), 132.8 (ArC(5)-Ar*C*), 133.2 (ArC(5)-Ar*C*), 135.4 (ArC(5)-Ar*C*), 139.8 (Ar*C*(5)), 149.4 (Ar*C*(1)), 155.4 (Ar*C*(4)), 189.5 (*C*HO); HRMS (APCI$^+$) $C_{28}H_{34}O_3$ ([M+Na]$^+$) requires 453.24002, found 453.2398 (–0.37 ppm).

Synthesis of 1⁵-(pyridin-3-yl)-2,15-dioxa-1(1,4)-benzenacyclopentadecaphane-1²-carbaldehyde S28

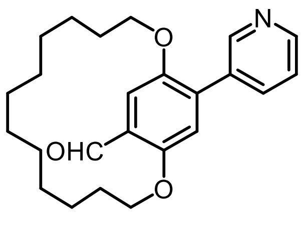

The title compound was prepared according to *General Procedure B* from **S12** (0.5 g, 1.31 mmol), 3-pyridine boronic acid (0.48 g, 3.93 mmol), $Pd(PPh_3)_4$ (75.7 mg, 0.065 mmol) and Cs_2CO_3 (1.28 g, 3.93 mmol) in degassed THF : H_2O (5:1, 12 ml) was stirred at 50 ℃ for 24 h. The mixture was purified by column chromatography (30%-50% EtOAc/Petrol ether) to afford **S28** as a yellow solid (0.51 g, 78%); mp 32–34 °C (EtOAc/Hexane); ν_{max} (film)/cm^{-1} 2924 (C–H), 2853 (C–H), 1678 (C=O), 1404 (C=C), 1182 (C–O); **^{1}H NMR** (500 MHz, $CDCl_3$) δ_H: 0.84-1.31 (14H, m, 7 × C*H*$_2$), 1.35-1.48 (3H, m, C*H*$_2$, C*H*^{A}H^B), 1.63-1.80 (2H, m, C*H*$_2$), 1.90-2.0 (1H, m, CH^A*H*^{B}), 4.08 (1H, ddd, *J* 12.2, *J* 8.7, *J* 3.9, ArC(4)-OC*H*^{A}H^B), 4.30 (1H, ddd, *J* 12.2, *J* 8.7, *J* 3.8, ArC(4)-OCH^A*H*^{B}), 4.38 (1H, ddd, *J* 11.8, *J* 5.9, *J* 4.0, ArC(1)-OC*H*^{A}H^B), 4.51 (1H, ddd, *J* 11.8, *J* 5.9, *J* 4.0, ArC(1)-OCH^A*H*^{B}), 7.05 (1H, s, ArC(6)*H*), 7.40 (1H, ddd, *J* 7.9, *J* 4.8, ArC(5)-ArC(4)*H*), 7.51 (1H, s, ArC(3)*H*), 7.94 (1H, dt, *J* 8.0, *J* 1.9, ArC(5)-ArC(5)*H*), 8.64 (1H, d, *J* 4.1, ArC(5)-ArC(6)*H*), 8.80 (1H, s, ArC(5)-ArC(2)*H*) , 10.53 (1H, s, C*H*O); **^{13}C{1H} NMR** (126 MHz, $CDCl_3$) δ_C: 23.7 (*C*H$_2$), 24.1 (*C*H$_2$), 26.5 (*C*H$_2$), 27.1 (*C*H$_2$), 27.4 (*C*H$_2$), 27.6 (*C*H$_2$), 27.9 (*C*H$_2$), 28.1 (*C*H$_2$), 28.5 (*C*H$_2$), 28.6 (*C*H$_2$), 68.5 (ArC(1)-OCH$_2$), 68.9 (ArC(4)-OCH$_2$), 113.1 (Ar*C*(6)H), 117.7 (Ar*C*(3)H), 123.0 (ArC(5)-Ar*C*), 125.8 (Ar*C*(2)), 133.5 (ArC(5)-Ar*C*), 135.7 (ArC(5)-Ar*C*), 137.0 (Ar*C*(5)H), 148.8 (ArC(5)-Ar*C*H), 149.2 (Ar*C*(1)), 149.7 (ArC(5)-Ar*C*H), 155.1 (Ar*C*(4)), 189.3 (*C*HO); HRMS (APCI$^+$) $C_{24}H_{31}O_3N$ ([M+Na]$^+$) requires 404.21962, found 404.2193 (–0.67 ppm).

Synthesis of 1^5-(furan-2-yl)-2,15-dioxa-1(1,4)-benzenacyclopentadecaphane-1^2-carbaldehyde S29

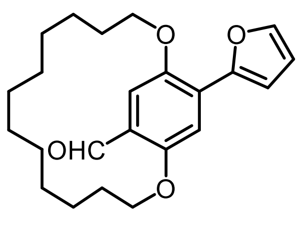

The title compound was prepared according to *General Procedure B* from **S12** (0.5 g, 1.31 mmol), 2-furanylboronic acid (0.44 g, 3.93 mmol), $Pd(PPh_3)_4$ (75.7 mg, 0.065 mmol) and Cs_2CO_3 (1.28 g, 3.93 mmol) in degassed THF : H_2O (5:1, 12 ml) to afford **S29** as a red solid (0.48 g, 99%); mp 44–46 °C (EtOAc/Hexane); ν_{max} (film)/cm^{-1} 2924 (C–H), 2851 (C–H), 1670 (C=O), 1602 (C=O), 1481 (C=C), 1188 (C–O); **^{1}H NMR** (500 MHz, $CDCl_3$) δ_H: 0.82-0.88 (4H, m, 2 × C*H*$_2$), 0.89-0.93 (2H, m, C*H*$_2$), 1.04-1.17 (3H, m, C*H*$_2$, C*H*^{A}H^B), 1.20-1.32 (5H,

m, 2 × C*H*$_2$, CH^A*H*^{B}), 1.35-1.42 (2H, m, C*H*$_2$), 1.57-1.69 (2H, m, C*H*$_2$), 1.84-1.98 (2H, m, C*H*$_2$), 4.22-4.36 (2H, m, ArC(4)-OC*H*$_2$), 4.57 (2H, m, ArC(1)-OCH$_2$), 6.57 (1H, dd, *J* 3.4, *J* 1.8, ArC(5)-ArC(4)*H*), 7.23 (1H, dd, *J* 3.4, *J* 0.7, ArC(5)-ArC(3)*H*), 7.47 (1H, s, ArC(6)*H*), 7.56 (1H, dd, *J* 1.7, *J* 0.5, ArC(5)-ArC(5)*H*), 7.57 (1H, s, ArC(3)*H*), 10.48 (1H, s, C*H*O); **^{13}C{1H} NMR** (126 MHz, CDCl$_3$) δ_C: 23.8 (CH$_2$), 24.2 (CH$_2$), 26.7 (CH$_2$), 27.0 (CH$_2$), 27.4 (CH$_2$), 27.6 (CH$_2$), 27.9 (CH$_2$), 28.0 (CH$_2$), 28.4 (CH$_2$), 28.5 (CH$_2$), 68.1 (ArC(4)-OCH$_2$), 68.8 (ArC(1)-OCH$_2$), 112.2 (Ar*C*(6)H), 112.3 ((ArC(5)-Ar*C*(3)H), 112.4 ((ArC(5)-Ar*C*(4)H), 113.4 (Ar*C*(3)H), 124.2 (Ar*C*(2)), 127.6 (Ar*C*(5)), 142.5 ((ArC(5)-Ar*C*(5)H), 147.7 (Ar*C*(1)), 149.4 (Ar*C*(4)), 155.2 (ArC(5)-ArC(2)), 189.2 (CHO); HRMS (APCI$^+$) $C_{23}H_{30}O_4$ ([M+Na]$^+$) requires 393.20363, found 393.2043 (+1.72 ppm).

Synthesis of 1^5-(thiophen-3-yl)-2,15-dioxa-1(1,4)-benzenacyclopentadecaphane-1^2-carbaldehyde S30

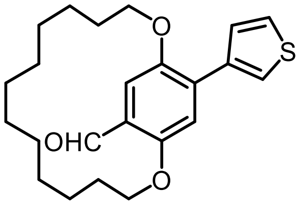

The title compound was prepared according to *General Procedure B* from **S12** (0.5 g, 1.31 mmol), 3-thienylboronic acid (0.50 g, 3.93 mmol), Pd(PPh$_3$)$_4$ (75.7 mg, 0.065 mmol) and Cs_2CO_3 (1.28 g, 3.93 mmol) in degassed THF : H_2O (5:1, 12 ml) to afford **S30** as a yellow oil (0.46 g, 91%); ν_{max} (film)/cm^{-1} 2924 (C–H), 2851 (C–H), 1670 (C=O), 1602 (C=O), 1481 (C=C), 1188 (C–O); **^{1}H NMR** (500 MHz, CDCl$_3$) δ_H: 0.84-1.32 (14H, m, 7 × C*H*$_2$), 1.33-1.44 (2H, m, C*H*$_2$), 1.47-1.58 (1H, m, C*H*^{A}H^B), 1.63-1.74 (1H, m, CH^A*H*^{B}, 1.76-1.96 (2H, m, C*H*$_2$), 4.18 (1H, ddd, *J* 12.1, *J* 8.7, *J* 4.0, ArC(4)-OC*H*^{A}H^B), 4.29 (1H, ddd, *J* 12.1, *J* 8.5, *J* 3.9, ArC(4)-OCH^A*H*^{B}), 4.44 (1H, ddd, *J* 11.9, *J* 6.0, *J* 4.1, ArC(1)-OC*H*^{A}H^B), 4.51 (1H, ddd, *J* 11.8, *J* 6.1, *J* 4.0, ArC(1)-OCH^A*H*^{B}), 7.21 (1H, s, ArC(6)*H*), 7.40 (1H, dd, *J* 5.1, *J* 3.0, ArC(5)-ArC(4)*H*), 7.45-7.52 (2H, m, ArC(5)-ArC(2)*H*, ArC(3)*H*), 7.79 (1H, dd, *J* 3.0, *J* 1.3, ArC(5)-ArC(5)*H*), 10.50 (1H, s, C*H*O). Data in agreement with the literature.[26i]

Synthesis of *tert*-butyl 3-(1^5-formyl-2,15-dioxa-1(1,4)-benzenacyclopentadecaphane-1^2-yl)-5-methoxy-1H-indole-1-carboxylate S31

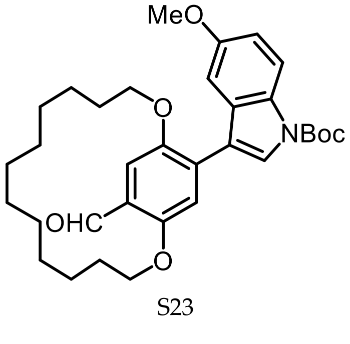

S23

The title compound was prepared according to *General Procedure B* from **S12** (0.5 g, 1.31 mmol), 5-methoxy-1H-indole-2-boronic acid (1.14 g, 3.93 mmol), $Pd(PPh_3)_4$ (75.7 mg, 0.065 mmol) and Cs_2CO_3 (1.28 g, 3.93 mmol) in degassed THF : H_2O (5:1, 12 ml) was stirred at 50 °C for 24 h. The mixture was purified by column chromatography (20%-30% EtOAc/Petrol ether) to afford **31** as a yellow oil (0.70 g, 97%); ν_{max} (film)/cm^{-1} 2924 (C–H), 2853 (C–H), 1732 (C=O), 1678 (C=O), 1123 (C–O); **^{1}H NMR** (500 MHz, $CDCl_3$) δ_H: 0.88-0.98 (8H, m, 4 × C*H*$_2$), 1.03-1.09 (3H, m, C*H*$_2$, C*H*^{A}H^B), 1.22-1.32 (4H, m, 2 × C*H*$_2$), 1.36 (9H, s, C(C*H*$_3$)), 1.40-1.46 (2H, m, C*H*$_2$), 1.64-1.74 (2H, m, C*H*$_2$), 1.94-2.06 (1H, m, CH^A*H*^{B}), 3.90 (3H, s, OC*H*$_3$), 3.99 (1H, ddd, *J* 12.1, *J* 8.5, *J* 3.9, ArC(4)-OC*H*^{A}H^B), 4.24-4.36 (2H, m, ArC(4)-OCH^A*H*^{B}, ArC(1)-OC*H*^{A}H^B), 4.53 (1H, ddd, *J* 11.9, *J* 5.9, *J* 3.9, ArC(1)-OCH^A*H*^{B}), 6.49 (1H, s, indoleC(2)*H*), 6.99 (1H, dd, *J* 9.0, *J* 2.6, indoleC(6)*H*), 7.08 (1H, d, *J* 2.5, indoleC(4)*H*), 7.14 (1H, s, ArC(6)*H*), 7.41 (1H, s, ArC(6)*H*), 8.12 (1H, d, *J* 9.0, indoleC(7)*H*), 10.53 (1H, s, C*H*O); **^{13}C{1H} NMR** (126 MHz, $CDCl_3$) δ_C: 23.5 (*C*H$_2$), 24.1 (*C*H$_2$), 26.6 (*C*H$_2$), 27.1 (*C*H$_2$), 27.3 (*C*H$_2$), 27.5 (C(*C*H$_3$)$_3$), 27.7 (*C*H$_2$), 28.0 (*C*H$_2$), 28.1 (*C*H$_2$), 28.6 (*C*H$_2$), 28.6 (*C*H$_2$), 55.7 (*C*(CH$_3$)), 68.1 (ArC(4)-O*C*H$_2$), 68.6 (ArC(1)-O*C*H$_2$), 83.4 (O*C*H$_3$), 103.0 (indole*C*(2)H), 109.7 (indole*C*(6)H), 111.8 (Ar*C*(6)H), 113.5 (indole*C*(4)H), 115.8 (indole*C*(7)H), 116.8 (Ar*C*(3)H), 125.3 (Ar*C*(2)), 129.4 (indole*C*(3)), 132.1 (indole*C*(3a)), 133.7 (Ar*C*(5)), 136.9 (indole*C*(7a)), 149.7 (Ar*C*(1)), 149.9 (N*C*=O), 155.1 (Ar*C*(4)), 155.8 (indole*C*(5)), 189.4 (*C*HO); HRMS (APCI$^+$) $C_{33}H_{43}O_6N$ ([M+Na]$^+$) requires 572.29826, found 572.2980 (–0.41 ppm).

Synthesis of 1^5-(pyren-1-yl)-2,15-dioxa-1(1,4)-benzenacyclopentadecaphane-1^2-carbaldehyde S32

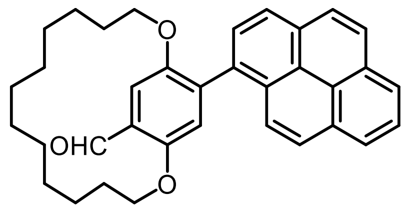

The title compound was prepared according to *General Procedure B* from **S12** (0.5 g, 1.31 mmol), 1-pyrene boronic acid (0.97 g, 3.93 mmol), $Pd(PPh_3)_4$ (75.7 mg, 0.065 mmol) and Cs_2CO_3 (1.28 g, 3.93 mmol) in degassed THF : H_2O (5:1, 12 ml) to afford **S32** as a yellow solid (0.57 g, 87%); mp 58–62 °C (EtOAc/Hexane); ν_{max} (film)/cm^{-1} 2920 (C–H), 2851 (C–H), 1678 (C=O), 1412 (C=C), 1194 (C–O), 1182 (C–O); **major diastereomer** (NMR time scale 59:41 dr, could not found by HPLC): **^{1}H NMR** (500 MHz, $CDCl_3$) δ_H: 1.01-1.56 (10.2H, m, 8 × C*H*$_2$, C*H*^{A}H^B), 1.59-1.77 (1.2H, m, C*H*$_2$), 1.96-2.11 (0.6H, m, CH^A*H*^{B}), 3.74-3.87 (0.6H, m, ArC(4)-OC*H*^{A}H^B), 4.20-4.33 (1.2H, m, ArC(4)-OCH^A*H*^{B}, ArC(1)-OC*H*^{A}H^B), 4.40-4.53 (0.6H, m, ArC(1)-OCH^A*H*^{B}), 7.16 (0.6H, s, ArC(6)*H*), 7.65 (0.6H, s, ArC(3)*H*), 7.85-8.33 (5.4H, m, 9 × ArC(5)-ArC*H*), 10.66 (0.6H, s, C*H*O). Data in agreement with the literature.[26f]

Minor diastereomer (NMR time scale 59:41 dr, could not found by HPLC): **^{1}H NMR** (500 MHz, $CDCl_3$) **^{1}H NMR** (500 MHz, $CDCl_3$) δ_H: 1.01-1.56 (6.8H, m, 8.5×C*H*$_2$, C*H*^{A}H^B), 1.59-1.77 (0.8H, m, C*H*$_2$), 1.96-2.11 (0.4H, m, CH^A*H*^{B}), 3.74-3.87 (0.4H, m, ArC(4)-OC*H*^{A}H^B), 4.20-4.33 (0.8H, m, ArC(4)-OCH^A*H*^{B}, ArC(1)-OC*H*^{A}H^B), 4.40-4.53 (0.4H, m, ArC(1)-OCH^A*H*^{B}), 7.13 (0.4H, s, ArC(6)*H*), 7.71 (0.4H, s, ArC(3)*H*), 7.85-8.33 (3.6H, m, 9 × ArC(5)-ArC*H*), 10.65 (0.4H, s, C*H*O).

Synthesis of 1^5-phenyl-2,16-dioxa-1(1,4)-benzenacyclotridecaphane-1^2-carbaldehyde S33

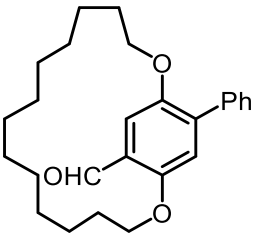

The title compound was prepared according to *General Procedure B* from **S14** (0.42 g, 1.05 mmol), phenyl boronic acid (0.38 g, 3.15 mmol), $Pd(PPh_3)_4$ (60.7 mg, 0.053 mmol) and Cs_2CO_3 (1.03 g, 3.15 mmol) in degassed THF : H_2O (5:1, 12 ml) to afford **S33** as a colourless oil (0.41 g, 99%); ν_{max} (film)/cm^{-1} 2922 (C–H), 2853 (C–H), 1680 (C=O), 1410 (C=C), 1188 (C–O); **^{1}H NMR** (500 MHz, $CDCl_3$) δ_H: 0.94-1.14 (11H, m, 5 × C*H*$_2$, C*H*^{A}H^B), 1.17-1.23 (2H, m, C*H*$_2$), 1.25-1.37 (3H, m, C*H*$_2$, CH^A*H*^{B}), 1.38-1.56 (3H, m, C*H*$_2$, C*H*^{A}H^B), 1.62-1.90 (3H, m, C*H*$_2$, CH^A*H*^{B}), 4.06 (1H, ddd, *J* 11.7, *J* 7.5, *J* 4.3, ArC(4)-OC*H*^{A}H^B), 4.20-4.33 (2H, m, ArC(1)-OC*H*^{A}H^B, ArC(4)-OCH^A*H*^{B}), 4.44 (1H, ddd, *J* 11.1, *J* 6.8, *J* 4.0, ArC(1)-OCH^A*H*^{B}), 7.05 (1H, s, ArC(6)*H*), 7.37-7.48 (3H, m, 3 × ArC(5)-PhC*H*), 7.49 (1H, s, ArC(3)*H*), 7.52-7.60 (2H, m, 2 × ArC(5)-PhC*H*), 10.53 (1H, s, C*H*O). Data in agreement with the literature.[26i]

Synthesis of 1^5-phenyl-2,17-dioxa-1(1,4)-benzenacyclotridecaphane-1^2-carbaldehyde S34

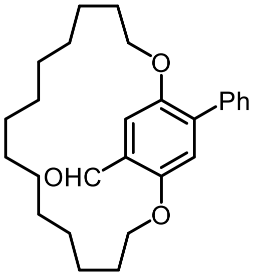

The title compound was prepared according to *General Procedure B* from **S15** (0.43 g, 1.05 mmol), phenyl boronic acid (0.38 g, 3.15 mmol), $Pd(PPh_3)_4$ (60.7 mg, 0.053 mmol) and Cs_2CO_3 (1.03 g, 3.15 mmol) in degassed THF : H_2O (5:1, 12 ml) to afford **S34** as a colourless oil (0.42 g, 98%); ν_{max} (film)/cm^{-1} 2922 (C–H), 2853 (C–H), 1680 (C=O), 1412 (C=C), 1194 (C–O); **^{1}H NMR** (500 MHz, $CDCl_3$) δ_H: 0.95-1.08 (8H, m, 4 × C*H*$_2$), 1.10-1.20 (5H,

m, 2 × CH_2, CH^AH^B), 1.22-1.33 (5H, m, 2 × CH_2, CH^AH^B), 1.36-1.55 (2H, m, CH_2), 1.61-1.70 (2H, m, *J* 6.9, CH_2), 1.72-1.91 (2H, m, *J* 6.9, CH_2), 4.06 (1H, dd, *J* 11.0, *J* 5.4, ArC(4)-OCH^AH^B), 4.14-4.30 (2H, m, ArC(4)-OCH^AH^B, ArC(1)-OCH^AH^B), 4.30-4.49 (1H, m, ArC(1)-OCH^AH^B), 7.02 (1H, s, ArC(6)*H*), 7.31-7.49 (4H, m, 3 × ArC(5)-PhC*H*, ArC(3)*H*), 7.50-7.63 (2H, m, 2 × ArC(5)-PhC*H*), 10.53 (1H, s, C*H*O). Data in agreement with the literature.[26f]

Synthesis of 4,1^5-dioxo-15-phenyl-2,17-dioxa-5,14-diaza-1(1,4)-benzenacycloheptadecaphane-1^2-carbaldehyde S35

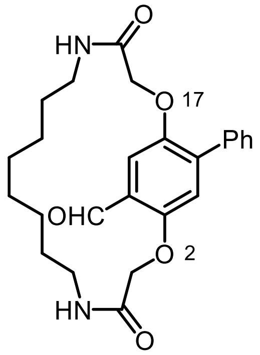

The title compound was prepared according to *General Procedure B* from **S18** (0.46 g, 1.05 mmol), phenyl boronic acid (0.38 g, 3.15 mmol), Pd(PPh_3)$_4$ (60.7 mg, 0.053 mmol) and Cs_2CO_3 (1.03 g, 3.15 mmol) in degassed THF : H_2O (5:1, 12 ml) was stirred at 50 °C for 24 h. The miture was purified by column chromatography (20%-35% Aceton/CH_2Cl_2) to afford **S35** as a colourless oil (0.40 g, 87%); ν_{max} (film)/cm^{-1} 2922 (C–H), 2851 (C–H), 1678 (C=O), 1410 (C=C), 1200 (C–O); **^{1}H NMR** (500 MHz, $CDCl_3$) δ_H: 0.76-0.88 (2H, m, CH_2), 0.94-1.04 (2H, m, CH_2), 1.04-1.20 (5H, m, 2 × CH_2, CH^AH^B), 1.38-1.51 (2H, m, CH_2), 2.66 (1H, m, CH^AH^B), 2.99 (1H, ddt, *J* 13.9, *J* 7.2, *J* 3.4, CH^AH^B), 3.58 (1H, m, CH^AH^B), 4.65-4.84 (4H, m, ArC(4)-OCH_2, ArC(1)-OCH_2), 5.88 (1H, dd, *J* 8.4, *J* 3.6, C(4)ON*H*), 6.72 (1H, dd, *J* 8.4, *J* 3.4, C(15)ON*H*), 7.02 (1H, s, ArC(6)*H*), 7.43-7.60 (6H, m, 5 × ArC(5)-PhC*H*, ArC(3)*H*), 10.39 (1H, s, C*H*O); **^{13}C{1H} NMR** (126 MHz, $CDCl_3$) δ_C: 26.5 (CH_2), 26.6 (CH_2), 28.4 (CH_2), 28.8 (CH_2), 29.6 (CH_2), 29.7 (CH_2), 39.0 (CH_2), 39.3 (CH_2), 68.5 (ArC(4)-OCH_2), 69.0 (ArC(1)-OCH_2), 114.3 (Ar*C*(6)H), 115.7 (Ar*C*(3)H), 124.4 (Ar*C*(2)), 128.7 (ArC(5)-Ph*C*(3,5)H), 129.0 (ArC(5)-Ph*C*(4)H), 129.5 (ArC(5)-Ph*C*(2,6)H), 135.8 (ArC(5)-PhC(1)), 138.9 (Ar*C*(5)), 149.5 (Ar*C*(1)), 154.0 (Ar*C*(4)), 168.0 (2 × CONH), 188.4 (*C*HO); HRMS (APCI$^+$) $C_{25}H_{30}O_5N_2$ ([M+Na]$^+$) requires 461.20469, found 461.2046 (–0.23 ppm).

Synthesis of 1^5-phenyl-2,18-dioxa-1(1,4)-benzenacyclotridecaphane-1^2-carbaldehyde S36

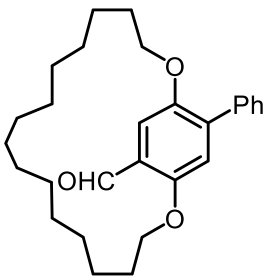

The title compound was prepared according to *General Procedure B* from **S16** (0.47 g, 1.10 mmol), phenyl boronic acid (0.40 g, 3.30 mmol), $Pd(PPh_3)_4$ (63.6 mg, 0.056 mmol) and Cs_2CO_3 (1.08 g, 3.30 mmol) in degassed THF : H_2O (5:1, 12 ml) to afford **S36** as a colourless oil (0.44 g, 94%); ν_{max} (film)/cm^{-1} 2922 (C–H), 2851 (C–H), 1678 (C=O), 1410 (C=C), 1196 (C–O); **^{1}H NMR** (500 MHz, $CDCl_3$) δ_H: 1.01-1.54 (22H, m, 11 × C*H*$_2$), 1.58-1.71 (2H, m, C*H*$_2$), 1.72-1.87 (2H, m, C*H*$_2$), 3.98-4.19 (2H, m, ArC(4)-OC*H*$_2$), 4.19-4.40 (2H, m, ArC(1)-OC*H*$_2$), 7.03 (1H, s, ArC(6)*H*), 7.36-7.49 (4H, m, 3 × ArC(5)-PhC*H*, ArC(3)*H*), 7.52-7.59 (2H, m, 2 × ArC(5)-PhC*H*), 10.53 (1H, s, C*H*O). Data in agreement with the literature.[26f]

Synthesis of 1^5-phenyl-2,19-dioxa-1(1,4)-benzenacyclotridecaphane-1^2-carbaldehyde S37

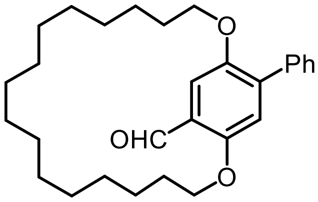

The title compound was prepared according to *General Procedure B* from **S17** (0.46 g, 1.05 mmol), phenyl boronic acid (0.38 g, 3.15 mmol), $Pd(PPh_3)_4$ (60.7 mg, 0.053 mmol) and Cs_2CO_3 (1.03 g, 3.15 mmol) in degassed THF : H_2O (5:1, 12 ml) to afford **S37** as a colourless oil (0.44 g, 97%); ν_{max} (film)/cm^{-1} 2922 (C–H), 2851 (C–H), 1678 (C=O), 1410 (C=C), 1200 (C–O); **^{1}H NMR** (500 MHz, $CDCl_3$) δ_H: 1.08-1.24 (16H, m, 8 × C*H*$_2$), 1.27-1.36 (6H, m, 3 × C*H*$_2$), 1.51 (2H, p, *J* 7.0, C*H*$_2$), 1.68 (2H, p, *J* 6.4, *J* 6.3, C*H*$_2$), 1.83 (2H, p, *J* 6.4, C*H*$_2$), 4.09 (2H, t, *J* 5.9, ArC(4)-OC*H*$_2$), 4.25 (2H, t, *J* 5.9, ArC(1)-OC*H*$_2$), 7.01 (1H, s, ArC(6)*H*), 7.36-7.48 (4H, m, ArC(5)-PhC(2,4,6)*H*, ArC(3)*H*), 7.52-7.59 (2H, m, ArC(5)-PhC(3,5)*H*), 10.54 (1H, s, C*H*O); **^{13}C{1H} NMR** (126 MHz, $CDCl_3$) δ_C: 23.9 (CH_2), 24.2 (CH_2), 26.6 (CH_2), 27.0 (CH_2), 27.5 (CH_2), 27.7 (CH_2), 27.9 (CH_2), 28.1 (CH_2), 28.1 (CH_2), 28.3 (CH_2), 28.6 (CH_2), 28.8 (CH_2), 29.3 (CH_2), 29.4 (CH_2), 67.8 (ArC(4)-OCH_2), 68.0 (ArC(1)-OCH_2), 111.1 (Ar*C*(6)H), 116.2 (Ar*C*(3)H), 124.5 (Ar*C*(2)), 127.8 (ArC(5)-Ph*C*(4)H), 128.0 (ArC(5)-Ph*C*(3,5)H), 129.5 (ArC(5)-Ph*C*(2,6)H), 137.7 (ArC(5)-Ph*C*(1)), 139.2 (Ar*C*(5)), 149.7 (Ar*C*(1)), 155.6 (Ar*C*(4)), 189.3 (*C*HO); HRMS (APCI$^+$) $C_{29}H_{41}O_3$ ($[M+Na]^+$) requires 437.30502, found 437.3046 (–0.97 ppm).

Synthesis of 1^5-(pyridin-3-yl)-2,13-dioxa-1(1,4)-benzenacyclotridecaphan-1^2-ol S38

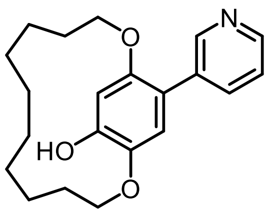

The title compound was prepared according to *General Procedure B* from **1** (0.40 g, 1.16 mmol), boronic acid (0.43 g, 3.48 mmol), $Pd(PPh_3)_4$ (0.06 g, 0.06 mmol) and Cs_2CO_3 (1.13 g, 3.48 mmol) in degassed THF : H_2O (5:1, 12 ml) was stirred at 50 °C for 24 h. The mixture was purified by column chromatography (25%-35% EtOAc/Petrol ether) to afford **S38** as a white solid (0.27 g, 69%); mp 87–90 °C (EtOAc/Hexane); ν_{max} (film)/cm^{-1} 3038 (O–H), 2924 (C–H), 2853 (C–H), 1508 (C=C), 1288 (C–O), 1169 (C–O); **^{1}H NMR** (500 MHz, $CDCl_3$) δ_H: 0.77-1.02 (7H, m, 3 × C*H*$_2$, C*H*^{A}H^B), 1.03-1.25 (4H, m, 2 × C*H*$_2$), 1.27-1.38 (2H, m, C*H*$_2$), 1.55-1.74 (2H, m, C*H*$_2$), 1.80-1.88 (1H, m, CH^A*H*^{B}), 3.96 (1H, ddd, *J* 12.4, *J* 8.7, *J* 3.8, ArC(4)-OC*H*^{A}H^B), 4.16-4.21 (1H, m, ArC(4)-OCH^A*H*^{B}), 4.21-4.24 (1H, m, ArC(1)-OC*H*^{A}H^B), 4.35 (1H, ddd, *J* 12.1, *J* 6.5, *J* 3.9, ArC(1)-OCH^A*H*^{B}), 6.44 (1H, s, ArC(2)-O*H*), 6.88 (1H, s, ArC(3)*H*), 7.10 (1H, s, ArC(6)*H*), 7.36 (1H, ddd, *J* 7.9, *J* 4.8, *J* 0.8, ArC(5)-ArC(4)*H*), 7.91 (1H, ddd, *J* 7.9, *J* 2.3, *J* 1.7, ArC(5)-ArC(5)*H*), 8.59 (1H, dd, *J* 4.9, *J* 1.7, ArC(5)-ArC(6)*H*), 8.78 (1H, d, *J* 1.8, ArC(5)-ArC(2)*H*); **^{13}C{1H} NMR** (126 MHz, $CDCl_3$) δ_C: 23.8 (*C*H$_2$), 23.9 (*C*H$_2$), 27.2 (*C*H$_2$), 27.3 (*C*H$_2$), 27.4 (*C*H$_2$), 27.5 (*C*H$_2$), 27.5 (*C*H$_2$), 27.8 (*C*H$_2$), 70.0 (ArC(1)-O*C*H$_2$), 70.4 (ArC(4)-O*C*H$_2$), 106.2 (Ar*C*(3)H), 118.6 (Ar*C*(6)H), 121.1 (Ar*C*(5)), 123.0 (ArC(5)-Ar*C*(5)H), 134.4 (ArC(5)-Ar*C*(3)), 137.0 (ArC(5)-Ar*C*(4)H), 139.9 (Ar*C*(1)), 147.4 (ArC(5)-Ar*C*(6)H), 148.4 (Ar*C*(2)), 149.8 (ArC(5)-Ar*C*(2)H), 151.1 (Ar*C*(4)); HRMS (APCI$^+$) $C_{21}H_{27}O_3N$ ([M+Na]$^+$) requires 364.18831, found 364.1881 (–0.56 ppm).

Synthesis of 1^5-vinyl-2,15-dioxa-1(1,4)-benzenacyclopentadecaphan-1^2-ol S39

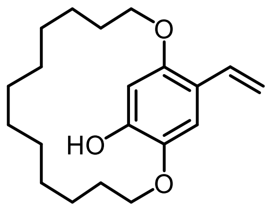

S47 (0.36 g, 0.98 mmol), potassium vinyltrifluoroborate (0.39 g, 2.94 mmol), $Pd(OAc)_2$ (6.6 mg, 0.04 mmol), xPhos (28.0 mg, 0.08 mmol), and Cs_2CO_3 (0.96 g, 2.94 mmol) were added to a dried flask. The flask was evacuated and backfilled with N_2 three times. Then the degassed THF : H_2O (5:1, 30 ml) was added to the reaction. The reaction was stirred at 50 °C for 24 h. After cooling to room temperature, diluted with water (20 ml) and extracted with EtOAc (3 × 40 mL). The combined organic layers were washed with water and brine

and dried over Na_2SO_4, filtered, and concentrated in *vacuo*. The residue was purified by column chromatography (2%-5% EtOAc/Petrol ether) to afford **S39** as a yellow solid (0.12 g, 40%); mp 70–75 °C (EtOAc/Hexane); ν_{max} (film)/cm^{-1} 3537 (O–H), 2922 (C–H), 2853 (C–H), 1503 (C=C), 1211 (C=C), 1161 (C–O); **^{1}H NMR** (500 MHz, $CDCl_3$) δ_H: 0.94-1.01 (7H, m, 3 × CH_2, CH^AH^B), 1.10-1.24 (4H, m, 2 × CH_2), 1.26-1.43 (5H, m, 2 × CH_2, CH^AH^B), 1.52-1.60 (2H, m, CH_2), 1.74-1.86 (2H, m, CH_2), 4.14 (1H, ddd, *J* 12.1, *J* 8.3, *J* 4.4, ArC(4)-OCH^AH^B), 4.22 (1H, ddd, *J* 12.1, *J* 8.2, *J* 4.6, ArC(4)-OCH^AH^B), 4.30 (1H, ddd, *J* 11.7, *J* 6.0, *J* 4.4, ArC(1)-OCH^AH^B), 4.37 (1H, ddd, *J* 11.1, *J* 6.0, *J* 4.7, ArC(1)-OCH^AH^B), 5.14 (1H, dd, *J* 11.1, *J* 1.5, ArC(5)-CH=CH^AH^B), 5.55 (1H, dd, *J* 17.7, *J* 1.5, ArC(5)-CH=CH^AH^B), 5.79 (1H, s, ArC(2)-O*H*), 6.60 (1H, s, ArC(3)*H*), 7.03 (1H, dd, *J* 17.7, *J* 11.1, ArC(5)-C*H*=CH_2), 7.07 (1H, s, ArC(6)*H*); **^{13}C{1H} NMR** (126 MHz, $CDCl_3$) δ_C: 23.9 (CH_2), 24.0 (CH_2), 26.9 (CH_2), 27.1 (CH_2), 27.4 (CH_2), 27.5 (CH_2), 27.8 (CH_2), 27.8 (CH_2), 28.4 (CH_2), 28.4 (CH_2), 68.6 (ArC(1)-OCH_2), 69.5 (ArC(4)-OCH_2), 102.3 (Ar*C*(3)H), 111.5 (Ar*C*(5)-CH=CH_2), 112.3 (Ar*C*(5)-CH=CH_2), 119.9 (ArC(5)), 131.6 (Ar*C*(6)H), 138.7 (Ar*C*(1)), 147.4 (Ar*C*(2)), 150.5 (Ar*C*(4)); HRMS (APCI$^+$) $C_{20}H_{29}O_3$ ([M+Na]$^+$) requires 317.21222, found 317.2121 (–0.24 ppm).

Synthesis of methyl 1,4-dihydroxy-2-naphthoate S40

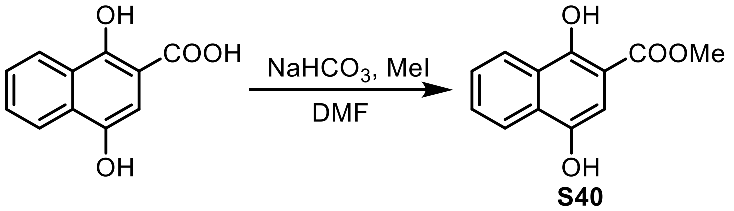

Following the method described by Armylisas *et al.*,[59] 1,4-Dihydroxy-2-naphthoic acid (3.68 g, 18.00 mmol) and $NaHCO_3$ (2.00 g, 22.00 mmol) were suspend in dry DMF (0.3 M, 60 ml) and stirred for 30 minutes with N_2 atmosphere. MeI (4.00 g, 28.00 mmol) was slowly added to the mixture and stirred for 22 h. The solution was quenched by saturated brine (200 ml) and followed by HCl (1 M, 120 mL), then extracted with Et_2O (3 × 80 mL). The combined organic layers were washed with water and brine and dried over Na_2SO_4, filtered, and concentrated in *vacuo*. The crude product **S40** was used for next step without any purification.

Synthesis of methyl 2,15-dioxa-1(1,4)-naphthalenacyclopentadecaphane-1^2-carboxylate S41

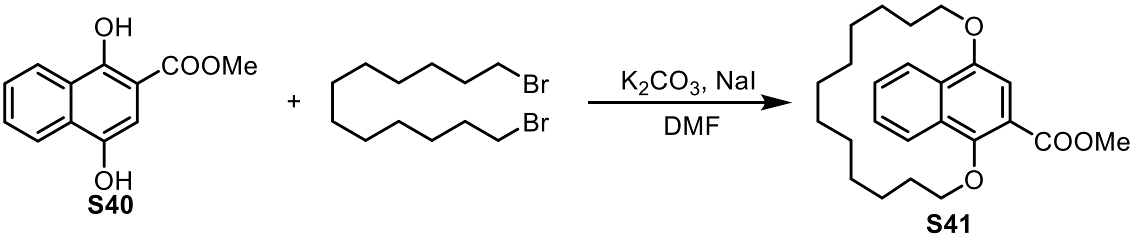

The title compound was prepared according to *General Procedure A* from **S40** (2.47 g, 12.00 mmol) and 1,12-dibromododecane (3.94 g, 12.00 mmol) in DMF (20 ml) were slowly added to a suspension of K_2CO_3 (4.15 g, 30.00 mmol) and NaI (198.0 mg, 1.32 mmol) in DMF (300 ml) at 140 °C, then mixture was stirred for overnight at 140 °C to afford **S41** as a yellow oil (2.12 g, 46%); ν_{max} (film)/cm^{-1} 2924 (C–H), 2853 (C–H), 1709 (C=O), 1435 (C=C), 1219 (C–O), 1088 (C–O); **^{1}H NMR** (500 MHz, $CDCl_3$) δ_H: 0.37-0.47 (1H, m, C*H*^{A}H^B), 0.47-0.56 (1H, m, CH^A*H*^{B}), 0.56-0.64 (1H, m, C*H*^{A}H^B), 0.64-0.74 (1H, m, CH^A*H*^{B}), 0.74-0.84 (4H, m, 2 × C*H*$_2$), 0.84-0.93 (2H, m, C*H*$_2$), 0.94-1.03 (2H, m, C*H*$_2$), 1.07-1.20 (2H, m, C*H*$_2$), 1.34-1.43 (1H, m, C*H*^{A}H^B), 1.44-1.58 (2H, m, C*H*$_2$), 1.69-1.96 (3H, m, C*H*$_2$, CH^A*H*^{B}), 4.01 (3H, s, OC*H*$_3$), 4.10 (1H, ddd, *J* 12.0, *J* 8.6, *J* 4.8, ArC(4)-OC*H*^{A}H^B), 4.39 (1H, ddd, *J* 10.7, *J* 6.0, *J* 4.3, ArC(4)-OCH^A*H*^{B}), 4.47 (1H, ddd, *J* 11.7, *J* 8.3, *J* 3.7, ArC(1)-OC*H*^{A}H^B), 4.54 (1H, dt, *J* 12.0, *J* 5.3, ArC(1)-OCH^A*H*^{B}), 7.23 (1H, s, ArC*H*), 7.57 (2H, m, 2 × ArC*H*), 8.27 (1H, dd, *J* 7.7, *J* 1.7, ArC*H*), 8.38 (1H, dd, *J* 7.3, *J* 1.4, ArC*H*); **^{13}C{1H} NMR** (126 MHz, $CDCl_3$) δ_C: 24.1 (CH_2), 24.8 (CH_2), 26.3 (CH_2), 27.4 (CH_2), 27.4 (CH_2), 27.6 (CH_2), 27.8 (CH_2), 27.8 (CH_2), 27.8 (CH_2), 29.3 (CH_2), 52.3 (OCH_3), 68.0 (ArC(4)-OCH_2), 75.7 (ArC(1)OCH_2), 106.5 (ArCH), 117.0 (Ar*C*), 121.9 (ArCH), 124.1 (ArCH), 126.5 (ArCH), 127.7 (ArCH), 129.2 (Ar*C*), 130.1 (Ar*C*), 149.8 (Ar*C*), 151.9 (Ar*C*), 167.5 (*C*=O); HRMS (APCI) $C_{24}H_{33}O_4$ ([M]) requires 385.23734, found 385.2370 (–0.91 ppm).

Synthesis of 2,15-dioxa-1(1,4)-naphthalenacyclopentadecaphane-1^2-ylmethanol S42

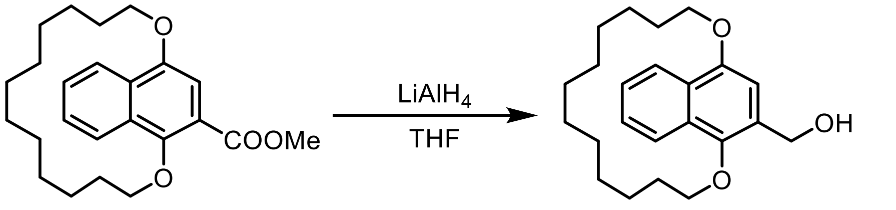

S41 **S42**

To a solution of **S41** (1.20 g, 3.12 mmol) in dry THF (30 ml) was slowly added $LiAlH_4$ (2.4 M, 2.6 ml, 6.24 mmol) at 0 °C and stirring for 10 min, then warm to room temperature and stirring for 6 h. The solution was quenched by saturated NH_4Cl (50 ml) and extracted with EtOAc (3 × 30 ml). The combined organic phase was washed by brine and dried over anhydrous Na_2SO_4, filtered, and concentrated in *vacuo*. The crude product **S42** (1.09 g, 98%) was used for next step without any purification.

Synthesis of 2,15-dioxa-1(1,4)-naphthalenacyclopentadecaphane-1^2-carbaldehyde S43

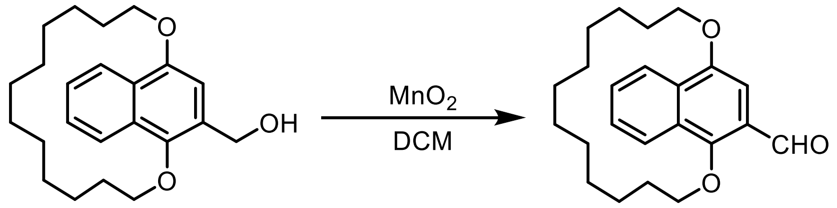

S42 **S43**

Following the method described by Latour *et al.*,[60] to the suspension of MnO_2 (2.61 g, 30.00 mmol) in dry dichloromethane (50 mL) was added **S42** (1.07 g, 3.00 mmol) at room temperature, then the mixture was heated to refluxed and stirring for 2 h. After cooling to room temperature, filtration over celite, and concentrated in *vacuo*. The crude product **S43** (0.97 g, 91%) was used for next step without any purification.

2.5 General Procedure C: Synthesis of macrocycle 2,5-dioxa-phenol derivatives **1, 10, S44-S52, S57-S67, S69**

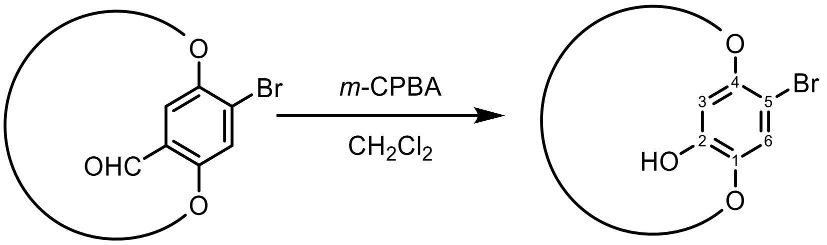

Following the method described by Pedulli *et al.*,[61] to a solution of aldehyde (1.0 equiv.) in CH_2Cl_2 (0.3 M) was slowly added *m*-CPBA (70% wt, 1.3 equiv.) and stirring for 8 h at room temperature. After totally consumed of starting materials, solution was concentrated in *vacuo*. The residue was dissolved in MeOH and stirred with excess 10% aqueous NaOH for 3 h. The mixture was acidified to pH 1 with 1 M aqueous HCl and extracted with CH_2Cl_2 (3 × 30 ml). The combined organic phase was washed by brine and dried over anhydrous Na_2SO_4, filtered, and concentrated in *vacuo*. The residue was purified by column chromatography (5%-15% EtOAc/Petrol ether) to afford phenol derivatives **1, 10, S44-S52, S57-S67, S69**.

2.6 General Procedure D: Synthesis of macrocycle 2,5-dioxa-phenol derivatives **36, S53-S56**

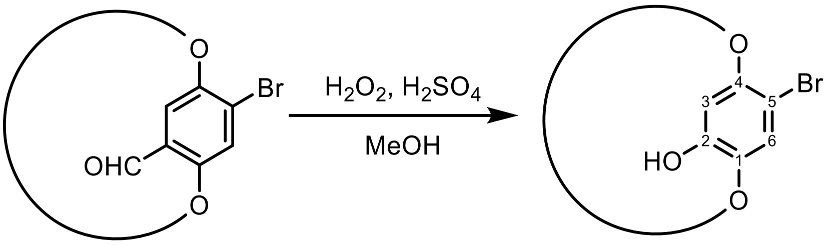

Following the method described by Woodward *et al.*,[62] to a solution of aldehyde (1.0 equiv.) in methanol (0.2 M) hydrogen peroxide (35% wt, 1.6 equiv.) and sulfuric acid (0.3 equiv.) were added dropwise and stirring for 20 h at room temperature. The reaction was extracted with CH_2Cl_2 (3 × 20 ml). The combined organic phase was washed by brine and dried over anhydrous Na_2SO_4, filtered, and concentrated in *vacuo*. The residue was purified by column chromatography (5%-15% EtOAc/Petrol ether) to afford phenol derivatives **36, S53-S56**.

Synthesis of 1^5-bromo-2,13-dioxa-1(1,4)-benzenacyclotridecaphan-1^2-ol 1

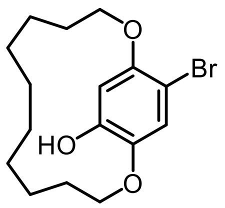

The title compound was prepared according to *General Procedure* C from **S10** (0.43 g, 1.20 mmol) and *m*-CPBA (0.39 g, 1.56 mmol, 70% wt) in CH_2Cl_2 (4 ml) were stirring for 8 h at room temperature to afford **1** as a white solid (0.31 g, 77%); mp 63–67 °C (EtOAc/Hexane); ν_{max} (film)/cm^{-1} 3503 (O–H), 2926 (C–H), 2853 (C–H), 1489 (C=C), 1187 (C–O), 1155 (C–O); **^{1}H NMR** (500 MHz, $CDCl_3$) δ_H: 0.73-0.88 (4H, m, 2 × C*H*$_2$), 0.93-1.05 (2H, m, C*H*$_2$), 1.06-1.34 (6H, m, 3 × C*H*$_2$), 1.55-1.65 (2H, m, C*H*$_2$), 1.69-1.78 (2H, m, C*H*$_2$), 4.25 (2H, ddd, *J* 12.0, *J* 8.3, *J* 3.8, ArC(1)-OC*H*$_2$), 4.33 (2H, ddd, *J* 12.9, *J* 6.8, *J* 3.9, ArC(4)-OC*H*$_2$), 5.79 (1H, s, ArC(2)-O*H*), 6.71 (1H, s, ArC(3)*H*), 7.13 (1H, s, ArC(6)*H*); **^{13}C{1H} NMR** (126 MHz, $CDCl_3$) δ_C: 23.6 (*C*H$_2$), 23.7 (*C*H$_2$), 27.3 (*C*H$_2$), 27.4 (*C*H$_2$), 27.4 (*C*H$_2$), 27.5 (*C*H$_2$), 27.6 (*C*H$_2$), 27.9 (*C*H$_2$), 70.1 (ArC(4)-OCH$_2$), 70.7 (ArC(1)-OCH$_2$), 103.5 (Ar*C*(5)), 105.9 (Ar*C*(3)H), 121.1 (Ar*C*(6)H), 140.0 (Ar*C*(1)), 147.5 (Ar*C*(2)), 150.3 (Ar*C*(4)); HRMS (APCI$^+$) $C_{16}H_{23}BrO_3$ ([M+Na]$^+$) requires 365.07228, found 365.0723 (+0.11 ppm).

Synthesis of 1^5-phenyl-2,13-dioxa-1(1,4)-benzenacyclotridecaphan-1^2-ol S44

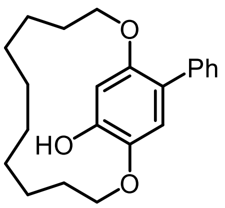

The title compound was prepared according to *General Procedure* C from **S20** (0.42 g, 1.20 mmol) and *m*-CPBA (0.39 g, 1.56 mmol, 70% wt) in CH_2Cl_2 (4 ml) were stirring for 8 h at room temperature to afford **S44** as a colourless oil (0.30 g, 74%); ν_{max} (film)/cm^{-1} 3520 (O–H), 2928 (C–H), 2855 (C–H), 1487 (C=C), 1263 (C–O), 1140 (C–O); **^{1}H NMR** (500 MHz, $CDCl_3$) δ_H: 0.77-1.06 (7H, m, 3 C*H*$_2$, C*H*^{A}H^B), 1.06-1.42 (6H, m, 3 × C*H*$_2$), 1.54-1.74 (2H, m, C*H*$_2$), 1.80-1.95 (1H, m, CH^A*H*^{B}), 3.86 (1H, ddd, *J* 12.3, *J* 8.4, *J* 4.0, ArC(4)-OC*H*$_A$H$_B$), 4.10 (1H, ddd, *J* 12.2, *J* 6.0, *J* 4.2, ArC(4)-OCH^A*H*^{B}), 4.27 (1H, ddd, *J* 12.1, *J* 8.3, *J* 3.9, ArC(1)-OC*H*^{A}H^B), 4.43 (1H, ddd, *J* 12.0, *J* 6.3, *J* 3.9, ArC(1)-OC*H*^{A}H^B), 5.81 (1H, s, ArC(2)-O*H*), 6.77 (1H, s, ArC(3)*H*), 6.97 (1H, s, ArC(6)*H*), 7.32 (1H, t, *J* 7.4, ArC(5)-PhC(4)*H*), 7.42 (2H, t, *J* 7.6, ArC(5)-PhC(3,5)*H*), 7.53 (2H, d, *J* 7.2, ArC(5)-PhC(2,6)*H*); **^{13}C{1H} NMR** (126 MHz, $CDCl_3$) δ_C: 23.6 (*C*H$_2$), 24.1 (*C*H$_2$), 27.2 (*C*H$_2$), 27.3 (*C*H$_2$), 27.4 (*C*H$_2$), 27.5 (*C*H$_2$), 27.7 (*C*H$_2$), 27.9 (*C*H$_2$), 69.8 (ArC(4)-OCH$_2$), 70.3 (ArC(1)-OCH$_2$), 106.8 (ArC(3)H), 118.7 (Ar*C*(6)H), 125.1 (Ar*C*(5)), 126.5

(ArC(5-Ph*C*(4)H), 128.1 (ArC(5)-Ph*C*(3,5)H), 129.3 (ArC(5)-Ph*C*(2,6)H), 138.8 (ArC(5)-Ph*C*(1)), 139.6 (Ar*C*(1)), 147.2 (Ar*C*(2)), 151.0 (Ar*C*(4)); HRMS (APCI+) $C_{22}H_{28}O_3$ ([M+Na]+) requires 363.19307, found 363.1930 (–0.11 ppm).

Synthesis of 1⁵-bromo-2,14-dioxa-1(1,4)-benzenacyclotetradecaphan-1²-ol S45

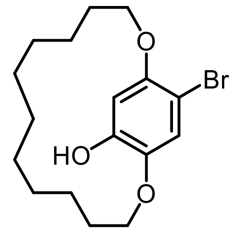

The title compound was prepared according to *General Procedure* C from **S11** (0.44 g, 1.20 mmol) and *m*-CPBA (0.39 g, 1.56 mmol, 70% wt) in CH_2Cl_2 (4 ml) were stirring for 8 h at room temperature to afford **S45** as a white solid (0.35 g, 81%); mp 53–56 °C (EtOAc/Hexane); ν_{max} (film)/cm^{-1} 3495 (O–H), 2922 (C–H), 2851 (C–H), 1489 (C=C), 1153 (C–O), 1049 (C–O); **¹H NMR** (500 MHz, $CDCl_3$) δ_H: 0.74-0.88 (2H, m, C*H*2), 0.93-1.12 (6H, m, 3 × C*H*2), 1.14-1.38 (6H, m, 3 × C*H*2), 1.57-1.77 (4H, m, 2 × C*H*2), 1.69-1.78 (2H, m, C*H*2), 4.21-4.38 (4H, m, ArC(1,4)-OC*H*2), 5.79 (1H, s, ArC(2)-O*H*), 6.70 (1H, s, ArC(3)*H*), 7.13 (1H, s, ArC(6)*H*); **¹³C{1H} NMR** (126 MHz, $CDCl_3$) δ_C: 23.8 (*C*H2), 23.9 (*C*H2), 27.0 (*C*H2), 27.1 (*C*H2), 27.2 (*C*H2), 28.5 (*C*H2), 28.5 (*C*H2), 28.5 (*C*H2), 28.6 (*C*H2), 69.9 (ArC(4)-O*C*H2), 70.5 (ArC(1)-O*C*H2), 102.5 (Ar*C*(5)), 104.5 (Ar*C*(3)H), 120.1 (Ar*C*(6)H), 140.3 (Ar*C*(1)), 147.0 (Ar*C*(2)), 150.6 (Ar*C*(4)); HRMS (APCI+) $C_{16}H_{23}BrO_3$ ([M+Na]+) requires 379.08793, found 379.0877 (–0.61 ppm).

Synthesis of 1⁵-phenyl-2,14-dioxa-1(1,4)-benzenacyclotetradecaphan-1²-ol S46

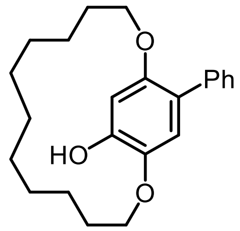

The title compound was prepared according to *General Procedure* C from **S21** (0.44 g, 1.20 mmol) and *m*-CPBA (0.39 g, 1.56 mmol, 70% wt) in CH_2Cl_2 (4 ml) were stirring for 8 h at room temperature to afford **S46** as a colourless oil (0.33 g, 77%); ν_{max} (film)/cm^{-1} 3493 (O–H), 2924 (C–H), 2853 (C–H), 1487 (C=C), 1165 (C–O), 1142 (C–O); **¹H NMR** (500 MHz, $CDCl_3$) δ_H: 0.83-0.93 (2H, m, C*H*2), 0.93-1.19 (9H, m, 4 × C*H*2, C*H*^A^H^B^), 1.21-1.35 (3H, m, C*H*2, CH^A^*H*^B^), 1.38-1.48 (1H, m, C*H*^A^H^B^), 1.53-1.60 (1H, m, CH^A^*H*^B^), 1.61-1.70 (1H, m, C*H*^A^H^B^),

1.74-1.85 (1H, m, CH^AH^B), 4.01 (1H, ddd, *J* 11.8, *J* 7.3, *J* 4.1, ArC(4)-OCH^AH^B), 4.19 (1H, ddd, *J* 12.0, *J* 7.1, *J* 3.8, ArC(4)-OCH^AH^B), 4.29 (1H, ddd, *J* 11.8, *J* 7.4, *J* 4.1, ArC(1)-OCH^AH^B), 4.40 (1H, ddd, *J* 11.3, *J* 7.0, *J* 3.9, ArC(1)-OCH^AH^B), 5.82 (1H, s, ArC(2)-O*H*), 6.75 (1H, s, ArC(3)*H*), 6.95 (1H, s, ArC(6)*H*), 7.29-7.35 (1H, m, ArC(5)-PhC(4)*H*), 7.38-7.45 (2H, m, ArC(5)-PhC(3,5)*H*), 7.48-7.55 (2H, m, ArC(5)-PhC(2,6)*H*); **^{13}C{1H} NMR** (126 MHz, $CDCl_3$) δ_C: 24.1 (CH_2), 24.1 (CH_2), 27.0 (CH_2), 27.2 (CH_2), 27.4 (CH_2), 28.5 (4 × CH_2), 69.4 (ArC(4)-OCH_2), 70.1 (ArC(1)-OCH_2), 104.6 (Ar*C*(3)H), 117.8 (Ar*C*(5)H), 124.3 (Ar*C*(5)), 126.4 (ArC(5)-Ph*C*(4)H), 128.0 (ArC(5)-Ph*C*(3,5)H), 129.5 (ArC(5)-Ph*C*(2,6)H), 138.9 (ArC(5)-Ph*C*(1)), 139.6 (Ar*C*(1)), 146.9 (Ar*C*(2)), 150.8 (Ar*C*(4)); HRMS (APCI$^+$) $C_{23}H_{30}O_3$ ([M+Na]$^+$) requires 377.20872, found 377.2084 (–0.83 ppm).

Synthesis of 1^5-bromo-2,15-dioxa-1(1,4)-benzenacyclopentadecaphan-1^2-ol S47

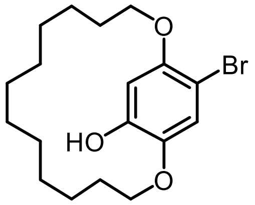

The title compound was prepared according to *General Procedure* C from **S12** (0.46 g, 1.20 mmol) and *m*-CPBA (0.39 g, 1.56 mmol, 70% wt) in CH_2Cl_2 (4 ml) were stirring for 8 h at room temperature to afford **S47** as a white solid (0.37 g, 83%); mp 49–52 °C (EtOAc/Hexane); ν_{max} (film)/cm^{-1} 3524 (C–H), 2924 (C–H), 2853 (C–H), 1761 (C=O), 1489 (C=C), 1157 (C–O), 1043 (C–O); **^{1}H NMR** (500 MHz, $CDCl_3$) δ_H: 0.94-1.07 (8H, m, 4 × C*H*$_2$), 1.10-1.39 (7H, m, 3 × C*H*$_2$, CH^AH^B), 1.41-1.51 (1H, m, CH^AH^B), 1.53-1.63 (2H, m, C*H*$_2$), 1.73-1.86 (2H, m, C*H*$_2$), 4.22 (2H, ddd, *J* 11.9, *J* 7.8, *J* 3.5, ArC(1)-OC*H*$_2$), 4.31 (2H, dd, *J* 10.7, *J* 5.4, ArC(4)-OC*H*$_2$), 5.72 (1H, s, ArC(2)-O*H*), 6.67 (1H, s, ArC(3)*H*), 7.10 (1H, s, ArC(6)*H*); **^{13}C{1H} NMR** (126 MHz, $CDCl_3$) δ_C: 23.9 (CH_2), 23.9 (CH_2), 26.8 (CH_2), 27.0 (CH_2), 27.5 (CH_2), 27.5 (CH_2), 27.8 (2 × CH_2), 28.4 (CH_2), 28.4 (CH_2), 69.3 (ArC(4)-OCH_2), 69.7 (ArC(1)-OCH_2), 101.8 (Ar*C*(5)), 103.3 (Ar*C*(3)H), 119.0 (Ar*C*(6)H), 139.1 (Ar*C*(1)), 146.8 (Ar*C*(2)), 149.4 (Ar*C*(4)); HRMS (APCI$^+$) $C_{18}H_{27}O_3Br$ ([M+Na]$^+$) requires 393.10358, found 393.1036 (+0.08 ppm).

Synthesis of 1^5-phenyl-2,15-dioxa-1(1,4)-benzenacyclopentadecaphan-1^2-ol 10

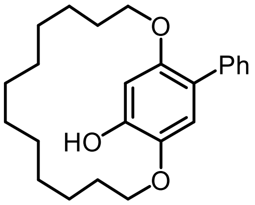

The title compound was prepared according to *General Procedure* C from **S22** (0.45 g, 1.20 mmol) and *m*-CPBA (0.39 g, 1.56 mmol, 70% wt) in CH_2Cl_2 (4 ml) were stirring for 8 h at room temperature to afford **10** as a colorless solid (0.32 g, 73%); mp 34–37 °C (EtOAc/Hexane); ν_{max} (film)/cm^{-1} 3526 (O–H), 2924 (C–H), 2853 (C–H), 1489 (C=C), 1173 (C–O), 1141 (C–O); **^{1}H NMR** (500 MHz, $CDCl_3$) δ_H: 1.01-1.07 (7H, m, 3 × C*H*$_2$, C*H*^{A}H^B), 1.09-1.32 (7H, m, 3 × C*H*$_2$, CH^A*H*^{B}), 1.32-1.40 (2H, m, C*H*$_2$), 1.40-1.51 (1H, m, C*H*^{A}H^B), 1.55-1.65 (1H, m, CH^A*H*^{B}), 1.66-1.76 (1H, m, C*H*^{A}H^B), 1.81-1.92 (1H, m, CH^A*H*^{B}), 3.96 (1H, ddd, *J* 11.8, *J* 8.2, *J* 4.6, ArC(4)-OC*H*^{A}H^B), 4.18 (1H, ddd, *J* 11.8, *J* 6.1, *J* 4.7, ArC(4)-OCH^A*H*^{B}), 4.23 (1H, ddd, *J* 11.8, *J* 8.4, *J* 4.4, ArC(1)-OC*H*^{A}H^B), 4.38 (1H, ddd, *J* 11.8, *J* 6.0, *J* 4.6, ArC(1)-OCH^A*H*^{B}), 5.79 (1H, s, ArC(2)-O*H*), 6.72 (1H, s, ArC(3)*H*), 6.92 (1H, s, ArC(6)*H*), 7.29-7.34 (1H, m, ArC(5)-PhC(4)*H*), 7.38-7.45 (2H, m, ArC(5)-PhC(3,5)*H*), 7.49-7.55 (2H, m, ArC(5)-PhC(2,6)*H*); **^{13}C{1H} NMR** (126 MHz, $CDCl_3$) δ_C: 23.9 (*C*H$_2$), 24.0 (*C*H$_2$), 27.0 (*C*H$_2$), 27.2 (*C*H$_2$), 27.2 (*C*H$_2$), 27.4 (*C*H$_2$), 27.7 (*C*H$_2$), 27.8 (*C*H$_2$), 27.9 (*C*H$_2$), 28.5 (*C*H$_2$), 68.7 (ArC(4)-O*C*H$_2$), 69.4 (ArC(1)-O*C*H$_2$), 103.3 (Ar*C*(3)H), 117.1 (Ar*C*(6)H), 123.6 (Ar*C*(5)), 126.4 (ArC(5)-Ph*C*(4)H), 127.9 (ArC(5)-Ph*C*(3,5)H), 129.5 (ArC(5)-Ph*C*(2,6)H), 138.7 (Ar*C*(1)), 138.9 (ArC(5)-Ph*C*(1)), 146.7 (Ar*C*(2)), 149.9 (Ar*C*(4)); HRMS (APCI$^+$) $C_{24}H_{32}O_3$ ([M+Na]$^+$) requires 391.22437, found 391.2243 (–0.22 ppm).

Synthesis of 1^5-(4-methoxyphenyl)-2,15-dioxa-1(1,4)-benzenacyclopentadecaphan-1^2-ol S48

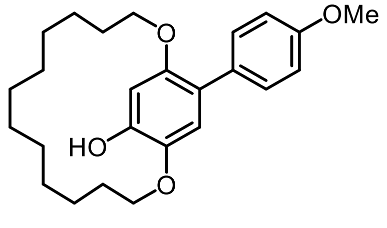

The title compound was prepared according to *General Procedure* C from **S23** (0.49 g, 1.20 mmol) and *m*-CPBA (0.39 g, 1.56 mmol, 70% wt) in CH_2Cl_2 (4 ml) were stirring for 8 h at room temperature to afford **S48** as a colourless oil (0.37 g, 78%); ν_{max} (film)/cm^{-1} 3526 (O–H), 2924 (C–H), 2853 (C–H), 1595 (C=C), 1487 (C=C), 1242 (C–O), 1175 (C–O); **^{1}H NMR** (500 MHz, $CDCl_3$) δ_H: 0.99-1.05 (7H, m, 3 × C*H*$_2$, C*H*^{A}H^B), 1.08-1.31 (7H, m, 3 × C*H*$_2$, CH^A*H*^{B}), 1.32-1.39 (2H, m, C*H*$_2$), 1.39-1.49 (1H, m, C*H*^{A}H^B), 1.54-1.63 (1H, m, CH^A*H*^{B}), 1.66-1.78 (1H, m, C*H*^{A}H^B), 1.80-1.91 (1H, m, CH^A*H*^{B}), 3.87 (3H, s, OC*H*$_3$), 3.96 (1H, ddd, *J* 11.8, *J* 8.2, *J* 4.6, ArC(4)-OC*H*^{A}H^B), 4.17 (1H, ddd, *J* 11.8, *J* 6.0, *J* 4.7, ArC(4)-OCH^A*H*^{B}), 4.22 (1H, ddd, *J* 11.8, *J* 8.3, *J* 4.5, ArC(1)-OC*H*^{A}H^B), 4.37 (1H, ddd, *J* 11.7, *J* 5.9, *J* 4.5, ArC(1)-OCH^A*H*^{B}), 5.76 (1H, s, ArC(2)-O*H*), 6.70 (1H, s, ArC(3)*H*), 6.89 (1H, s, ArC(6)*H*), 6.92-6.99 (2H, m, ArC(5)- Ar(3,5)*H*), 7.41-7.48 (2H, m, ArC(5)-ArC(2,4)*H*); **^{13}C{1H} NMR** (126 MHz, $CDCl_3$) δ_C: 23.9 (CH$_2$), 24.0 (CH$_2$), 27.0 (CH$_2$), 27.2 (CH$_2$), 27.4 (CH$_2$), 27.6 (CH$_2$), 27.7 (CH$_2$), 27.9 (CH$_2$), 28.5 (2 × CH$_2$), 55.3 (OCH$_3$), 68.7 (ArC(1)-OCH$_2$), 69.4 (ArC(4)-OCH$_2$), 103.4 (Ar*C*(3)H), 113.4 (ArC(5)-Ar*C*(3,5)H), 116.9

(ArC(6)H), 123.3 (ArC(5)), 130.5 (ArC(5)-ArC(2,6)H), 131.4 (ArC(5)-ArC(1)), 138.6 (ArC(1)), 146.3 (ArC(2)), 149.8 (ArC(4)), 158.2 (ArC(5)-ArC(4)); HRMS (APCI+) $C_{25}H_{34}O_4$ ($[M+Na]^+$) requires 421.23493, found 421.2348 (–0.41 ppm).

Synthesis of 1^5-(4-(trifluoromethyl)phenyl)-2,15-dioxa-1(1,4)-benzenacyclopentadecaphan-1^2-ol S49

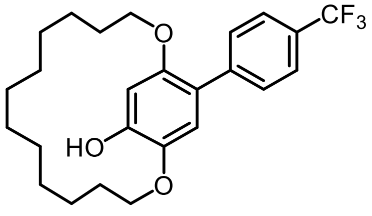

The title compound was prepared according to *General Procedure* C from **S24** (0.45 g, 1.00 mmol) and *m*-CPBA (0.33 g, 1.30 mmol, 70% wt) in CH_2Cl_2 (4 ml) were stirring for 8 h at room temperature to afford **S49** as a colorless solid (0.32 g, 73%); mp 33–35 °C (EtOAc/Hexane); ν_{max} (film)/cm^{-1} 3526 (O–H), 2926 (C–H), 2855 (C–H), 1499 (C=C), 1323 (C=C), 1123 (C–O), 729 (C–F); **^{1}H NMR** (500 MHz, $CDCl_3$) δ_H: 0.94-1.08 (8H, m, 4 × C*H*₂), 1.11-1.29 (6H, m, 3 × C*H*₂), 1.31-1.41 (2H, m, C*H*₂), 1.40-1.49 (1H, m, CH^AH^B), 1.56-1.65 (1H, m, CH^AH^B), 1.67-1.79 (1H, m, CH^AH^B), 1.80-1.94 (1H, m, CH^AH^B), 4.00 (1H, ddd, *J* 11.8, *J* 8.3, *J* 4.4, ArC(4)-OCH^AH^B), 4.19-4.29 (2H, m, ArC(4)-OCH^AH^B, ArC(1)-OCH^AH^B), 4.38 (1H, ddd, *J* 11.8, *J* 6.0, *J* 4.5, ArC(1)-OCH^AH^B), 5.86 (1H, s, ArC(2)-O*H*), 6.74 (1H, s, ArC(3)*H*), 6.91 (1H, s, ArC(6)*H*), 7.59-7.69 (4H, m, ArC(5)-Ar*H*); **^{13}C{1H} NMR** (126 MHz, $CDCl_3$) δ_C: 23.8 (CH_2), 23.9 (CH_2), 26.8 (CH_2), 27.2 (CH_2), 27.4 (CH_2), 27.6 (CH_2), 27.8 (CH_2), 27.9 (CH_2), 28.5 (2 × CH_2), 68.6 (ArC(1)-OCH_2), 69.5 (ArC(4)-OCH_2), 103.0 (ArC(3)H), 116.8 (ArC(6)H), 121.9 (ArC(5)), 124.5 (q, $^1J_{CF}$ 271.8, ArC(5)-ArCF_3), 124.8 (q, $^3J_{CF}$ 3.8, ArC(5)-ArC(3,5)H), 128.3 (q, $^2J_{CF}$ 32.3, ArC(5)-ArC(4)), 129.7 (ArC(5)-ArC(2,6)H), 138.7 (ArC(1)), 142.5 (ArC(5)-ArC(1)), 147.5 (ArC(2)), 150.0 (ArC(4)); **^{19}F{1H} NMR** (470 MHz, $CDCl_3$) δ_F: –62.28 (CF_3); HRMS (APCI+) $C_{25}H_{30}O_3F_3$ ($[M+Na]^+$) requires 435.21525, found 435.2151 (–0.35 ppm).

Synthesis of 1^5-(2-methoxyphenyl)-2,15-dioxa-1(1,4)-benzenacyclopentadecaphan-1^2-ol S50

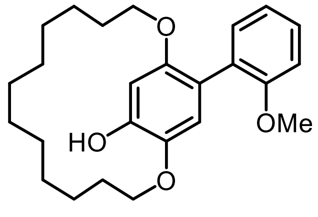

The title compound was prepared according to *General Procedure* C from **S25** (0.49 g, 1.20 mmol) and *m*-CPBA (0.39 g, 1.56 mmol, 70% wt) in CH_2Cl_2 (4 ml) were stirring for 8 h at room temperature to afford **S50** as a white solid (0.39 g, 82%); mp 67–70 °C (EtOAc/Hexane); ν_{max} (film)/cm^{-1} 3526 (O–H), 2924 (C–H), 2855 (C–H), 1597 (C=C), 1487 (C=C), 1217 (C–O), 1051 (C–O); **^{1}H NMR** (500 MHz, $CDCl_3$) δ_H: 1.02-1.31 (14H, m, 7 × CH_2), 1.33-1.43 (3H, m, CH_2, CH^AH^B), 1.48-1.58 (1H, m, CH^AH^B), 1.60-1.72 (1H, m, CH^AH^B), 1.86-1.98 (1H, m, CH^AH^B), 3.79 (3H, s, OCH_3), 3.86 (1H, ddd, *J* 11.7, *J* 7.9, *J* 5.1, ArC(4)-OCH^AH^B), 4.09 (1H, ddd, *J* 11.4, *J* 5.6, *J* 5.6, ArC(4)-OCH^AH^B), 4.19 (1H, ddd, *J* 11.7, *J* 8.9, *J* 4.1, ArC(1)-OCH^AH^B), 4.35 (1H, ddd, *J* 11.7, *J* 5.5, *J* 4.5, ArC(1)-OCH^AH^B), 5.76 (1H, s, ArC(2)-O*H*), 6.71 (1H, s, ArC(3)*H*), 6.85 (1H, s, ArC(6)*H*), 6.99 (1H, dd, *J* 8.4, *J* 1.1, ArC(5)-ArC(3)*H*), 7.02 (1H, dd, *J* 7.4, *J* 1.1, ArC(5)-ArC(5)*H*), 7.26-7.30 (1H, m, ArC(5)-ArC(6)*H*), 7.33 (1H, ddd, *J* 8.1, *J* 7.4, *J* 1.8, ArC(5)-ArC(4)*H*); **^{13}C{1H} NMR** (126 MHz, $CDCl_3$) δ_C: 23.5 (*C*H$_2$), 24.0 (*C*H$_2$), 26.8 (*C*H$_2$), 27.0 (*C*H$_2$), 27.4 (*C*H$_2$), 27.6 (*C*H$_2$), 27.8 (*C*H$_2$), 27.9 (*C*H$_2$), 28.4 (*C*H$_2$), 28.5 (*C*H$_2$), 55.6 (O*C*H$_3$), 68.8 (ArC(1)-O*C*H$_2$), 69.2 (ArC(4)-O*C*H$_2$), 103.4 (Ar*C*(3)H), 111.0 (ArC(5)-Ar*C*(3)H), 117.9 (Ar*C*(6)H), 120.1 (ArC(5)-Ar*C*(5)H), 120.3 (Ar*C*(5)), 127.9 (ArC(5)-Ar*C*(1)), 128.2 (ArC(5)-Ar*C*(4)H), 132.0 (ArC(5)-Ar*C*(6)H), 137.8 (Ar*C*(1)), 146.4 (Ar*C*(2)), 150.3 (Ar*C*(4)), 158.2 (ArC(5)-Ar*C*(2)); HRMS (APCI$^+$) $C_{25}H_{34}O_4$ ([M+Na]$^+$) requires 421.23493, found 421.2348 (–0.42 ppm).

Synthesis of 1^5-(naphthalen-1-yl)-2,15-dioxa-1(1,4)-benzenacyclopentadecaphan-1^2-ol S51

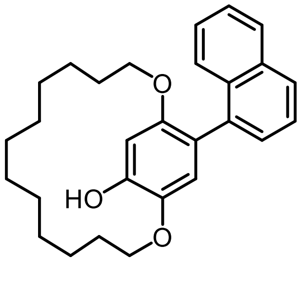

The title compound was prepared according to *General Procedure* C from **S26** (0.43 g, 1.00 mmol) and *m*-CPBA (0.33 g, 1.30 mmol, 70% wt) in CH_2Cl_2 (4 ml) were stirring for 8 h at room temperature to afford **S51** as a white solid (0.32 g, 76%); mp 108–112 °C (EtOAc/Hexane); ν_{max} (film)/cm^{-1} 3530 (O–H), 2924 (C–H), 2853 (C–H), 1506 (C=C), 1217 (C=C), 1175 (C–O); **major diastereomer** (NMR time scale 57:43 dr, could not found by HPLC): **^{1}H NMR** (500 MHz, $CDCl_3$) δ_H: 0.81-1.46 (10.2H, m, 8 × CH_2, CH^AH^B), 1.50-1.72 (1.2H, m, CH_2), 1.85-1.98 (0.6H, m, CH^AH^B), 3.73-3.79 (0.6H, m, ArC(4)-OCH^AH^B), 4.06 (0.6H, dt, *J* 12.1, *J* 5.9, *J* 5.9, ArC(4)-OCH^AH^B), 4.20 (0.6H, ddt, *J* 12.6, *J* 8.9, *J* 4.2, ArC(1)-OCH^AH^B), 4.33 (0.6H, ddd, *J* 12.4, *J* 9.8, *J* 4.9, ArC(1)-OCH^AH^B), 5.85 (0.6H, s, ArC(2)-O*H*), 6.78 (0.6H, s, ArC(3)*H*), 6.86 (0.6H, s, ArC(6)*H*), 7.37-7.57 (2.4H, m, ArC(5)-ArC(2,3,6,7)*H*), 7.70 (0.6H, d, *J* 8.4, ArC(5)-ArC(8)*H*), 7.84-7.88 (0.6H, m, ArC(5)-ArC(5)*H*), 7.89 (0.6H,

d, J 8.3, ArC(5)-ArC(4)H); **^{13}C{1H} NMR** (126 MHz, $CDCl_3$) δ_C: 23.7 (CH_2), 24.0 (CH_2), 27.1 (2 × CH_2), 27.5 (CH_2), 27.7 (2 × CH_2), 28.0 (CH_2), 28.6 (CH_2), 28.6 (CH_2), 68.3 (ArC(1)-OCH_2), 69.3 (ArC(4)-OCH_2), 103.0 (ArC(3)H), 118.0 (ArC(6)H), 122.2 (ArC(5)), 125.2 (ArC(5)-ArC(6)H), 125.4 (ArC(5)-ArC(3)H), 125.7 (ArC(5)-ArC(7)H), 126.9 (ArC(5)-ArC(8)H), 127.1 (ArC(5)-ArC(2)H), 127.4 (ArC(5)-ArC(4)H), 127.9 (ArC(5)-ArC(5)H), 132.6 (ArC(5)-ArC(8a)), 133.4 (ArC(5)-ArC(1)), 137.7 (ArC(5)-ArC(5a)), 138.3 (ArC(1)), 146.9 (ArC(2)), 150.4 (ArC(4)); HRMS (APCI$^+$) $C_{28}H_{34}O_3$ ([M+Na]$^+$) requires 441.24002, found 441.2393 (–1.56 ppm).

Minor diastereomer (NMR time scale 57:43 dr, could not found by HPLC): **^{1}H NMR** (500 MHz, $CDCl_3$) δ_H: 0.81-1.46 (6.8H, m, 8 × CH_2, CH^AH^B), 1.50-1.72 (0.8H, m, CH_2), 1.85-1.98 (0.4H, m, CH^AH^B), 3.65-3.73 (0.4H, m, ArC(4)-OCH^AH^B), 4.06 (0.4H, dt, J 12.1, J 5.9, J 5.9, ArC(4)-OCH^AH^B), 4.20 (0.4H, ddt, J 12.6, J 8.9, J 4.2, ArC(1)-OCH^AH^B), 4.33 (0.4H, ddd, J 12.4, J 9.8, J 4.9, ArC(1)-OCH^AH^B), 5.87 (0.4H, s, ArC(2)-OH), 6.84 (0.4H, s, ArC(3)H), 6.86 (0.4H, s, ArC(6)H), 7.37-7.57 (1.6H, m, ArC(5)-ArC(2,3,6,7)H), 7.84-7.88 (0.8H, m, 2 × ArC(4)-ArC(5,8)H), 7.93 (0.4H, d, J 8.2, ArC(4)-ArC(4)H); **^{13}C{1H} NMR** (126 MHz, $CDCl_3$) δ_C: 23.7 (CH_2), 24.3 (CH_2), 26.9 (CH_2), 27.4 (CH_2), 27.4 (CH_2), 27.8 (CH_2), 28.1 (CH_2), 28.2 (CH_2), 28.6 (CH_2), 28.6 (CH_2), 68.7 (ArC(1)-OCH_2), 69.7 (ArC(4)-OCH_2), 104.0 (ArC(3)H), 118.8 (ArC(6)H), 121.7 (ArC(5)), 125.7 (ArC(5)-ArC(6)H), 125.4 (ArC(5)-ArC(3)H), 126.2 (ArC(5)-ArC(7)H), 126.9 (ArC(5)-ArC(8)H), 127.2 (ArC(5)-ArC(2)H), 128.3 (ArC(5)-ArC(4)H), 128.4 (ArC(5)-ArC(5)H), 132.5 (ArC(5)-ArC(8a)), 133.9 (ArC(5)-ArC(1)), 136.3 (ArC(5)-ArC(5a)), 137.9 (ArC(1)), 146.8 (ArC(2)), 151.0 (ArC(4)).

Synthesis of 1^5-(naphthalen-2-yl)-2,15-dioxa-1(1,4)-benzenacyclopentadecaphan-1^2-ol S52

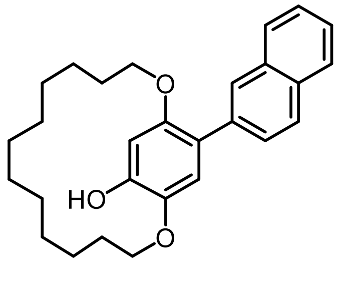

The title compound was prepared according to *General Procedure* C from **S27** (0.52 g, 1.20 mmol) and *m*-CPBA (0.39 g, 1.56 mmol, 70% wt) in CH_2Cl_2 (4 ml) were stirring for 8 h at room temperature to afford **S52** as a colourless oil (0.36 g, 72%); ν_{max} (film)/cm^{-1} 3520 (O–H), 2922 (C–H), 2853 (C–H), 1499 (C=C), 1281 (C=C), 1217 (C=C), 1171 (C–O), 1140 (C–O); **^{1}H NMR** (500 MHz, $CDCl_3$) δ_H: 0.97-1.37 (15H, m, 7 × CH_2, CH^AH^B), 1.39-1.48 (2H, m, CH_2), 1.59-1.77 (2H, m, CH_2), 1.83-1.96 (1H, m, CH^AH^B), 3.97 (1H, ddd, J 11.8, J 8.1, J 4.6, ArC(4)-OCH^AH^B), 4.20 (1H, ddd, J 11.8, J 6.0, J 4.7, ArC(4)-OCH^AH^B), 4.26 (1H, ddd, J 11.7, J 8.2, J 4.4, ArC(1)-OCH^AH^B), 4.42 (1H, ddd, J 11.8, J 6.1, J 4.6, ArC(1)-OCH^AH^B), 5.82 (1H, s, ArC(2)-OH), 6.77 (1H, s, ArC(3)H), 7.03 (1H, s,

ArC(6)*H*), 7.45-7.55 (2H, m, ArC(4)-ArC(6,7)*H*), 7.71 (1H, d, *J* 8.5, *J* 1.8, ArC(4)-ArC(3)*H*), 7.84-7.92 (3H, m, ArC(4)-ArC(4,5,8)*H*), 7.92-7.97 (1H, m, ArC(4)-ArC(1)*H*); **^{13}C{1H} NMR** (126 MHz, $CDCl_3$) δ_C: 23.9 (CH_2), 24.0 (CH_2), 27.0 (CH_2), 27.2 (CH_2), 27.4 (2 × CH_2), 27.7 (CH_2), 27.8 (CH_2), 28.0 (CH_2), 28.5 (2 × CH_2), 68.8 (ArC(1)-OCH_2), 69.5 (ArC(4)-OCH_2), 103.5 (Ar*C*(3)H), 117.3 (Ar*C*(6)H), 123.6 (Ar*C*(5)), 125.6 (ArC(5)-Ar*C*(6)H), 125.9 (ArC(5)-Ar*C*(7)H), 127.2 (ArC(5)-Ar*C*(5)H), 127.6 (ArC(5)-Ar*C*(8)H), 127.8 (ArC(5)-Ar*C*(3)H), 128.0 (ArC(5)-Ar*C*(4)H), 128.3 (ArC(5)-Ar*C*(1)H), 132.2 (ArC(4)-Ar*C*(5a)), 133.4 (ArC(4)-Ar*C*(8a)), 136.6 (ArC(4)-Ar*C*(2)), 138.8 (Ar*C*(2)), 146.9 (Ar*C*(1)), 150.2 (Ar*C*(5)); HRMS (APCI$^+$) $C_{28}H_{34}O_3$ ([M+Na]$^+$) requires 441.24002, found 441.2407 (+1.54 ppm).

Synthesis of 1^5-(pyridin-3-yl)-2,15-dioxa-1(1,4)-benzenacyclopentadecaphan-1^2-ol S53

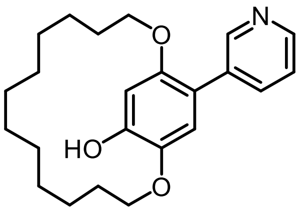

The title compound was prepared according to *General Procedure* D from **S28** (0.46 g, 1.20 mmol), hydrogen peroxide (167 µl, 1.92 mmol, 35% wt) and sulfuric acid (19 µl, 0.36 mmol) in methanol (6 ml) were stirring for 20 h at room temperature to afford **S53** as a yellow solid (0.16 g, 36%); mp 38–40 °C (EtOAc/Hexane); ν_{max} (film)/cm^{-1} 3524 (O–H), 2922 (C–H), 2853 (C–H), 1508 (C=C), 1298 (C=C), 1175 (C–O), 1155 (C–O); **^{1}H NMR** (500 MHz, $CDCl_3$) δ_H: 0.95-1.05 (8H, m, 4 × CH_2), 1.07-1.30 (6H, m, 3 × CH_2), 1.30-1.38 (2H, m, CH_2), 1.39-1.48 (1H, m, CH^AH^B), 1.55-1.65 (1H, m, CH^AH^B), 1.66-1.77 (1H, m, , CH^AH^B), 1.81-1.92 (1H, m, CH^AH^B), 4.01 (1H, ddd, *J* 12.1, *J* 8.3, *J* 4.3, ArC(4)-OCH^AH^B), 4.19-4.29 (2H, m, ArC(4)-OCH^AH^B, ArC(1)-OCH^AH^B), 4.38 (1H, ddd, *J* 11.9, *J* 5.9, *J* 4.6, ArC(1)-OCH^AH^B), 6.29 (1H, s, ArC(2)-O*H*), 6.73 (1H, s, ArC(3)*H*), 6.92 (1H, s, ArC(6)*H*), 7.33 (1H, ddd, *J* 7.9, *J* 4.8, *J* 0.8, ArC(5)-ArC(4)*H*), 7.87 (1H, dt, *J* 7.9, *J* 2.0, ArC(5)-ArC(5)*H*), 8.53 (1H, dd, *J* 4.8, *J* 1.7, ArC(5)-ArC(6)*H*), 8.75 (1H, d, *J* 2.3, ArC(5)-ArC(2)*H*); **^{13}C{1H} NMR** (126 MHz, $CDCl_3$) δ_C: 23.8 (CH_2), 23.9 (CH_2), 26.8 (CH_2), 27.1 (CH_2), 27.3 (CH_2), 27.6 (CH_2), 27.8 (CH_2), 27.9 (CH_2), 28.4 (CH_2), 28.5 (CH_2), 68.6 (ArC(1)-OCH_2), 69.5 (ArC(4)-OCH_2), 103.0 (Ar*C*(3)H), 116.8 (Ar*C*(6)H), 119.5 (Ar*C*(5)), 122.8 (ArC(5)-Ar*C*(5)), 134.6 (ArC(5)-Ar*C*(3)), 136.9 (ArC(5)-Ar*C*(4)H), 138.9 (Ar*C*(1)), 147.3 (ArC(5)-Ar*C*(6)H), 147.7 (Ar*C*(2)), 150.0 (ArC(5)-Ar*C*(2)H), 150.2 (Ar*C*(4)); HRMS (APCI$^+$) $C_{22}H_{28}O_3$ ([M+Na]$^+$) requires 370.23767, found 370.2374 (–0.76 ppm).

Synthesis of 1^5-(furan-2-yl)-2,15-dioxa-1(1,4)-benzenacyclopentadecaphan-1^2-ol S54

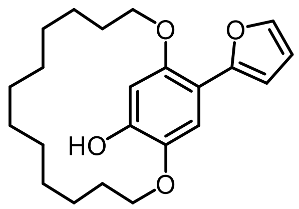

The title compound was prepared according to *General Procedure* D from **S29** (0.44 g, 1.20 mmol), hydrogen peroxide (167 µl, 1.92 mmol, 35% wt) and sulfuric acid (19 µl, 0.36 mmol) in methanol (6 ml) were stirring for 20 h at room temperature to afford **S54** as a green solid (0.22 g, 52%); mp 44–46 °C (EtOAc/Hexane); ν_{max} (film)/cm^{-1} 3541 (O–H), 2920 (C–H), 2853 (C–H), 1495 (C=C), 1219 (C=C), 1169 (C–O), 1153 (C–O); **^{1}H NMR** (500 MHz, $CDCl_3$) δ_H: 0.86-1.01 (8H, m, 4 × C*H*$_2$), 1.10-1.25 (4H, m, 2 × C*H*$_2$), 1.27-1.36 (4H, m, 2 × C*H*$_2$), 1.53-1.65 (2H, m, C*H*$_2$), 1.77-1.93 (2H, m, C*H*$_2$), 4.18-4.29 (2H, m, ArC(4)-OC*H*^{A}H^B, ArC(1)-OC*H*^{A}H^B), 4.37-4.49 (2H, m, ArC(4)-OCH^A*H*^{B}, ArC(1)-OCH^A*H*^{B}), 5.81 (1H, s, ArC(2)-O*H*), 6.49 (1H, t, *J* 2.6, ArC(5)-ArC(4)*H*), 6.68 (1H, s, ArC(3)*H*), 6.86 (1H, d, *J* 3.3, ArC(5)-ArC(3)*H*), 7.43 (1H, s, ArC(5)-ArC(5)*H*), 7.43 (1H, s, ArC(6)*H*); **^{13}C{1H} NMR** (126 MHz, $CDCl_3$) δ_C: 23.9 (*C*H$_2$), 24.0 (*C*H$_2$), 26.9 (*C*H$_2$), 27.0 (*C*H$_2$), 27.1 (*C*H$_2$), 27.4 (*C*H$_2$), 27.6 (*C*H$_2$), 27.8 (*C*H$_2$), 28.4 (*C*H$_2$), 28.5 (*C*H$_2$), 68.2 (ArC(1)-O*C*H$_2$), 69.5 (ArC(4)-O*C*H$_2$), 102.0 (Ar*C*(3)H), 108.0 (ArC(5)-Ar*C*(3)H), 111.7 (ArC(5)-Ar*C*(4)H), 112.3 (Ar*C*(6)H), 113.2 (Ar*C*(5)), 138.4 (Ar*C*(1)), 140.0 (ArC(5)-Ar*C*(5)H), 146.6 (Ar*C*(2)), 149.1 (Ar*C*(4)), 150.6 (ArC(5)-Ar*C*(2)); HRMS (APCI$^+$) $C_{22}H_{30}O_4$ ([M+Na]$^+$) requires 381.20363, found 381.2036 (–0.04 ppm).

Synthesis of 1^5-(thiophen-3-yl)-2,15-dioxa-1(1,4)-benzenacyclopentadecaphan-1^2-ol S55

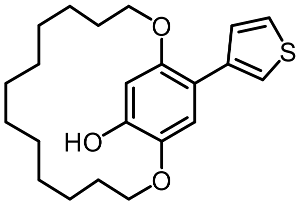

The title compound was prepared according to *General Procedure* D from **S30** (0.39 g, 1.00 mmol), hydrogen peroxide (139 µl, 1.60 mmol, 35% wt) and sulfuric acid (16 µl, 0.30 mmol) in methanol (5 ml) were stirring for 20 h at room temperature to afford **S55** as a colourless solid (0.27 g, 72%); mp 32–34 °C (EtOAc/Hexane); ν_{max} (film)/cm^{-1} 3524 (O–H), 2924 (C–H), 2853 (C–H), 1497 (C=C), 1215 (C=C), 1169 (C–O); **^{1}H NMR** (500 MHz, $CDCl_3$) δ_H: 0.92-1.05 (8H, m, 4 × C*H*$_2$), 1.09-1.37 (8H, m, 4 × C*H*$_2$), 1.47-1.56 (1H, m, C*H*^{A}H^B), 1.56-1.63 (1H, m, CH^A*H*^{B}), 1.74-1.89 (2H, m, C*H*$_2$), 4.10 (1H, ddd, *J* 11.8, *J* 8.4, *J* 4.3, ArC(4)-OC*H*^{A}H^B), 4.20-4.32 (2H, m, ArC(1)-OC*H*^{A}H^B, ArC(4)-OCH^A*H*^{B}), 4.38 (1H, ddd, *J* 11.8, *J* 6.0, *J* 4.6, ArC(1)-OCH^A*H*^{B}), 5.78 (1H, s, ArC(2)-O*H*), 6.70

(1H, s, ArC(3)*H*), 7.07 (1H, s, ArC(6)*H*), 7.34 (1H, dd, *J* 5.0, *J* 3.0, ArC(5)-ArC(2)*H*), 7.41 (1H, dd, *J* 5.0, *J* 1.3, ArC(5)-ArC(4)*H*), 7.52 (1H, dd, *J* 3.0, *J* 1.3, ArC(5)-ArC(5)*H*); **^{13}C{1H} NMR** (126 MHz, $CDCl_3$) δ_C: 23.9 (CH_2), 24.0 (CH_2), 26.9 (CH_2), 27.2 (CH_2), 27.4 (CH_2), 27.6 (CH_2), 27.7 (CH_2), 27.9 (CH_2), 28.4 (CH_2), 28.4 (CH_2), 68.5 (ArC(1)-OCH_2), 69.6 (ArC(4)-OCH_2), 102.8 (Ar*C*(3)H), 116.1 (Ar*C*(6)H), 118.0 (Ar*C*(5)), 122.0 (ArC(5)-Ar*C*(2)H), 124.2 (ArC(5)-Ar*C*(4)H), 128.5 (ArC(5)-Ar*C*(5)H), 138.4 (Ar*C*(1)), 138.6 (ArC(5)-Ar*C*(3)), 146.6 (Ar*C*(2)), 150.0 (Ar*C*(4)); HRMS (APCI$^+$) $C_{22}H_{30}O_3S$ ([M+Na]$^+$) requires 397.18079, found 397.1807 (–0.18 ppm).

Synthesis of *tert*-butyl 3-(1^5-hydroxy-2,15-dioxa-1(1,4)-benzenacyclopentadecaphane-1^2-yl)-5-methoxy-1H-indole-1-carboxylate S56

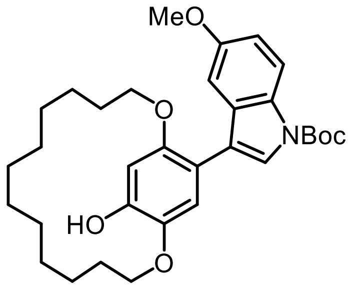

The title compound was prepared according to *General Procedure* D from **S31** (0.66 g, 1.20 mmol), hydrogen peroxide (167 µl, 1.92 mmol, 35% wt) and sulfuric acid (19 µl, 0.36 mmol) in methanol (6 ml) were stirring for 20 h at room temperature to afford **S56** as a white solid (0.45 g, 70%); mp 47–51 °C (EtOAc/Hexane); ν_{max} (film)/cm^{-1} 3526 (O–H), 2926 (C–H), 2855 (C–H), 1728 (C=O), 1474 (C=C), 1217 (C=C), 1125 (C–O); **^{1}H NMR** (500 MHz, $CDCl_3$) δ_H: 0.87-1.12 (12H, m, 6 × C*H*$_2$), 1.18-1.35 (5H, m, 2 × C*H*$_2$, C*H*^{A}H^B), 1.37 (9H, s, C(C*H*$_3$)$_3$) 1.58-1.70 (2H, m, C*H*$_2$), 1.85-1.95 (1H, m, CH^A*H*^{B}), 3.89 (3H, s, OC*H*$_3$), 3.93 (1H, m, ArC(4)-OC*H*^{A}H^B), 4.15 (1H, ddd, *J* 11.6, *J* 6.1, *J* 4.4, ArC(4)-OCH^A*H*^{B}), 4.23 (1H, ddd, *J* 11.8, *J* 8.3, *J* 4.4, ArC(1)-OC*H*^{A}H^B), 4.38 (1H, ddd, *J* 11.7, *J* 5.9, *J* 4.5, ArC(1)-OCH^A*H*^{B}), 5.81 (1H, s, ArC(2)-O*H*), 6.36 (1H, s, indoleC(2)*H*), 6.64 (1H, s, ArC(3)*H*), 6.94 (1H, dd, *J* 9.1, *J* 2.6, indoleC(6)*H*), 6.96 (1H, s, ArC(6)*H*), 7.05 (1H, d, *J* 2.6, indoleC(4)*H*), 8.10 (1H, d, *J* 9.0, indoleC(7)*H*); **^{13}C{1H} NMR** (126 MHz, $CDCl_3$) δ_C: 23.6 (CH_2), 23.9 (CH_2), 26.9 (CH_2), 27.1 (CH_2), 27.3 (CH_2), 27.6 ($C(CH_3)_3$), 27.7 (CH_2), 27.8 (CH_2), 28.0 (CH_2), 28.5 (2 × CH_2), 55.7 (*C*(CH_3)), 68.1 (ArC(4)-OCH_2), 69.4 (ArC(1)-OCH_2), 82.7 (OCH_3), 102.3 (Ar*C*(3)H), 102.8 (indole*C*(4)H), 108.5 (indole*C*(2)H), 112.4 (indole*C*(6)H), 115.7 (indole*C*(7)H), 116.4 (Ar*C*(6)H), 117.2 (Ar*C*(5)), 129.7 (indole*C*(7a)), 131.9 (indole*C*(4a)), 137.9 (Ar*C*(1)), 138.4 (indole*C*(3)), 147.5 (Ar*C*(2)), 150.3 (*C*=O), 150.6 (Ar*C*(4)), 155.6 (indole*C*(5)); HRMS (APCI$^+$) $C_{32}H_{43}O_6N$ ([M+Na]$^+$) requires 560.29826, found 560.2978 (–0.89 ppm).

Synthesis of 1^5-(pyren-1-yl)-2,15-dioxa-1(1,4)-benzenacyclopentadecaphan-1^2-ol S57

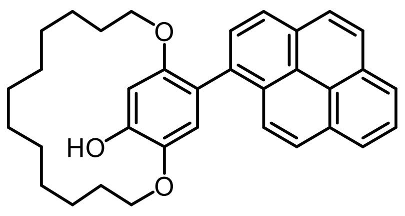

The title compound was prepared according to *General Procedure* C from **S32** (0.50 g, 1.00 mmol) and *m*-CPBA (0.33 g, 1.30 mmol, 70% wt) in CH_2Cl_2 (4 ml) were stirring for 8 h at room temperature to afford **S57** as a yellow solid (0.27 g, 55%); mp 60–64 °C (EtOAc/Hexane); ν$_{max}$ (film)/cm^{-1} 3503 (O–H), 2922 (C–H), 2851 (C–H), 1497 (C=C), 1281 (C=C), 1171 (C–O), 1146 (C–O); **major diastereomer** (NMR time scale 56:44 dr, could not found by HPLC): **^{1}H NMR** (500 MHz, $CDCl_3$) δ$_{H}$: 0.97-1.56 (10.2H, m, 8 × C*H*$_2$, C*H*^{A}H^{B}), 1.59-1.69 (1.2H, m, C*H*$_2$), 1.89-2.07 (0.6H, m, C*H*$_2$), 3.66-3.76 (0.6H, m, ArC(4)-OC*H*^{A}H^{B}), 4.01-4.11 (0.6H, m, ArC(4)-OCH^{A}*H*^{B}), 4.19-4.28 (0.6H, m, ArC(1)-OC*H*^{A}H^{B}), 4.32-4.43 (0.6H, m, ArC(1)-OCH^{A}*H*^{B}), 5.92 (0.6H, s, ArC(2)-O*H*), 6.86 (0.6H, s, ArC(3)*H*), 7.00 (0.6H, s, ArC(6)*H*), 7.87-8.33 (5.4H, m, 9 × ArC(5)-ArC*H*); **^{13}C{1H} NMR** (126 MHz, $CDCl_3$) δ$_{C}$: 23.7 (CH_2), 24.1 (CH_2), 26.9 (CH_2), 27.2 (CH_2), 27.3 (CH_2), 27.5 (CH_2), 27.8 (CH_2), 28.1 (CH_2), 28.6 (2 × CH_2), 68.5 (ArC(1)-OCH$_2$), 69.4 (ArC(4)-OCH$_2$), 103.1 (Ar*C*(3)H), 118.3 (Ar*C*(6)H), 122.6 (Ar*C*(5)), 124.4 (ArC(5)-Ar*C*H), 124.8 (ArC(5)-Ar*C*H), 124.9 (ArC(5)-Ar*C*H), 125.1 (ArC(5)-Ar*C*), 125.9 (ArC(5)-Ar*C*H), 125.9 (ArC(5)-Ar*C*H), 127.1 (ArC(5)-Ar*C*H), 127.2 (ArC(5)-Ar*C*H), 127.4 (ArC(5)-Ar*C*H), 127.9 (ArC(5)-Ar*C*H), 129.1 (ArC(5)-Ar*C*), 129.7 (ArC(5)-Ar*C*), 130.5 (ArC(5)-Ar*C*), 131.2 (ArC(5)-Ar*C*), 131.4 (ArC(5)-Ar*C*), 134.9 (ArC(5)-Ar*C*(1)), 138.6 (Ar*C*(1)), 147.1 (Ar*C*(2)), 150.6 (Ar*C*(4)); HRMS (APCI^{+}) $C_{34}H_{36}O_3$ ([M+Na]$^{+}$) requires 515.25567, found 515.2553 (–0.62 ppm).

Minor diastereomer (NMR time scale 56:44 dr, could not found by HPLC): **^{1}H NMR** (500 MHz, $CDCl_3$) δ$_{H}$: 0.97-1.56 (6.8H, m, 8 × C*H*$_2$, C*H*^{A}H^{B}), 1.59-1.69 (0.8H, m, C*H*$_2$), 1.89-2.07 (0.4H, m, C*H*$_2$), 3.66-3.76 (0.4H, m, ArC(4)-OC*H*^{A}H^{B}), 4.01-4.11 (0.4H, m, ArC(4)-OCH^{A}*H*^{B}), 4.19-4.28 (0.4H, m, ArC(1)-OC*H*^{A}H^{B}), 4.32-4.43 (0.4H, m, ArC(1)-OCH^{A}*H*^{B}), 5.93 (0.4H, s, ArC(2)-O*H*), 6.92 (0.4H, s, ArC(3)*H*), 6.97 (0.4H, s, ArC(6)*H*), 7.87-8.33 (3.6H, m, 9 × ArC(4)-ArC*H*); **^{13}C{1H} NMR** (126 MHz, $CDCl_3$) δ$_{C}$: 23.7 (CH_2), 24.3 (CH_2), 27.0 (CH_2), 27.2 (CH_2), 27.4 (CH_2), 27.8 (CH_2), 27.9 (CH_2), 28.3 (CH_2), 28.6 (CH_2), 28.7 (CH_2), 68.9 (ArC(1)-OCH$_2$), 69.6 (ArC(4)-OCH$_2$), 104.4 (Ar*C*(3)H), 119.1 (Ar*C*(6)H), 122.2 (Ar*C*(5)), 124.4 (ArC(5)-Ar*C*H), 124.7 (ArC(5)-Ar*C*H), 125.0 (ArC(5)-Ar*C*), 125.1 (ArC(5)-Ar*C*H), 125.7 (ArC(5)-Ar*C*H), 126.4 (ArC(5)-Ar*C*H), 126.9 (ArC(5)-Ar*C*H), 127.1 (ArC(5)-Ar*C*H), 127.6 (ArC(5)-Ar*C*H), 129.1 (ArC(5)-Ar*C*), 129.2 (ArC(5)-Ar*C*H), 129.7 (ArC(5)-Ar*C*), 130.3 (ArC(5)-Ar*C*), 131.0 (ArC(5)-Ar*C*), 131.5 (ArC(5)-Ar*C*), 134.4 (ArC(5)-Ar*C*(1)), 138.1 (Ar*C*(1)), 146.9 (Ar*C*(2)), 151.0 (Ar*C*(4)).

Synthesis of 2,15-dioxa-1(1,4)-naphthalenacyclopentadecaphan-1²-ol 36

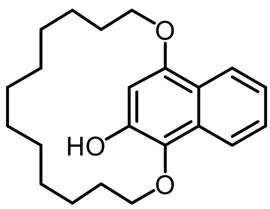

The title compound was prepared according to *General Procedure* D from **S43** (0.43 g, 1.20 mmol), hydrogen peroxide (167 µl, 1.92 mmol, 35% wt) and sulfuric acid (19 µl, 0.36 mmol) in methanol (6 ml) were stirring for 20 h at room temperature to afford **36** as an off-white solid (0.19 g, 46%); mp 62–64 °C (EtOAc/Hexane); ν_{max} (film)/cm^{-1} 3391 (O–H), 2924 (C–H), 2853 (C–H), 1626 (C=C), 1366 (C=C), 1088 (C–O); **¹H NMR** (500 MHz, $CDCl_3$) δ_H: 0.64-1.28 (14H, m, 7 × C*H*₂), 1.38-1.57 (2H, m, C*H*₂), 1.57-1.74 (3H, m, C*H*₂, C*H*^{A}H^B), 1.86-1.20 (1H, m, CH^A*H*^{B}), 4.18-4.40 (3H, m, ArC(4)-OC*H*₂, ArC(1)-OC*H*^{A}H^B), 4.44-4.53 (1H, m, ArC(1)-OCH^A*H*^{B}), 5.93 (1H, s, ArC(2)-O*H*), 6.68 (1H, s, ArC(3)*H*), 7.32 (1H, t, *J* 7.64, ArC(7)*H*), 7.49 (1H, t, *J* 7.6, ArC(6)*H*), 7.91 (1H, d, *J* 8.5, ArC(5)*H*), 8.21 (1H, d, *J* 8.4, ArC(8)*H*); **¹³C{1H} NMR** (126 MHz, $CDCl_3$) δ_C: 24.0 (CH_2), 24.4 (CH_2), 26.5 (CH_2), 27.0 (CH_2), 27.1 (CH_2), 27.2 (CH_2), 27.6 (CH_2), 27.7 (CH_2), 28.0 (CH_2), 28.2 (CH_2), 67.9 (ArC(4)-OCH_2), 72.7 (ArC(1)-OCH_2), 98.7 (Ar*C*(3)H), 120.5 (Ar*C*(6)H), 122.4 (Ar*C*(5a)), 122.5 (Ar*C*(7)H), 122.8 (Ar*C*(5)H), 126.6 (Ar*C*(8)H), 128.5 (Ar*C*(8a)), 130.9 (Ar*C*(1)), 145.9 (Ar*C*(2)), 151.1 (Ar*C*(4)); HRMS (APCI$^+$) $C_{22}H_{30}O_3$ ([M+Na]$^+$) requires 365.20872, found 365.2083 (−1.26 ppm).

Synthesis of 1⁵-bromo-2,6,11,15-tetraoxa-1(1,4)-benzenacyclopentadecaphan-1²-ol S58

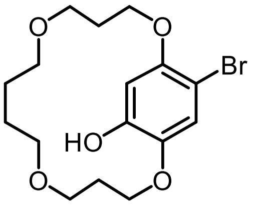

The title compound was prepared according to *General Procedure* C from **S13** (0.27 g, 0.70 mmol) and *m*-CPBA (0.23 g, 0.91 mmol, 70% wt) in CH_2Cl_2 (3 ml) were stirring for 8 h at room temperature to afford **S58** as a white solid (0.24 g, 88%); mp 105–108 °C (EtOAc/Hexane); ν_{max} (film)/cm^{-1} 3331 (O–H), 2935 (C–H), 2864 (C–H), 1494 (C=C), 1186 (C–O), 1107 (C–O), 1044 (C–O), 797 (C–Br); **¹H NMR** (500 MHz, $CDCl_3$) δ_H: 1.05-1.25 (4H, m, 2 × C*H*₂), 1.82-2.09 (4H, m, 2 × C*H*₂), 3.01-3.16 (2H, m, OC*H*₂), 3.30 (1H, ddd, *J* 9.9, *J* 7.8, *J* 5.7, OC*H*^{A}H^B), 3.30-3.48 (4H, m, 2 × OC*H*₂), 3.63 (1H, ddd, *J* 10.6, *J* 8.9, *J* 3.5, OCH^A*H*^{B}), 4.23-4.41 (4H, m, ArC(4)-OC*H*₂, ArC(1)-OC*H*₂), 5.75 (1H, s, ArC(2)-O*H*), 6.67 (1H, s, ArC(3)*H*), 7.11 (1H, s, ArC(6)*H*); **¹³C{1H} NMR** (126 MHz, $CDCl_3$)

δC: 25.8 (CH2), 25.8 (CH2), 30.3 (CH2), 31.0 (CH2), 65.6 (OCH2), 66.3 (OCH2), 67.9 (ArC(4)-OCH2), 68.9 (ArC(1)-OCH2), 70.9 (OCH2), 71.2 (OCH2), 101.5 (ArC(5)), 104.0 (ArC(3)H), 119.2 (ArC(6)H), 141.9 (ArC(1)), 146.6 (ArC(2)), 151.9 (ArC(4)); HRMS (APCI+) $C_{16}H_{23}O_5Br$ ([M+Na]+) requires 397.06211, found397.0621 (–0.07 ppm).

Synthesis of 15-bromo-2,16-dioxa-1(1,4)-benzenacyclohexadecaphan-12-ol S59

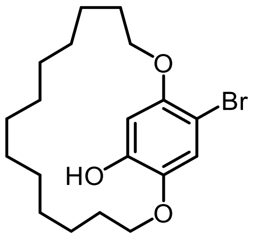

The title compound was prepared according to *General Procedure* C from **S14** (0.40 g, 1.00 mmol) and *m*-CPBA (0.33 g, 1.30 mmol, 70% wt) in CH_2Cl_2 (4 ml) were stirring for 8 h at room temperature to afford **S59** as a white solid (0.34 g, 88%); mp 42–44 °C (EtOAc/Hexane); ν_{max} (film)/cm^{-1} 3501 (O–H), 2926 (C–H), 2851 (C–H), 1497 (C=C), 1304 (C=C), 1163 (C–O); **^{1}H NMR** (500 MHz, $CDCl_3$) δ_H: 0.95-1.25 (12H, m, 6 × C*H*2), 1.25-1.43 (5H, m, 2 × C*H*2, C*H*^{A}H^B), 1.44-1.55 (1H, m, CH^A*H*^{B}), 1.57-1.64 (1H, m, C*H*2), 1.66-1.80 (2H, m, C*H*2), 4.11-4.29 (4H, m, ArC(1,4)-OC*H*2), 5.75 (1H, s, ArC(2)-O*H*), 6.65 (1H, s, ArC(3)*H*), 7.09 (1H, s, ArC(6)*H*); **^{13}C{1H} NMR** (126 MHz, $CDCl_3$) δ_C: 23.8 (CH2), 23.9 (CH2), 27.3 (CH2), 27.4 (CH2), 27.5 (CH2), 27.6 (CH2), 27.9 (CH2), 28.0 (CH2), 28.1 (CH2), 28.2 (CH2), 29.4 (CH2), 69.0 (ArC(4)-OCH2), 69.5 (ArC(1)-OCH2), 101.5 (ArC(5)), 102.6 (ArC(3)H), 118.5 (ArC(6)H), 139.3 (ArC(1)), 146.6 (ArC(2)), 149.6 (ArC(4)); HRMS (APCI+) $C_{19}H_{29}O_3Br$ ([M+Na]+) requires 407.11923, found 407.1190 (–0.64 ppm).

Synthesis of 15-phenyl-2,16-dioxa-1(1,4)-benzenacyclohexadecaphan-12-ol S60

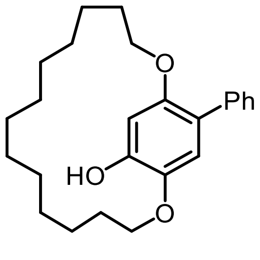

The title compound was prepared according to *General Procedure* C from **S33** (0.39 g, 1.00 mmol) and *m*-CPBA (0.33 g, 1.30 mmol, 70% wt) in CH_2Cl_2 (4 ml) were stirring for 8 h at room temperature to afford **S60** as a colorless oil (0.31 g, 81%); ν_{max} (film)/cm^{-1} 3526 (O–H), 2922 (C–H), 2853 (C–H), 1487 (C=C), 1285 (C=C), 1171 (C–O); **^{1}H NMR** (500 MHz, $CDCl_3$) δ_H: 1.00-1.39 (18H, m, 9 × C*H*2), 1.47-1.57 (1H, m, C*H*^{A}H^B), 1.60-1.70 (2H,

m, CH_2), 1.70-1.87 (1H, m, CH^AH^B), 3.91-4.18 (2H, m, ArC(4)-OCH_2), 4.18-4.41 (2H, m, ArC(1)-OCH_2), 5.78 (1H, s, ArC(2)-O*H*), 6.71 (1H, s, ArC(3)*H*), 6.92 (1H, s, ArC(6)*H*), 7.29-7.34 (1H, m, ArC(5)-PhC(4)*H*), 7.38-7.45 (2H, m, ArC(5)-PhC(3,5)*H*), 7.50-7.54 (2H, m, ArC(5)-PhC(2,6)*H*); **^{13}C{1H} NMR** (126 MHz, CDCl$_3$) δ$_C$: 23.8 (CH_2), 24.1 (CH_2), 27.3 (CH_2), 27.7 (2×CH_2), 27.8 (CH_2), 28.0 (CH_2), 28.0 (CH_2), 28.1 (CH_2), 28.3 (CH_2), 29.4 (CH_2), 68.3 (ArC(4)-OCH_2), 69.5 (ArC(1)-OCH_2), 102.3 (Ar*C*(3)H), 116.6 (Ar*C*(6)H), 123.3 (Ar*C*(5)), 126.3 (ArC(5)-Ph*C*(4)H), 127.9 (ArC(5)-Ph*C*(3,5)H), 129.6 (ArC(5)-Ph*C*(2,6)H), 138.8 (ArC(5)-Ph*C*(1)), 138.9 (Ar*C*(1)), 146.6 (Ar*C*(2)), 150.1 (Ar*C*(4)); HRMS (APCI$^+$) $C_{25}H_{34}O_3$ ([M+Na]$^+$) requires 405.24002, found 405.2397 (–0.88 ppm).

Synthesis of 1^5-bromo-2,17-dioxa-1(1,4)-benzenacyclohexadecaphan-1^2-ol S61

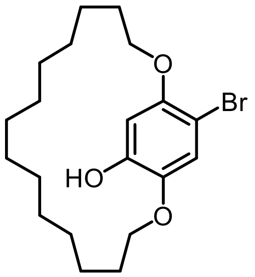

The title compound was prepared according to *General Procedure* C from **S15** (0.41 g, 1.00 mmol) and *m*-CPBA (0.33 g, 1.30 mmol, 70% wt) in CH_2Cl_2 (4 ml) were stirring for 8 h at room temperature to afford **S61** as a white solid (0.32 g, 80%); mp 48–50 °C (EtOAc/Hexane); ν$_{max}$ (film)/cm^{-1} 3503 (O–H), 2922 (C–H), 2851 (C–H), 1497 (C=C), 1300 (C=C), 1165 (C–O), 733 (C–Br); **^{1}H NMR** (500 MHz, CDCl$_3$) δ$_H$: 1.00-1.31 (16H, m, 8 × CH_2), 1.32-1.42 (2H, m, CH_2), 1.42-1.52 (2H, m, CH_2), 1.66-1.78 (4H, m, 2 × CH_2), 4.17 (2H, t, *J* 5.8, ArC(4)-OCH_2), 4.20 (2H, t, *J* 5.9, ArC(1)-OCH_2), 5.71 (1H, s, ArC(2)-O*H*), 6.64 (1H, s, ArC(3)*H*), 7.07 (1H, s, ArC(6)*H*); **^{13}C{1H} NMR** (126 MHz, CDCl$_3$) δ$_C$: 23.8 (CH_2), 23.8 (CH_2), 26.8 (CH_2), 27.1 (CH_2), 27.3 (CH_2), 27.4 (CH_2), 27.8 (CH_2), 27.9 (CH_2), 28.1 (CH_2), 28.3 (CH_2), 28.8 (CH_2), 28.9 (CH_2), 68.7 (ArC(4)-OCH_2), 69.0 (ArC(1)-OCH_2), 101.0 (Ar*C*(5)), 102.2 (ArC(3)H), 117.7 (Ar*C*(6)H), 139.2 (ArC(1)), 146.3 (Ar*C*(2)), 149.6 (ArC(4)); HRMS (APCI$^+$) $C_{20}H_{31}O_3Br$ ([M+Na]$^+$) requires 421.13488, found 421.1348 (–0.17 ppm).

Synthesis of 1^5-phenyl-2,17-dioxa-1(1,4)-benzenacyclohexadecaphan-1^2-ol S62

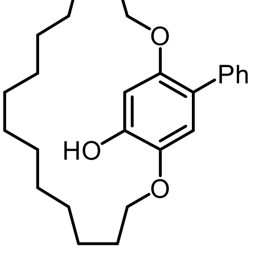

The title compound was prepared according to *General Procedure* C from **S34** (0.41 g, 1.00 mmol) and *m*-CPBA (0.33 g, 1.30 mmol, 70% wt) in CH_2Cl_2 (4 ml) were stirring for 8 h at room temperature to afford **S62** as a colorless oil (0.33 g, 83%); ν_{max} (film)/cm^{-1} 3530 (O–H), 2922 (C–H), 2853 (C–H), 1489 (C=C), 1294 (C=C), 1175 (C–O); **^{1}H NMR** (500 MHz, $CDCl_3$) δ_H: 1.02-1.23 (14H, m, 7 × C*H*$_2$), 1.28 (4H, p, *J* 6.8, 2 × C*H*$_2$), 1.40 (2H, p, *J* 7.1, C*H*$_2$), 1.64 (2H, p, *J* 6.8, C*H*$_2$), 1.74 (2H, p, *J* 7.3, *J* 6.9, C*H*$_2$), 4.03 (2H, t, *J* 5.8, ArC(4)-OC*H*$_2$), 4.25 (2H, t, *J* 5.9, ArC(1)-OC*H*$_2$), 5.75 (1H, s, ArC(2)-O*H*), 6.69 (1H, s, ArC(3)*H*), 6.90 (1H, s, ArC(6)*H*), 7.29-7.33 (1H, m, ArC(5)-PhC(4)*H*), 7.36-7.47 (2H, m, ArC(5)-PhC(3,5)*H*), 7.47-7.57 (2H, m, ArC(5)-PhC(2,6)*H*); **^{13}C{1H} NMR** (126 MHz, $CDCl_3$) δ_C: 23.8 (CH_2), 24.0 (CH_2), 26.6 (CH_2), 27.4 (2×CH_2), 27.5 (CH_2), 27.9 (CH_2), 28.0 (CH_2), 28.1 (CH_2), 28.5 (CH_2), 28.7 (CH_2), 29.1 (CH_2), 67.8 (ArC(4)-OCH_2), 69.0 (ArC(1)-OCH_2), 101.6 (Ar*C*(3)H), 116.1 (Ar*C*(6)H), 122.8 (Ar*C*(5)), 126.3 (ArC(5)-Ph*C*(4)H), 127.8 (ArC(5)-Ph*C*(3,5)H), 129.6 (ArC(5)-Ph*C*(2,6)H), 138.6 (ArC(5)-Ph*C*(1)), 138.9 (Ar*C*(1)), 146.4 (Ar*C*(2)), 150.2 (Ar*C*(4)); HRMS (APCI$^+$) $C_{26}H_{36}O_3$ ($[M+Na]^+$) requires 419.25567, found 419.2556 (–0.25 ppm).

Synthesis of 1^2-bromo-1^5-hydroxy-2,17-dioxa-5,14-diaza-1(1,4)-benzenacycloheptadecaphane-4,15-dione S63

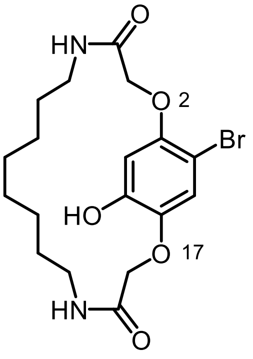

The title compound was prepared according to *General Procedure* C from **S18** (0.31 g, 0.70 mmol) and *m*-CPBA (0.23 g, 0.91 mmol, 70% wt) in CH_2Cl_2 (3 ml) were stirring for 8 h at room temperature to afford **S63** as a white solid (0.19 g, 63%); mp 162–166 °C (EtOAc/Hexane); ν_{max} (film)/cm^{-1} 3370 (N–H), 3242 (O–H), 2928 (C–H), 2855 (C–H), 1651 (C=O), 1549 (N–H), 1505 (N–H), 1192 (C–O), 1038 (C–O); **^{1}H NMR** (500 MHz, DMSO-d^6) δ_H: 0.94-1.19 (8H, m, 4 × C*H*$_2$), 1.29-1.50 (4H, m, 2 × C*H*$_2$), 3.13 (4H, s, C(6,13)*H*$_2$), 4.51 (4H, s, ArC(1,4)-OC*H*$_2$), 6.53 (1H, s, ArC(3)*H*), 7.33 (1H, s, ArC(6)*H*), 7.66 (1H, t, *J* 5.8, C(4)ON*H*), 7.96 (1H, t, *J* 5.7, C(15)ON*H*), 10.01 (1H, s, ArC(2)-O*H*); **^{13}C{1H} NMR** (126 MHz, DMSO-d^6) δ_C: 26.3 (2 × CH_2), 29.2 (CH_2), 29.4 (CH_2), 29.7 (CH_2), 29.7 (CH_2), 38.7 (CH_2), 38.8 (CH_2), 69.0 (ArC(1)-OCH_2), 71.4 (ArC(4)-OCH_2), 99.3 (Ar*C*(5)), 102.9 (ArC(3)H), 122.2 (ArC(6)H), 141.6 (ArC(2)), 148.2 (ArC(1)), 150.4 (Ar*C*(4)), 167.9 (*C*(4)ONH), 169.0 (*C*(15)ONH); HRMS (APCI$^+$) $C_{18}H_{24}O_5N_2Br$ ($[M+Na]^+$) requires 427.08741, found 427.0870 (–0.91 ppm).

Synthesis of 1^2-hydroxy-1^5-phenyl-2,17-dioxa-5,14-diaza-1(1,4)-benzenacycloheptadecaphane-4,15-dione S64

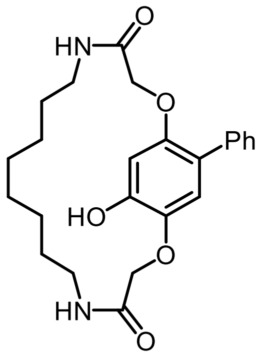

The title compound was prepared according to *General Procedure* C from **S35** (0.31 g, 0.70 mmol) and *m*-CPBA (0.23 g, 0.91 mmol, 70% wt) in CH_2Cl_2 (3 ml) were stirring for 8 h at room temperature to afford **S64** as an off-white solid (0.17 g, 57%); mp 146–150 °C (EtOAc/Hexane); ν_{max} (film)/cm^{-1} 3287 (N–H), 3245 (O–H), 2924 (C–H), 2853 (C–H), 1638 (C=O), 1516 (C=C), 1489 (C=C), 1184 (C–O), 1071 (C–O); **^{1}H NMR** (500 MHz, $CDCl_3$) δ_H: 1.09-1.29 (8H, m, 4 × C*H*$_2$), 1.36-1.69 (4H, m, 2 × C*H*$_2$), 2.70-3.92 (4H, m, 2 × C(6,13)*H*$_2$), 4.32-4.87 (4H, m, ArC(1,4)-OC*H*$_2$), 6.44 (1H, t, *J* 6.0, C(4)ON*H*), 6.68 (1H, s, ArC(3)*H*), 6.99 (1H, s, ArC(6)*H*), 7.32-7.40 (1H, m, ArC(5)-PhC(4)*H*), 7.40-7.50 (4H, m, 4 × ArC(5)-PhC*H*), 7.52 (1H, t, *J* 5.8, C(15)ON*H*), 9.29 (1H, s, ArC(2)-O*H*); **^{13}C{1H} NMR** (126 MHz, $CDCl_3$) δ_C: 26.4 (*C*H$_2$), 26.5 (*C*H$_2$), 28.9 (*C*H$_2$), 29.1 (3 × *C*H$_2$), 29.5 (*C*H$_2$), 29.6 (*C*H$_2$), 69.1 (ArC(1)-O*C*H$_2$), 71.2 (ArC(4)-O*C*H$_2$), 101.6 (Ar*C*(3)H), 119.1 (Ar*C*(6)H), 122.7 (Ar*C*(5)), 127.2 (ArC(5)-Ph*C*(4)H), 128.3 (ArC(5)-Ph*C*(3,5)H), 129.5 (ArC(5)-Ph*C*(2,6)H), 137.1 (ArC(5)-Ph*C*(1)), 141.5 (Ar*C*(2)), 147.3 (Ar*C*(1)), 150.2 (Ar*C*(4)), 169.4 (*C*(4)ONH), 170.0 (*C*(15)ONH); HRMS (APCI$^+$) $C_{24}H_{30}O_5N_2Na$ ([M+Na]$^+$) requires 449.20469, found 449.2042 (–1.10 ppm).

Synthesis of 1^2-hydroxy-1^5-phenyl-2,8,10,16-tetraoxa-5,13-diaza-1(1,2),9(1,4)-dibenzenacyclo-hexadecaphane-4,14-dione S65

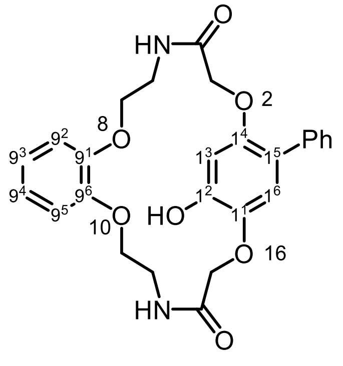

The title compound was prepared according to *General Procedure* C from **S19** (0.34 g, 0.70 mmol) and *m*-CPBA (0.23 g, 0.91 mmol, 70% wt) in CH_2Cl_2 (3 ml) were stirring for 8 h at room temperature to afford **S65** as a white solid (0.20 g, 60%); mp 163–166 °C (EtOAc/Hexane); ν_{max} (film)/cm^{-1} 3383 (N–H), 3064 (O–H), 2918 (C–H), 1674 (C=O), 1663 (C=O), 1645 (C=O), 1524 (C=C), 1506 (C=C), 1242 (C–O), 1211 (N–C), 1061 (C–O); **^{1}H NMR** (500 MHz, DMSO-d^6) δ_H: 3.35 (2H, s, C(6)H_2), 3.51 (2H, s, C(12)H_2), 4.06 (4H, t, *J* 4.7, C(7,11)H_2), 4.46 (2H, s, ArC(1^4)-OCH_2), 4.58 (2H, s, ArC(1^1)-OCH_2), 6.59 (1H, s, ArC(1^3)*H*), 6.86-7.00 (4H, m, 4 × ArC(9^{2,3,4,5})*H*), 7.04 (1H, s, ArC(1^6)*H*), 7.16-7.26 (3H, m, ArC(1^5)-PhC(3,4,5)*H*), 7.46 (1H, t, *J* 5.5, C(4)ON*H*), 7.53 (2H, m, ArC(1^5)-PhC(2,6)*H*), 8.34 (1H, t, *J* 5.4, C(14)ON*H*), 10.02 (1H, s, ArC(2)-O*H*); **^{13}C{1H} NMR** (126 MHz, DMSO-d^6) δ_C: 38.9 (*C*(6)H_2), 39.4 (*C*(12)H_2), 66.9 (*C*(7)H_2), 67.1 (*C*(11)H_2), 69.2 (ArC(1^4)-OCH_2), 71.4 (ArC(1^1)-OCH_2), 103.2 (Ar*C*(1^3)H), 113.5 (Ar*C*(9^2)H), 113.9 (Ar*C*(9^5)H), 119.8 (Ar*C*(1^6)H), 121.3 (Ar*C*(9^3)H), 121.3 (Ar*C*(9^4)H), 122.0 (Ar*C*(1^5)), 126.9 (ArC(1^5)-Ph*C*(4)H), 128.2 (ArC(1^5)-Ph*C*(3,5)H), 130.2 (ArC(1^5)-Ph*C*(2,6)H), 137.9 (ArC(1^5)-Ph*C*(1)), 141.4 (Ar*C*(1^1)), 148.1 (Ar*C*(1^2)), 148.3 (Ar*C*(9^6)), 148.6 (Ar*C*(9^1)), 150.4 (Ar*C*(1^4)), 169.0 (*C*(4)ONH), 169.8 (*C*(14)ONH); HRMS (APCI$^+$) $C_{26}H_{26}N_2O_7Na$ ([M+Na]$^+$) requires 501.16322, found 501.1634 (+0.36 ppm).

Synthesis of 1^5-phenyl-2,18-dioxa-1(1,4)-benzenacyclohexadecaphan-1^2-ol S66

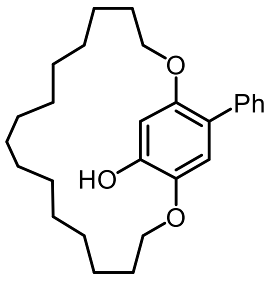

The title compound was prepared according to *General Procedure* C from **S36** (0.42 g, 1.00 mmol) and *m*-CPBA (0.33 g, 1.30 mmol, 70% wt) in CH_2Cl_2 (4 ml) were stirring for 8 h at room temperature to afford **S66** as a colorless oil (0.35 g, 85%); ν_{max} (film)/cm^{-1} 3528 (O–H), 2922 (C–H), 2853 (C–H), 1489 (C=C), 1294 (C=C), 1173 (C–O); **^{1}H NMR** (500 MHz, $CDCl_3$) δ_H: 1.06-1.25 (16H, m, 8 × CH_2), 1.30 (4H, p, *J* 6.8, *J* 6.7, 2 × CH_2), 1.42 (2H, p, *J* 7.0, CH_2), 1.63 (2H, p, *J* 6.5, CH_2), 1.73 (2H, dt, *J* 7.4, *J* 6.0, CH_2), 4.03 (2H, t, *J* 6.0, ArC(4)-OCH_2), 4.25 (2H, t, *J* 5.9, ArC(1)-OCH_2), 5.76 (1H, s, ArC(2)-O*H*), 6.70 (1H, s, ArC(3)*H*), 6.91 (1H, s, ArC(6)*H*), 7.29-7.34 (1H, m, ArC(5)-PhC(4)*H*), 7.37-7.43 (2H, m, ArC(5)-PhC(3,5)*H*), 7.47-7.56 (2H, m, ArC(5)-PhC(2,6)*H*); **^{13}C{1H} NMR** (126 MHz, $CDCl_3$) δ_C: 24.3 (CH_2), 24.6 (CH_2), 27.2 (CH_2), 27.7 (CH_2), 27.8 (CH_2), 28.1 (CH_2), 28.1 (CH_2), 28.3 (2 × CH_2), 28.3 (CH_2), 28.6 (CH_2), 29.0 (CH_2), 29.3 (CH_2), 68.3 (ArC(4)-OCH_2), 69.4 (ArC(1)-OCH_2), 101.8 (Ar*C*(3)H), 116.0 (Ar*C*(6)H), 123.0 (Ar*C*(5)), 126.3 (ArC(5)-Ph*C*(4)H), 127.8 (ArC(5)-Ph*C*(3,5)H), 129.6 (ArC(5)-

PhC(2,6)H), 138.9 (ArC(5)-PhC(1)), 139.0 (ArC(1)), 146.5 (ArC(2)), 150.3 (ArC(4)); HRMS (APCI$^+$) $C_{27}H_{38}O_3$ ([M+Na]$^+$) requires 433.27132, found 433.2707 (–1.52 ppm).

Synthesis of 1^5-phenyl-2,19-dioxa-1(1,4)-benzenacyclohexadecaphan-1^2-ol S67

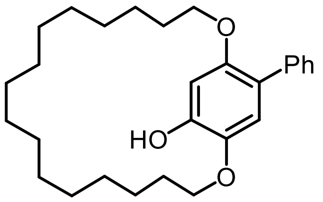

The title compound was prepared according to *General Procedure* C from **S37** (0.44 g, 1.00 mmol) and *m*-CPBA (0.33 g, 1.30 mmol, 70% wt) in CH_2Cl_2 (4 ml) were stirring for 8 h at room temperature to afford **S67** as a colorless oil (0.34 g, 80%); ν_{max} (film)/cm^{-1} 3528 (O–H), 2924 (C–H), 2853 (C–H), 1489 (C=C), 1296 (C=C), 1177 (C–O); **^{1}H NMR** (500 MHz, $CDCl_3$) δ_H: 1.11-1.27 (18H, m, 9 × CH_2), 1.32 (4H, p, *J* 7.0, *J* 6.8, 2 × CH_2), 1.45 (2H, p, *J* 7.1, *J* 7.0, CH_2), 1.66 (2H, p, *J* 6.4, CH_2), 1.77 (2H, p, *J* 6.5, CH_2), 3.99 (2H, t, *J* 6.1, ArC(4)-OCH_2), 4.20 (2H, t, *J* 6.0, ArC(1)-OCH_2), 5.73 (1H, s, ArC(2)-O*H*), 6.69 (1H, s, ArC(3)*H*), 6.89 (1H, s, ArC(6)*H*), 7.29-7.33 (1H, m, ArC(5)-PhC(4)*H*), 7.36-7.43 (2H, m, ArC(5)-PhC(3,5)*H*), 7.49-7.58 (2H, m, ArC(5)-PhC(2,6)*H*); **^{13}C{1H} NMR** (126 MHz, $CDCl_3$) δ_C: 24.1 (CH$_2$), 24.4 (CH$_2$), 26.8 (CH$_2$), 27.4 (CH$_2$), 27.7 (CH$_2$), 27.7 (CH$_2$), 27.9 (CH$_2$), 28.1 (CH$_2$), 28.2 (CH$_2$), 28.4 (CH$_2$), 28.5 (CH$_2$), 28.9 (CH$_2$), 29.1 (CH$_2$), 29.3 (CH$_2$), 68.0 (ArC(4)-OCH$_2$), 69.0 (ArC(1)-OCH$_2$), 101.4 (ArC(3)H), 115.5 (ArC(6)H), 122.6 (ArC(5)), 126.3 (ArC(5)-PhC(4)H), 127.8 (ArC(5)-PhC(3,5)H), 129.6 (ArC(5)-PhC(2,6)H), 138.9 (ArC(5)-PhC(1)), 139.0 (ArC(1)), 146.3 (ArC(2)), 150.3 (ArC(4)); HRMS (APCI$^+$) $C_{28}H_{40}O_3$ ([M+Na]$^+$) requires 447.28697, found 447.2866 (–0.83 ppm).

Synthesis of 1^5-butyl-2,15-dioxa-1(1,4)-benzenacyclotridecaphane-1^2-carbaldehyde S68

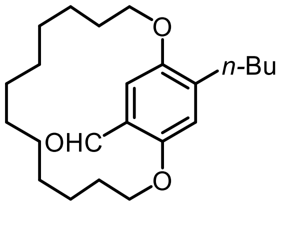

In a 25 ml flask, **S12** (0.34 g, 0.9 mmol) and Pd[P(*t*-Bu)$_3$]$_2$ (2.5 mol%, 0.023 mmol, 11.5 mg) were added to 3.0 ml of deionized water, at room temperature and in air. The mixture was vigorously stirred for 10 min. Then a solution of *n*-BuZnCl (2.7 mmol) was rapidly spread over the mixture under air and with vigorous stirring at room temperature to generate an emulsion. After 5 min, the mixture was acidified with HCl (1 M) and the mixture was diluted with water (20 ml) and extracted with CH_2Cl_2 (2 × 30 ml). The combined organic layers

were washed with water and brine, dried over Na_2SO_4, filtered, and concentrated in *vacuo*. The residue was purified by column chromatography (pure toluene) to afford the cross-coupling product **S68** (0.28 g, 87%); ν_{max} (film)/cm^{-1} 2924 (C–H), 2855 (C–H), 1678 (C=O), 1487 (C=C), 1418 (C=C), 1192 (C–O); **^{1}H NMR** (500 MHz, $CDCl_3$) δ_H: 0.78-0.94 (8H, m, 4 × CH_2), 0.96 (3H, t, *J* 7.3, Ar(5)-$CH_2CH_2CH_2CH_3$), 1.02-1.23 (4H, 2 × CH_2), 1.24-1.35 (4H, m, 2 × CH_2), 1.41 (2H, h, *J* 7.4, Ar(5)-$CH_2CH_2CH_2CH_3$), 1.49-1.69 (4H, m, CH_2, Ar(5)-$CH_2CH_2CH_2CH_3$), 1.76-1.89 (2H, m, CH_2), 2.62 (1H, ddd, *J* 13.7, *J* 9.1, *J* 6.3, Ar(5)-$CH^AH^BC_3H_7$), 2.71 (1H, ddd, *J* 13.6, *J* 9.1, *J* 6.4, Ar(5)-$CH^AH^BC_3H_7$), 4.14 (1H, ddd, *J* 12.1, *J* 8.9, *J* 3.8, ArC(4)-OCH^AH^B), 4.21 (1H, ddd, *J* 12.1, *J* 8.6, *J* 3.8, ArC(2)-OCH^AH^B), 4.34-4.51 (2H, m, ArC(4)-OCH^AH^B, ArC(2)-OCH^AH^B), 6.86 (1H, s, ArC(3)*H*), 7.32 (1H, s, ArC(6)*H*), 10.44 (1H, s, C*H*O); **^{13}C{1H} NMR** (126 MHz, $CDCl_3$) δ_C: 14.0 (Ar(5)-$CH_2CH_2CH_2CH_3$), 22.7 (Ar(5)-$CH_2CH_2CH_2CH_3$), 23.9 (CH_2), 24.2 (CH_2), 26.7 (CH_2), 27.2 (CH_2), 27.5 (CH_2), 27.7 (CH_2), 27.9 (CH_2), 28.0 (CH_2), 28.5 (CH_2), 28.5 (CH_2), 30.6 (Ar(5)-$CH_2CH_2CH_2CH_3$), 31.6 (Ar(5)-$CH_2CH_2CH_2CH_3$), 67.5 (ArC(4)-OCH_2), 68.9 (ArC(1)-OCH_2), 110.6 (Ar*C*(3)H), 117.4 (Ar*C*(6)H), 124.2 (Ar*C*(2)), 141.7 (ArC(5)), 150.0 (Ar*C*(4)), 155.2 (Ar*C*(1)), 189.5 (*C*=O); HRMS (APCI$^+$) $C_{23}H_{36}O_3$ ([M+Na]$^+$) requires 383.25567, found 383.2552 (–1.11 ppm).

Synthesis of 1^5-butyl-2,15-dioxa-1(1,4)-benzenacyclopentadecaphan-1^2-ol S69

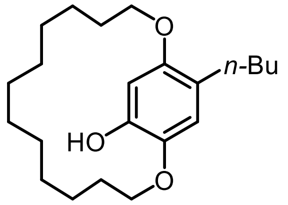

The title compound was prepared according to *General Procedure* C from **S68** (0.18 g, 0.51 mmol) and *m*-CPBA (0.12 g, 0.68 mmol, 70% wt) in CH_2Cl_2 (4 ml) were stirring for 8 h at room temperature to afford **S69** as a white solid (0.16 g, 89%); ν_{max} (film)/cm^{-1} 3404 (O–H), 2924 (C–H), 2853 (C–H), 1505 (C=C), 1433 (C=C), 1213 (C–O), 1161 (C–O); **^{1}H NMR** (500 MHz, $CDCl_3$) δ_H: 0.90-1.08 (11H, m, 4 × CH_2, Ar(5)-$CH_2CH_2CH_2CH_3$), 1.09-1.35 (7H, m, 3 × CH_2, CH^AH^B), 1.36-1.44 (3H, m, CH^AH^B, Ar(5)-$CH_2CH_2CH_2CH_3$), 1.46-1.64 (4H, m, CH_2, Ar(5)-$CH_2CH_2CH_2CH_3$), 1.70-1.90 (2H, m, CH_2), 2.51 (1H, ddd, *J* 13.8, *J* 9.0, *J* 6.2, Ar(5)-$CH^AH^BC_3H_7$), 2.61 (1H, ddd, *J* 13.7, *J* 9.1, *J* 6.4, Ar(5)-$CH^AH^BC_3H_7$), 4.09 (1H, ddd, *J* 12.1, *J* 8.3, *J* 4.3, ArC(1)-OCH^AH^B), 4.17 (1H, ddd, *J* 11.8, *J* 8.2, *J* 4.6, ArC(4)-OCH^AH^B), 4.22-4.35 (2H, m, ArC(1)-OCH^AH^B, ArC(4)-OCH^AH^B), 5.66 (1H, s, ArC(2)-O*H*), 6.57 (1H, s, ArC(3)*H*), 6.73 (1H, s, ArC(6)*H*); **^{13}C{1H} NMR** (126 MHz, $CDCl_3$) δ_C: 14.1 (Ar(5)-$CH_2CH_2CH_2CH_3$), 22.6 (Ar(5)-$CH_2CH_2CH_2CH_3$), 24.0 (CH_2), 24.0 (CH_2), 27.0 (CH_2), 27.3 (CH_2), 27.5 (CH_2), 27.7 (CH_2), 27.7 (CH_2), 27.8 (CH_2), 28.4 (CH_2), 28.4 (CH_2), 29.4 (Ar(5)-$CH_2CH_2CH_2CH_3$), 32.5 (Ar(5)-$CH_2CH_2CH_2CH_3$), 67.7 (ArC(4)-

OCH_2), 69.6 (ArC(1)-OCH_2), 101.4 (Ar*C*(3)H), 116.9 (Ar*C*(6)H), 123.3 (Ar*C*(4)), 137.7 (ArC(1)), 145.3 (Ar*C*(2)), 150.4 (Ar*C*(4)); HRMS (APCI$^+$) $C_{18}H_{27}O_3Br$ ([M+Na]$^+$) requires 371.25567, found 371.2552 (–1.03 ppm)

2.7 General Procedure E: Kinetic resolution of macrocycle phenol derivatives

Isobutyric anhydride (0.12 mmol, 0.6 equiv.) was added to a solution of macrocycle phenol derivatives (0.20 mmol, 1.0 equiv.), (*R*)-BTM (5 mol%) and quinaldine (0.12 mmol, 0.6 equiv.) in toluene (2 ml, 0.1 M), the resulting mixture was then stirred overnight at room temperature. The mixture was then concentrated under reduced pressure to give the crude residue, which was subsequently purified by column chromatography.

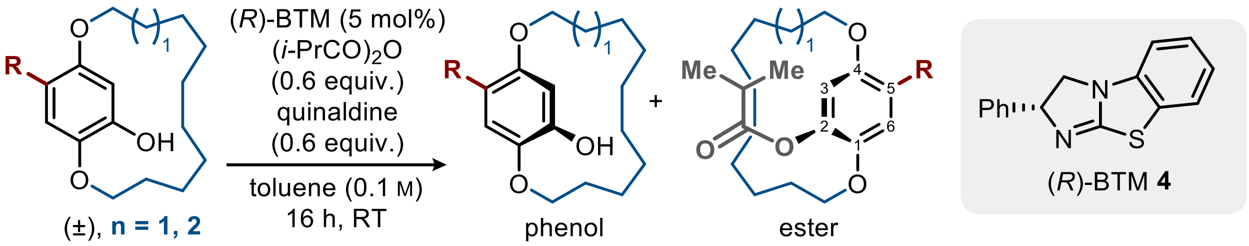

Acylative kinetic resolution of chiral paracyclophanols using $CHCl_3$ as reaction solvent

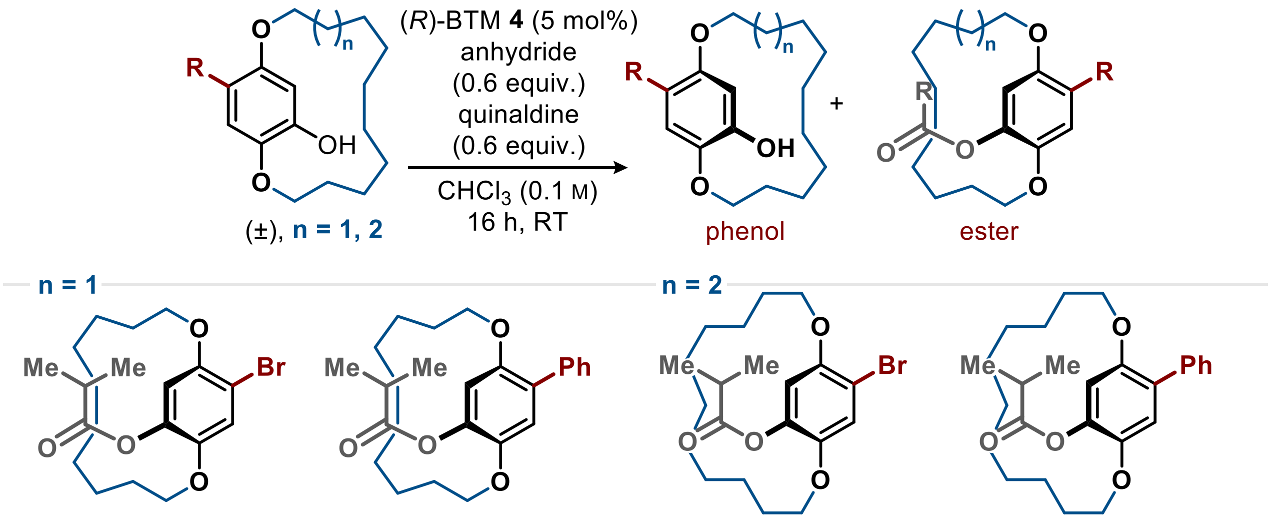

**2**[a,b], **c = 52, *s* = 16**	**5**[a,b], **c = 51, *s* = 12**	**8**[a,b], **c = 51, *s* = 13**	**9**[a,b], **c = 43, *s* = 13**
**phenol:** 90:10 er, 46%	**phenol:** 86:14 er, 39%	**phenol:** 87:13 er, 42%	**phenol:** 79:21 er, 45%
**ester:** 87:13 er, 45%	**ester:** 85:15 er, 46%	**ester:** 86:14 er, 47%	**ester:** 88:12 er, 39%

a) Conversion (c) and selectivity factor (*s*) calculated using the enantiomeric ratios of recovered alcohol and ester as measured by HPLC analysis on a chiral stationary phase. *s* values rounded according to estimated errors; b) Isobutyric anhydride used.

2.8 General Procedure F: Dynamic kinetic resolution of macrocycle phenol derivatives

Isobutyric anhydride (0.10 mmol, 1.0 equiv.) was added to a solution of macrocycle phenol derivatives (0.10 mmol, 1.0 equiv.), (*R*)-BTM (5 mol%) and *i*-Pr_2NEt (0.10 mmol, 1.0 equiv.) in toluene (1 ml, 0.1 M), the resulting mixture was then stirred 24 h at room temperature. The mixture was then concentrated under reduced pressure to give the crude residue, which was subsequently purified by column chromatography.

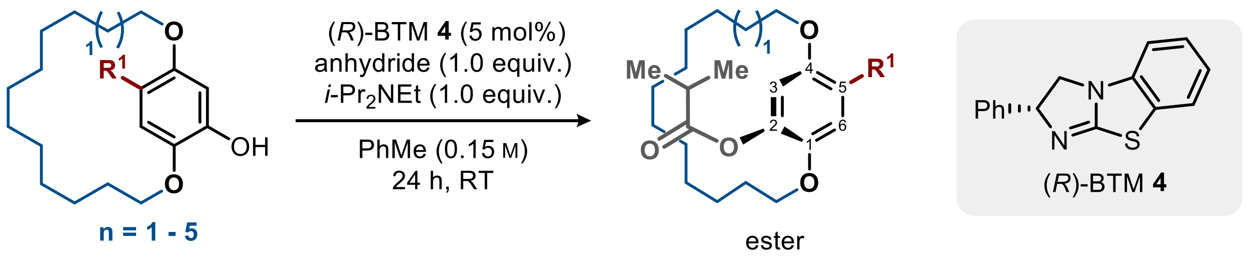

2.8.1 Macrocycle phenols products and macrocycle esters products

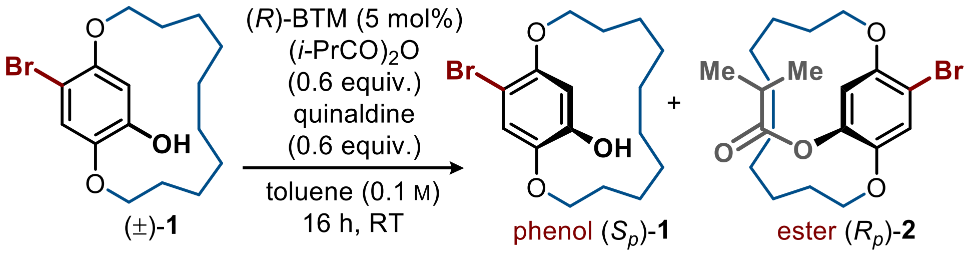

Following General Procedure E and purification of the residue by silica gel chromatography column using 10:1 petrol ether/EtOAc as eluent gave the product (*Sp*)-**1** (yield 38%, 93:7 er); $[\alpha]_D^{20}$ –6.7 (c 0.9 in $CHCl_3$); chiral HPLC analysis, ChiralPak OD-H (15% *i*-PrOH : hexane, flow rate 1 mLmin^{-1}, 254 nm, 30 °C), tR minor: 5.1 min, tR major: 6.9 min, 93:7 er; and product (*Rp*)-**2** as colourless oil (yield 47%, 95:5 er); $[\alpha]_D^{20}$ –10.2 (c 1.8 in $CHCl_3$); chiral HPLC analysis, ChiralPak ID-H (3% *i*-PrOH : hexane, flow rate 1 mLmin^{-1}, 254 nm, 30 °C), tR major: 8.8 min, tR minor: 25.4 min, 95:5 er; ν_{max} (film)/cm^{-1} 2970 (C–H), 2928 (C–H), 1759 (C=O), 1487 (C=C), 1134 (C–O), 1092 (C–O); **^{1}H NMR** (500 MHz, $CDCl_3$) δ_H: 0.72-0.85 (4H, m, 2 × C*H*$_2$), 0.90-1.02 (2H, m, C*H*$_2$), 1.06-1.28 (6H, m, 3 × C*H*$_2$), 1.31 (3H, d, *J* 6.9, CH(C*H*$_3$)^A(CH$_3$)^B), 1.34 (3H, d, *J* 7.0, CH(CH$_3$)^A(C*H*$_3$)^B), 1.49-1.62 (2H, m, C*H*$_2$), 1.63-1.81 (2H, m, C*H*$_2$), 2.83 (1H, hept, *J* 7.0, C*H*(CH$_3$)$_2$), 4.09-4.16 (1H, m, ArC(1)-OC*H*^{A}H^B), 4.21-4.25 (1H, m, ArC(1)-OCH^A*H*^{B}), 4.26-4.30 (1H, m, ArC(4)-OC*H*^{A}H^B), 4.30-4.37 (1H, m, ArC(4)-OCH^A*H*^{B}), 6.77 (1H, s, ArC(3)*H*), 7.26 (1H, s, ArC(6)*H*); **^{13}C{1H} NMR** (126 MHz, $CDCl_3$) δ_C: 18.9 (CH(CH$_3$)^A(CH$_3$)^B), 19.2 (CH(CH$_3$)^A(CH$_3$)^B), 23.5 (CH$_2$), 23.5 (CH$_2$), 27.1 (CH$_2$), 27.1 (CH$_2$), 27.3 (CH$_2$), 27.5 (CH$_2$), 27.6 (CH$_2$), 28.1 (CH$_2$), 34.0 (*C*H(CH$_3$)$_2$), 70.3 (ArC(4)-OCH$_2$), 70.4 (ArC(1)-OCH$_2$), 110.8 (Ar*C*(5)), 113.4 (Ar*C*(3)H), 123.4 (Ar*C*(6)H), 141.4 (ArC(2)), 145.1 (ArC(1)), 149.4 (ArC(4)), 174.6 (*C*=O); HRMS (APCI$^+$) $C_{20}H_{29}O_4Br$ ([M+Na]$^+$) requires 435.1141, found 435.1146 (+0.9 ppm).

(*Sp*)-1^5-bromo-2,13-dioxa-1(1,4)-benzenacyclotridecaphan-1^2-ol 1

<Chromatogram>

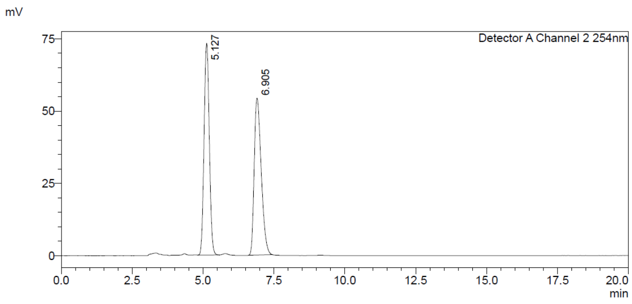

<Peak Table>

Detector A Channel 2 254nm

Peak#	Ret. Time	Area%
1	5.127	50.173
2	6.905	49.827
Total		100.000

<Chromatogram>

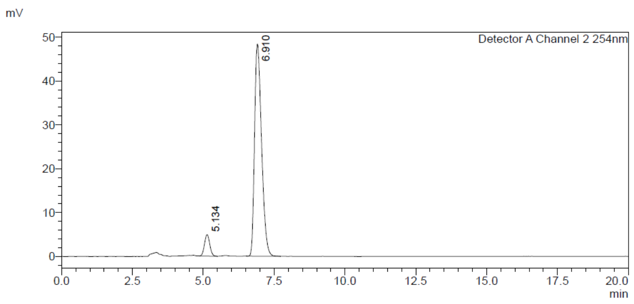

<Peak Table>

Detector A Channel 2 254nm

Peak#	Ret. Time	Area%
1	5.134	7.045
2	6.910	92.955
Total		100.000

(*Rp*)-1^5-bromo-2,13-dioxa-1(1,4)-benzenacyclotridecaphane-1^2-yl isobutyrate 2

<Chromatogram>

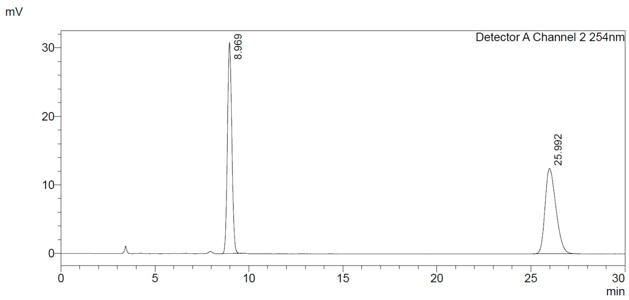

<Peak Table>

Detector A Channel 2 254nm

Peak#	Ret. Time	Area%
1	8.969	49.857
2	25.992	50.143
Total		100.000

<Chromatogram>

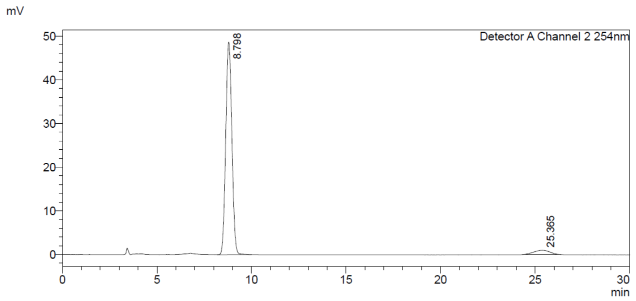

<Peak Table>

Detector A Channel 2 254nm

Peak#	Ret. Time	Area%
1	8.798	94.927
2	25.365	5.073
Total		100.000

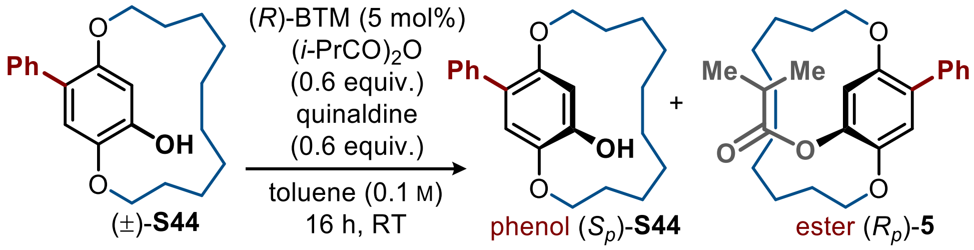

Following General Procedure E and purification of the residue by silica gel chromatography column using 10:1 petrol ether/EtOAc as eluent gave the product (*Sp*)-**S44** (yield 35%, 96:4 er); $[\alpha]_D^{20}$ +14.5 (c 1.1 in $CHCl_3$); chiral HPLC analysis, ChiralPak OD-H (5% *i*-PrOH : hexane, flow rate 1 mLmin^{-1}, 254 nm, 30 °C), tR minor: 7.4 min, tR major: 10.2 min, 96:4 er; and the product (*Rp*)-**5** as a colourless oil (yield 44%, 92:8 er); $[\alpha]_D^{20}$ –17.3 (c 2.2 in $CHCl_3$); chiral HPLC analysis, ChiralPak ID-H (3% *i*-PrOH : hexane, flow rate 1 mLmin^{-1}, 254 nm, 30 °C), tR major: 7.0 min, tR minor: 21.4 min, 92:8 er; ν_{max} (film)/cm^{-1} 2926 (C–H), 2855 (C–H), 1759 (C=O), 1487 (C=C), 1182 (C–O), 1124 (C–O); **^{1}H NMR** (500 MHz, $CDCl_3$) δ_H: 0.86-1.18 (10H, m, 5 × C*H*$_2$), 1.22-1.32 (3H, m, C*H*$_2$, C*H*^{A}H^B), 1.37 (3H, d, *J* 7.0, CH(C*H*$_3$)^A(CH$_3$)^B), 1.40 (3H, d, *J* 7.0, CH(CH$_3$)^A(C*H*$_3$)^B), 1.56-1.63 (1H, m,

CH^A*H*^{B}), 1.65-1.87 (2H, m, C*H*$_2$), 2.89 (1H, hept, *J* 7.0, C*H*(CH$_3$)$_2$), 3.88 (1H, ddd, *J* 12.3, *J* 8.6, *J* 3.9, ArC(4)-OC*H*^{A}H^B), 4.11 (1H, ddd, *J* 12.3, *J* 5.9, *J* 4.1, ArC(4)-OCH^A*H*^{B}), 4.19 (1H, ddd, *J* 12.1, *J* 8.1, *J* 3.9, ArC(1)-OC*H*^{A}H^B), 4.36 (1H, ddd, *J* 12.2, *J* 6.4, *J* 3.9, ArC(1)-OCH^A*H*^{B}), 6.87 (1H, s, ArC(3)*H*), 7.09 (1H, s, ArC(6)*H*), 7.35 (1H, t, *J* 7.4, ArC(5)-PhC(4)*H*), 7.44 (2H, t, *J* 7.6, ArC(5)-PhC(3,5)*H*), 7.56 (2H, d, *J* 7.9, ArC(5)-PhC(2,6)*H*); **^{13}C{1H} NMR** (126 MHz, CDCl$_3$) δ_C: 18.9 (CH(*C*H$_3$)^A(CH$_3$)^B), 19.2 (CH(CH$_3$)^A(*C*H$_3$)^B), 23.7 (*C*H$_2$), 24.0 (*C*H$_2$), 27.1 (2 × *C*H$_2$), 27.4 (*C*H$_2$), 27.5 (*C*H$_2$), 27.7 (*C*H$_2$), 28.1 (*C*H$_2$), 34.1 (*C*H(CH$_3$)$_2$), 70.0 (ArC(4)-O*C*H$_2$), 70.1 (ArC(1)-O*C*H$_2$), 114.4 (Ar*C*(3)H), 121.1 (Ar*C*(6)H), 127.1 (ArC(5)-Ph*C*(4)H), 128.2 (ArC(5)-Ph*C*(3,5)H), 129.4 (ArC(5)-Ph*C*(2,6)H), 131.1 (Ar*C*(5)), 138.2 (ArC(5)-Ph*C*(1)), 141.2 (Ar*C*(2)), 144.6 (Ar*C*(1)), 150.1 (Ar*C*(4)), 174.8 (*C*=O); HRMS (APCI$^+$) $C_{26}H_{34}O_4$ ([M+Na]$^+$) requires 433.23493, found 433.2347 (–0.63 ppm).

(*Sp*)-1^5-phenyl-2,13-dioxa-1(1,4)-benzenacyclotridecaphan-1^2-ol S44

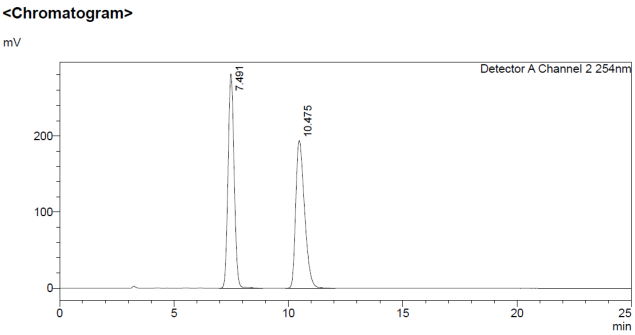

<Peak Table>

Detector A Channel 2 254nm

Peak#	Ret. Time	Area%
1	7.491	50.060
2	10.475	49.940
Total		100.000

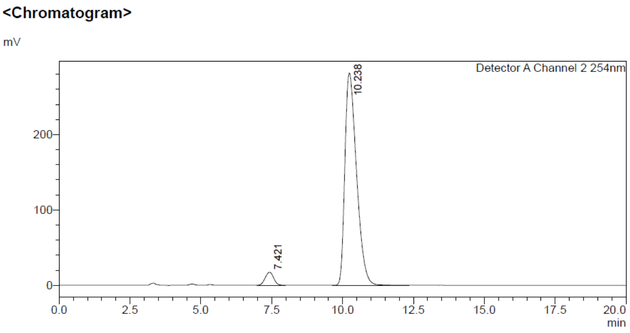

<Peak Table>

Detector A Channel 2 254nm

Peak#	Ret. Time	Area%
1	7.421	4.274
2	10.238	95.726
Total		100.000

(*Rp*)-1^5-phenyl-2,13-dioxa-1(1,4)-benzenacyclotridecaphane-1^2-yl isobutyrate 5

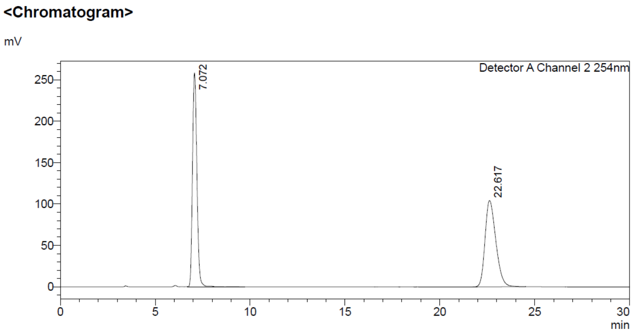

<Peak Table>

Detector A Channel 2 254nm

Peak#	Ret. Time	Area%
1	7.072	50.319
2	22.617	49.681
Total		100.000

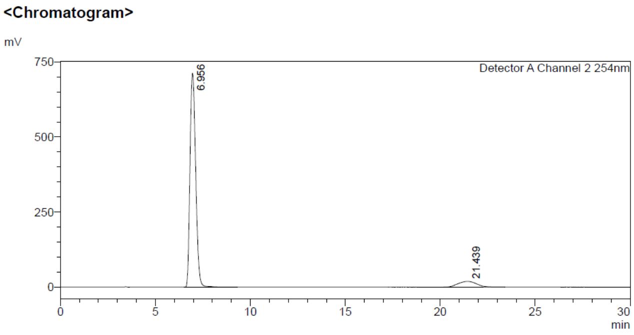

<Peak Table>

Detector A Channel 2 254nm

Peak#	Ret. Time	Area%
1	6.956	91.971
2	21.439	8.029
Total		100.000

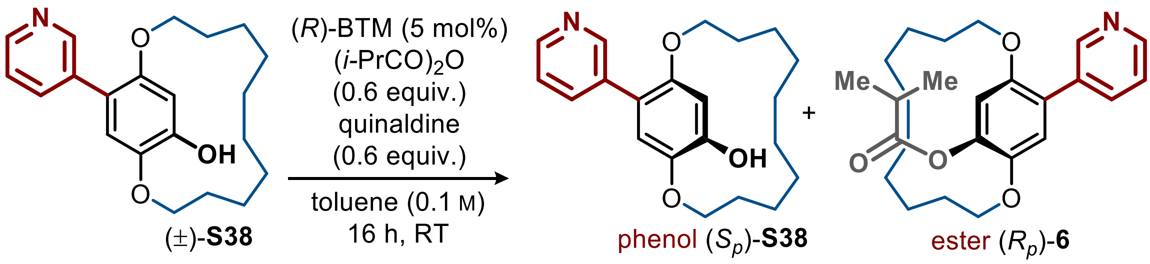

Following General Procedure E and purification of the residue by silica gel chromatography column using 3:1 petrol ether/EtOAc as eluent gave the product (*Sp*)-**S38** (yield 39%, >99:1 er); $[\alpha]_D^{20}$ –12.4 (c 0.9 in $CHCl_3$); chiral HPLC analysis, ChiralPak OD-H (15% *i*-PrOH : hexane, flow rate 1 $mLmin^{-1}$, 254 nm, 30 °C), tR minor: 6.7 min, tR major: 8.8 min, >99:1 er; and the product (*Rp*)-**6** as a colourless oil (yield 53%, 88:12 er); $[\alpha]_D^{20}$ –15.3 (c 1.0 in $CHCl_3$); chiral HPLC analysis, ChiralPak IB-H (5% *i*-PrOH : hexane, flow rate 1 $mLmin^{-1}$, 270 nm, 30 °C), tR major: 10.7 min, tR minor: 12.4 min, 88:12 er; ν_{max} (film)/cm^{-1} 2926 (C–H), 1759 (C=O), 1504 (C=C), 1182 (C–O), 1128 (C–O); **^{1}H NMR** (500 MHz, $CDCl_3$) δ_H: 0.80-1.18 (10H, m, 5 × C*H*$_2$), 1.22-1.31 (3H, m, C*H*$_2$, CH^AH^B), 1.36 (3H, d, *J* 7.0, CH(CH_3)^A(CH_3)^B), 1.39 (3H, d, *J* 7.1, CH(CH_3)^A(CH_3)^B), 1.55-1.85 (3H, m, C*H*$_2$, CH^AH^B), 2.88 (1H, hept, *J* 7.0, C*H*(CH_3)$_2$), 3.96 (1H, ddd, *J* 12.4, *J* 8.7, *J* 3.8, ArC(4)-OCH^AH^B), 4.16-4.24 (2H, m, ArC(1)-OCH^AH^B, ArC(4)-OCH^AH^B), 4.35 (1H, ddd, *J* 12.1, *J* 6.5, *J* 3.9, ArC(1)-OCH^AH^B), 6.88 (1H, s, ArC(3)*H*), 7.10 (1H, s, ArC(6)*H*), 7.36 (1H, ddd, *J* 7.9, *J* 4.8, *J* 0.8, ArC(5)-ArC(4)*H*), 7.91 (1H, dt, *J* 7.9, *J* 2.3, *J* 1.7, ArC(5)-ArC(5)*H*), 8.59 (1H, dd, *J* 4.9, *J* 1.7, ArC(5)-ArC(6)*H*), 8.78 (1H, d, *J* 1.8, ArC(5)-ArC(2)*H*); **^{13}C{1H} NMR** (126 MHz, $CDCl_3$) δ_C: 18.9 (CH(CH_3)^A(CH_3)^B), 19.2 (CH(CH_3)^A(CH_3)^B), 23.8 (CH_2), 23.8 (CH_2), 27.2 (CH_2), 27.2 (CH_2), 27.3 (CH_2), 27.4 (CH_2), 27.5 (CH_2), 28.2 (CH_2), 34.1 (CH(CH_3)$_2$), 69.9 (ArC(1)-OCH_2), 70.3 (ArC(4)-OCH_2), 113.8 (ArC(3)H), 120.9 (ArC(6)H), 123.0 (ArC(5)-ArC(5)H), 127.3 (ArC(5)), 133.8 (ArC(5)-ArC(3)), 137.1 (ArC(5)-ArC(4)H), 142.2 (ArC(2)), 144.8 (ArC(1)), 148.1 (ArC(5)-ArC(6)H), 149.8 (ArC(5)-ArC(2)H), 150.1 (ArC(4)), 174.6 (*C*=O); HRMS (APCI$^+$) $C_{25}H_{33}O_3N$ ([M+Na]$^+$) requires 434.23018, found 434.2297 (–1.16 ppm).

(*Sp*)-1^5-(pyridin-3-yl)-2,13-dioxa-1(1,4)-benzenacyclotridecaphan-1^2-ol S38

<Chromatogram>

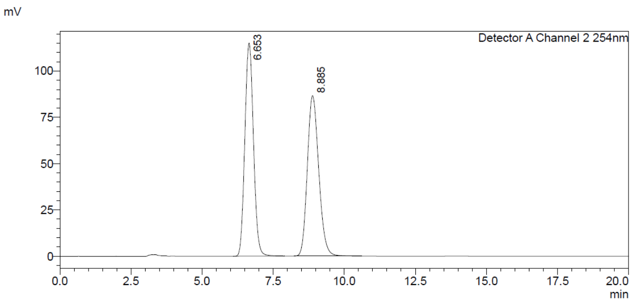

<Peak Table>

Detector A Channel 2 254nm

Peak#	Ret. Time	Area%
1	6.653	50.078
2	8.885	49.922
Total		100.000

<Chromatogram>

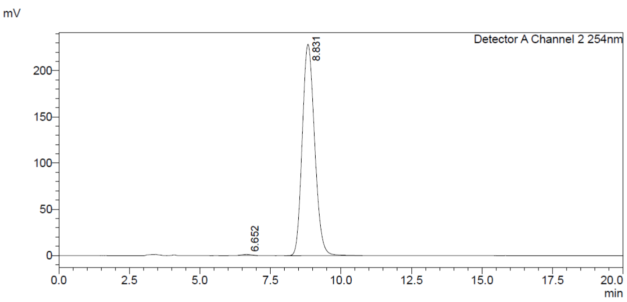

<Peak Table>

Detector A Channel 2 254nm

Peak#	Ret. Time	Area%
1	6.652	0.317
2	8.831	99.683
Total		100.000

(*Rp*)-1^{5}-(pyridin-3-yl)-2,13-dioxa-1(1,4)-benzenacyclotridecaphane-1^{2}-yl isobutyrate 6

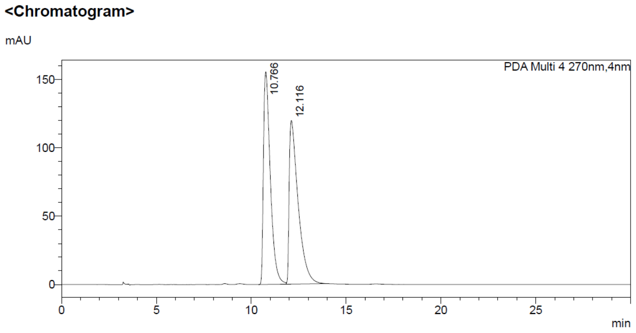

<Peak Table>

PDA Ch4 270nm

Peak#	Ret. Time	Area%
1	10.766	49.877
2	12.116	50.123
Total		100.000

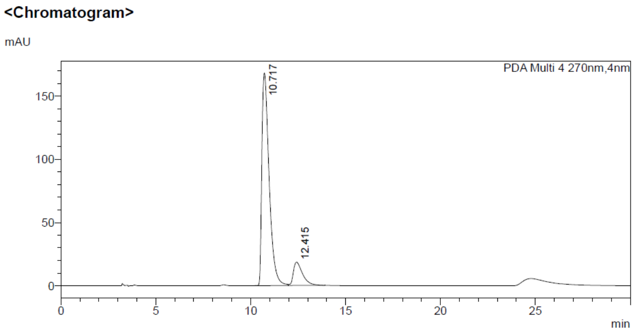

<Peak Table>

PDA Ch4 270nm

Peak#	Ret. Time	Area%
1	10.717	87.771
2	12.415	12.229
Total		100.000

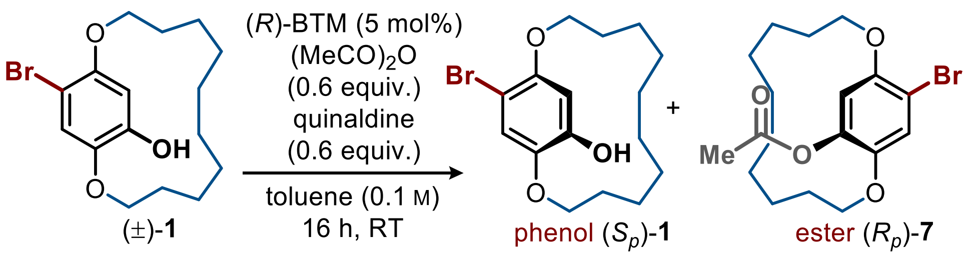

Acetic anhydride (0.12 mmol, 0.6 equiv.) was added to a solution of macrocycle phenol derivatives (0.20 mmol, 1.0 equiv.), isothiourea (5 mol%) and quinaldine (0.12 mmol, 0.6 equiv.) in toluene (2 ml, 0.1 M), the resulting mixture was then stirred overnight at room temperature. The mixture was then concentrated under reduced pressure to give the crude residue, which was subsequently purified by silica gel chromatography column using 10:1 petrol ether/EtOAc as eluent gave the phenol (*Sp*)-**1** (yield 47%, 89.5:10.5 er) as a mixture with ester (*Rp*)-**7** (yield 46%, 90:10 er); chiral HPLC analysis, ChiralPak ID-H (3% *i*-PrOH : hexane, flow rate 1 mLmin^{-1}, 254 nm, 30 °C), tR major: 13.9 min, tR minor: 15.5 min, 89.5:10.5 er er; and the product ester (*Rp*)-**7** as a mixture with phenol (*Sp*)-**1** (90:10 er); chiral HPLC analysis, ChiralPak ID-H (3% *i*-PrOH : hexane, flow rate 1 mLmin^{-1}, 254 nm, 30 °C), tR major: 11.9 min, tR minor: 43.9 min, 90:10 er; ν_{max} (film)/cm^{-1} 2926 (C–H), 2855 (C–H), 1765 (C=O), 1487 (C=C), 1184 (C–O), 1134 (C–O), 895 1134 (C–Br); **^{1}H NMR** (500 MHz, $CDCl_3$) δ_H: 0.77-0.90 (4H, m, 2 × C*H*$_2$), 0.92-1.05 (2H, m, C*H*$_2$), 1.07-1.31 (6H, m, 3 × C*H*$_2$), 1.51-1.87 (4H, m, 2 × C*H*$_2$), 2.33 (3H, d, *J* 7.0, C*H*$_3$), 4.17 (1H, ddd, *J* 12.0, *J* 8.0, *J* 3.8, ArC(1)-OC*H*^{A}H^B), 4.23-4.39 (3H, m, ArC(1)-OCH^A*H*^{B}, ArC(4)-OCH$_2$), 6.80 (1H, s, ArC(3)*H*), 7.30 (1H, s, ArC(6)*H*); **^{13}C{1H} NMR** (126 MHz, $CDCl_3$) δ_C: 20.7 (CH_3), 23.6 (CH_2), 23.7(CH_2), 27.2 (CH_2), 27.2 (CH_2), 27.3 (CH_2), 27.4 (CH_2), 27.5 (CH_2), 28.1 (CH_2), 70.4 (ArC(4)-OCH$_2$), 70.4 (ArC(1)-OCH$_2$), 110.0 (Ar*C*(5)), 113.5 (ArC(3)H), 123.7 (ArC(6)H), 141.3 (Ar*C*(2)), 145.0 (ArC(1)), 149.5 (Ar*C*(4)), 168.4 (*C*=O); HRMS (APCI$^+$) $C_{18}H_{25}O_4Br$ ([M+Na]$^+$) requires 407.08284, found 407.0825 (–0.72 ppm).

(*Sp*)-1^5-bromo-2,13-dioxa-1(1,4)-benzenacyclotridecaphan-1^2-ol 1

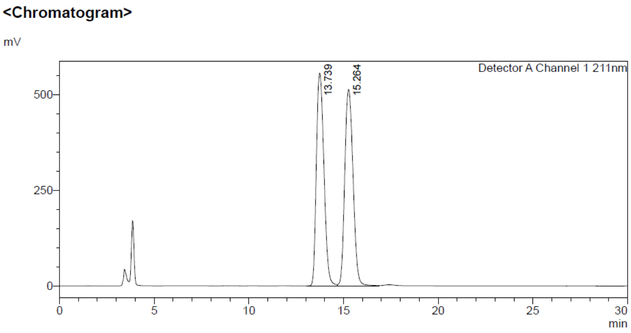

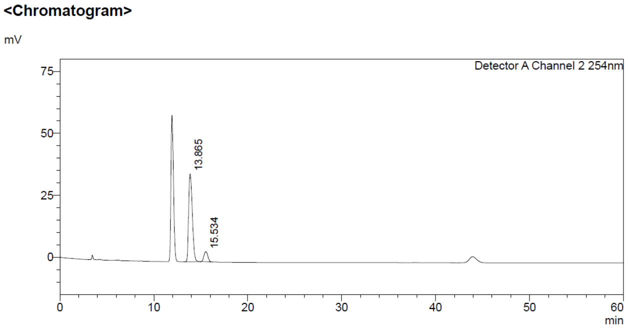

<Peak Table>

Detector A Channel 1 211nm

Peak#	Ret. Time	Area%
1	13.739	49.683
2	15.264	50.317
Total		100.000

<Peak Table>

Detector A Channel 2 254nm

Peak#	Ret. Time	Area%
1	13.865	89.417
2	15.534	10.583
Total		100.000

(*Rp*)-1^5-bromo-2,13-dioxa-1(1,4)-benzenacyclotridecaphane-1^2-yl acetate 7

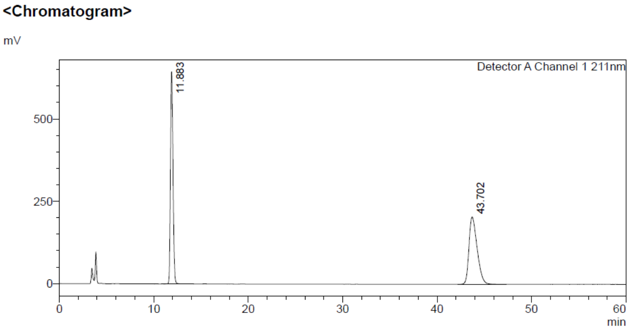

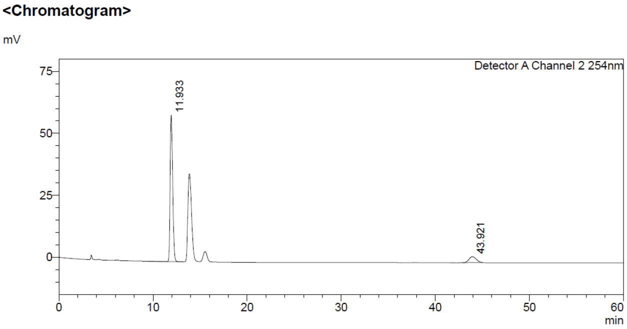

<Peak Table>

Detector A Channel 1 211nm

Peak#	Ret. Time	Area%
1	11.883	49.513
2	43.702	50.487
Total		100.000

<Peak Table>

Detector A Channel 2 254nm

Peak#	Ret. Time	Area%
1	11.933	89.703
2	43.921	10.297
Total		100.000

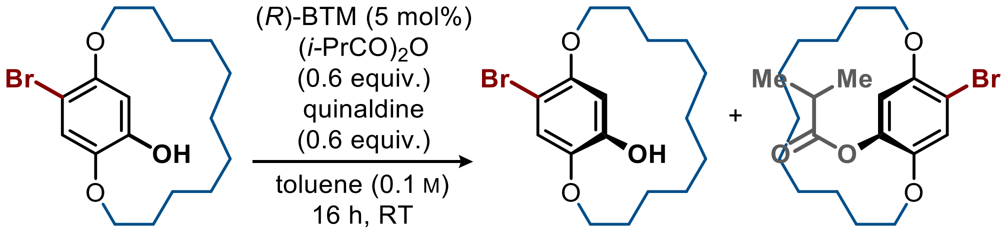

(±)-**S45** phenol (S_p)-**S45** ester (R_p)-**8**

Following General Procedure E and purification of the residue by silica gel chromatography column using 10:1 petrol ether/EtOAc as eluent gave the product (*Sp*)-**S45** (yield 39%, 96:4 er); $[\alpha]^{20}_{D}$ +7.2 (c 1.2 in $CHCl_3$); chiral HPLC analysis, ChiralPak OD-H (5% *i*-PrOH : hexane, flow rate 1 mLmin^{-1}, 254 nm, 30 °C), tR minor: 8.0 min, tR major: 12.4 min, 96:4 er; and the product (*Rp*)-**8** as a colourless oil (yield 49%, 90.5:9.5 er); $[\alpha]^{20}_{D}$ –10.6 (c 1.8 in $CHCl_3$); chiral HPLC analysis, ChiralPak ID-H (3% *i*-PrOH : hexane, flow rate 1 mLmin^{-1}, 254 nm, 30 °C), tR major: 8.0 min, tR minor: 23.3 min, 90.5:9.5 er; ν_{max} (film)/cm^{-1} 2968 (C–H), 2924 (C–H), 1761 (C=O), 1489 (C=C), 1136 (C–O), 1090 (C–O); ^{1}**H NMR** (500 MHz, $CDCl_3$) δ_H: 0.8-0.9 (2H, m, CH_2), 0.93-1.13

(6H, m, 3 × C*H*$_2$), 1.14-1.31 (6H, m, 3 × C*H*$_2$), 1.33 (3H, d, *J* 6.9, CH(C*H*$_3$)^A(CH$_3$)^B), 1.36 (3H, d, *J* 7.0, CH(CH$_3$)^A(C*H*$_3$)^B), 1.59-1.77 (4H, m, 2 × C*H*$_2$), 2.84 (1H, hept, *J* 7.0, C*H*(CH$_3$)$_2$), 4.17-4.28 (2H, m, ArC(4)-OC*H*$_2$), 4.31 (2H, t, *J* 5.5, ArC(1)-OC*H*$_2$), 6.78 (1H, s, ArC(3)*H*), 7.27 (1H, s, ArC(6)*H*); **^{13}C{1H} NMR** (126 MHz, CDCl$_3$) δ_C: 18.9 (CH(*C*H$_3$)^A(CH$_3$)^B), 19.2 (CH(CH$_3$)^A(*C*H$_3$)^B), 23.9 (*C*H$_2$), 23.9 (*C*H$_2$), 27.1 (*C*H$_2$), 27.1 (*C*H$_2$), 27.2 (*C*H$_2$), 28.3 (*C*H$_2$), 28.5 (*C*H$_2$), 28.6 (*C*H$_2$), 28.7 (*C*H$_2$), 34.0 (*C*H(CH$_3$)$_2$), 70.2 (ArC(4)-O*C*H$_2$), 70.3 (ArC(1)-O*C*H$_2$), 110.1 (Ar*C*(5)), 112.3 (Ar*C*(3)H), 122.0 (Ar*C*(6)H), 141.0 (Ar*C*(2)), 145.3 (Ar*C*(1)), 149.7 (Ar*C*(4)), 174.6 (*C*=O); HRMS (APCI$^+$) $C_{21}H_{31}O_4Br$ ([M+Na]$^+$) requires 449.12979, found 449.1296 (–0.52 ppm).

(*Sp*)-1^5-bromo-2,14-dioxa-1(1,4)-benzenacyclotetradecaphan-1^2-ol S45

<Chromatogram>

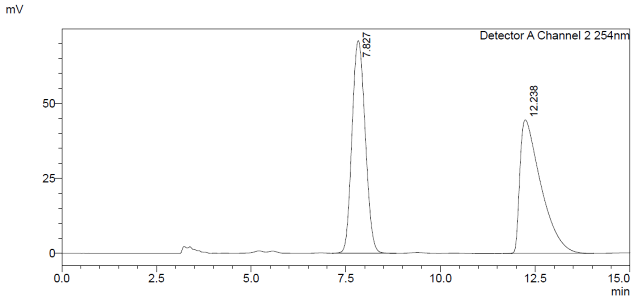

<Peak Table>

Detector A Channel 2 254nm

Peak#	Ret. Time	Area%
1	7.827	50.076
2	12.238	49.924
Total		100.000

<Chromatogram>

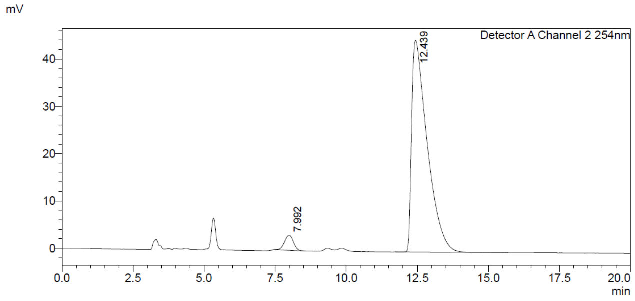

<Peak Table>

Detector A Channel 2 254nm

Peak#	Ret. Time	Area%
1	7.992	3.945
2	12.439	96.055
Total		100.000

(*Rp*)-1^5-bromo-2,14-dioxa-1(1,4)-benzenacyclotetradecaphane-1^2-yl isobutyrate 8

<Chromatogram>

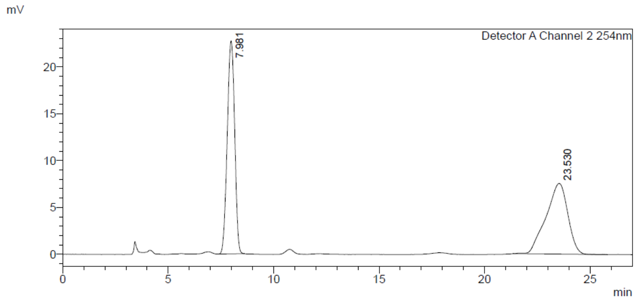

<Peak Table>

Detector A Channel 2 254nm

Peak#	Ret. Time	Area%
1	7.981	50.705
2	23.530	49.295
Total		100.000

<Chromatogram>

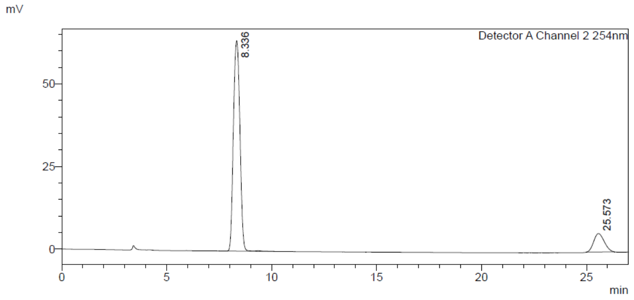

<Peak Table>

Detector A Channel 2 254nm

Peak#	Ret. Time	Area%
1	8.336	87.561
2	25.573	12.439
Total		100.000

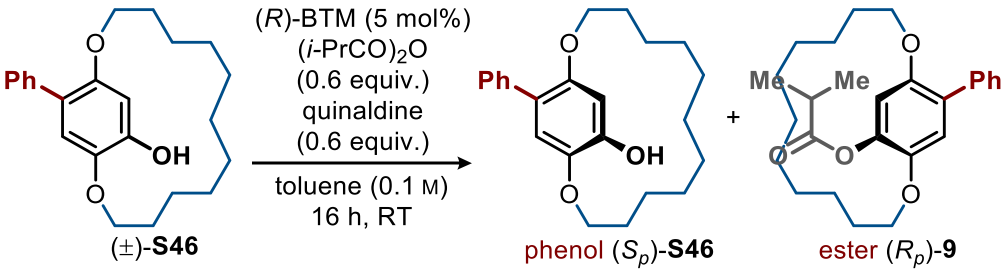

Following General Procedure E and purification of the residue by silica gel chromatography column using 10:1 petrol ether/EtOAc as eluent gave the product (*Sp*)-**S46** (yield 40%, 93.5:6.5 er); $[\alpha]_D^{20}$ +26.3 (c 1.0 in $CHCl_3$); chiral HPLC analysis, ChiralPak OD-H (5% *i*-PrOH : hexane, flow rate 1 mLmin^{-1}, 254 nm, 30 °C), tR minor: 6.8 min, tR major: 10.3 min, 93.5:6.5 er; and the product (*Rp*)-**9** as a colourless oil (yield 48%, 88:12 er); $[\alpha]_D^{20}$ –32.8 (c 1.6 in $CHCl_3$); chiral HPLC analysis, ChiralPak ID-H (3% *i*-PrOH : hexane, flow rate 1 mLmin^{-1}, 254 nm, 30 °C), tR major: 6.4 min, tR minor: 20.3 min, 88:12 er; ν_{max} (film)/cm^{-1} 2924 (C–H), 2853 (C–H), 1759 (C=O), 1487 (C=C), 1190 (C–O), 1126 (C–O); **^{1}H NMR** (500 MHz, $CDCl_3$) δ_H: 0.85-1.17 (11H, m, 5 × C*H*$_2$, C*H*^{A}H^B), 1.20-1.35 (3H, m, C*H*$_2$, CH^A*H*^{B}), 1.37 (3H, d, *J* 7.0, CH(C*H*$_3$)^A(CH$_3$)^B), 1.40 (3H, d, *J* 7.0, CH(CH$_3$)^A(C*H*$_3$)^B), 1.41-1.47 (1H, m, C*H*^{A}H^B), 1.55-1.60 (1H, m, CH^A*H*^{B}), 1.61-1.77 (2H, m, C*H*$_2$), 2.88 (1H, hept, *J* 7.0, C*H*(CH$_3$)$_2$), 4.02 (1H, ddd, *J* 11.8, *J* 7.4, *J* 4.0, ArC(4)-OC*H*^{A}H^B), 4.14-4.27 (2H, m, ArC(4)-OCH^A*H*^{B}, ArC(1)-OC*H*^{A}H^B), 4.34 (1H, ddd, *J* 11.4, *J* 7.2, *J* 3.9, ArC(1)-OCH^A*H*^{B}), 6.83 (1H, s, ArC(3)*H*), 7.06 (1H, s, ArC(6)*H*), 7.32-7.38 (1H, m, ArC(5)-PhC(4)*H*), 7.40-7.47 (2H, m, ArC(5)-PhC(3,5)*H*), 7.51-7.57 (2H, m, ArC(5)-PhC(2,6)*H*); **^{13}C{1H} NMR** (126 MHz, $CDCl_3$) δ_C: 18.9 (CH(*C*H$_3$)^A(CH$_3$)^B), 19.3 (CH(CH$_3$)^A(*C*H$_3$)^B), 24.1 (*C*H$_2$), 24.1 (*C*H$_2$), 27.2 (*C*H$_2$), 27.3 (*C*H$_2$), 27.3 (*C*H$_2$), 28.4 (*C*H$_2$), 28.5 (*C*H$_2$), 28.6 (*C*H$_2$), 28.7 (*C*H$_2$), 34.1 (*C*H(CH$_3$)$_2$), 69.6 (ArC(4)-OCH$_2$), 69.8 (ArC(1)-OCH$_2$), 112.4 (ArC(3)H), 119.7 (ArC(6)H), 127.0 (ArC(5)-PhC(4)H), 128.0 (ArC(5)-Ph*C*(3,5)H), 129.5 (ArC(5)-PhC(2,6)H), 130.6 (Ar*C*(5)), 138.3 (ArC(5)-Ph*C*(1)), 140.9 (Ar*C*(2)), 144.6 (Ar*C*(1)), 149.9 (Ar*C*(4)), 174.9 (*C*=O); HRMS (APCI$^+$) $C_{27}H_{36}O_4$ ([M+Na]$^+$) requires 447.25058, found 447.2502 (–0.93 ppm).

(*Sp*)-1^5-phenyl-2,14-dioxa-1(1,4)-benzenacyclotetradecaphan-1^2-ol S46

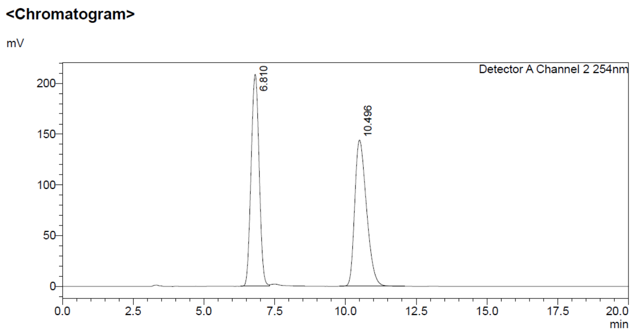

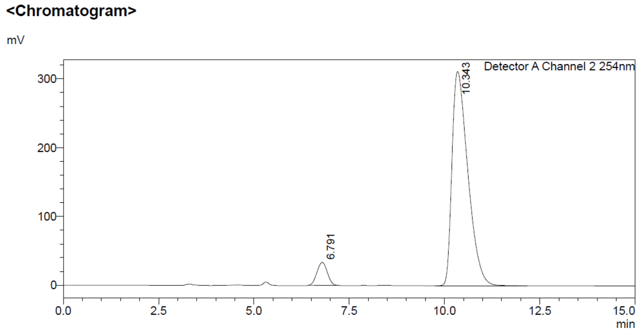

<Peak Table>

Detector A Channel 2 254nm

Peak#	Ret. Time	Area%
1	6.810	49.723
2	10.496	50.277
Total		100.000

<Peak Table>

Detector A Channel 2 254nm

Peak#	Ret. Time	Area%
1	6.791	6.529
2	10.343	93.471
Total		100.000

(*Rp*)-1[5]-phenyl-2,14-dioxa-1(1,4)-benzenacyclotetradecaphane-1[2]-yl isobutyrate 9

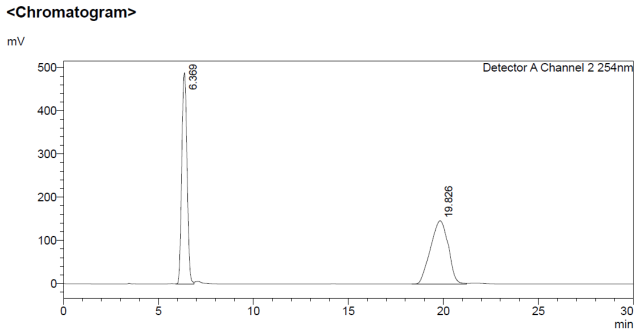

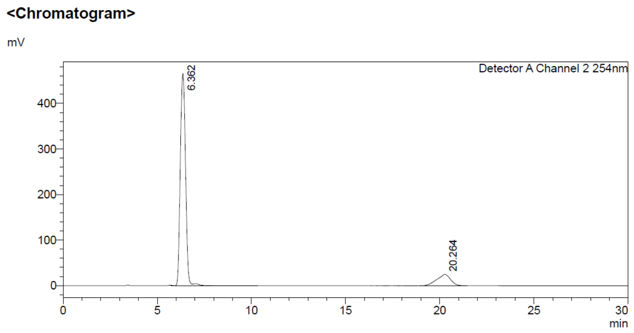

<Peak Table>

Detector A Channel 2 254nm

Peak#	Ret. Time	Area%
1	6.369	49.877
2	19.826	50.123
Total		100.000

<Peak Table>

Detector A Channel 2 254nm

Peak#	Ret. Time	Area%
1	6.362	88.114
2	20.264	11.886
Total		100.000

(*Rp*)-1[5]-bromo-2,15-dioxa-1(1,4)-benzenacyclopentadecaphane-1[2]-yl isobutyrate 12

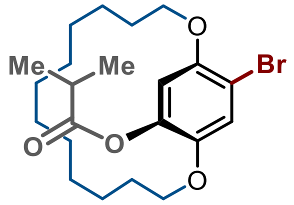

Following General Procedure F and purification of the residue by silica gel chromatography column using 10:1 petrol ether/EtOAc as eluent gave the product **12** as a colourless oil (yield 96%, 93:7 er); $[\alpha]_D^{20}$ −28.3 (c 2.4 in $CHCl_3$); chiral HPLC analysis, ChiralPak ID-H (3% *i*-PrOH : hexane, flow rate 1 $mLmin^{-1}$, 211 nm, 30 °C), tR major: 7.0 min, tR minor: 18.0 min, 93:7 er; ν_{max} (film)/cm^{-1} 2924 (C–H), 2853 (C–H), 1761 (C=O), 1491 (C=C), 1200 (C–O), 1136 (C–O), 1090 (C–O); **^{1}H NMR** (500 MHz, $CDCl_3$) δ_H: 0.97-1.32 (15H, m, 7 × CH_2, CH^AH^B), 1.34 (3H, d, *J* 7.0, $CH(CH_3)^A(CH_3)^B$), 1.36 (3H, d, *J* 7.0, $CH(CH_3)^A(CH_3)^B$), 1.41-1.50 (1H, m, CH^AH^B), 1.54-1.62 (2H, m, CH_2), 1.67-1.88 (2H, m, CH_2), 2.85 (1H, hept, *J* 7.0, $CH(CH_3)_2$), 4.08-4.17 (1H, m, ArC(1)-OCH^AH^B), 4.21-4.25 (1H, m, ArC(1)-OCH^AH^B), 4.26-4.28 (1H, m, ArC(4)-OCH^AH^B), 4.28-4.34 (1H, m, ArC(4)-

OCH^AH^B), 6.73 (1H, s, ArC(3)*H*), 7.23 (1H, s, ArC(6)*H*); ^{13}**C{1H} NMR** (126 MHz, $CDCl_3$) δ_C: 18.9 (CH(CH_3)^A(CH_3)^B), 19.2 (CH(CH_3)^A(CH_3)^B), 23.6 (CH_2), 24.0 (CH_2), 26.7 (CH_2), 27.3 (CH_2), 27.4 (CH_2), 27.6 (CH_2), 27.9 (CH_2), 27.9 (CH_2), 28.4 (CH_2), 28.5 (CH_2), 34.0 (CH(CH_3)$_2$), 69.3 (ArC(4)-OCH_2), 69.6 (ArC(1)-OCH_2), 109.4 (Ar*C*(5)), 111.3 (Ar*C*(3)H), 120.8 (Ar*C*(6)H), 140.6 (Ar*C*(2)), 144.2 (Ar*C*(1)), 148.6 (Ar*C*(4)), 174.7 (*C*=O); HRMS (APCI$^+$) $C_{22}H_{33}O_4Br$ ([M+Na]$^+$) requires 463.14544, found 463.1453 (–0.32 ppm).

<Chromatogram>

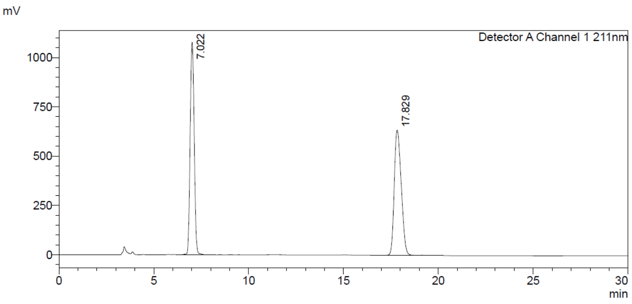

<Peak Table>

Detector A Channel 1 211nm

Peak#	Ret. Time	Area%
1	7.022	49.595
2	17.829	50.405
Total		100.000

<Chromatogram>

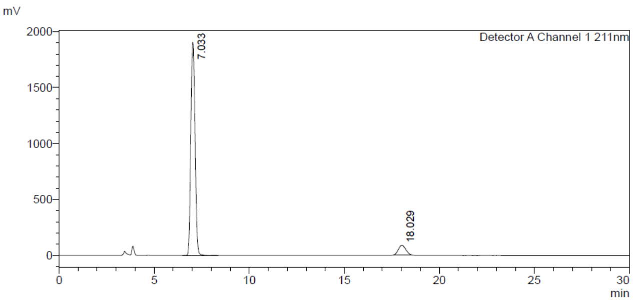

<Peak Table>

Detector A Channel 1 211nm

Peak#	Ret. Time	Area%
1	7.033	92.777
2	18.029	7.223
Total		100.000

(*Rp*)-1^5-vinyl-2,15-dioxa-1(1,4)-benzenacyclopentadecaphane-1^2-yl isobutyrate 13

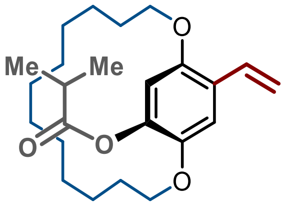

Following General Procedure F with N_2 atmosphere and purification of the residue by silica gel chromatography column using 4:1 petrol ether/EtOAc as eluent gave the product **13** as a colourless oil (yield 88%, 95:5 er) (without N_2 atmosphere: yield 18%, 83:17 er); $[\alpha]_D^{20}$ –19.2 (c 0.5 in $CHCl_3$); chiral HPLC analysis, ChiralPak ID-H (3% *i*-PrOH : hexane, flow rate 1 mLmin^{-1}, 254 nm, 30 °C), tR major: 6.5 min, tR minor: 14.4 min, 95:5 er; ν_{max} (film)/cm^{-1} 2924 (C–H), 2855 (C–H), 1759 (C=O), 1497 (C=C), 1194 (C–O), 1115 (C–O), 1094 (C–O); ^{1}**H NMR** (500 MHz, $CDCl_3$) δ_H: 0.92-1.05 (8H, m, 4 × CH_2), 1.06-1.32 (6H, m, 3 × CH_2), 1.34 (4H, d, *J* 7.0, CH(CH_3)^A(CH_3)^B, CH^AH^B), 1.37 (4H, d, *J* 7.0, CH(CH_3)^A(CH_3)^B, CH^AH^B), 1.50-1.60 (2H, m, CH_2), 1.67-1.88 (2H, m, CH_2), 2.85 (1H, hept, *J* 7.0, C*H*(CH_3)$_2$), 4.07-4.20 (2H, m, ArC(1)-OCH^AH^B, ArC(4)-OCH^AH^B), 4.21-4.36 (2H, m, ArC(4)-OCH^AH^B, ArC(1)-OCH^AH^B), 5.26 (1H, dd, *J* 11.1, *J* 1.4, ArC(5)-CH=CH_AH$_B$), 5.66 (1H, dd, *J* 17.7, *J* 1.4, ArC(5)-CH=CH$_A$$H_B$), 6.67 (1H, s, ArC(3)*H*), 7.05 (1H, dd, *J* 17.7, *J* 11.1, ArC(5)-C*H*=CH_2), 7.15 (1H, s, ArC(6)*H*); ^{13}**C{1H} NMR** (126 MHz, $CDCl_3$) δ_C: 18.9 (CH(CH_3)^A(CH_3)^B), 19.2 (CH(CH_3)^A(CH_3)^B), 23.7 (CH_2), 24.1 (CH_2), 26.9 (CH_2), 27.4 (CH_2), 27.5 (CH_2), 27.5 (CH_2), 27.8 (CH_2), 27.9 (CH_2), 28.4 (CH_2), 28.4 (CH_2), 34.1

(CH(CH$_3$)$_2$), 68.8 (ArC(1)-OCH$_2$), 69.2 (ArC(4)-OCH$_2$), 110.3 (Ar*C*(3)H), 114.0 (Ar*C*(5)-CH=CH$_2$), 114.4 (Ar*C*(5)-CH=CH$_2$), 125.9 (Ar*C*(5)), 131.4 (Ar*C*(6)H), 141.2 (Ar*C*(2)), 143.6 (Ar*C*(1)), 149.4 (Ar*C*(4)), 174.9 (*C*=O); HRMS (APCI$^+$) $C_{24}H_{36}O_4$ ([M+Na]$^+$) requires 411.25058, found 411.2501 (–1.20 ppm).

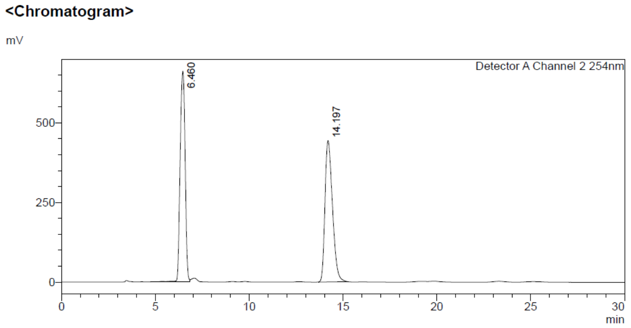

<Peak Table>

Detector A Channel 2 254nm

Peak#	Ret. Time	Area%
1	6.460	49.976
2	14.197	50.024
Total		100.000

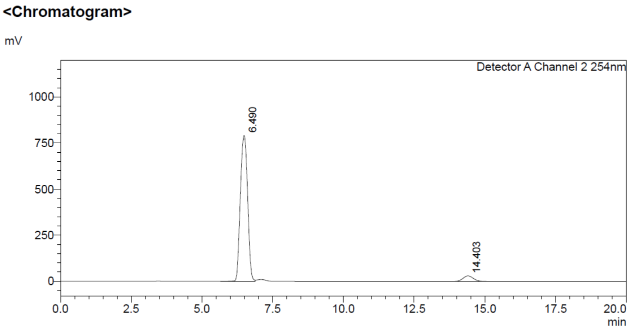

<Peak Table>

Detector A Channel 2 254nm

Peak#	Ret. Time	Area%
1	6.490	94.977
2	14.403	5.023
Total		100.000

(*Rp*)-1^5-phenyl-2,15-dioxa-1(1,4)-benzenacyclopentadecaphane-1^2-yl isobutyrate 11

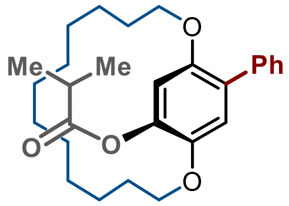

Following General Procedure F and purification of the residue by silica gel chromatography column using 10:1 petrol ether/EtOAc as eluent gave the product **11** as a colourless oil (yield 92%, 97:3 er); $[\alpha]_D^{20}$ –41.6 (c 1.9 in CHCl$_3$); chiral HPLC analysis, ChiralPak ID-H (3% *i*-PrOH : hexane, flow rate 1 mLmin^{-1}, 254 nm, 30 °C), tR major: 5.7 min, tR minor: 15.0 min, 97:3 er; ν_{max} (film)/cm^{-1} 2924 (C–H), 2853 (C–H), 1759 (C=O), 1487 (C=C), 1192 (C–O), 1124 (C–O); **^{1}H NMR** (500 MHz, CDCl$_3$) δ_H: 0.95-1.35 (16H, m, 8 × C*H*$_2$), 1.37 (3H, d, *J* 6.9, CH(C*H*$_3$)^A(CH$_3$)^B), 1.41 (3H, d, *J* 7.0, CH(CH$_3$)^A(C*H*$_3$)^B), 1.43-1.63 (2H, m, C*H*$_2$), 1.69-1.89 (2H, m, C*H*$_2$), 2.89 (1H, hept, *J* 7.0, C*H*(CH$_3$)$_2$), 3.98 (1H, ddd, *J* 12.2, *J* 8.5, *J* 4.3, ArC(4)-OC*H*^{A}H^B), 4.11-4.16 (1H, m, ArC(1)-OC*H*^{A}H^B), 4.16-4.22 (1H, m, ArC(4)-OCH^A*H*^{B}), 4.32 (1H, ddd, *J* 11.1, *J* 6.2, *J* 4.4, ArC(1)-OCH^A*H*^{B}), 6.79 (1H, s, ArC(3)*H*), 7.02 (1H, s, ArC(6)*H*), 7.33-7.39 (1H, m, ArC(5)-PhC(4)*H*), 7.41-7.48 (2H, m, ArC(5)-PhC(3,5)*H*), 7.52-7.58 (2H, m, ArC(5)-PhC(2,6)*H*); **^{13}C{1H} NMR** (126 MHz, CDCl$_3$) δ_C: 19.0 (CH(CH$_3$)^A(CH$_3$)^B), 19.3 (CH(CH$_3$)^A(CH$_3$)^B), 23.6 (CH$_2$), 23.8 (CH$_2$), 26.7 (CH$_2$), 27.3 (CH$_2$), 27.4 (CH$_2$), 27.5 (CH$_2$), 27.7 (CH$_2$), 28.0 (CH$_2$), 28.5 (CH$_2$), 28.5 (CH$_2$), 34.1 (CH(CH$_3$)$_2$), 68.7 (ArC(4)-OCH$_2$), 69.0 (ArC(1)-OCH$_2$), 111.0 (Ar*C*(3)H), 118.8 (Ar*C*(6)H), 127.0 (ArC(5)-Ph*C*(4)H), 128.0 (ArC(5)-Ph*C*(3,5)H), 129.5 (ArC(5)-PhC(2,6)H), 129.8 (ArC(5)), 138.4 (ArC(5)-

PhC(1)), 140.5 (ArC(2)), 143.5 (ArC(1)), 148.9 (ArC(4)), 175.1 (C=O); HRMS (APCI$^+$) $C_{28}H_{38}O_4$ ([M+Na]$^+$) requires 461.26623, found 461.2665 (+0.58 ppm).

<Chromatogram>

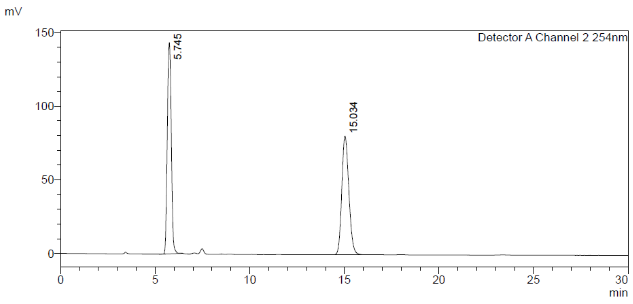

<Peak Table>

Detector A Channel 2 254nm

Peak#	Ret. Time	Area%
1	5.745	50.071
2	15.034	49.929
Total		100.000

<Chromatogram>

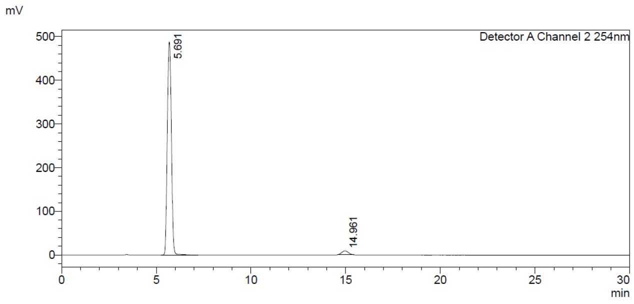

<Peak Table>

Detector A Channel 2 254nm

Peak#	Ret. Time	Area%
1	5.691	97.025
2	14.961	2.975
Total		100.000

(*Rp*)-1^5-phenyl-2,15-dioxa-1(1,4)-benzenacyclopentadecaphane-1^2-yl propionate S70

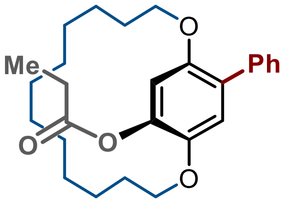

Propionic anhydride (0.10 mmol, 1.0 equiv.) was added to a solution of macrocycle phenol **10** (0.10 mmol, 1.0 equiv.), (*R*)-BTM (5 mol%) and *i*-Pr_2NEt (0.10 mmol, 1.0 equiv.) in toluene (1 ml, 0.1 M), the resulting mixture was then stirred 24 h at room temperature. The mixture was then concentrated under reduced pressure to give the crude residue and purification of the residue by silica gel chromatography column using 10:1 petrol ether/EtOAc as eluent gave the product **S70** as a colourless oil (yield 88%, 92:8 er); $[\alpha]_D^{20}$ −15.2 (c 1.3 in $CHCl_3$); chiral HPLC analysis, ChiralPak ID-H (3% *i*-PrOH : hexane, flow rate 1 mLmin^{-1}, 254 nm, 30 °C), tR major: 6.6 min, tR minor: 19.2 min, 92:8 er; ν_{max} (film)/cm^{-1} 2924 (C–H), 2853 (C–H), 1763 (C=O), 1487 (C=C), 1188 (C–O), 1126 (C–O); **^{1}H NMR** (500 MHz, $CDCl_3$) δ_H: 0.78-1.28 (14H, m, 7 × CH_2), 1.28-1.36 (4H, CH_3, CH^AH^B), 1.37-1.50 (2H, d, CH_2), 1.51-1.63 (1H, m, CH^AH^B), 1.67-1.88 (2H, m, CH_2), 2.66 (2H, q, *J* 7.6, CH_2CH_3), 3.89-4.05 (1H, m, ArC(4)-OCH^AH^B), 4.09-4.32 (2H, m, ArC(1)-OCH^AH^B, ArC(4)-OCH^AH^B), 4.25-4.38 (1H, m, ArC(1)-OCH^AH^B), 6.80 (1H, s, ArC(3)*H*), 7.02 (1H, s, ArC(6)*H*), 7.31-7.39 (1H, m, ArC(5)-PhC(4)*H*), 7.39-7.48 (2H, m, ArC(5)-PhC(3,5)*H*), 7.49-7.59 (2H, m, ArC(5)-PhC(2,6)*H*); **^{13}C{1H} NMR** (126 MHz, $CDCl_3$) δ_C: 9.3 (CH_3), 23.7 (CH_2), 23.8 (CH_2), 26.8 (CH_2), 27.4 (CH_2), 27.5 (CH_2), 27.6 (CH_2), 27.6 (CH_2CH_3), 27.8 (CH_2), 28.0 (CH_2), 28.5 (CH_2), 28.5 (CH_2), 68.8 (ArC(4)-OCH_2), 69.2 (ArC(1)-OCH_2), 111.2 (ArC(3)H), 119.0 (ArC(6)H), 127.0 (ArC(5)-PhC(4)H), 128.0 (ArC(5)-PhC(3,5)H), 129.5 (ArC(5)-Ph*C*(2,6)H), 129.9 (ArC(5)), 138.4 (ArC(5)-

Ph*C*(1)), 140.5 (Ar*C*(2)), 143.5 (Ar*C*(1)), 149.0 (Ar*C*(4)), 172.4 (*C*=O); HRMS (APCI$^+$) $C_{27}H_{36}O_4$ ([M+Na]$^+$) requires 447.25058, found 447.2502 (–0.83 ppm).

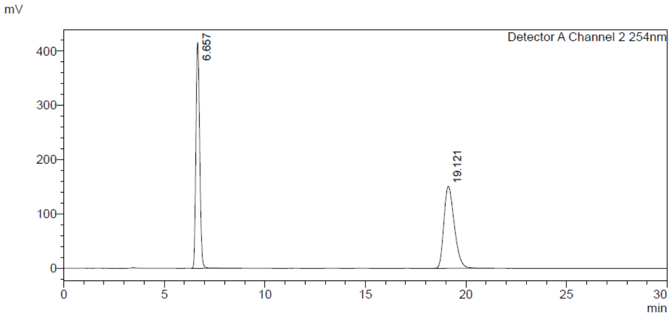

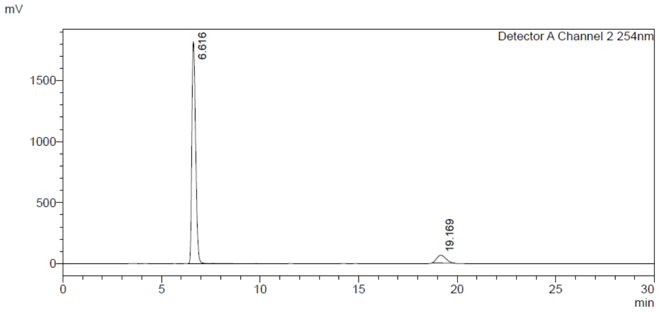

<Peak Table>

Detector A Channel 2 254nm

Peak#	Ret. Time	Area%
1	6.657	50.033
2	19.121	49.967
Total		100.000

<Peak Table>

Detector A Channel 2 254nm

Peak#	Ret. Time	Area%
1	6.616	92.063
2	19.169	7.937
Total		100.000

(*Rp*)-1^5-phenyl-2,15-dioxa-1(1,4)-benzenacyclopentadecaphane-1^2-yl benzoate S71

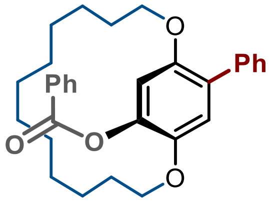

Benzoic anhydride (0.10 mmol, 1.0 equiv.) was added to a solution of macrocycle phenol **10** (0.10 mmol, 1.0 equiv.), (*R*)-BTM (5 mol%) and *i*-Pr_2NEt (0.10 mmol, 1.0 equiv.) in toluene (1 ml, 0.1 M), the resulting mixture was then stirred 24 h at room temperature. The mixture was then concentrated under reduced pressure to give the crude residue and purification of the residue by silica gel chromatography column using 10:1 petrol ether/EtOAc as eluent gave the product **S71** as a colourless oil (yield 83%, 97:3 er); $[\alpha]_D^{20}$ –43.5 (c 2.1 in $CHCl_3$); chiral HPLC analysis, ChiralPak ID-H (3% *i*-PrOH : hexane, flow rate 1 mLmin^{-1}, 254 nm, 30 °C), tR major: 9.1 min, tR minor: 42.1 min, 97:3 er; ν_{max} (film)/cm^{-1} 2924 (C–H), 2855 (C–H), 1742 (C=O), 1487 (C=C), 1260 (C=C), 1192 (C–O); ^{1}**H NMR** (500 MHz, $CDCl_3$) δ_H: 1.00-1.18 (10H, m, 5 × C*H*$_2$), 1.18-1.25 (3H, C*H*$_2$, C*H*^{A}H^B), 1.25-1.38 (2H, m, C*H*$_2$), 1.38-1.52 (2H, m, C*H*$_2$), 1.53-1.63 (1H, m, CH^A*H*^{B}), 1.71-1.86 (2H, m, C*H*$_2$), 4.01 (1H, ddd, *J* 12.2, *J* 8.3, *J* 4.4, ArC(4)-OC*H*^{A}H^B), 4.10-4.25 (2H, m, ArC(1)-OC*H*^{A}H^B, ArC(4)-OCH^A*H*^{B}), 4.31 (1H, ddd, *J* 11.1, *J* 6.2, *J* 4.5, ArC(1)-OCH^A*H*^{B}), 6.95 (1H, s, ArC(3)*H*), 7.09 (1H, s, ArC(6)*H*), 7.32-7.40 (1H, m, ArC(5)-PhC(4)*H*), 7.41-7.49 (2H, m, ArC(5)-PhC(3,5)*H*), 7.49-7.60 (4H, m, ArC(5)-PhC(2,6)*H*, C=OPhC(3,5)*H*), 7.61-7.72 (1H, m, C=OPhC(4)*H*), 8.20-8.34 (2H, m, C=OPhC(2,6)*H*); ^{13}**C{1H} NMR** (126 MHz, $CDCl_3$) δ_C: 23.8 (CH_2), 23.9 (CH_2), 26.9 (CH_2), 27.4 (CH_2), 27.5 (CH_2), 27.6 (CH_2), 27.8 (CH_2), 28.1 (CH_2), 28.6 (CH_2), 28.6 (CH_2), 68.8 (ArC(4)-OCH_2), 69.4 (ArC(1)-OCH_2), 111.2 (Ar*C*(3)H), 119.5 (Ar*C*(6)H), 127.0 (ArC(5)-Ph*C*(4)H), 128.0 (ArC(5)-Ph*C*(3,5)H), 128.6 (C=OPh*C*(3,5)H), 129.5 (ArC(5)-Ph*C*(2,6)H), 129.6 (C=OPh*C*(1)), 130.1 (Ar*C*(5)),

130.3 (C=OPh*C*(2,6)H), 133.5 (C=OPh*C*(4)H), 138.4 (ArC(5)-Ph*C*(1)), 140.7 (Ar*C*(2)), 143.8 (Ar*C*(1)), 149.2 (Ar*C*(4)), 164.6 (*C*=O); HRMS (APCI$^+$) $C_{31}H_{36}O_4$ ([M+Na]$^+$) requires 495.25058, found 495.2505 (–0.23 ppm).

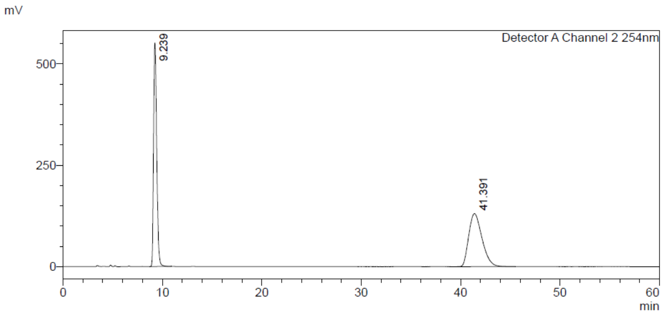

<Peak Table>

Detector A Channel 2 254nm

Peak#	Ret. Time	Area%
1	9.239	49.935
2	41.391	50.065
Total		100.000

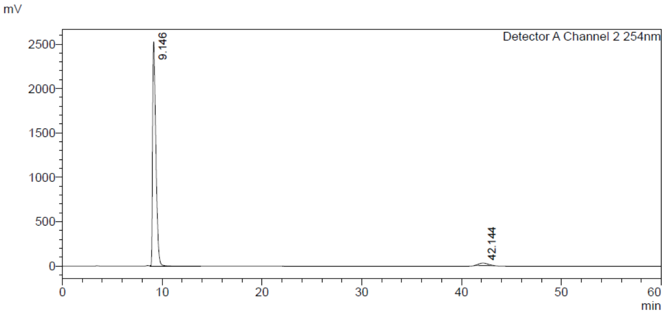

<Peak Table>

Detector A Channel 2 254nm

Peak#	Ret. Time	Area%
1	9.146	97.012
2	42.144	2.988
Total		100.000

(*Rp*)-1^5-(4-methoxyphenyl)-2,15-dioxa-1(1,4)-benzenacyclopentadecaphane-1^2-yl isobutyrate 14

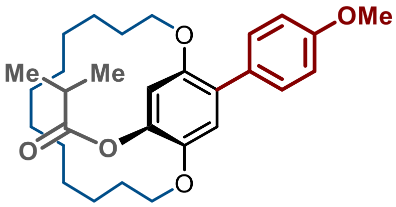

Following General Procedure F and purification of the residue by silica gel chromatography column using 10:1 petrol ether/EtOAc as eluent gave the product **14** as a colourless oil (yield 94%, 97:3 er); $[\alpha]_D^{20}$ –24.1 (c 2.5 in $CHCl_3$); chiral HPLC analysis, ChiralPak ID-H (3% *i*-PrOH : hexane, flow rate 1 mLmin^{-1}, 254 nm, 30 °C), tR major: 8.6 min, tR minor: 29.5 min, 97:3 er; ν_{max} (film)/cm^{-1} 2926 (C–H), 2853 (C–H), 1759 (C=O), 1493 (C=C), 1177 (C–O), 1125 (C–O); **^{1}H NMR** (500 MHz, $CDCl_3$) δ_H: 0.87-1.33 (15H, m, 7 × C*H*$_2$, C*H*^{A}H^B), 1.36 (3H, d, *J* 7.0, CH(C*H*$_3$)^A(CH$_3$)^B), 1.39 (3H, d, *J* 7.0, CH(CH$_3$)^A(C*H*$_3$)^B), 1.40-1.51 (2H, m, C*H*$_2$), 1.53-1.60 (1H, m, CH^A*H*^{B}), 1.67-1.85 (2H, m, C*H*$_2$), 2.88 (1H, hept, *J* 7.0, C*H*(CH$_3$)$_2$), 3.88 (3H, s, OC*H*$_3$), 3.97 (1H, ddd, *J* 12.2, *J* 8.2, *J* 4.5, ArC(4)-OC*H*^{A}H^B), 4.10-4.20 (2H, m, ArC(1)-OC*H*^{A}H^B, ArC(4)-OCH^A*H*^{B}), 4.31 (1H, ddd, *J* 11.2, *J* 6.3, *J* 4.5, ArC(1)-OCH^A*H*^{B}), 6.77 (1H, s, ArC(3)*H*), 6.97 (2H, d, *J* 8.6, ArC(5)-ArC(3,5)*H*), 6.99 (1H, s, ArC(6)*H*), 7.47 (2H, d, *J* 8.7, ArC(5)-ArC(2,6)*H*); **^{13}C{1H} NMR** (126 MHz, $CDCl_3$) δ_C: 19.0 (CH(CH$_3$)^A(CH$_3$)^B), 19.3 (CH(CH$_3$)^A(CH$_3$)^B), 23.7 (CH$_2$), 23.9 (CH$_2$), 26.9 (CH$_2$), 27.5 (2 × CH$_2$), 27.5 (CH$_2$), 27.8 (CH$_2$), 28.0 (CH$_2$), 28.4 (CH$_2$), 28.5 (CH$_2$), 34.1 (CH(CH$_3$)$_2$), 55.3 (OCH$_3$), 68.9 (ArC(1)-OCH$_2$), 69.1 (ArC(4)-OCH$_2$), 111.3 (ArC(3)H), 113.4 (ArC(5)-ArC(3,5)H), 118.6 (ArC(6)H), 129.6 (ArC(5)), 130.5 (ArC(5)-Ar*C*(2,6)H), 130.8 (ArC(5)-Ar*C*(1)), 140.2 (ArC(2)), 143.6 (Ar*C*(1)), 149.0 (Ar*C*(4)), 158.6 (ArC(5)-Ar*C*(4)), 175.0 (*C*=O); HRMS (APCI$^+$) $C_{29}H_{40}O_5$ ([M+Na]$^+$) requires 491.27680, found 491.2766 (–0.37 ppm).

<Chromatogram>

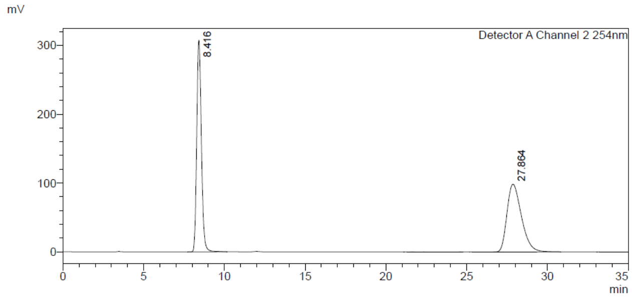

<Peak Table>

Detector A Channel 2 254nm

Peak#	Ret. Time	Area%
1	8.416	50.118
2	27.864	49.882
Total		100.000

<Chromatogram>

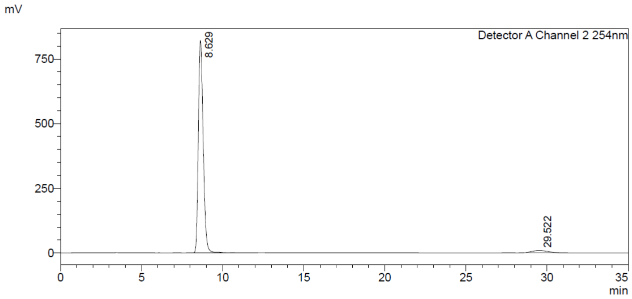

<Peak Table>

Detector A Channel 2 254nm

Peak#	Ret. Time	Area%
1	8.629	97.188
2	29.522	2.812
Total		100.000

(*Rp*)-1^5-(4-(trifluoromethyl)phenyl)-2,15-dioxa-1(1,4)-benzenacyclopentadecaphane-1^2-yl isobutyrate 15

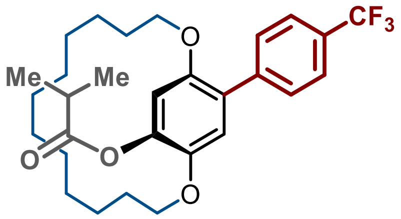

Following General Procedure F and purification of the residue by silica gel chromatography column using 10:1 petrol ether/EtOAc as eluent gave the product **15** as a colourless oil (yield 87%, 97:3 er); $[\alpha]_D^{20}$ –30.8 (c 0.6 in $CHCl_3$); chiral HPLC analysis, ChiralPak ID-H (3% *i*-PrOH : hexane, flow rate 1 $mLmin^{-1}$, 254 nm, 30 °C), tR major: 4.4 min, tR minor: 7.2 min, 97:3 er; ν_{max} (film)/cm^{-1} 2926 (C–H), 2855 (C–H), 1759 (C=O), 1497 (C=C), 1323 (C=C), 1123 (C–O), 908 (C–F); **^{1}H NMR** (500 MHz, $CDCl_3$) δ_H: 0.75-1.35 (16H, m, 8 × CH_2), 1.37 (3H, d, *J* 7.0, CH(CH_3)^A(CH_3)^B), 1.40 (3H, d, *J* 7.0, CH(CH_3)^A(CH_3)^B), 1.42-1.51 (1H, m, CH^AH^B), 1.56-1.64 (1H, m, CH^AH^B), 1.69-1.87 (2H, m, CH_2), 2.89 (1H, hept, *J* 7.0, C*H*(CH_3)$_2$), 4.00 (1H, ddd, *J* 12.2, *J* 8.4, *J* 4.2, ArC(4)-OCH^AH^B), 4.11-4.25 (2H, m, ArC(1)-OCH^AH^B, ArC(4)-OCH^AH^B), 4.32 (1H, ddd, *J* 11.2, *J* 6.3, *J* 4.4, ArC(1)-OCH^AH^B), 6.81 (1H, s, ArC(3)*H*), 7.00 (1H, s, ArC(5)*H*), 7.67 (4H, d, *J* 8.2, ArC(5)-ArC(2,3,5,6)*H*); **^{13}C{1H} NMR** (126 MHz, $CDCl_3$) δ_C: 18.9 (CH(CH_3)^A(CH_3)^B), 19.2 (CH(CH_3)^A(CH_3)^B), 23.7 (CH_2), 23.9 (CH_2), 26.8 (CH_2), 27.4 (2 × CH_2), 27.5 (CH_2), 27.8 (CH_2), 28.0 (CH_2), 28.5 (CH_2), 28.5 (CH_2), 34.1 (CH(CH_3)$_2$), 68.8 (ArC(1)-OCH_2), 69.2 (ArC(4)-OCH_2), 111.0 (ArC(3)H), 118.7 (ArC(6)H), 124.4 (q, $^1J_{CF}$ 272.0, ArC(5)-ArCF_3), 124.9 (q, $^3J_{CF}$ 3.5, ArC(5)-Ar*C*(3,5)H), 128.2 (Ar*C*(5)), 128.9 (q, $^2J_{CF}$ 32.4, ArC(5)-Ar*C*(4)), 129.8 (ArC(5)-Ar*C*(2,6)H), 141.3 (Ar*C*(2)), 142.0 (ArC(5)-Ph*C*(1)), 143.7 (Ar*C*(1)), 149.0 (Ar*C*(4)), 174.9 (*C*=O); **^{19}F{1H} NMR** (470 MHz, $CDCl_3$) δ_F: –62.36 (CF_3); HRMS (APCI$^+$) $C_{29}H_{40}O_5$ ([M+Na]$^+$) requires 529.25365, found 529.2537 (0.00 ppm).

<Chromatogram>

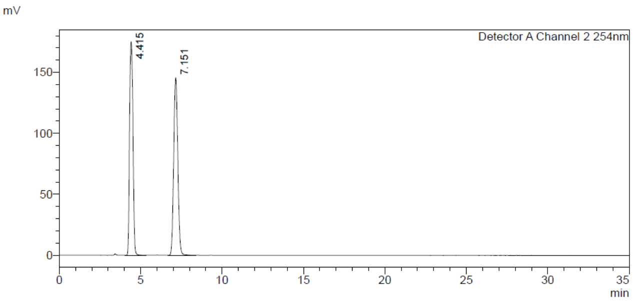

<Peak Table>

Detector A Channel 2 254nm

Peak#	Ret. Time	Area%
1	4.415	50.044
2	7.151	49.956
Total		100.000

<Chromatogram>

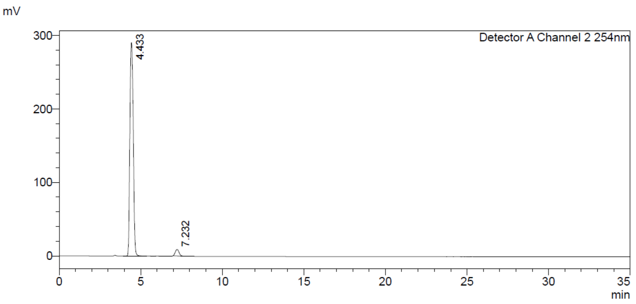

<Peak Table>

Detector A Channel 2 254nm

Peak#	Ret. Time	Area%
1	4.433	96.812
2	7.232	3.188
Total		100.000

(*Rp*)-1^5-(2-methoxyphenyl)-2,15-dioxa-1(1,4)-benzenacyclopentadecaphane-1^2-yl isobutyrate 16

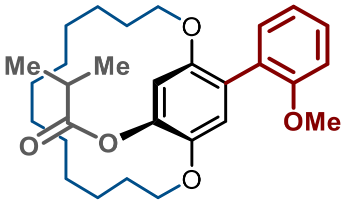

Following General Procedure F and purification of the residue by silica gel chromatography column using 10:1 petrol ether/EtOAc as eluent gave the product **16** as a colourless oil (yield 77%, 96:4 er); $[\alpha]_D^{20}$ –37.0 (c 0.3 in $CHCl_3$); chiral HPLC analysis, ChiralPak ID-H (3% *i*-PrOH : hexane, flow rate 1 mLmin^{-1}, 254 nm, 30 °C), tR major: 6.8 min, tR minor: 16.7 min, 96:4 er; ν_{max} (film)/cm^{-1} 2926 (C–H), 2854 (C–H), 1759 (C=O), 1485 (C=C), 1180 (C–O), 1128 (C–O), 1115 (C–O); **^{1}H NMR** (500 MHz, $CDCl_3$) δ_H: 1.01-1.35 (16H, m, 8 × CH_2), 1.36 (3H, d, *J* 7.0, CH(CH_3)^A(CH_3)^B), 1.39 (3H, d, *J* 7.0, CH(CH_3)^A(CH_3)^B), 1.41-1.56 (2H, m, CH_2), 1.62-1.74 (1H, m, CH^AH^B), 1.80-1.92 (1H, m, CH^AH^B), 2.88 (1H, hept, *J* 7.0, C*H*(CH_3)$_2$), 3.79 (3H, s, OCH_3), 3.87 (1H, ddd, *J* 11.8, *J* 8.1, *J* 5.0, ArC(4)-OCH^AH^B), 4.05-4.14 (2H, m, ArC(4)-OCH^AH^B, ArC(1)-OCH^AH^B), 4.29 (1H, ddd, *J* 11.7, *J* 5.8, *J* 4.3, ArC(1)-OCH^AH^B), 6.77 (1H, s, ArC(3)*H*), 6.96 (1H, s, ArC(6)*H*), 6.99 (1H, dd, *J* 8.5, *J* 1.1, ArC(5)-Ar(3)*H*), 7.03 (1H, dd, *J* 7.5, *J* 1.1, ArC(5)-Ar(5)*H*), 7.27-7.32 (1H, m, ArC(5)-ArC(6)*H*), 7.34 (1H, ddd, *J* 8.2, *J* 7.4, *J* 1.8, ArC(5)-Ar(4)*H*); **^{13}C{1H} NMR** (126 MHz, $CDCl_3$) δ_C: 19.0 (CH(CH_3)^A(CH_3)^B), 19.3 (CH(CH_3)^A(CH_3)^B), 23.6 (CH_2), 23.8 (CH_2), 26.9 (CH_2), 27.0 (CH_2), 27.5 (CH_2), 27.6 (CH_2), 27.7 (CH_2), 27.9 (CH_2), 28.5 (CH_2), 28.5 (CH_2), 34.1 (*C*H(CH_3)$_2$), 55.5 (O*C*H_3), 68.8 (ArC(1)-OCH_2), 69.0 (ArC(4)-OCH_2), 111.0 (ArC(5)-Ar*C*(3)H), 111.3 (Ar*C*(3)H), 119.7 (ArC(6)H), 120.1 (ArC(5)-Ar*C*(5)H), 126.7 (ArC(5)), 127.4 (ArC(5)-Ar*C*(1)), 128.6 (ArC(5)-Ar*C*(4)H), 132.0 (ArC(5)-ArC(6)H), 140.2 (ArC(2)), 142.7 (ArC(1)), 149.4 (Ar*C*(4)), 156.9 (ArC(5)-Ar*C*(2)), 175.0 (*C*=O); HRMS (APCI$^+$) $C_{29}H_{40}O_5$ ([M+Na]$^+$) requires 491.27680, found 491.2763 (–1.09 ppm).

<Chromatogram>

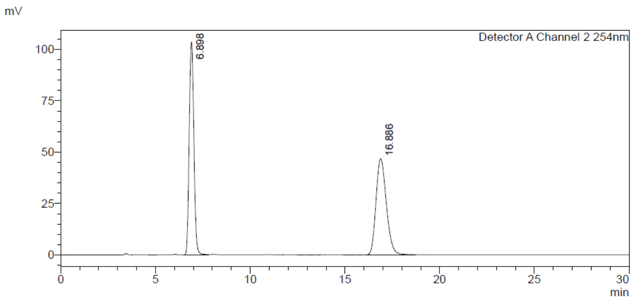

<Peak Table>

Detector A Channel 2 254nm

Peak#	Ret. Time	Area%
1	6.898	49.974
2	16.886	50.026
Total		100.000

<Chromatogram>

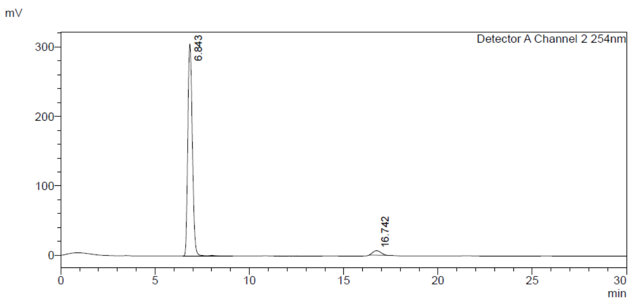

<Peak Table>

Detector A Channel 2 254nm

Peak#	Ret. Time	Area%
1	6.843	96.327
2	16.742	3.673
Total		100.000

(*Rp*)-1^5-(naphthalen-1-yl)-2,15-dioxa-1(1,4)-benzenacyclopentadecaphane-1^2-yl isobutyrate 17

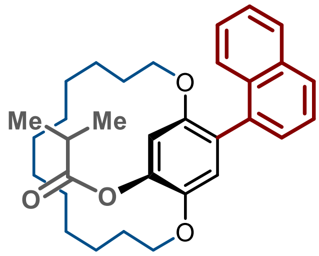

Following General Procedure F with N_2 atmosphere and purification of the residue by silica gel chromatography column using 10:1 petrol ether/EtOAc as eluent gave the product **17** as a white solid (yield 90%, 97:3 er)(without N_2 atmosphere: yield 67%, 95:5 er); mp 54–58 °C (EtOAc/Hexane); $[\alpha]_D^{20}$ –37.5 (c 0.6 in $CHCl_3$); chiral HPLC analysis, ChiralPak ID-H (3% *i*-PrOH : hexane, flow rate 1 mLmin^{-1}, 254 nm, 30 °C), tR major: 5.5 min, tR minor: 9.4 min, 97:3 er; ν_{max} (film)/cm^{-1} 2920 (C–H), 2853 (C–H), 1759 (C=O), 1501 (C=C), 1194 (C–O), 1125 (C–O); **major diastereomer** (NMR time scale 60:40 dr, could not found by HPLC): **^{1}H NMR** (500 MHz, $CDCl_3$) δ_H: 0.98-1.39 (10.2H, m, 8 × C*H*$_2$, C*H*^{A}H^B), 1.41 (1.8H, d, *J* 7.0, CH(C*H*$_3$)^A(CH$_3$)^B), 1.43 (1.8H, d, *J* 7.1, CH(CH$_3$)^A(C*H*$_3$)^B), 1.47-1.55 (0.6H, m, CH^A*H*^{B}), 1.63-1.77 (0.6H, m, C*H*^{A}H^B), 1.78-1.89 (0.6H, m, CH^A*H*^{B}), 2.93 (0.6H, hept, *J* 7.0, C*H*(CH$_3$)$_2$), 3.75-3.83 (0.6H, m, ArC(4)-OC*H*^{A}H^B), 4.02-4.17 (1.2H, m, ArC(4)-OCH^A*H*^{B}, ArC(1)-OC*H*^{A}H^B), 4.27-4.32 (0.6H, m, ArC(1)-OCH^A*H*^{B}), 6.85 (0.6H, s, ArC(3)*H*), 6.99 (0.6H, s, ArC(6)*H*), 7.40-7.59 (2.4H, m, ArC(5)-ArC(4,5,6,7)*H*), 7.71 (0.6H, d, *J* 8.4, ArC(5)-ArC(8)*H*), 7.85-7.92 (1.2H, m, ArC(5)-ArC(2,3)*H*); **^{13}C{1H} NMR** (126 MHz, $CDCl_3$) δ_C: 19.0 (CH(*C*H$_3$)^A(CH$_3$)^B), 19.3 (CH(CH$_3$)^A(*C*H$_3$)^B), 23.8 (2 × *C*H$_2$), 26.8 (*C*H$_2$), 27.4 (*C*H$_2$), 27.6 (*C*H$_2$), 27.7 (*C*H$_2$), 27.9 (*C*H$_2$), 28.1 (*C*H$_2$), 28.6 (*C*H$_2$), 28.7 (*C*H$_2$), 34.1 (*C*H(CH$_3$)$_2$), 68.4 (ArC(4)-O*C*H$_2$), 68.9 (ArC(1)-O*C*H$_2$), 110.7 (Ar*C*(3)H), 119.6 (Ar*C*(6)H), 125.1 (ArC(5)-Ar*C*(6)H), 125.8 (ArC(5)-Ar*C*(3)H), 125.9 (ArC(5)-Ar*C*(7)H), 126.9 (ArC(5)-Ar*C*(8)H), 126.9 (ArC(5)-Ar*C*(2)H), 127.8 (ArC(5)-Ar*C*(4)H), 127.8 (ArC(5)-Ar*C*(5)H), 128.5 (ArC(5)), 132.2 (ArC(5)-Ar*C*(8a)), 133.3 (ArC(5)-Ar*C*(1)), 137.1 (ArC(5)-Ar*C*(5a)), 140.9 (ArC(2)), 143.4 (Ar*C*(1)), 149.4 (Ar*C*(4)), 175.0 (*C*=O); **^{1}H NOESY** (500

MHz, $CDCl_3$) irradiated 6.85 ppm (ArC(3)*H*) same phase response for 6.91 ppm; HRMS (APCI$^+$) $C_{32}H_{40}O_4$ ([M+Na]$^+$) requires 511.28188, found 511.2817 (–0.35 ppm).

Minor diastereomer (NMR time scale 60:40 dr, could not found by HPLC): **^{1}H NMR** (500 MHz, $CDCl_3$) δ_H: 0.98-1.39 (6.8H, m, 8 × C*H*$_2$, C*H*^{A}H^B), 1.40 (1.2H, d, *J* 6.9, CH(C*H*$_3$)^A(CH$_3$)^B), 1.43 (1.2H, d, *J* 7.1, CH(CH$_3$)^A(C*H*$_3$)^B), 1.47-1.55 (0.4H, m, CH^A*H*^{B}), 1.63-1.77 (0.4H, m, C*H*^{A}H^B), 1.78-1.89 (0.4H, m, CH^A*H*^{B}), 2.93 (0.4H, hept, *J* 7.0, C*H*(CH$_3$)$_2$), 3.69-3.75 (0.4H, m, ArC(4)-OC*H*^{A}H^B), 4.02-4.17 (0.8H, m, ArC(4)-OCH^A*H*^{B}, ArC(1)-OC*H*^{A}H^B), 4.23-4.27 (0.4H, m, ArC(1)-OCH^A*H*^{B}), 6.91 (0.4H, s, ArC(3)*H*), 6.98 (0.4H, s, ArC(6)*H*), 7.40-7.59 (1.6H, m, ArC(5)-ArC(4,5,6,7)*H*), 7.85-7.92 (0.8H, m, ArC(5)-ArC(2,3)*H*), 7.95 (0.4H, d, *J* 8.3, ArC(5)-ArC(8)*H*); **^{13}C{1H} NMR** (126 MHz, $CDCl_3$) δ_C: 19.0 (CH(*C*H$_3$)^A(CH$_3$)^B), 19.3 (CH(CH$_3$)^A(*C*H$_3$)^B), 23.8 (*C*H$_2$), 24.0 (*C*H$_2$), 27.3 (*C*H$_2$), 27.4 (*C*H$_2$), 27.5 (*C*H$_2$), 27.8 (*C*H$_2$), 27.9 (*C*H$_2$), 28.2 (*C*H$_2$), 28.6 (*C*H$_2$), 28.6 (*C*H$_2$), 34.1 (*C*H(CH$_3$)$_2$), 68.9 (ArC(4)-O*C*H$_2$), 69.3 (ArC(1)-O*C*H$_2$), 111.9 (Ar*C*(3)H), 120.4 (Ar*C*(6)H), 125.4 (ArC(5)-Ar*C*(6)H), 125.5 (ArC(5)-Ar*C*(3)H), 125.6 (ArC(5)-Ar*C*(7)H), 126.0 (ArC(5)-Ar*C*(8)H), 127.5 (ArC(5)-Ar*C*(2)H), 128.1 (Ar*C*(5)), 128.3 (ArC(5)-Ar*C*(4)H), 128.4 (ArC(5)-Ar*C*(5)H), 132.1 (ArC(5)-Ar*C*(8a)), 133.9 (ArC(5)-Ar*C*(1)), 135.8 (ArC(5)-Ar*C*(5a)), 140.7 (Ar*C*(2)), 142.9 (Ar*C*(1)), 150.1 (Ar*C*(4)), 175.0 (*C*=O).

<Chromatogram>

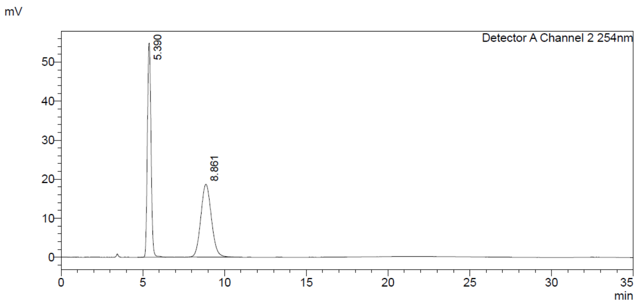

<Peak Table>

Detector A Channel 2 254nm

Peak#	Ret. Time	Area%
1	5.390	50.136
2	8.861	49.864
Total		100.000

<Chromatogram>

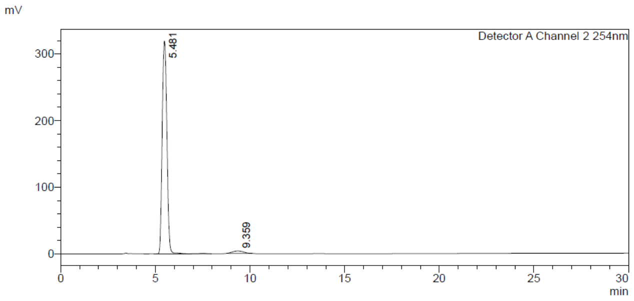

<Peak Table>

Detector A Channel 2 254nm

Peak#	Ret. Time	Area%
1	5.481	97.321
2	9.359	2.679
Total		100.000

(*Rp*)-1^5-(naphthalen-2-yl)-2,15-dioxa-1(1,4)-benzenacyclopentadecaphane-1^2-yl isobutyrate 18

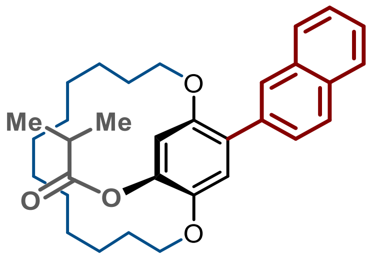

Z

Following General Procedure F and purification of the residue by silica gel chromatography column using 10:1 petrol ether/EtOAc as eluent gave the product **18** as a colourless oil (yield 88%, 96.5:3.5 er); $[\alpha]_D^{20}$ –28.7

(c 0.3 in $CHCl_3$); chiral HPLC analysis, ChiralPak ID-H (3% *i*-PrOH : hexane, flow rate 1 $mLmin^{-1}$, 254 nm, 30 °C), tR major: 7.5 min, tR minor: 16.4 min, 96.5:3.5 er; ν_{max} (film)/cm^{-1} 2924 (C–H), 2853 (C–H), 1759 (C=O), 1499 (C=C), 1182 (C–O), 1121 (C–O); **^{1}H NMR** (500 MHz, $CDCl_3$) δ_H: 1.02-1.38 (15H, m, 7 × CH_2, CH^AH^B), 1.40 (3H, d, *J* 7.0, $CH(CH_3)^A(CH_3)^B$), 1.43 (3H, d, *J* 7.1, $CH(CH_3)^A(CH_3)^B$), 1.44-1.51 (2H, m, CH_2), 1.59-1.68 (1H, m, CH^AH^B), 1.68-1.79 (1H, m, CH^AH^B), 1.79-1.90 (1H, m, CH^AH^B), 2.91 (1H, hept, *J* 7.0, $CH(CH_3)_2$), 3.98 (1H, ddd, *J* 12.2, *J* 8.3, *J* 4.4, ArC(4)-OCH^AH^B), 4.10-4.25 (2H, m, ArC(1)-OCH^AH^B, ArC(4)-OCH^AH^B), 4.36 (1H, ddd, *J* 11.2, *J* 6.4, *J* 4.4, ArC(1)-OCH^AH^B), 6.85 (1H, s, ArC(3)*H*), 7.14 (1H, s, ArC(6)*H*), 7.48-7.57 (2.4H, m, ArC(5)-ArC(6,7)*H*), 7.73 (1H, dd, *J* 8.4, *J* 1.8, ArC(5)-ArC(3)*H*), 7.87-7.96 (3H, m, ArC(5)-ArC(4,5,8)*H*), 7.96-8.03 (1H, m, ArC(5)-ArC(1)*H*); **^{13}C{1H} NMR** (126 MHz, $CDCl_3$) δ_C: 19.0 ($CH(CH_3)^A(CH_3)^B$), 19.3 ($CH(CH_3)^A(CH_3)^B$), 23.8 (CH_2), 24.0 (CH_2), 27.0 (CH_2), 27.5 (CH_2), 27.5 (CH_2), 27.6 (CH_2), 27.9 (CH_2), 28.1 (CH_2), 28.5 (CH_2), 28.6 (CH_2), 34.1 ($CH(CH_3)_2$), 69.0 (ArC(4)-OCH_2), 69.2 (ArC(1)-OCH_2), 111.5 (Ar*C*(3)H), 119.1 (ArC(6)H), 125.8 (ArC(5)-Ar*C*(7)H), 126.0 (ArC(5)-Ar*C*(6)H), 127.3 (ArC(5)-Ar*C*(8)H), 127.6 (ArC(5)-Ar*C*(5)H), 128.0 (ArC(5)-Ar*C*(3)H), 128.1 (ArC(5)-Ar*C*(1)H), 128.2 (ArC(5)-Ar*C*(4)H), 129.9 (ArC(5)), 132.5 (ArC(5)-Ar*C*(5a)), 133.4 (ArC(5)-Ar*C*(8a)), 136.1 (ArC(5)-Ar*C*(2)), 140.7 (Ar*C*(2)), 143.8 (Ar*C*(1)), 149.3 (Ar*C*(4)), 175.0 (*C*=O); HRMS (APCI$^+$) $C_{32}H_{40}O_4$ ($[M+Na]^+$) requires 511.28188, found 511.2818 (–0.17 ppm).

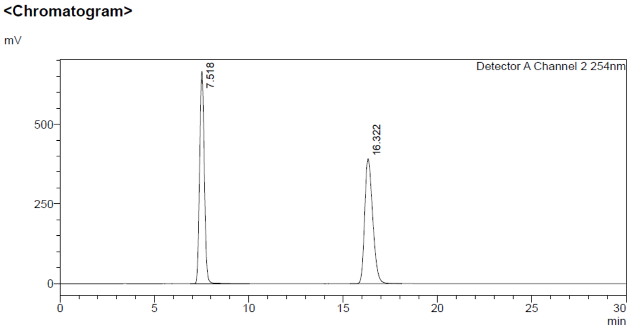

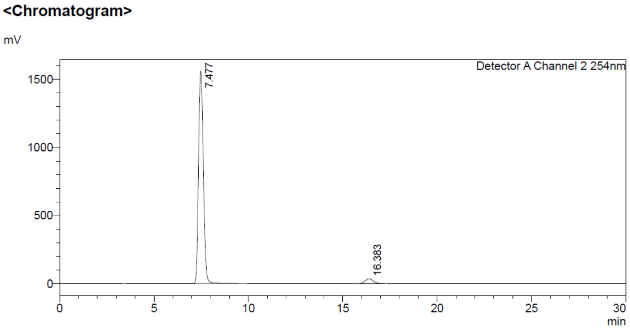

<Peak Table>

Detector A Channel 2 254nm

Peak#	Ret. Time	Area%
1	7.518	49.960
2	16.322	50.040
Total		100.000

<Peak Table>

Detector A Channel 2 254nm

Peak#	Ret. Time	Area%
1	7.477	96.446
2	16.383	3.554
Total		100.000

(*Rp*)-1^5-(pyridin-3-yl)-2,15-dioxa-1(1,4)-benzenacyclopentadecaphane-1^2-yl isobutyrate 19

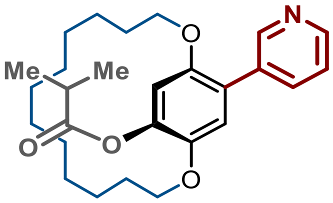

Following General Procedure F and purification of the residue by silica gel chromatography column using 3:1 petrol ether/EtOAc as eluent gave the product **19** as a yellow oil (yield 81%, 96.5:3.5 er); $[\alpha]_D^{20}$ –39.0 (c 0.3 in $CHCl_3$); chiral HPLC analysis, ChiralPak IB-H (5% *i*-PrOH : hexane, flow rate 1 $mLmin^{-1}$, 254 nm, 30 °C),

tR major: 8.7 min, tR minor: 10.6 min, 96.5:3.5 er; ν_{max} (film)/cm^{-1} 2926 (C–H), 2855 (C–H), 1761 (C=O), 1506 (C=C), 1200 (C–O), 1128 (C–O); **^{1}H NMR** (500 MHz, $CDCl_3$) δ_H: 0.93-1.34 (16H, m, 8 × C*H*$_2$), 1.36 (3H, d, *J* 7.0, CH(C*H*$_3$)^A(CH$_3$)^B), 1.39 (3H, d, *J* 7.1, CH(CH$_3$)^A(C*H*$_3$)^B), 1.41-1.48 (1H, m, C*H*^{A}H^B), 1.52-1.63 (1H, m, CH^A*H*^{B}), 1.68-1.85 (2H, m, C*H*$_2$), 2.88 (1H, hept, *J* 7.0, C*H*(CH$_3$)$_2$), 4.03 (1H, ddd, *J* 12.2, *J* 8.4, *J* 4.1, ArC(4)-OC*H*^{A}H^B), 4.16 (1H, ddd, *J* 12.1, *J* 8.0, *J* 4.4, ArC(1)-OC*H*^{A}H^B), 4.22 (1H, ddd, *J* 10.8, *J* 6.0, *J* 4.3, ArC(4)-OCH^A*H*^{B}), 4.32 (1H, ddd, *J* 11.1, *J* 6.2, *J* 4.4, ArC(1)-OCH^A*H*^{B}), 6.81 (1H, s, ArC(3)*H*), 7.02 (1H, s, ArC(6)*H*), 7.35 (1H, dd, *J* 7.9, *J* 4.8, ArC(5)-ArC(4)*H*), 7.88 (1H, dd, *J* 7.9, *J* 2.0, ArC(5)-ArC(5)*H*), 8.58 (1H, d, *J* 3.3, ArC(5)-ArC(6)*H*), 8.77 (1H, d, *J* 2.2, ArC(5)-ArC(2)*H*); **^{13}C{1H} NMR** (126 MHz, $CDCl_3$) δ_C: 18.9 (CH(CH$_3$)^A(CH$_3$)^B), 19.2 (CH(CH$_3$)^A(CH$_3$)^B), 23.7 (CH_2), 23.9 (CH_2), 26.7 (CH_2), 27.4 (CH_2), 27.4 (CH_2), 27.6 (CH_2), 27.8 (CH_2), 28.0 (CH_2), 28.5 (CH_2), 28.5 (CH_2), 34.1 (CH(CH$_3$)$_2$), 68.8 (ArC(1)-OCH$_2$), 69.2 (ArC(4)-OCH$_2$), 110.9 (Ar*C*(3)H), 118.5 (Ar*C*(6)H), 122.9 (ArC(5)-Ar*C*(5)H), 125.9 (Ar*C*(5)), 134.0 (ArC(5)-Ar*C*(3)), 137.0 (ArC(5)-Ar*C*(4)H), 141.4 (Ar*C*(2)), 143.8 (Ar*C*(1)), 148.1 (ArC(5)-Ar*C*(6)H), 149.2 (Ar*C*(4)), 150.1 (ArC(5)-Ar*C*(2)H), 174.9 (*C*=O); HRMS (APCI$^+$) $C_{27}H_{38}O_4N$ ([M+Na]$^+$) requires 440.27954, found 440.2801 (+1.34 ppm).

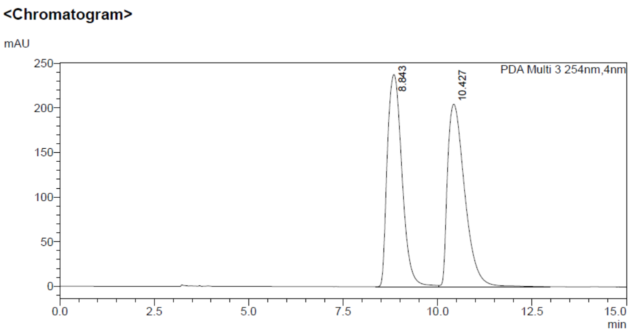

<Peak Table>

PDA Ch3 254nm

Peak#	Ret. Time	Area%
1	8.843	49.799
2	10.427	50.201
Total		100.000

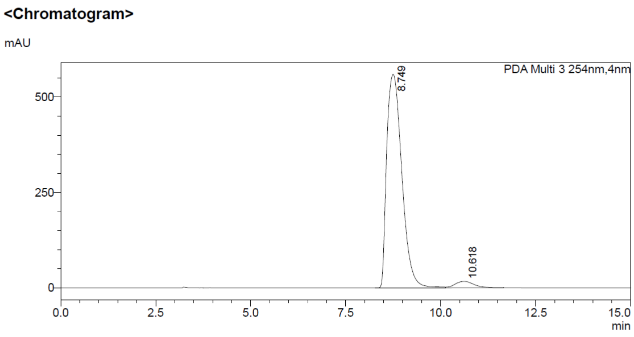

<Peak Table>

PDA Ch3 254nm

Peak#	Ret. Time	Area%
1	8.749	96.500
2	10.618	3.500
Total		100.000

(*Rp*)-1^5-(furan-2-yl)-2,15-dioxa-1(1,4)-benzenacyclopentadecaphane-1^2-yl isobutyrate 20

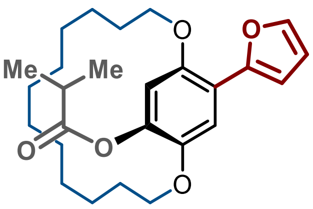

Following General Procedure F and purification of the residue by silica gel chromatography column using 10:1 petrol ether/EtOAc as eluent gave the product **20** as a white solid (yield 93%, 95.5:4.5 er); mp 62–65 °C (EtOAc/Hexane); $[\alpha]_D^{20}$ –13.8 (c 0.6 in $CHCl_3$); chiral HPLC analysis, ChiralPak ID-H (3% *i*-PrOH : hexane, flow rate 1 mLmin^{-1}, 254 nm, 30 °C), tR major: 9.3 min, tR minor: 31.4 min, 95.5:4.5 er; ν_{max} (film)/cm^{-1} 2922

(C–H), 2853 (C–H), 1757 (C=O), 1510 (C=C), 1495 (C=C), 1194 (C–O), 1126 (C–O), 1043 (C–O); ^{1}**H NMR** (500 MHz, $CDCl_3$) δ_H: 0.82-0.99 (9H, m, 4 × C*H*$_2$, C*H*^{A}H^B), 1.09-1.22 (5H, m, 2 ×C*H*$_2$, CH^A*H*^{B}), 1.30-1.33 (2H, m, C*H*$_2$), 1.36 (3H, d, *J* 7.0, CH(C*H*$_3$)^A(CH$_3$)^B), 1.38 (3H, d, *J* 7.1, CH(CH$_3$)^A(C*H*$_3$)^B), 1.52-1.60 (2H, m, C*H*$_2$), 1.74-1.82 (1H, m, C*H*^{A}H^B), 1.83-1.92 (1H, m, CH^A*H*^{B}), 2.87 (1H, hept, *J* 7.0, C*H*(CH$_3$)$_2$), 4.16 (1H, ddd, *J* 12.0, *J* 8.0, *J* 4.4, ArC(1)-OC*H*^{A}H^B), 4.24 (1H, ddd, *J* 12.2, *J* 8.7, *J* 4.0, ArC(4)-OC*H*^{A}H^B), 4.33-4.45 (2H, m, ArC(1)-OCH^A*H*^{B}, ArC(4)-OCH^A*H*^{B}), 6.51 (1H, dd, *J* 3.3, *J* 1.8, ArC(5)-ArC(4)*H*), 6.76 (1H, s, ArC(3)*H*), 6.98 (1H, d, *J* 3.3, ArC(5)-ArC(3)*H*), 7.47 (1H, d, *J* 1.8, ArC(5)-ArC(5)*H*), 7.54 (1H, s, ArC(6)*H*); ^{13}**C{1H} NMR** (126 MHz, $CDCl_3$) δ_C: 18.9 (CH(*C*H$_3$)^A(CH$_3$)^B), 19.2 (CH(CH$_3$)^A(*C*H$_3$)^B), 23.8 (*C*H$_2$), 24.0 (*C*H$_2$), 26.8 (*C*H$_2$), 27.4 (*C*H$_2$), 27.5 (*C*H$_2$), 27.5 (*C*H$_2$), 27.8 (*C*H$_2$), 27.9 (*C*H$_2$), 28.4 (2 × *C*H$_2$), 34.1 (*C*H(CH$_3$)$_2$), 68.5 (ArC(1)-O*C*H$_2$), 69.1 (ArC(4)-O*C*H$_2$), 109.8 (ArC(5)-Ar*C*(3)H), 110.1 (Ar*C*(3)H), 111.8 (ArC(5)-Ar*C*(4)H), 113.8 (Ar*C*(6)H), 118.9 (Ar*C*(5)), 140.3 (Ar*C*(2)), 140.9 (ArC(5)-Ar*C*(5)H), 143.5 (Ar*C*(1)), 147.8 (Ar*C*(4)), 150.0 (ArC(5)-Ar*C*(2)), 174.9 (*C*=O); HRMS (APCI$^+$) $C_{26}H_{36}O_5$ ([M+Na]$^+$) requires 451.24550, found 451.2451 (–0.97 ppm).

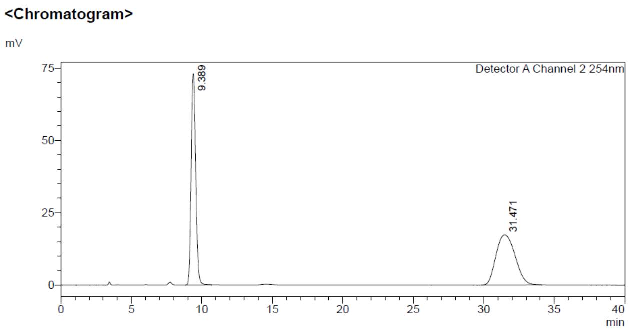

<Peak Table>

Detector A Channel 2 254nm

Peak#	Ret. Time	Area%
1	9.389	50.168
2	31.471	49.832
Total		100.000

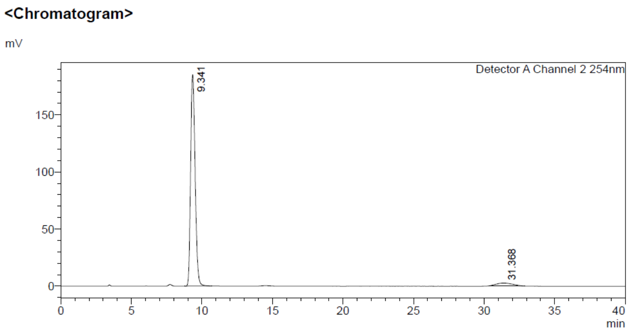

<Peak Table>

Detector A Channel 2 254nm

Peak#	Ret. Time	Area%
1	9.341	95.530
2	31.368	4.470
Total		100.000

(*Rp*)-1^5-(thiophen-3-yl)-2,15-dioxa-1(1,4)-benzenacyclopentadecaphane-1^2-yl isobutyrate 21

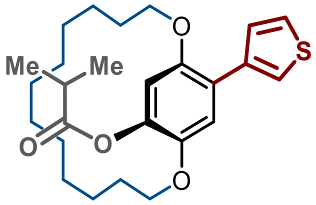

Following General Procedure F and purification of the residue by silica gel chromatography column using 10:1 petrol ether/EtOAc as eluent gave the product **21** as a yellow oil (yield 89%, 97:3 er); $[\alpha]_D^{20}$ –29.4 (c 0.5 in $CHCl_3$); chiral HPLC analysis, ChiralPak ID-H (3% *i*-PrOH : hexane, flow rate 1 mLmin^{-1}, 254 nm, 30 °C), tR major: 8.7 min, tR minor: 32.8 min, 97:3 er; ν_{max} (film)/cm^{-1} 2924 (C–H), 2853 (C–H), 1759 (C=O), 1495 (C=C), 1194 (C–O), 1123 (C–O); ^{1}**H NMR** (500 MHz, $CDCl_3$) δ_H: 0.93-1.34 (16H, m, 8 × C*H*$_2$), 1.36 (3H, d, *J* 7.0,

CH(CH_3)^A(CH$_3$)^B), 1.39 (3H, d, *J* 7.0, CH(CH$_3$)^A(CH_3)^B), 1.47-1.61 (2H, m, CH_2), 1.71-1.85 (2H, m, CH_2), 2.88 (1H, hept, *J* 7.0, C*H*(CH$_3$)$_2$), 4.07-4.19 (2H, m, ArC(4)-OCH^AH^B, ArC(1)-OCH^AH^B), 4.23-4.36 (2H, m, ArC(4)-OCH^AH^B, ArC(1)-OCH^AH^B), 6.78 (1H, s, ArC(3)*H*), 7.17 (1H, s, ArC(6)*H*), 7.36 (1H, dd, *J* 5.0, *J* 3.0, ArC(5)-ArC(2)*H*), 7.43 (1H, d, *J* 5.0, *J* 1.3, ArC(5)-ArC(4)*H*), 7.60 (1H, d, *J* 3.0, *J* 1.3, ArC(5)-ArC(5)*H*); **^{13}C{1H} NMR** (126 MHz, CDCl$_3$) δ_C: 19.0 (CH(*C*H$_3$)^A(CH$_3$)^B), 19.2 (CH(CH$_3$)^A(*C*H$_3$)^B), 23.8 (*C*H$_2$), 24.0 (*C*H$_2$), 26.9 (*C*H$_2$), 27.5 (*C*H$_2$), 27.5 (2 × *C*H$_2$), 27.8 (*C*H$_2$), 28.0 (*C*H$_2$), 28.4 (*C*H$_2$), 28.5 (*C*H$_2$), 34.1 (*C*H(CH$_3$)$_2$), 68.7 (ArC(1)-O*C*H$_2$), 69.3 (ArC(4)-O*C*H$_2$), 110.8 (Ar*C*(3)H), 118.0 (Ar*C*(6)H), 123.1 (ArC(5)-Ar*C*(2)H), 124.2 (Ar*C*(5)), 124.4 (ArC(5)-Ar*C*(4)H), 128.5 (ArC(5)-Ar*C*(5)H), 138.1 (ArC(5)-Ar*C*(3)), 140.5 (Ar*C*(2)), 143.5 (Ar*C*(1)), 149.1 (Ar*C*(4)), 174.9 (*C*=O); HRMS (APCI$^+$) $C_{26}H_{36}O_4S$ ([M+Na]$^+$) requires 467.22265, found 467.2222 (–0.92 ppm).

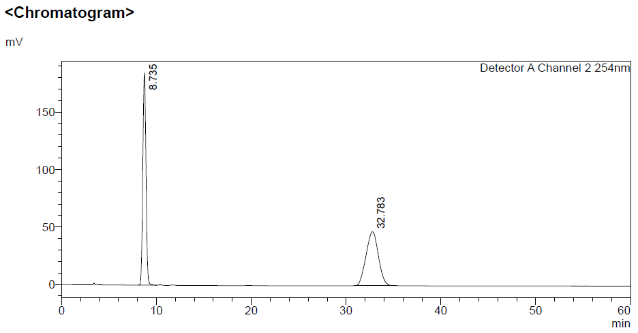

<Peak Table>

Detector A Channel 2 254nm

Peak#	Ret. Time	Area%
1	8.735	50.148
2	32.783	49.852
Total		100.000

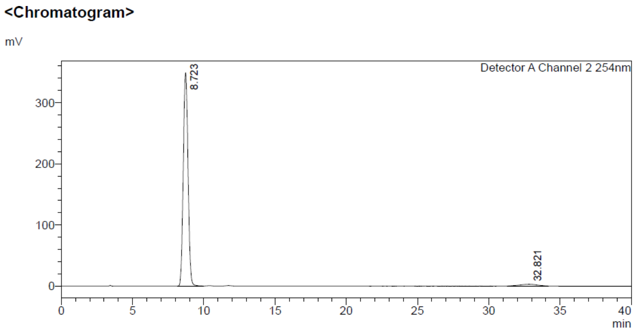

<Peak Table>

Detector A Channel 2 254nm

Peak#	Ret. Time	Area%
1	8.723	96.924
2	32.821	3.076
Total		100.000

(*Rp*)-*tert*-butyl 3-(1^5-(isobutyryloxy)-2,15-dioxa-1(1,4)-benzenacyclopentadecaphane-1^2-yl)-5-methoxy-1H-indole-1-carboxylate 22

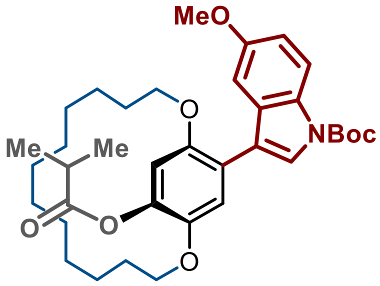

Following General Procedure F and purification of the residue by silica gel chromatography column using 4:1 petrol ether/EtOAc as eluent gave the product **22** as a colourless oil (yield 89%, 85:15 er); $[\alpha]_D^{20}$ +8.8 (c 0.4 in CHCl$_3$); chiral HPLC analysis, ChiralPak ID-H (3% *i*-PrOH : hexane, flow rate 1 mLmin^{-1}, 254 nm, 30 °C), tR major: 6.4 min, tR minor: 7.9 min, 85:15 er; ν_{max} (film)/cm^{-1} 2926 (C–H), 2855 (C–H), 1761 (C=O), 1728 (C=O), 1468 (C=C), 1125 (C–O); **^{1}H NMR** (500 MHz, CDCl$_3$) δ_H: 0.97-1.33 (16H, m, 8 × CH_2), 1.35 (9H, s, C(CH_3)$_3$), 1.37 (4H, d, *J* 7.1, CH(CH_3)^A(CH$_3$)^B, CH^AH^B), 1.40 (3H, d, *J* 7.1, CH(CH$_3$)^A(CH_3)^B), 1.54-1.62 (1H, m, CH^AH^B), 1.63-

1.73 (1H, m, CH^AH^B), 1.78-1.89 (1H, m, , CH^AH^B), 2.88 (1H, hept, *J* 7.0, C*H*(CH_3)$_2$), 3.90 (3H, s, OCH_3), 3.94 (1H, ddd, *J* 12.0, *J* 8.4, *J* 4.2, ArC(4)-OCH^AH^B), 4.10-4.21 (2H, m, ArC(4)-OCH^A*H*^{B}, ArC(1)-OCH^AH^B), 4.32 (1H, ddd, *J* 11.1, *J* 6.3, *J* 4.4, ArC(1)-OCH^A*H*^{B}), 6.42 (1H, s, indoleC(7)*H*), 6.71 (1H, s, ArC(3)*H*), 6.96 (1H, dd, *J* 9.1, *J* 2.6, indoleC(4)*H*), 7.07 (1H, s, ArC(6)*H*), 7.07 (1H, d, *J* 2.5, indoleC(6)*H*), 8.15 (1H, d, *J* 9.0, indoleC(2)*H*); **^{13}C{1H} NMR** (126 MHz, $CDCl_3$) δ_C: 19.0 (CH(CH_3)^A(CH_3)^B), 19.3 (CH(CH_3)^A(CH_3)^B)P, 23.7 (CH_2), 23.7 (CH_2), 26.8 (CH_2), 27.4 (CH_2), 27.4 (CH_2), 27.5 (C(CH_3)$_3$), 27.6 (CH_2), 27.9 (CH_2), 28.0 (CH_2), 28.5 (CH_2), 28.6 (CH_2), 34.1 (CH(CH_3)$_2$), 55.7 (*C*(CH_3)), 68.3 (ArC(1)-OCH_2), 69.0 (ArC(4)-OCH_2), 83.1 (OCH_3), 102.8 (indole*C*(6)H), 108.9 (indole*C*(7)H), 110.2 (Ar*C*(3)H), 112.8 (indole*C*(4)H), 115.7 (indole*C*(2)H), 118.0 (Ar*C*(6)H), 123.7 (Ar*C*(5)), 129.6 (indole*C*(7a)), 132.0 (indole*C*(4a)), 137.5 (indole*C*(3)), 141.3 (Ar*C*(2)), 143.1 (Ar*C*(1)), 149.6 (Ar*C*(4)), 150.2 (*C*=O), 155.6 (indole*C*(5)), 174.9 (*C*=O); HRMS (APCI$^+$) $C_{36}H_{49}O_7N$ ([M+Na]$^+$) requires 630.34012, found 630.3398 (–0.58 ppm).

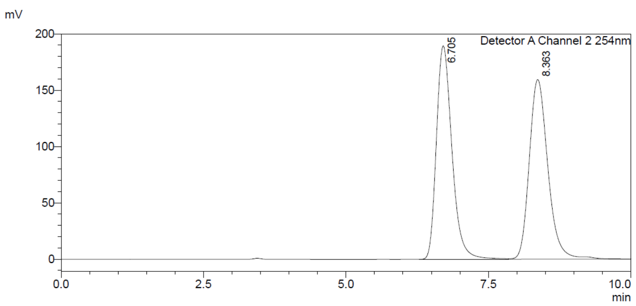

<Peak Table>

Detector A Channel 2 254nm

Peak#	Ret. Time	Area%
1	6.705	49.856
2	8.363	50.144
Total		100.000

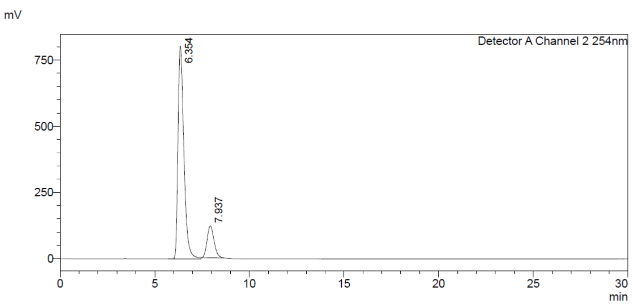

<Peak Table>

Detector A Channel 2 254nm

Peak#	Ret. Time	Area%
1	6.354	84.917
2	7.937	15.083
Total		100.000

(*Rp*)-1^5-(pyren-1-yl)-2,15-dioxa-1(1,4)-benzenacyclopentadecaphane-1^2-yl isobutyrate 23

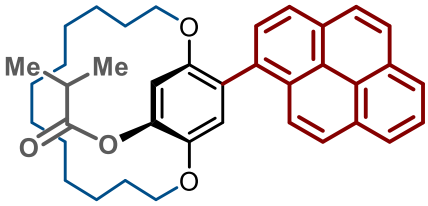

Following General Procedure F and purification of the residue by silica gel chromatography column using 10:1 petrol ether/EtOAc as eluent gave the product **23** as a colourless oil (yield 93%, 97:3 er); $[\alpha]_D^{20}$ +17.6 (c 0.3 in $CHCl_3$); chiral HPLC analysis, ChiralPak ID-H (3% *i*-PrOH : hexane, flow rate 1 mLmin^{-1}, 254 nm, 30 °C), tR major: 7.8 min, tR minor: 14.6 min, 97:3 er; ν_{max} (film)/cm^{-1} 2924 (C–H), 2853 (C–H), 1757 (C=O), 1495 (C=C), 1196 (C–O), 1128 (C–O); **major diastereomer** (NMR time scale 55:45 dr, could not found by HPLC): **^{1}H NMR** (500 MHz, $CDCl_3$) δ_H: 1.02-1.41 (9.6H, m, 8 × CH_2), 1.43 (1.8H, d, *J* 7.0, CH(CH_3)^A(CH_3)^B), 1.46 (1.8H,

d, *J* 7.0, $CH(CH_3)^A(CH_3)^B$), 1.48-1.58 (0.6H, m, CH^AH^B), 1.59-1.73 (1.2H, m, CH_2), 1.83-2.00 (0.6H, m, CH^AH^B), 2.96 (0.6H, hept, *J* 7.1, $CH(CH_3)_2$), 3.65-3.81 (0.6H, m, ArC(4)-OCH^AH^B), 4.07 (0.6H, dq, *J* 11.4, *J* 5.7, *J* 5.5, ArC(4)-OCH^AH^B), 4.16 (0.6H, dddd, *J* 11.8, *J* 8.2, *J* 6.5, *J* 3.8, ArC(1)-OCH^AH^B), 4.32 (0.6H, ddt, *J* 14.2, *J* 11.4, *J* 4.6, ArC(1)-OCH^AH^B), 6.93 (0.6H, s, ArC(3)*H*), 7.12 (0.6H, s, ArC(6)*H*), 7.91-8.31 (5.4H, m, 9 × ArC(5)-ArC*H*); **^{13}C{1H} NMR** (126 MHz, $CDCl_3$) δ_C: 19.0 ($CH(CH_3)^A(CH_3)^B$), 19.3 ($CH(CH_3)^A(CH_3)^B$), 23.8 (2 × CH_2), 26.8 (CH_2), 27.4 (CH_2), 27.6 (CH_2), 27.9 (2 × CH_2), 28.2 (CH_2), 28.7 (CH_2), 28.7 (CH_2), 34.2 ($CH(CH_3)_2$), 68.6 (ArC(4)-OCH_2), 69.0 (ArC(1)-OCH_2), 110.9 (ArC(3)H), 120.0 (Ar*C*(6)H), 124.4 (ArC(5)-Ar*C*H), 124.8 (ArC(5)-Ar*C*), 124.9 (ArC(5)-Ar*C*H), 125.0 (ArC(5)-Ar*C*H), 125.9 (ArC(5)-Ar*C*H), 125.9 (ArC(5)-Ar*C*H), 127.2 (ArC(5)-Ar*C*H), 127.4 (ArC(5)-ArCH), 127.4 (ArC(5)-ArCH), 127.6 (ArC(5)-Ar*C*H), 128.6 (ArC(5)-Ar*C*), 129.5 (ArC(5)-Ar*C*), 130.7 (ArC(5)-Ar*C*), 131.2 (ArC(5)-Ar*C*), 131.4 (ArC(5)-Ar*C*), 134.2 (ArC(5)-Ar*C*(1)), 141.0 (Ar*C*(2)), 143.5 (Ar*C*(1)), 149.6 (Ar*C*(4)), 175.1 (*C*=O); **^{1}H NOESY** (500 MHz, $CDCl_3$) irradiated 6.93 ppm (ArC(3)*H*) same phase response for 6.99 ppm; HRMS (APCI$^+$) $C_{38}H_{42}O_4$ ($[M+Na]^+$) requires 585.29753, found 585.2973 (–0.39 ppm).

Minor diastereomer (NMR time scale 55:45 dr, could not found by HPLC): **^{1}H NMR** (500 MHz, $CDCl_3$) δ_H: 1.02-1.41 (6.4H, m, 8 × CH_2), 1.42 (1.2H, d, *J* 6.9, $CH(CH_3)^A(CH_3)^B$), 1.46 (1.2H, d, *J* 7.0, $CH(CH_3)^A(CH_3)^B$), 1.48-1.58 (0.4H, m, CH^AH^B), 1.59-1.73 (0.8H, m, CH_2), 1.83-2.00 (0.4H, m, CH^AH^B), 2.95 (0.4H, hept, *J* 7.0, *J* 6.9, $CH(CH_3)_2$), 3.65-3.81 (0.4H, m, ArC(4)-OCH^AH^B), 4.07 (0.4H, dq, *J* 11.4, *J* 5.7, *J* 5.5, ArC(4)-OCH^AH^B), 4.16 (0.4H, dddd, *J* 11.8, *J* 8.2, *J* 6.5, *J* 3.8, ArC(1)-OCH^AH^B), 4.32 (0.4H, ddt, *J* 14.2, *J* 11.4, *J* 4.6, ArC(1)-OCH^AH^B), 6.99 (0.4H, s, ArC(3)*H*), 7.09 (0.4H, s, ArC(6)*H*), 7.91-8.31 (3.6H, m, 9 × ArC(5)-ArC*H*); **^{13}C{1H} NMR** (126 MHz, $CDCl_3$) δ_C: 19.0 ($CH(CH_3)^A(CH_3)^B$), 19.3 ($CH(CH_3)^A(CH_3)^B$), 23.8 (CH_2), 24.0 (CH_2), 27.2 (CH_2), 27.3 (CH_2), 27.5 (CH_2), 27.7 (CH_2), 27.9 (CH_2), 28.3 (CH_2), 28.6 (CH_2), 28.7 (CH_2), 34.2 ($CH(CH_3)_2$), 69.1 (ArC(4)-OCH_2), 69.3 (ArC(1)-OCH_2), 112.3 (Ar*C*(3)H), 120.8 (Ar*C*(6)H), 124.5 (ArC(5)-Ar*C*H), 124.7 (ArC(5)-Ar*C*), 124.8 (ArC(5)-ArCH), 125.2 (ArC(5)-Ar*C*H), 125.4 (ArC(5)-Ar*C*H), 126.3 (ArC(5)-Ar*C*H), 127.1 (ArC(5)-Ar*C*H), 127.3 (ArC(5)-Ar*C*H), 127.6 (ArC(5)-Ar*C*H), 128.9 (ArC(5)-Ar*C*H), 128.9 (ArC(5)-Ar*C*), 129.5 (ArC(5)-Ar*C*), 130.6 (ArC(5)-Ar*C*), 130.9 (ArC(5)-Ar*C*), 131.4 (ArC(5)-Ar*C*), 133.8 (ArC(5)-Ar*C*(1)), 140.8 (Ar*C*(2)), 143.1 (Ar*C*(1)), 150.1 (Ar*C*(4)), 175.0 (*C*=O).

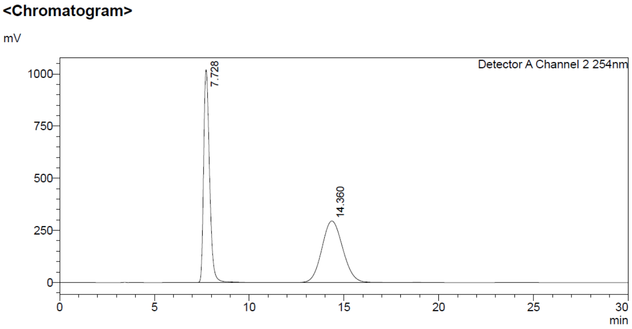

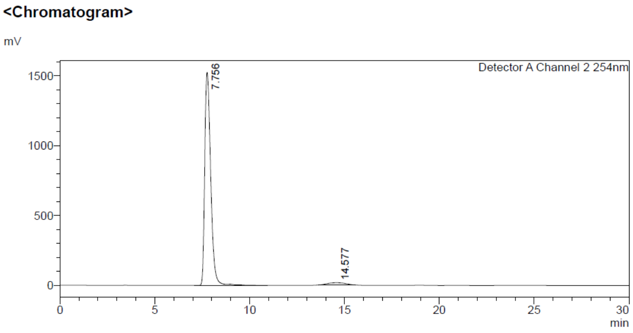

<Peak Table>

Detector A Channel 2 254nm

Peak#	Ret. Time	Area%
1	7.728	49.383
2	14.360	50.617
Total		100.000

<Peak Table>

Detector A Channel 2 254nm

Peak#	Ret. Time	Area%
1	7.756	96.958
2	14.577	3.042
Total		100.000

(*Rp*)-2,15-dioxa-1(1,4)-naphthalenacyclopentadecaphane-1²-yl isobutyrate 24

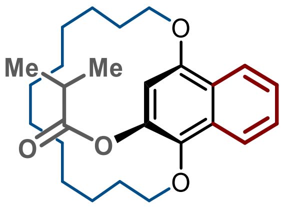

Following General Procedure F and purification of the residue by silica gel chromatography column using 4:1 petrol ether/EtOAc as eluent gave the product **24** as a colourless oil (yield 95%, 98:2 er); $[\alpha]_D^{20}$ –28.3 (c 2.4 in $CHCl_3$); chiral HPLC analysis, ChiralPak ID-H (3% *i*-PrOH : hexane, flow rate 1 mLmin^{-1}, 254 nm, 30 °C), tR major: 8.1 min, tR minor: 15.1 min, 98:2 er; ν_{max} (film)/cm^{-1} 2924 (C–H), 2853 (C–H), 1755 (C=O), 1589 (C=C), 1115 (C–O), 1088 (C–O); **¹H NMR** (500 MHz, $CDCl_3$) δ_H: 0.50-0.73 (4H, m, 2 × C*H*$_2$), 0.78-1.24 (10H, m, 5 × C*H*$_2$), 1.41 (4H, d, *J* 7.0, CH(C*H*$_3$)^A(CH$_3$)^B, C*H*^{A}H^B), 1.43 (3H, d, *J* 7.0, CH(CH$_3$)^A(C*H*$_3$)^B), 1.48-1.68 (3H, m, C*H*$_2$, CH^A*H*^{B}), 1.73-1.97 (2H, m, C*H*$_2$), 2.93 (1H, hept, *J* 7.0, C*H*(CH$_3$)$_2$), 4.29 (2H, t, *J* 6.4, ArC(1)-OCH$_2$), 4.34 (1H, ddd, *J* 11.2, *J* 7.1, *J* 4.1, ArC(4)-OC*H*^{A}H^B), 4.43 (1H, ddd, *J* 11.2, *J* 7.3, *J* 3.8, ArC(4)-OCH^A*H*^{B}), 6.60 (1H, s, ArC(3)*H*), 7.46 (1H, ddd, *J* 8.2, *J* 6.8, *J* 1.3, ArC(6)*H*), 7.52 (1H, ddd, *J* 8.3, *J* 6.8, *J* 1.3, ArC(7)*H*), 8.19 (1H, d, *J* 7.9, ArC(5)*H*), 8.25 (1H, d, *J* 7.8, ArC(8)*H*); **¹³C{1H} NMR** (126 MHz, $CDCl_3$) δ_C: 19.0 (CH(*C*H$_3$)^A(CH$_3$)^B), 19.1 (CH(CH$_3$)^A(*C*H$_3$)^B), 24.3 (*C*H$_2$), 24.3 (*C*H$_2$), 26.8 (*C*H$_2$), 26.9 (*C*H$_2$), 27.4 (2 × CH$_2$), 27.8 (*C*H$_2$), 27.8 (*C*H$_2$), 28.1 (*C*H$_2$), 28.3 (*C*H$_2$), 34.2 (*C*H(CH$_3$)$_2$), 68.1 (ArC(1)-OCH$_2$), 72.8 (ArC(4)-OCH$_2$),103.3 (Ar*C*(3)H), 122.2 (ArC(6)H), 122.6 (Ar*C*(7)H), 125.0 (Ar*C*(5)H), 125.3 (Ar*C*(5a)), 126.4 (Ar*C*(8)H), 130.0 (Ar*C*(8a)), 138.2 (Ar*C*(2)), 138.7 (Ar*C*(1)), 150.4 (Ar*C*(4)), 174.8 (*C*=O); HRMS (APCI$^+$) $C_{26}H_{36}O_4$ ([M+Na]$^+$) requires 435.25058, found 435.2504 (–0.47 ppm).

<Chromatogram>

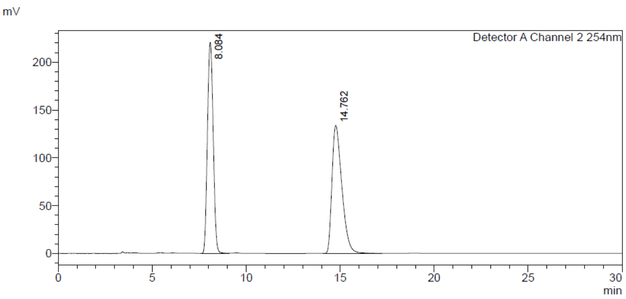

<Peak Table>

Detector A Channel 2 254nm

Peak#	Ret. Time	Area%
1	8.084	49.079
2	14.762	50.921
Total		100.000

<Chromatogram>

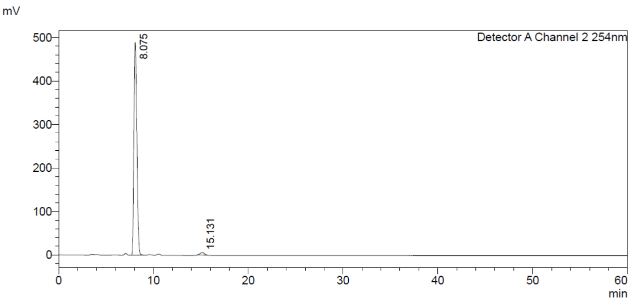

<Peak Table>

Detector A Channel 2 254nm

Peak#	Ret. Time	Area%
1	8.075	98.338
2	15.131	1.662
Total		100.000

(*Rp*)-1^5-butyl-2,15-dioxa-1(1,4)-benzenacyclopentadecaphane-1^2-yl isobutyrate 25

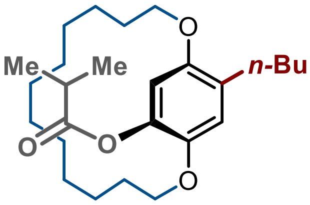

Following General Procedure F and purification of the residue by silica gel chromatography column using 10:1 petrol ether/EtOAc as eluent gave the product **25** as a colourless oil (yield 87%, 96:4 er); $[\alpha]_D^{20}$ –11.3 (c 1.7 in $CHCl_3$); chiral HPLC analysis, ChiralPak ID-H (3% *i*-PrOH : hexane, flow rate 1 mLmin^{-1}, 211 nm, 30 °C), tR major: 4.9 min, tR minor: 8.0 min, 96:4 er; ν_{max} (film)/cm^{-1} 2926 (C–H), 2857 (C–H), 1759 (C=O), 1504 (C=C), 1207 (C–O), 1194 (C–O); **^{1}H NMR** (500 MHz, $CDCl_3$) δ_H: 0.92-1.09 (11H, m, 4 × C*H*$_2$, Ar(5)-$CH_2CH_2CH_2C$*H*$_3$), 1.10-1.31 (6H, 3 × C*H*$_2$), 1.31-1.35 (4H, m, CH(C*H*$_3$)^A(CH$_3$)^B, C*H*^{A}H^B), 1.35-1.38 (4H, m, CH(CH$_3$)^A(C*H*$_3$)^B), CH^A*H*^{B}), 1.38-1.46 (2H, m, Ar(5)-CH_2CH_2C*H*$_2$CH$_3$), 1.48-1.61 (4H, m, C*H*$_2$, Ar(5)-CH$_2$C*H*$_2$CH$_2$CH$_3$), 1.62-1.75 (1H, m, C*H*^{A}H^B), 1.76-1.89 (1H, m, CH^A*H*^{B}), 2.55 (1H, ddd, *J* 13.7, *J* 8.9, *J* 6.3, Ar(5)-C*H*^{A}H^{B}C$_3$H$_7$), 2.65 (1H, ddd, *J* 13.7, *J* 9.0, *J* 6.5, Ar(5)-CH^A*H*^{B}C$_3$H$_7$), 2.84 (1H, hept, *J* 7.0, C*H*(CH$_3$)$_2$), 4.04-4.16 (2H, m, ArC(4)-OC*H*$_2$), 4.19-4.31 (2H, m, ArC(1)-OC*H*$_2$), 6.61 (1H, s, ArC(3)*H*), 6.84 (1H, s, ArC(6)*H*); **^{13}C{1H} NMR** (126 MHz, $CDCl_3$) δ_C: 14.0 (Ar(5)-$CH_2CH_2CH_2CH_3$), 18.9 (CH(*C*H$_3$)^A(CH$_3$)^B), 19.3 (CH(CH$_3$)^A(*C*H$_3$)^B), 22.6 (Ar(5)-$CH_2CH_2CH_2CH_3$), 23.9 (CH$_2$), 24.1 (CH$_2$), 26.9 (CH$_2$), 27.5 (CH$_2$), 27.6 (CH$_2$), 27.6 (CH$_2$), 27.8 (CH$_2$), 28.0 (CH$_2$), 28.4 (CH$_2$), 28.5 (CH$_2$), 29.6 (Ar(5)-$CH_2CH_2CH_2CH_3$), 32.1 (Ar(5)-$CH_2CH_2CH_2CH_3$), 34.1 (CH(CH$_3$)$_2$), 67.9 (ArC(4)-OCH$_2$), 69.4 (ArC(1)-OCH$_2$), 108.7 (ArC(3)H), 118.8 (ArC(6)H), 129.8 (ArC(5)), 139.3 (Ar*C*(2)), 142.7 (ArC(1)), 149.8 (Ar*C*(4)), 175.1 (*C*=O); HRMS (APCI$^+$) $C_{26}H_{42}O_4$ ([M+Na]$^+$) requires 441.29753, found 441.2968 (–1.64 ppm).

<Chromatogram>

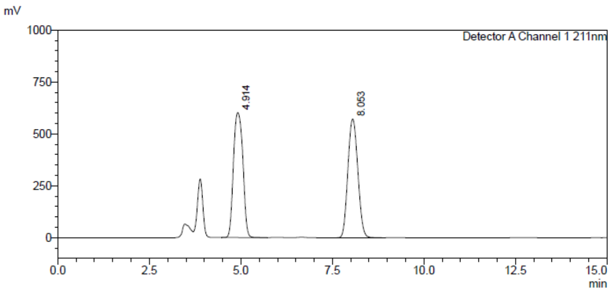

<Peak Table>

Detector A Channel 1 211nm

Peak#	Ret. Time	Area%
1	4.914	49.848
2	8.053	50.152
Total		100.000

<Chromatogram>

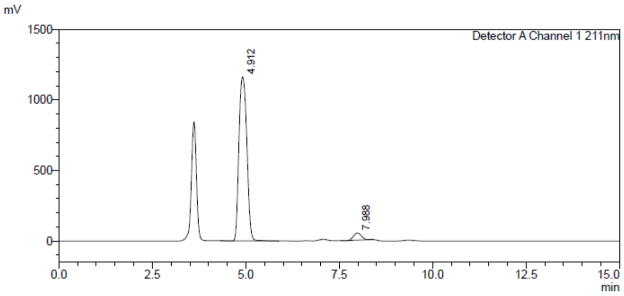

<Peak Table>

Detector A Channel 1 211nm

Peak#	Ret. Time	Area%
1	4.912	95.776
2	7.988	4.224
Total		100.000

(*Rp*)-1^5-bromo-2,6,11,15-tetraoxa-1(1,4)-benzenacyclopentadecaphane-1^2-yl isobutyrate 26

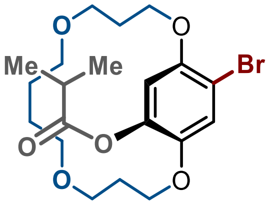

Following General Procedure F and purification of the residue by silica gel chromatography column using 4:1 petrol ether/EtOAc as eluent gave the product **26** as a colourless oil (yield 91%, 97:3 er); $[\alpha]_D^{20}$ –28.3 (c 2.4 in $CHCl_3$); chiral HPLC analysis, ChiralPak ID-H (3% *i*-PrOH : hexane, flow rate 1 mLmin^{-1}, 254 nm, 30 °C), tR major: 11.8 min, tR minor: 28.7 min, 97:3 er; ν_{max} (film)/cm^{-1} 2938 (C–H), 2859 (C–H), 1759 (C=O), 1493 (C=C), 1198 (C–O), 1111 (C–O), 1043 (C–O); ^{1}**H NMR** (500 MHz, $CDCl_3$) δ_H: 1.07-1.23 (4H, m, 2 × C*H*$_2$), 1.31-1.38 (6H, m, CH(C*H*$_3$)$_2$), 1.84-1.95 (3H, m, C*H*$_2$, C*H*^{A}H^B), 2.00-2.12 (1H, m, CH^A*H*^{B}), 2.83 (1H, hept, *J* 7.0, C*H*(CH$_3$)$_2$), 3.05-3.18 (2H, m, OC*H*$_2$), 3.29 (1H, ddd, *J* 9.7, *J* 7.2, *J* 5.9, OC*H*^{A}H^B), 3.34-3.47 (2H, m, OC*H*$_2$), 3.68 (1H, ddd, *J* 10.5, *J* 9.0, *J* 3.5, OCH^A*H*^{B}), 4.22 (1H, dt, *J* 11.1, *J* 4.5, ArC(1)-OC*H*^{A}H^B), 4.25-4.38 (3H, m, ArC(1)-OCH^A*H*^{B}, ArC(4)-OC*H*$_2$), 6.73 (1H, s, ArC(3)*H*), 7.26 (1H, s, ArC(6)*H*); ^{13}**C{1H} NMR** (126 MHz, $CDCl_3$) δ_C: 19.0 (CH(CH$_3$)^A(CH$_3$)^B), 19.1 (CH(CH$_3$)^A(CH$_3$)^B), 25.7 (CH$_2$), 25.9 (CH$_2$), 30.1 (CH$_2$), 31.0 (CH$_2$), 34.0 ((CH(CH$_3$)$_2$)), 65.3 (OCH$_2$), 65.5 (OCH$_2$), 68.2 (ArC(4)-OCH$_2$), 68.3 (ArC(1)-OCH$_2$), 70.9 (OCH$_2$), 71.0 (OCH$_2$), 109.0 (ArC(5)), 111.3 (Ar*C*(3)H), 121.7 (Ar*C*(6)H), 140.5 (Ar*C*(2)), 146.7 (Ar*C*(1)), 151.4 (Ar*C*(4)), 174.5 (*C*=O); HRMS (APCI$^+$) $C_{20}H_{29}O_6Br$ ([M+Na]$^+$) requires 467.10397, found 467.1038 (–0.36 ppm).

<Chromatogram>

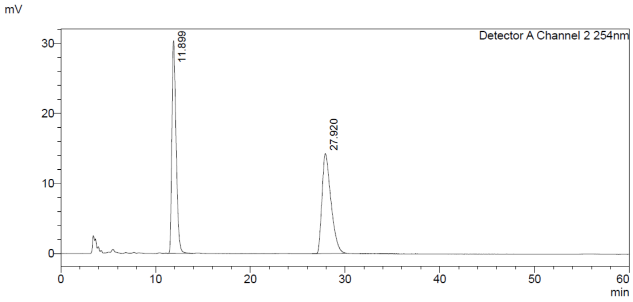

<Peak Table>

Detector A Channel 2 254nm

Peak#	Ret. Time	Area%
1	11.899	49.967
2	27.920	50.033
Total		100.000

<Chromatogram>

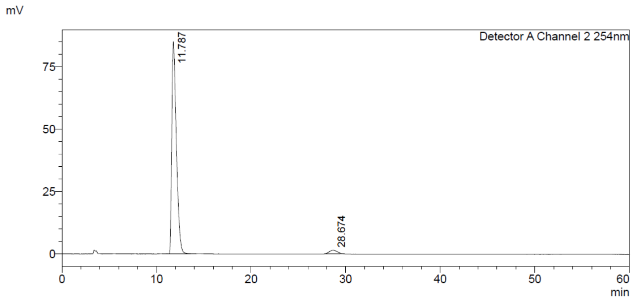

<Peak Table>

Detector A Channel 2 254nm

Peak#	Ret. Time	Area%
1	11.787	97.101
2	28.674	2.899
Total		100.000

(*Rp*)-1[5]-bromo-2,16-dioxa-1(1,4)-benzenacyclohexadecaphane-1[2]-yl isobutyrate 27

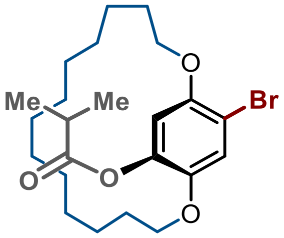

Following General Procedure F and purification of the residue by silica gel chromatography column using 4:1 petrol ether/EtOAc as eluent gave the product **27** as a colourless oil (yield 88%, 96:4 er); $[\alpha]_D^{20}$ –34.1 (c 2.2 in $CHCl_3$); chiral HPLC analysis, ChiralPak ID-H (3% *i*-PrOH : hexane, flow rate 1 $mLmin^{-1}$, 211 nm, 30 °C), tR major: 6.4 min, tR minor: 15.6 min, 96:4 er; ν_{max} (film)/cm^{-1} 2924 (C–H), 2853 (C–H), 1761 (C=O), 1491 (C=C), 1200 (C–O), 1136 (C–O), 1090 (C–O); **^{1}H NMR** (500 MHz, $CDCl_3$) δ_H: 0.98-1.32 (15H, m, 7 × C*H*$_2$, C*H*^{A}H^B), 1.33 (4H, d, *J* 7.0, CH(C*H*$_3$)^A(CH$_3$)^B, CH^A*H*^{B}), 1.36 (4H, d, *J* 7.0, CH(CH$_3$)^A(C*H*$_3$)^B, C*H*^{A}H^B), 1.44-1.55 (1H, m, CH^A*H*^{B}), 1.58-1.75 (4H, m, 2 × C*H*$_2$), 2.84 (1H, hept, *J* 7.0, C*H*(CH$_3$)$_2$), 4.12 (1H, ddd, *J* 11.4, *J* 6.8, *J* 4.7, ArC(1)-OC*H*^{A}H^B), 4.19 (1H, *J* 11.4, *J* 6.9, *J* 4.5, ArC(1)-OCH^A*H*^{B}), 4.23 (2H, t, *J* 5.7, ArC(4)-OC*H*$_2$), 6.72 (1H, s, ArC(3)*H*), 7.22 (1H, s, ArC(6)*H*); **^{13}C{1H} NMR** (126 MHz, $CDCl_3$) δ_C: 18.9 (CH(CH$_3$)^A(CH$_3$)^B), 19.2 (CH(CH$_3$)^A(*C*H$_3$)^B), 23.7 (CH$_2$), 24.0 (CH$_2$), 27.3 (CH$_2$), 27.4 (CH$_2$), 27.4 (CH$_2$), 27.9 (CH$_2$), 27.9 (CH$_2$), 28.1 (CH$_2$), 28.2 (CH$_2$), 28.3 (CH$_2$), 29.4 (*C*H$_2$), 34.0 (*C*H(CH$_3$)$_2$), 69.0 (ArC(4)-OCH$_2$), 69.4 (ArC(1)-OCH$_2$), 109.2 (ArC(5)), 110.7 (Ar*C*(3)H), 120.0 (Ar*C*(6)H), 140.5 (Ar*C*(2)), 144.5 (Ar*C*(1)), 148.8 (Ar*C*(4)), 174.6 (*C*=O); HRMS (APCI$^+$) $C_{23}H_{35}O_4Br$ ([M+Na]$^+$) requires 477.16109 found 477.1607 (–0.74 ppm).

<Chromatogram>

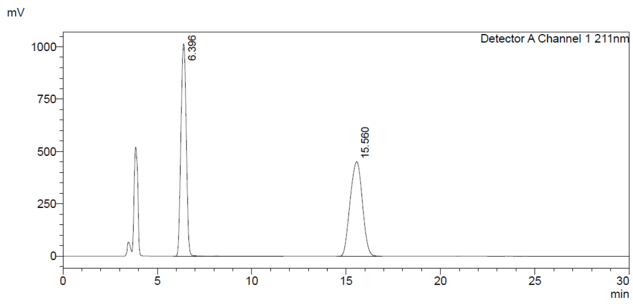

<Peak Table>

Detector A Channel 1 211nm

Peak#	Ret. Time	Area%
1	6.396	49.433
2	15.560	50.567
Total		100.000

<Chromatogram>

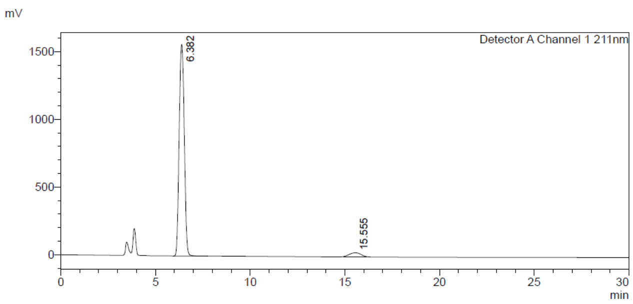

<Peak Table>

Detector A Channel 1 211nm

Peak#	Ret. Time	Area%
1	6.382	96.265
2	15.555	3.735
Total		100.000

(*Rp*)-1⁵-phenyl-2,16-dioxa-1(1,4)-benzenacyclohexadecaphane-1²-yl isobutyrate 28

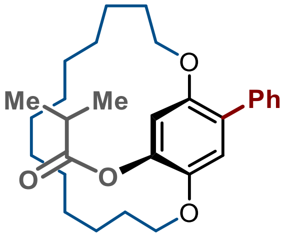

Following General Procedure F and purification of the residue by silica gel chromatography column using 4:1 petrol ether/EtOAc as eluent gave the product **28** as a colourless oil (yield 88%, 97.5:2.5 er); $[\alpha]_D^{20}$ –36.4 (c 1.8 in $CHCl_3$); chiral HPLC analysis, ChiralPak ID-H (3% *i*-PrOH : hexane, flow rate 1 mLmin^{-1}, 254 nm, 30 °C), tR major: 5.6 min, tR minor: 13.6 min, 97.5:2.5 er; ν_{max} (film)/cm^{-1} 2926 (C–H), 2855 (C–H), 1759 (C=O), 1487 (C=C), 1194 (C–O), 1128 (C–O); **^{1}H NMR** (500 MHz, $CDCl_3$) δ_H: 0.99-1.36 (17H, m, 8 ×C*H*$_2$, C*H*^{A}H^B), 1.38 (3H, d, *J* 7.0, CH(C*H*$_3$)^A(CH$_3$)^B), 1.40 (3H, d, *J* 7.0, CH(CH$_3$)^A(C*H*$_3$)^B), 1.42-1.58 (2H, m, C*H*$_2$), 1.60-1.76 (3H, m, C*H*$_2$, CH^A*H*^{B}), 2.89 (1H, hept, *J* 7.0, C*H*(CH$_3$)$_2$), 4.01 (1H, ddd, *J* 11.6, *J* 7.3, *J* 4.6, ArC(4)-OC*H*^{A}H^B), 4.08-4.19 (2H, m, ArC(4)-OCH^A*H*^{B}, ArC(1)-OC*H*^{A}H^B), 4.27 (1H, ddd, *J* 11.3, *J* 7.0, *J* 4.3, ArC(1)-OCH^A*H*^{B}), 6.78 (1H, s, ArC(3)*H*), 7.02 (1H, s, ArC(6)*H*), 7.32-7.38 (1H, m, ArC(5)-PhC(4)*H*), 7.40-7.47 (2H, m, ArC(5)-PhC(3,5)*H*), 7.51-7.58 (2H, m, ArC(5)-PhC(2,6)*H*); **^{13}C{1H} NMR** (126 MHz, $CDCl_3$) δ_C: 19.0 (CH(CH$_3$)^A(CH$_3$)^B), 19.3 (CH(CH$_3$)^A(CH$_3$)^B), 24.0 (CH$_2$), 24.0 (CH$_2$), 27.4 (CH$_2$), 27.5 (CH$_2$), 27.6 (CH$_2$), 28.1 (CH$_2$), 28.1(CH$_2$), 28.1 (CH$_2$), 28.2 (CH$_2$), 28.4 (CH$_2$), 29.4 (CH$_2$), 34.1 (CH(CH$_3$)$_2$), 68.7 (ArC(4)-OCH$_2$), 69.0 (ArC(1)-OCH$_2$), 110.3 (ArC(3)H), 118.2 (Ar*C*(6)H), 126.9 (ArC(5)-Ph*C*(4)H), 127.9 (ArC(5)-Ph*C*(3,5)H), 129.6 (ArC(5)-Ph*C*(2,6)H), 129.7 (ArC(5)-Ph), 138.4 (ArC(5)-PhC(1)), 140.5 (ArC(2)), 143.9 (ArC(1)), 149.3 (ArC(4)), 175.0 (*C*=O); HRMS (APCI$^+$) $C_{29}H_{40}O_4$ ([M+Na]$^+$) requires 475.28188, found 475.2815 (–0.86 ppm).

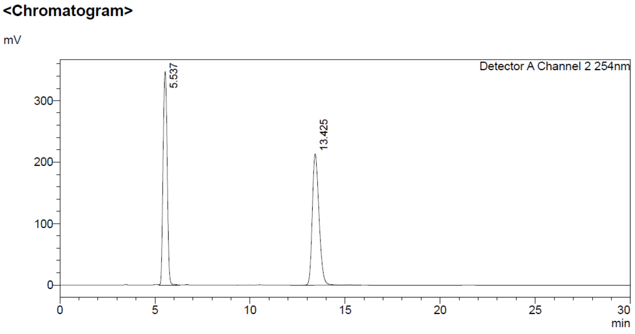

<Peak Table>

Detector A Channel 2 254nm

Peak#	Ret. Time	Area%
1	5.537	49.991
2	13.425	50.009
Total		100.000

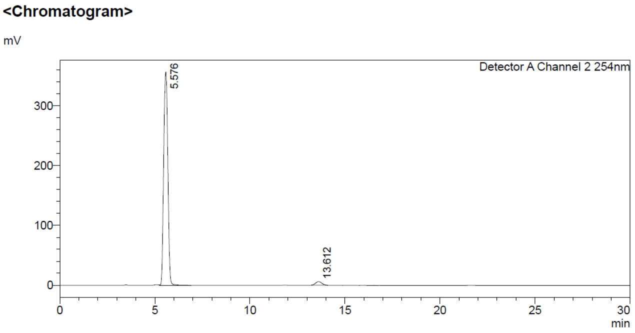

<Peak Table>

Detector A Channel 2 254nm

Peak#	Ret. Time	Area%
1	5.576	97.569
2	13.612	2.431
Total		100.000

(*Rp*)-1^5-bromo-2,17-dioxa-1(1,4)-benzenacyclohexadecaphane-1^2-yl isobutyrate 29

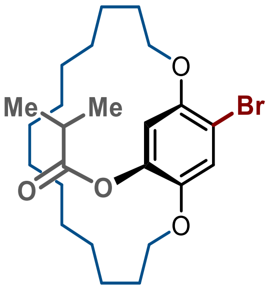

Following General Procedure F and purification of the residue by silica gel chromatography column using 4:1 petrol ether/EtOAc as eluent gave the product **29** as a colourless oil (yield 92%); ν_{max} (film)/cm^{-1} 2924 (C–H), 2853 (C–H), 1763 (C=O), 1495 (C=C), 1203 (C–O), 1136 (C–O), 731 (C–Br); **^{1}H NMR** (500 MHz, $CDCl_3$) δ_H: 0.92-1.32 (18H, m, 9 × C*H*$_2$), 1.34 (3H, d, *J* 7.0, CH(C*H*$_3$)^A(CH$_3$)^B), 1.36 (4H, d, *J* 7.1, CH(CH$_3$)^A(C*H*$_3$)^B), 1.40-1.52 (1H, m, C*H*^{A}H^B), 1.53-1.63 (1H, m, CH^A*H*^{B}), 1.63-1.79 (4H, m, 2 × C*H*$_2$), 2.84 (1H, hept, *J* 7.0, C*H*(CH$_3$)$_2$), 4.06-4.15 (2H, m, ArC(1)-OC*H*$_2$), 4.15-4.24 (2H, m, ArC(4)-OCH$_2$), 6.70 (1H, s, ArC(3)*H*), 7.19 (1H, s, ArC(6)*H*); **^{13}C{1H} NMR** (126 MHz, $CDCl_3$) δ_C: 18.9 (CH(CH$_3$)^A(CH$_3$)^B), 19.1 (CH(CH$_3$)^A(CH$_3$)^B), 23.6 (CH$_2$), 23.8 (CH$_2$), 26.8 (CH$_2$), 27.0 (CH$_2$), 27.2 (CH$_2$), 27.5 (CH$_2$), 27.9 (CH$_2$), 28.0 (CH$_2$), 28.2 (CH$_2$), 28.3 (CH$_2$), 28.9 (CH$_2$), 29.0 (CH$_2$), 34.0 (CH(CH$_3$)$_2$), 68.4 (ArC(4)-OCH$_2$), 68.9 (ArC(1)-OCH$_2$), 108.7 (ArC(5)), 110.1 (Ar*C*(3)H), 119.2 (Ar*C*(6)H), 140.1 (Ar*C*(2)), 144.3 (Ar*C*(1)), 148.6 (Ar*C*(4)), 174.6 (*C*=O); HRMS (APCI$^+$) $C_{24}H_{37}O_4Br$ ([M+Na]$^+$) requires 491.17674 found 491.1764 (–0.70 ppm).

(*Rp*)-1^5-phenyl-2,17-dioxa-1(1,4)-benzenacyclohexadecaphane-1^2-yl isobutyrate 30

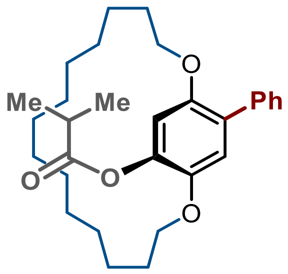

Following General Procedure F and purification of the residue by silica gel chromatography column using 4:1 petrol ether/EtOAc as eluent gave the product **30** as a colourless oil (yield 86%, 97.5:2.5 er); $[\alpha]_D^{20}$ –27.5 (c 1.2 in $CHCl_3$); chiral HPLC analysis, ChiralPak ID-H (3% *i*-PrOH : hexane, flow rate 1 $mLmin^{-1}$, 254 nm, 30 °C), tR major: 5.1 min, tR minor: 10.5 min, 97.5:2.5 er; ν_{max} (film)/cm^{-1} 2924 (C–H), 2855 (C–H), 1759 (C=O), 1489 (C=C), 1198 (C–O), 1128 (C–O); **^{1}H NMR** (500 MHz, $CDCl_3$) δ_H: 1.03-1.35 (19H, m, 9 × C*H*$_2$, C*H*^{A}H^B), 1.37 (3H, d, *J* 7.0, CH(C*H*$_3$)^A(CH$_3$)^B), 1.40 (3H, d, *J* 7.0, CH(CH$_3$)^A(C*H*$_3$)^B), 1.45-1.56 (1H, m, CH^A*H*^{B}), 1.60-1.75 (4H, m, 2 × C*H*$_2$), 2.88 (1H, hept, *J* 7.0, C*H*(CH$_3$)$_2$), 3.99 (1H, dt, *J* 10.9, *J* 5.5, ArC(4)-OC*H*^{A}H^B), 4.07 (1H, dt, *J* 11.2, *J* 6.2, ArC(4)-OCH^A*H*^{B}), 4.12 (1H, ddd, *J* 11.6, *J* 6.7, *J* 5.1, ArC(1)-OC*H*^{A}H^B), 4.22 (1H, ddd, *J* 11.5, *J* 7.3, *J* 4.5, ArC(1)-OCH^A*H*^{B}), 6.75 (1H, s, ArC(3)*H*), 6.99 (1H, s, ArC(6)*H*), 7.31-7.38 (1H, m, ArC(5)-PhC(4)*H*), 7.40-7.46 (2H, m, ArC(5)-PhC(3,5)*H*), 7.49-7.57 (2H, m, ArC(5)-PhC(2,6)*H*); **^{13}C{1H} NMR** (126 MHz, $CDCl_3$) δ_C: 19.0 (CH(CH$_3$)^A(CH$_3$)^B), 19.2 (CH(CH$_3$)^A(CH$_3$)^B), 23.8 (CH$_2$), 23.8 (CH$_2$), 26.8 (CH$_2$), 27.2 (CH$_2$), 27.4 (CH$_2$), 27.6 (CH$_2$), 27.9 (CH$_2$), 28.0 (CH$_2$), 28.3 (CH$_2$), 28.4 (CH$_2$), 28.8 (CH$_2$), 29.0 (CH$_2$), 34.1 (CH(CH$_3$)$_2$), 68.1 (ArC(4)-OCH$_2$), 68.5 (ArC(1)-OCH$_2$), 109.5 (ArC(3)H), 117.6 (ArC(6)H), 126.9 (ArC(5)-PhC(4)H), 127.9 (ArC(5)-Ph*C*(3,5)H), 129.2 (ArC(5)-Ph*C*(2,6)H), 129.6 (ArC(5)), 138.4 (ArC(5)-Ph*C*(1)), 140.2 (Ar*C*(2)), 143.6 (Ar*C*(1)), 149.2 (Ar*C*(4)), 175.0 (*C*=O); HRMS (APCI$^+$) $C_{30}H_{42}O_4$ ([M+Na]$^+$) requires 489.29753, found 489.2973 (–0.38 ppm).

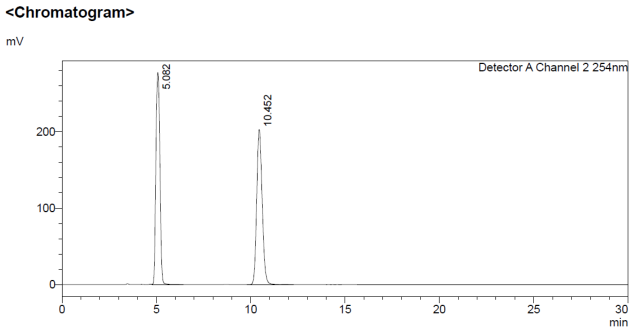

<Peak Table>

Detector A Channel 2 254nm

Peak#	Ret. Time	Area%
1	5.082	50.061
2	10.452	49.939
Total		100.000

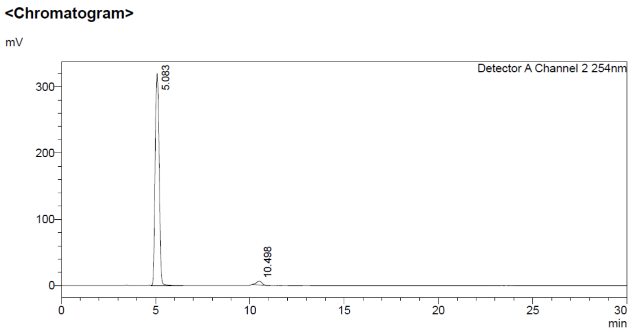

<Peak Table>

Detector A Channel 2 254nm

Peak#	Ret. Time	Area%
1	5.083	97.517
2	10.498	2.483
Total		100.000

(*Rp*)-1^5-bromo-4,15-dioxo-2,17-dioxa-5,14-diaza-1(1,4)-benzenacycloheptadecaphane-1^2-yl isobutyrate 31

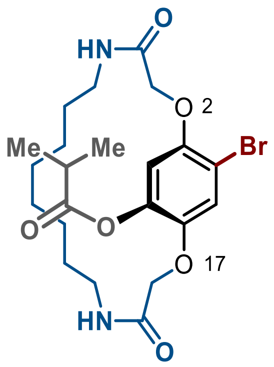

Following General Procedure F and purification of the residue by silica gel chromatography column using 4:1 petrol ether/EtOAc as eluent gave the product **31** as a colourless oil (yield 92%); ν_{max} (film)/cm^{-1} 3364 (N–H), 2930 (C–H), 2853 (C–H), 1751 (C=O), 1676 (C=O), 1655 (C=O), 1497 (C=C), 1204 (C–O), 1123 (C–O), 1045 (C–O); **^{1}H NMR** (500 MHz, $CDCl_3$) δ_H: 0.81-0.92 (2H, m, C*H*$_2$), 0.98-1.17 (2H, m, C*H*$_2$), 1.33 (3H, d, *J* 7.2, CH(C*H*$_3$)^A(CH$_3$)^B), 1.35 (3H, d, *J* 7.1, CH(CH$_3$)^A(C*H*$_3$)^B), 1.36-1.64 (4H, m, 2 × C*H*$_2$), 2.81-2.91 (2H, m, C*H*(CH$_3$)$_2$, C(6)*H*^{A}H^B), 3.02 (1H, ddd, *J* 13.9, *J* 7.2, *J* 3.5, C(6)H^A*H*^{B}), 3.56-3.72 (2H, m, C(13) *H*$_2$), 4.48-4.65 (3H, m, ArC(4)-OC*H*^{A}H^B, ArC(1)-OC*H*$_2$), 4.73 (1H, d, *J* 16.1, ArC(4)-OCH^A*H*^{B}), 6.53 (1H, dd, *J* 8.0, *J* 3.5, C(4)ON*H*), 6.68 (1H, s, ArC(3)*H*), 6.95 (1H, dd, *J* 7.9, *J* 3.0, C(15)ON*H*), 7.11 (1H, s, ArC(6)*H*); **^{13}C{1H} NMR** (126 MHz, $CDCl_3$) δ_C: 18.8 (CH(CH$_3$)^A(CH$_3$)^B), 19.1 (CH(CH$_3$)^A(CH$_3$)^B), 25.7 (CH$_2$), 26.2 (CH$_2$), 28.0 (CH$_2$), 28.6 (CH$_2$), 29.3 (CH$_2$), 29.5 (CH$_2$), 34.0 (CH(CH$_3$)$_2$), 39.1 (CH$_2$), 39.2 (CH$_2$), 66.8 (ArC(1)-OCH$_2$), 68.8 (ArC(4)-OCH$_2$), 107.9 (Ar*C*(5)), 108.3 (Ar*C*(3)H), 116.2 (Ar*C*(6)H), 139.2 (Ar*C*(2)), 143.7 (Ar*C*(1)), 148.4 (Ar*C*(4)), 167.4 (*C*(4)ONH), 167.8 (*C*(15)ONH), 175.3 (*C*=O); HRMS (APCI$^+$) $C_{22}H_{31}O_6N_2Br$ ([M+Na]$^+$) requires 521.12577 found 521.1250 (–1.40 ppm).

(*Rp*)-4,15-dioxo-1^5-phenyl-2,17-dioxa-5,14-diaza-1(1,4)-benzenacycloheptadecaphane-1^2-yl isobutyrate 32

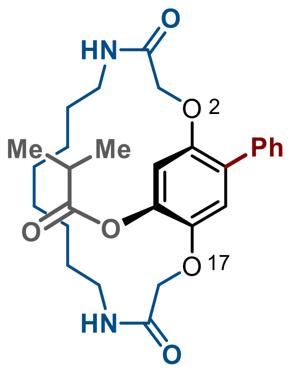

Following General Procedure F and purification of the residue by silica gel chromatography column using 4:1 petrol ether/EtOAc as eluent gave the product **32** as a colourless oil (yield 93%, 90:10 er); $[\alpha]_D^{20}$ –6.0 (c 0.5 in $CHCl_3$); chiral HPLC analysis, ChiralPak ID-H (3% *i*-PrOH : hexane, flow rate 1 mLmin^{-1}, 254 nm, 30 °C),

tR major: 8.5 min, tR minor: 10.4 min, 90:10 er; ν_{max} (film)/cm^{-1} 3368 (N–H), 2930 (C–H), 2855 (C–H), 1748 (C=O), 1668 (C=O), 1487 (C=C), 1196 (C–O), 1132 (C–O); **^{1}H NMR** (500 MHz, $CDCl_3$) δ_H: 0.86-1.09 (6H, m, 3 × CH_2), 1.11-1.22 (3H, m, CH_2, C$H^A$$H^B$), 1.36 (3H, d, *J* 7.0, CH(CH_3)^A(CH_3)^B), 1.37 (3H, d, *J* 7.0, CH(CH_3)^A(CH_3)^B), 1.40-1.58 (3H, m, CH_2, C$H^A$$H^B$), 2.73 (1H, ddt, *J* 14.0, *J* 7.3, *J* 3.5, C(6)$H^A$$H^B$), 2.84-2.95 (2H, m, C*H*(CH_3)$_2$, C(6)$H^A$$H^B$), 3.55 (1H, dtd, *J* 13.3, *J* 8.1, *J* 3.5, C(13)$H^A$$H^B$), 3.70 (1H, dtd, *J* 13.4, *J* 7.7, *J* 3.7, C(13)$H^A$$H^B$), 4.47 (1H, d, *J* 16.1, ArC(4)-OC$H^A$$H^B$), 4.55-4.69 (3H, m, ArC(4)-OC$H^A$$H^B$, ArC(1)-OC$H_2$), 6.05 (1H, dd, *J* 7.9, *J* 3.7, C(4)ON*H*), 6.71 (1H, s, ArC(3)*H*), 6.88 (1H, s, ArC(6)*H*), 7.14 (1H, dd, *J* 7.9, *J* 3.6, C(14)ON*H*), 7.37-7.51 (5H, m, ArC(5)-PhC(2,3,4,5,6)*H*); **^{13}C{1H} NMR** (126 MHz, $CDCl_3$) δ_C: 18.9 (CH(CH_3)^A(CH_3)^B), 19.2 (CH(CH_3)^A(CH_3)^B), 25.7 (CH_2), 26.2 (CH_2), 27.9 (CH_2), 28.8 (CH_2), 29.3 (CH_2), 29.5 (CH_2), 34.1 (CH(CH_3)$_2$), 38.9 (CH_2), 39.0 (CH_2), 66.5 (ArC(1)-OCH_2), 69.1 (ArC(4)-OCH_2), 108.3 (Ar*C*(3)H), 113.7 (Ar*C*(6)H), 127.9 (ArC(5)-Ph*C*(4)H), 128.5 (ArC(5)-Ph*C*(3,5)H), 129.0 (ArC(5)), 129.6 (ArC(5)-Ph*C*(2,6)H), 136.6 (ArC(5)-Ph*C*(1)), 139.0 (Ar*C*(2)), 143.3 (Ar*C*(1)), 148.7 (Ar*C*(4)), 168.0 (*C*(4)ONH), 168.4 (*C*(15)ONH), 175.6 (*C*=O); HRMS (APCI$^+$) $C_{28}H_{36}O_4N_2$ ([M+Na]$^+$) requires 519.24656, found 519.2458 (–1.55 ppm).

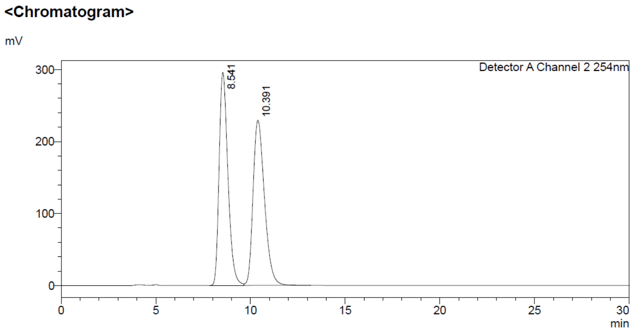

<Peak Table>

Detector A Channel 2 254nm

Peak#	Ret. Time	Area%
1	8.541	49.876
2	10.391	50.124
Total		100.000

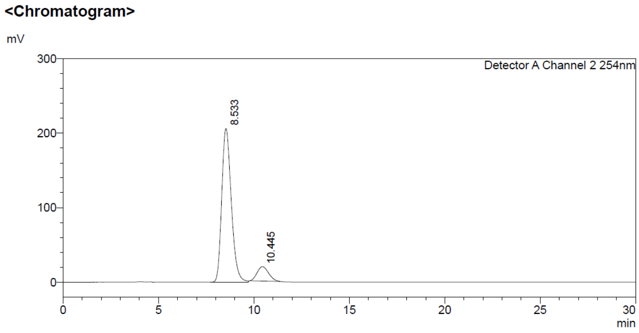

<Peak Table>

Detector A Channel 2 254nm

Peak#	Ret. Time	Area%
1	8.533	89.883
2	10.445	10.117
Total		100.000

(*Rp*)-4,14-dioxo-1^5-phenyl-2,8,10,16-tetraoxa-5,13-diaza-1(1,2),9(1,4)-dibenzenacyclohexadecaphane-1^2-yl isobutyrate 33

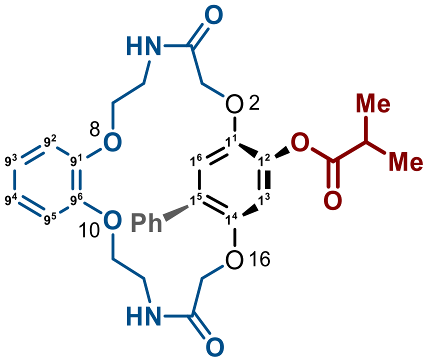

Following General Procedure F and purification by silica gel chromatography column using 4:1 petrol ether/EtOAc as eluent gave the product **33** as a colourless oil (yield 83%, 68:32 er); $[\alpha]_D^{20}$ –17.1 (c 0.6 in $CHCl_3$); chiral HPLC analysis, ChiralPak ID-H (25% *i*-PrOH : hexane, flow rate 0.8 $mLmin^{-1}$, 254 nm, 30 °C), tR minor: 25.6 min, tR major: 33.9 min, 68:32 er; ν_{max} (film)/cm^{-1} 3420 (N–H), 2938 (C–H), 2880 (C–H), 1759 (C=O), 1667 (C=O), 1505 (C=C), 1487 (C=C), 1196 (C–O), 1126 (C–O), 1053 (C–O); **^{1}H NMR** (500 MHz, $CDCl_3$) δ_H: 1.33 (3H, d, *J* 7.0, $CH(CH_3)^A(CH_3)^B$), 1.35 (3H, d, *J* 7.1, $CH(CH_3)^A(CH_3)^B$), 2.86 (1H, hept, *J* 7.0, $CH(CH_3)_2$), 3.37 (2H, m, C(6)H^AH^B, C(12)H^AH^B), 3.56 (1H, ddd, *J* 14.3, *J* 7.1, *J* 2.5, C(6)H^AH^B), 3.84 (1H, ddd, *J* 10.3, *J* 7.1, *J* 2.5, C(11)H^AH^B), 3.92-4.05 (3H, m, C(12)H^AH^B, C(11)H^AH^B, C(7)H^AH^B), 4.33 (1H, m, C(7)H^AH^B), 4.56-4.74 (4H, m, ArC(4)-OCH_2, ArC(1)-OCH_2), 6.14 (1H, t, *J* 5.5, C(4)ON*H*), 6.75 (1H, dd, *J* 7.8, *J* 1.5, ArC(9^3)*H*), 6.77 (1H, s, ArC(1^3)*H*), 6.83 (1H, s, ArC(1^6)*H*), 6.91-7.02 (5H, m, ArC($9^{2,4,5}$)*H*, ArC(1^5)-PhC(3,5)*H*), 7.18-7.23 (1H, m, ArC(1^5)-PhC(4)*H*), 7.23-7.27 (2H, m, ArC(1^5)-PhC(2,6)*H*), 7.32 (1H, t, *J* 5.4, C(14)ON*H*); **^{13}C{1H} NMR** (126 MHz, $CDCl_3$) δ_C: 18.8 ($CH(CH_3)^A(CH_3)^B$), 19.1 ($CH(CH_3)^A(CH_3)^B$), 34.0 ($CH(CH_3)_2$), 38.8 (*C*(6)H_2), 39.9 (*C*(12)H_2), 66.4 (ArC(1)-OCH_2), 66.7 (*C*(7)H_2), 67.3 (*C*(11)H_2), 67.8 (ArC(4)-OCH_2), 108.1 (Ar*C*(1^3)H), 113.1 (Ar*C*(9^3)H), 113.4 (Ar*C*(9^4)H), 114.4 (Ar*C*(1^6)H), 121.2 (Ar*C*(9^2)H), 121.6 (Ar*C*(9^5)H), 127.7 (ArC(1^5)-Ph*C*(4)H), 128.1 (ArC(1^5)-Ph*C*(3,5)H), 129.7 (ArC(1^5)-Ph*C*(2,6)H), 129.9 (Ar*C*(1^5)), 136.3 (ArC(1^5)-Ph*C*(1)), 139.1 (Ar*C*(1^2)), 143.0 (ArC(1^1)), 147.7 (Ar*C*(1^4)), 147.9 (Ar*C*(9^6)), 148.7 (Ar*C*(9^1)), 168.5 (*C*(4)ONH), 168.7 (*C*(14)ONH), 175.5 (*C*=O); HRMS (APCI$^+$) $C_{30}H_{32}O_8N_2$ ([M+Na]$^+$) requires 571.20509, found 571.2047 (–0.68 ppm).

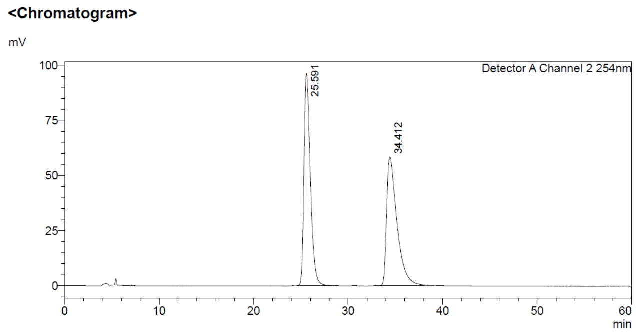

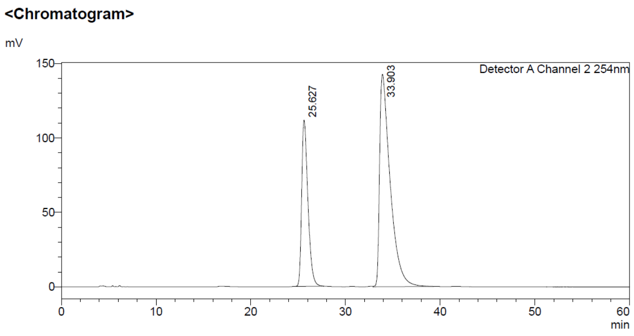

<Peak Table>

Detector A Channel 2 254nm

Peak#	Ret. Time	Area%
1	25.591	50.190
2	34.412	49.810
Total		100.000

<Peak Table>

Detector A Channel 2 254nm

Peak#	Ret. Time	Area%
1	25.627	31.661
2	33.903	68.339
Total		100.000

(*Rp*)-1^5-phenyl-2,18-dioxa-1(1,4)-benzenacyclohexadecaphane-1^2-yl isobutyrate 34

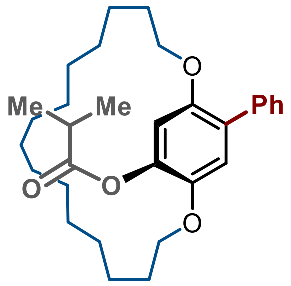

Following General Procedure F and purification of the residue by silica gel chromatography column using 4:1 petrol ether/EtOAc as eluent gave the product **34** as a colourless oil (yield 88%, 97:3 er); $[\alpha]_D^{20}$ –27.0 (c 0.3 in $CHCl_3$); chiral HPLC analysis, ChiralPak ID-H (3% *i*-PrOH : hexane, flow rate 1 mLmin^{-1}, 254 nm, 30 °C), tR major: 5.0 min, tR minor: 10.1 min, 97:3 er; ν_{max} (film)/cm^{-1} 2924 (C–H), 2853 (C–H), 1759 (C=O), 1489 (C=C), 1196 (C–O), 1128 (C–O); **^{1}H NMR** (500 MHz, $CDCl_3$) δ_H: 1.09-1.33 (21H, m, 10 × C*H*$_2$, C*H*^{A}H^B), 1.38 (3H, d, *J* 7.0, CH(C*H*$_3$)^A(CH$_3$)^B), 1.40 (3H, d, *J* 7.0, CH(CH$_3$)^A(C*H*$_3$)^B), 1.42-1.59 (2H, m, C*H*$_2$), 1.62-1.77 (3H, m, C*H*$_2$, CH^A*H*^{B}), 2.88 (1H, hept, *J* 7.0, C*H*(CH$_3$)$_2$), 3.96-4.06 (2H, m, ArC(4)-OC*H*$_2$), 4.11 (1H, dt, *J* 11.0, *J* 5.4, ArC(1)-OC*H*^{A}H^B), 4.18 (1H, ddd, *J* 10.9, *J* 7.4, *J* 4.8, ArC(1)-OCH^A*H*^{B}), 6.76 (1H, s, ArC(3)*H*), 7.00 (1H, s, ArC(6)*H*), 7.31-7.38 (1H, m, ArC(5)-PhC(4)*H*), 7.39-7.46 (2H, m, ArC(5)-PhC(3,5)*H*), 7.51-7.58 (2H, m, ArC(5)-PhC(2,6)*H*); **^{13}C{1H} NMR** (126 MHz, $CDCl_3$) δ_C: 19.0 (CH(*C*H$_3$)^A(CH$_3$)^B), 19.2 (CH(CH$_3$)^A(*C*H$_3$)^B), 24.3 (*C*H$_2$), 24.4 (*C*H$_2$), 27.2 (*C*H$_2$), 27.5 (*C*H$_2$), 27.8 (*C*H$_2$), 28.2 (*C*H$_2$), 28.2 (*C*H$_2$), 28.3 (*C*H$_2$), 28.4 (*C*H$_2$), 28.8 (*C*H$_2$), 29.1 (*C*H$_2$), 29.3 (*C*H$_2$), 34.1 (*C*H(CH$_3$)$_2$), 68.5 (ArC(4)-O*C*H$_2$), 68.9 (ArC(1)-O*C*H$_2$), 109.6 (Ar*C*(3)H), 117.6 (Ar*C*(6)H), 126.9 (ArC(5)-Ph*C*(4)H), 127.9 (ArC(5)-Ph*C*(3,5)H), 129.4 (ArC(5)-Ph*C*(2,6)H), 129.6 (Ar*C*(5)), 138.4 (ArC(5)-Ph*C*(1)), 140.3 (Ar*C*(2)), 144.0 (Ar*C*(1)), 149.5 (Ar*C*(4)), 175.0 (*C*=O); HRMS (APCI$^+$) $C_{31}H_{44}O_4$ ([M+Na]$^+$) requires 503.31318, found 503.3130 (–0.45 ppm).

<Chromatogram>

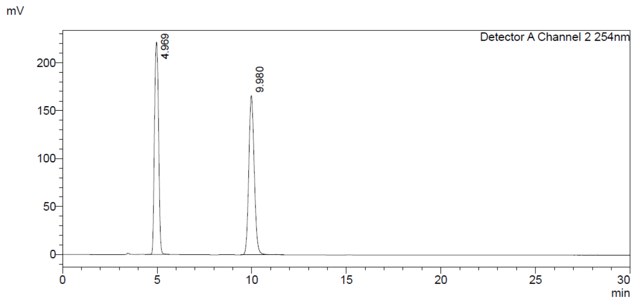

<Peak Table>

Detector A Channel 2 254nm

Peak#	Ret. Time	Area%
1	4.969	49.986
2	9.980	50.014
Total		100.000

<Chromatogram>

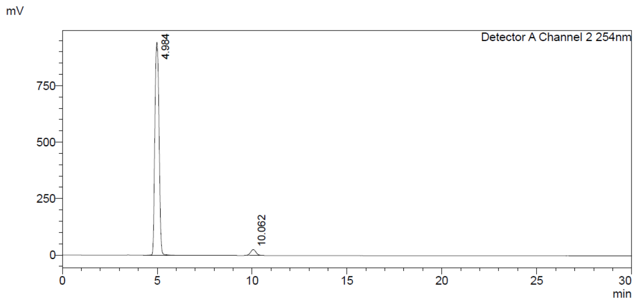

<Peak Table>

Detector A Channel 2 254nm

Peak#	Ret. Time	Area%
1	4.984	96.785
2	10.062	3.215
Total		100.000

(*Rp*)-1^5-phenyl-2,19-dioxa-1(1,4)-benzenacyclohexadecaphane-1^2-yl isobutyrate 35

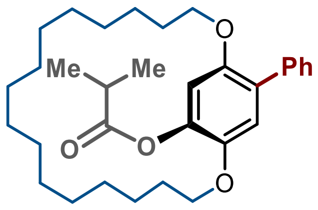

Following General Procedure F and purification of the residue by silica gel chromatography column using 4:1 petrol ether/EtOAc as eluent gave the product **35** as a colourless oil (yield 87%, 96:4 er); $[\alpha]_D^{20}$ –24.5 (c 0.4

in $CHCl_3$); chiral HPLC analysis, ChiralPak ID-H (3% *i*-PrOH : hexane, flow rate 1 $mLmin^{-1}$, 254 nm, 30 °C), tR major: 4.7 min, tR minor: 8.5 min, 96:4 er; ν_{max} (film)/cm^{-1} 2924 (C–H), 2853 (C–H), 1761 (C=O), 1489 (C=C), 1200 (C–O), 1126 (C–O); **^{1}H NMR** (500 MHz, $CDCl_3$) δ_H: 1.09-1.35 (23H, m, 11 × CH_2, C$H^A$$H^B$), 1.38 (3H, d, *J* 7.0, CH(CH_3)^A(CH_3)^B), 1.40 (3H, d, *J* 7.1, CH(CH_3)^A(CH_3)^B), 1.50-1.59 (2H, m, CH_2), 1.62-1.84 (3H, m, CH_2, C$H^A$$H^B$), 2.88 (1H, hept, *J* 7.0, C*H*(CH_3)$_2$), 3.91-4.05 (2H, m, ArC(4)-OCH_2), 4.05-4.21 (2H, m, ArC(1)-OCH_2), 6.74 (1H, s, ArC(3)*H*), 6.98 (1H, s, ArC(6)*H*), 7.31-7.38 (1H, m, ArC(5)-PhC(4)*H*), 7.38-7.49 (2H, m, ArC(5)-PhC(3,5)*H*), 7.50-7.62 (2H, m, ArC(5)-PhC(2,6)*H*); **^{13}C{1H} NMR** (126 MHz, $CDCl_3$) δ_C: 19.0 (CH(CH_3)^A(CH_3)^B), 19.2 (CH(CH_3)^A(CH_3)^B), 24.2 (2×CH_2), 26.8 (CH_2), 27.2 (CH_2), 27.6 (CH_2), 27.9 (CH_2), 28.0 (CH_2), 28.2 (CH_2), 28.2 (CH_2), 28.4 (CH_2), 28.6 (CH_2), 28.8 (CH_2), 29.3 (CH_2), 29.3 (CH_2), 34.1 (CH(CH_3)$_2$), 68.2 (ArC(4)-OCH_2), 68.5 (ArC(1)-OCH_2), 109.1 (Ar*C*(3)H), 117.1 (Ar*C*(6)H), 126.8 (ArC(5)-Ph*C*(4)H), 127.9 (ArC(5)-Ph*C*(3,5)H), 129.0 (ArC(5)-Ph*C*(2,6)H), 129.6 (ArC(5)), 138.4 (ArC(5)-Ph*C*(1)), 140.0 (Ar*C*(2)), 143.9 (Ar*C*(1)), 149.4 (Ar*C*(4)), 175.0 (*C*=O); HRMS (APCI$^+$) $C_{32}H_{46}O_4$ ([M+Na]$^+$) requires 517.32883, found 517.3293 (+0.86 ppm).

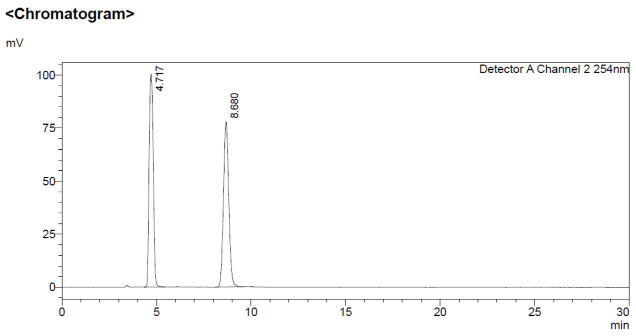

<Peak Table>

Detector A Channel 2 254nm

Peak#	Ret. Time	Area%
1	4.717	50.223
2	8.680	49.777
Total		100.000

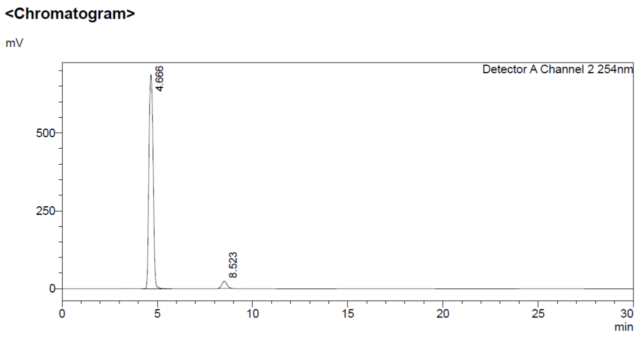

<Peak Table>

Detector A Channel 2 254nm

Peak#	Ret. Time	Area%
1	4.666	96.103
2	8.523	3.897
Total		100.000

3 Racemization experiment

3.1 Dynamic HPLC

Following the method described by Armstrong *et al.*,[37] weigh ~2 mg of the racemic macrocycle phenols and macrocycle esters compounds into a 2 ml glass HPLC vial and dissolved in neat 2-propanol. Carry out standard analytical HPLC runs (1 mLmin^{-1}; 15 μl injection volume; 30 ℃) aiming to achieve separation of the two enantiomer peaks with a plateau region in between. Export a copy of the raw HPLC data as an ASCII file format document (e.g., a .DAT, .TXT or .CSV document). The HPLC data was analyzed by DCXplorer and obtains the rate constant for enantiomerization, k_{ent}, for each substrate. k_{ent} is related to the racemization rate constant, k_{rac}, according to the following equation:

$$k_{rac} = 2k_{ent}$$

The half-life of racemization $t_{rac}^{1/2}$ at that temperature can be calculated as:

$$t_{rac}^{1/2} = \frac{\ln 2}{k_{rac}}$$

Substitute the value of k_{ent} along with the temperature of the column oven (this was 303.15 K) into the Eyring equation to calculate the $\Delta G^{\ddagger}$.

$$\Delta G^{\ddagger} = RT\ln(\frac{K_B T}{k_{ent} h})$$

R = Gas constant = 8.31454 J·K^{-1}·mol^{-1}, h = Planck constant = 6.62608×10^{-34} J·s, K_B = Boltzmann constant = 1.38066×10^{-23} J·K^{-1}.

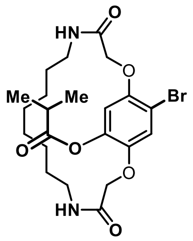

31
k_{rac}= 3.918 x 10^{-3} s^{-1}
ΔG_{303}= 21.9 kcal/mol

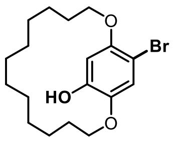

S47
k_{rac}= 7.840 X 10^{-4} s^{-1}
ΔG_{303}= 22.9 kcal/mol

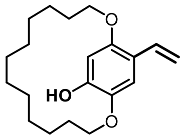

S39
k_{rac}= 9.030 x 10^{-4} s^{-1}
ΔG_{303}= 22.8 kcal/mol

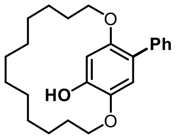

10
k_{rac}= 1.706 x 10^{-3} s^{-1}
ΔG_{303}= 22.4 kcal/mol

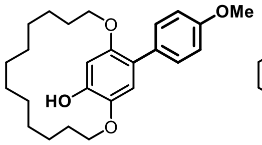

S48
k_{rac}= 1.451 x 10^{-3} s^{-1}
ΔG_{303}= 22.5 kcal/mol

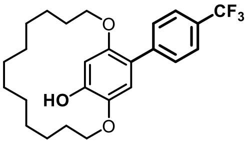

S49
k_{rac}= 1.614 x 10^{-3} s^{-1}
ΔG_{303}= 22.5 kcal/mol

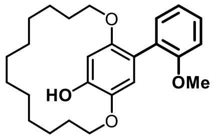

S50
k_{rac}= 1.186 x 10^{-3} s^{-1}
ΔG_{303}= 22.6 kcal/mol

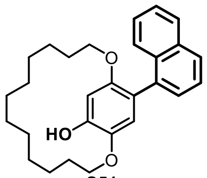

S51
k_{rac}= 1.952 x 10^{-3} s^{-1}
ΔG_{303}= 22.3 kcal/mol

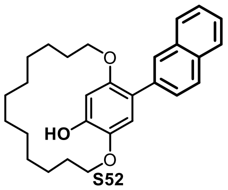

S52
k_{rac}= 1.406 x 10^{-3} s^{-1}
ΔG_{303}= 22.5 kcal/mol

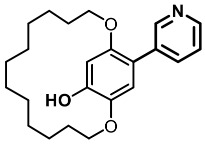

S53
k_{rac}= 1.305 x 10^{-3} s^{-1}
ΔG_{303}= 22.6 kcal/mol

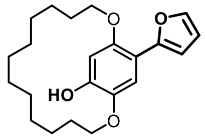

S54
k_{rac}= 1.238 x 10^{-3} s^{-1}
ΔG_{303}= 22.6 kcal/mol

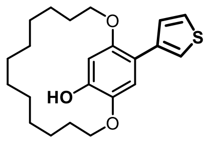

S55
k_{rac}= 1.501 x 10^{-3} s^{-1}
ΔG_{303}= 22.5 kcal/mol

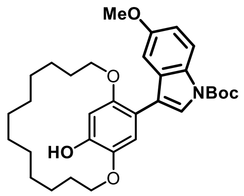

S56
k_{rac}= 1.476 x 10^{-3} s^{-1}
ΔG_{303}= 22.5 kcal/mol

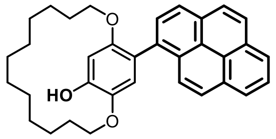

S57
k_{rac}= 8.266 x 10^{-4} s^{-1}
ΔG_{303}= 22.9 kcal/mol

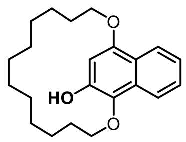

36
k_{rac}= 5.710 x 10^{-3} s^{-1}
ΔG_{303}= 21.7 kcal/mol

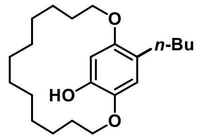

S69
k_{rac}= 1.201 x 10^{-3} s^{-1}
ΔG_{303}= 22.6 kcal/mol

3.2 Monitoring the rate of racemization

Following the method described by Armstrong *et al.*,[37] the chiral macrocycle (*Sp*)-**S45** was dissolved in toluene (0.012 M) and heated to 100 °C under N_2 protected. Determination of k_{rac} and racemization barriers $\Delta G^{\ddagger}$ of (*Sp*)-**S45** at 100 °C in toluene (Table S1).

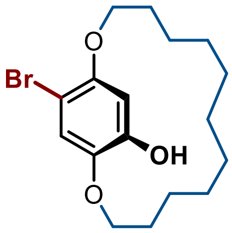

(S_p)-**S45**
toluene, 100 °C
k_{rac}= 2 x 10^{-6} s^{-1}
ΔG_{373}= 32.6 kcal/mol

Table S1. Racemization Kinetics of (*Sp*)-S45.

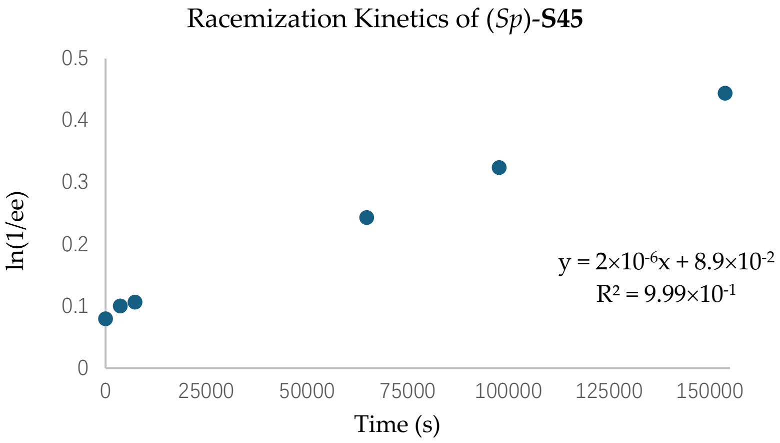

Time (s)	ln(1/ee)
0	0.079259681
3600	0.10001925
7320	0.106294285
64800	0.24237734
97668	0.323157275
153780	0.443509713

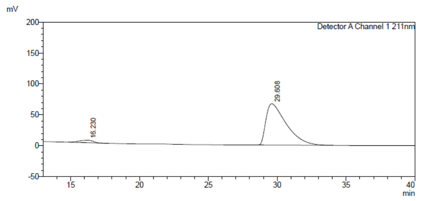

<Peak Table>

Detector A Channel 1 211nm

Peak#	Ret. Time	Area%
1	16.230	3.810
2	29.608	96.190
Total		100.000

Time (s): 0

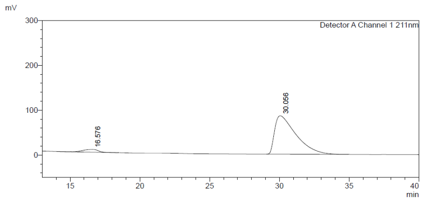

<Peak Table>

Detector A Channel 1 211nm

Peak#	Ret. Time	Area%
1	16.576	4.759
2	30.056	95.241
Total		100.000

Time (s): 3600

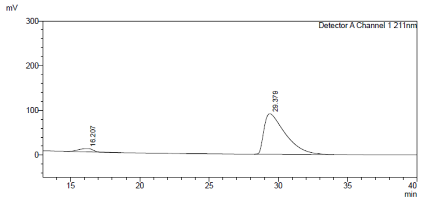

<Peak Table>

Detector A Channel 1 211nm

Peak#	Ret. Time	Area%
1	16.207	5.042
2	29.379	94.958
Total		100.000

Time (s): 7320

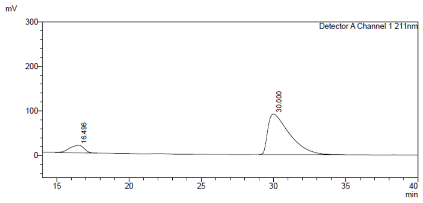

<Peak Table>

Detector A Channel 1 211nm

Peak#	Ret. Time	Area%
1	16.496	10.762
2	30.000	89.238
Total		100.000

Time (s): 64800

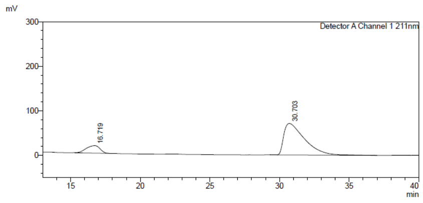

<Peak Table>

Detector A Channel 1 211nm

Peak#	Ret. Time	Area%
1	16.719	13.807
2	30.703	86.193
Total		100.000

Time (s): 97668

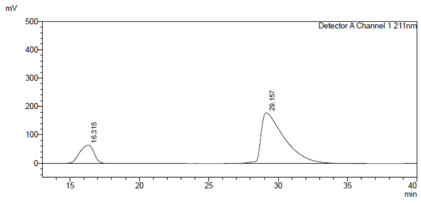

<Peak Table>

Detector A Channel 1 211nm

Peak#	Ret. Time	Area%
1	16.315	17.911
2	29.157	82.089
Total		100.000

Time (s): 153780

Racemization Kinetics of (*Rp*)-12

Following the method described by Armstrong *et al.*,[37] the chiral macrocycle product (*Rp*)-**12** was dissolved in toluene (0.010 M) and heated to 100 °C under N_2 protected, the ee value was then determined by chiral HPLC analysis. No racemization of (*Rp*)-**12** was observed after 48 h (Table S2).

Table S2. Racemization Kinetics of (*Rp*)-12.

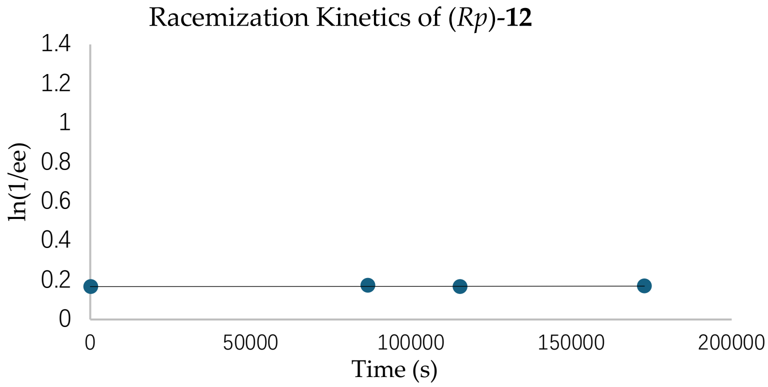

Racemization Kinetics of (*Rp*)-27

Following the method described by Armstrong *et al.*,[37] the chiral macrocycle product (*Rp*)-**27** was dissolved in toluene (0.009 M) and heated to 100 °C under N_2 protected. Determination of k_{rac} and racemization barriers $\Delta G^{\ddagger}$ of (*Rp*)-**27** at 100 °C in toluene (Table S3).

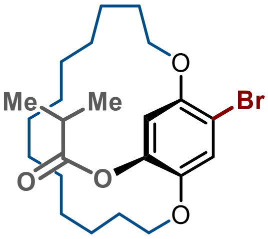

(R_p)-**27**
toluene, 100 °C
k_{rac}= 4.4 x 10^{-5} s^{-1}
ΔG_{373}= 30.3 kcal/mol

Table S3. Racemization Kinetics of (*Rp*)-27.

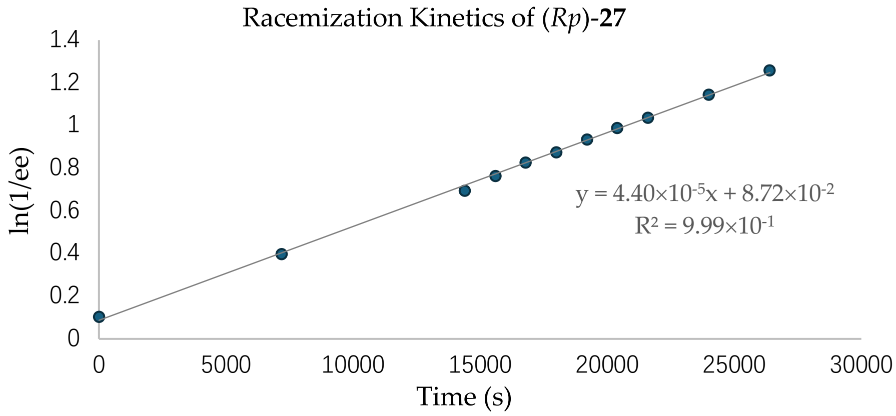

Time (s)	ln(1/ee)
0	0.104250021
7200	0.397199364
14400	0.696352312
15600	0.764428383
16800	0.827822084
18000	0.87562875
19200	0.935473551
20400	0.987786734
21600	1.037893554
24000	1.143818882
26400	1.260190484

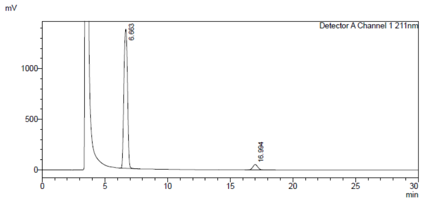

<Peak Table>

Detector A Channel 1 211nm

Peak#	Ret. Time	Area%
1	6.663	95.045
2	16.994	4.955
Total		100.000

Time (s): 0

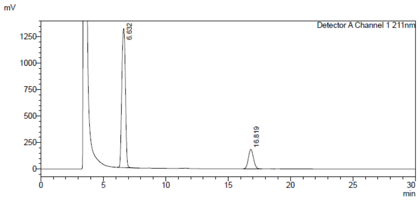

<Peak Table>

Detector A Channel 1 211nm

Peak#	Ret. Time	Area%
1	6.632	83.607
2	16.819	16.393
Total		100.000

Time (s): 7200

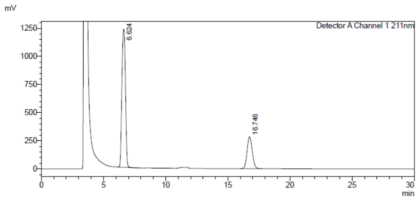

<Peak Table>

Detector A Channel 1 211nm

Peak#	Ret. Time	Area%
1	6.624	74.916
2	16.746	25.084
Total		100.000

Time (s): 14400

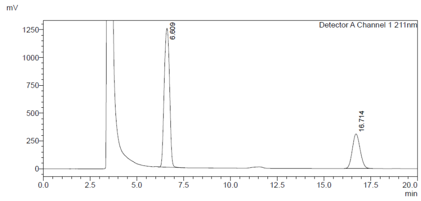

<Peak Table>

Detector A Channel 1 211nm

Peak#	Ret. Time	Area%
1	6.609	73.284
2	16.714	26.716
Total		100.000

Time (s): 15600

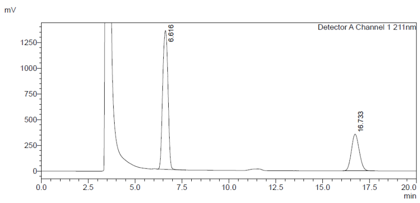

<Peak Table>

Detector A Channel 1 211nm

Peak#	Ret. Time	Area%
1	6.616	71.853
2	16.733	28.147
Total		100.000

Time (s): 16800

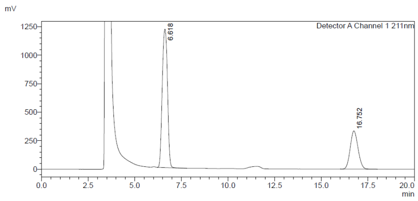

<Peak Table>

Detector A Channel 1 211nm

Peak#	Ret. Time	Area%
1	6.618	70.825
2	16.752	29.175
Total		100.000

Time (s): 18000

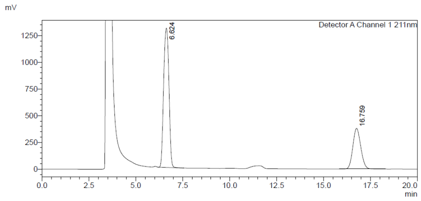

<Peak Table>

Detector A Channel 1 211nm

Peak#	Ret. Time	Area%
1	6.624	69.621
2	16.759	30.379
Total		100.000

Time (s): 19200

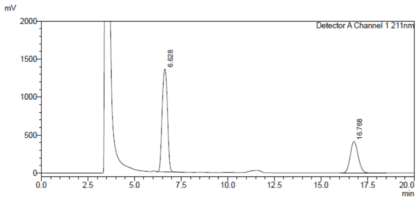

<Peak Table>

Detector A Channel 1 211nm

Peak#	Ret. Time	Area%
1	6.628	68.617
2	16.768	31.383
Total		100.000

Time (s): 20400

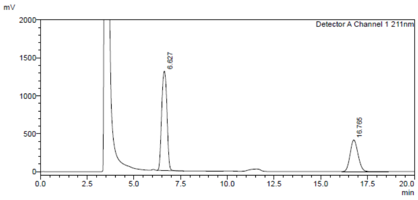

<Peak Table>

Detector A Channel 1 211nm

Peak#	Ret. Time	Area%
1	6.627	67.712
2	16.765	32.288
Total		100.000

Time (s): 21600

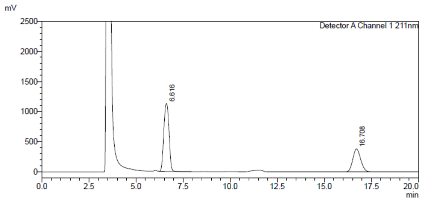

<Peak Table>

Detector A Channel 1 211nm

Peak#	Ret. Time	Area%
1	6.616	65.934
2	16.708	34.066
Total		100.000

Time (s): 24000

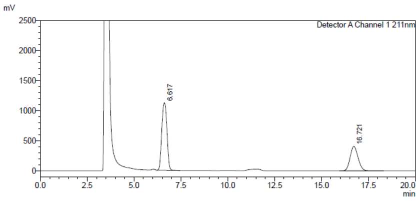

<Peak Table>

Detector A Channel 1 211nm

Peak#	Ret. Time	Area%
1	6.617	64.180
2	16.721	35.820
Total		100.000

Time (s): 26400

4 Single crystal X-ray diffraction data

X-ray diffraction data for compound **20** were collected at 100 K using a Rigaku MM-007HF High Brilliance RA generator/confocal optics [Cu Kα radiation (λ = 1.54187 Å)] with XtaLAB P100 diffractometer. Intensity data were collected (using a calculated strategy) and processed (including correction for Lorentz, polarization and absorption) using CrysAlisPro. The structure was solved by dual-space methods (SHELXT[63]) and refined by full-matrix least-squares against F^2 (SHELXL-2019/3[64]). Non-hydrogen atoms were refined anisotropically, and hydrogen atoms were refined using a riding model. All calculations were performed using the Olex2[65] interface. Selected crystallographic data are presented in Table S4.

CCDC 2432400 contains the supplementary crystallographic data for this paper. These data can be obtained free of charge from The Cambridge Crystallographic Data Centre via www.ccdc.cam.ac.uk/structures.

Table S4. Selected crystallographic data.

	(Rp)-**20**
formula	$C_{26}H_{36}O_5$
fw	428.55
crystal description	Colourless prism
crystal size [mm³]	0.16×0.13×0.04
space group	$P2_12_12_1$
a [Å]	11.05280(10)
b [Å]	11.45520(10)
c [Å]	18.6378(2)
vol [Å]³	2359.77(4)
Z	4
reflections collected	46131
independent reflections (R_{int})	0.0687
R_1 [$I > 2\sigma(I)$]	0.0625
wR_2 (all data)	0.1803
Flack parameter	0.00(10)

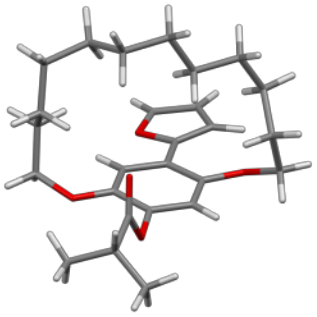

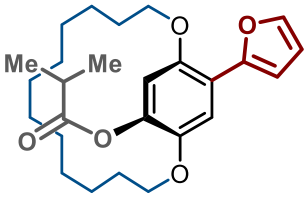

(R_p)-**20**

Datablock 20 - ellipsoid plot

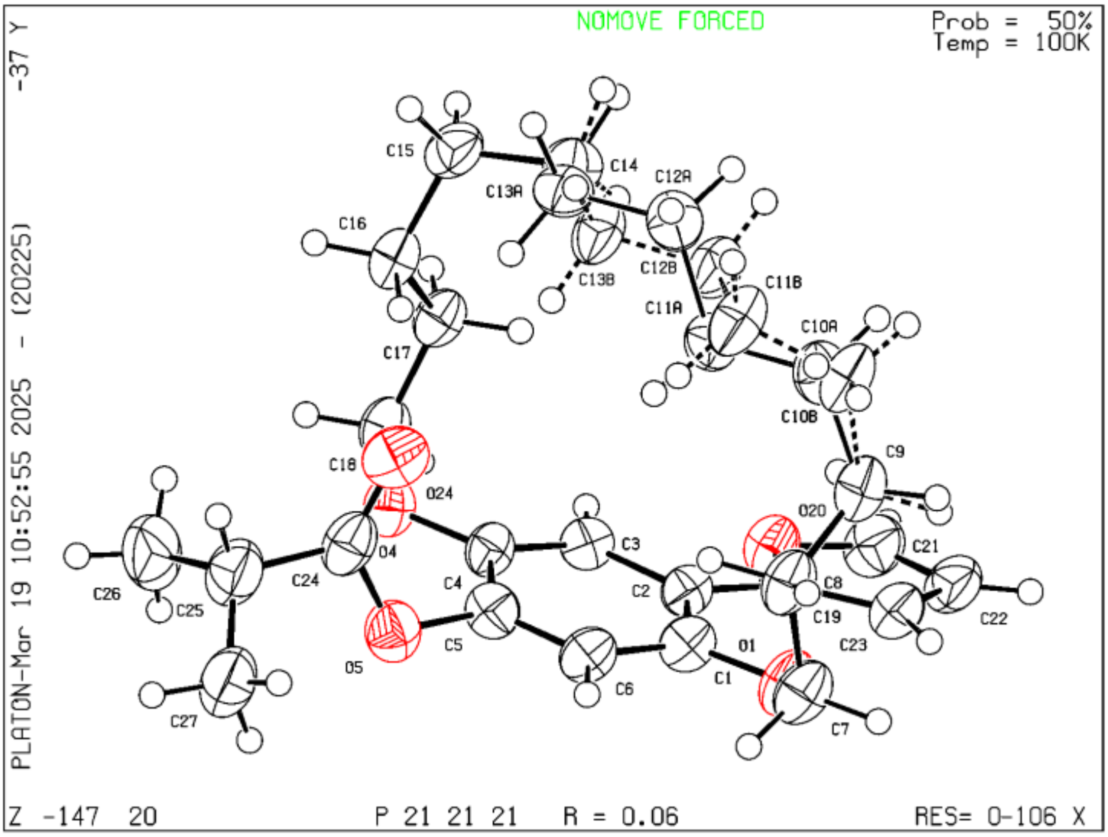

5 Reference

[1] a) W. H. Powell, *Pure Appl. Chem.* **1998**, *70*, 1513-1545; b) H. A. Favre, D. Hellwinkel, W. H. Powell, H. A. J. Smith, S. S.-C. Tsay, *Pure Appl. Chem.* **2002**, *74*, 809-834.

[2] a) V. Martí-Centelles, M. D. Pandey, M. I. Burguete, S. V. Luis, *Chem. Rev.* **2015**, *115*, 8736-8834; b) S. Kotha, M. E. Shirbhate, G. T. Waghule, *Beilstein J. Org. Chem.* **2015**, *11*, 1274-1331; c) K. Tanaka, *Bull. Chem. Soc. Jpn.* **2017**, *91*, 187-194; d) Z. Hassan, E. Spuling, D. M. Knoll, J. Lahann, S. Bräse, *Chem. Soc. Rev.* **2018**, *47*, 6947-6963; e) X. Zhang, G. Lu, M. Sun, M. Mahankali, Y. Ma, M. Zhang, W. Hua, Y. Hu, Q. Wang, J. Chen, G. He, X. Qi, W. Shen, P. Liu, G. Chen, *Nat. Chem.* **2018**, *10*, 540-548; f) P. Zhao, C. Song, in *Studies in Natural Products Chemistry, Vol. 55* (Ed.: R. Atta ur), Elsevier, **2018**, pp. 73-110; g) N. H. Thuan, T. T. An, A. Shrestha, N. X. Canh, J. K. Sohng, D. Dhakal, *Front. Chem.* **2019**, *7*; h) Z. Hassan, E. Spuling, D. M. Knoll, S. Bräse, *Angew. Chem. Int. Ed.* **2020**, *59*, 2156-2170; i) K. Ohmori, K. Suzuki, *Synlett* **2021**, *33*, 429-439; j) S. Tang, G. Vincent, *Chem. Eur. J.* **2021**, *27*, 2612-2622; k) Y. Wang, M. M. Joullié, *Chem. Rec.* **2021**, *21*, 906-923; l) P. Yang, M. J. Širvinskas, B. Li, N. W. Heller, H. Rong, G. He, A. K. Yudin, G. Chen, *J. Am. Chem. Soc.* **2023**, *145*, 13968-13978; m) Z. Dong, J. Li, C. Zhao, *Eur. J. Org. Chem.* **2024**, *27*, e202400841; n) G. Yang, J. Wang, *Angew. Chem. Int. Ed.* **2024**, *63*, e202412805; o) Y.-H. Zhao, D. Zhu, Z.-M. Chen, *ChemCatChem* **2024**, *16*, e202401312.

[3] a) D. J. Cram, J. M. Cram, *Acc. Chem. Res.* **1971**, *4*, 204-213; b) Z. Liu, S. K. M. Nalluri, J. F. Stoddart, *Chem. Soc. Rev.* **2017**, *46*, 2459-2478; c) C. L. Firme, D. M. Araújo, *Comput. Theor. Chem.* **2018**, *1135*, 18-27; d) R. López, C. Palomo, *Angew. Chem. Int. Ed.* **2022**, *61*, e202113504; e) P. Nanudorn, S. Thiengmag, F. Biermann, P. Erkoc, S. D. Dirnberger, T. N. Phan, R. Fürst, R. Ueoka, E. J. N. Helfrich, *Angew. Chem. Int. Ed.* **2022**, *61*, e202208361.

[4] a) L.-X. Dai, T. Tu, S.-L. You, W.-P. Deng, X.-L. Hou, *Acc. Chem. Res.* **2003**, *36*, 659-667; b) K. Yoshida, R. Yasue, *Chem. Eur. J.* **2018**, *24*, 18575-18586; c) L. Dai, L. Zhao, D. Xu, C. Yang, X.-K. Zhang, *Molecules* **2024**, *29*, 968.

[5] T. Focken, G. Raabe, C. Bolm, *Tetrahedron Asymmetry* **2004**, *15*, 1693-1706.

[6] G. C. Fu, *Acc. Chem. Res.* **2004**, *37*, 542-547.

[7] S. Yuan, C. Liao, W.-H. Zheng, *Org. Lett.* **2021**, *23*, 4142-4146.

[8] D. Ramaiah, P. P. Neelakandan, A. K. Nair, R. R. Avirah, *Chem. Soc. Rev.* **2010**, *39*, 4158-4168.

[9] a) T. Gulder, P. S. Baran, *Nat. Prod. Rep.* **2012**, *29*, 899-934; b) T. Q. N. Nguyen, Y. W. Tooh, R. Sugiyama, T. P. D. Nguyen, M. Purushothaman, L. C. Leow, K. Hanif, R. H. S. Yong, I. Agatha, F. R. Winnerdy, M. Gugger, A. T. Phan, B. I. Morinaka, *Nat. Chem.* **2020**, *12*, 1042-1053; c) J. A. Swain, S. R. Walker, M. B. Calvert, M. A. Brimble, *Nat. Prod. Rep.* **2022**, *39*, 410-443.

[10] a) K. R. Schramma, L. B. Bushin, M. R. Seyedsayamdost, *Nat. Chem.* **2015**, *7*, 431-437; b) P. W. Glunz, L. Mueller, D. L. Cheney, V. Ladziata, Y. Zou, N. R. Wurtz, A. Wei, P. C. Wong, R. R. Wexler, E. S. Priestley, *J. Med. Chem.* **2016**, *59*, 4007-4018; c) P. W. Glunz, *Bioorg. Med. Chem. Lett.* **2018**, *28*, 53-60; d) H. Kaur, R. P. Jakob, J. K. Marzinek, R. Green, Y. Imai, J. R. Bolla, E. Agustoni, C. V. Robinson, P. J. Bond, K. Lewis, T. Maier, S. Hiller, *Nature* **2021**, *593*, 125-129; e) G. Yao, S. Kosol, M. T. Wenz, E. Irran, B. G. Keller, O. Trapp, R. D. Süssmuth, *Nat. Commun.* **2022**, *13*, 6488.

[11] H. D. Doan, C. Rugen, C. Golz, M. Alcarazo, *Org. Lett.* **2023**, *25*, 7181-7185.

[12] a) M. Toyota, T. Yoshida, Y. Kan, S. Takaoka, Y. Asakawa, *Tetrahedron Lett.* **1996**, *37*, 4745-4748; b) D. C. Harrowven, T. Woodcock, P. D. Howes, *Angew. Chem. Int. Ed.* **2005**, *44*, 3899-3901; c) K. Harada, K. Makino, N. Shima, H. Okuyama, T. Esumi, M. Kubo, H. Hioki, Y. Asakawa, Y. Fukuyama, *Tetrahedron* **2013**, *69*, 6959-6968; d) H. Takiguchi, K. Ohmori, K. Suzuki, *Angew. Chem. Int. Ed.* **2013**, *52*, 10472-10476; e) P. Zhao, C. M. Beaudry, *Org. Lett.* **2013**, *15*, 402-405.

[13] a) A. Plaza, J. L. Keffer, G. Bifulco, J. R. Lloyd, C. A. Bewley, *J. Am. Chem. Soc.* **2010**, *132*, 9069-9077; b) C. R. Fullenkamp, Y.-P. Hsu, E. M. Quardokus, G. Zhao, C. A. Bewley, M. VanNieuwenhze, G. A.

Sulikowski, *J. Am. Chem. Soc.* **2020**, *142*, 16161-16166; c) C. A. Bewley, G. A. Sulikowski, Z. J. Yang, G. Bifulco, H.-M. Cho, C. R. Fullenkamp, *Acc. Chem. Res.* **2023**, *56*, 414-424.

[14] a) Y. Imai, K. J. Meyer, A. Iinishi, Q. Favre-Godal, R. Green, S. Manuse, M. Caboni, M. Mori, S. Niles, M. Ghiglieri, C. Honrao, X. Ma, J. J. Guo, A. Makriyannis, L. Linares-Otoya, N. Böhringer, Z. G. Wuisan, H. Kaur, R. Wu, A. Mateus, A. Typas, M. M. Savitski, J. L. Espinoza, A. O'Rourke, K. E. Nelson, S. Hiller, N. Noinaj, T. F. Schäberle, A. D'Onofrio, K. Lewis, *Nature* **2019**, *576*, 459-464; b) S. Groß, F. Panter, D. Pogorevc, C. E. Seyfert, S. Deckarm, C. D. Bader, J. Herrmann, R. Müller, *Chem. Sci.* **2021**, *12*, 11882-11893; c) A. Dutta, P. Sharma, D. Dass, V. Yarlagadda, *ACS Infect. Dis.* **2024**, *10*, 2584-2599.

[15] a) T. L. Collier, M. D. Normandin, N. A. Stephenson, E. Livni, S. H. Liang, D. W. Wooten, S. A. Esfahani, M. G. Stabin, U. Mahmood, J. Chen, W. Wang, K. Maresca, R. N. Waterhouse, G. El Fakhri, P. Richardson, N. Vasdev, *Nat. Commun.* **2017**, *8*, 15761; b) B. Li, R. W. Barnhart, J. E. Hoffman, A. Nematalla, J. Raggon, P. Richardson, N. Sach, J. Weaver, *Org. Process Res. Dev.* **2018**, *22*, 1289-1293; c) R. Dugger, B. Li, P. Richardson, in *Complete Accounts of Integrated Drug Discovery and Development: Recent Examples from the Pharmaceutical Industry Volume 2, Vol. 1332*, American Chemical Society, **2019**, pp. 27-59; d) Y. Y. Syed, *Drugs* **2019**, *79*, 93-98.

[16] a) T. Hashimoto, T. Yoshida, Y. Kan, S. Takaoka, M. Tori, Y. Asakawa, *Tetrahedron Lett.* **1994**, *35*, 909-910; b) T. Yoshida, T. Hashimoto, S. Takaoka, Y. Kan, M. Tori, Y. Asakawa, J. M. Pezzuto, T. Pengsuparp, G. A. Cordell, *Tetrahedron* **1996**, *52*, 14487-14500; c) T. Yoshida, M. Toyota, Y. Asakawa, *J. Nat. Prod.* **1997**, *60*, 145-147; d) T. Yamada, H. Takiguchi, K. Ohmori, K. Suzuki, *Org. Lett.* **2018**, *20*, 3579-3582; e) K. Sen, M. I. Khan, R. Paul, U. Ghoshal, Y. Asakawa, in *Plants, Vol. 12*, **2023**.

[17] a) T. P. Wyche, A. C. Ruzzini, L. Schwab, C. R. Currie, J. Clardy, *J. Am. Chem. Soc.* **2017**, *139*, 12899-12902; b) S. H. Reisberg, Y. Gao, A. S. Walker, E. J. N. Helfrich, J. Clardy, P. S. Baran, *Science* **2020**, *367*, 458-463.

[18] a) J. R. Knox, R. F. Pratt, *Antimicrob. Agents Chemother.* **1990**, *34*, 1342-1347; b) D. A. Evans, M. R. Wood, B. W. Trotter, T. I. Richardson, J. C. Barrow, J. L. Katz, *Angew. Chem. Int. Ed.* **1998**, *37*, 2700-2704; c) A. M. A. van Wageningen, P. N. Kirkpatrick, D. H. Williams, B. R. Harris, J. K. Kershaw, N. J. Lennard, M. Jones, S. J. M. Jones, P. J. Solenberg, *Chem. Biol.* **1998**, *5*, 155-162; d) K. C. Nicolaou, H. J. Mitchell, N. F. Jain, N. Winssinger, R. Hughes, T. Bando, *Angew. Chem. Int. Ed.* **1999**, *38*, 240-244; e) D. P. Levine, *Clin. Infect. Dis.* **2006**, *42*, S5-S12; f) R. C. Moellering, Jr., *Clin. Infect. Dis.* **2006**, *42*, S3-S4; g) M. J. Moore, S. Qu, C. Tan, Y. Cai, Y. Mogi, D. Jamin Keith, D. L. Boger, *J. Am. Chem. Soc.* **2020**, *142*, 16039-16050; h) E. Mühlberg, F. Umstätter, C. Kleist, C. Domhan, W. Mier, P. Uhl, *Can. J. Microbiol.* **2020**, *66*, 11-16.

[19] S.-J. Nam, S. P. Gaudêncio, C. A. Kauffman, P. R. Jensen, T. P. Kondratyuk, L. E. Marler, J. M. Pezzuto, W. Fenical, *J. Nat. Prod.* **2010**, *73*, 1080-1086.

[20] G. P. Moss, P. A. S. Smith, D. Tavernier, *Pure Appl. Chem.* **1995**, *67*, 1307-1375.

[21] a) K. Tanaka, T. Hori, T. Osaka, K. Noguchi, M. Hirano, *Org. Lett.* **2007**, *9*, 4881-4884; b) T. Hori, Y. Shibata, K. Tanaka, *Tetrahedron Asymmetry* **2010**, *21*, 1303-1306; c) M. Groh, D. Meidlinger, G. Bringmann, A. Speicher, *Org. Lett.* **2012**, *14*, 4548-4551; d) M. Q. Salih, C. M. Beaudry, *Org. Lett.* **2013**, *15*, 4540-4543; e) Q. Ding, Q. Wang, H. He, Q. Cai, *Org. Lett.* **2017**, *19*, 1804-1807; f) C. Gagnon, É. Godin, C. Minozzi, J. Sosoe, C. Pochet, S. K. Collins, *Science* **2020**, *367*, 917-921; g) S. Yu, G. Shen, F. He, X. Yang, *Chem. Commun.* **2022**, *58*, 7293-7296; h) S. Wei, L.-Y. Chen, J. Li, *ACS Catal.* **2023**, *13*, 7450-7456; i) L. Tan, M. Sun, H. Wang, J. Wang, J. Kim, M. Lee, *Nat. Synth.* **2023**, *2*, 1222-1231; j) X. Lv, F. Su, H. Long, F. Lu, Y. Zeng, M. Liao, F. Che, X. Wu, Y. R. Chi, *Nat. Commun.* **2024**, *15*, 958; k) J. Wang, M. Wang, Y. Wen, P. Teng, C. Li, C. Zhao, *Org. Lett.* **2024**, *26*, 1040-1045; l) G. Yang, Y. He, T. Wang, Z. Li, J. Wang, *Angew. Chem. Int. Ed.* **2024**, *63*, e202316739.

[22] a) K. Tanaka, H. Sagae, K. Toyoda, K. Noguchi, M. Hirano, *J. Am. Chem. Soc.* **2007**, *129*, 1522-1523; b) K. Tanaka, H. Sagae, K. Toyoda, M. Hirano, *Tetrahedron* **2008**, *64*, 831-846; c) T. Araki, K. Noguchi, K.

Tanaka, *Angew. Chem. Int. Ed.* **2013**, *52*, 5617-5621; d) J. Nogami, Y. Tanaka, H. Sugiyama, H. Uekusa, A. Muranaka, M. Uchiyama, K. Tanaka, *J. Am. Chem. Soc.* **2020**, *142*, 9834-9842; e) Y. Kawai, J. Nogami, Y. Nagashima, K. Tanaka, *Chem. Sci.* **2023**, *14*, 3963-3972.

[23] a) N. Kanomata, T. Nakata, *Angew. Chem. Int. Ed.* **1997**, *36*, 1207-1211; b) N. Kanomata, T. Nakata, *J. Am. Chem. Soc.* **2000**, *122*, 4563-4568; c) N. Kanomata, Y. Ochiai, *Tetrahedron Lett.* **2001**, *42*, 1045-1048; d) T. Ueda, N. Kanomata, H. Machida, *Org. Lett.* **2005**, *7*, 2365-2368; e) G. J. Rowlands, *Org. Biomol. Chem.* **2008**, *6*, 1527-1534; f) K. Mori, K. Ohmori, K. Suzuki, *Angew. Chem. Int. Ed.* **2009**, *48*, 5638-5641; g) M. Blangetti, D. F. O'Shea, *Tetrahedron Lett.* **2020**, *61*, 152492; h) S. Felder, S. Wu, J. Brom, L. Micouin, E. Benedetti, *Chirality* **2021**, *33*, 506-527.

[24] a) M. Blangetti, H. Müller-Bunz, D. F. O'Shea, *Chem. Commun.* **2013**, *49*, 6125-6127; b) D. Weinzierl, M. Waser, *Helv. Chim. Acta* **2021**, *104*, e2100073.

[25] a) K. Kanda, K. Endo, T. Shibata, *Org. Lett.* **2010**, *12*, 1980-1983; b) T. Shibata, M. Fukai, R. Sekine, M. Hazra, K. S. Kanyiva, *Synthesis* **2016**, *48*, 2664-2670; c) Y. An, X.-Y. Zhang, Y.-N. Ding, Y. Li, X.-Y. Liu, Y.-M. Liang, *Org. Lett.* **2022**, *24*, 7294-7299.

[26] a) K. Kanda, T. Koike, K. Endo, T. Shibata, *Chem. Commun.* **2009**, 1870-1872; b) K. Mori, H. Kishi, T. Akiyama, *Synthesis* **2017**, *49*, 365-370; c) K. Akagawa, J. Higuchi, I. Yoshikawa, K. Kudo, *Eur. J. Org. Chem.* **2018**, *2018*, 5278-5281; d) D. Wang, Y.-B. Shao, Y. Chen, X.-S. Xue, X. Yang, *Angew. Chem. Int. Ed.* **2022**, *61*, e202201064; e) Z. Dong, J. Li, T. Yao, C. Zhao, *Angew. Chem. Int. Ed.* **2023**, *62*, e202315603; f) J. Li, C. Zhao, *ACS Catal.* **2023**, *13*, 14155-14162; g) S. Yu, H. Bao, D. Zhang, X. Yang, *Nat. Commun.* **2023**, *14*, 5239; h) C.-Y. Guan, S. Zou, C. Luo, Z.-Y. Li, M. Huang, L. Huang, X. Xiao, D. Wei, M.-C. Wang, G.-J. Mei, *Nat. Commun.* **2024**, *15*, 4580; i) J. Li, Z. Dong, Y. Chen, Z. Yang, X. Yan, M. Wang, C. Li, C. Zhao, *Nat. Commun.* **2024**, *15*, 2338; j) Q. Liu, K. Teng, Y. Zhang, Y. Lv, Y. R. Chi, Z. Jin, *Angew. Chem. Int. Ed.* **2024**, *63*, e202406386; k) D. Zhu, T. Mu, Z.-L. Li, H.-Y. Luo, R.-F. Cao, X.-S. Xue, Z.-M. Chen, *Angew. Chem. Int. Ed.* **2024**, *63*, e202318625; l) S. Huh, E. Linne, L. Estaque, G. Pieters, M. Devereux, O. Baudoin, *Angew. Chem. Int. Ed.* **2025**, e202500653; m) J. Li, Z. Dong, S. Liu, X. Liu, C. Zhao, *Chem. Eur. J.* **2025**, *n/a*, e202404610; n) Z. Wu, S. Fang, J. He, J. Che, Z. Liu, X. Wei, Z. Su, T. Wang, *Angew. Chem. Int. Ed.* **2025**, *64*, e202423702.

[27] D. Weinzierl, M. Waser, *Beilstein J. Org. Chem.* **2021**, *17*, 800-804.

[28] a) S. E. Potter, I. O. Sutherland, *J. Chem. Soc., Chem. Commun.* **1972**, 754-755; b) A. Bacchi, G. Pelizzi, *J. Comput. Aided Mol. Des.* **1999**, *13*, 385-396; c) K. Sakamoto, M. Oki, *Bull. Chem. Soc. Jpn.* **2006**, *50*, 3388-3392.

[29] a) John M. Keith, Jay F. Larrow, Eric N. Jacobsen, *Adv. Synth. Catal.* **2001**, *343*, 5-26; b) E. Vedejs, M. Jure, *Angew. Chem. Int. Ed.* **2005**, *44*, 3974-4001.

[30] a) A. C. Spivey, S. Arseniyadis, in *Asymmetric Organocatalysis* (Ed.: B. List), Springer Berlin Heidelberg, Berlin, Heidelberg, **2009**, pp. 233-280; b) N. De Rycke, F. Couty, O. R. P. David, *Chem. Eur. J.* **2011**, *17*, 12852-12871; c) H. Pellissier, *Adv. Synth. Catal.* **2011**, *353*, 1613-1666; d) in *Stereoselective Synthesis 3, Vol. Volume 3*, 1st Edition ed., Georg Thieme Verlag KG, Stuttgart, **2011**.

[31] A. S. Burns, A. J. Wagner, J. L. Fulton, K. Young, A. Zakarian, S. D. Rychnovsky, *Org. Lett.* **2017**, *19*, 2953-2956.

[32] a) V. B. Birman, *Aldrichim. Acta* **2016**, *49*, 23-41; b) J. Merad, J.-M. Pons, O. Chuzel, C. Bressy, *Eur. J. Org. Chem.* **2016**, *2016*, 5589-5610; c) J. Seliger, M. Oestreich, *Chem. Eur. J.* **2019**, *25*, 9358-9365; d) T. Zhang, B. K. Redden, S. L. Wiskur, *Eur. J. Org. Chem.* **2019**, *2019*, 4827-4831; e) Z. Gong, A. Smith, A. O. Farah, S. D. Dickerson, G. A. González-Montiel, J. M. Laddusaw, P. H.-Y. Cheong, S. L. Wiskur, *J. Org. Chem.* **2023**, *88*, 16898-16905; f) C. J. Harrison, S. D. Dickerson, Z. Gong, A. S. McGowan, J. Vista, S. L. Wiskur, *Eur. J. Org. Chem.* **2024**, *27*, e202400641.

[33] B. Ding, Q. Xue, S. Jia, H.-G. Cheng, Q. Zhou, *Synthesis* **2022**, *54*, 1721-1732.

[34] V. B. Birman, X. Li, *Org. Lett.* **2006**, *8*, 1351-1354.

[35] E. S. Munday, M. A. Grove, T. Feoktistova, A. C. Brueckner, D. M. Walden, C. M. Young, A. M. Z. Slawin, A. D. Campbell, P. H.-Y. Cheong, A. D. Smith, *Angew. Chem. Int. Ed.* **2020**, *59*, 7897-7905.

[36] S. Qu, M. D. Greenhalgh, A. D. Smith, *Chem. Eur. J.* **2019**, *25*, 2816-2823.

[37] J.-P. Heeb, J. Clayden, M. D. Smith, R. J. Armstrong, *Nat. Protoc.* **2023**, *18*, 2745-2771.

[38] H. B. Kagan, J. C. Fiaud, in *Topics in Stereochemistry*, **1988**, pp. 249-330.

[39] M. D. Greenhalgh, J. E. Taylor, A. D. Smith, *Tetrahedron* **2018**, *74*, 5554-5560.

[40] The X-ray structural data can be obtained from The Cambridge Crystallographic Data Centre (www.ccdc.cam.ac.uk/structures) as deposition number 2432400.

[41] a) I. Shiina, K. Nakata, K. Ono, Y.-S. Onda, M. Itagaki, *J. Am. Chem. Soc.* **2010**, *132*, 11629-11641; b) I. Shiina, K. Ono, K. Nakata, *Chemistry Lett.* **2011**, *40*, 147-149; c) K. Nakata, A. Sekiguchi, I. Shiina, *Tetrahedron Asymmetry* **2011**, *22*, 1610-1619; d) P. Liu, X. Yang, V. B. Birman, K. N. Houk, *Org. Lett.* **2012**, *14*, 3288-3291; e) X. Yang, P. Liu, K. N. Houk, V. B. Birman, *Angew. Chem. Int. Ed.* **2012**, *51*, 9638-9642; f) K. Nakata, K. Gotoh, K. Ono, K. Futami, I. Shiina, *Org. Lett.* **2013**, *15*, 1170-1173; g) I. Shiina, K. Ono, T. Nakahara, *Chem. Commun.* **2013**, *49*, 10700-10702; h) M. D. Greenhalgh, S. M. Smith, D. M. Walden, J. E. Taylor, Z. Brice, E. R. T. Robinson, C. Fallan, D. B. Cordes, A. M. Z. Slawin, H. C. Richardson, M. A. Grove, P. H.-Y. Cheong, A. D. Smith, *Angew. Chem. Int. Ed.* **2018**, *57*, 3200-3206; i) T. Murata, T. Kawanishi, A. Sekiguchi, R. Ishikawa, K. Ono, K. Nakata, I. Shiina, *Molecules* **2018**, *23*, 2003; j) T. Desrues, J. Merad, D. Andrei, J.-M. Pons, J.-L. Parrain, M. Médebielle, A. Quintard, C. Bressy, *Angew. Chem. Int. Ed.* **2021**, *60*, 24924-24929; k) S. M. Smith, M. D. Greenhalgh, T. Feoktistova, D. M. Walden, J. E. Taylor, D. B. Cordes, A. M. Z. Slawin, P. H.-Y. Cheong, A. D. Smith, *Eur. J. Org. Chem.* **2022**, *2022*, e202101111; l) S. K. Agrawal, P. K. Majhi, A. S. Goodfellow, R. K. Tak, D. B. Cordes, A. P. McKay, K. Kasten, M. Bühl, A. D. Smith, *Angew. Chem. Int. Ed.* **2024**, *63*, e202402909; m) H. Zhu, A. Manchado, A. Omar Farah, A. P. McKay, D. B. Cordes, P. H.-Y. Cheong, K. Kasten, A. D. Smith, *Angew. Chem. Int. Ed.* **2024**, *63*, e202402908.

[42] a) C. Bleiholder, R. Gleiter, D. B. Werz, H. Köppel, *Inorg. Chem.* **2007**, *46*, 2249-2260; b) R. Gleiter, G. Haberhauer, D. B. Werz, F. Rominger, C. Bleiholder, *Chem. Rev.* **2018**, *118*, 2010-2041; c) S. Kolb, G. A. Oliver, D. B. Werz, *Angew. Chem. Int. Ed.* **2020**, *59*, 22306-22310.

[43] a) C. E. Cannizzaro, K. N. Houk, *J. Am. Chem. Soc.* **2002**, *124*, 7163-7169; b) S. Xu, I. Held, B. Kempf, H. Mayr, W. Steglich, H. Zipse, *Chem. Eur. J.* **2005**, *11*, 4751-4757; c) V. Lutz, J. Glatthaar, C. Würtele, M. Serafin, H. Hausmann, P. R. Schreiner, *Chem. Eur. J.* **2009**, *15*, 8548-8557; d) E. Larionov, M. Mahesh, A. C. Spivey, Y. Wei, H. Zipse, *J. Am. Chem. Soc.* **2012**, *134*, 9390-9399; e) R. C. Johnston, P. H.-Y. Cheong, *Org. Biomol. Chem.* **2013**, *11*, 5057-5064.

[44] a) Q. Xu, H. Zhou, X. Geng, P.-R. Chen, *Tetrahedron* **2009**, *65*, 2232-2238; b) P.-R. Chen, Y. Zhang, H. Zhou, Q. Xu, *Acta Chim. Sinica* **2010**, *68*, 1431-1436; c) D. Belmessieri, C. Joannesse, P. A. Woods, C. MacGregor, C. Jones, C. D. Campbell, C. P. Johnston, N. Duguet, C. Concellón, R. A. Bragg, A. D. Smith, *Org. Biomol. Chem.* **2011**, *9*, 559-570; d) X. Li, H. Jiang, E. W. Uffman, L. Guo, Y. Zhang, X. Yang, V. B. Birman, *J. Org. Chem.* **2012**, *77*, 1722-1737; e) S. F. Musolino, O. S. Ojo, N. J. Westwood, J. E. Taylor, A. D. Smith, *Chem. Eur. J.* **2016**, *22*, 18916-18922.

[45] I. Shiina, K. Nakata, K. Ono, M. Sugimoto, A. Sekiguchi, *Chem. Eur. J.* **2010**, *16*, 167-172.

[46] a) A. F. Burchat, J. M. Chong, N. Nielsen, *J. Organomet. Chem.* **1997**, *542*, 281-283; b) A. Krasovskiy, P. Knochel, *Synthesis* **2006**, *2006*, 0890-0891.

[47] *Pure Appl. Chem.* **1974**, *37*, 445-462.

[48] W. C. Still, M. Kahn, A. Mitra, *J. Org. Chem.* **1978**, *43*, 2923-2925.

[49] G. P. Moss, *Pure Appl. Chem.* **1996**, *68*, 2193-2222.

[50] J. E. Bertie, *Pure Appl. Chem.* **1998**, *70*, 2039-2045.

[51] G. R. Fulmer, A. J. M. Miller, N. H. Sherden, H. E. Gottlieb, A. Nudelman, B. M. Stoltz, J. E. Bercaw, K. I. Goldberg, *Organomet.* **2010**, *29*, 2176-2179.

[52] a) *Pure Appl. Chem.* **1972**, *29*, 625-628; b) *Pure Appl. Chem.* **1976**, *45*, 217-220.
[53] J. F. J. Todd, *Pure Appl. Chem.* **1991**, *63*, 1541-1566.
[54] M. A. Brimble, D. S. Black, R. Hartshorn, A. P. Rauter, C.-K. Sha, L. K. Sydnes, *Pure Appl. Chem.* **2013**, *85*, 307-313.
[55] a) H. A. Favre, W. H. Powell, in *Nomenclature of Organic Chemistry. IUPAC Recommendations and Preferred Names 2013.*, The Royal Society of Chemistry, **2013**; b) R. S. Cahn, C. K. Ingold, V. Prelog, *Experientia* **1956**, *12*, 81-94; c) *Pure Appl. Chem.* **1965**, *11*, 1-260.
[56] J. I. Choi, E. S. An, in *WO2014104861A1, Vol. WO2014104861A1* (Ed.: W. P. Organization), Korea Research Institute of Chemical Technology, Korea, **2014**.
[57] B. Huang, S. M. Santos, V. Felix, P. D. Beer, *Chem. Commun.* **2008**, 4610-4612.
[58] P.-Y. Yang, H. Zou, E. Chao, L. Sherwood, V. Nunez, M. Keeney, E. Ghartey-Tagoe, Z. Ding, H. Quirino, X. Luo, G. Welzel, G. Chen, P. Singh, A. K. Woods, P. G. Schultz, W. Shen, *Proc. Nat. Acad. Sci.* **2016**, *113*, 4140-4145.
[59] P. C. Bulman Page, Y. Chan, A. H. Noor Armylisas, M. Alahmdi, *Tetrahedron* **2016**, *72*, 8406-8416.
[60] C. Belle, C. Bougault, M.-T. Averbuch, A. Durif, J.-L. Pierre, J.-M. Latour, L. Le Pape, *J. Am. Chem. Soc.* **2001**, *123*, 8053-8066.
[61] M. Wijtmans, D. A. Pratt, J. Brinkhorst, R. Serwa, L. Valgimigli, G. F. Pedulli, N. A. Porter, *J. Org. Chem.* **2004**, *69*, 9215-9223.
[62] M. Abid, R. Nouch, T. D. Bradshaw, W. Lewis, S. Woodward, *Eur. J. Inorg. Chem.* **2019**, *2019*, 2774-2780.
[63] G. M. Sheldrick, *Acta Crystallogr., Sect . A: Found. Crystallogr.* **2015**, *71*, 3-8.
[64] G. M. Sheldrick, *Acta Crystallogr., Sect. C: Cryst. Struct. Commun.* **2015**, *71*, 3-8.
[65] O. V. Dolomanov, L. J. Bourhis, R. J. Gildea, J. A. Howard, H. Puschmann, *Appl. Crystallogr.* **2009**, *42*, 339-341.

Appendix I: ^{1}H, $^{13}C\{^{1}H\}$, 2D ^{1}H COSY, 2D ^{1}H–^{13}C HSQC and 2D ^{1}H–^{13}C HMBC, NOESY, NMR Spectra

O,O′-bis(2-(α-bromoacetylamino)ethyl)catechol S9

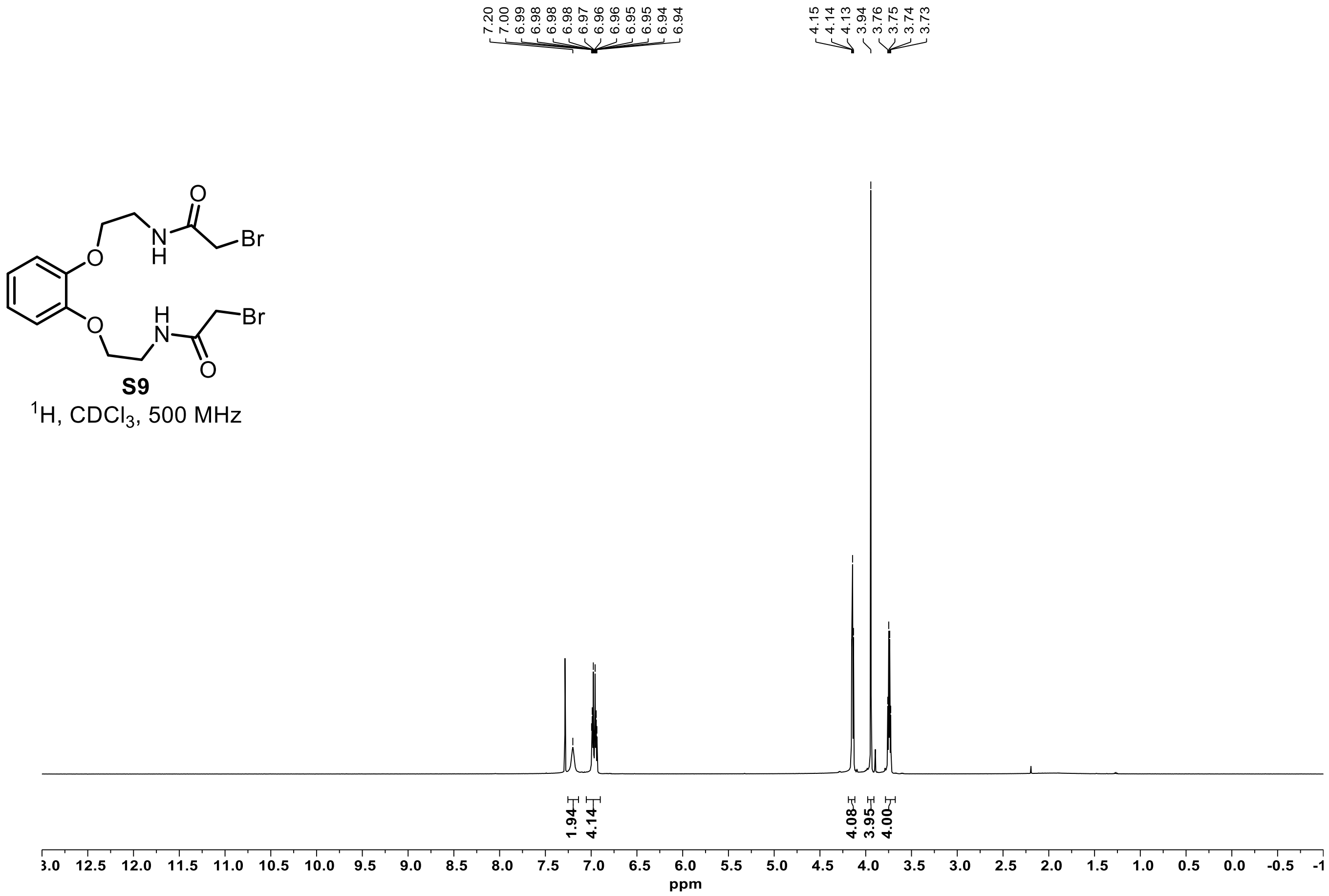

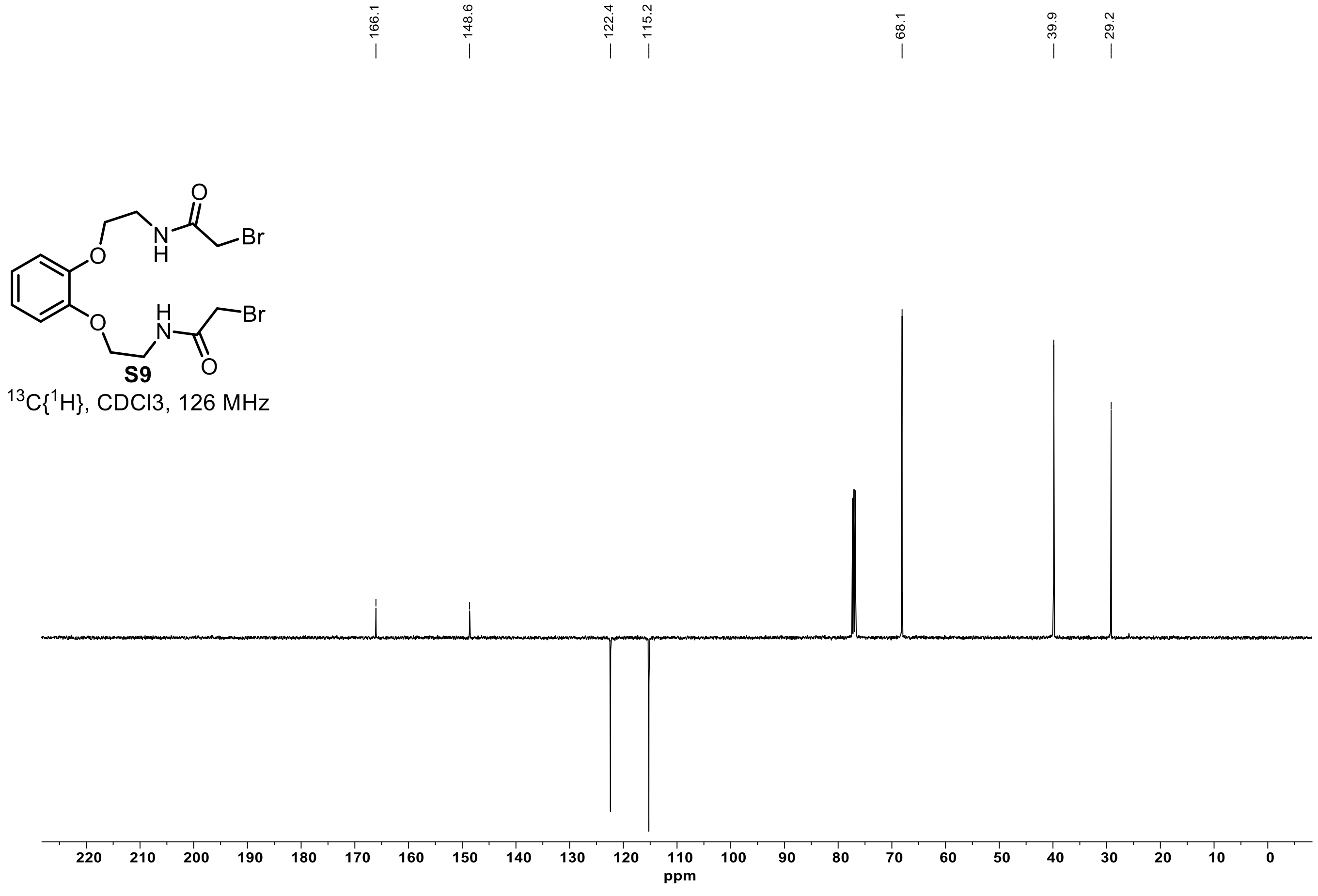

1²-bromo-1⁵-formyl-2,6,11,15-tetraoxa-1(1,4)-benzenacyclopentadecaphane S13

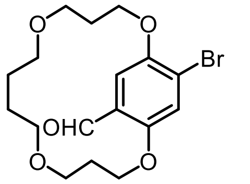

S13

^{1}H, $CDCl_3$, 500 MHz

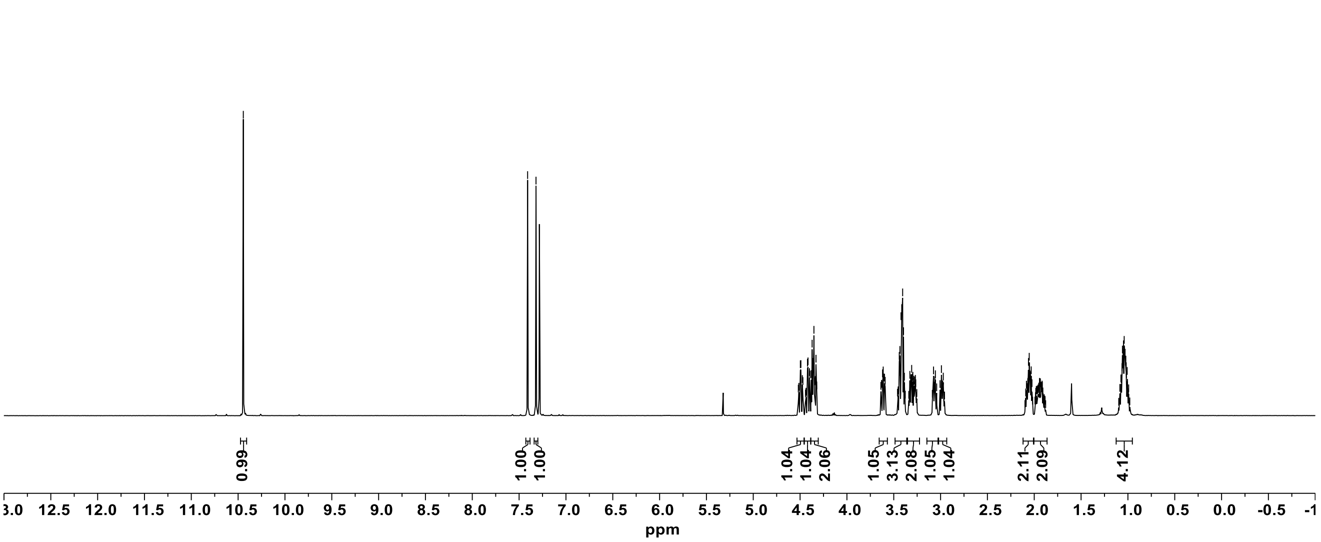

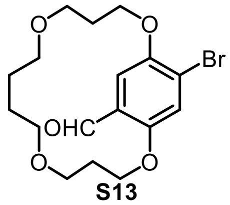

S13

$^{13}C\{^{1}H\}$, $CDCl_3$, 126 MHz

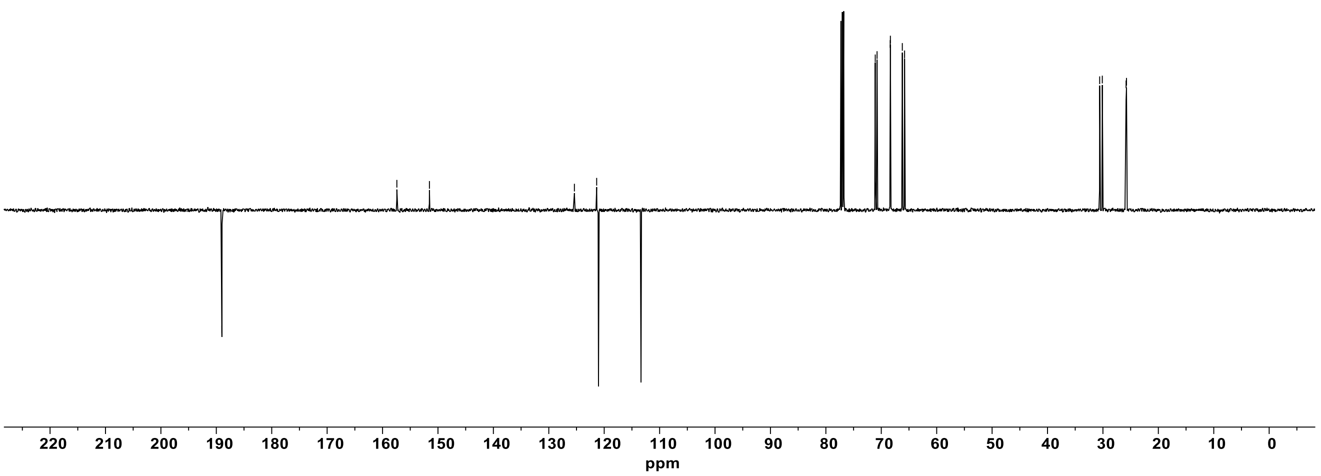

Synthesis of 1^2-bromo-1^5-formyl-4,15-dioxo-2,17-dioxa-5,14-diaza-1(1,4)-benzenacycloheptadecaphane S18

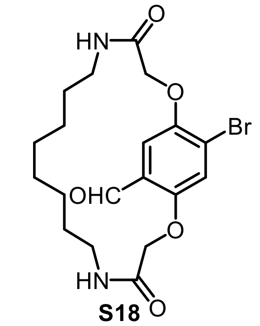

S18

^{1}H, $CDCl_3$, 500 MHz

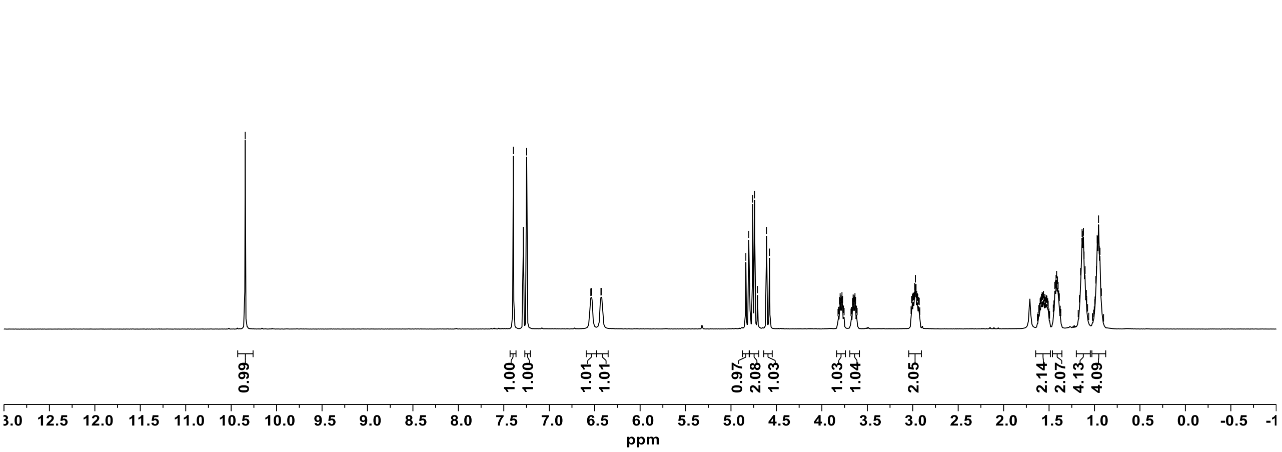

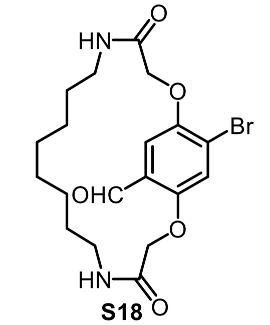

S18

$^{13}C\{^1H\}$, $CDCl_3$, 126 MHz

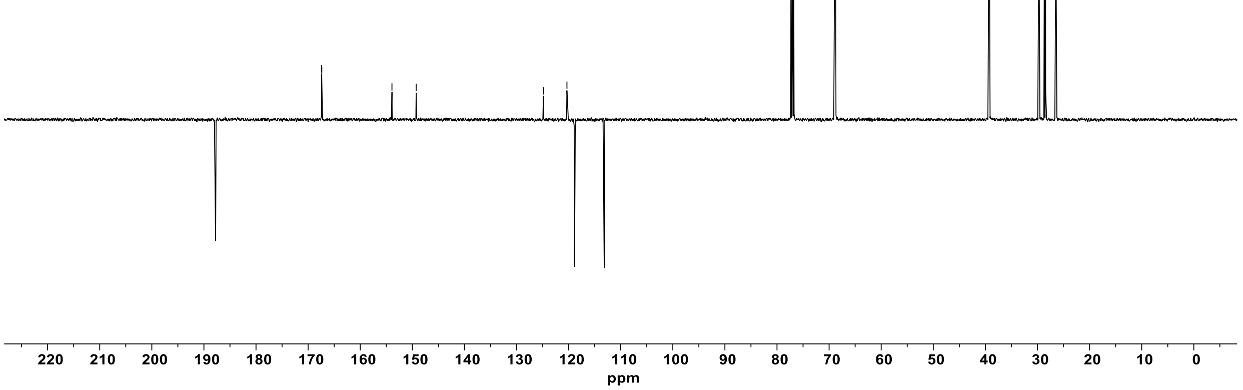

1²-formyl-4,14-dioxo-1⁵-phenyl-5,13-diaza-2,8,10,16-tetraoxa-1(1,2),9(1,4)-dibenzena-cyclohexadecaphane

S19

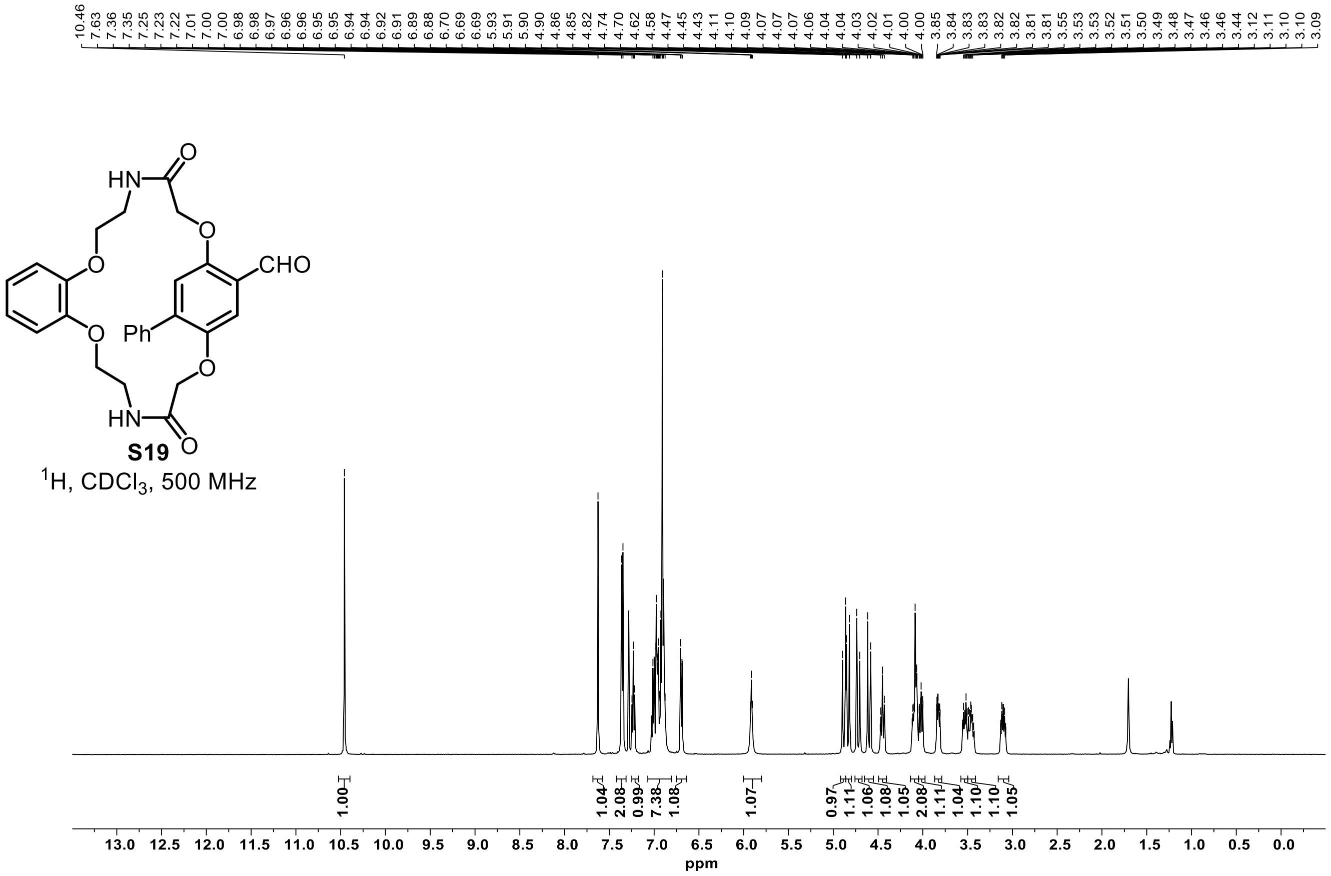

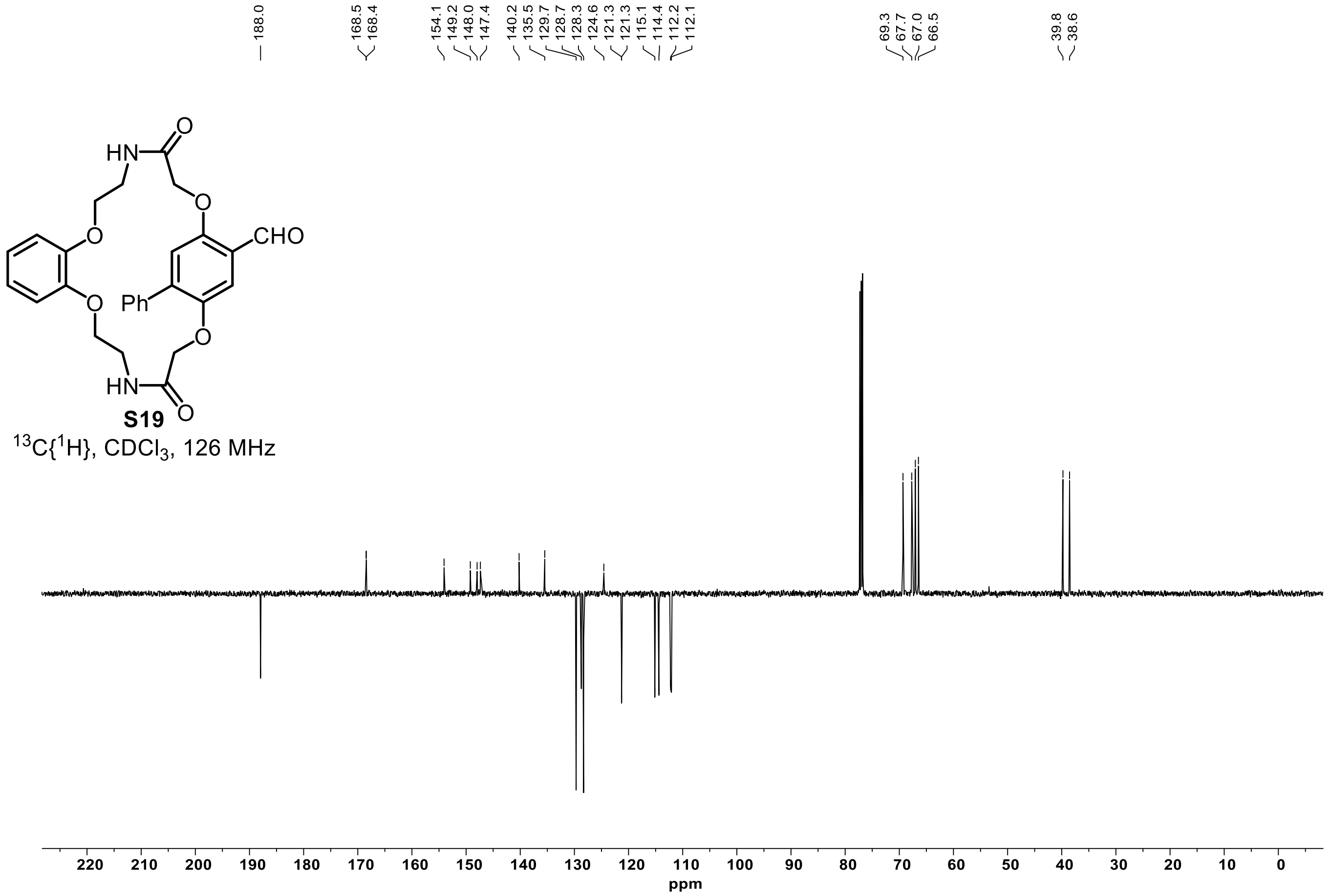

1^5-phenyl-2,14-dioxa-1(1,4)-benzenacyclotridecaphane-1^2-carbaldehyde S21

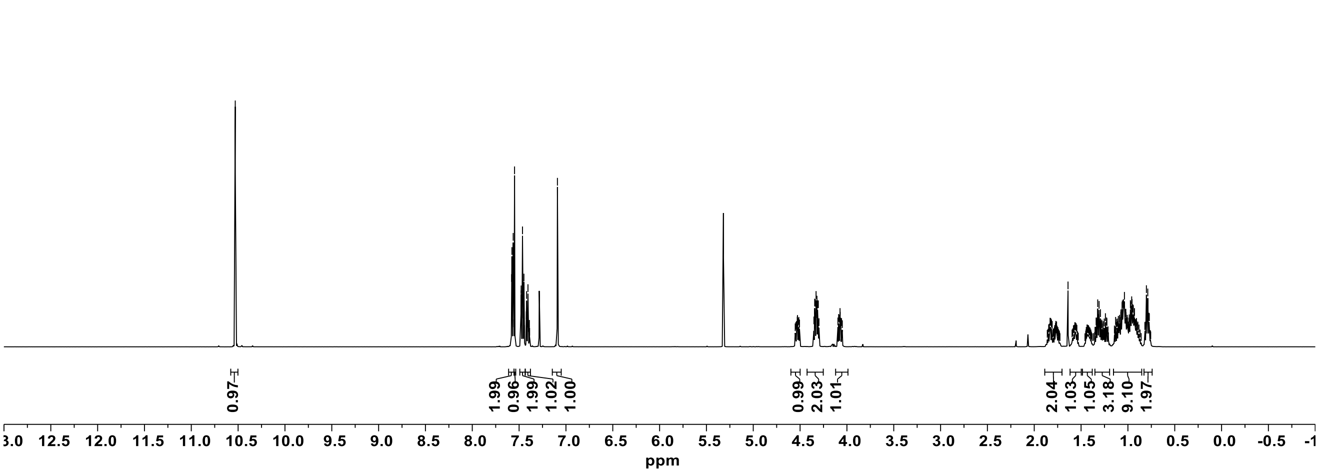

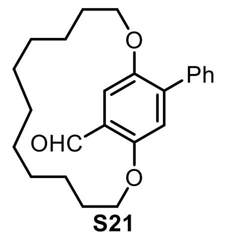

S21

^{1}H, $CDCl_3$, 500 MHz

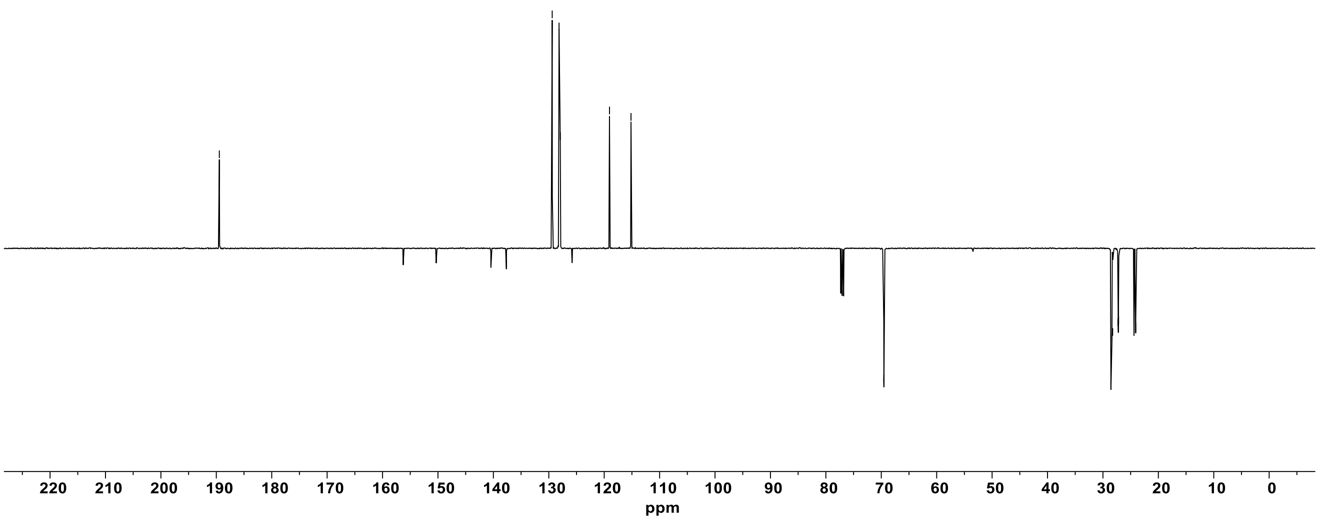

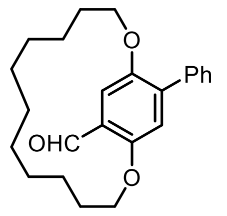

S21

$^{13}C\{^1H\}$, $CDCl_3$, 126 MHz

1[5]-(4-methoxyphenyl)-2,15-dioxa-1(1,4)-benzenacyclopentadecaphane-1[2]-carbaldehyde S23

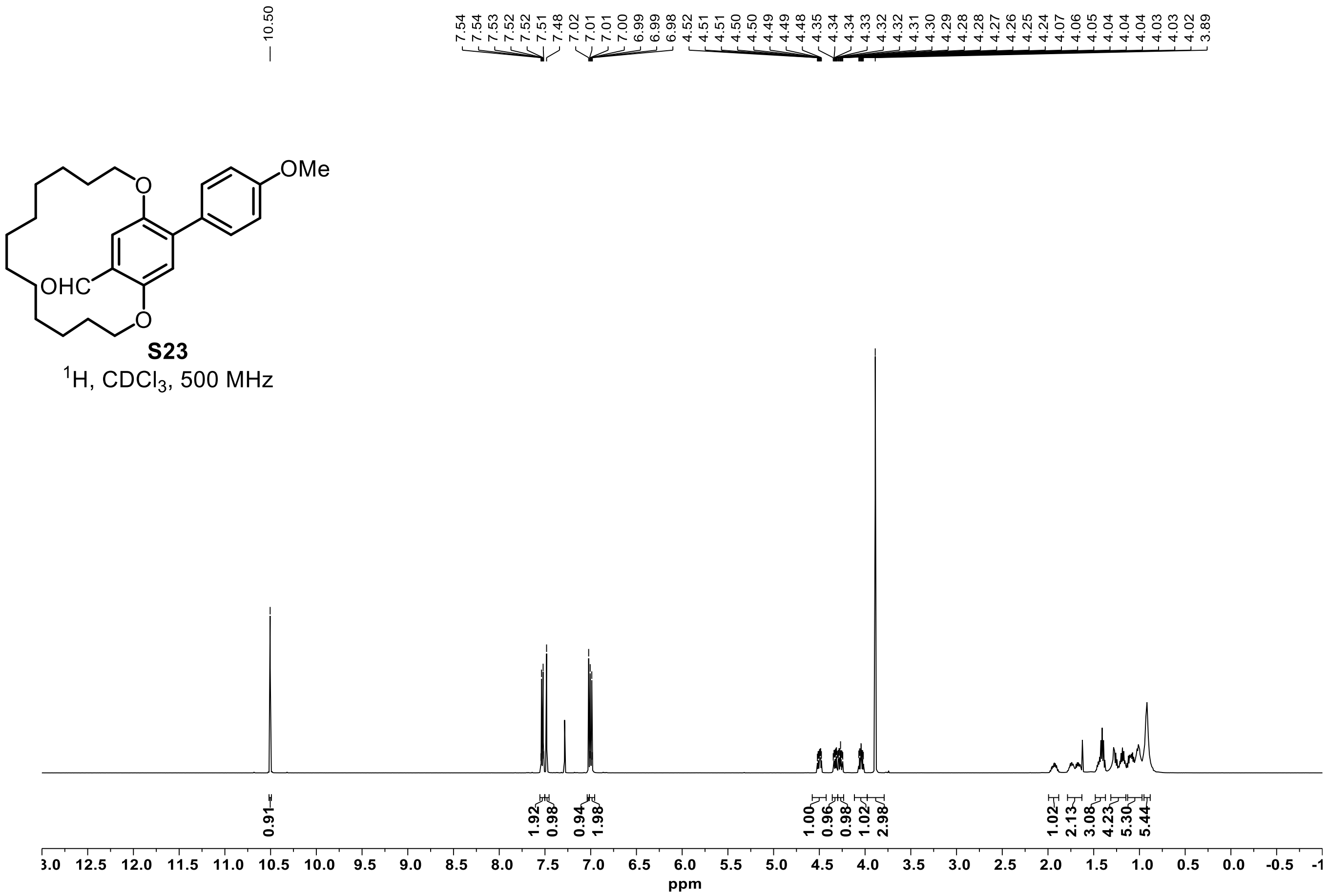

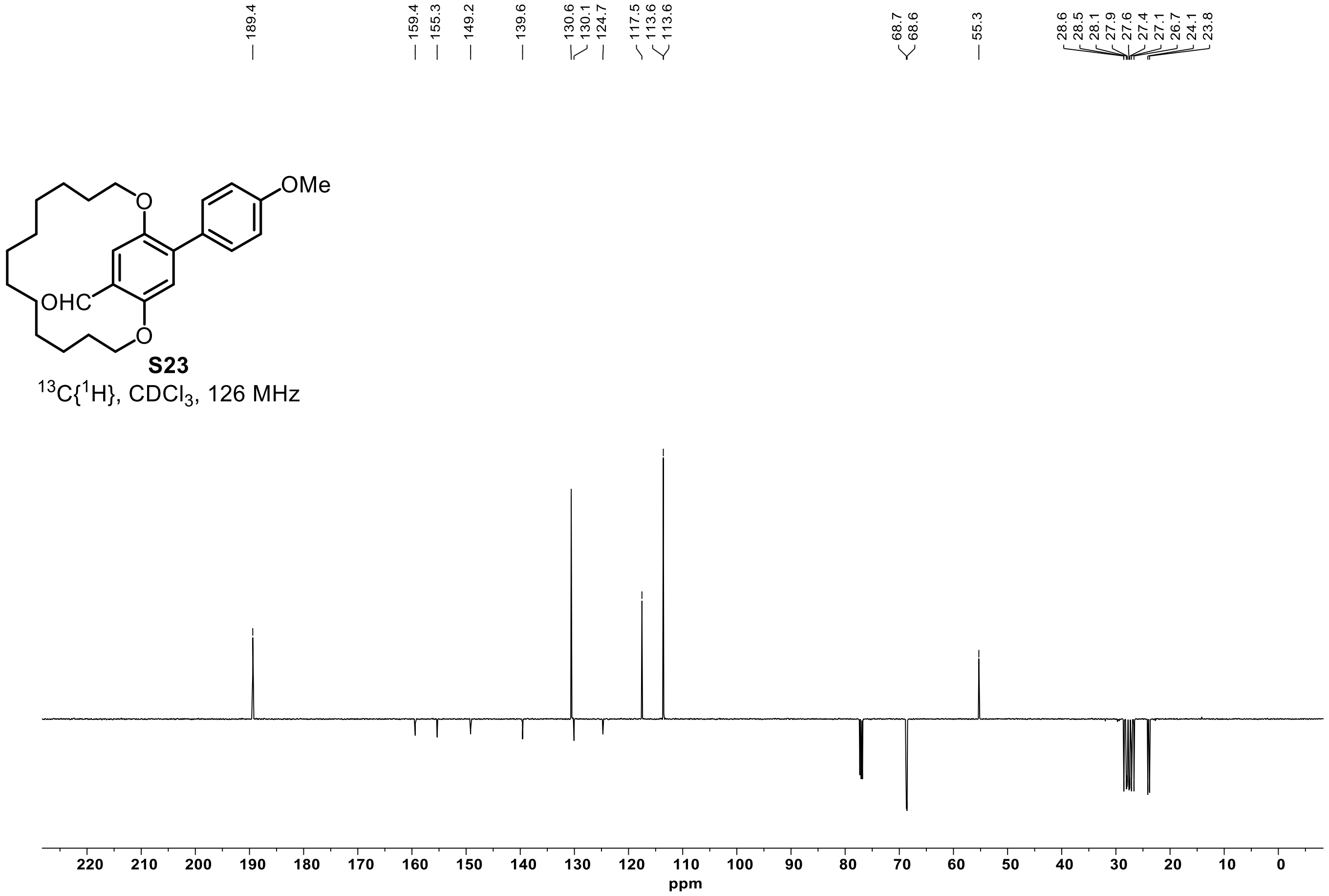

1⁵-(4-(trifluoromethyl)phenyl)-2,15-dioxa-1(1,4)-benzenacyclopentadecaphane-1²-carbaldehyde S24

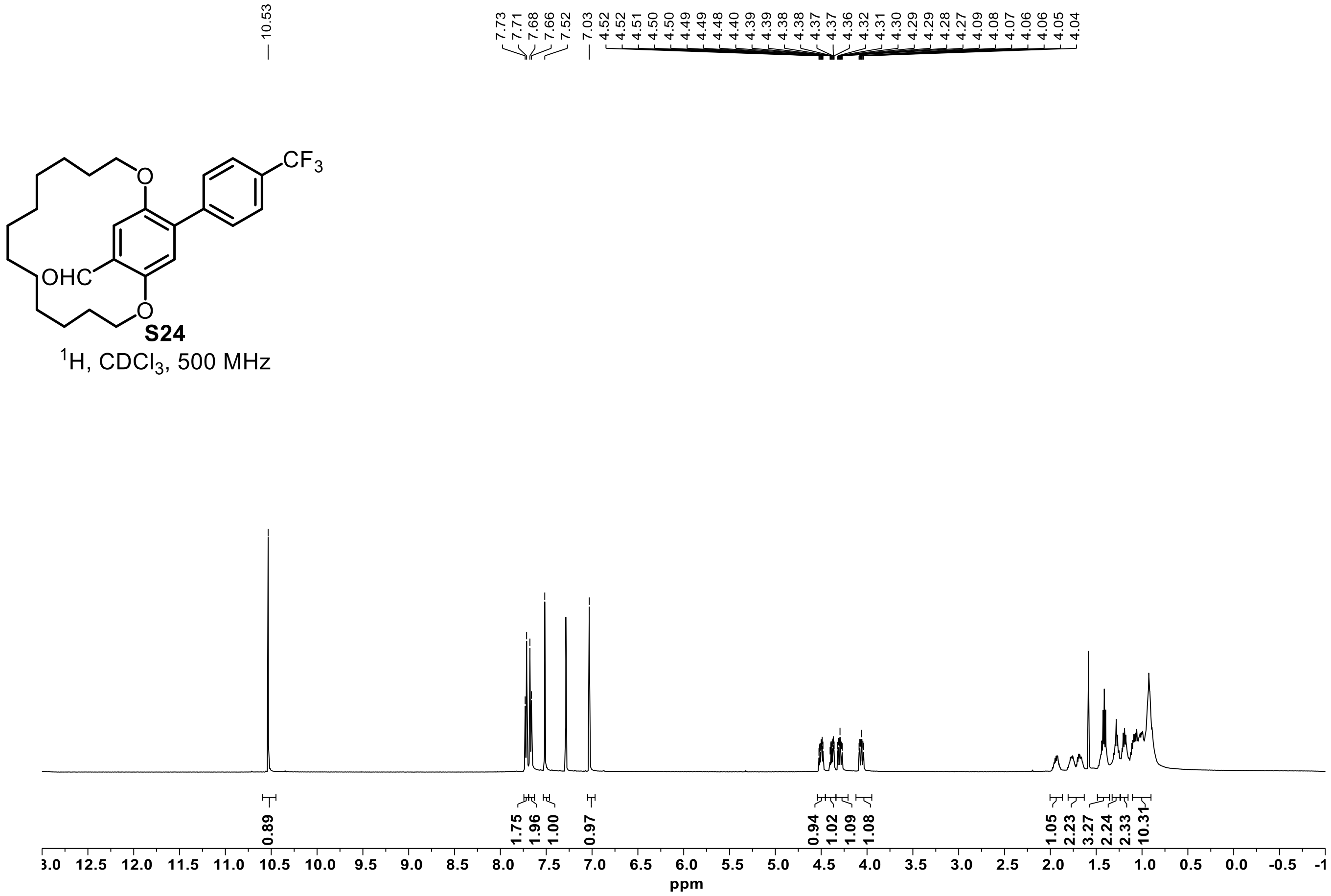

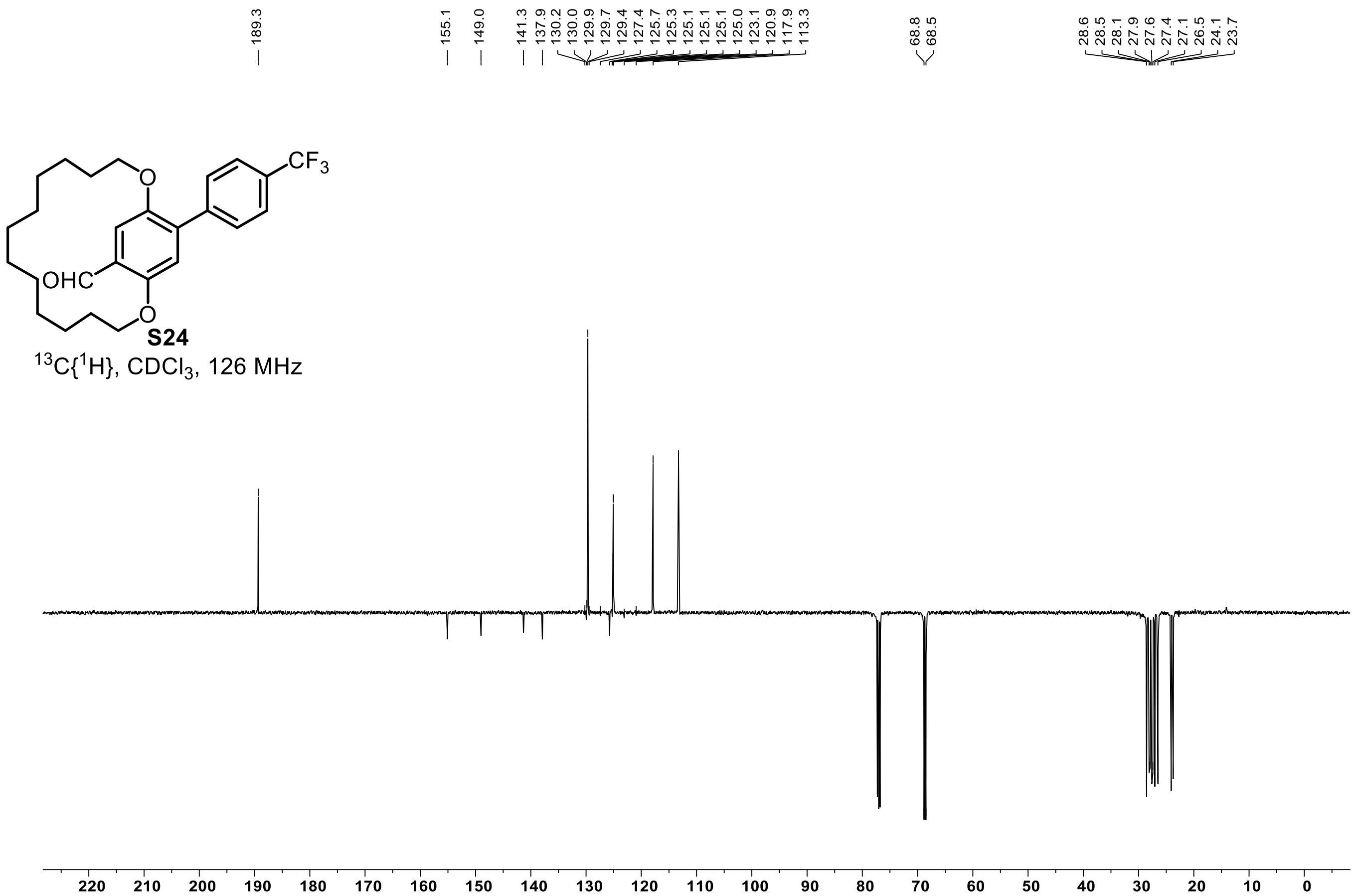

1⁵-(2-methoxyphenyl)-2,15-dioxa-1(1,4)-benzenacyclopentadecaphane-1²-carbaldehyde S25

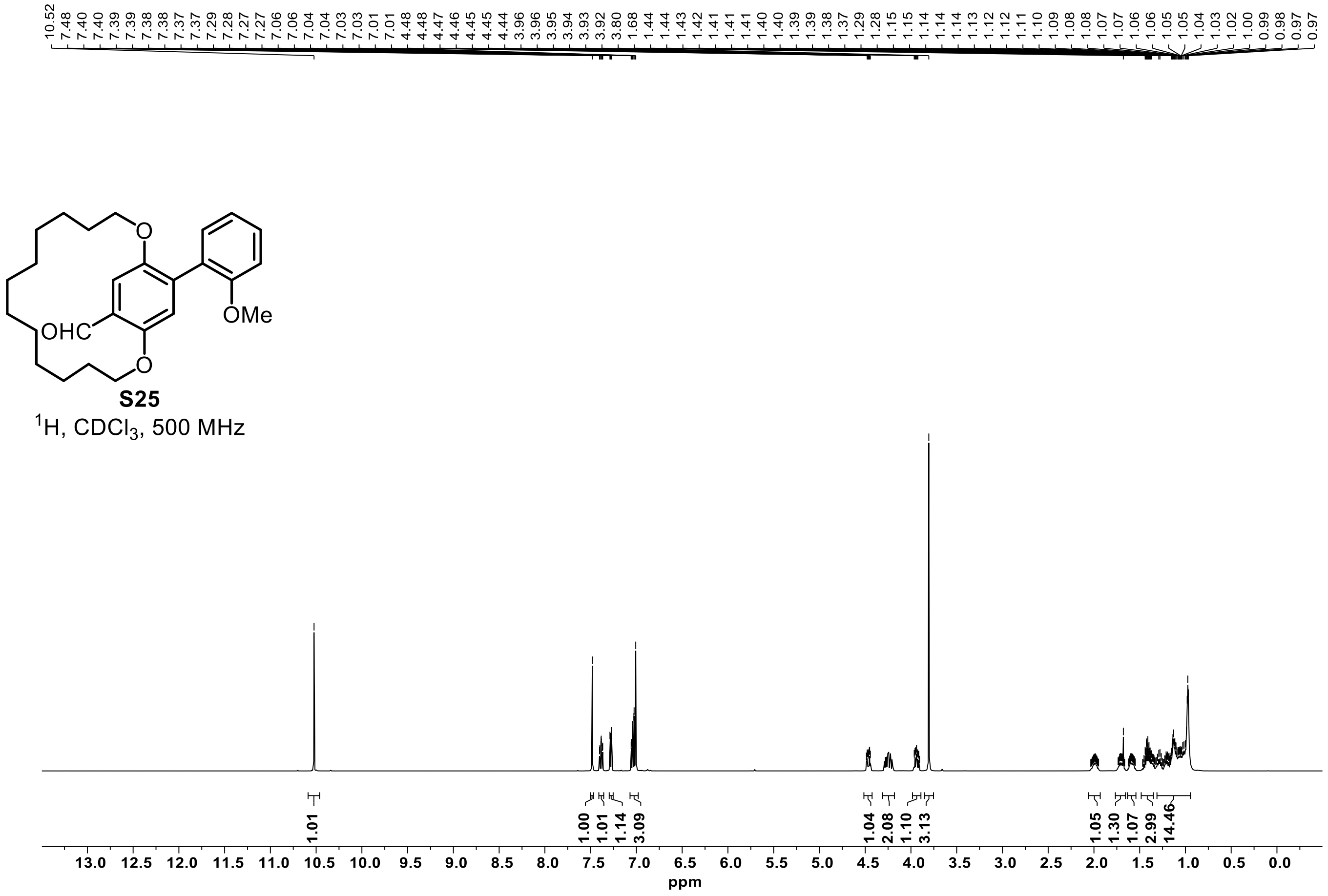

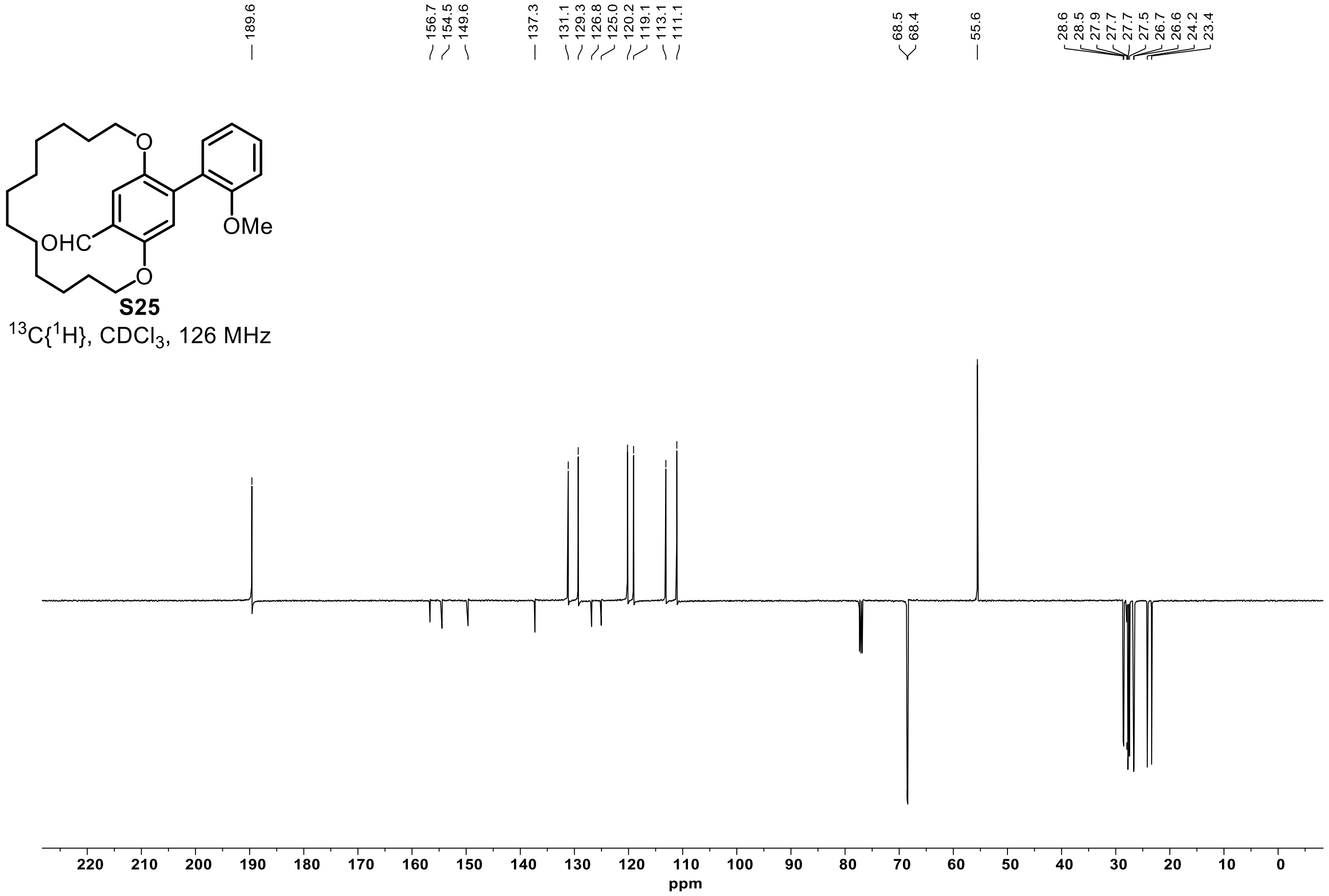

1⁵-(naphthalen-1-yl)-2,15-dioxa-1(1,4)-benzenacyclopentadecaphane-1²-carbaldehyde S26

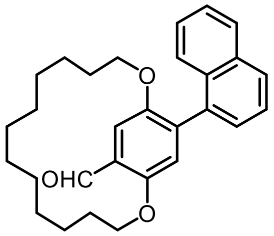

S26

^{1}H, $CDCl_3$, 500 MHz

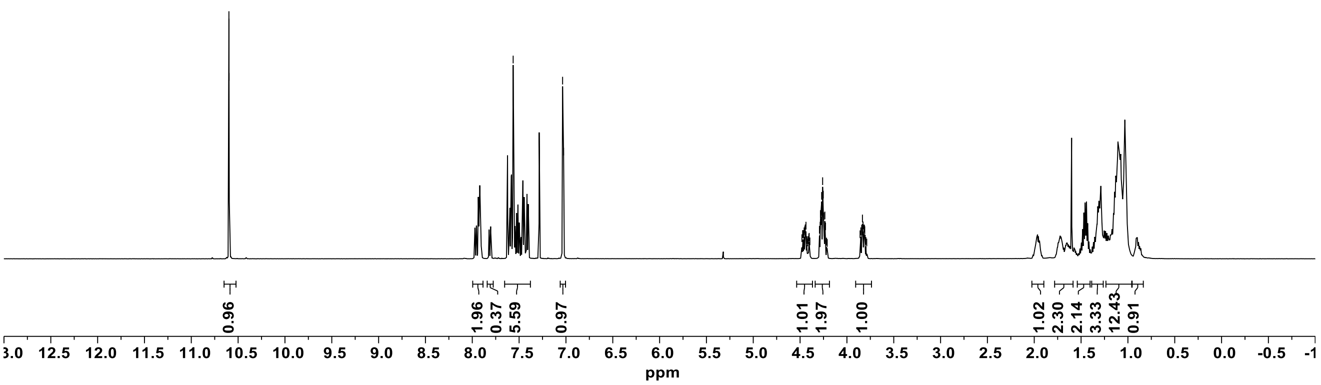

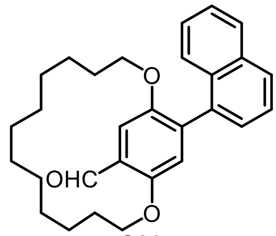

S26

$^{13}C\{^{1}H\}$, $CDCl_3$, 126 MHz

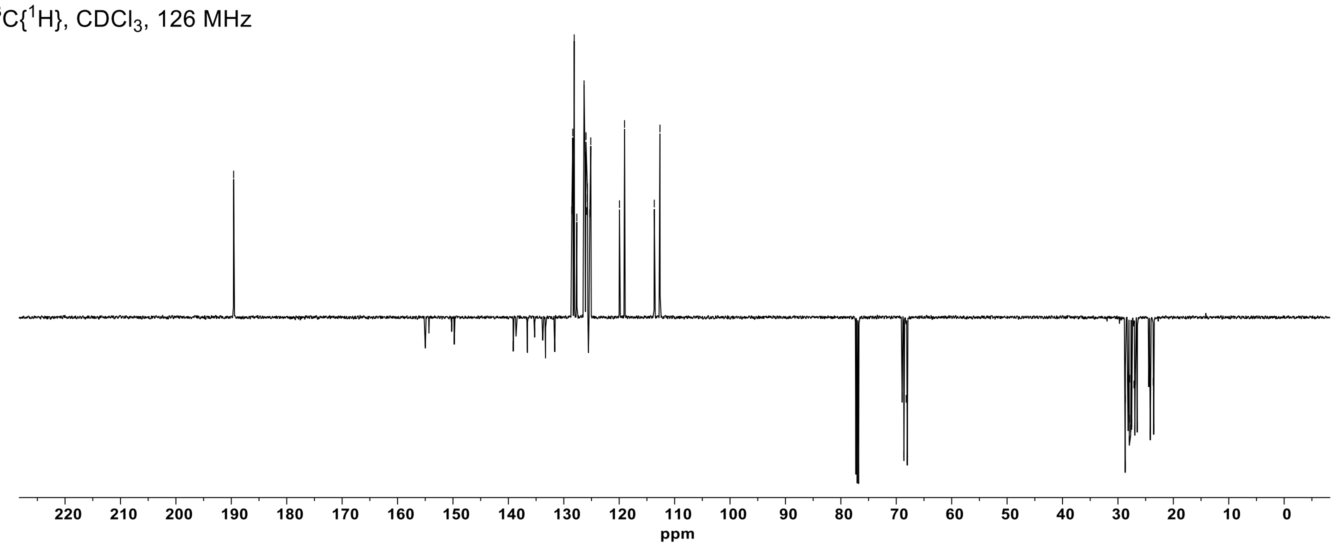

1⁵-(naphthalen-2-yl)-2,15-dioxa-1(1,4)-benzenacyclopentadecaphane-1²-carbaldehyde S27

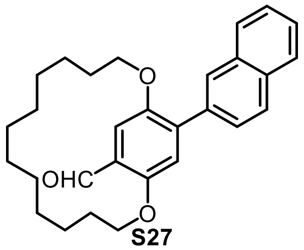

S27

^{1}H, $CDCl_3$, 500 MHz

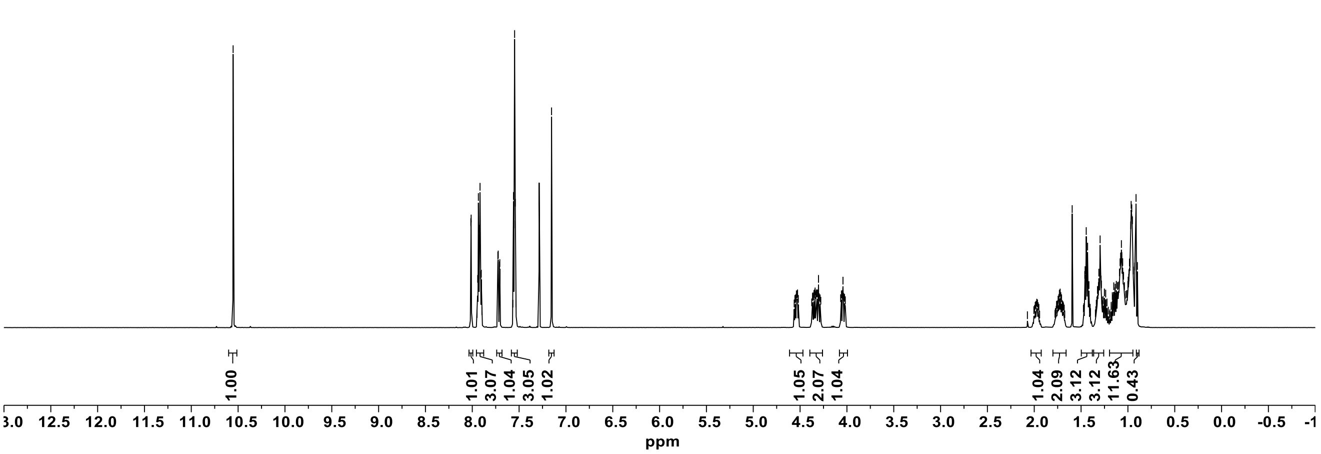

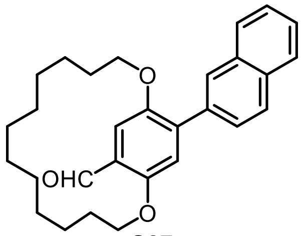

S27

$^{13}C\{^1H\}$, $CDCl_3$, 126 MHz

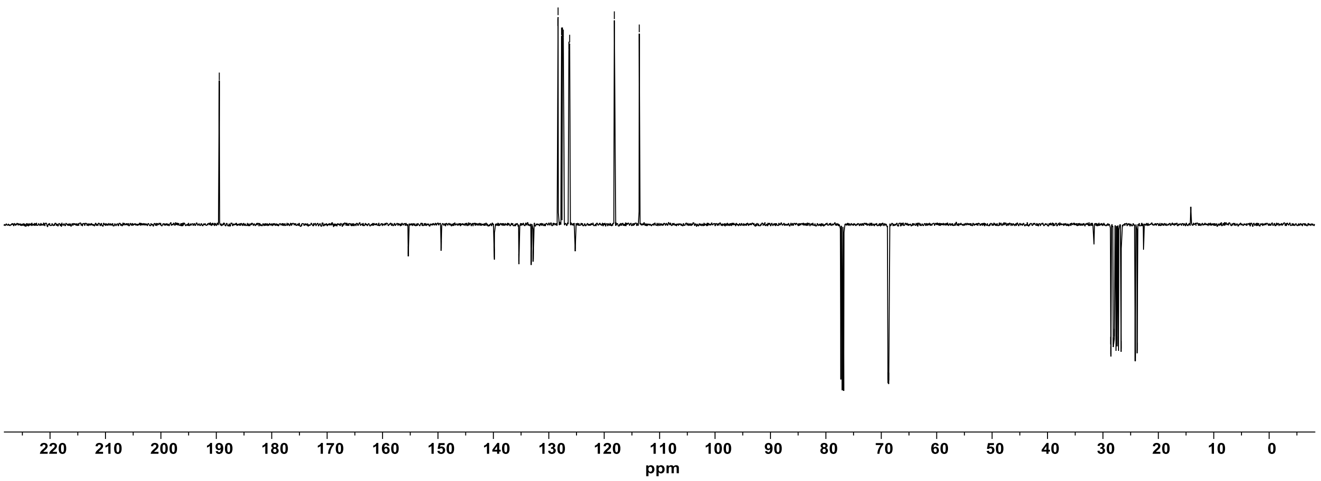

1[5]-(pyridin-3-yl)-2,15-dioxa-1(1,4)-benzenacyclopentadecaphane-1[2]-carbaldehyde S28

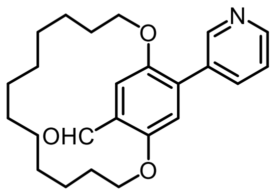

S28
^1H, $CDCl_3$, 500 MHz

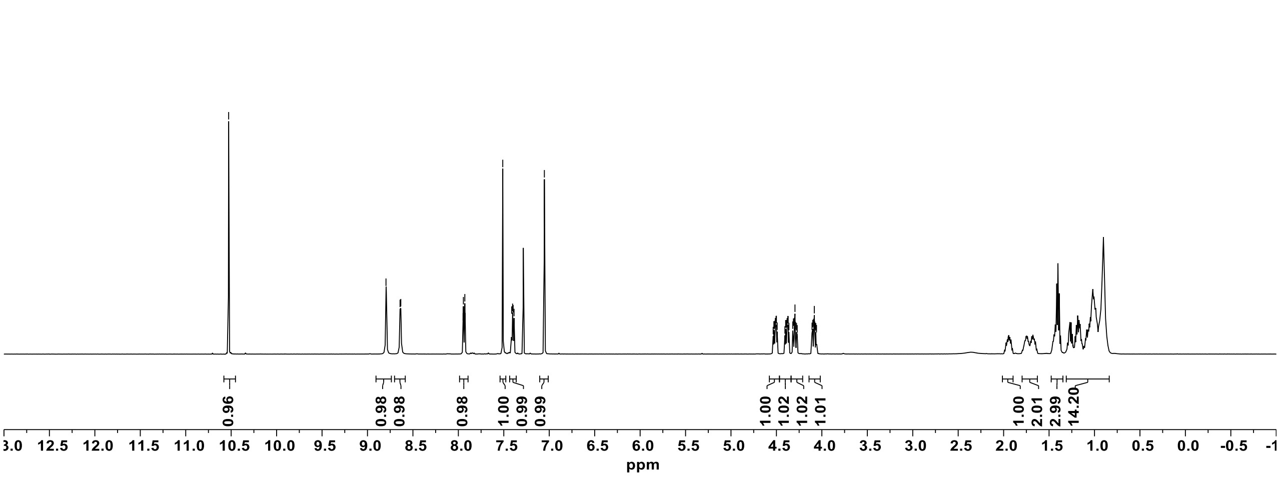

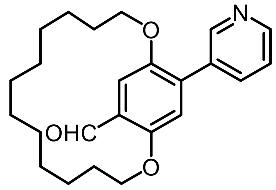

S28
$^{13}C\{^1H\}$, $CDCl_3$, 126 MHz

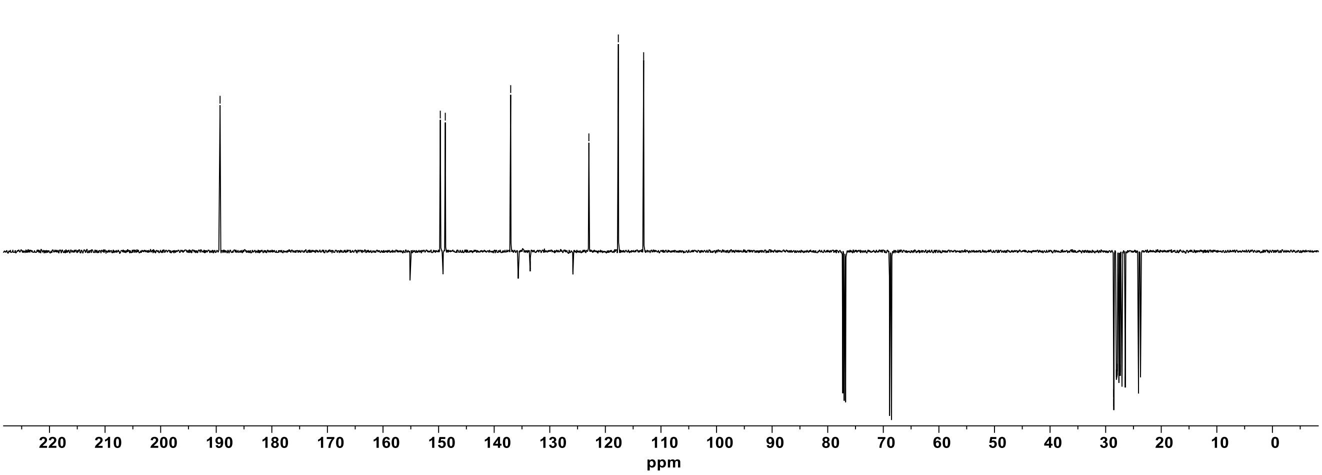

1^5-(furan-2-yl)-2,15-dioxa-1(1,4)-benzenacyclopentadecaphane-1^2-carbaldehyde S29

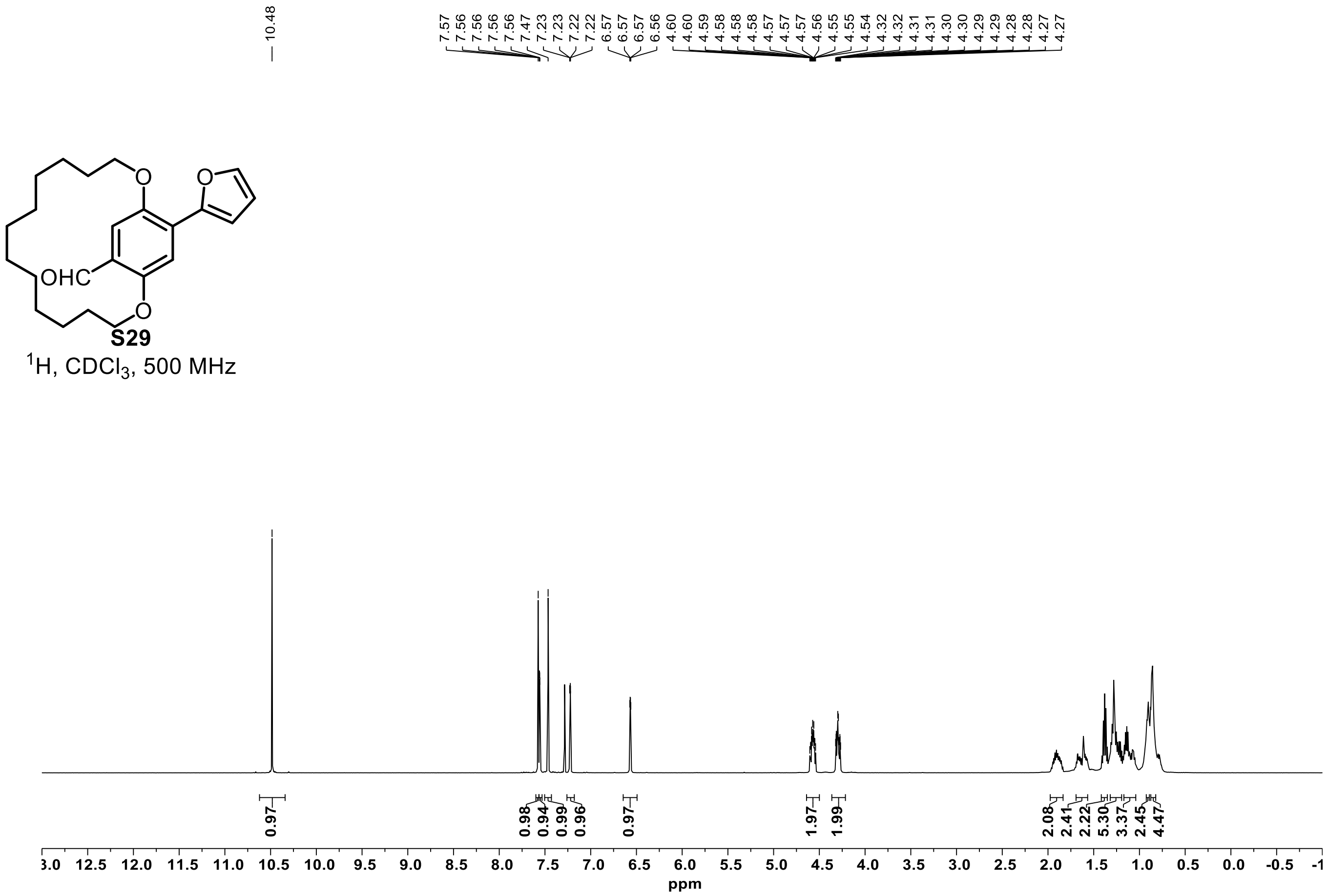

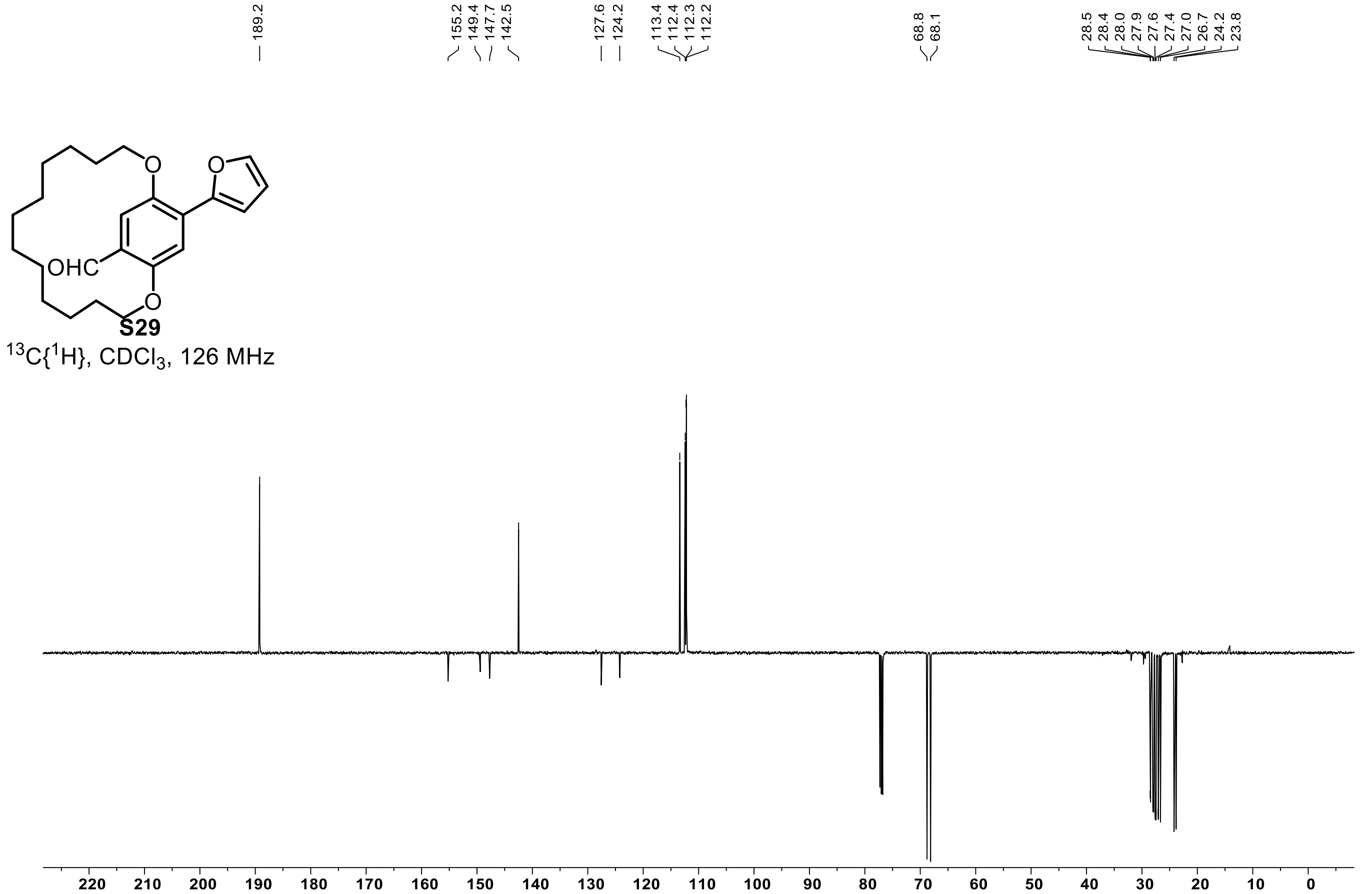

tert-butyl 3-(1⁵-formyl-2,15-dioxa-1(1,4)-benzenacyclopentadecaphane-1²-yl)-5-methoxy-1H-indole-1-carboxylate S31

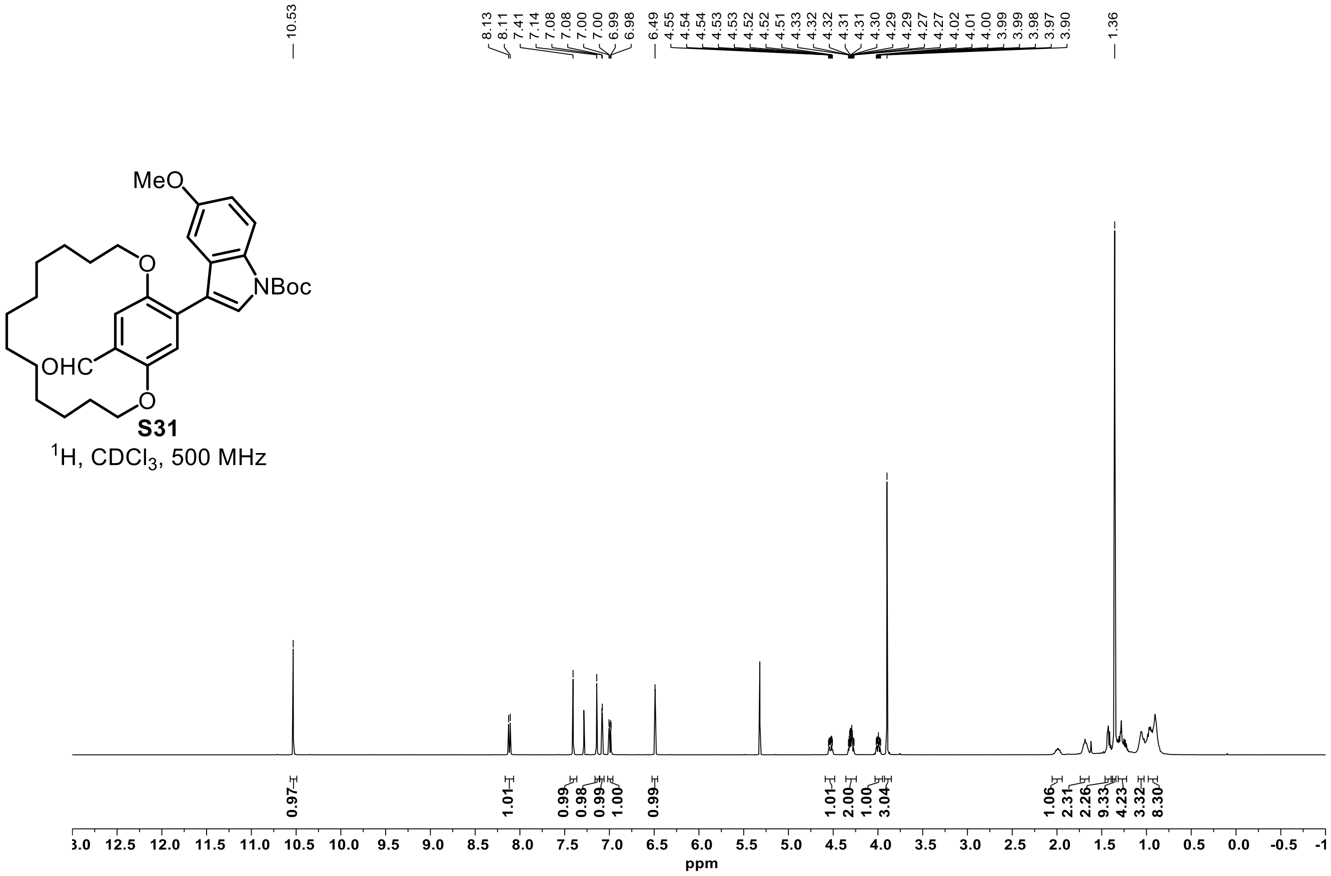

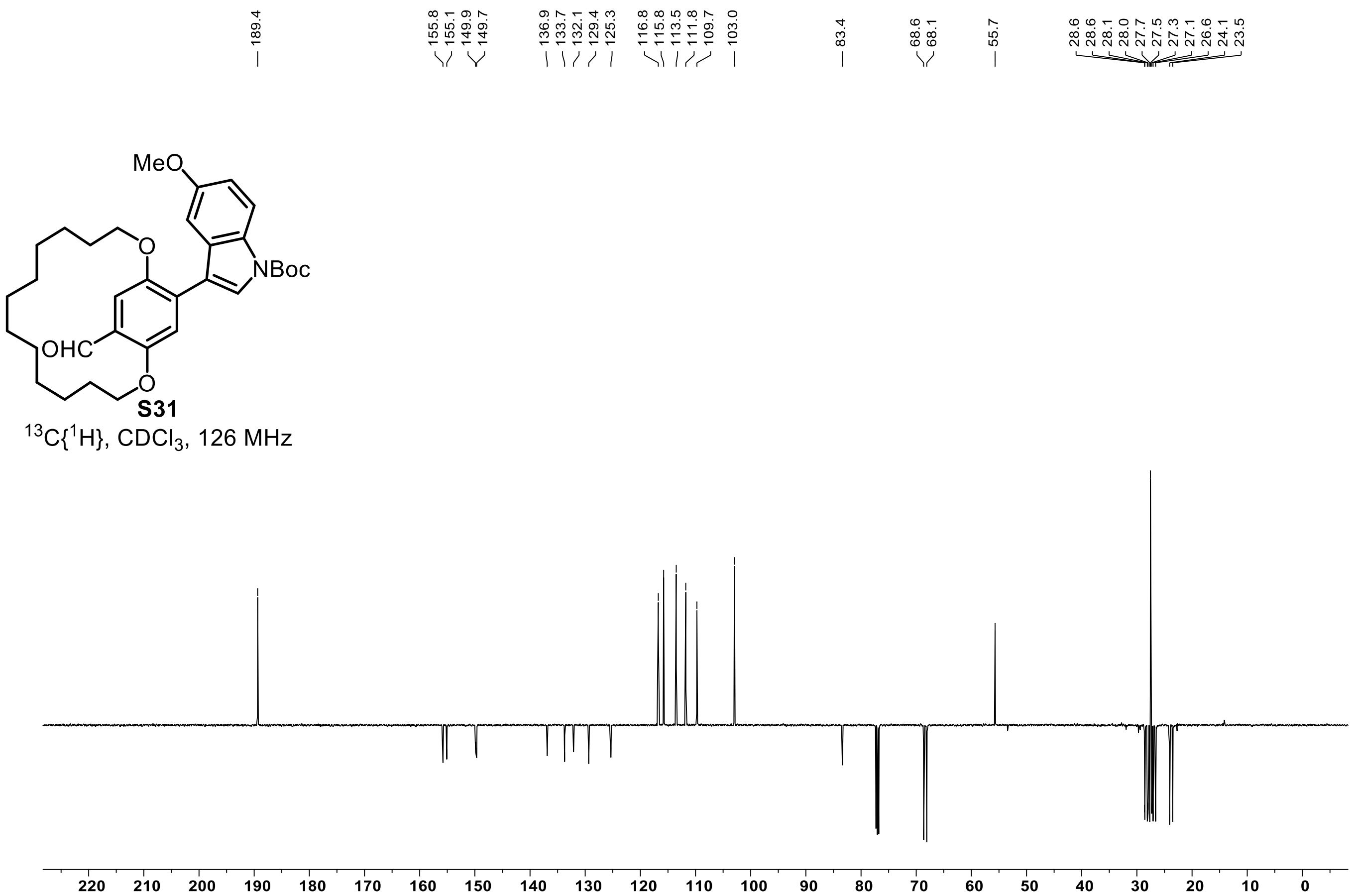

4,1⁵-dioxo-15-phenyl-2,17-dioxa-5,14-diaza-1(1,4)-benzenacycloheptadecaphane-1²-carbaldehyde S35

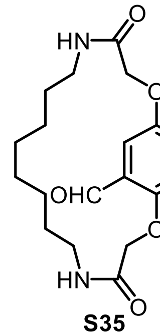

S35

^{1}H, CDCl$_3$, 500 MHz

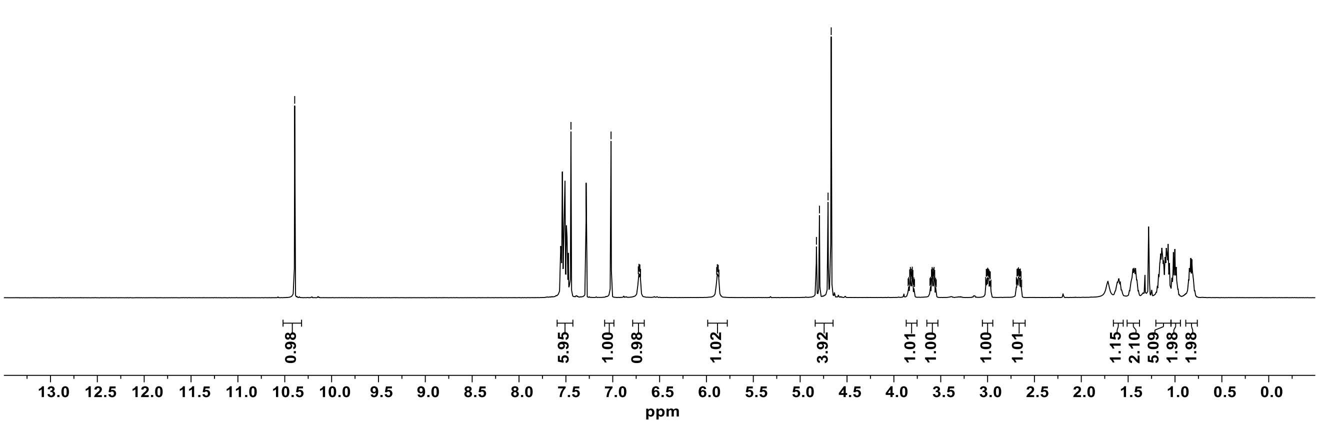

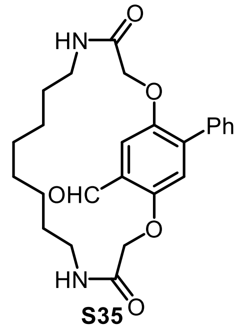

S35

^{13}C{^{1}H}, CDCl$_3$, 126 MHz

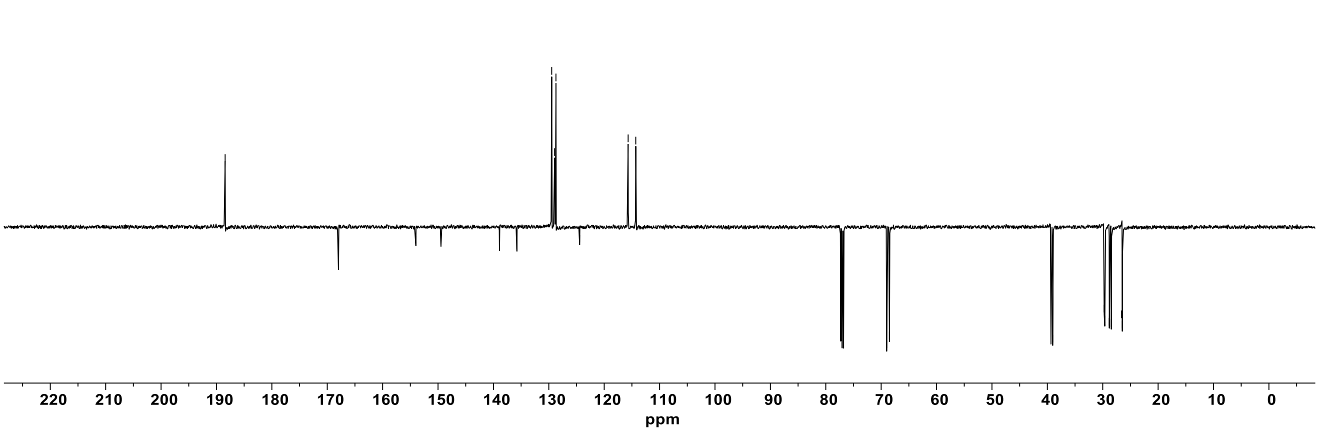

1⁵-phenyl-2,19-dioxa-1(1,4)-benzenacyclotridecaphane-1²-carbaldehyde S37

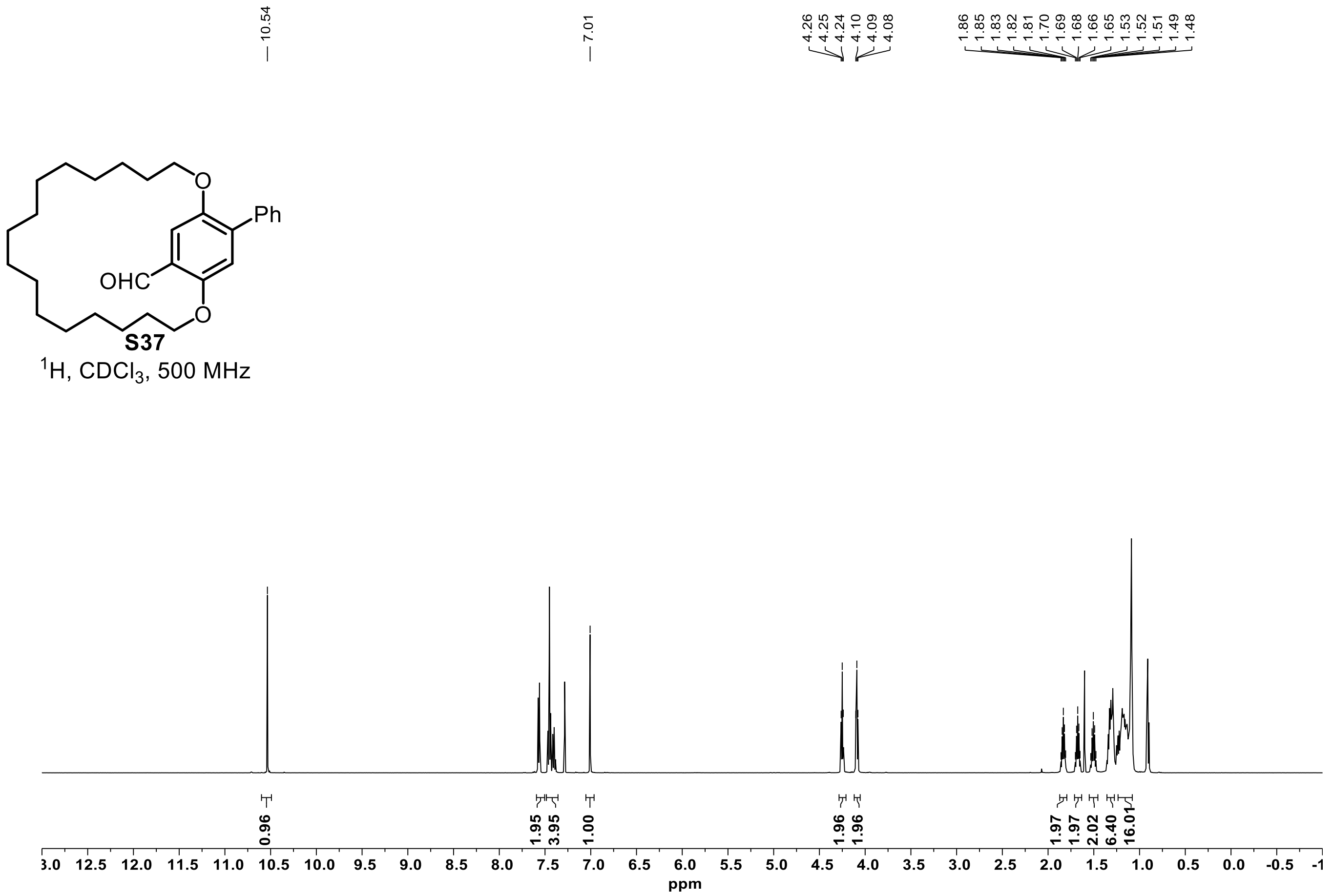

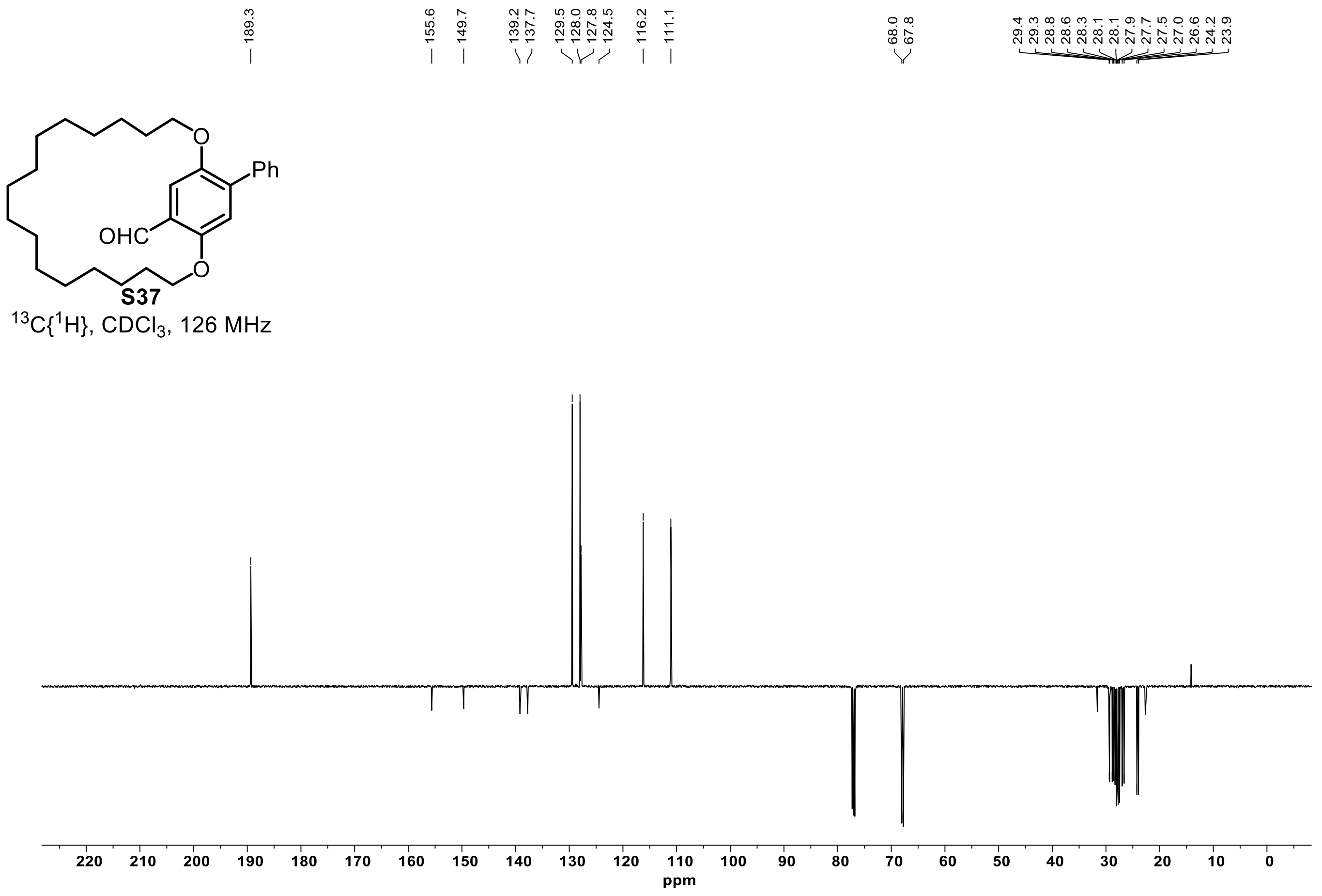

1⁵-(pyridin-3-yl)-2,13-dioxa-1(1,4)-benzenacyclotridecaphan-1²-ol S38

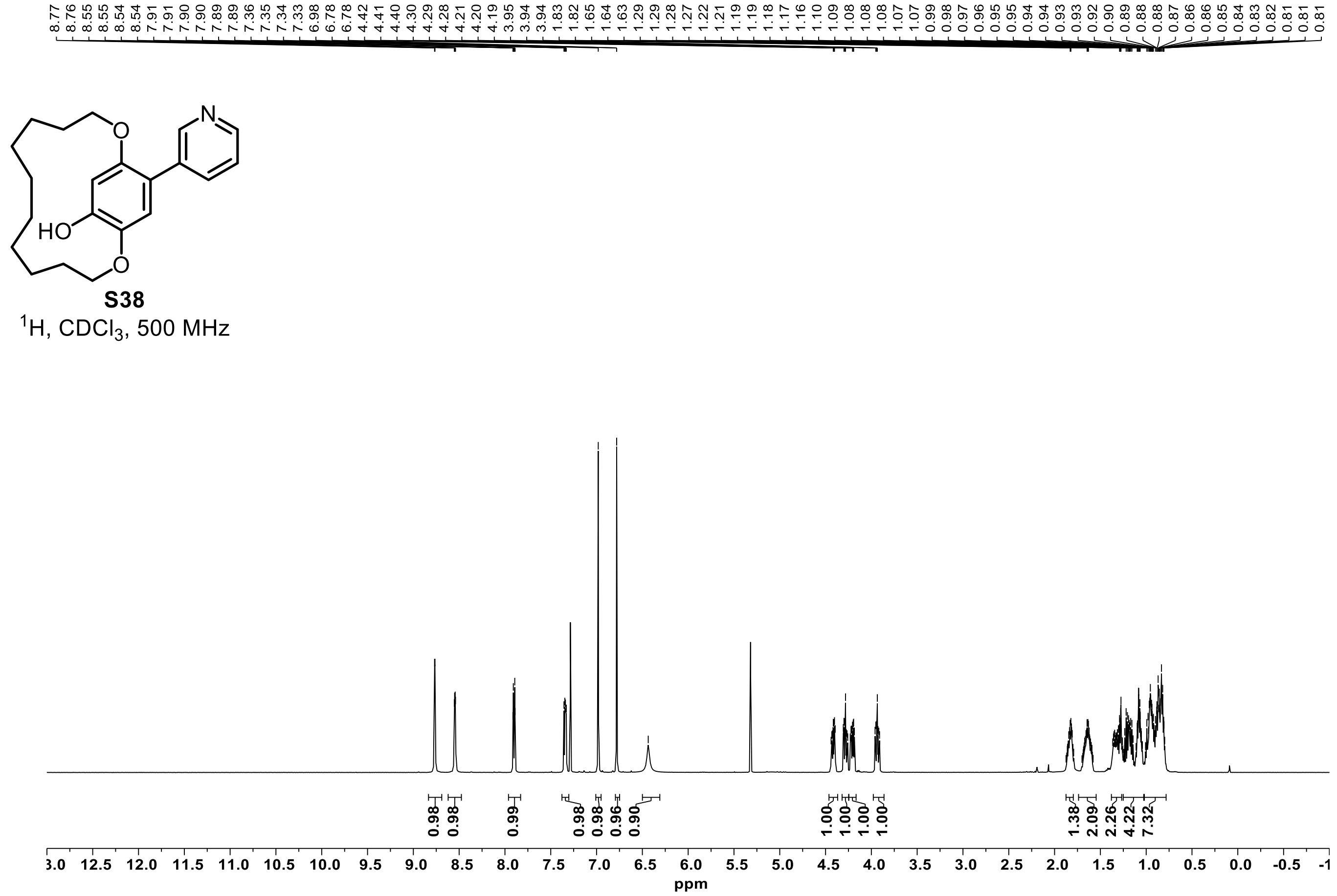

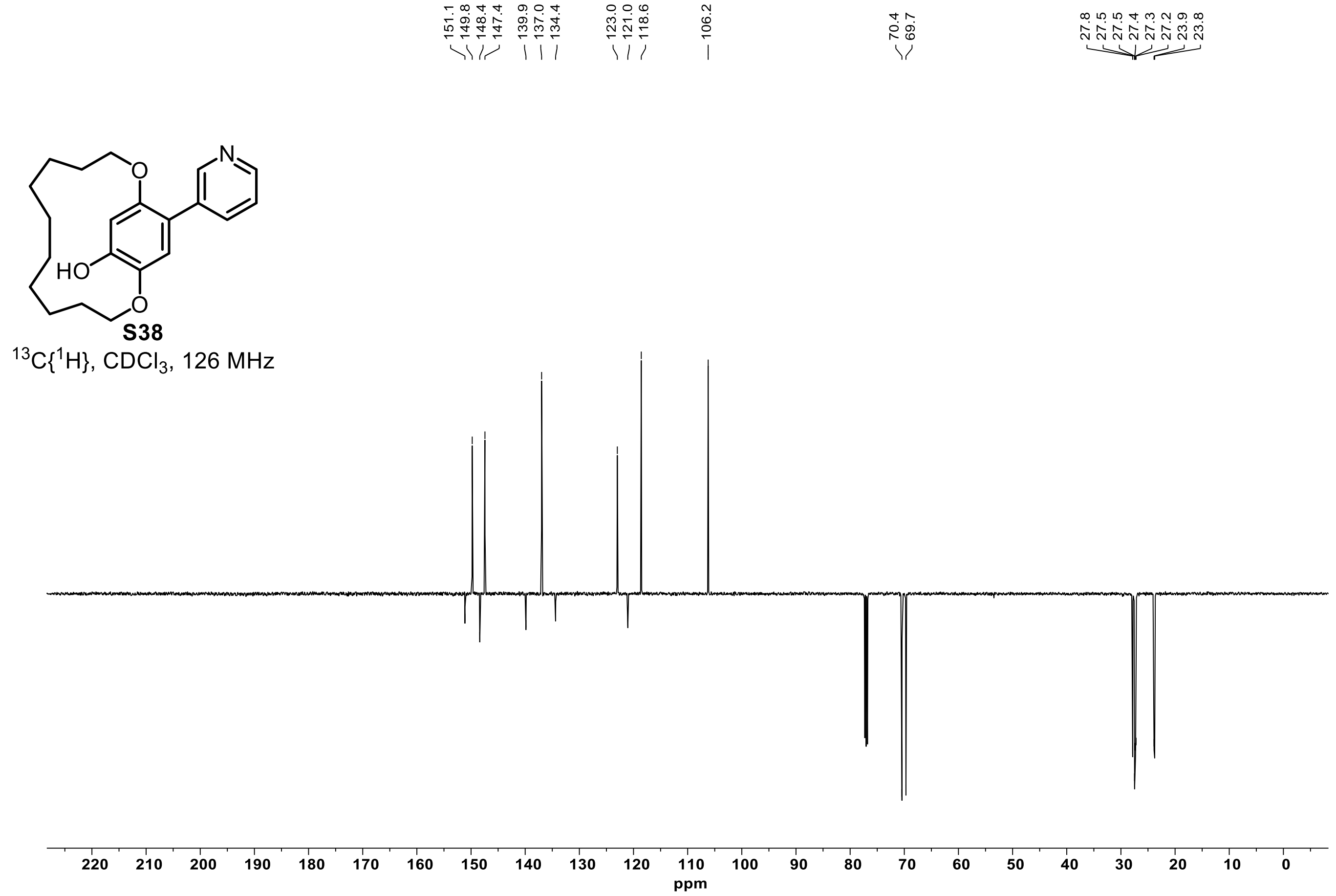

1[5]-vinyl-2,15-dioxa-1(1,4)-benzenacyclopentadecaphan-1[2]-ol S39

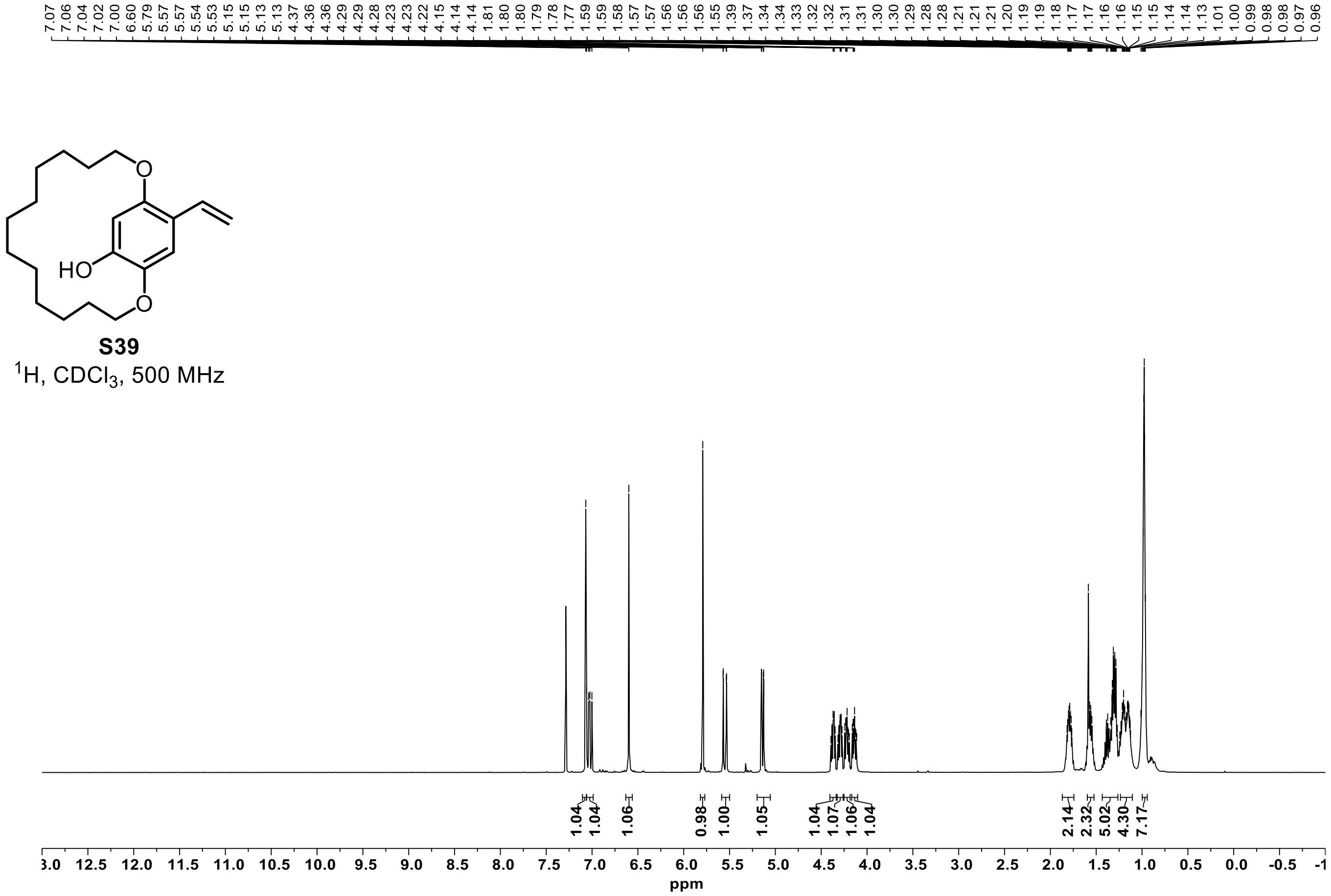

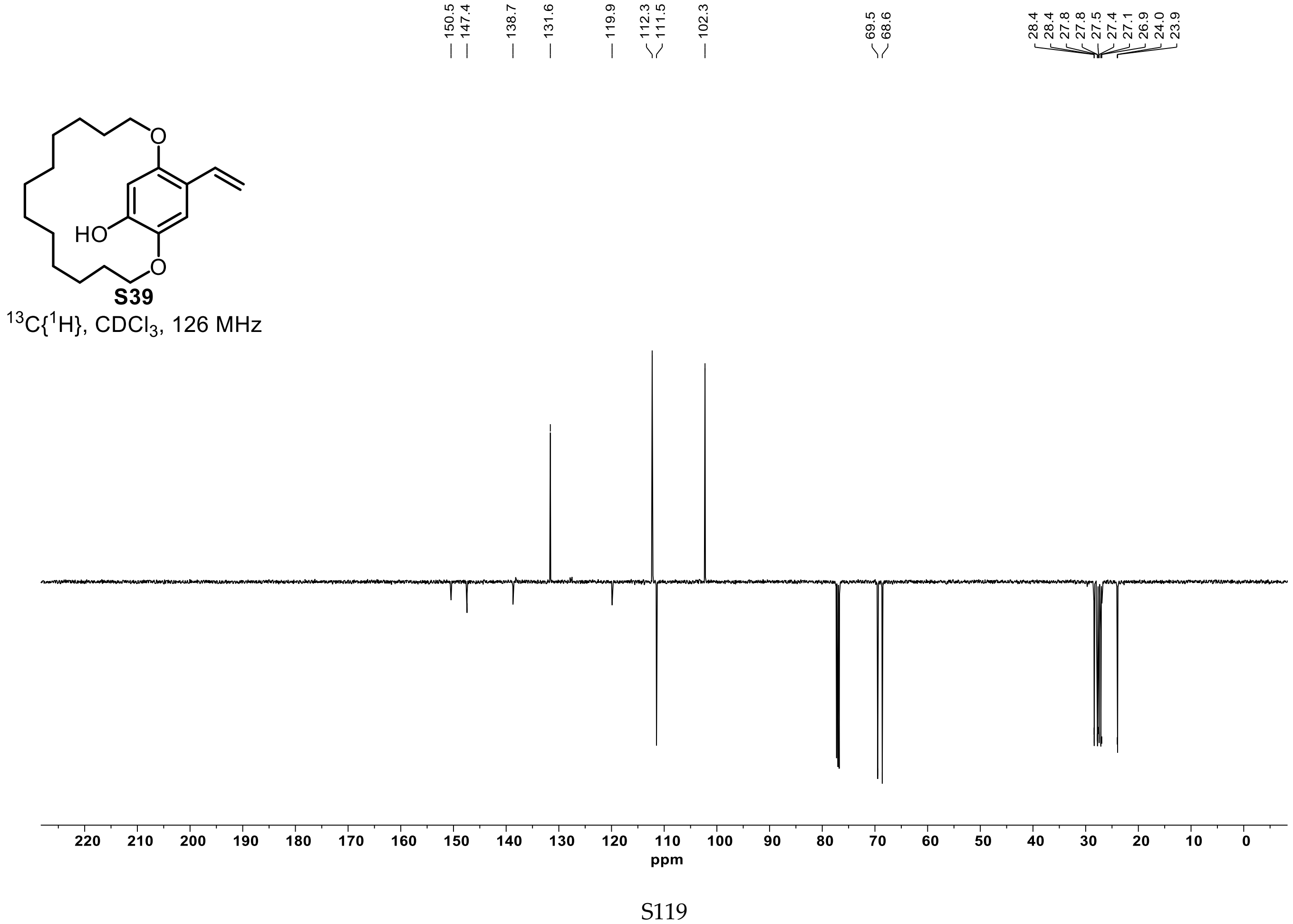

methyl 2,15-dioxa-1(1,4)-naphthalenacyclopentadecaphane-1²-carboxylate S41

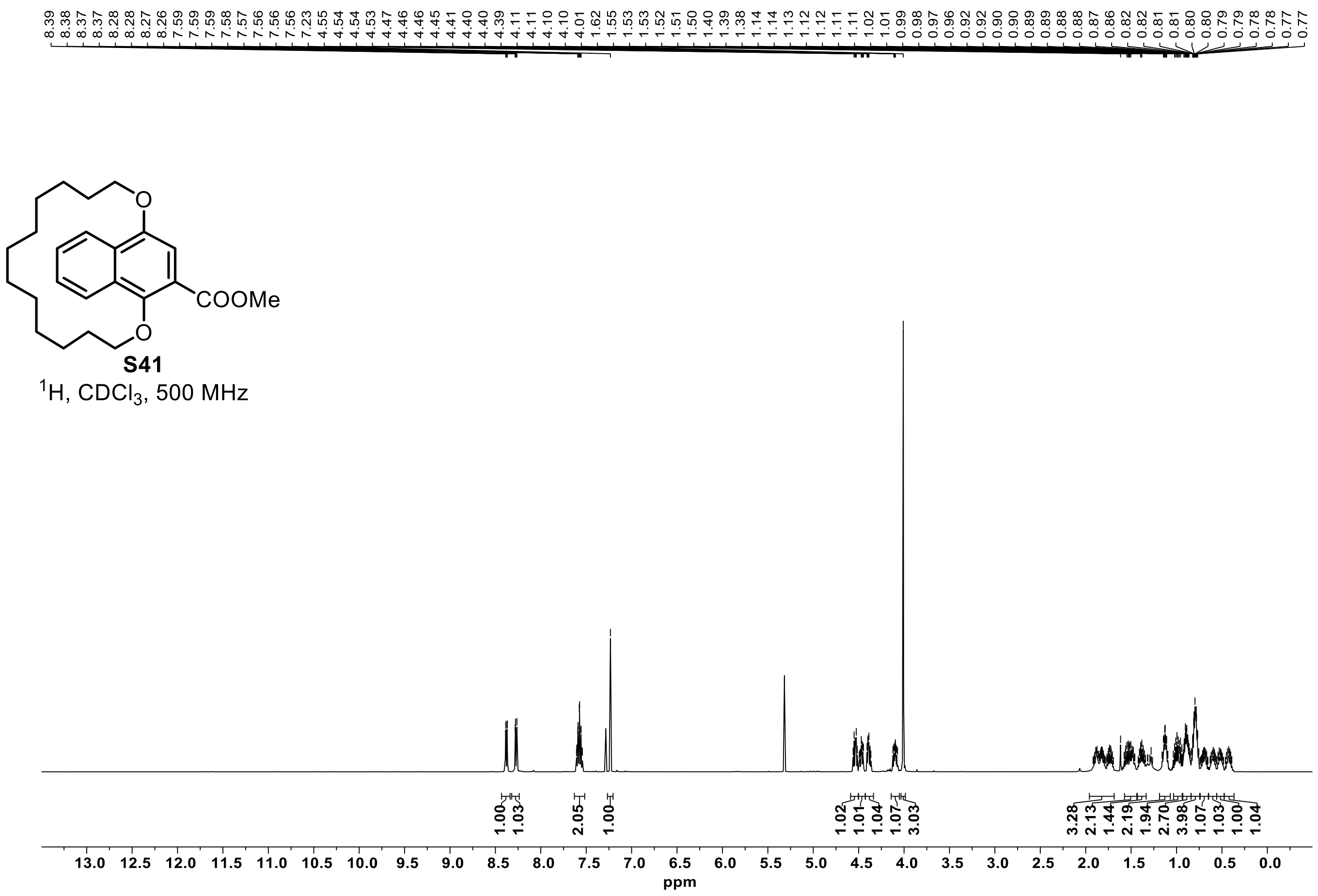

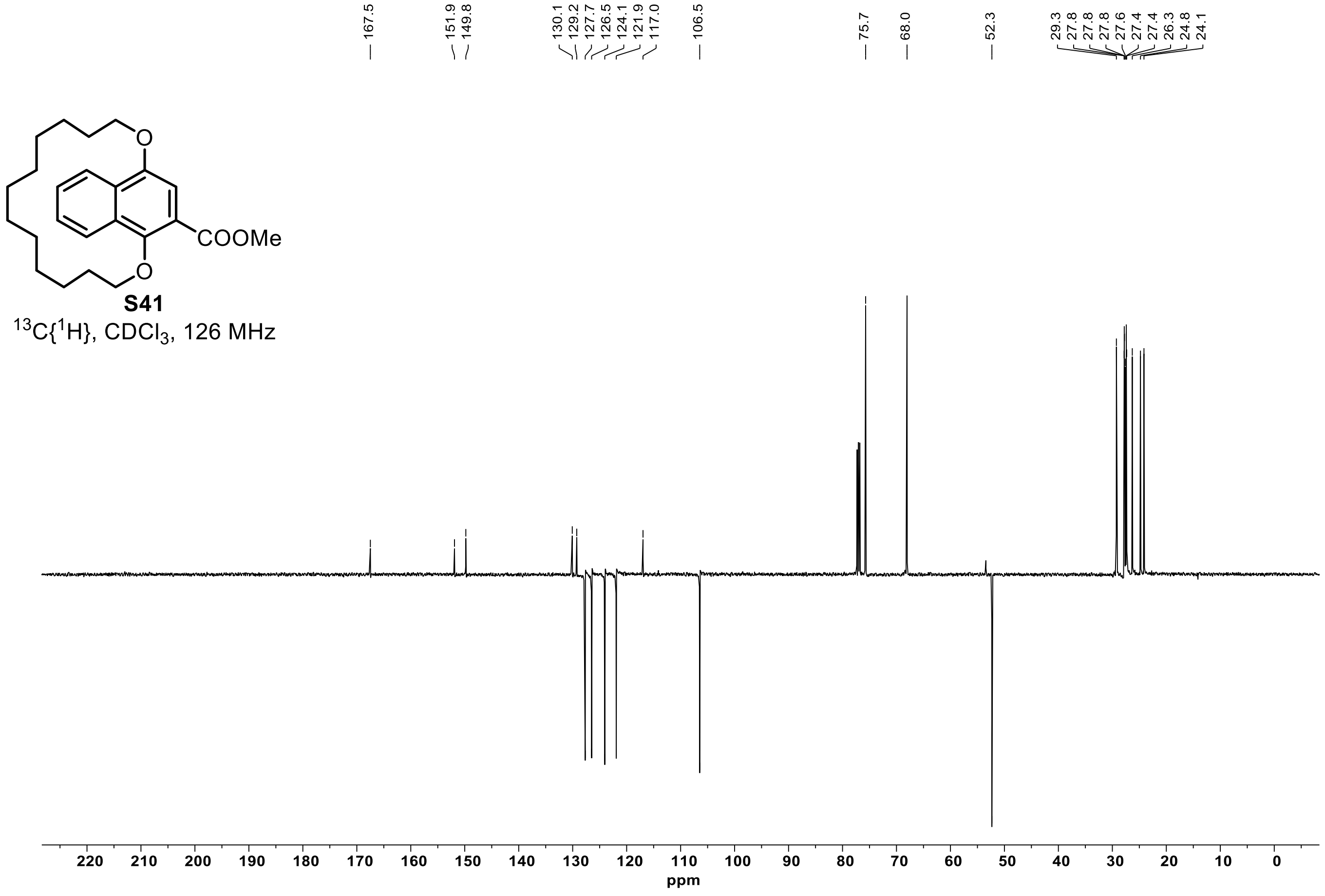

1⁵-bromo-2,13-dioxa-1(1,4)-benzenacyclotridecaphan-1²-ol 1

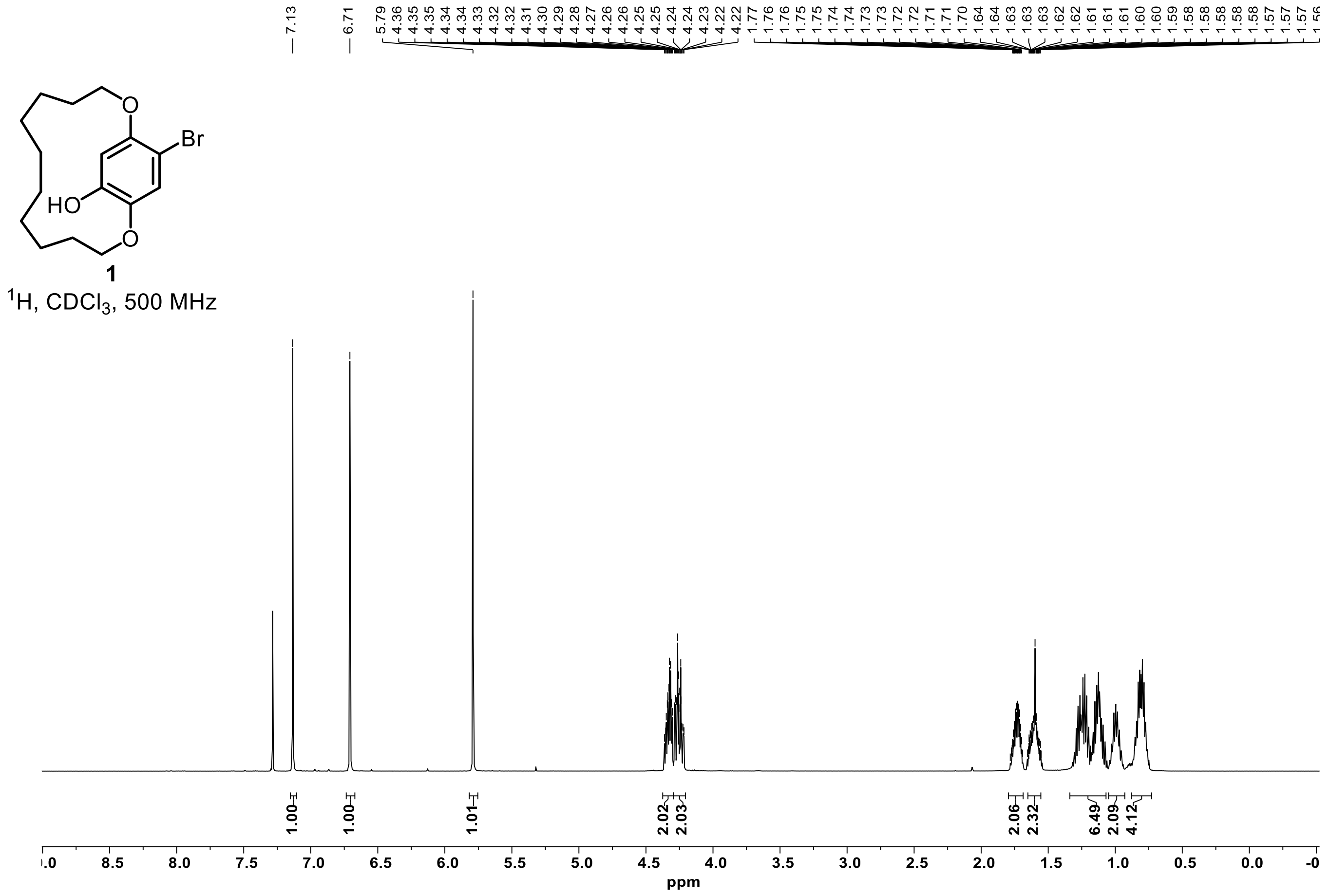

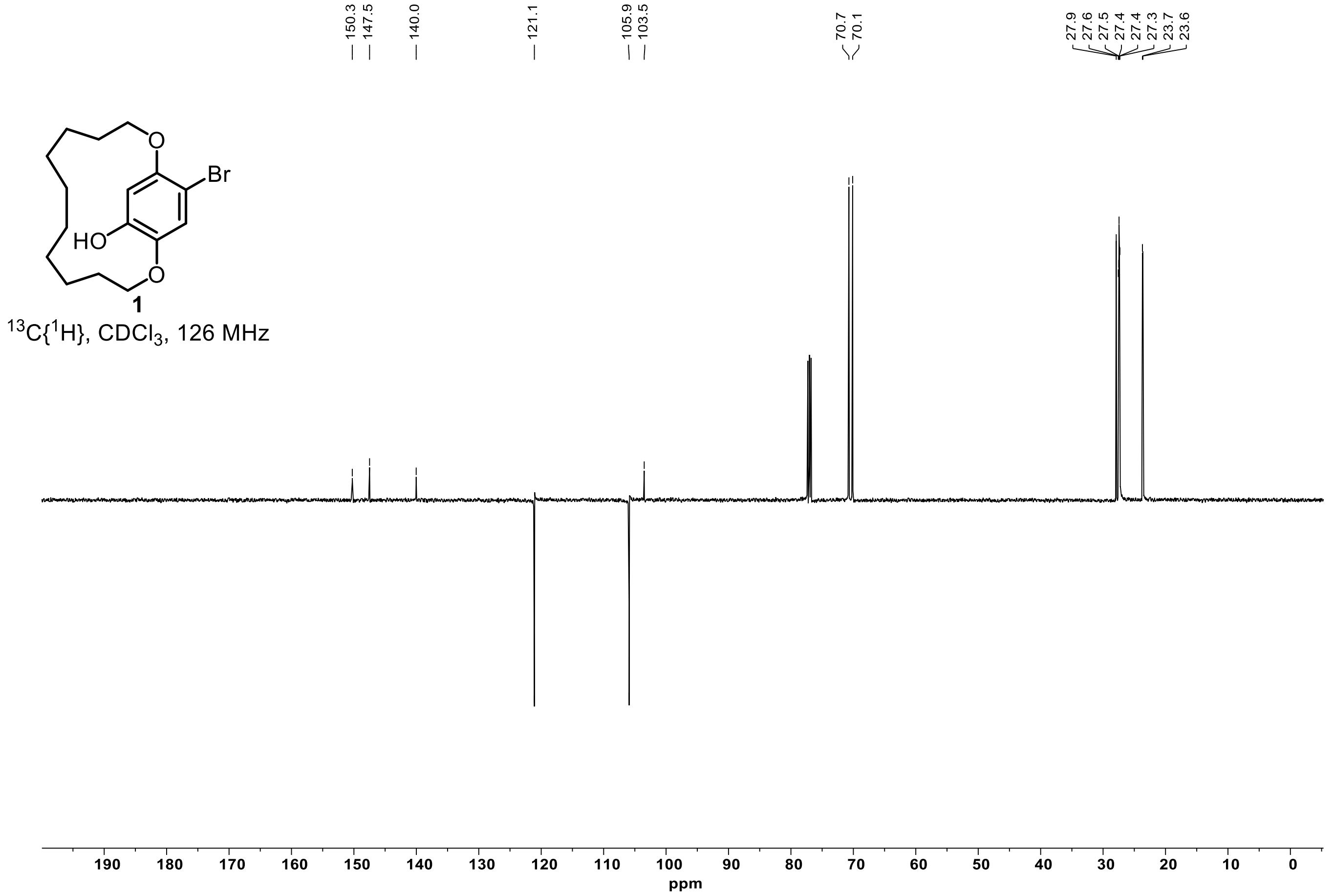

1^5-phenyl-2,13-dioxa-1(1,4)-benzenacyclotridecaphan-1^2-ol S44

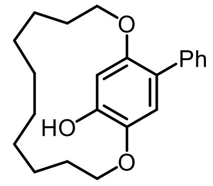

S44

^{1}H, CDCl$_3$, 500 MHz

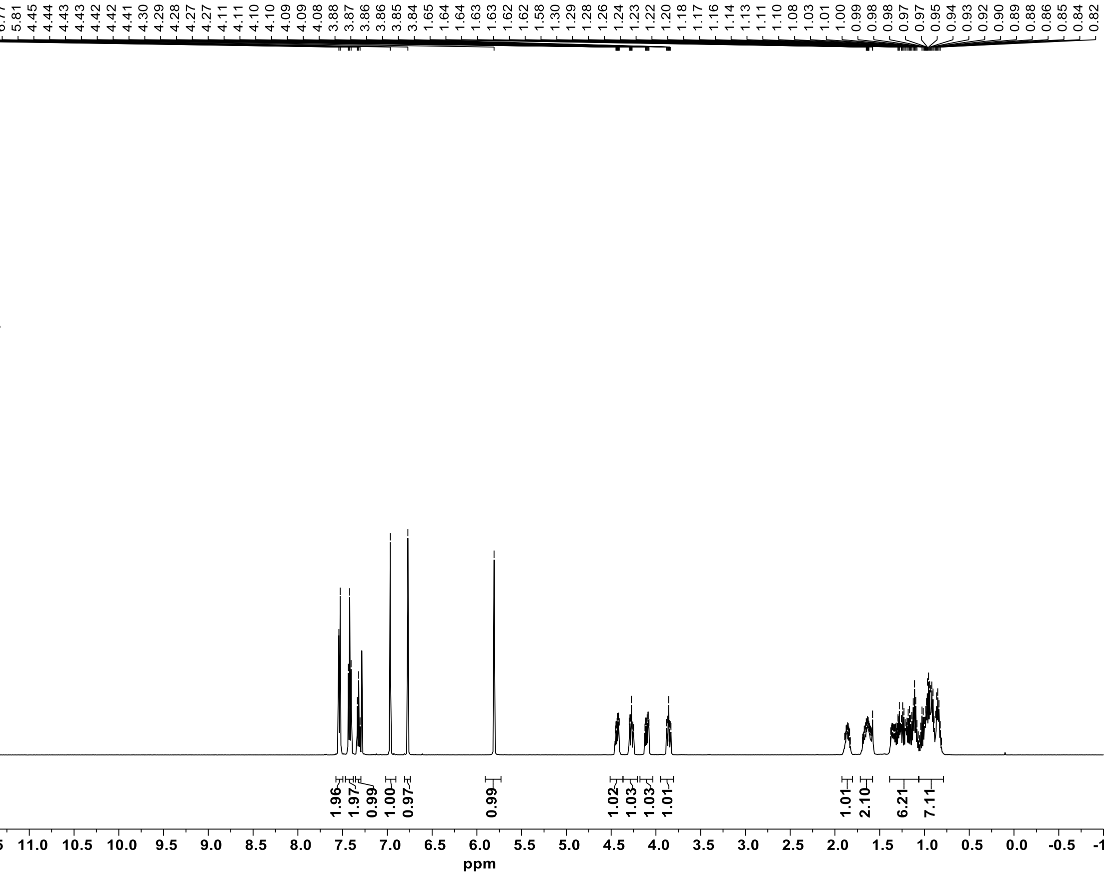

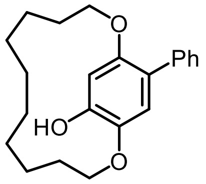

S44

^{13}C{^{1}H}, CDCl$_3$, 126 MHz

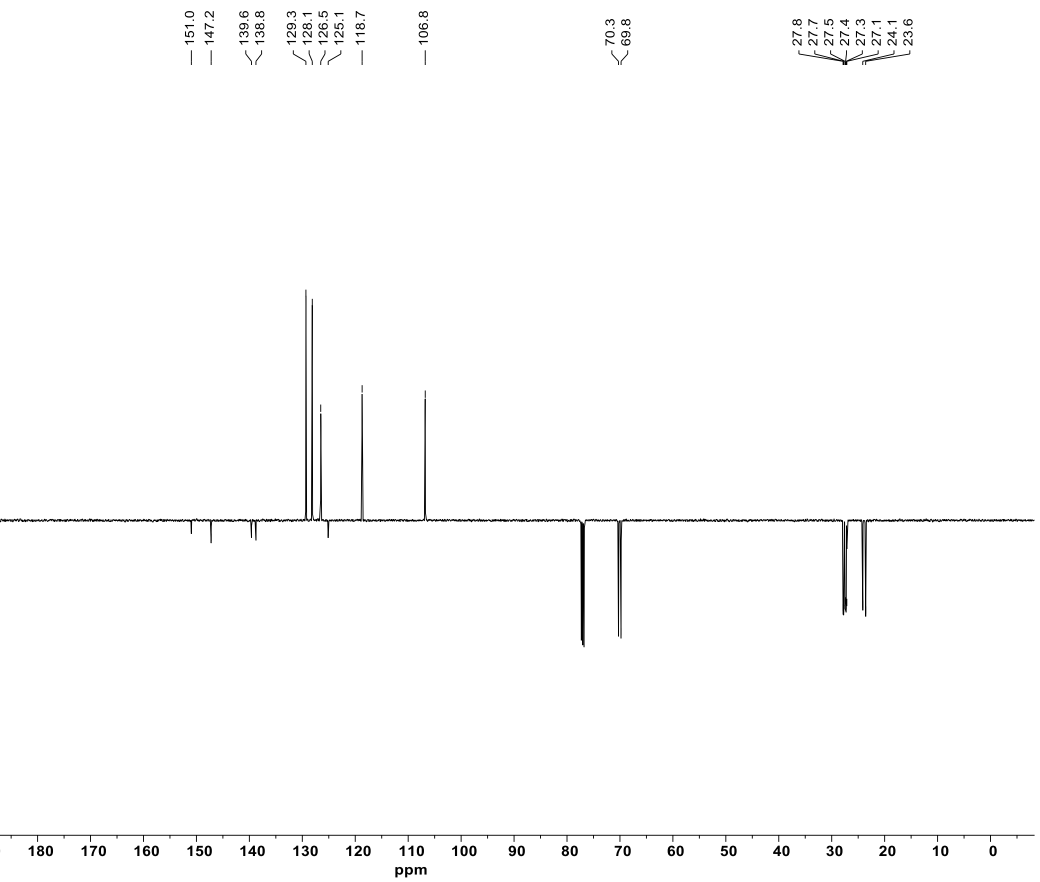

1⁵-bromo-2,14-dioxa-1(1,4)-benzenacyclotetradecaphan-1²-ol S45

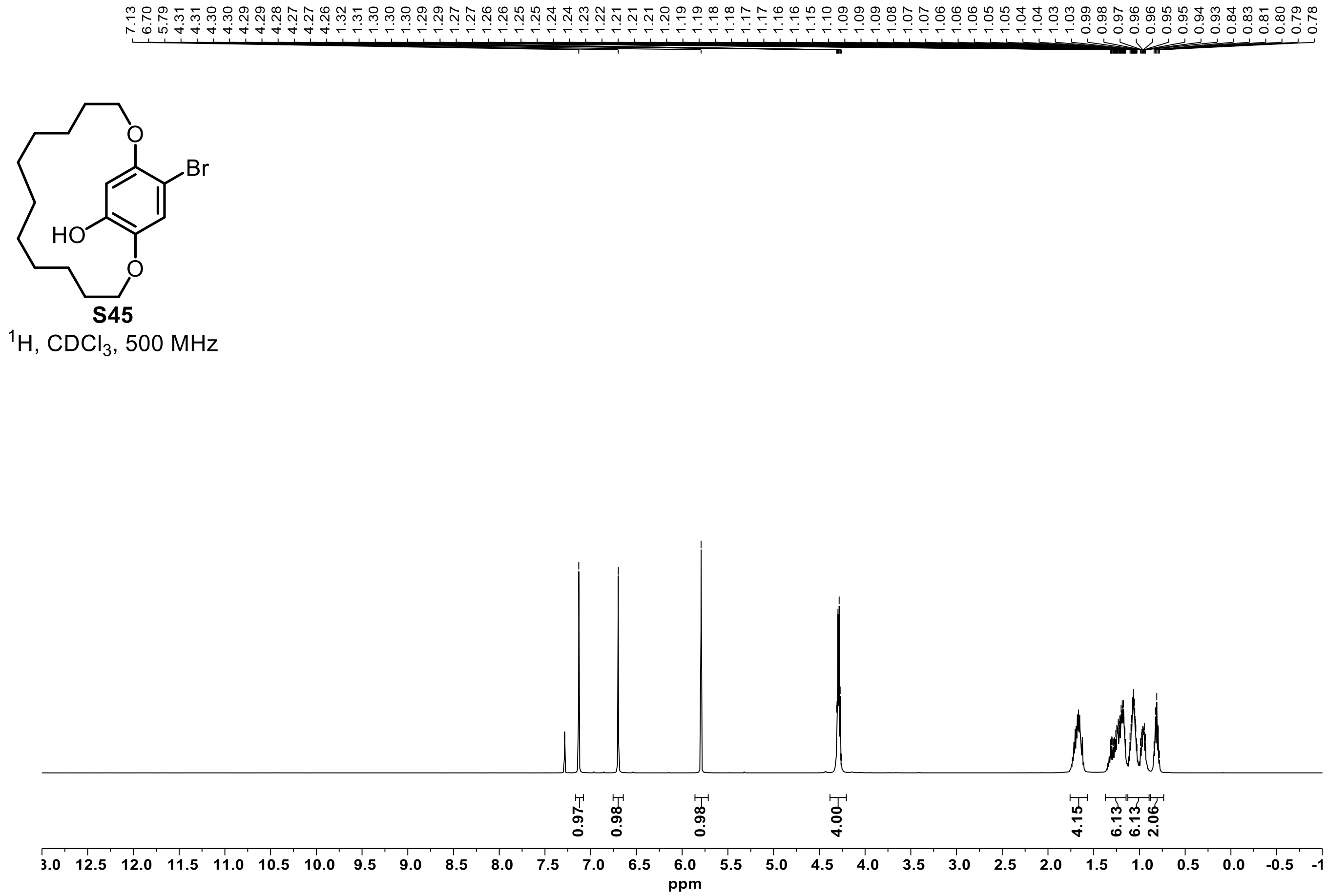

S45
^{1}H, $CDCl_3$, 500 MHz

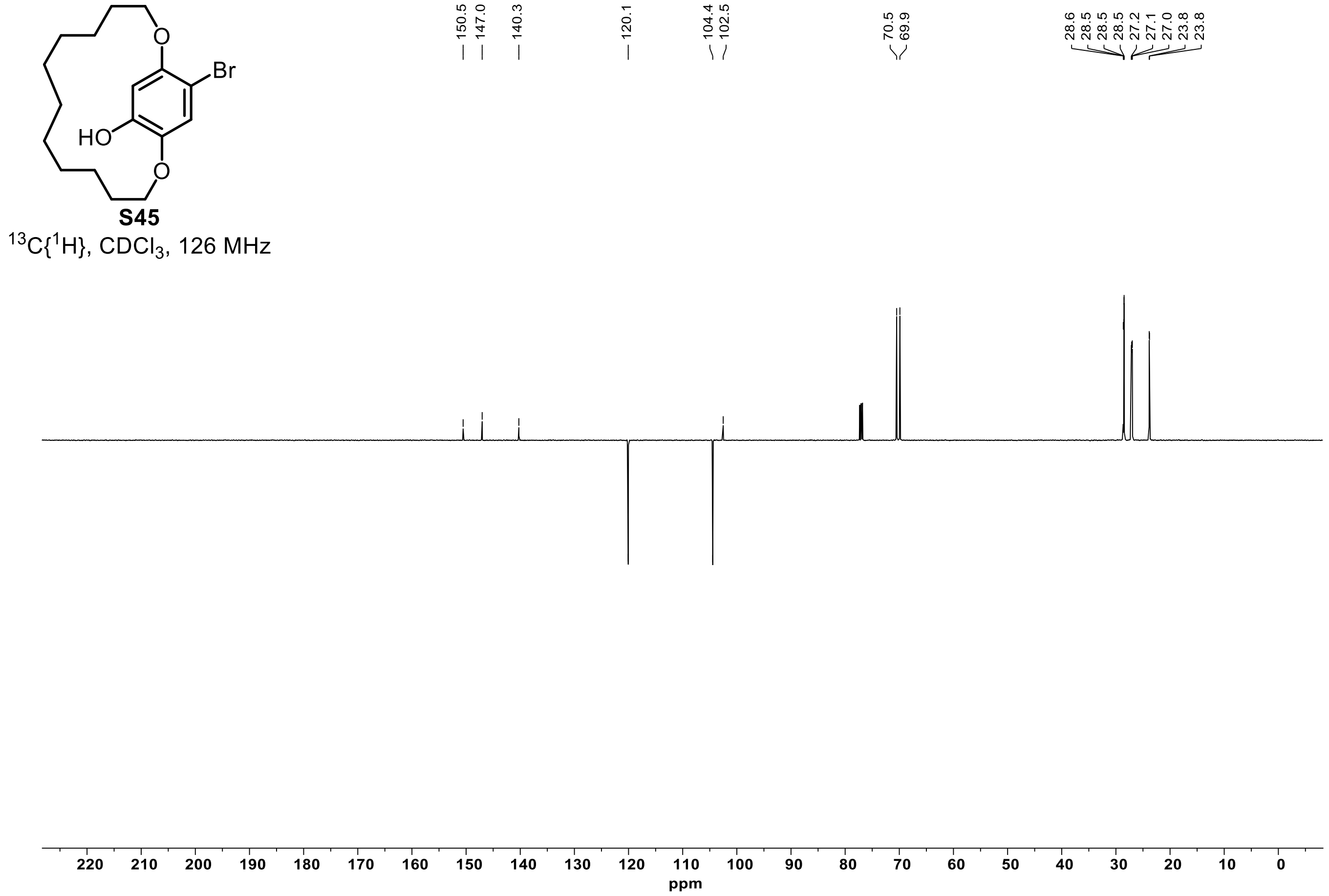

S45
$^{13}C\{^{1}H\}$, $CDCl_3$, 126 MHz

1⁵-phenyl-2,14-dioxa-1(1,4)-benzenacyclotetradecaphan-1²-ol S46

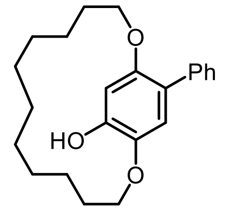

S46

^{1}H, $CDCl_3$, 500 MHz

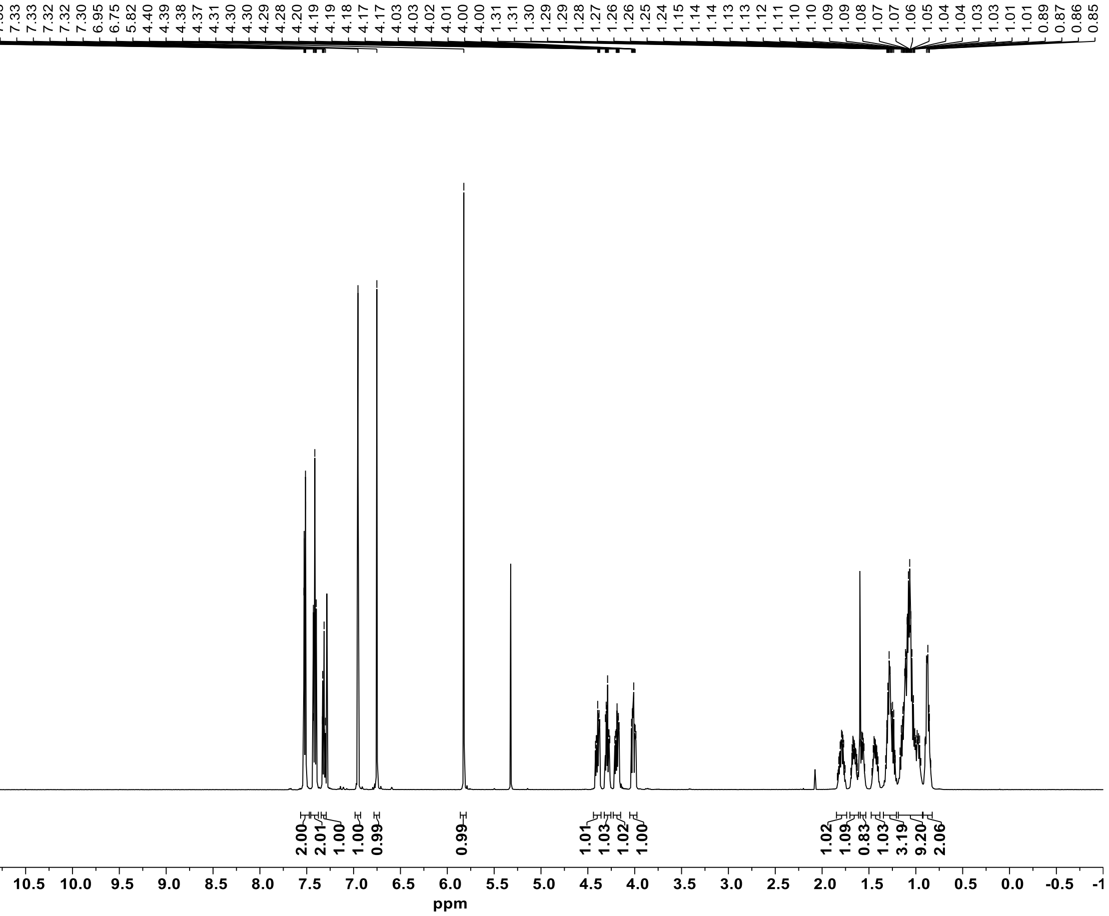

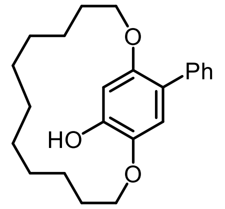

S46

$^{13}C\{^1H\}$, $CDCl_3$, 126 MHz

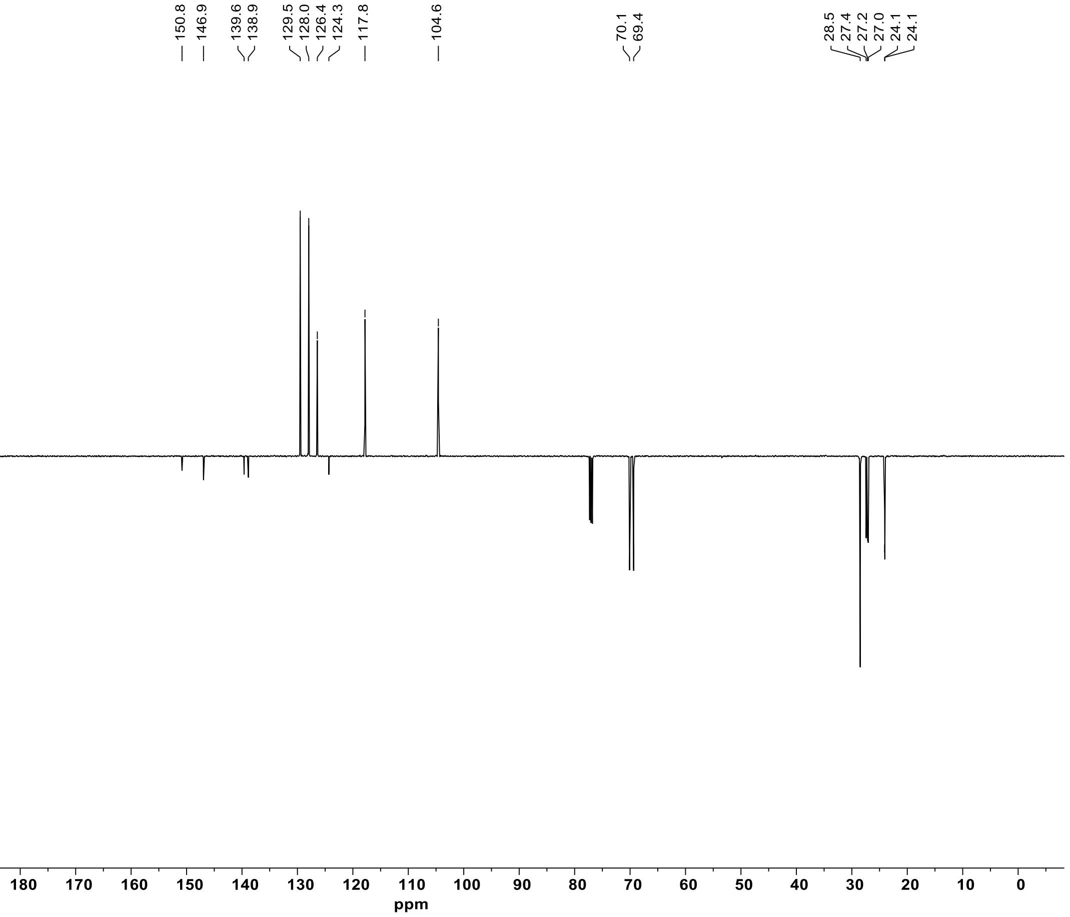

1⁵-bromo-2,15-dioxa-1(1,4)-benzenacyclopentadecaphan-1²-ol S47

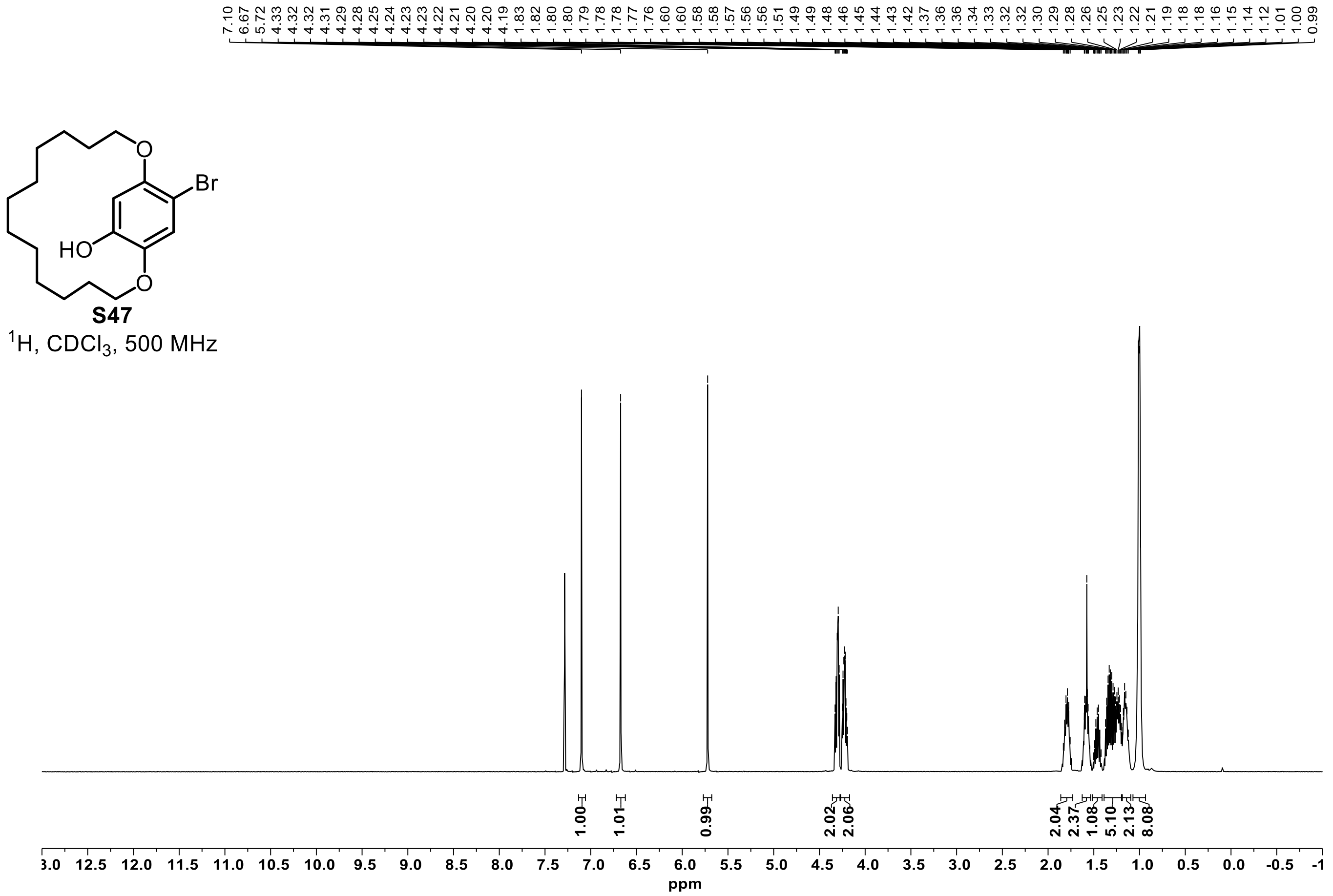

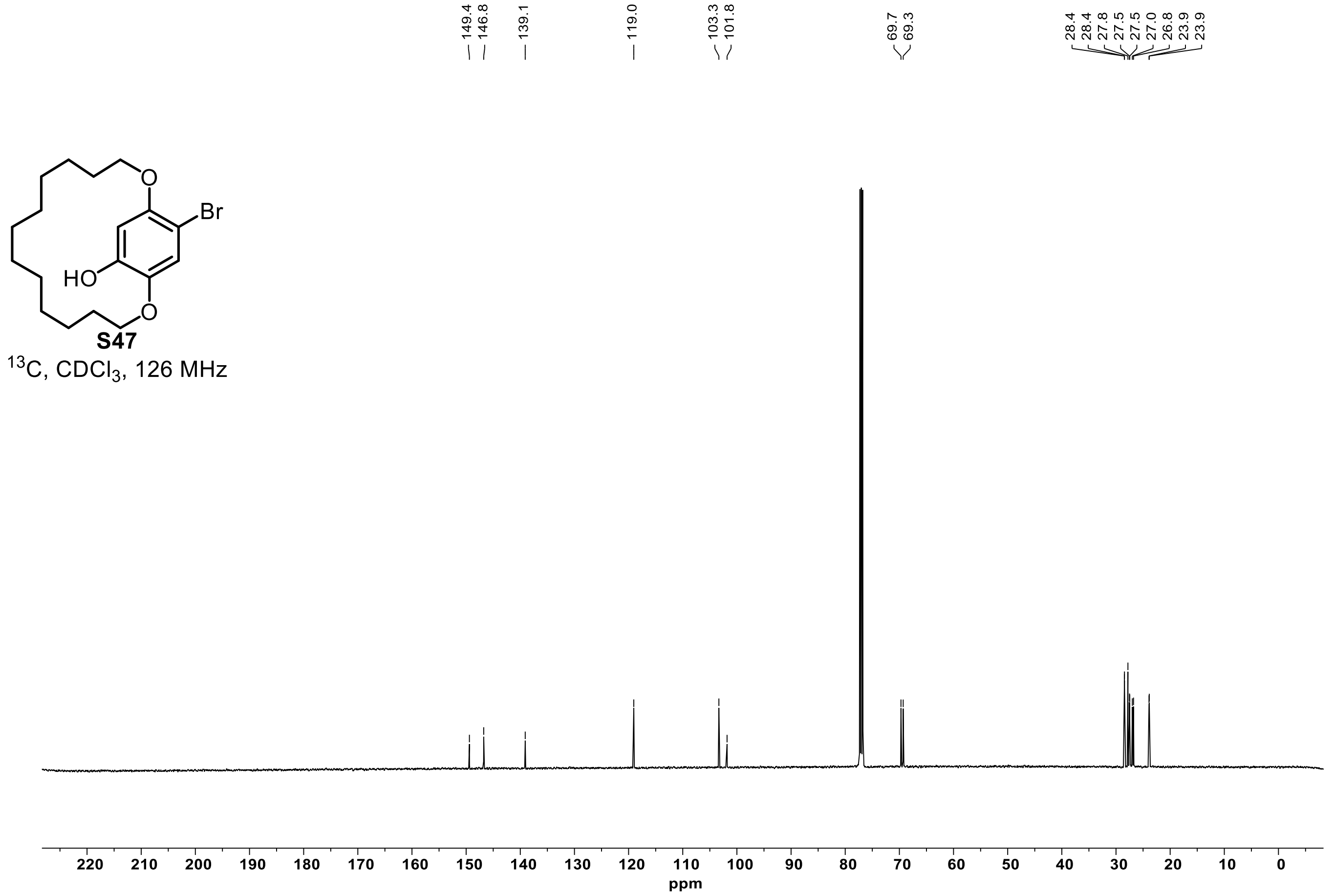

1⁵-phenyl-2,15-dioxa-1(1,4)-benzenacyclopentadecaphan-1²-ol 10

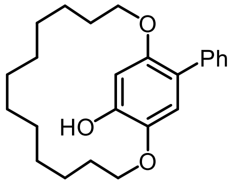

10

^{1}H, $CDCl_3$, 500 MHz

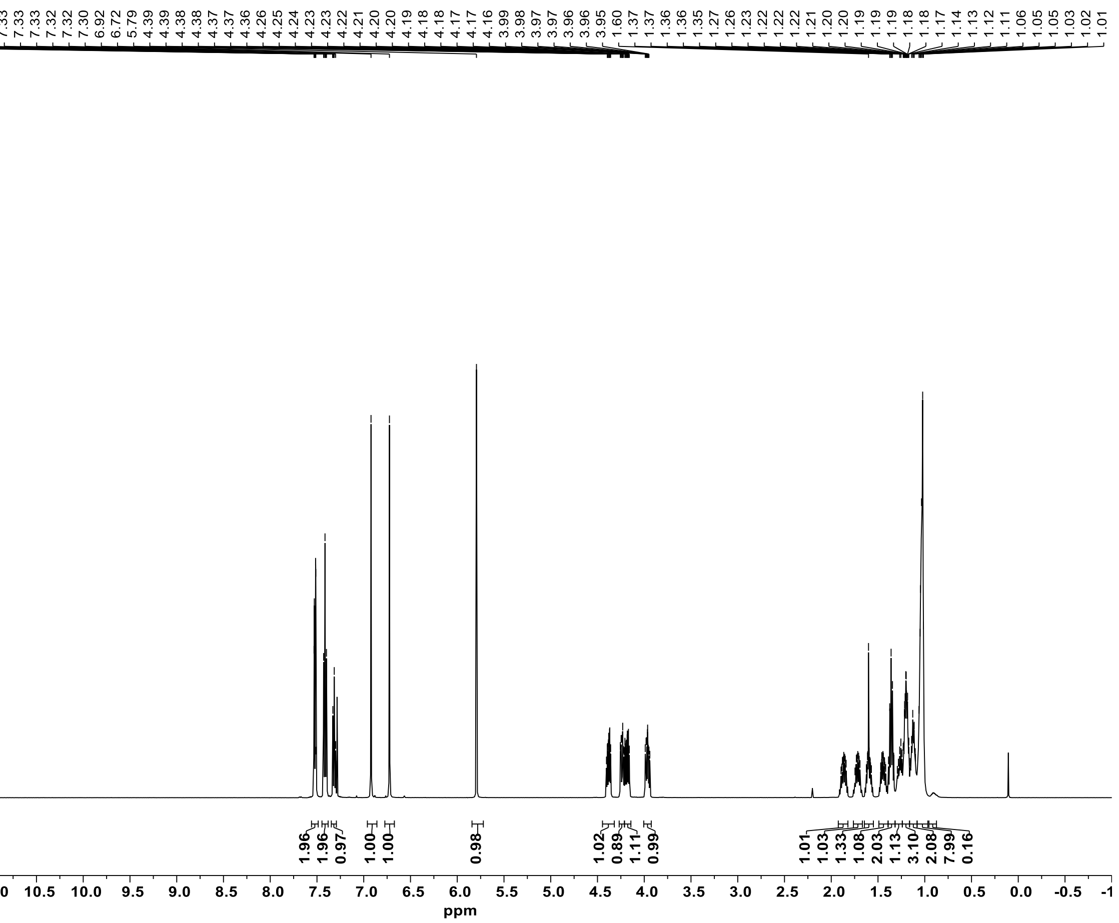

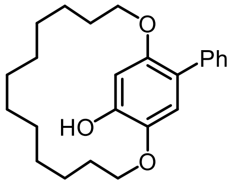

10

$^{13}C\{^{1}H\}$, $CDCl_3$, 126 MHz

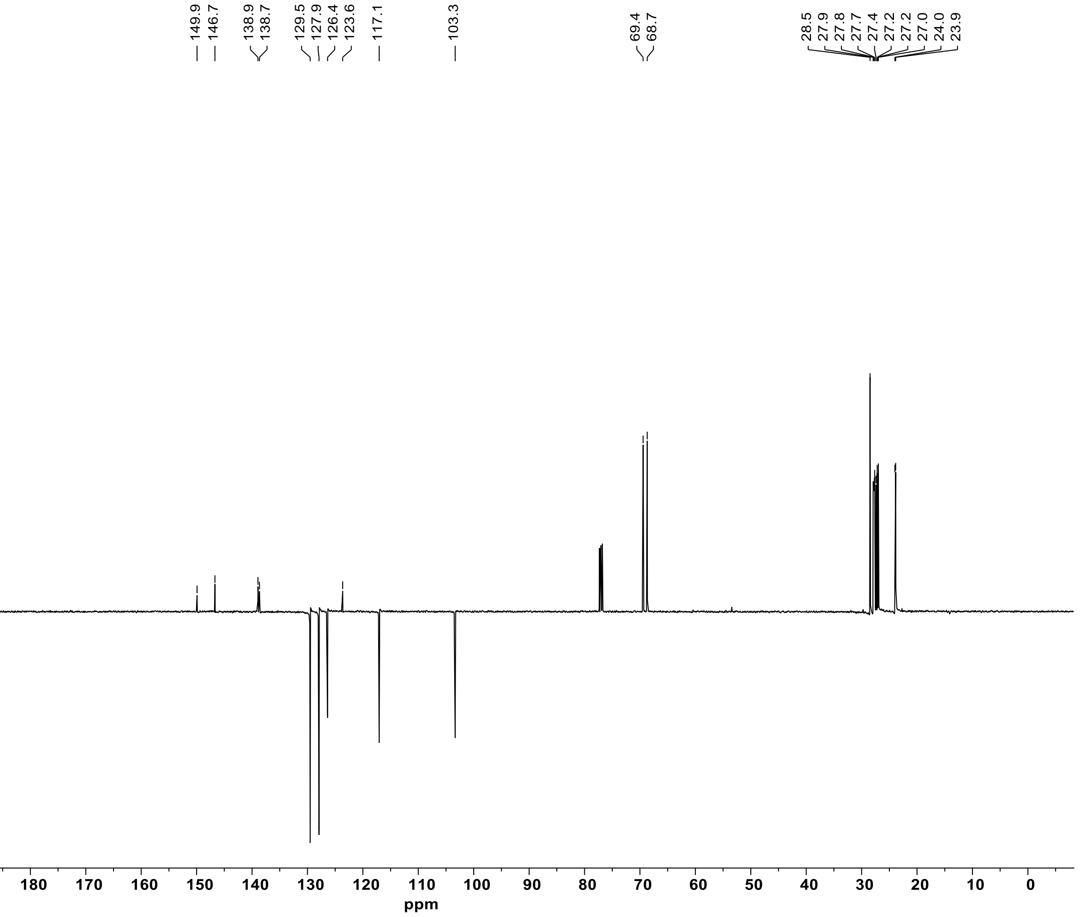

1⁵-(4-methoxyphenyl)-2,15-dioxa-1(1,4)-benzenacyclopentadecaphan-1²-ol S48

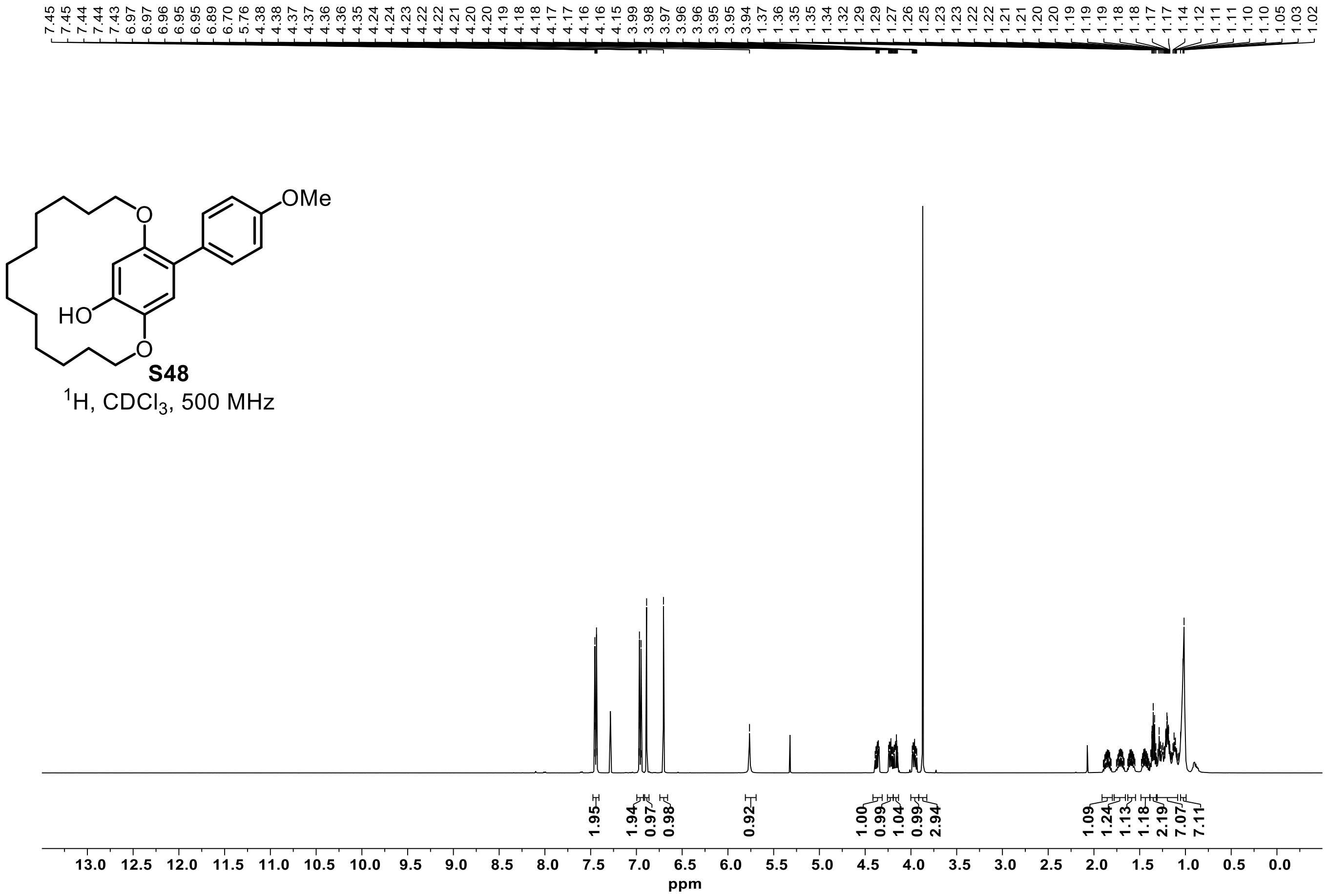

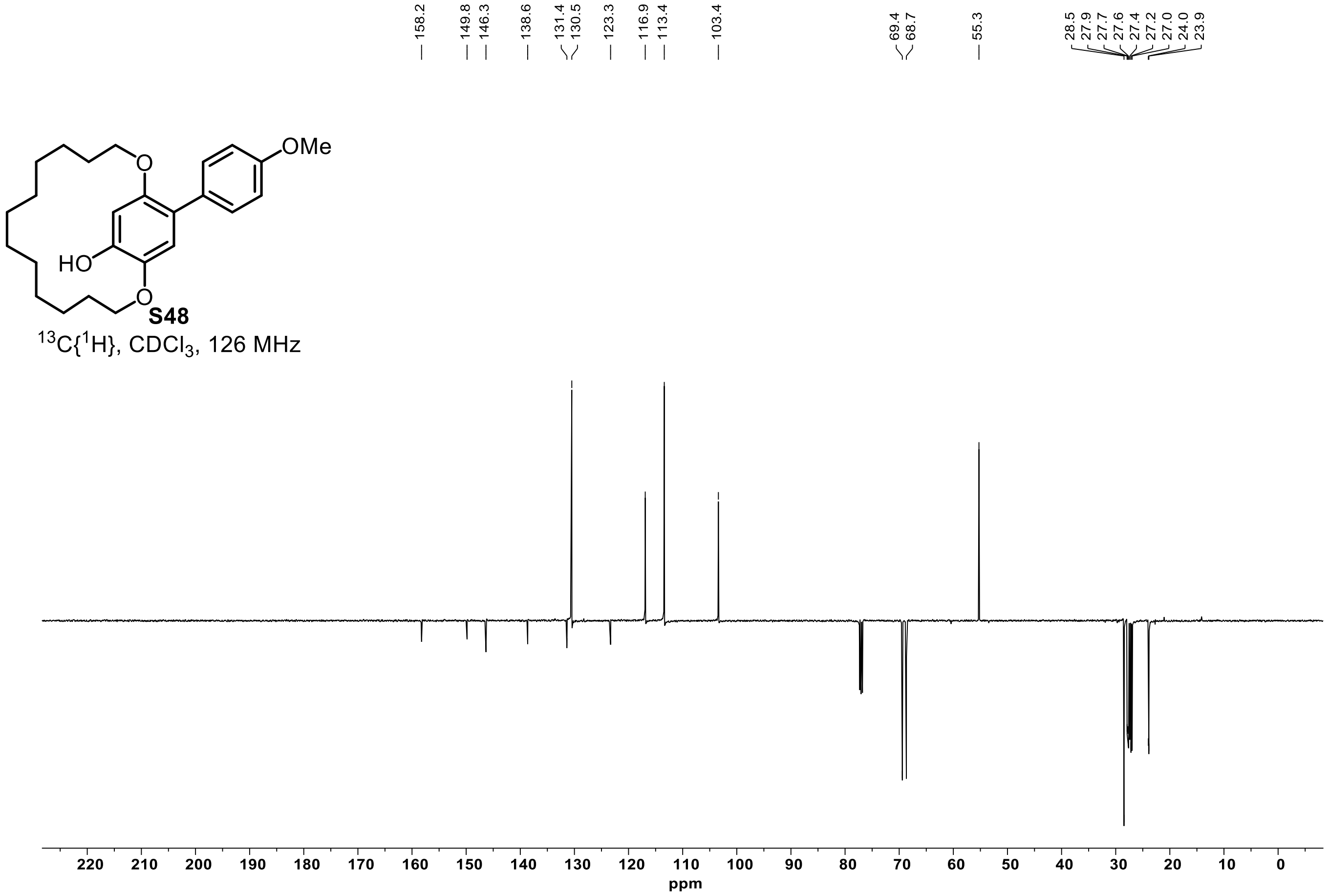

1⁵-(4-(trifluoromethyl)phenyl)-2,15-dioxa-1(1,4)-benzenacyclopentadecaphan-1²-ol S49

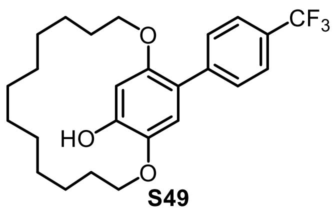

S49

^{1}H, $CDCl_3$, 500 MHz

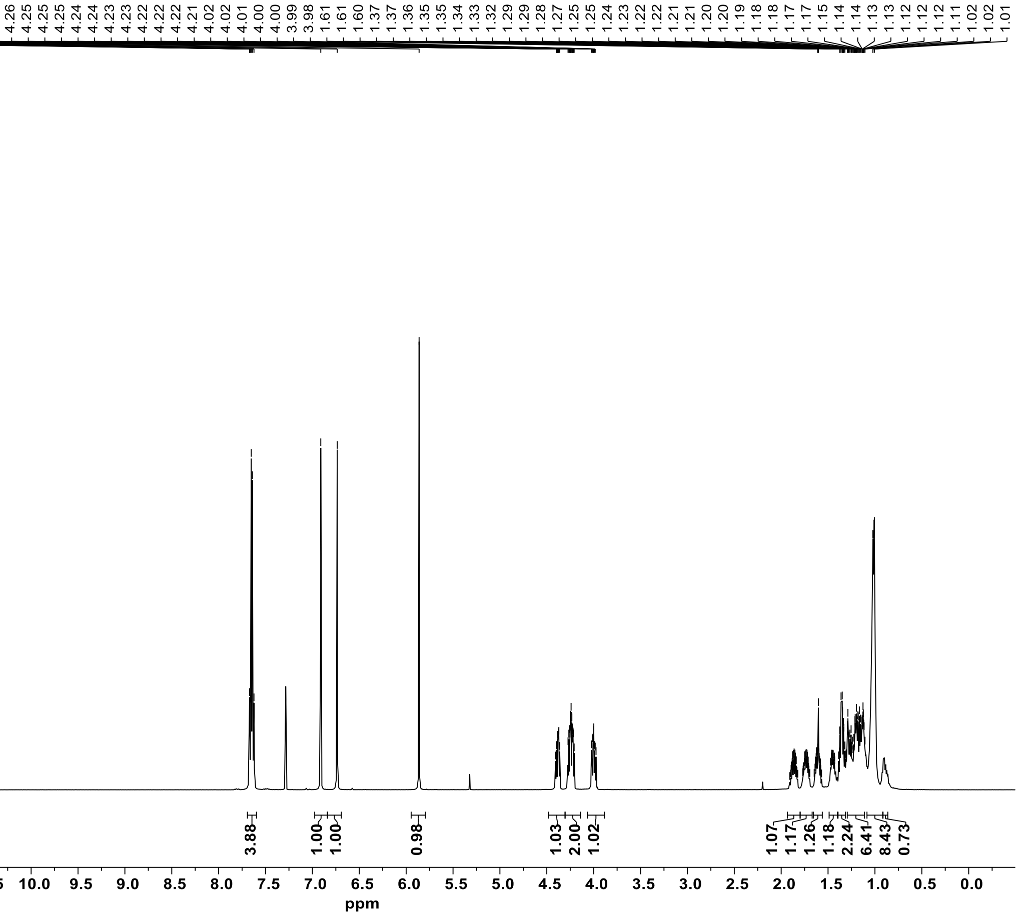

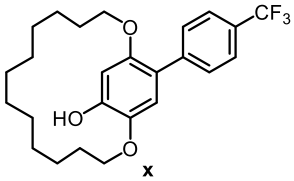

x

$^{13}C\{^{1}H\}$, $CDCl_3$, 126 MHz

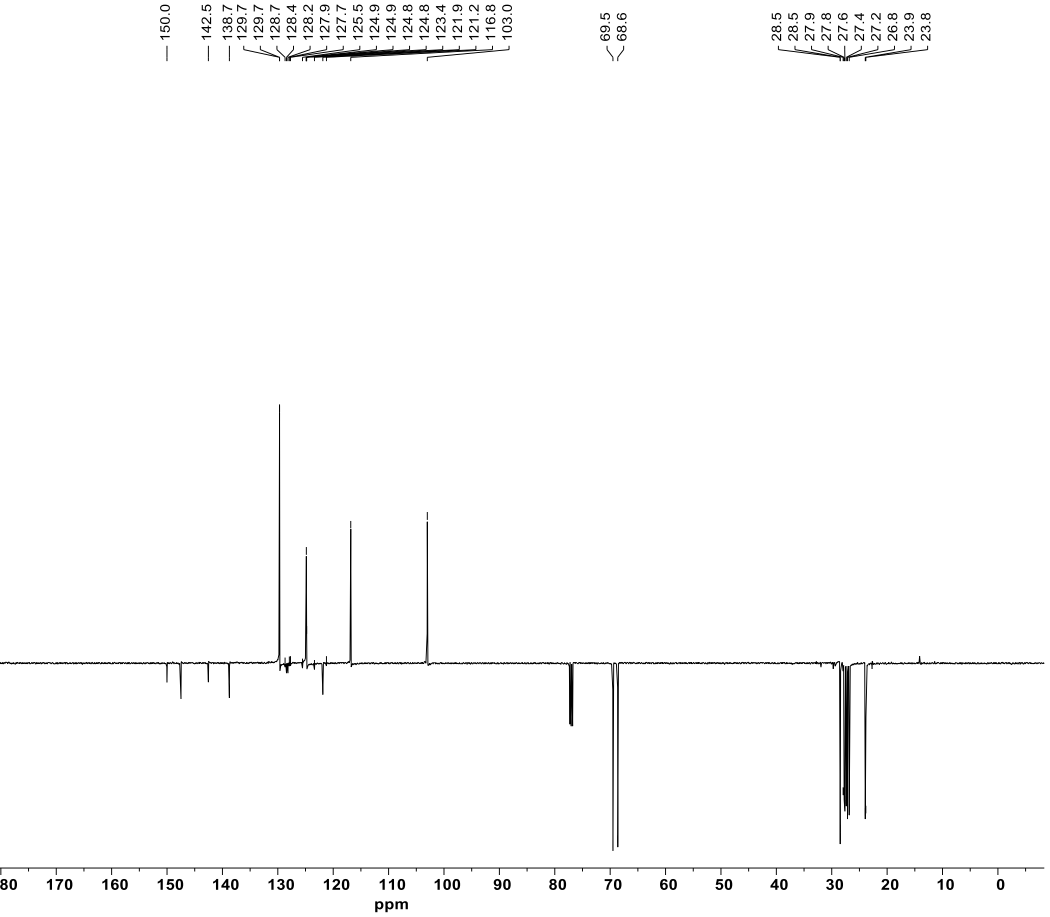

1⁵-(2-methoxyphenyl)-2,15-dioxa-1(1,4)-benzenacyclopentadecaphan-1²-ol S50

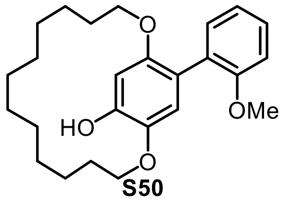

S50

^{1}H, CDCl$_3$, 500 MHz

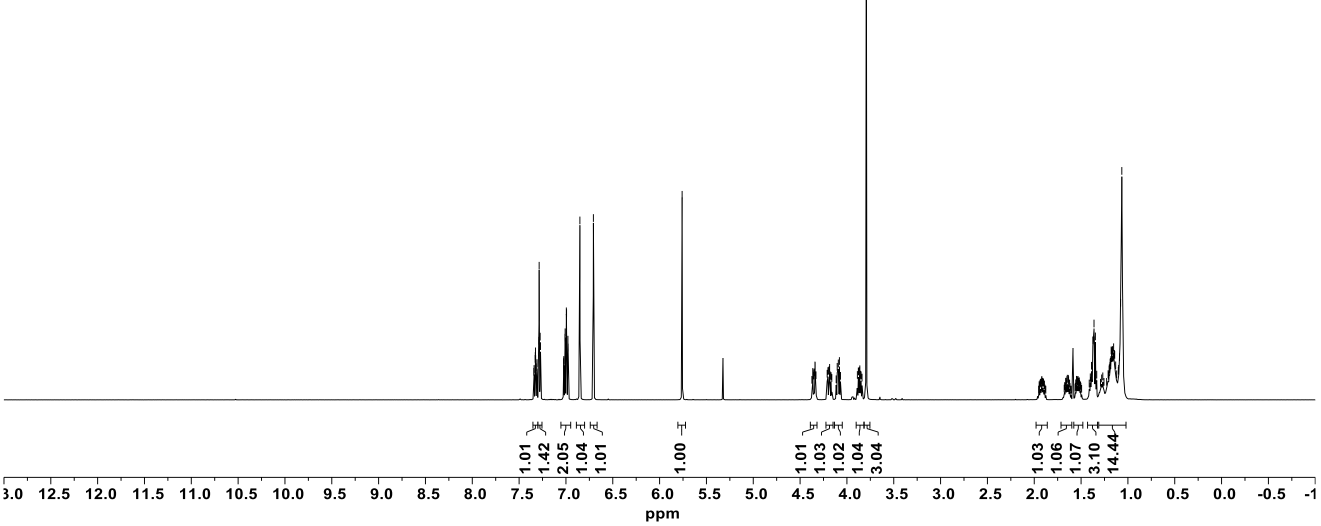

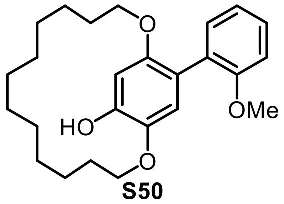

S50

^{13}C{^{1}H}, CDCl$_3$, 126 MHz

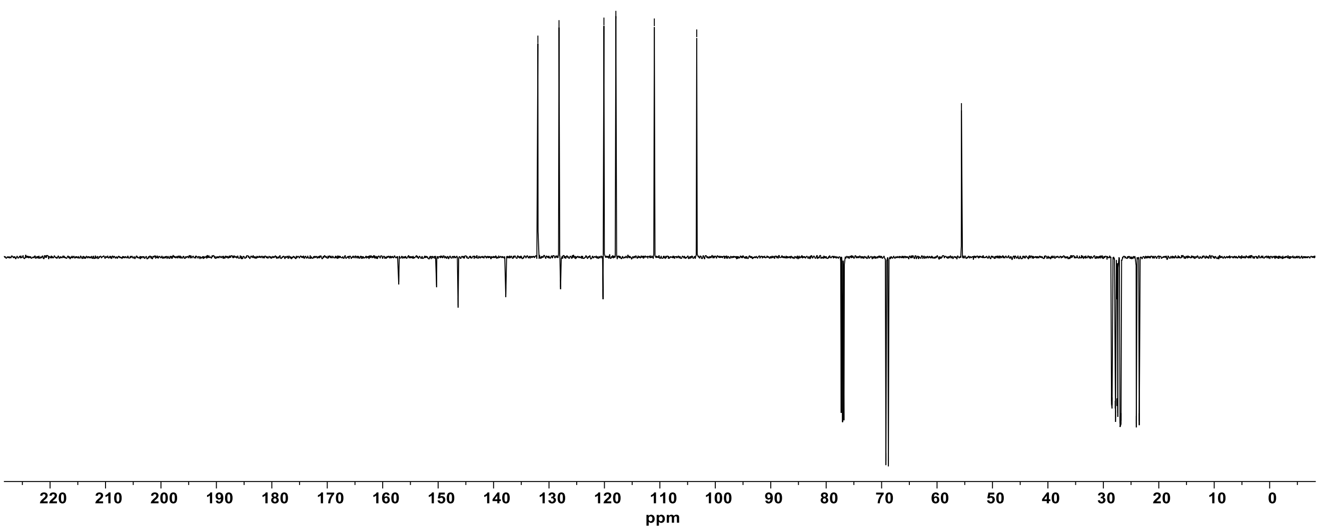

1[5]-(naphthalen-1-yl)-2,15-dioxa-1(1,4)-benzenacyclopentadecaphan-1[2]-ol S51

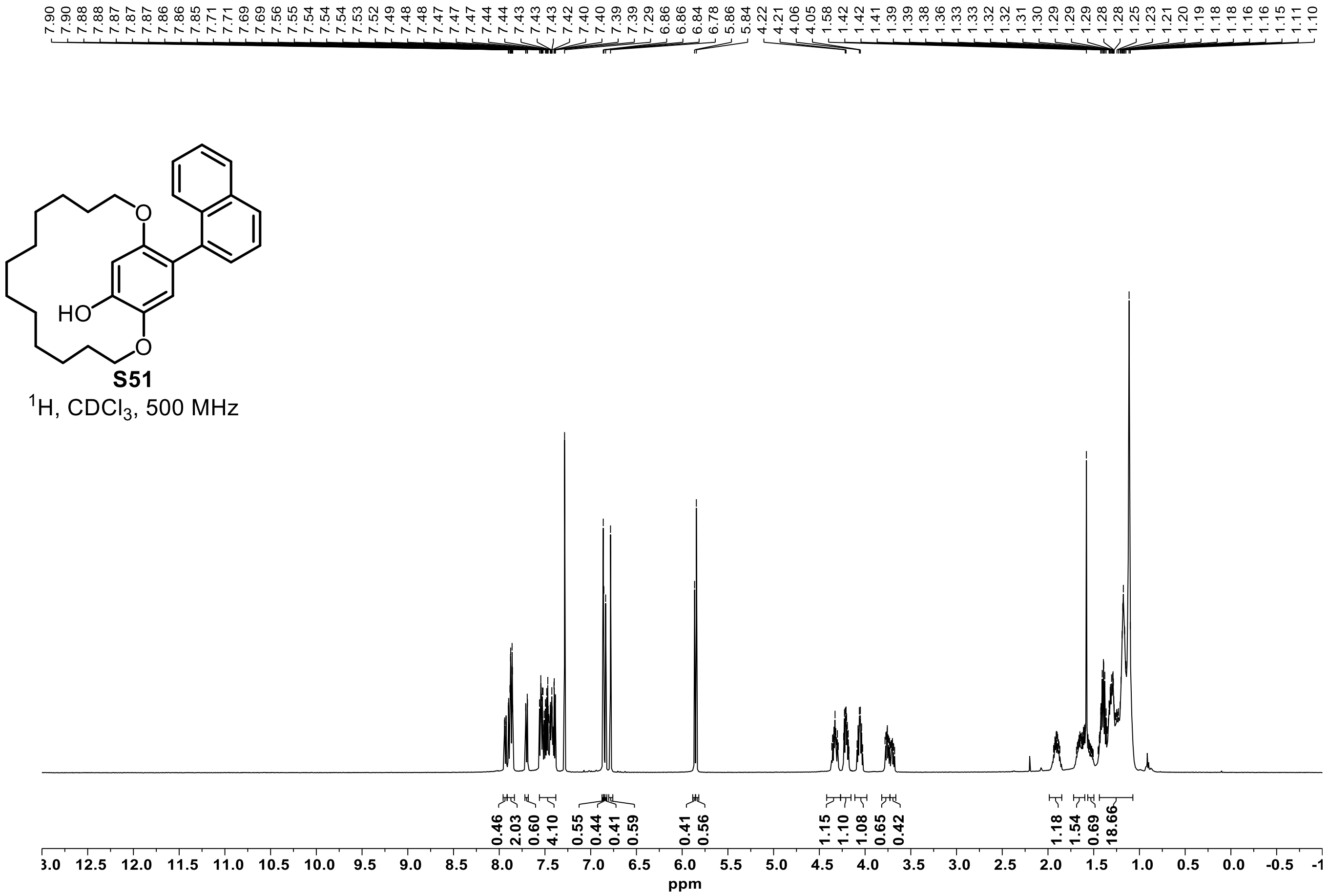

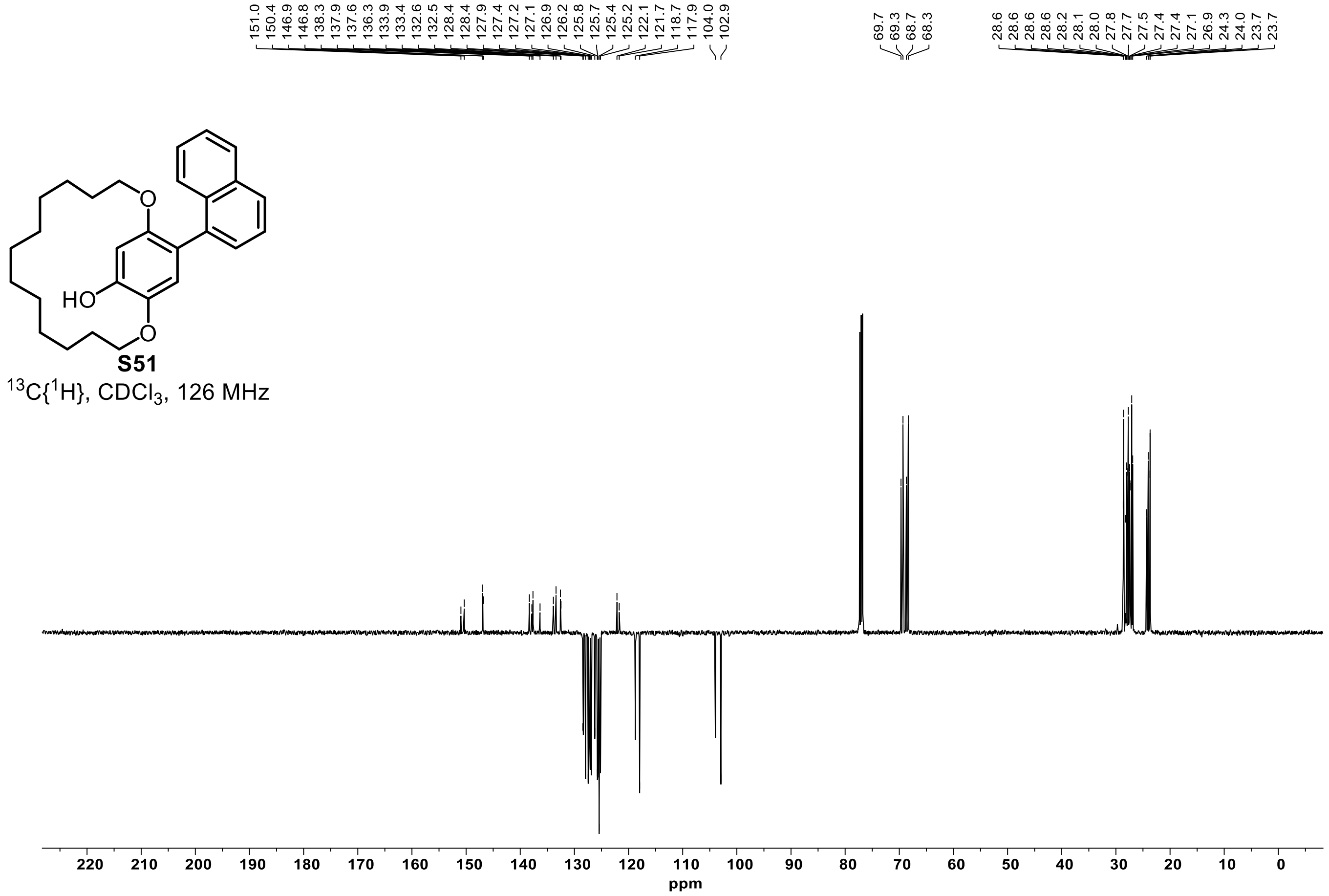

1⁵-(naphthalen-2-yl)-2,15-dioxa-1(1,4)-benzenacyclopentadecaphan-1²-ol S52

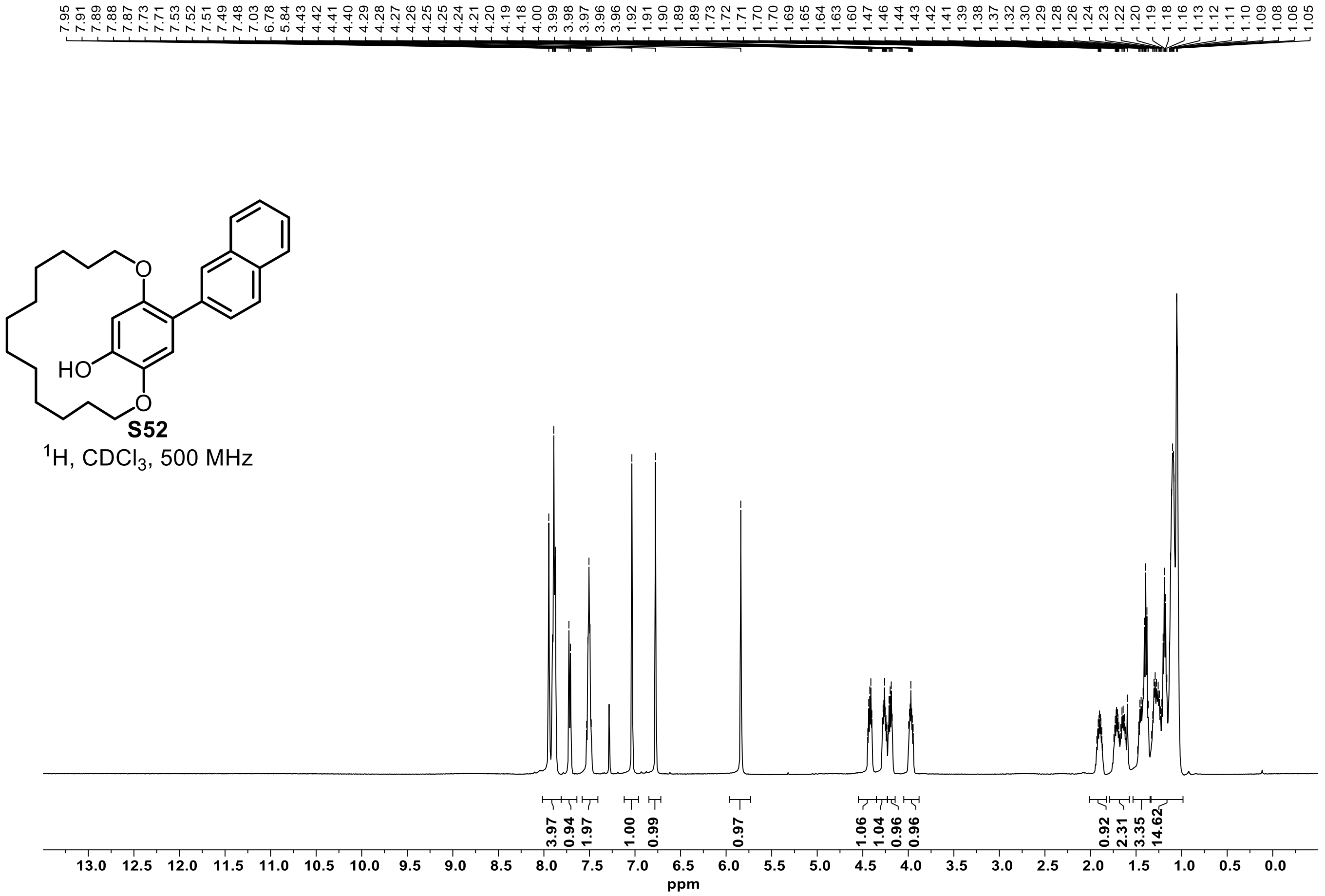

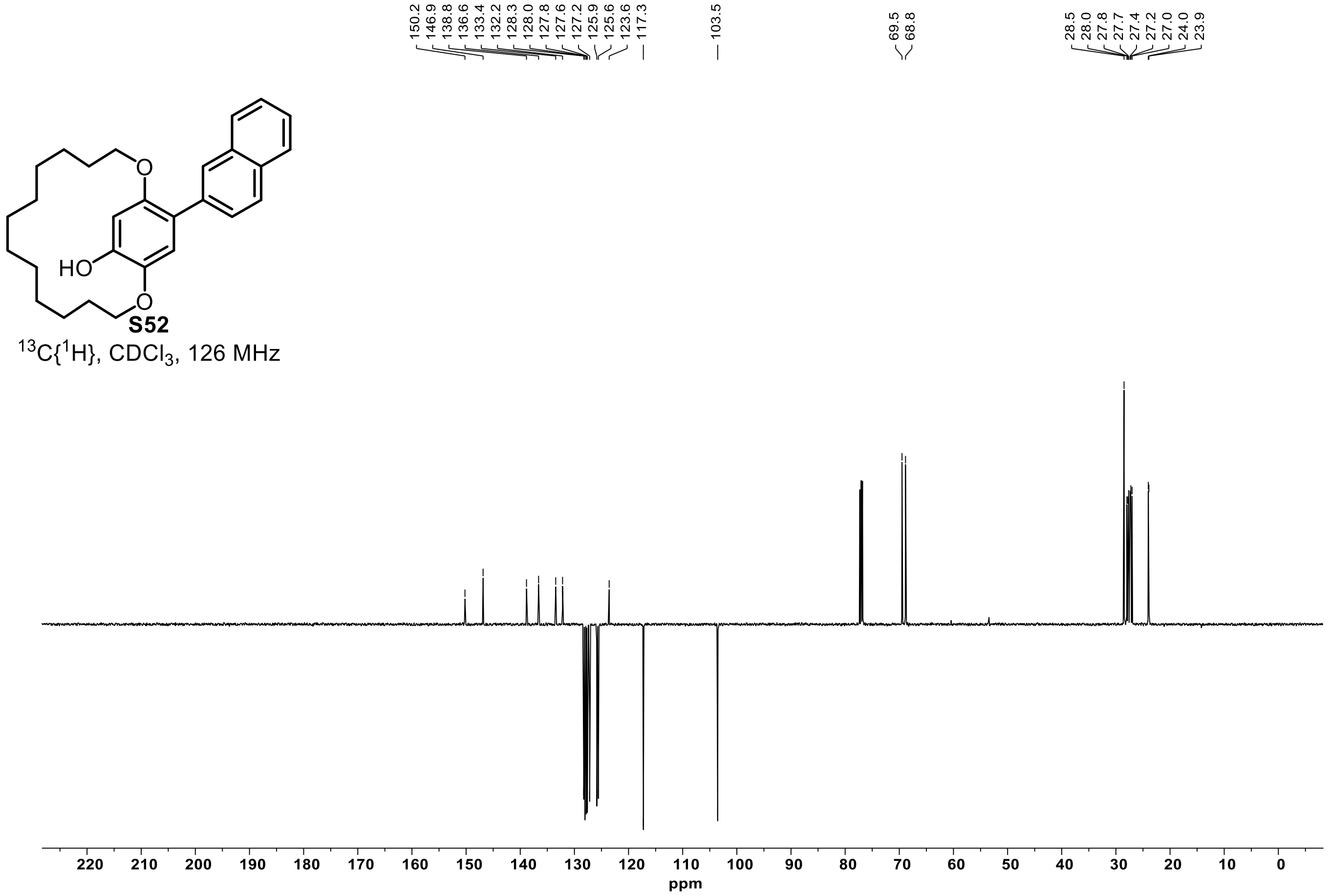

1⁵-(pyridin-3-yl)-2,15-dioxa-1(1,4)-benzenacyclopentadecaphan-1²-ol S53

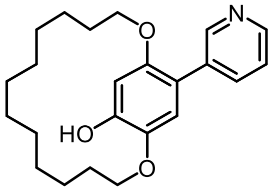

S53

^{1}H, $CDCl_3$, 500 MHz

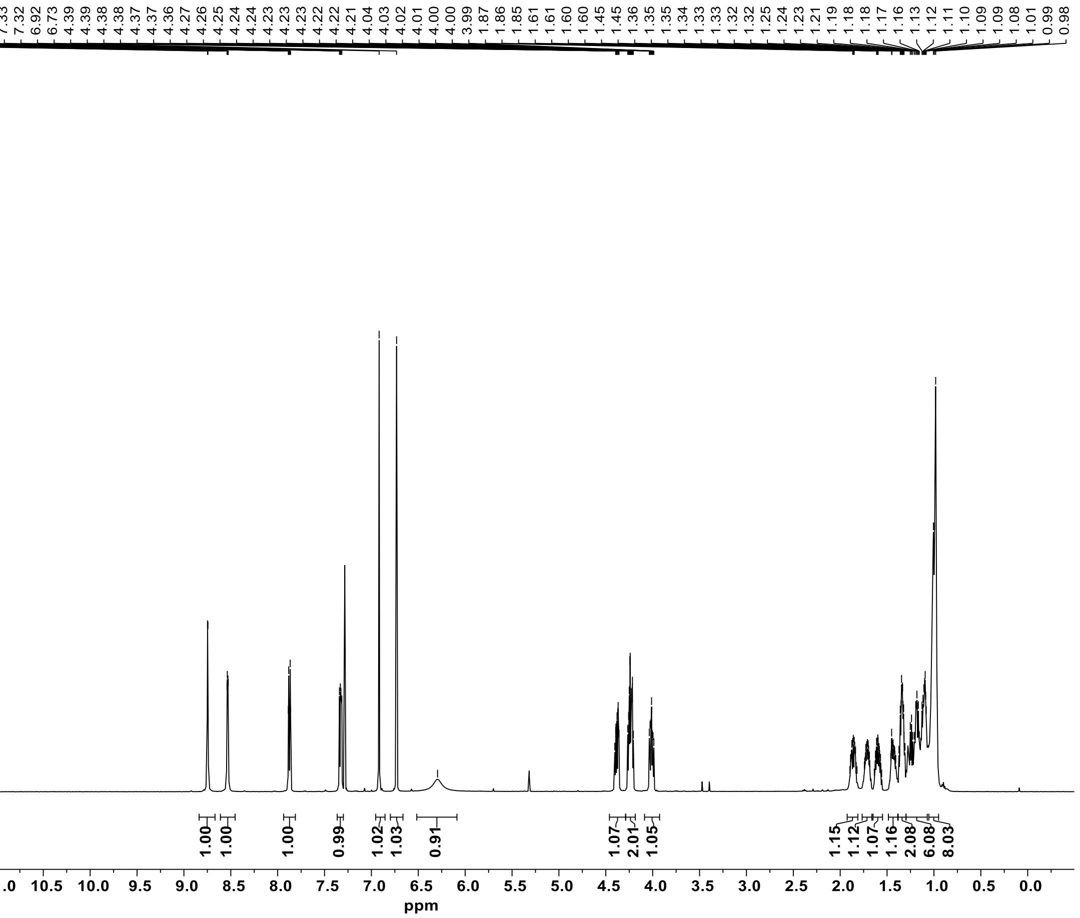

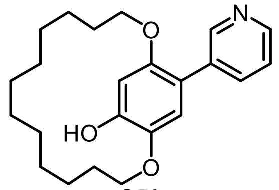

S53

$^{13}C\{^{1}H\}$, $CDCl_3$, 126 MHz

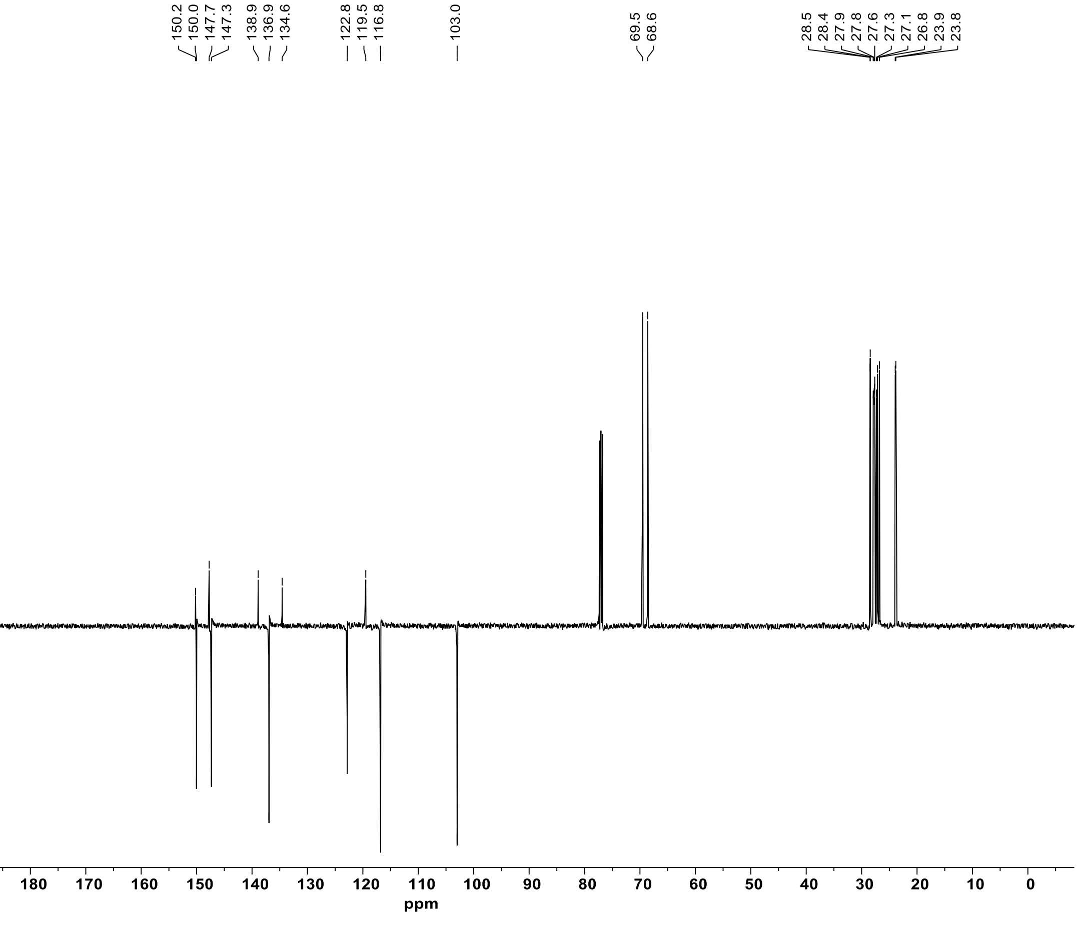

1⁵-(furan-2-yl)-2,15-dioxa-1(1,4)-benzenacyclopentadecaphan-1²-ol S54

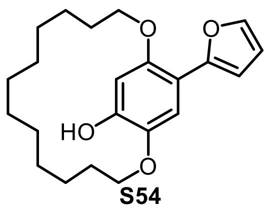

S54

^{1}H, CDCl$_3$, 500 MHz

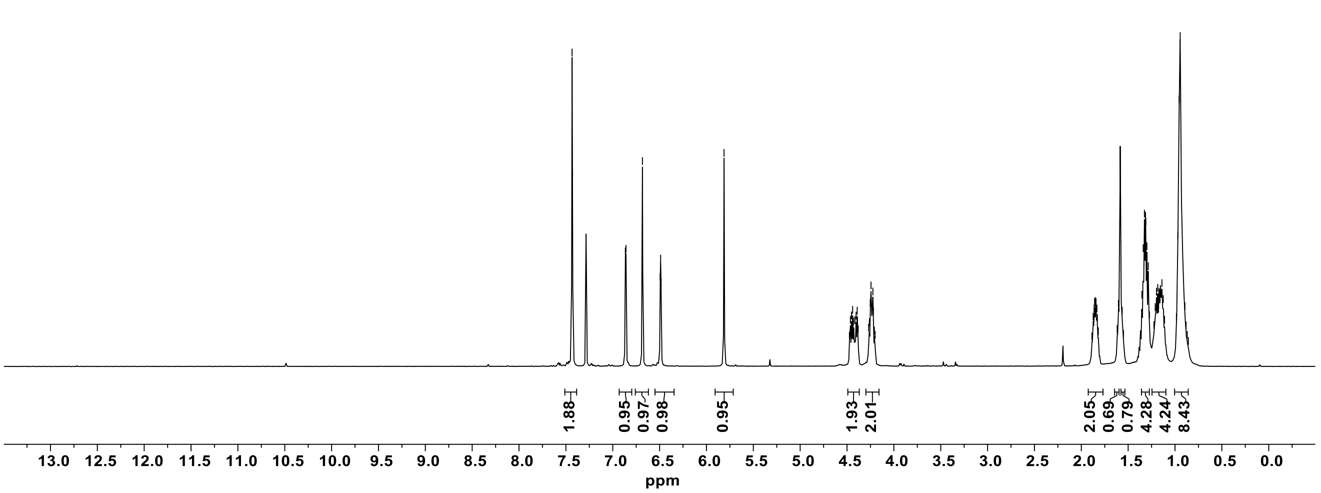

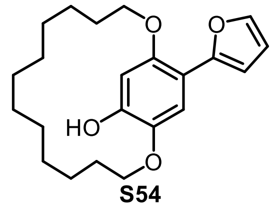

S54

^{13}C{^{1}H}, CDCl$_3$, 126 MHz

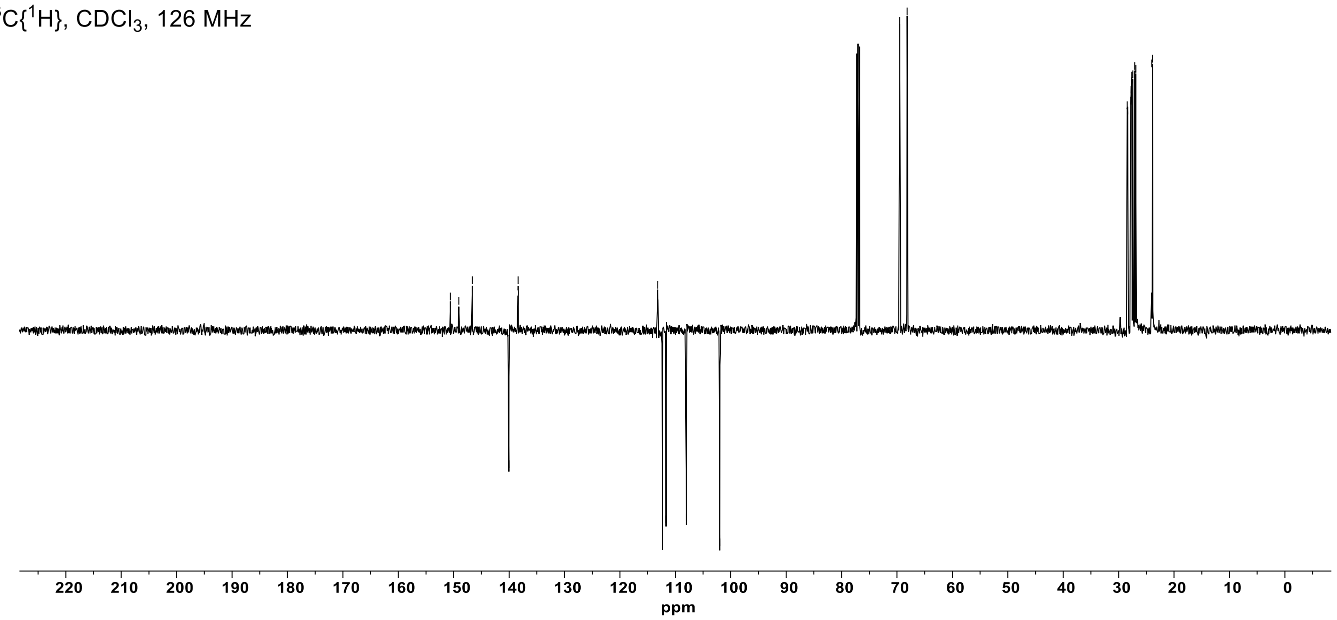

1^5-(thiophen-3-yl)-2,15-dioxa-1(1,4)-benzenacyclopentadecaphan-1^2-ol S55

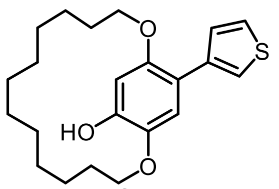

S55

^1H, $CDCl_3$, 500 MHz

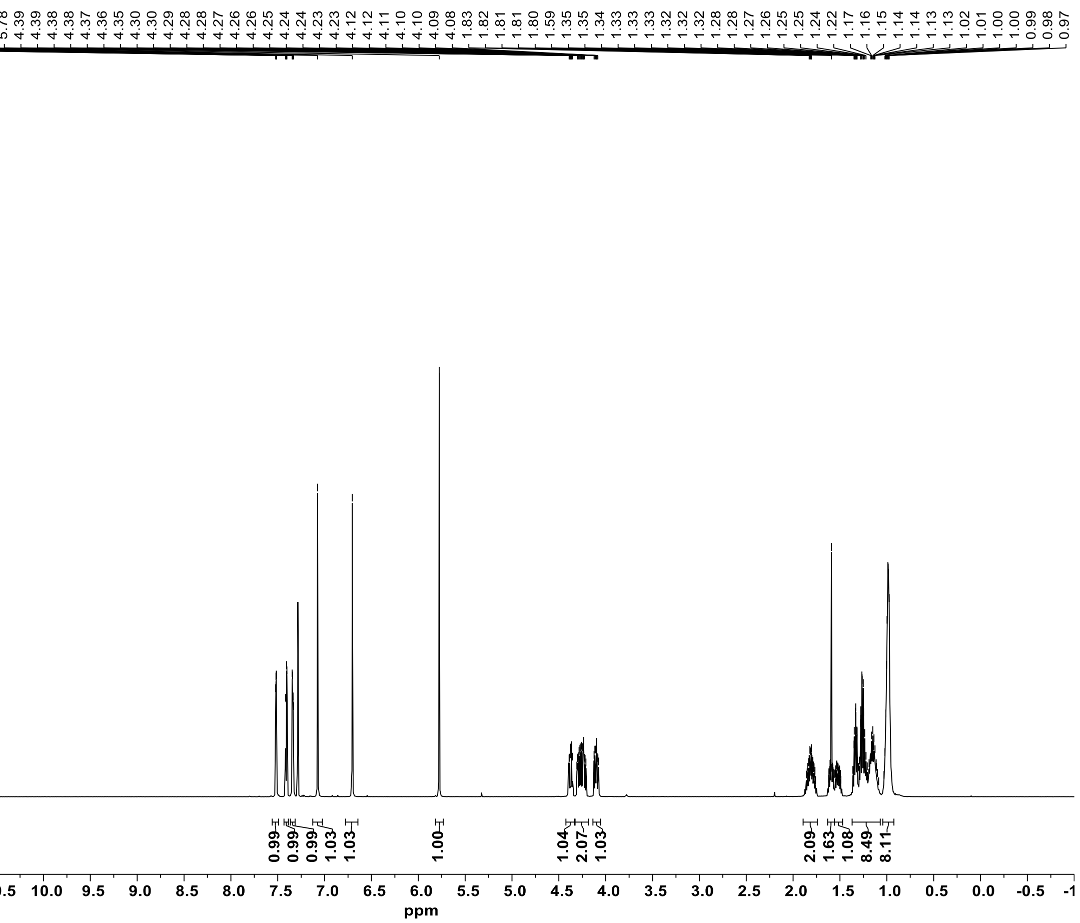

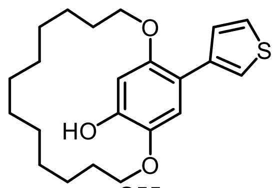

S55

$^{13}C\{^1H\}$, $CDCl_3$, 126 MHz

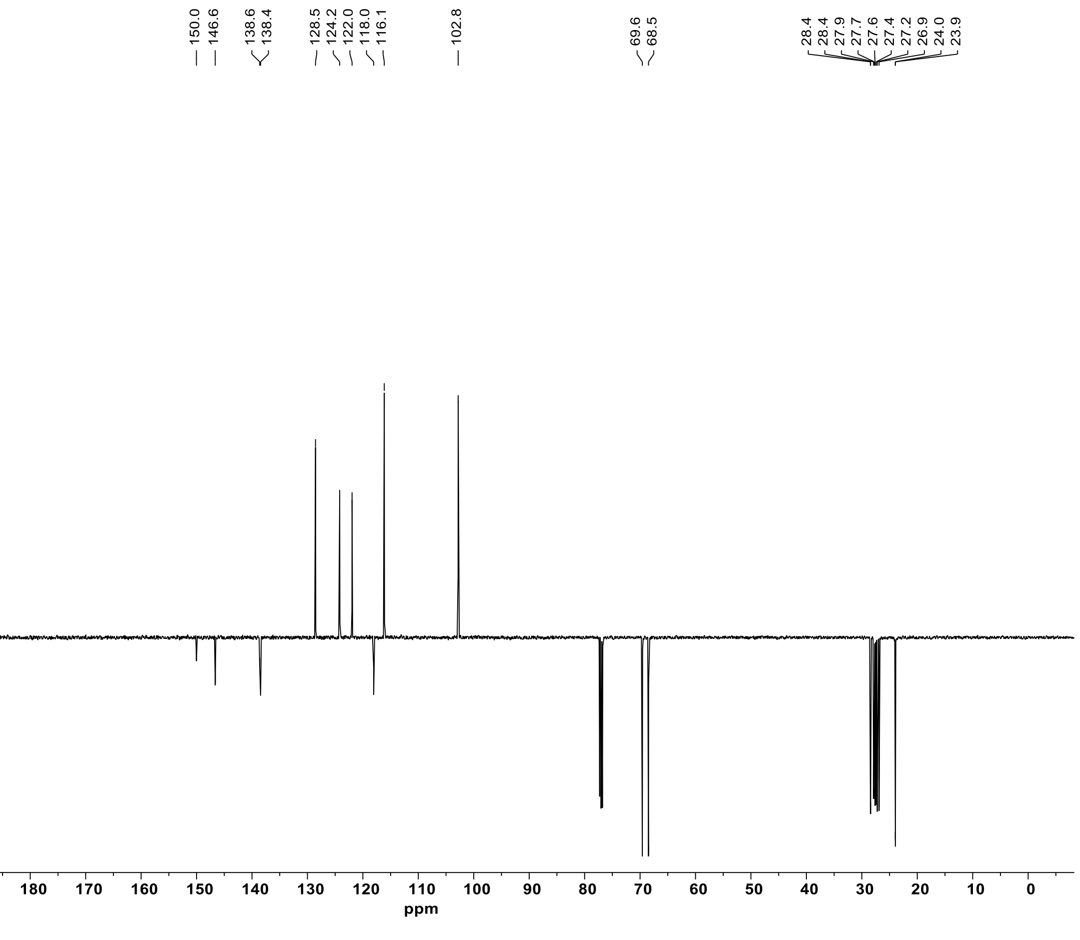

***tert*-butyl 3-(1⁵-hydroxy-2,15-dioxa-1(1,4)-benzenacyclopentadecaphane-1²-yl)-5-methoxy-1H-indole-1-carboxylate S56**

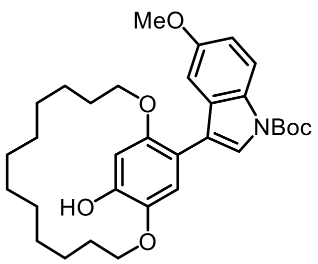

S56
^{1}H, $CDCl_3$, 500 MHz

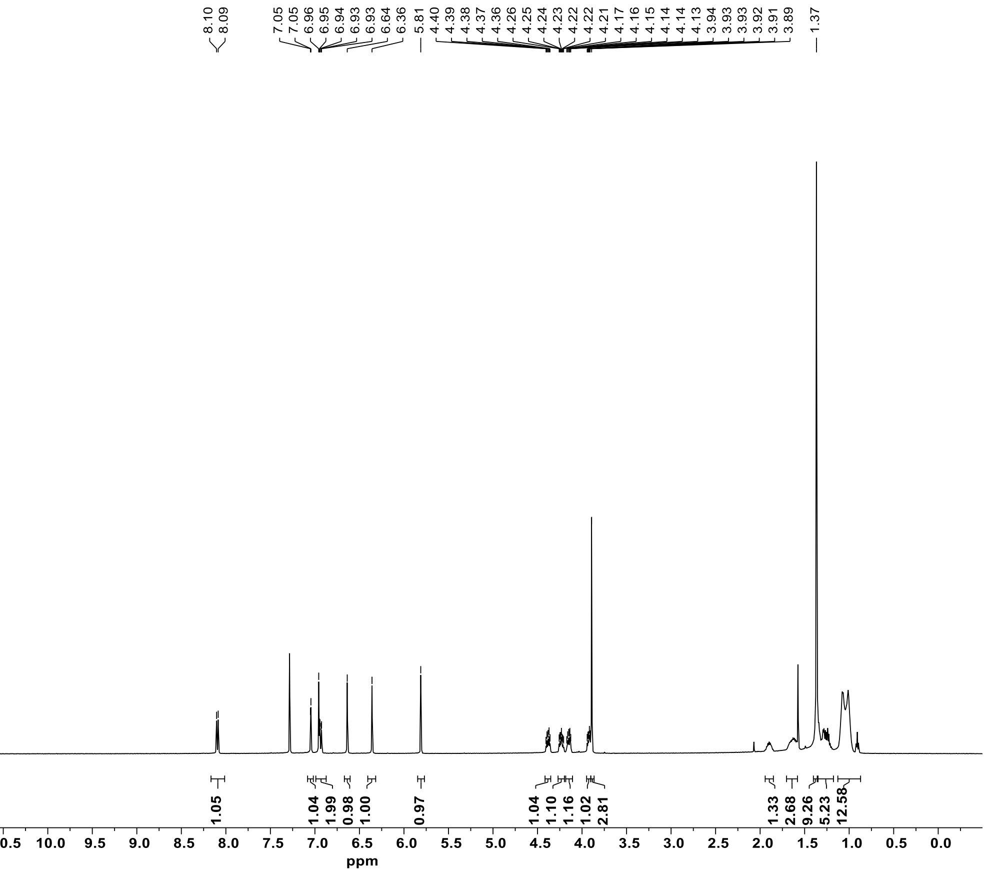

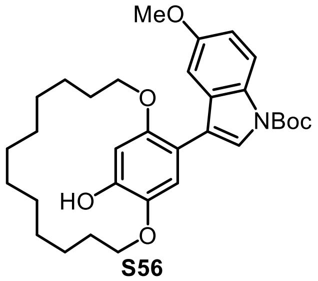

S56
$^{13}C\{^{1}H\}$, $CDCl_3$, 126 MHz

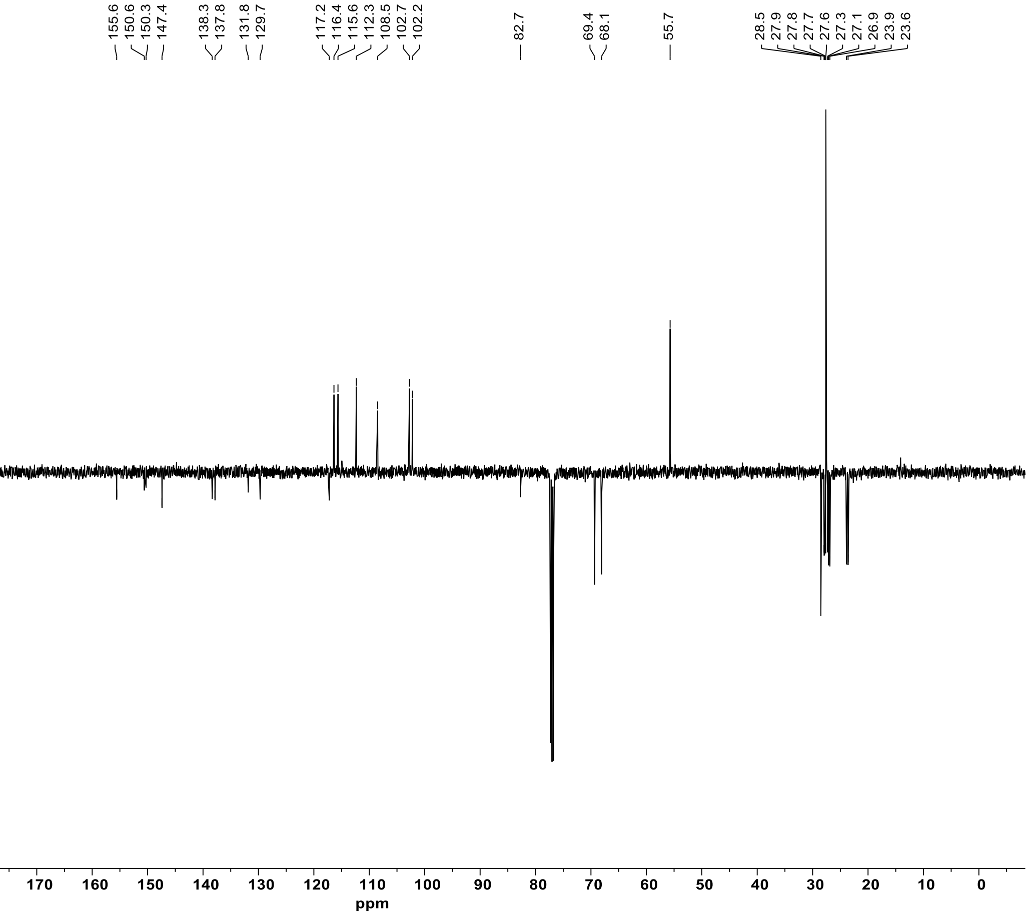

1[5]-(pyren-1-yl)-2,15-dioxa-1(1,4)-benzenacyclopentadecaphan-1[2]-ol S57

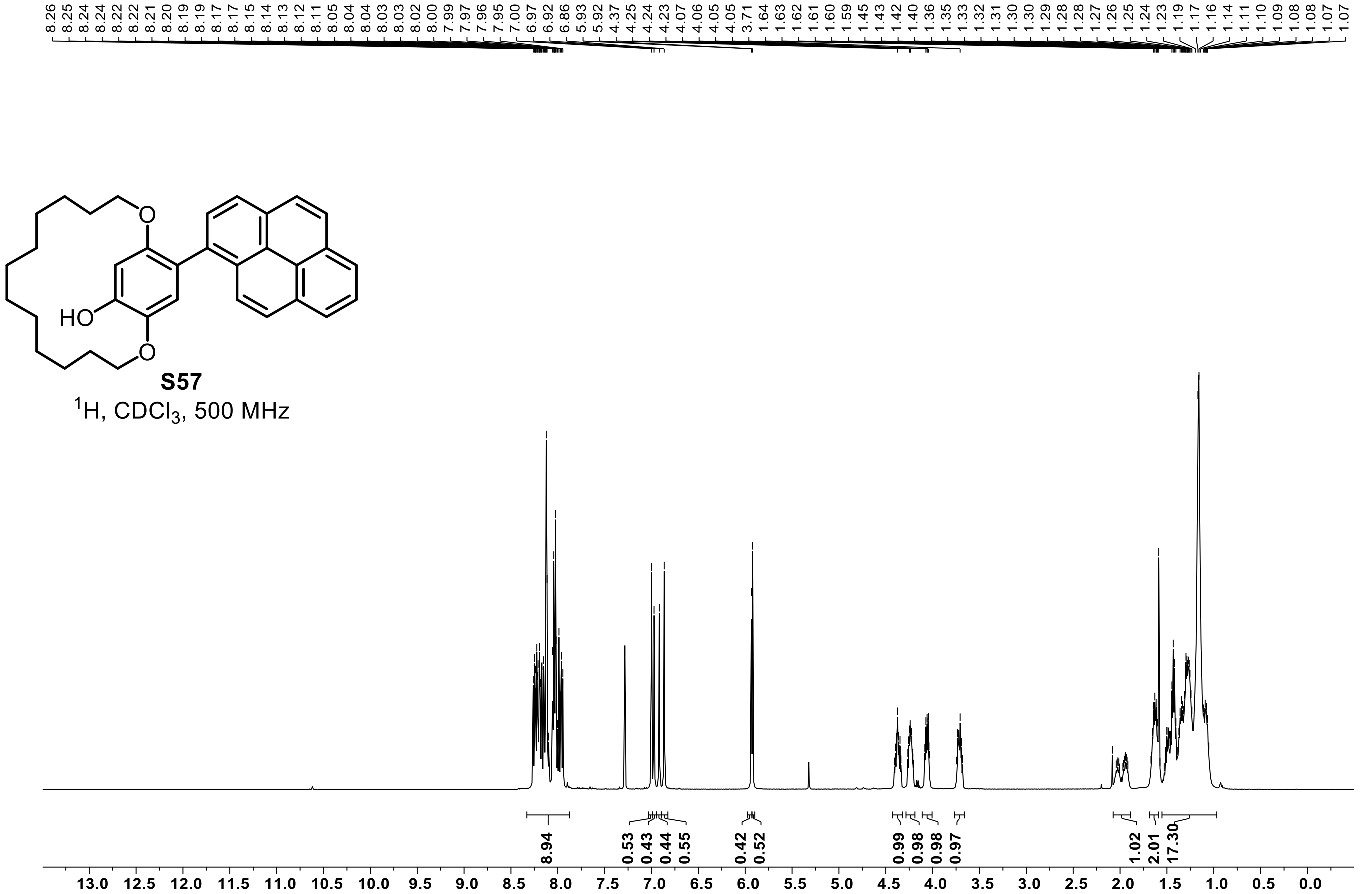

S57

^{1}H, $CDCl_3$, 500 MHz

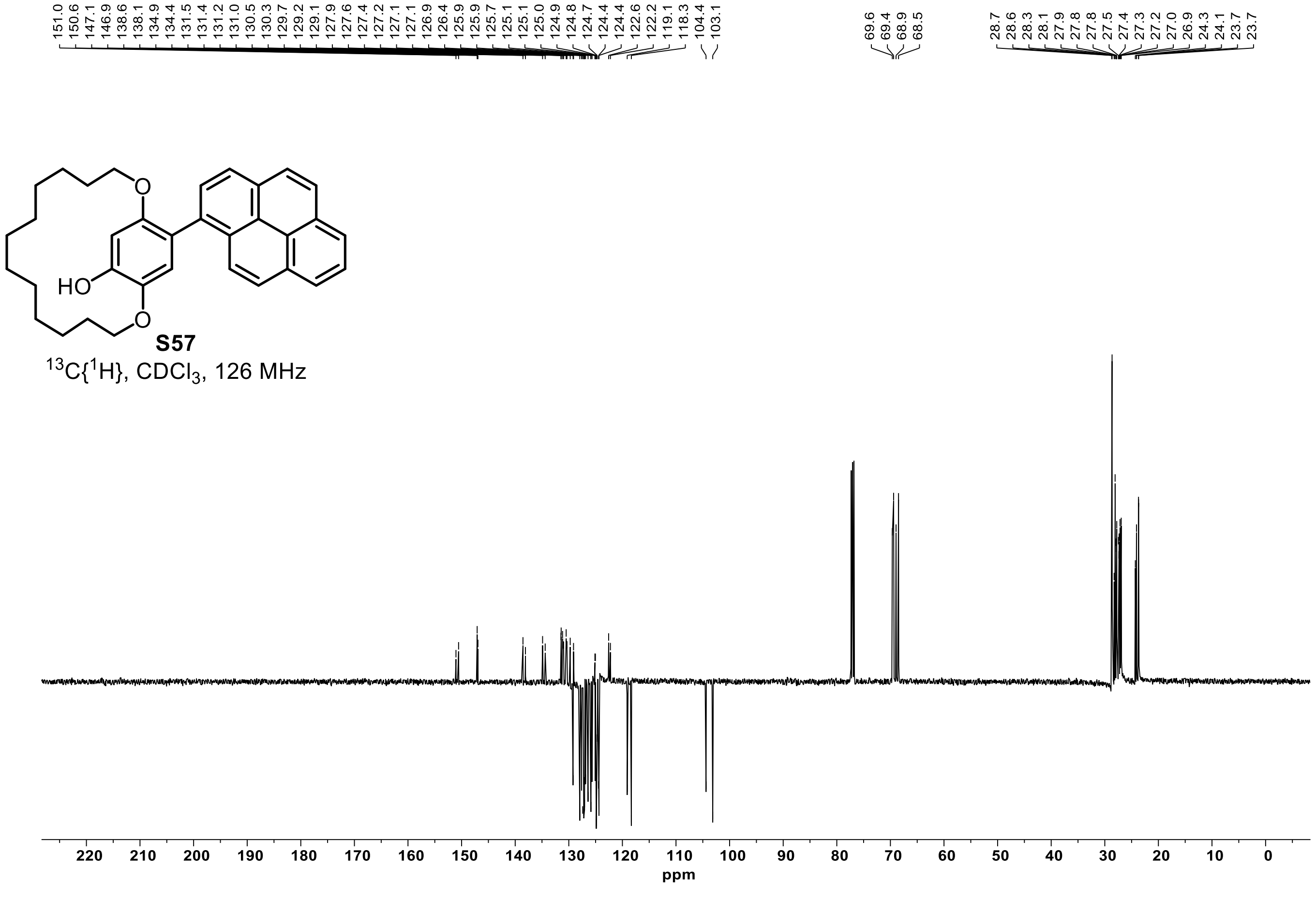

S57

$^{13}C\{^{1}H\}$, $CDCl_3$, 126 MHz

2,15-dioxa-1(1,4)-naphthalenacyclopentadecaphan-1^2-ol 36

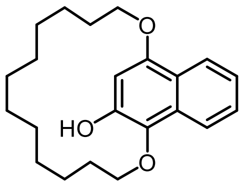

36

^{1}H, $CDCl_3$, 500 MHz

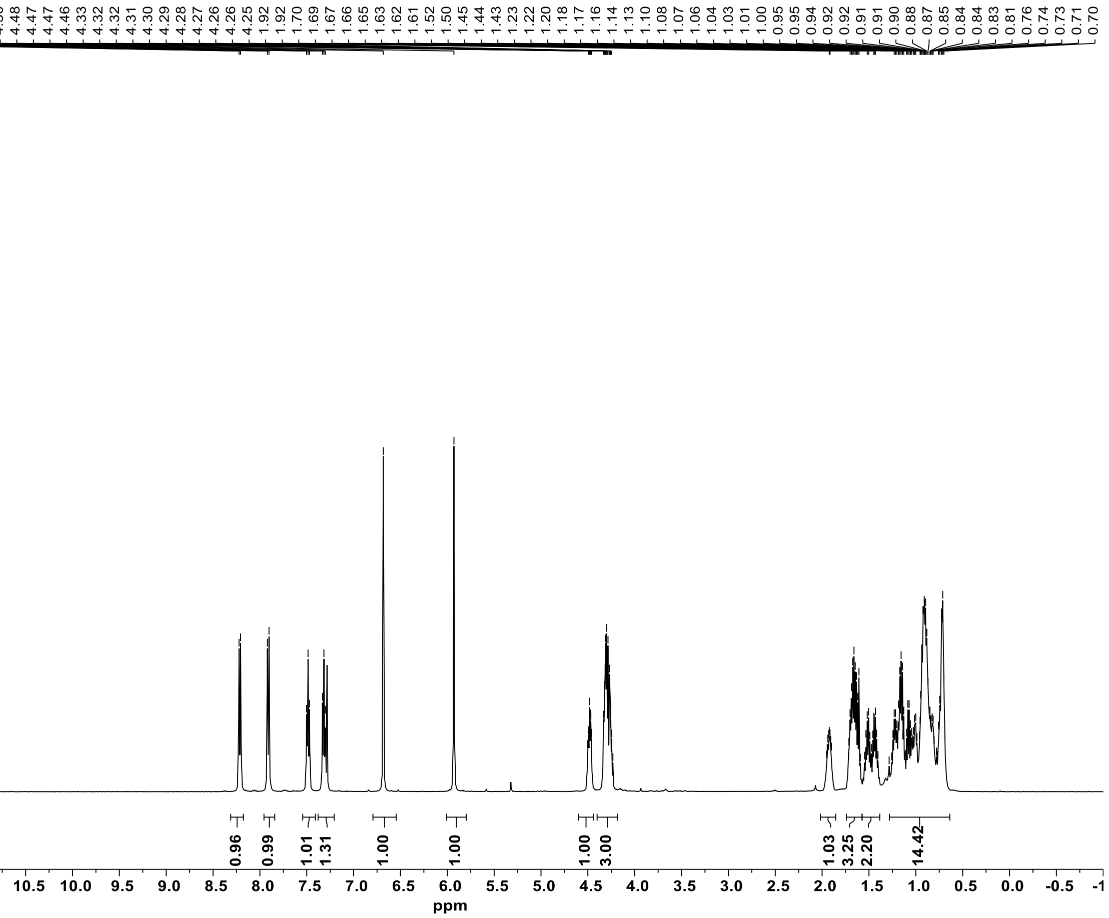

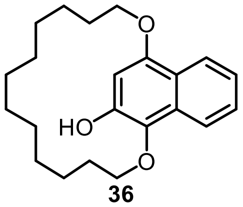

36

$^{13}C\{^1H\}$, $CDCl_3$, 126 MHz

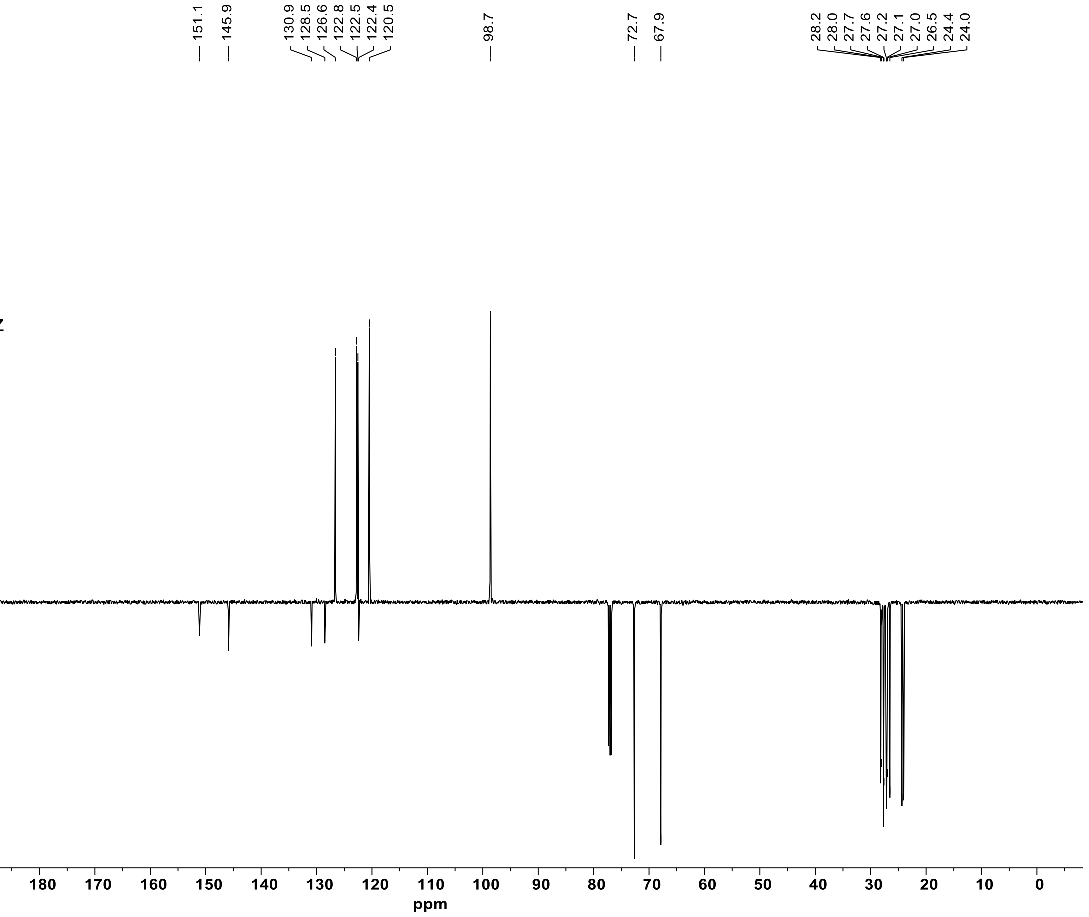

1^5-bromo-2,6,11,15-tetraoxa-1(1,4)-benzenacyclopentadecaphan-1^2-ol S58

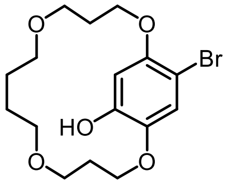

S58
^1H, $CDCl_3$, 500 MHz

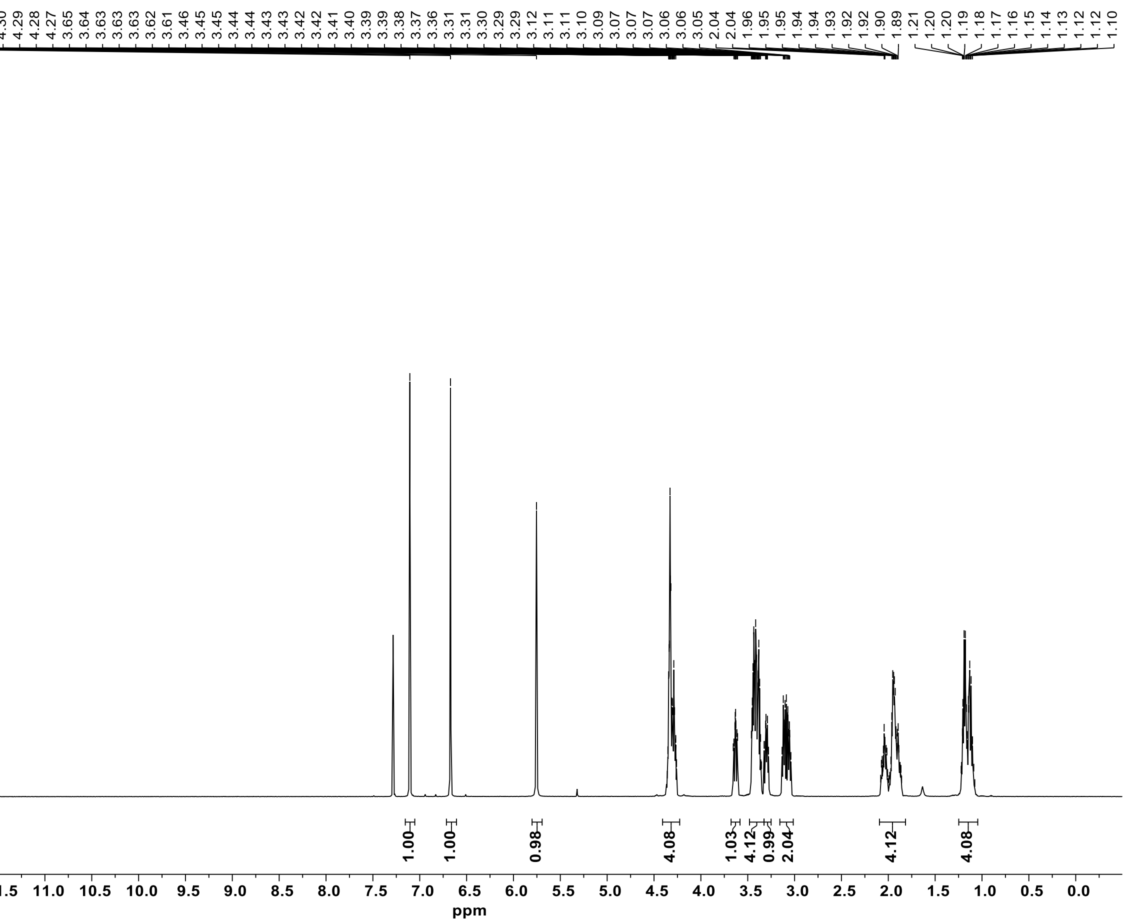

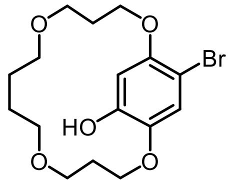

S58
$^{13}C\{^1H\}$, $CDCl_3$, 126 MHz

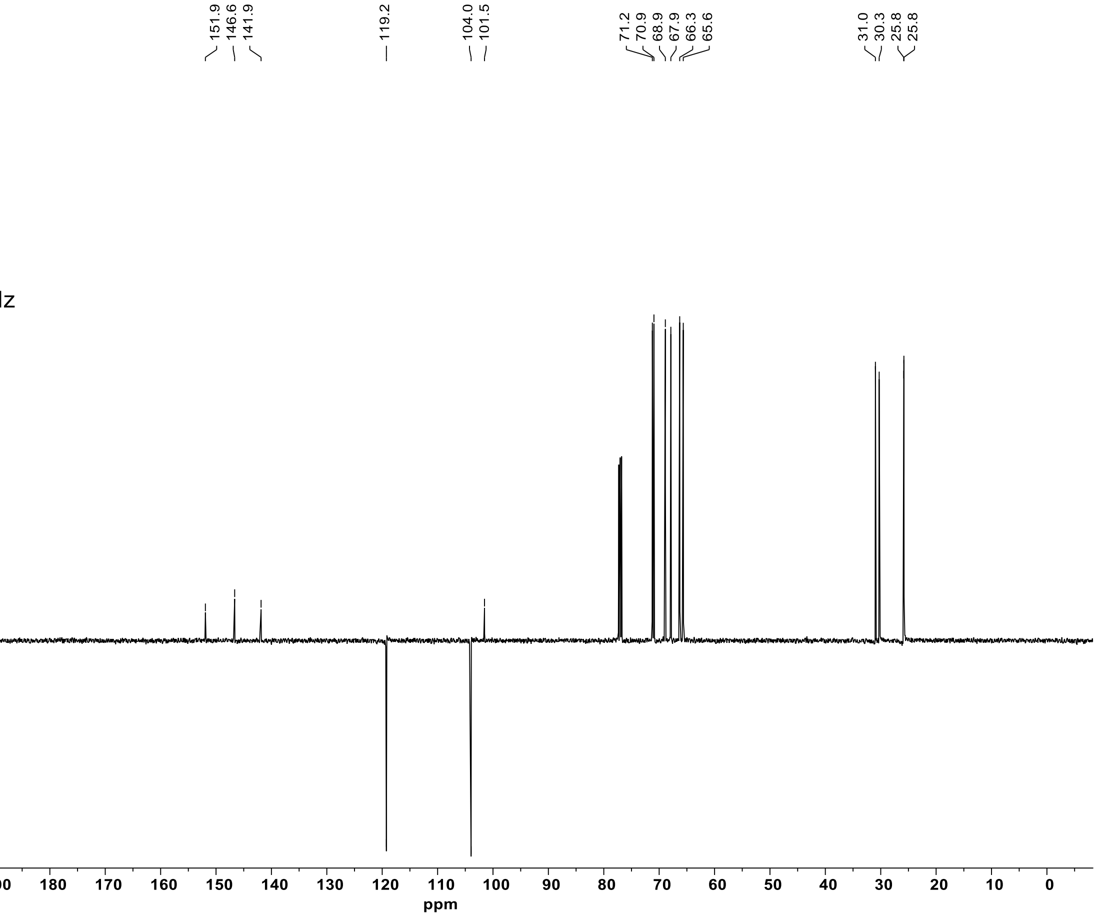

1[5]-bromo-2,16-dioxa-1(1,4)-benzenacyclohexadecaphan-1[2]-ol S59

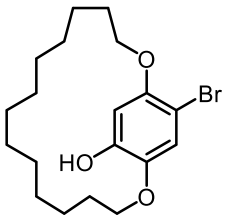

S59

^{1}H, $CDCl_3$, 500 MHz

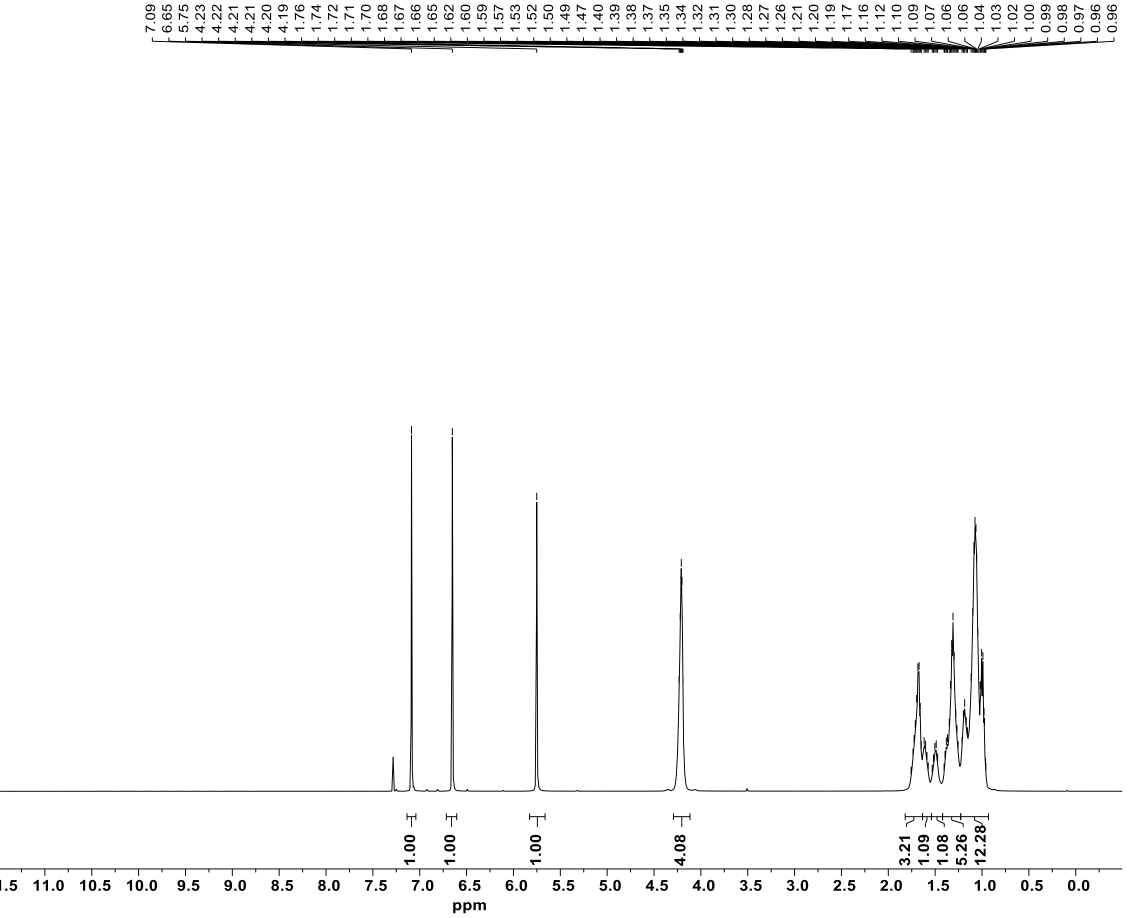

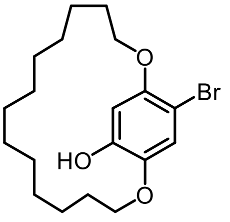

S59

$^{13}C\{^1H\}$, $CDCl_3$, 126 MHz

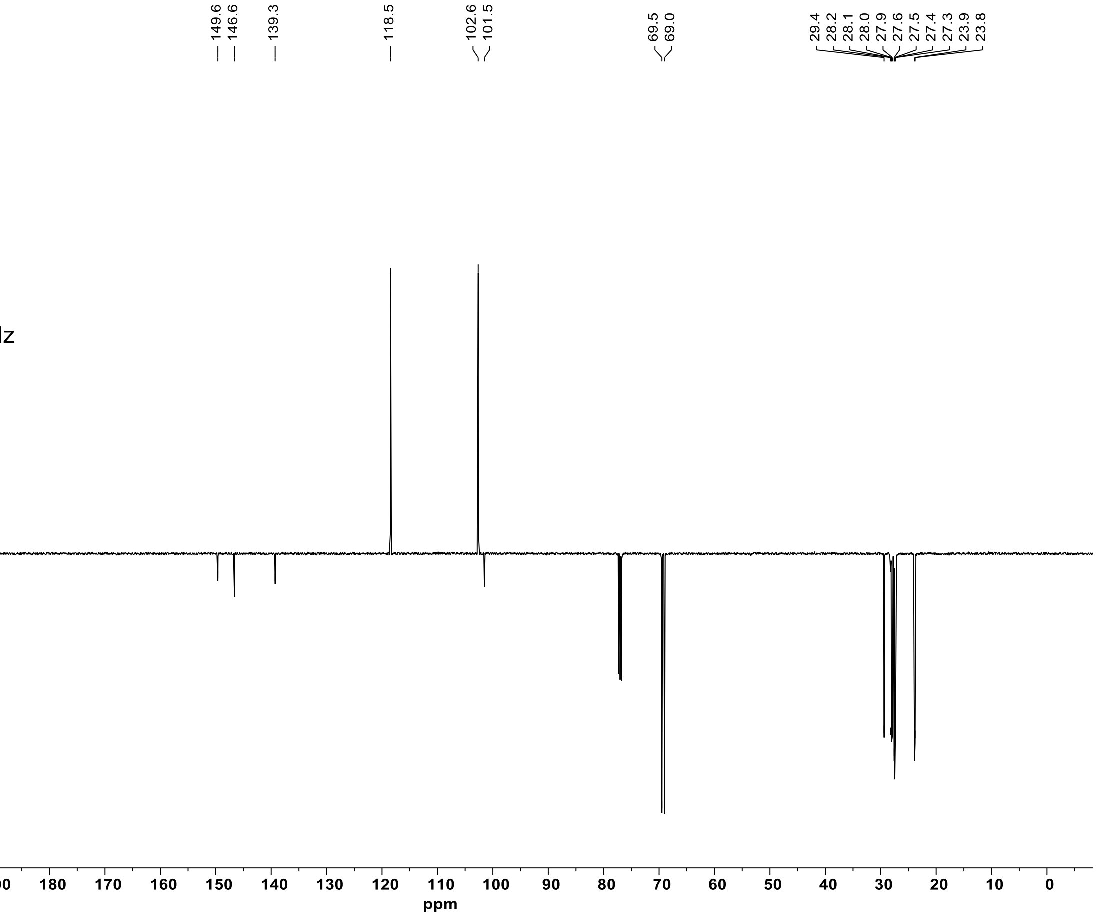

1^5-phenyl-2,16-dioxa-1(1,4)-benzenacyclohexadecaphan-1^2-ol S60

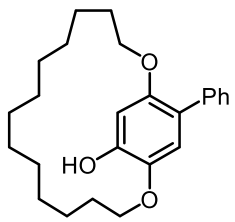

S60

^{1}H, $CDCl_3$, 500 MHz

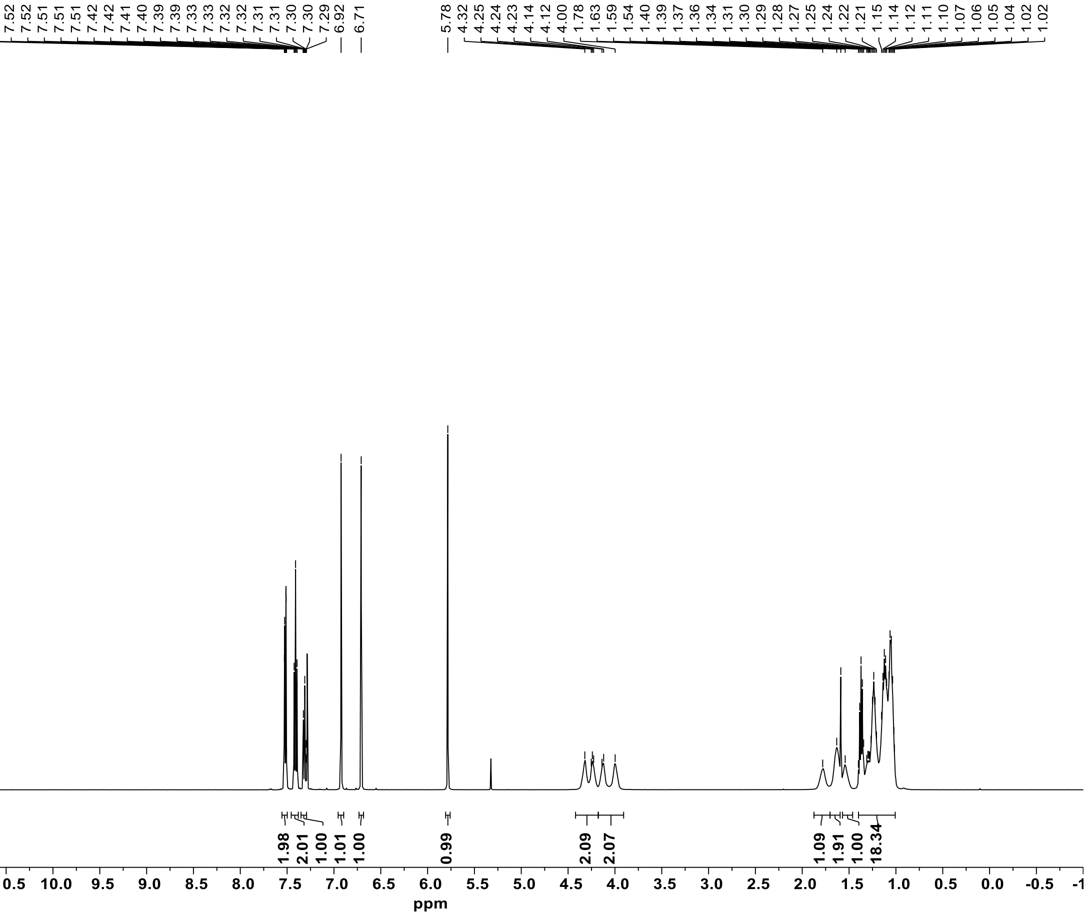

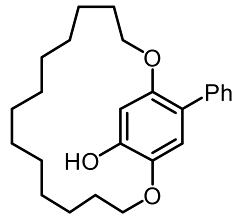

S60

$^{13}C\{^1H\}$, $CDCl_3$, 126 MHz

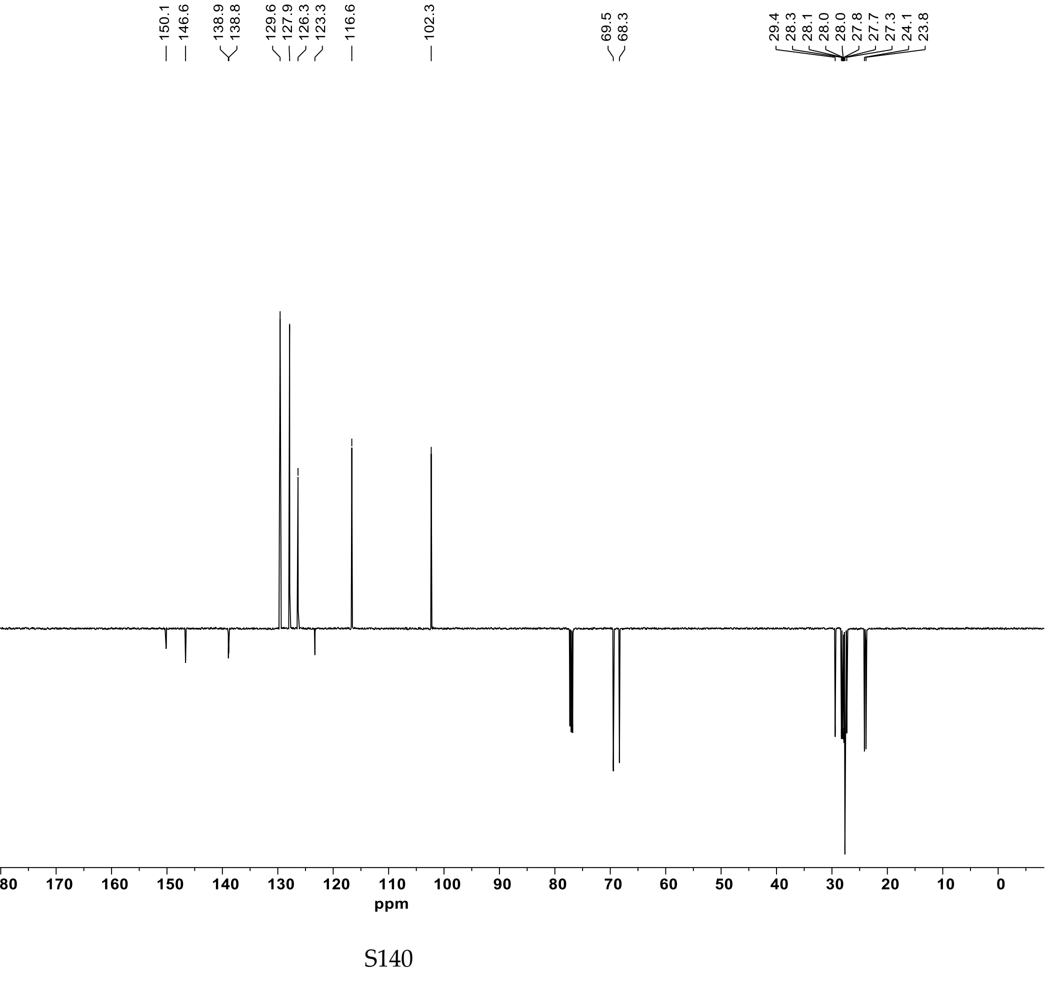

1[5]-bromo-2,17-dioxa-1(1,4)-benzenacyclohexadecaphan-1[2]-ol S61

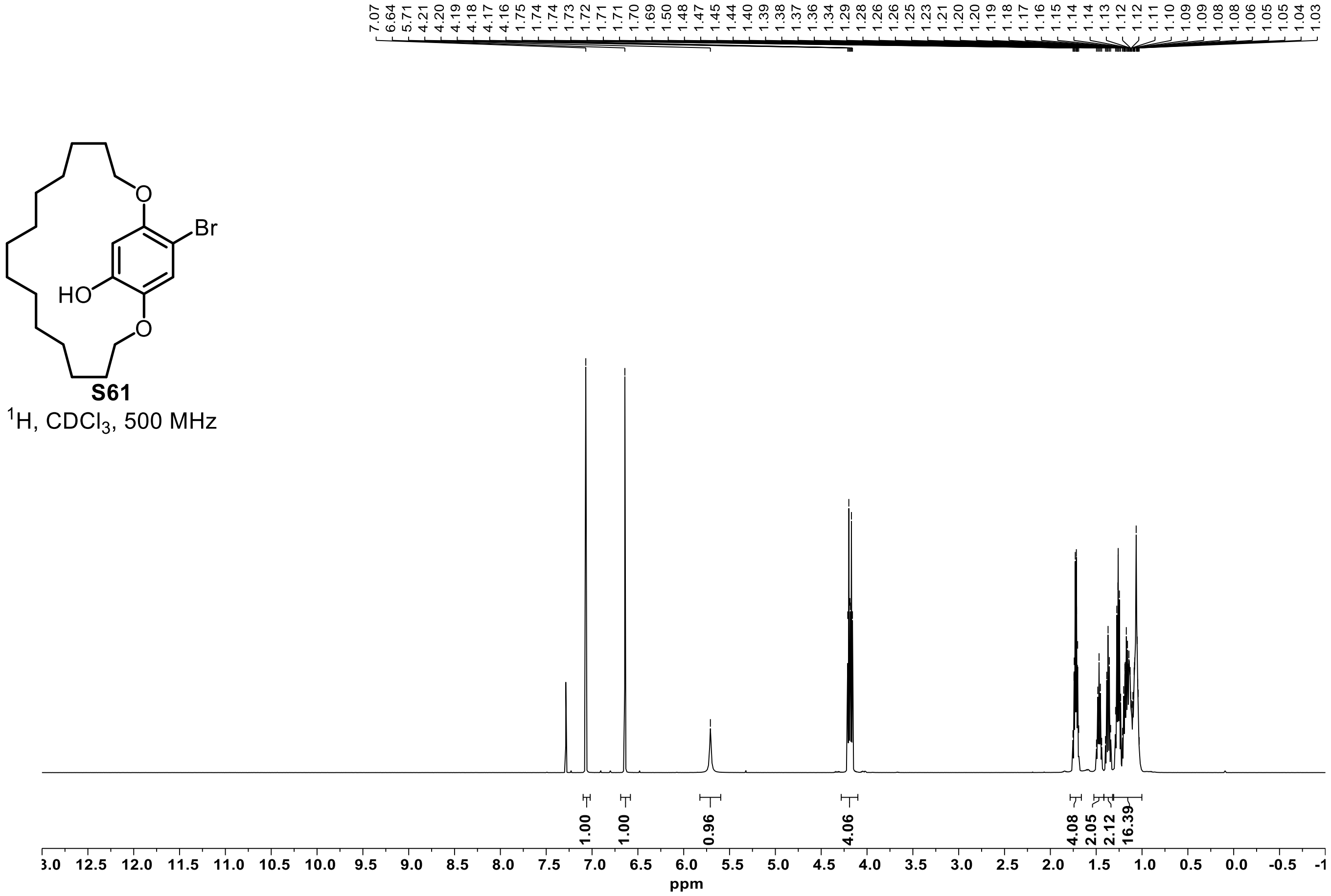

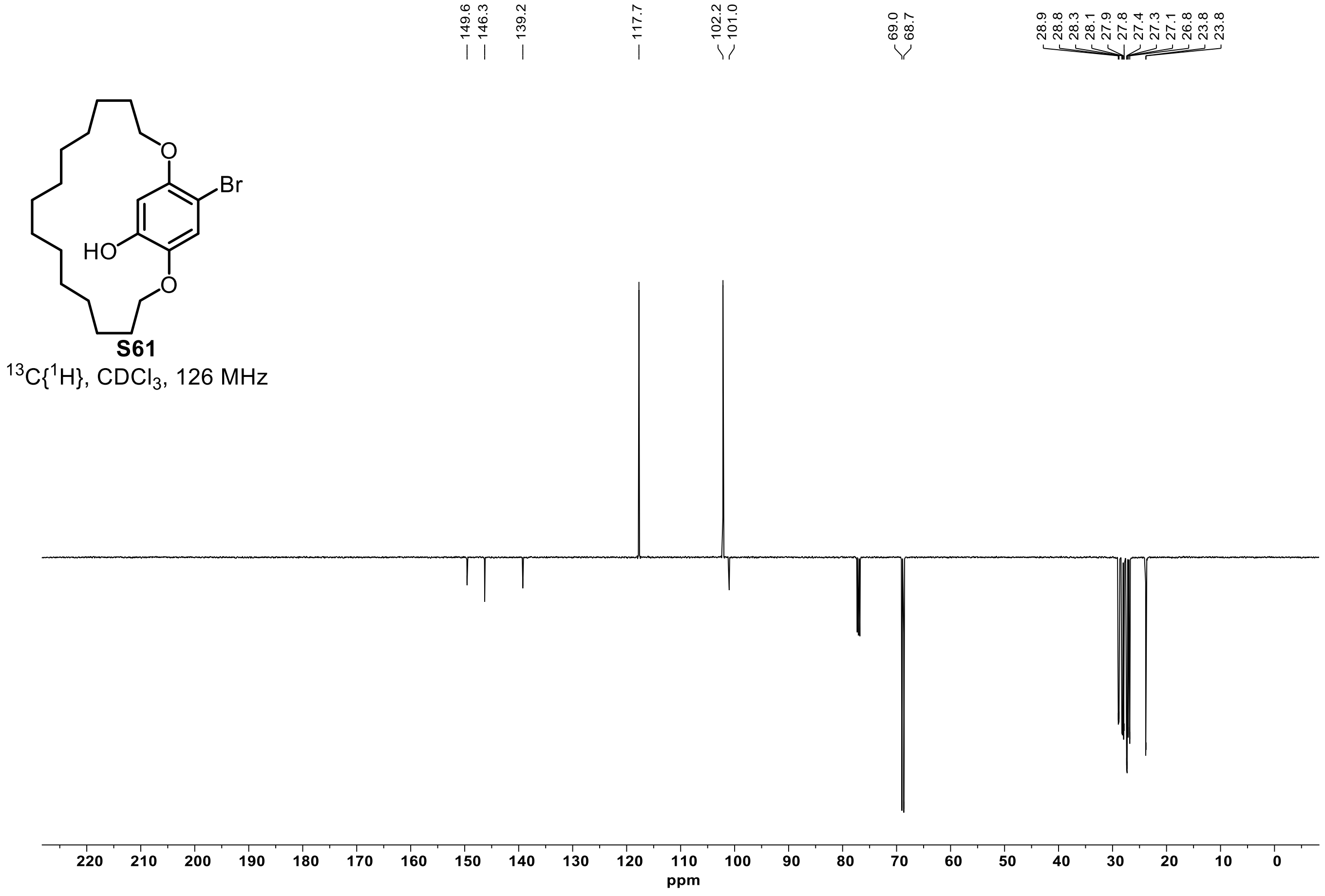

1[5]-phenyl-2,17-dioxa-1(1,4)-benzenacyclohexadecaphan-1[2]-ol S62

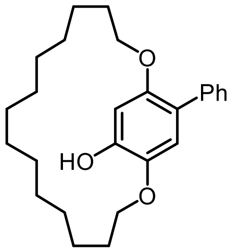

S62

^{1}H, $CDCl_3$, 500 MHz

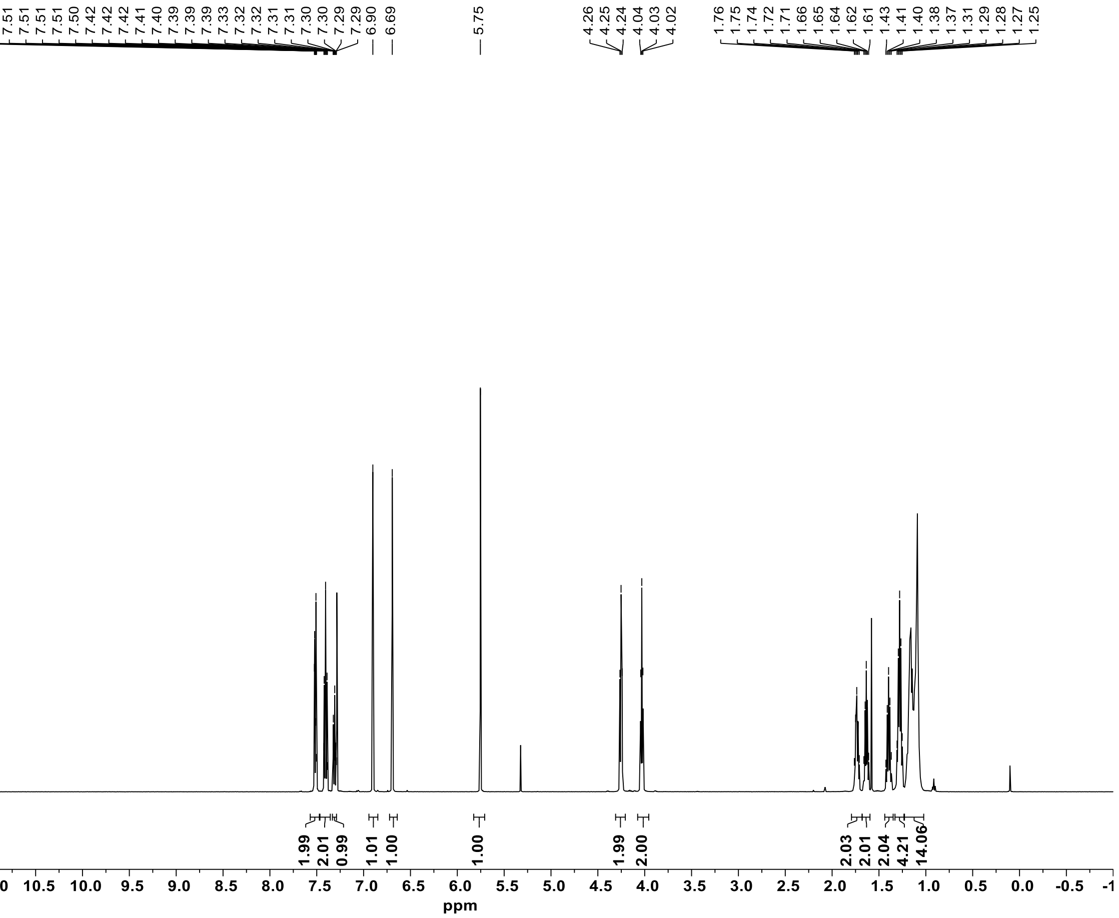

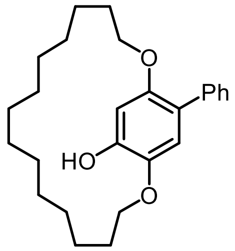

S62

$^{13}C\{^{1}H\}$, $CDCl_3$, 126 MHz

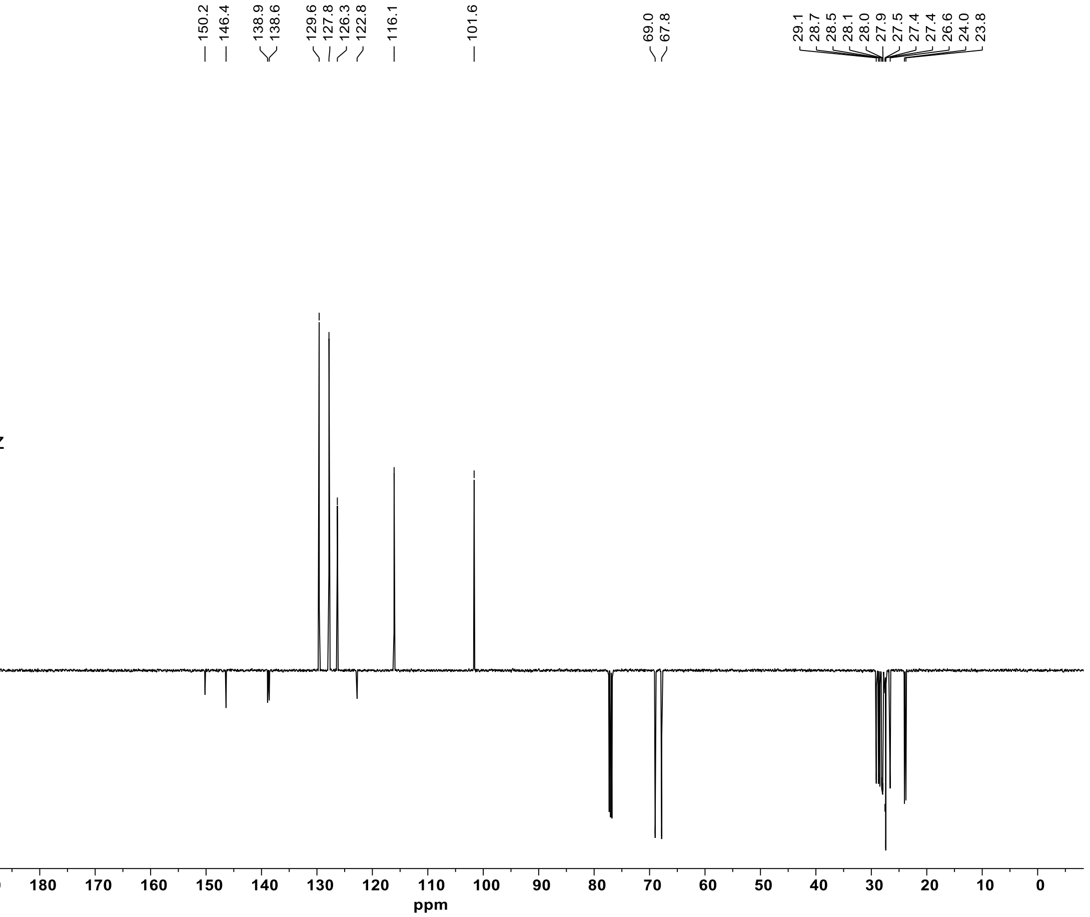

1²-bromo-1⁵-hydroxy-2,17-dioxa-5,14-diaza-1(1,4)-benzenacycloheptadecaphane-4,15-dione S63

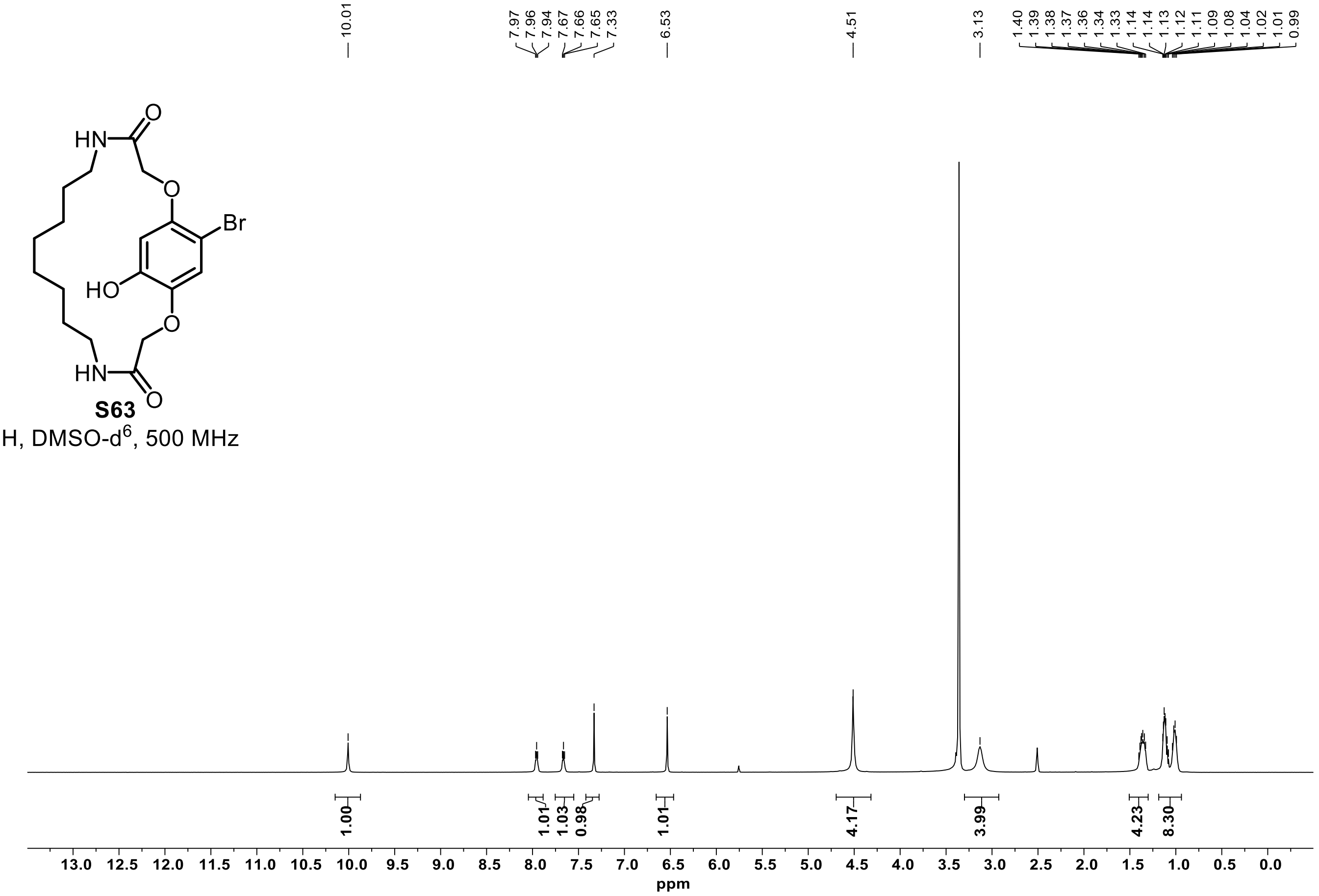

S63
^{1}H, DMSO-d^6, 500 MHz

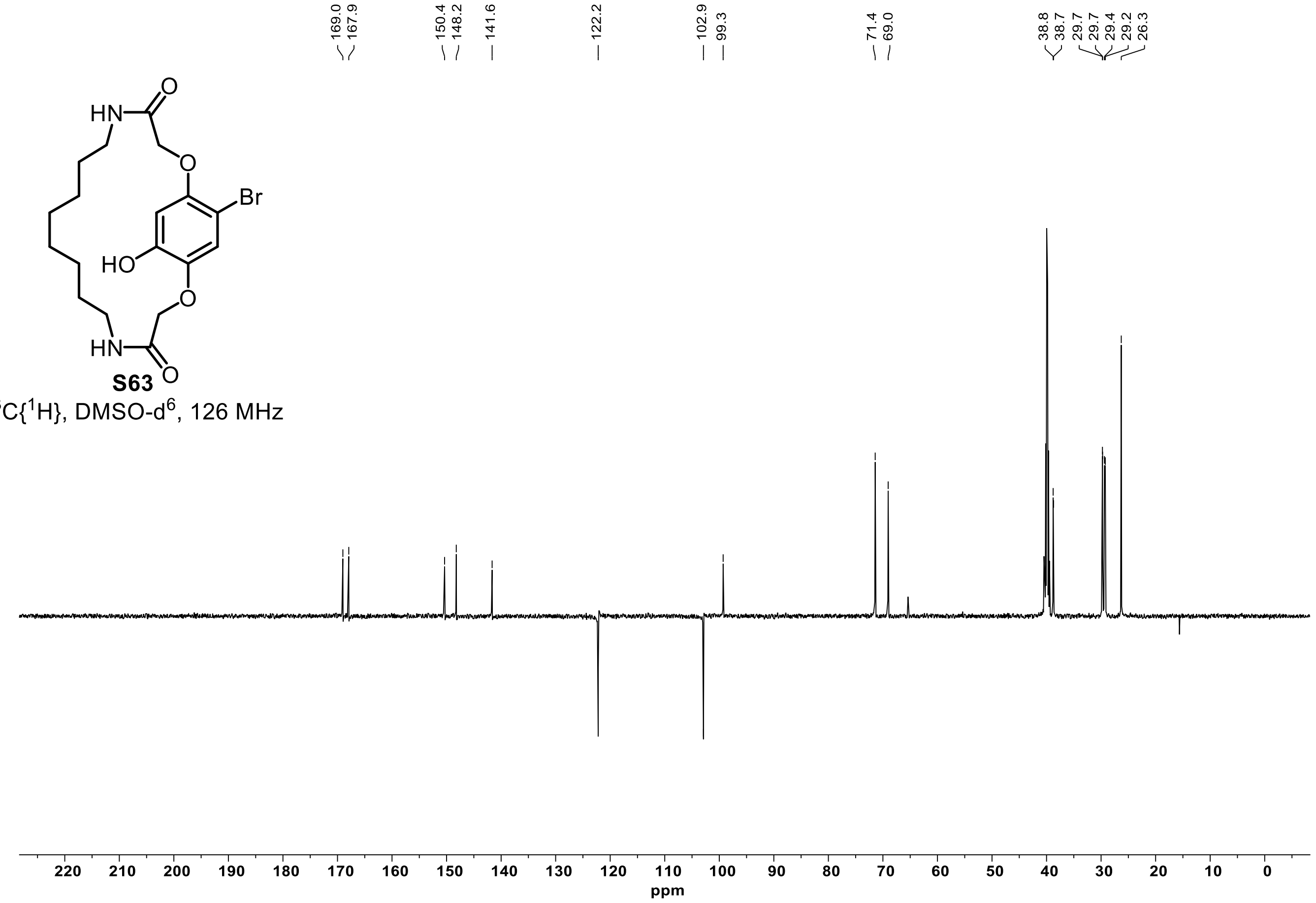

S63
^{13}C{^{1}H}, DMSO-d^6, 126 MHz

1²-hydroxy-1⁵-phenyl-2,17-dioxa-5,14-diaza-1(1,4)-benzenacycloheptadecaphane-4,15-dione S64

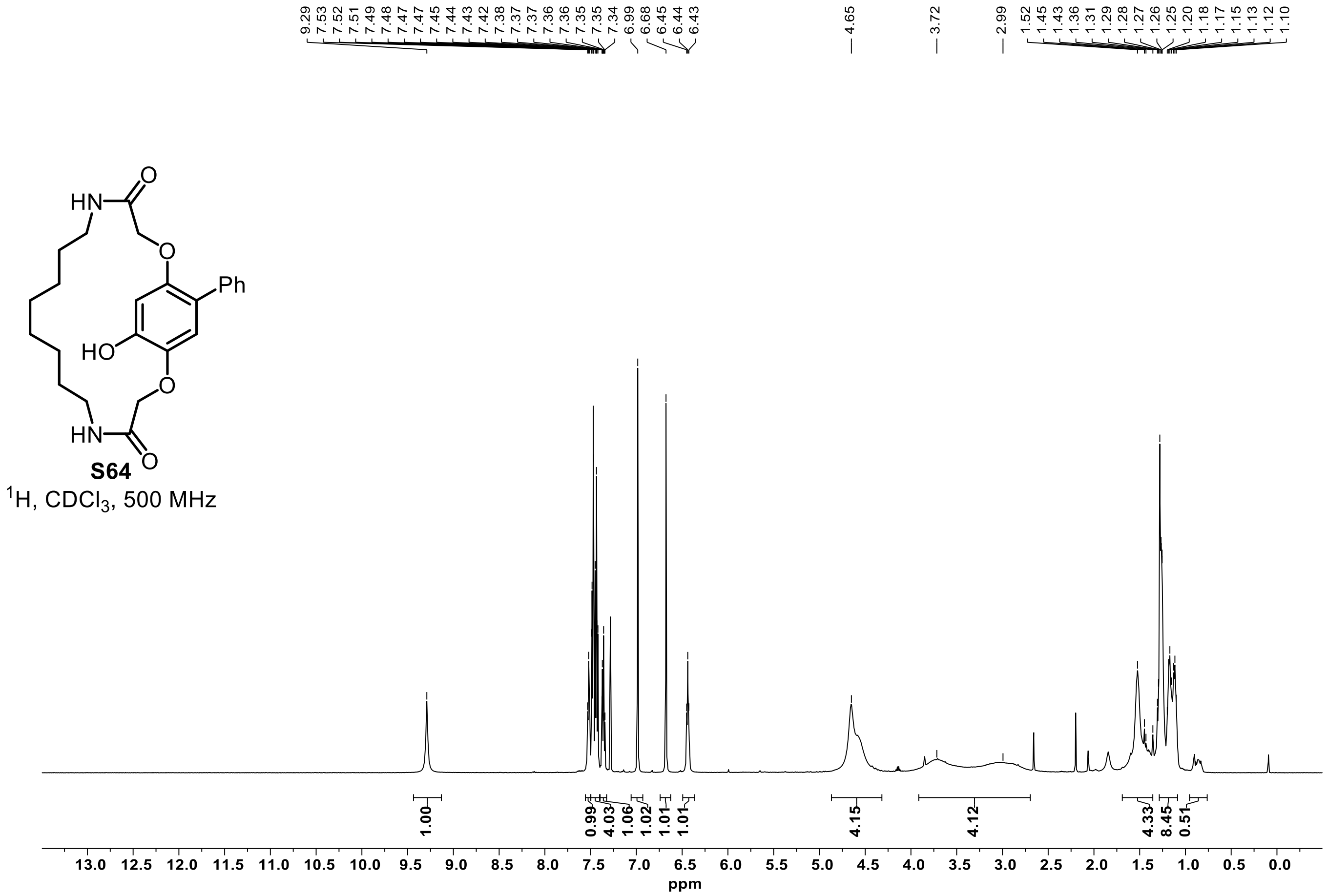

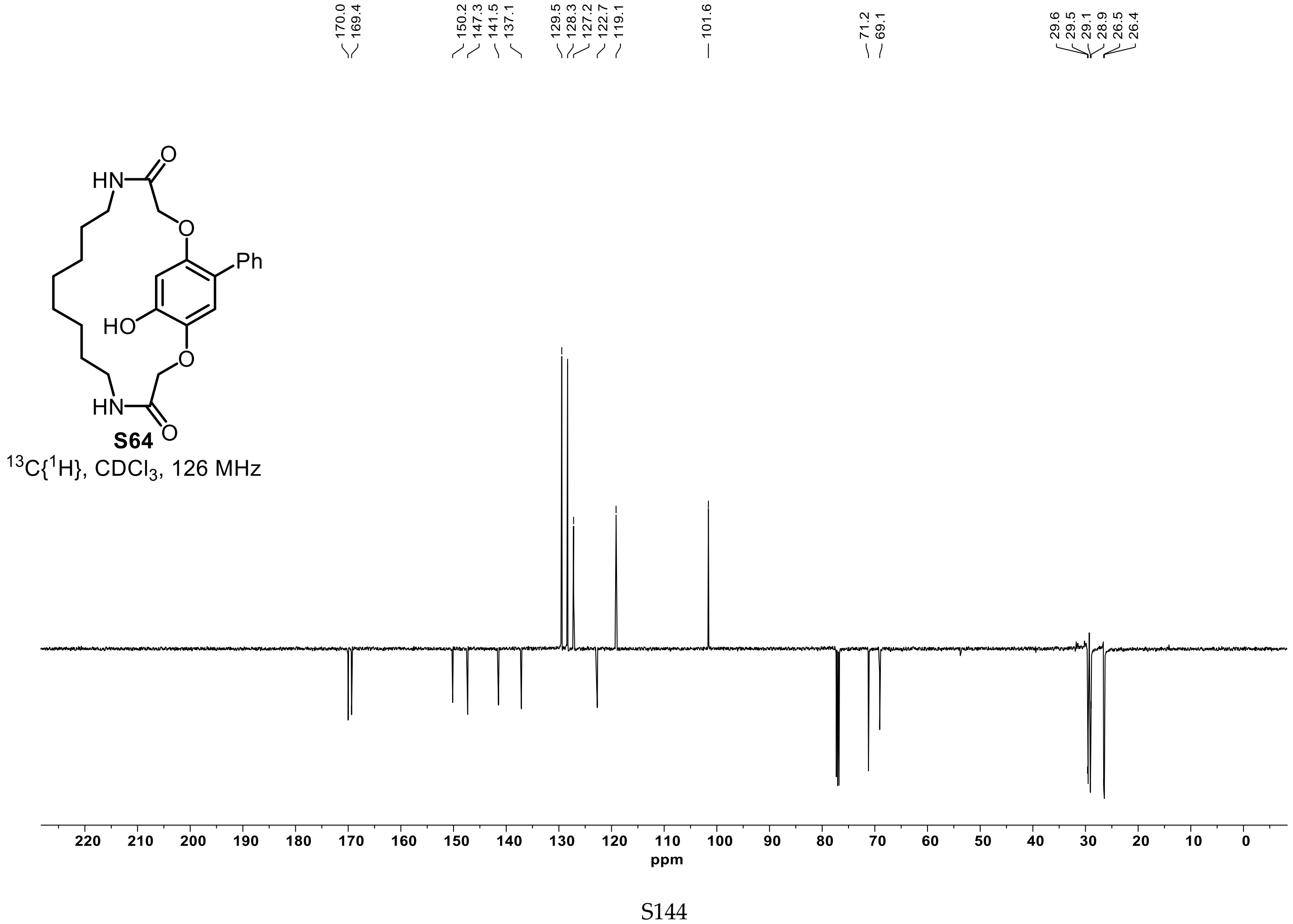

1²-hydroxy-1⁵-phenyl-2,8,10,16-tetraoxa-5,13-diaza-1(1,2),9(1,4)-dibenzenacyclo-hexadecaphane-4,14-dione S65

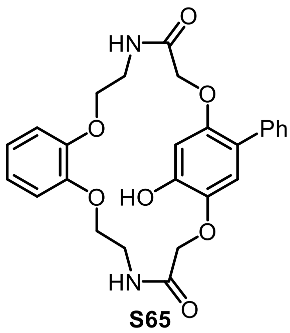

S65
^{1}H, DMSO-d^{6}, 500 MHz

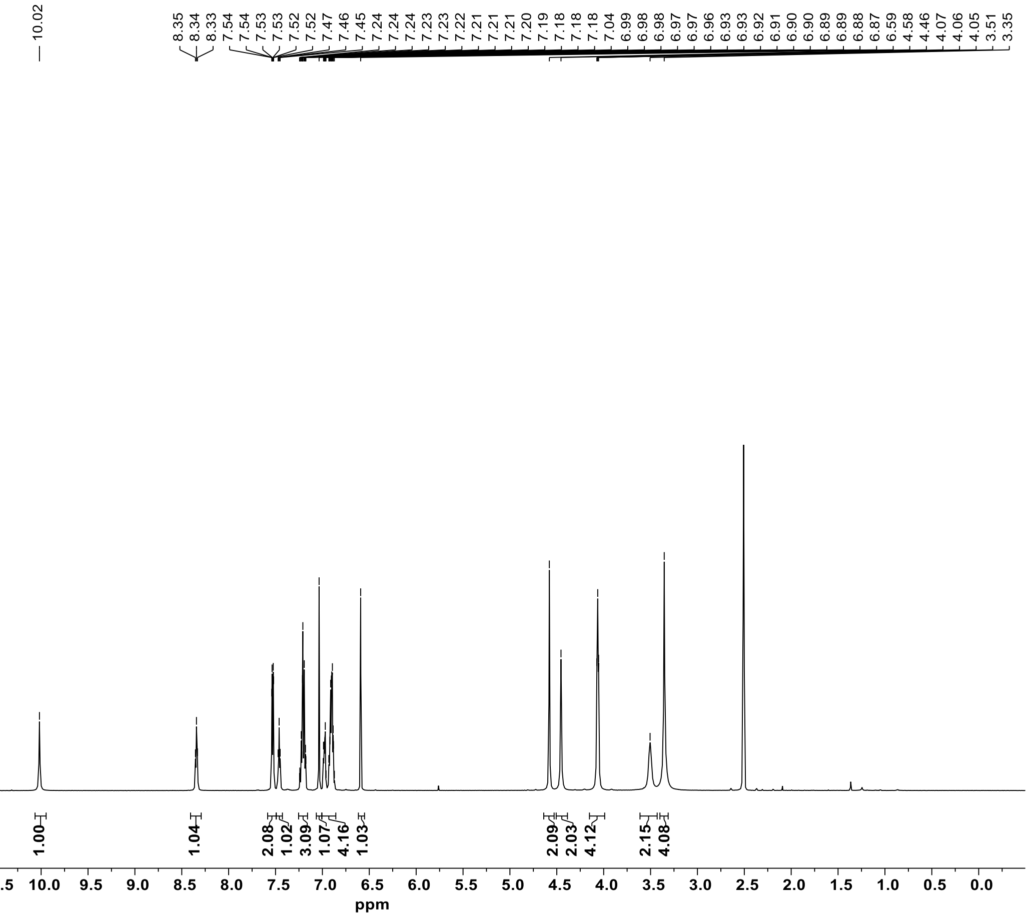

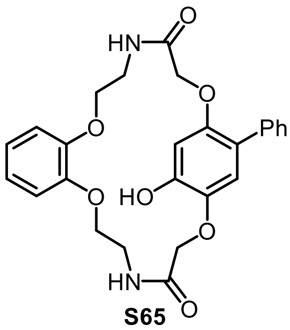

S65
^{13}C{^{1}H}, DMSO-d^{6}, 126 MHz

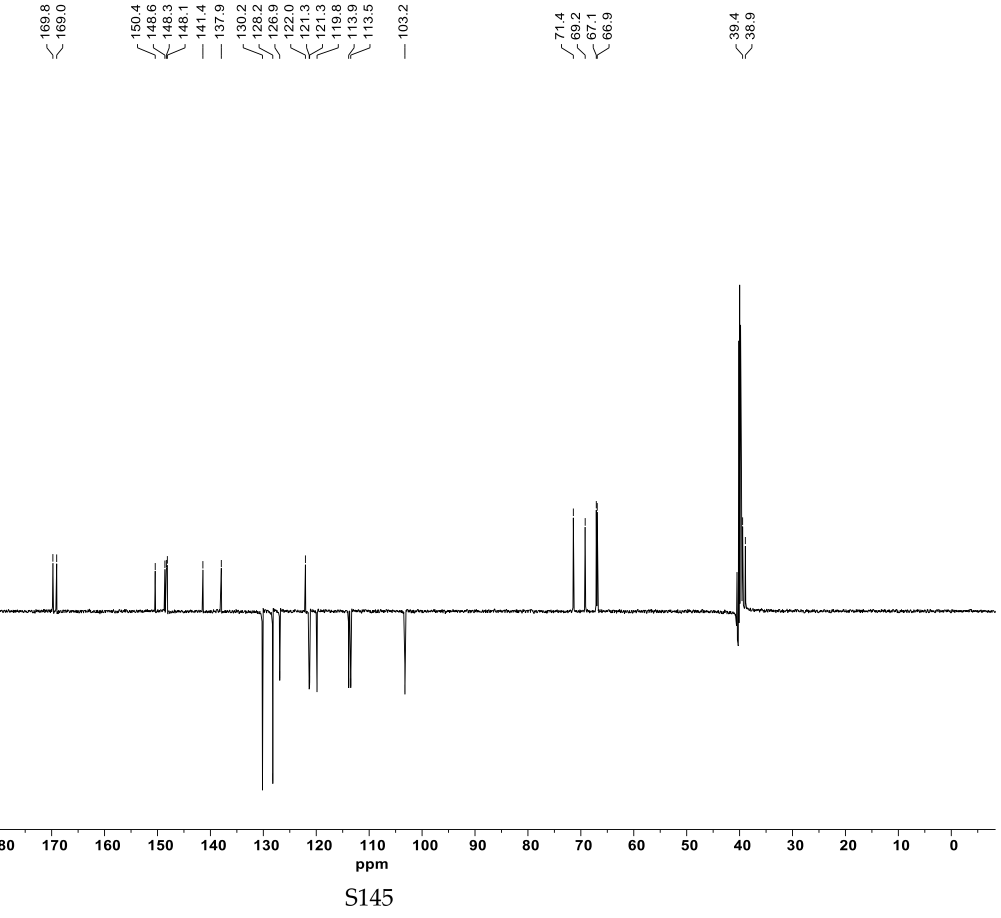

1⁵-phenyl-2,18-dioxa-1(1,4)-benzenacyclohexadecaphan-1²-ol S66

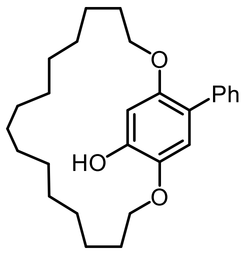

S66
^{1}H, $CDCl_3$, 500 MHz

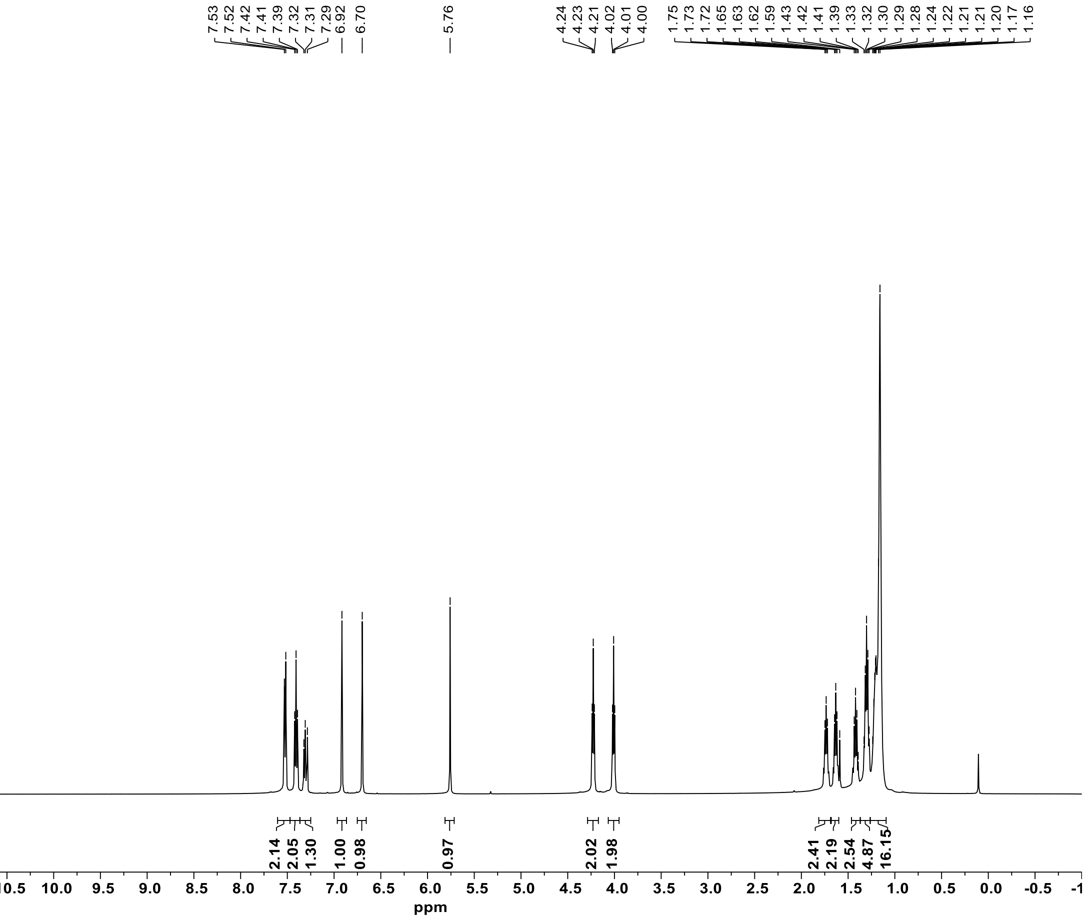

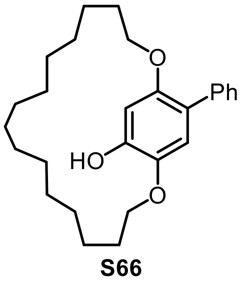

S66
$^{13}C\{^{1}H\}$, $CDCl_3$, 126 MHz

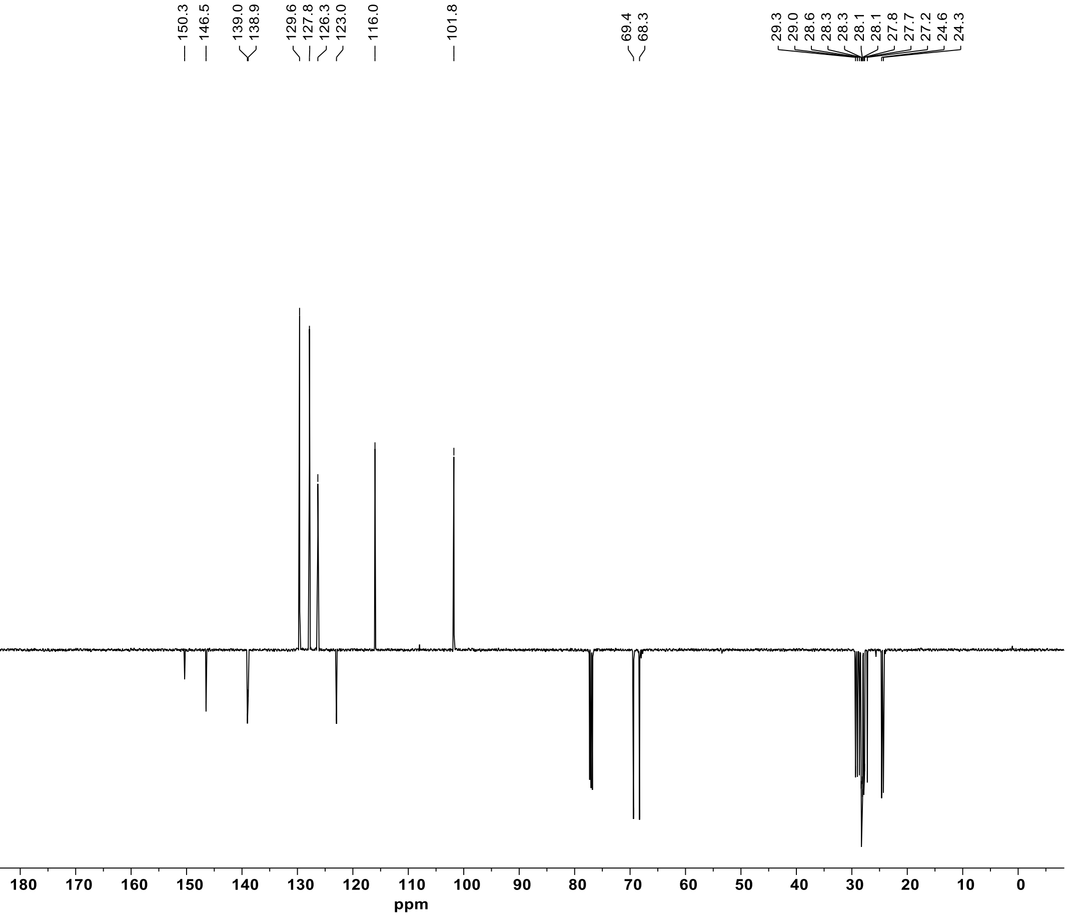

1⁵-phenyl-2,19-dioxa-1(1,4)-benzenacyclohexadecaphan-1²-ol S67

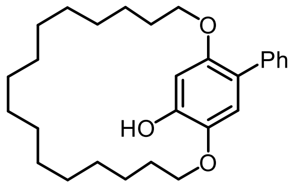

S67

^{1}H, $CDCl_3$, 500 MHz

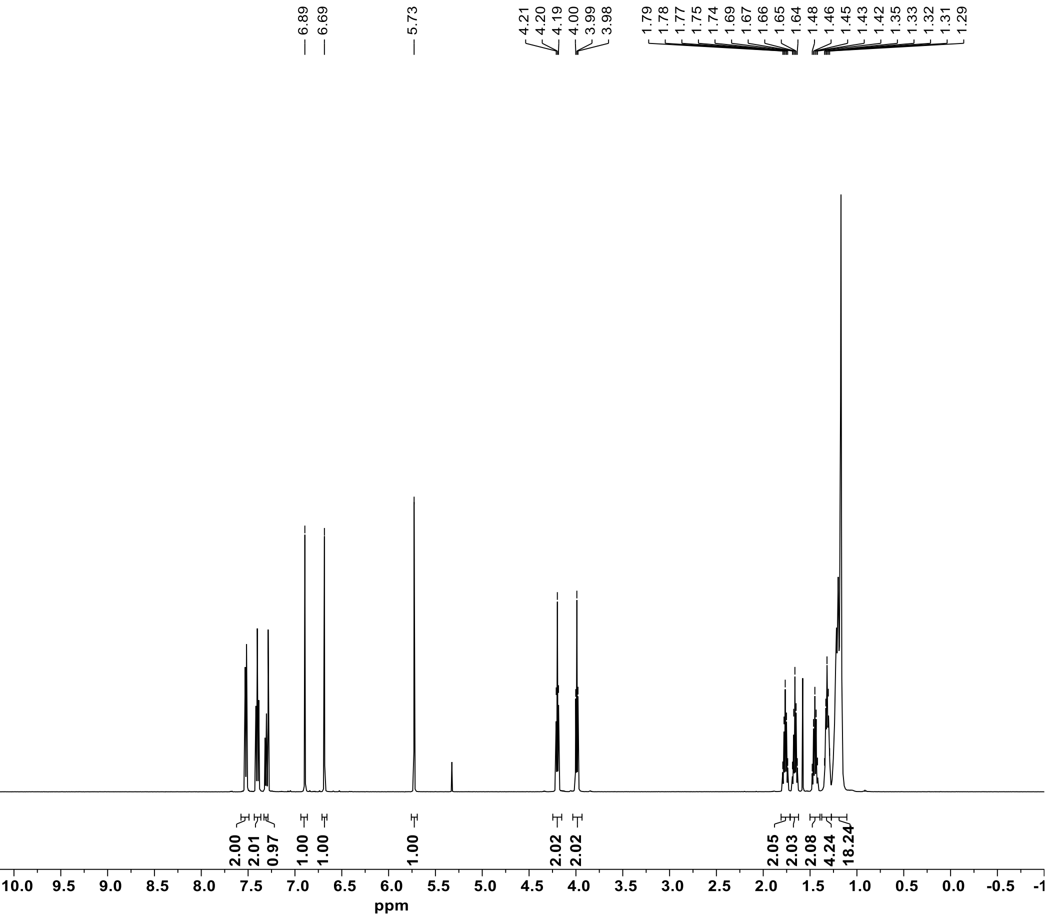

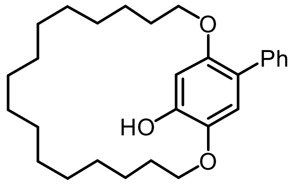

S67

$^{13}C\{^{1}H\}$, $CDCl_3$, 126 MHz

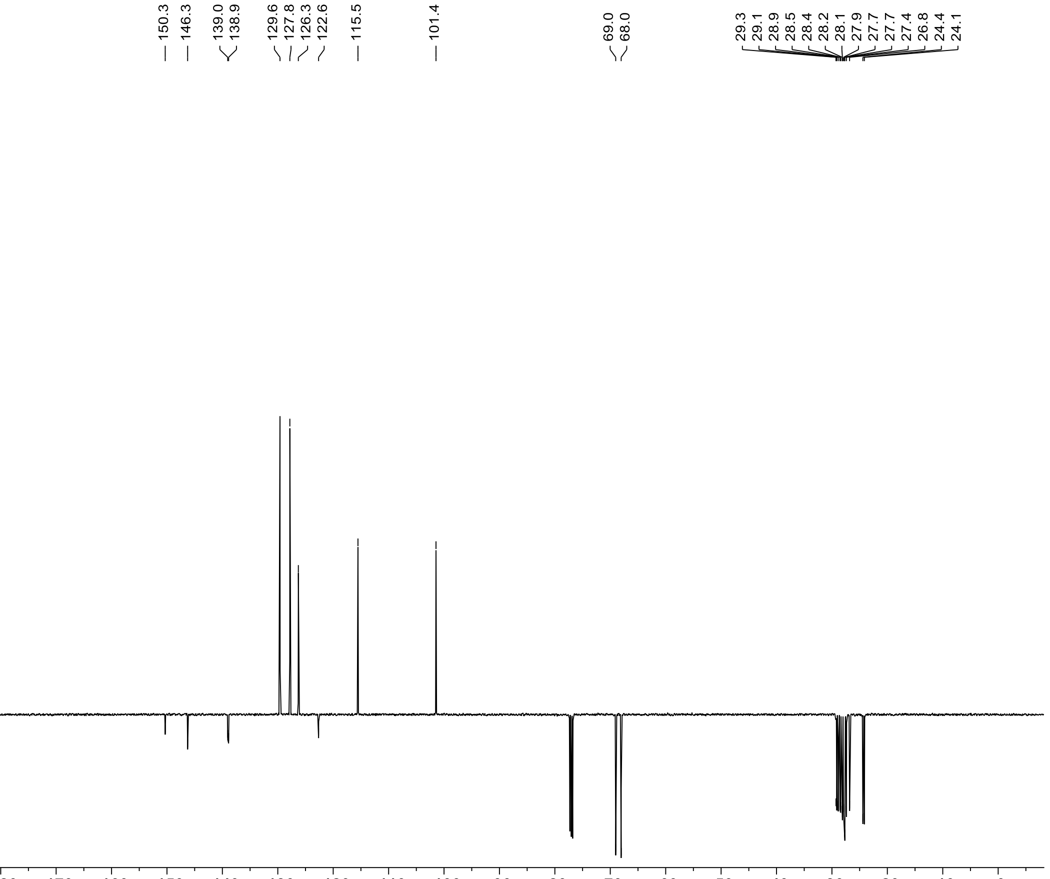

Synthesis of 1⁵-butyl-2,15-dioxa-1(1,4)-benzenacyclotridecaphane-1²-carbaldehyde S68

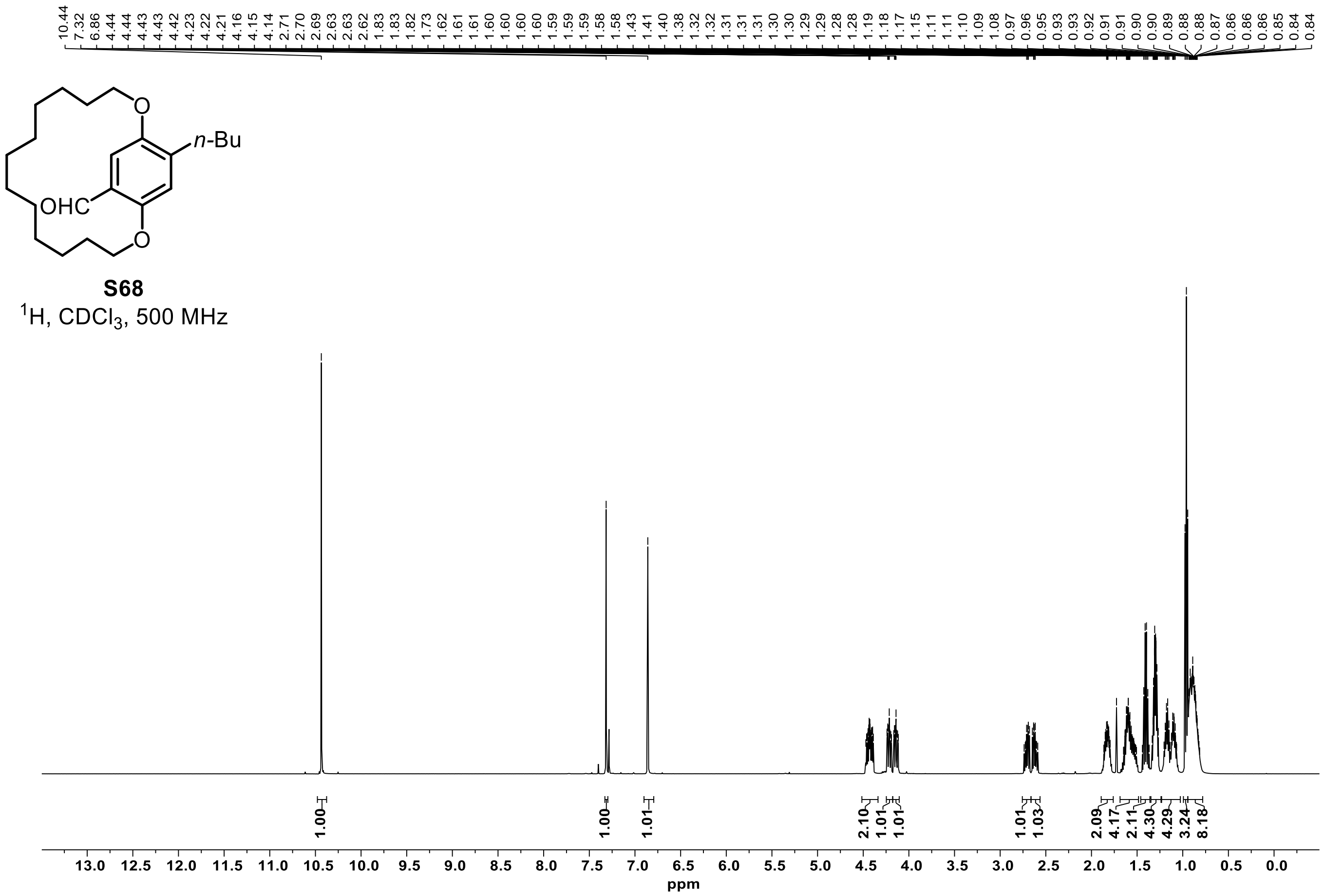

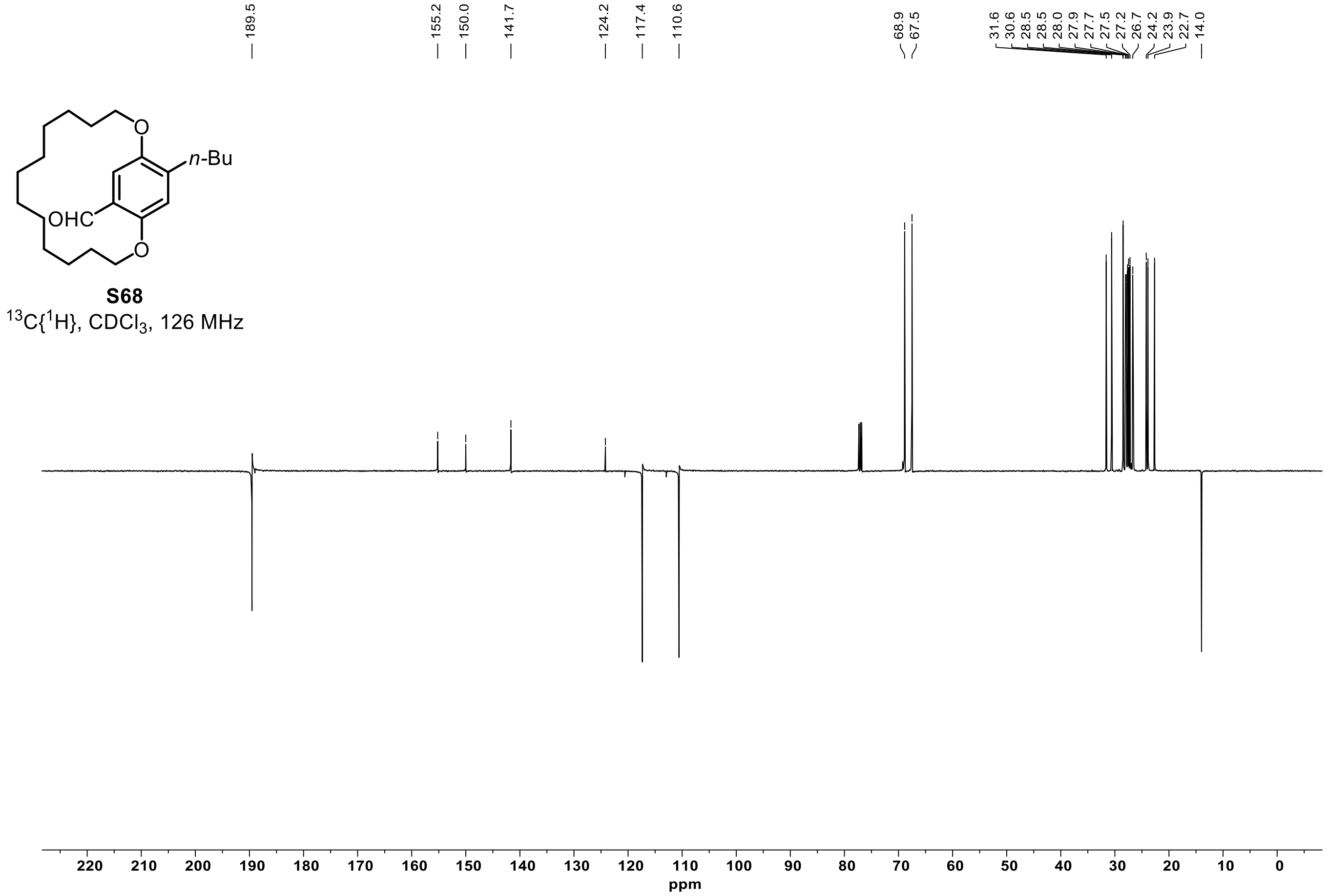

Synthesis of 1^5-butyl-2,15-dioxa-1(1,4)-benzenacyclopentadecaphan-1^2-ol S69

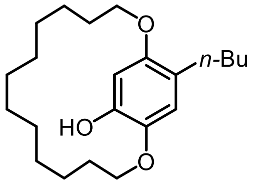

S69
^{1}H, $CDCl_3$, 500 MHz

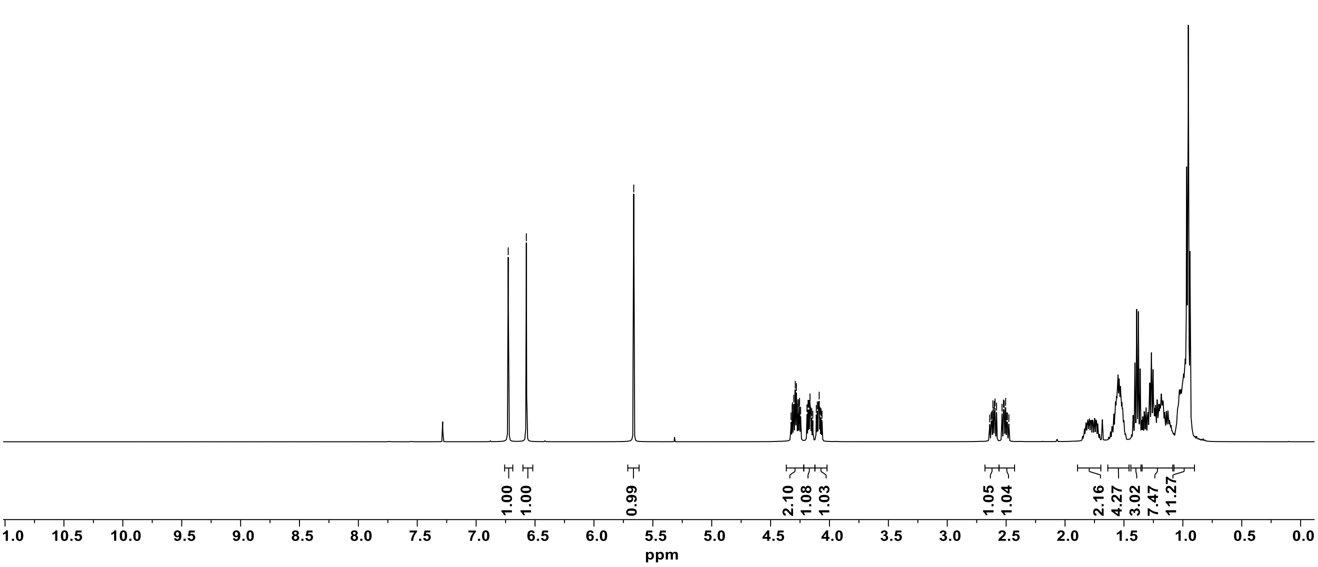

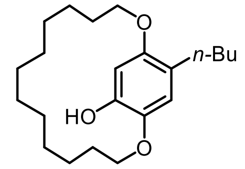

S69
$^{13}C\{^1H\}$, $CDCl_3$, 126 MHz

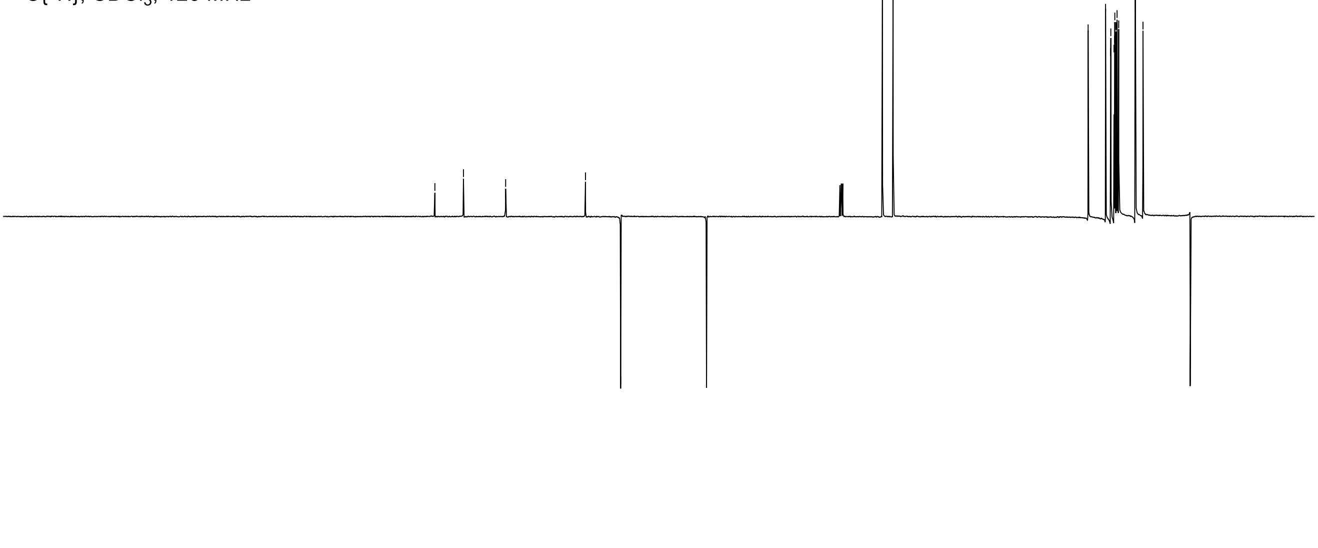

(*Rp*)-1⁵-bromo-2,13-dioxa-1(1,4)-benzenacyclotridecaphane-1²-yl isobutyrate 2

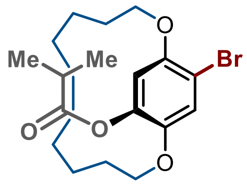

2
^{1}H, $CDCl_3$, 500 MHz

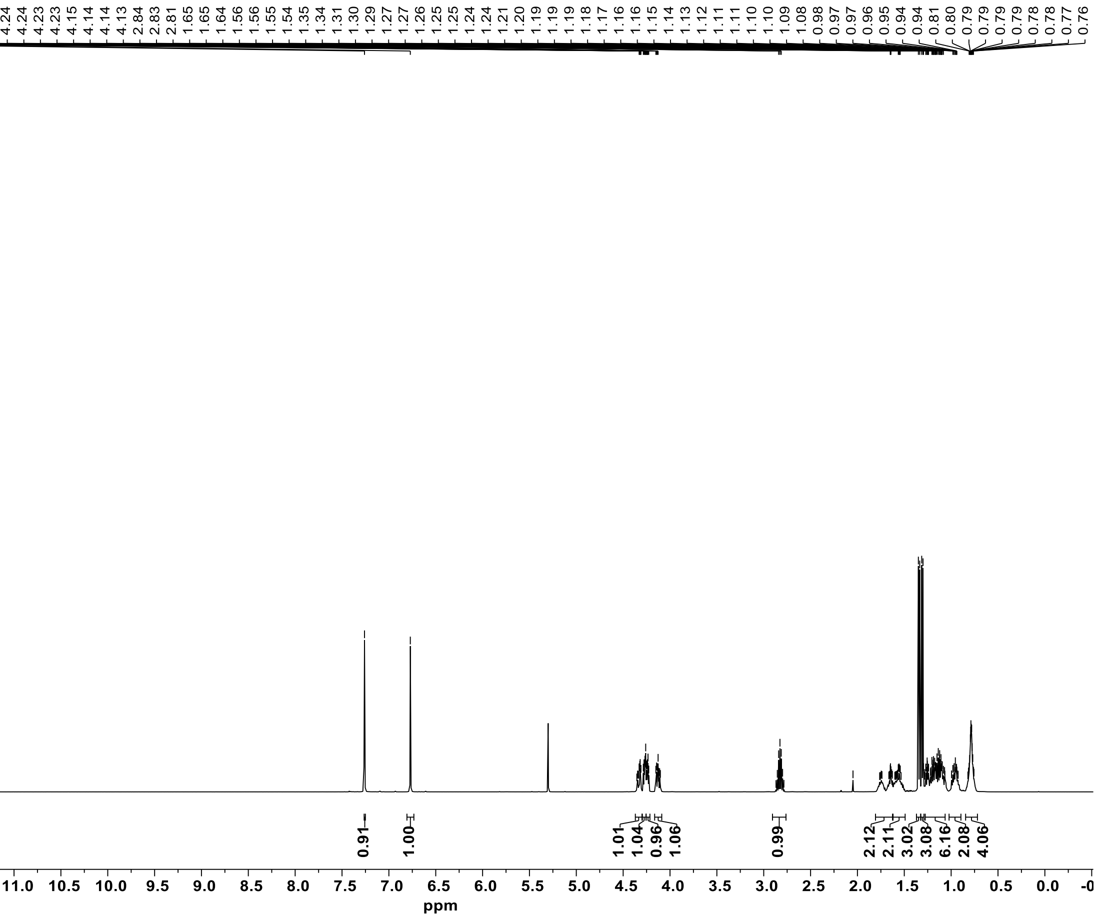

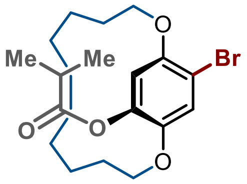

2
$^{13}C\{^{1}H\}$, $CDCl_3$, 126 MHz

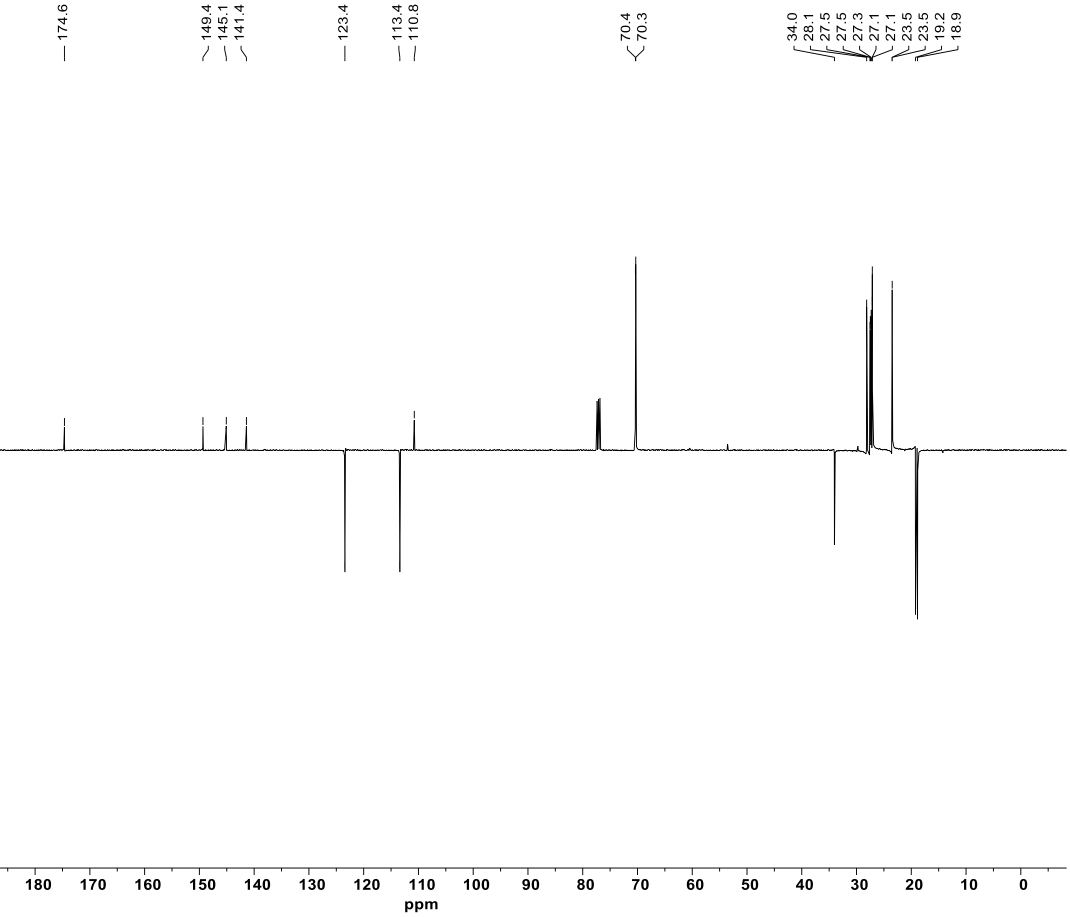

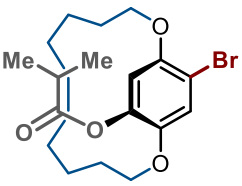

2

2D ^{1}H, $CDCl_3$, COSY

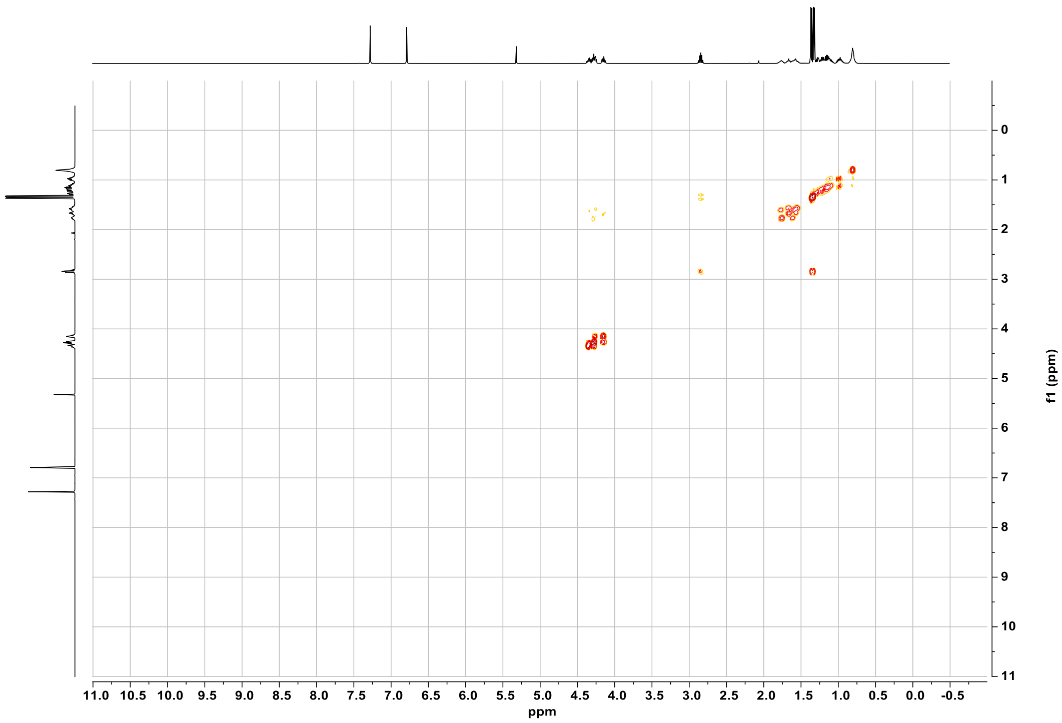

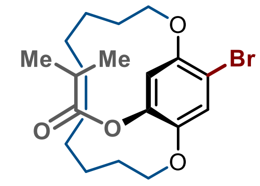

2

2D ^{1}H-^{13}C, $CDCl_3$, HSQC

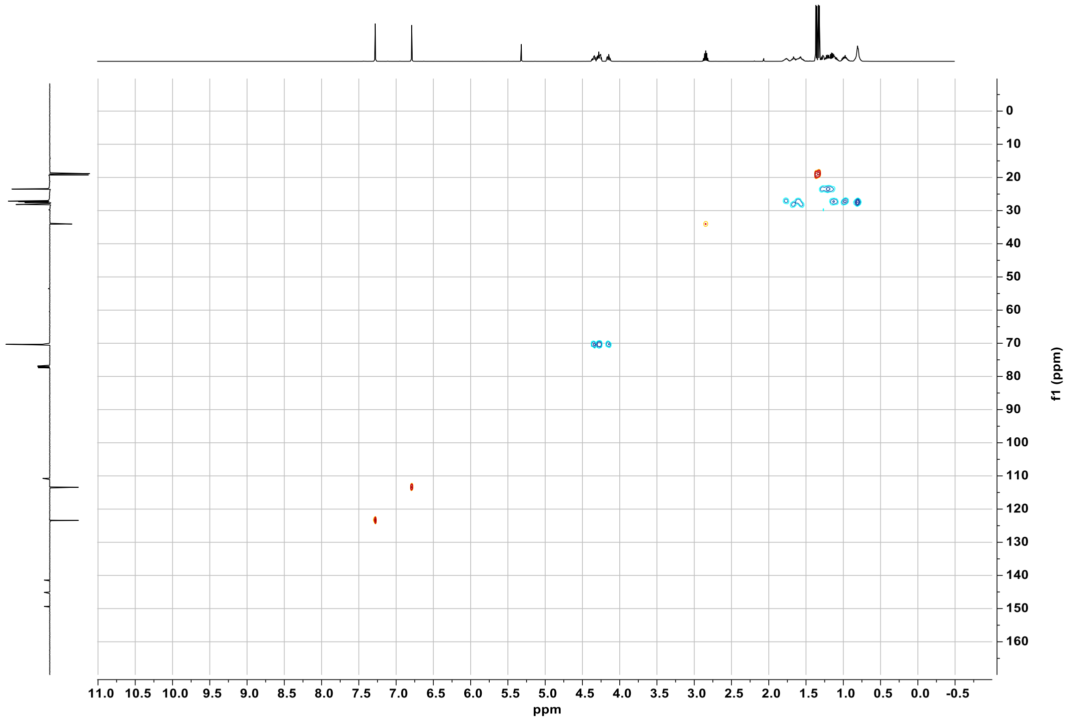

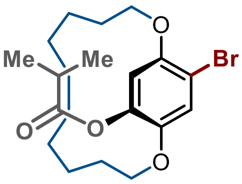

2

2D ^{1}H-^{13}C, $CDCl_3$, HMBC

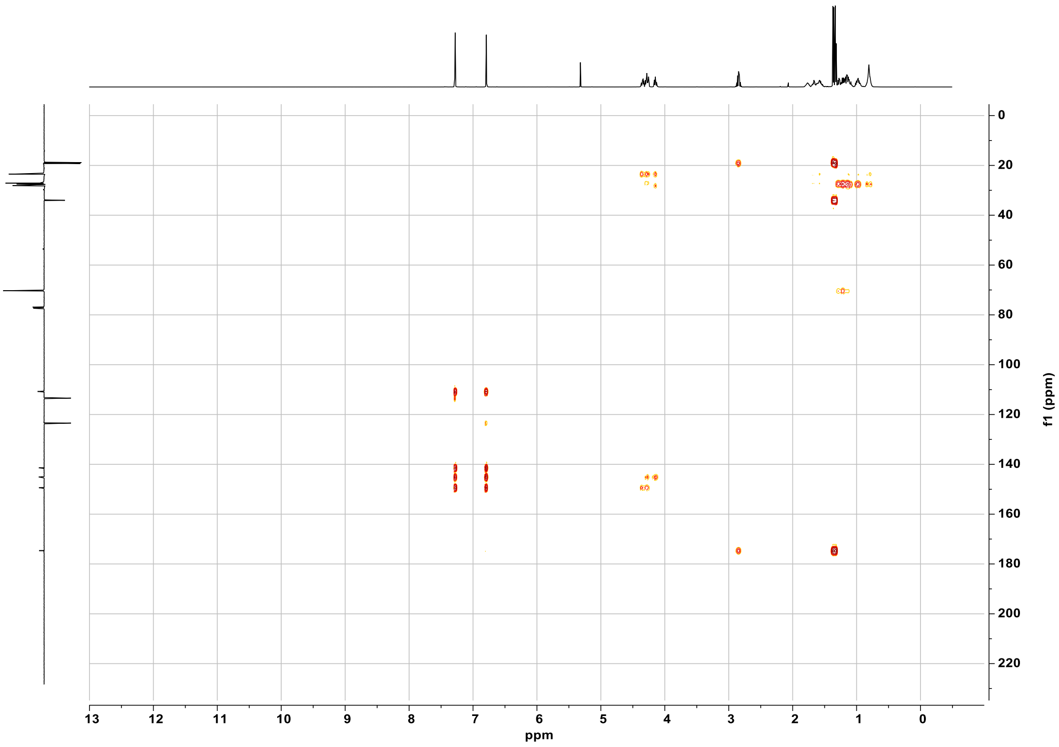

(*Rp*)-1⁵-phenyl-2,13-dioxa-1(1,4)-benzenacyclotridecaphane-1²-yl isobutyrate 5

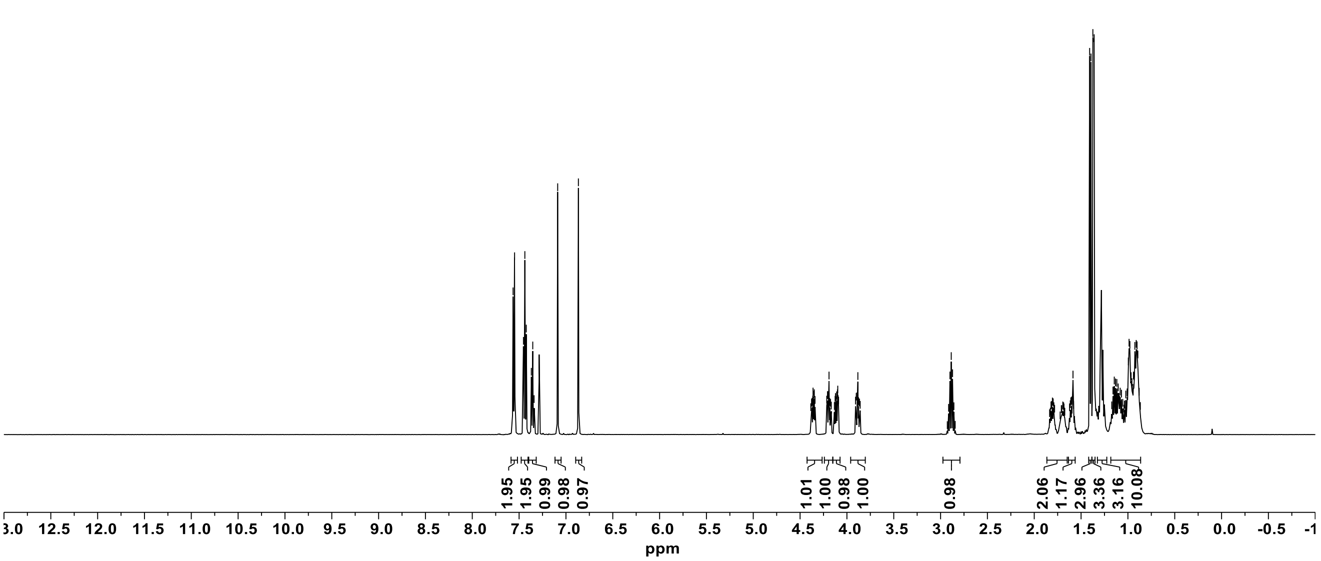

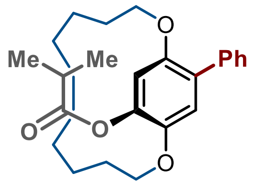

5

^{1}H, $CDCl_3$, 500 MHz

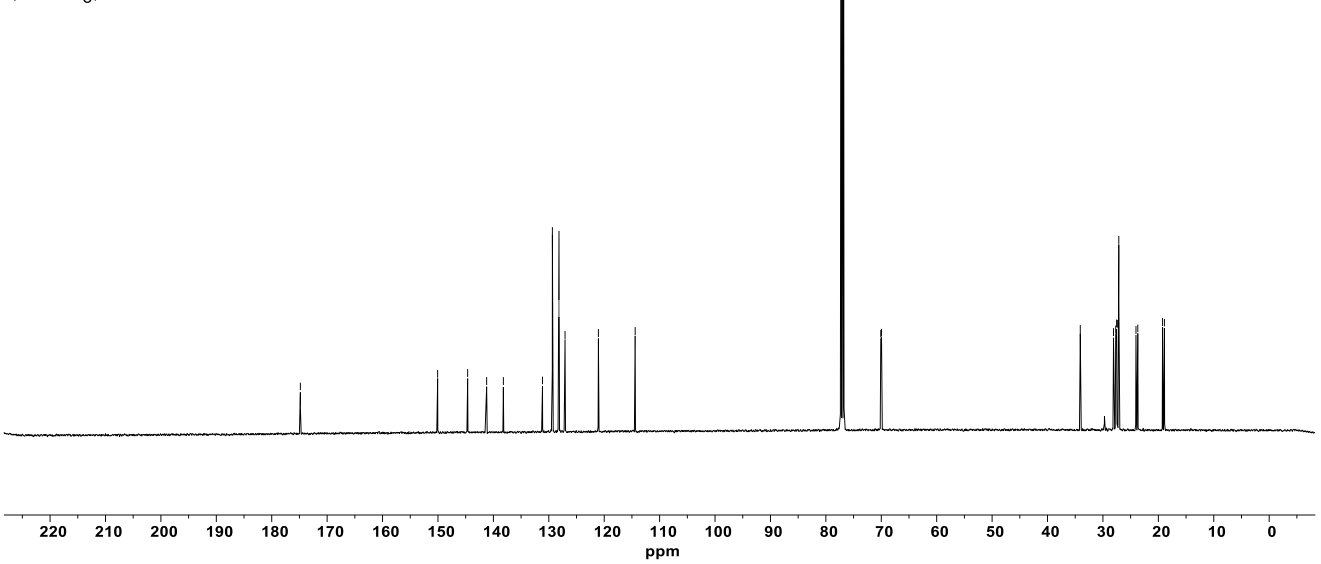

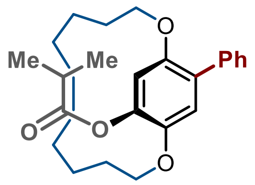

5

^{13}C, $CDCl_3$, 126 MHz

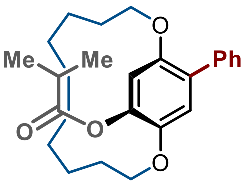

5

2D ^{1}H, $CDCl_3$, COSY

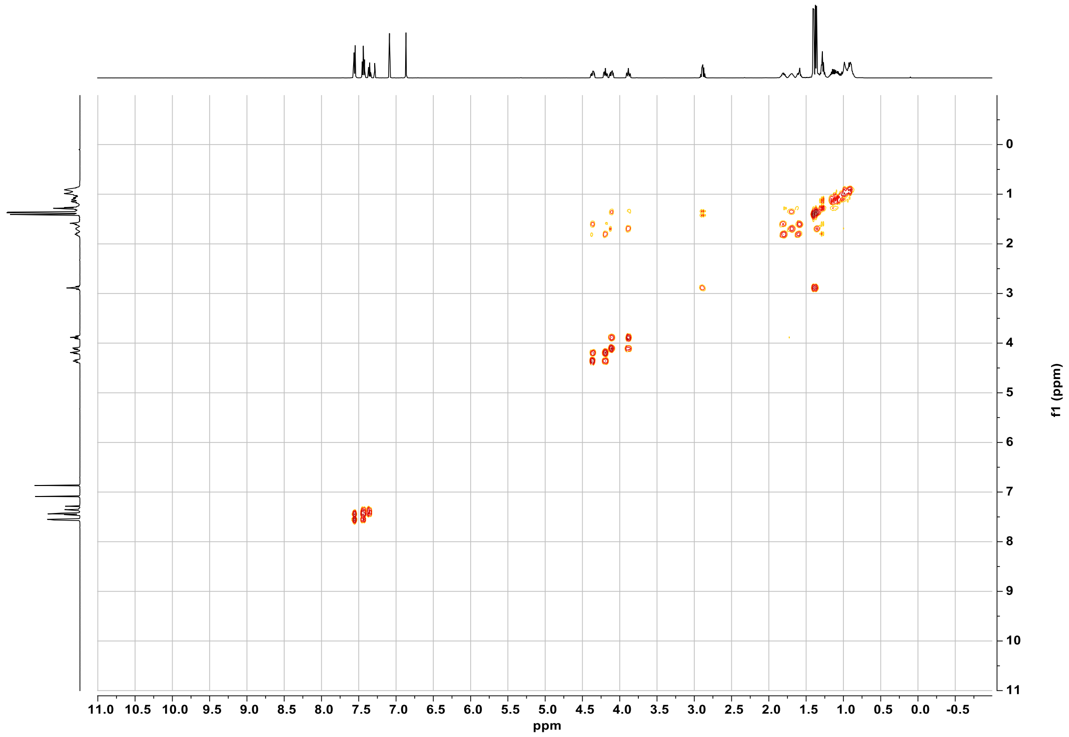

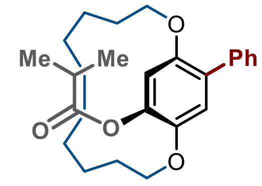

5

2D ^{1}H-^{13}C, $CDCl_3$, HSQC

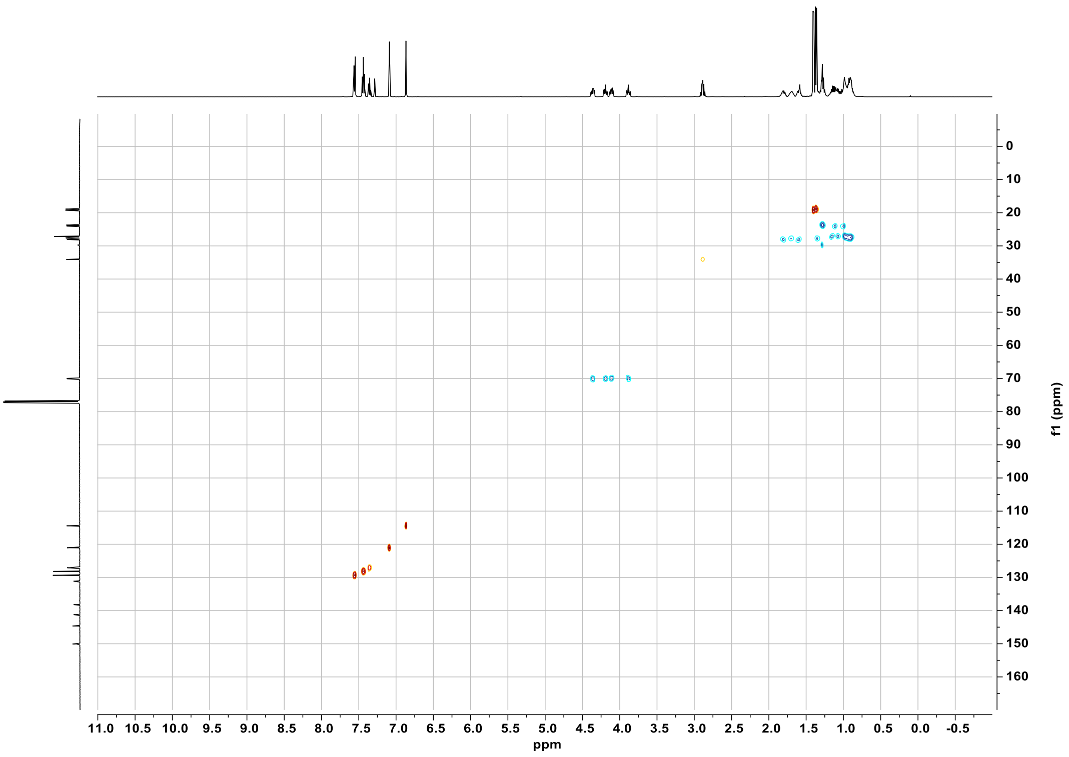

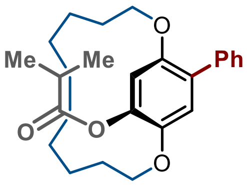

5

2D ^{1}H-^{13}C, $CDCl_3$, HMBC

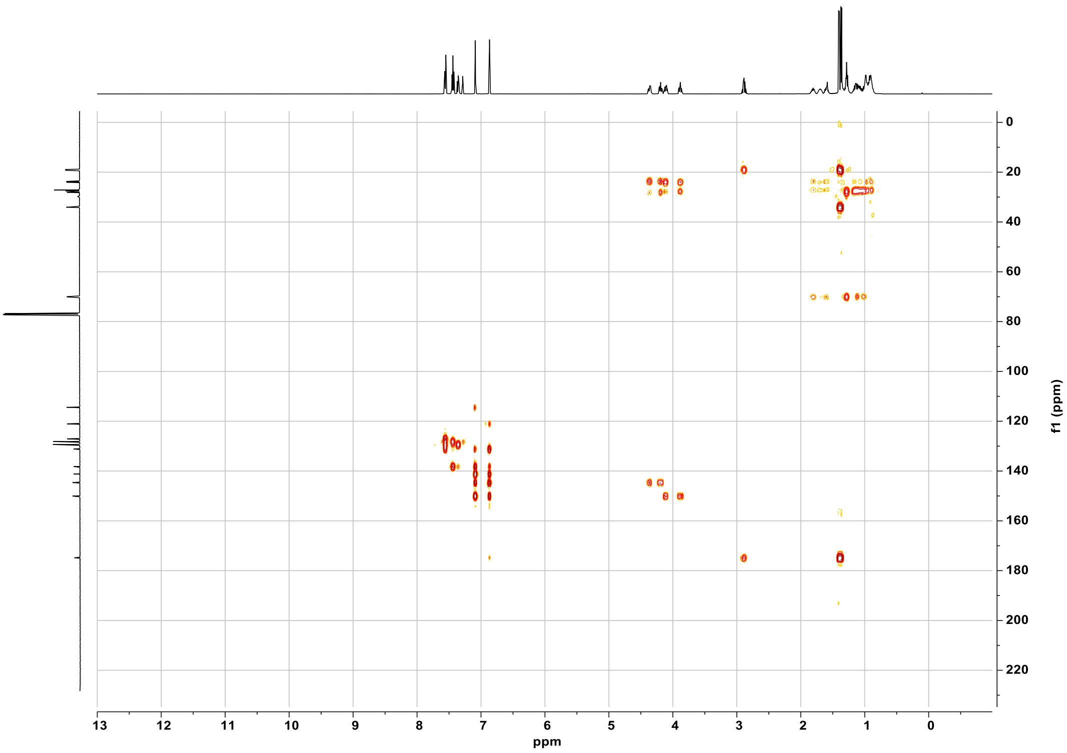

(*Rp*)-1⁵-(pyridin-3-yl)-2,13-dioxa-1(1,4)-benzenacyclotridecaphane-1²-yl isobutyrate 6

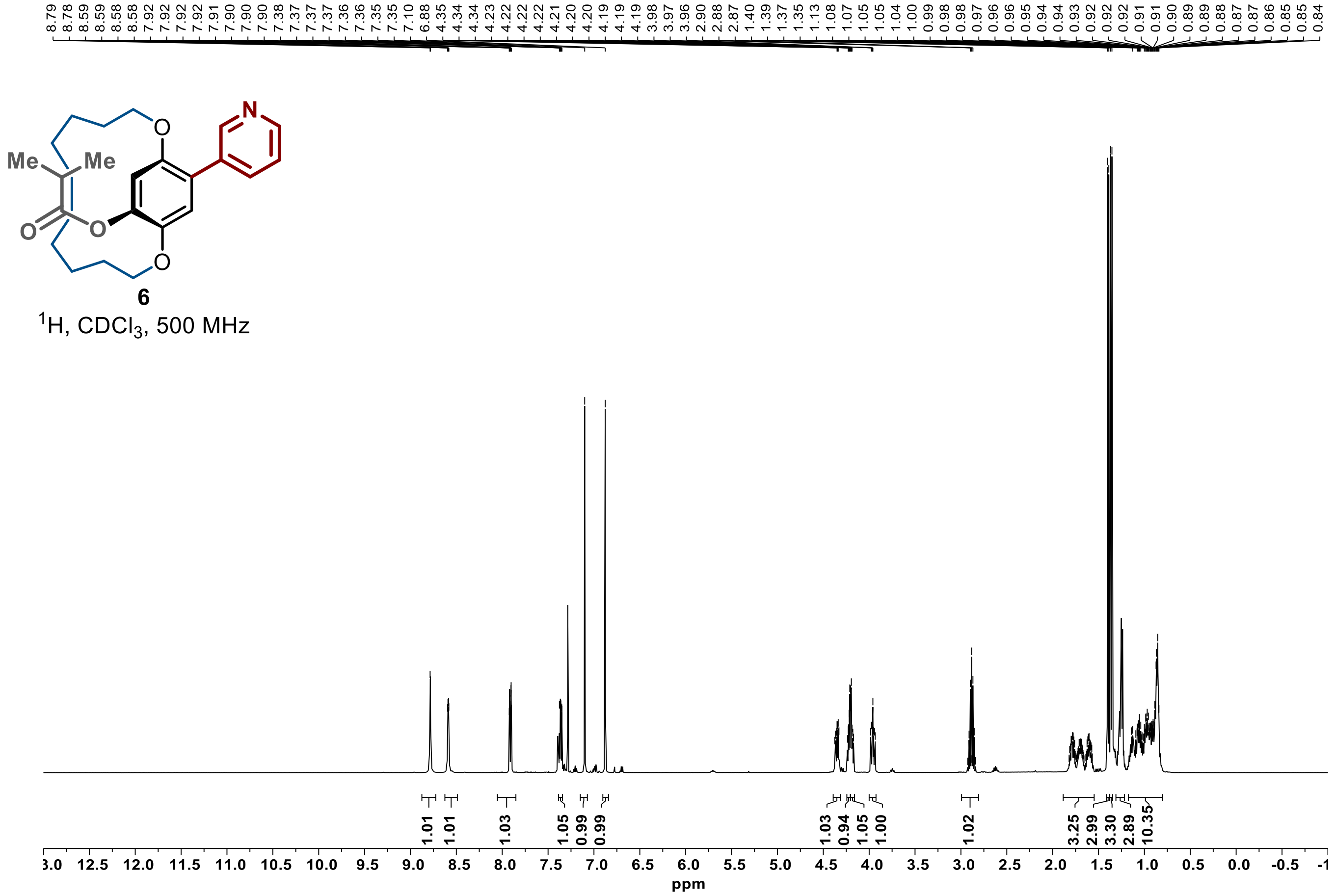

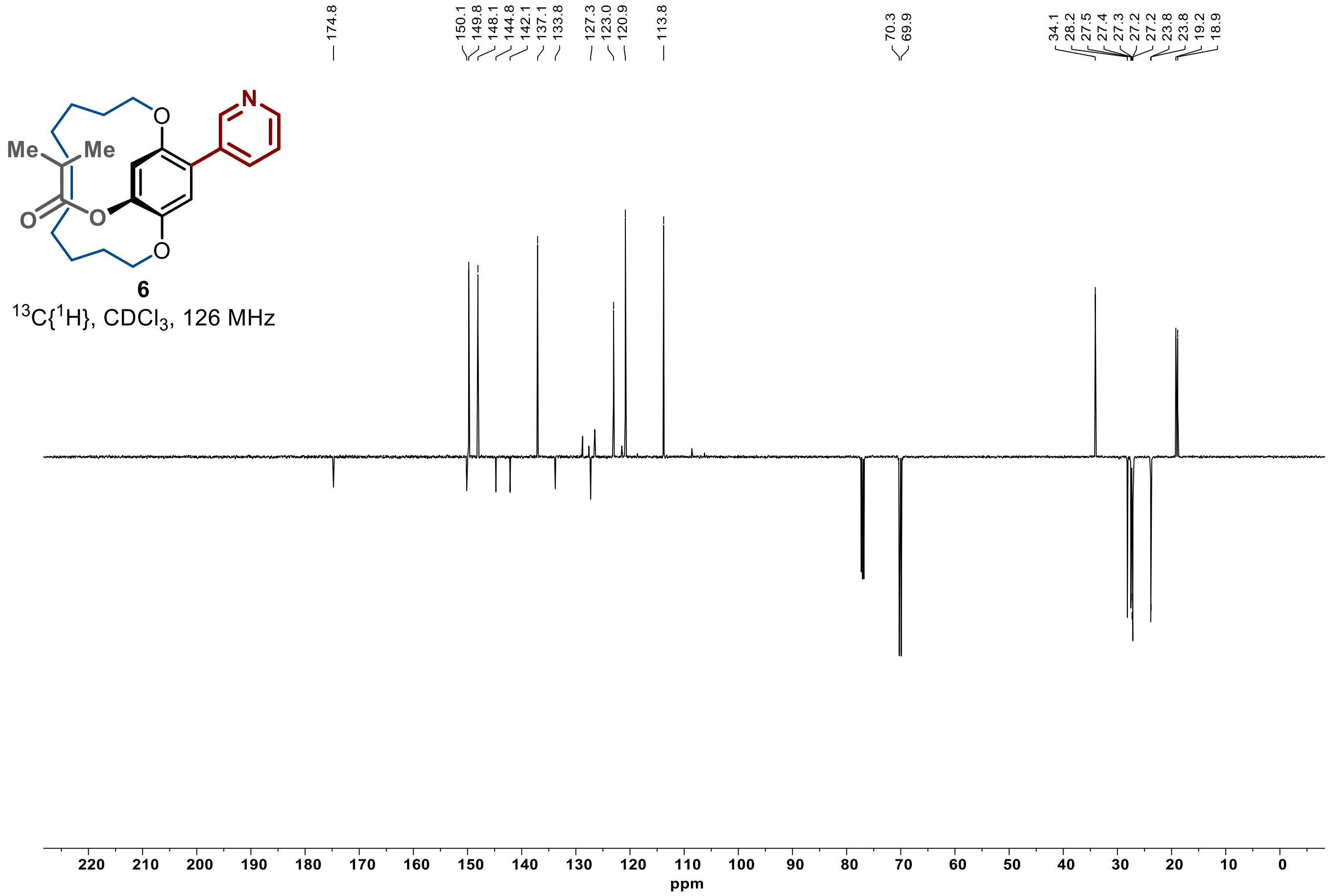

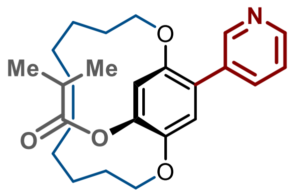

6

2D ^{1}H, $CDCl_3$, COSY

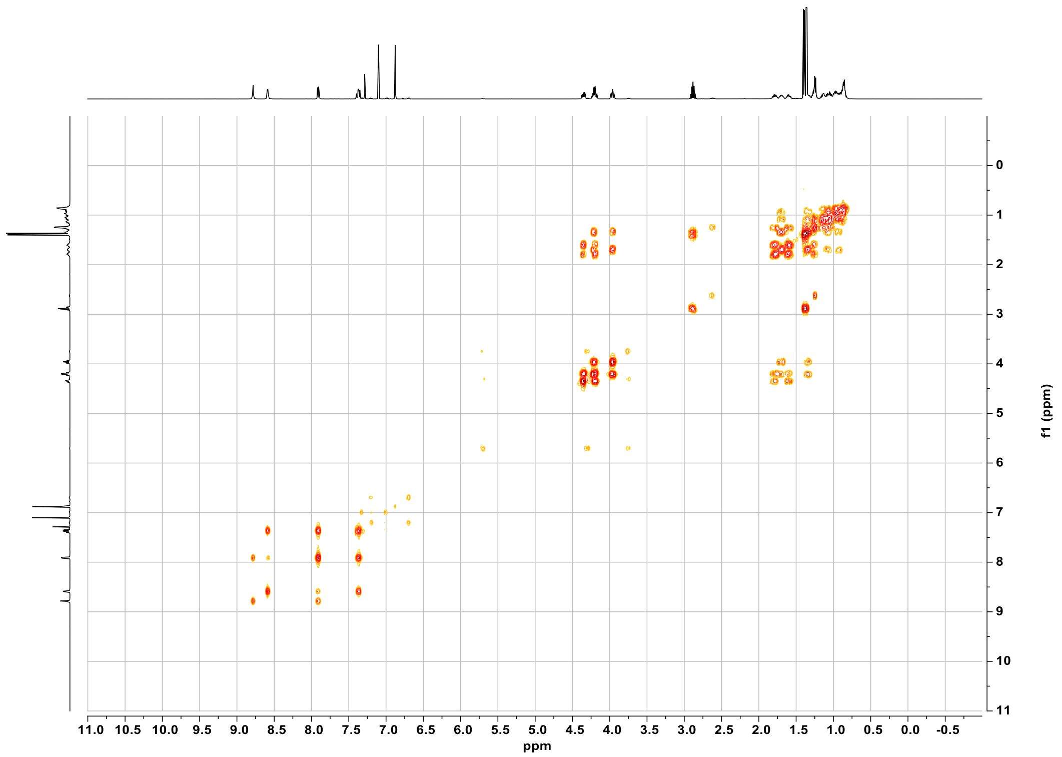

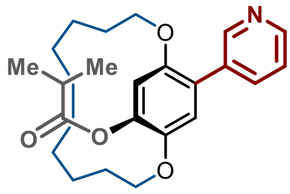

6

2D ^{1}H-^{13}C, $CDCl_3$, HSQC

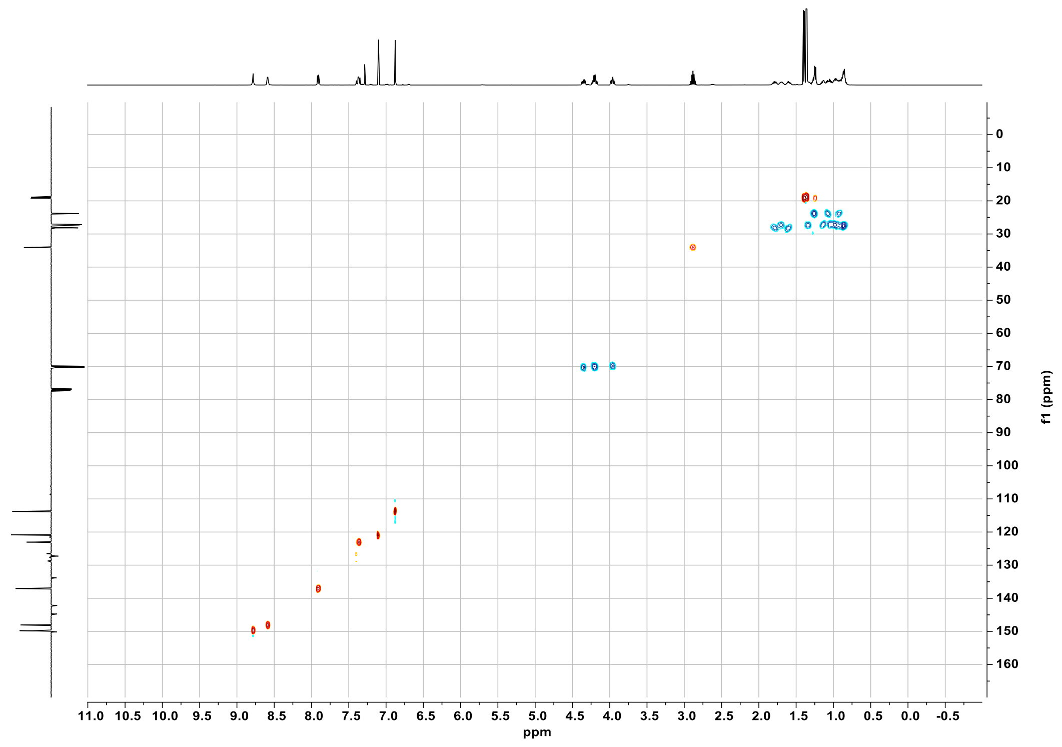

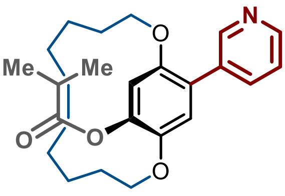

6

2D ^1H-^{13}C, $CDCl_3$, HMBC

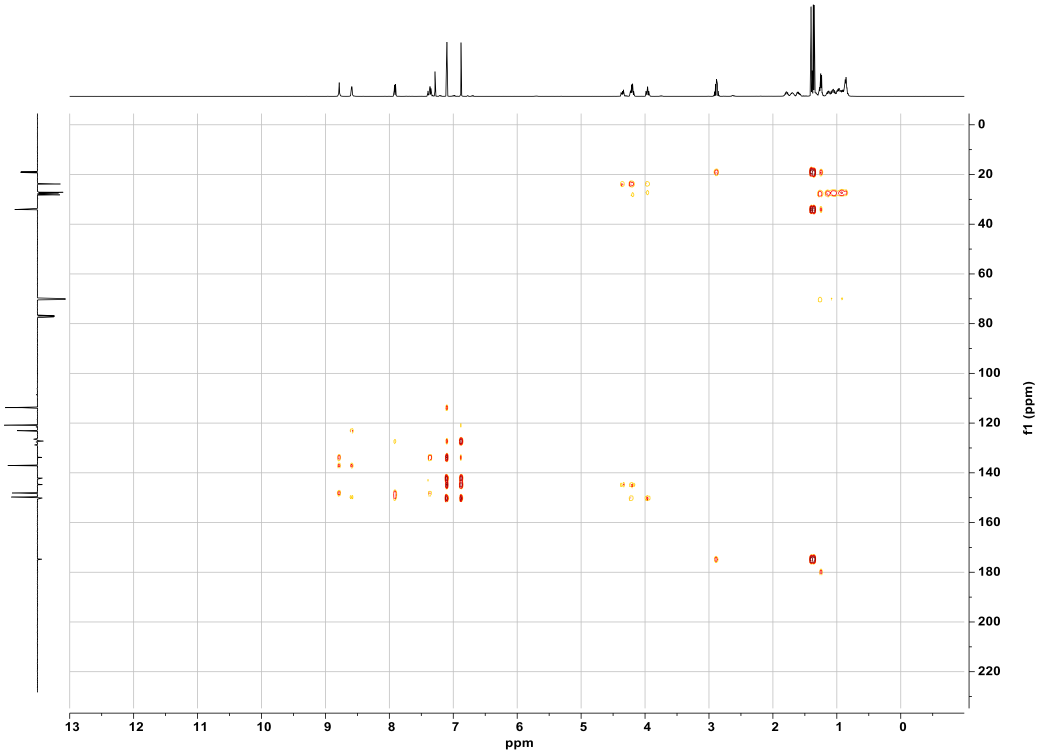

(*Rp*)-1⁵-bromo-2,13-dioxa-1(1,4)-benzenacyclotridecaphane-1²-yl acetate 7

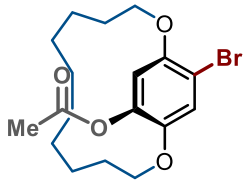

7

^{1}H, $CDCl_3$, 500 MHz

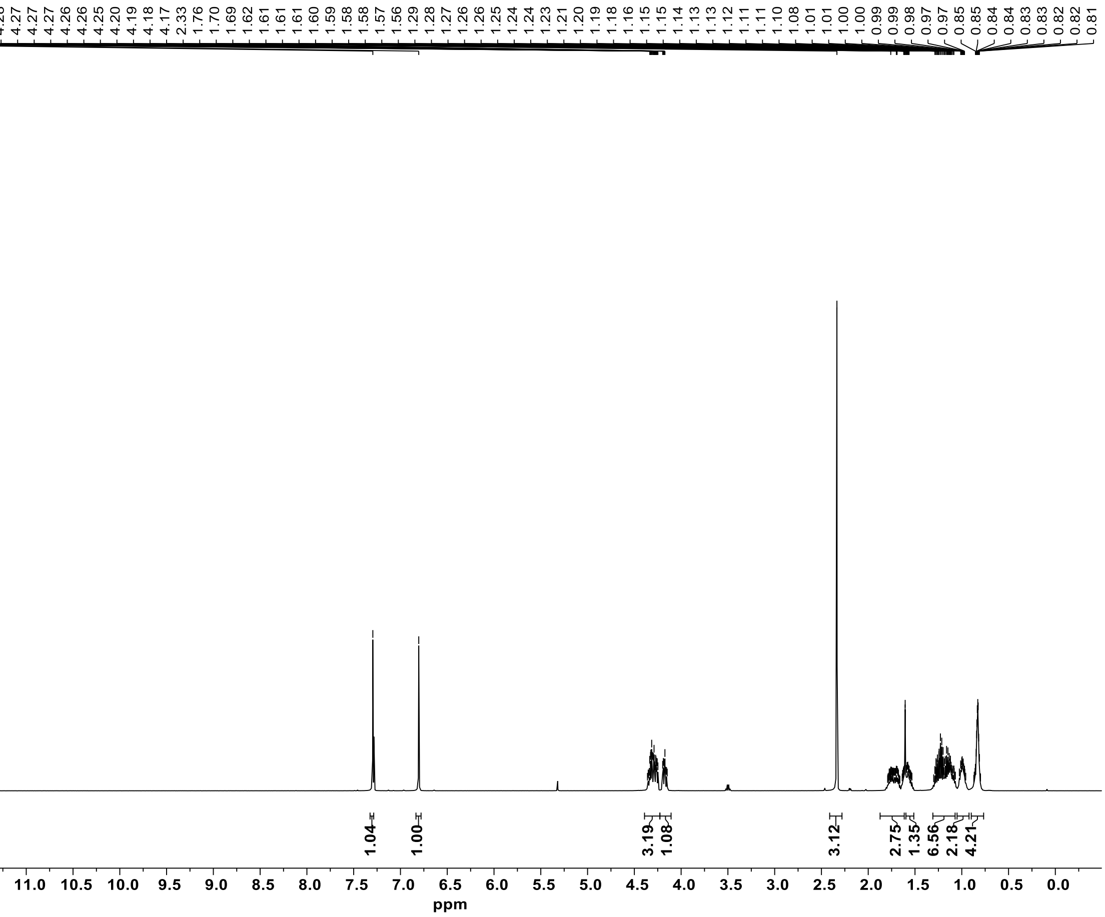

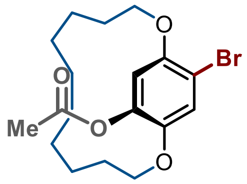

7

$^{13}C\{^{1}H\}$, $CDCl_3$, 126 MHz

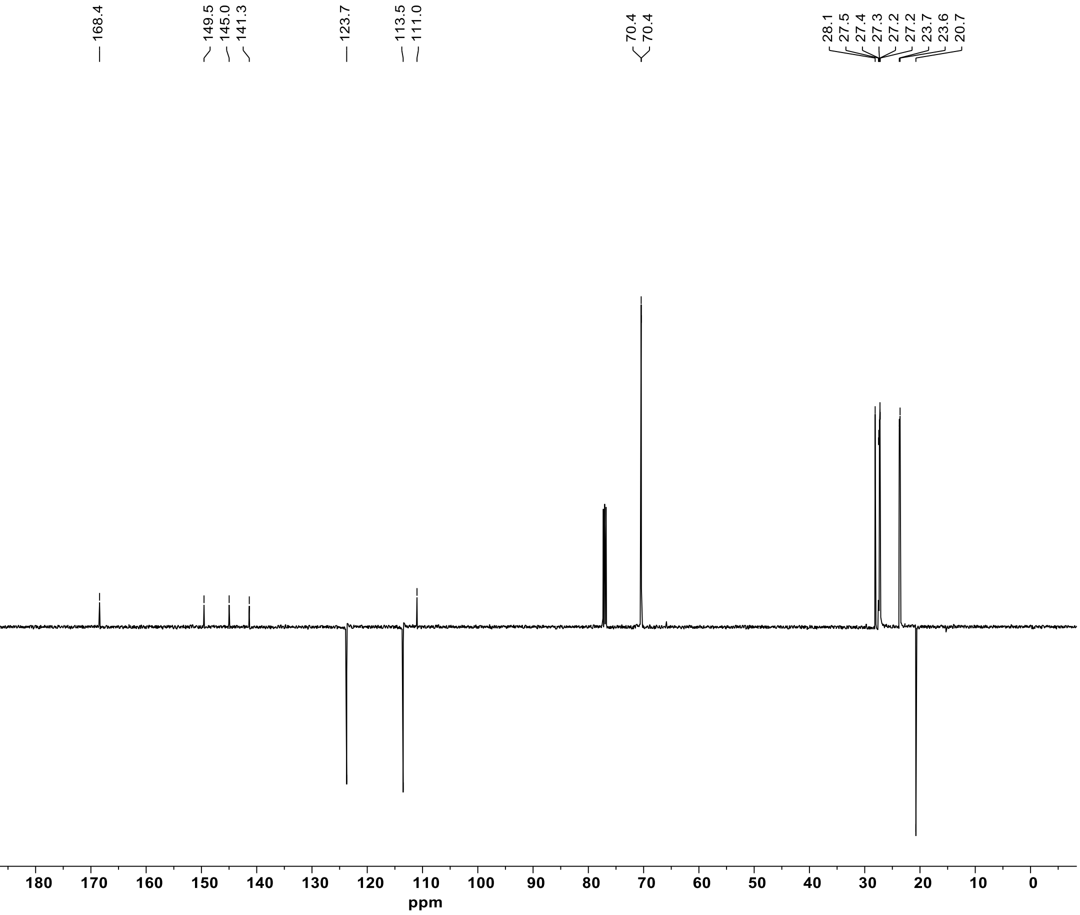

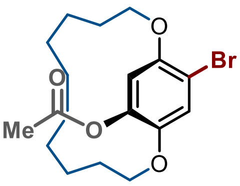

7

2D ^{1}H, $CDCl_3$, COSY

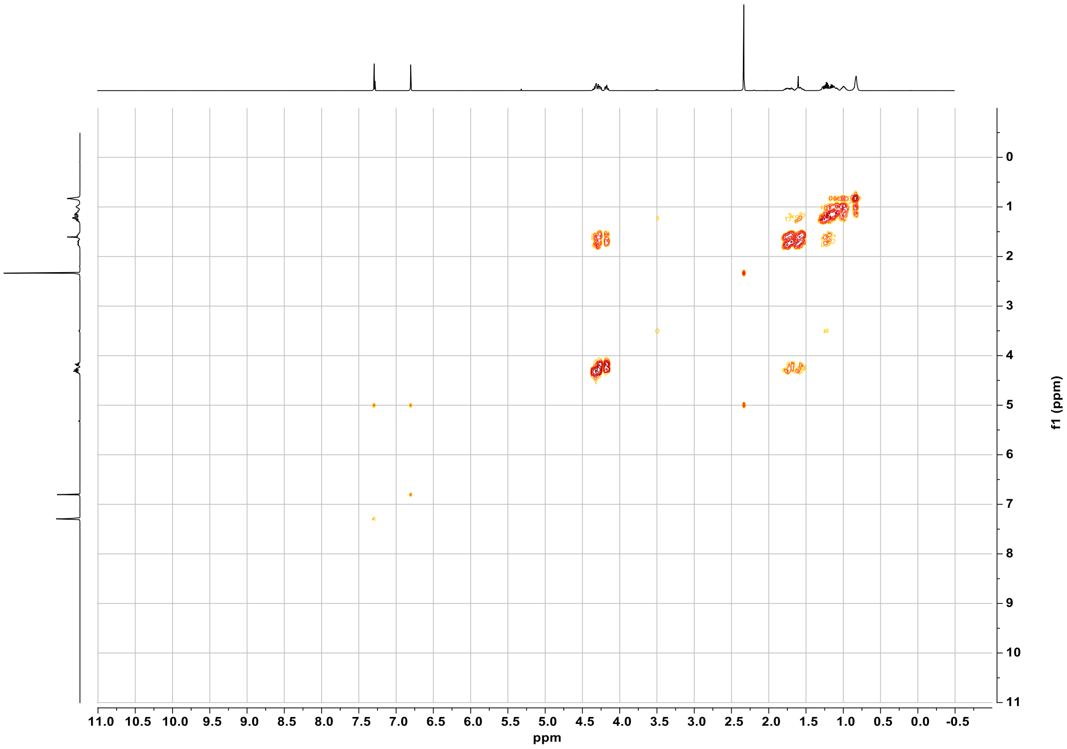

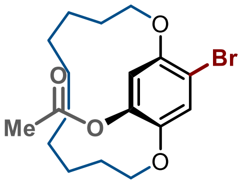

7

2D ^{1}H-^{13}C, $CDCl_3$, HSQC

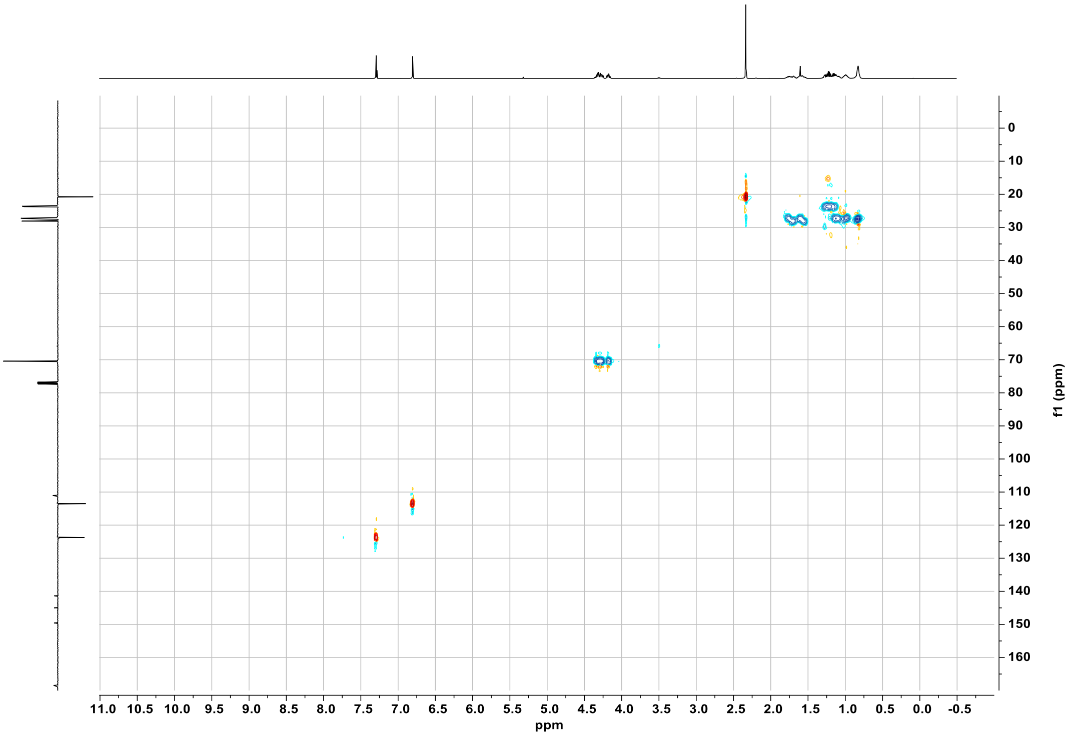

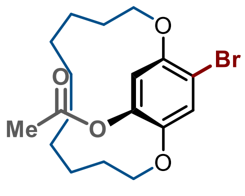

7

2D ^{1}H-^{13}C, $CDCl_3$, HMBC

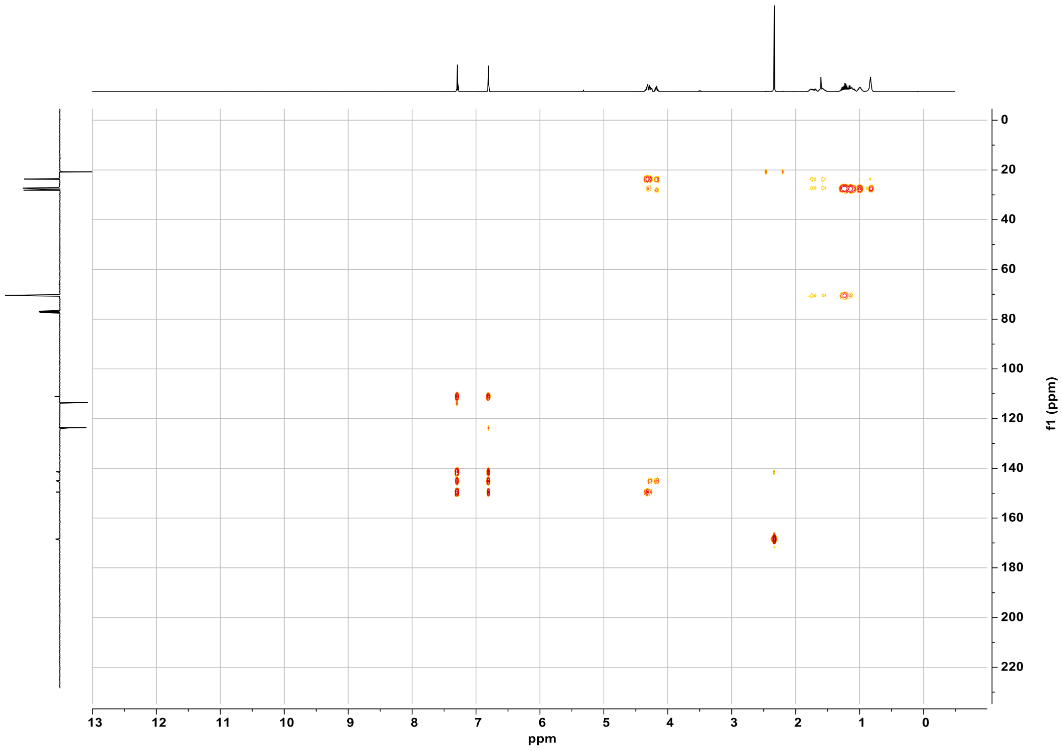

(*Rp*)-1⁵-bromo-2,14-dioxa-1(1,4)-benzenacyclotetradecaphane-1²-yl isobutyrate 8

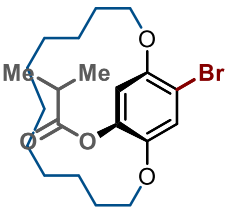

8

^{1}H, $CDCl_3$, 500 MHz

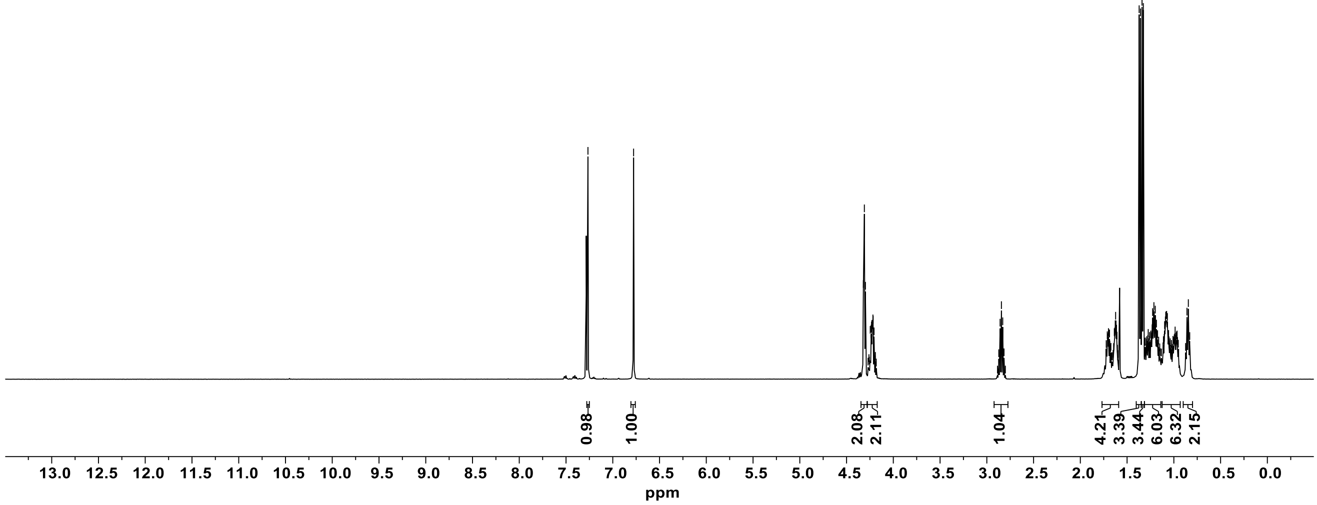

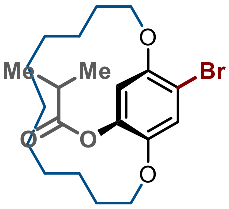

8

$^{13}C\{^{1}H\}$, $CDCl_3$, 126 MHz

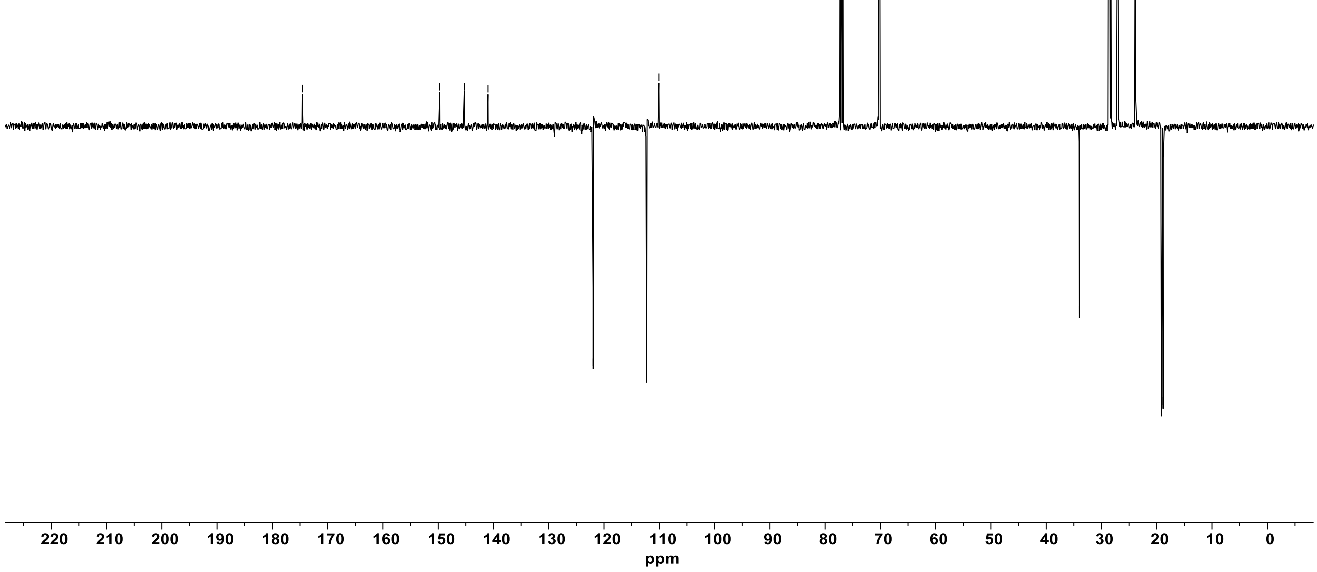

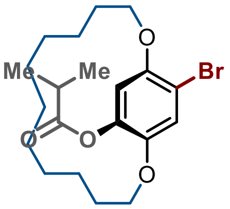

8

2D ^{1}H, $CDCl_3$, COSY

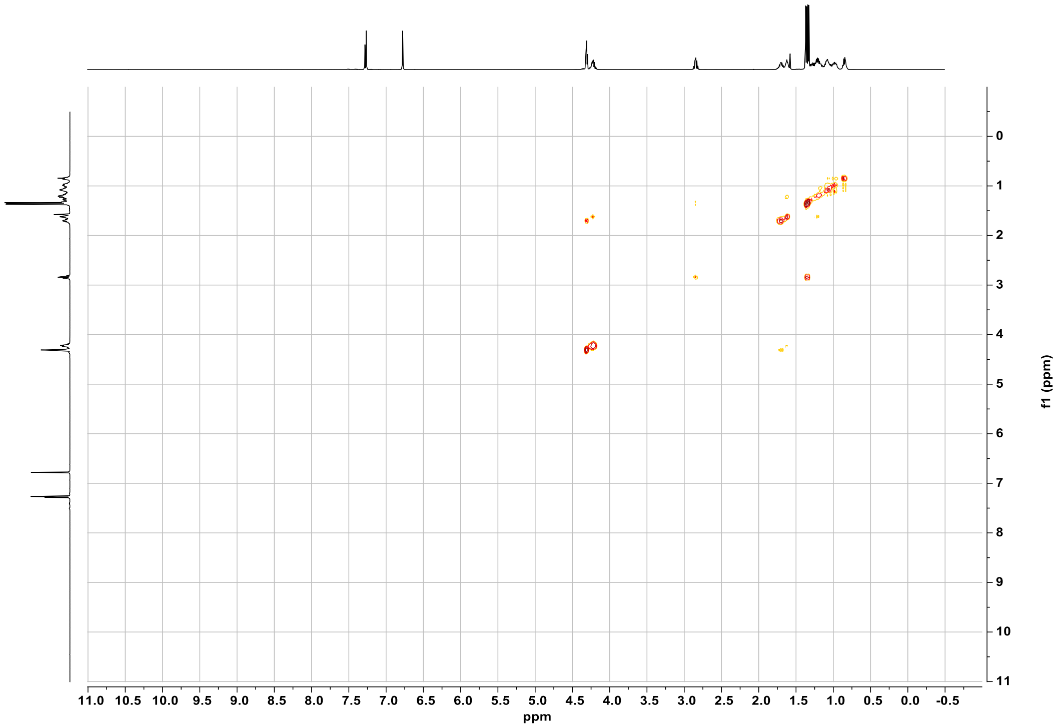

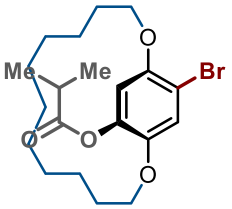

8

2D ^{1}H-^{13}C, $CDCl_3$, HSQC

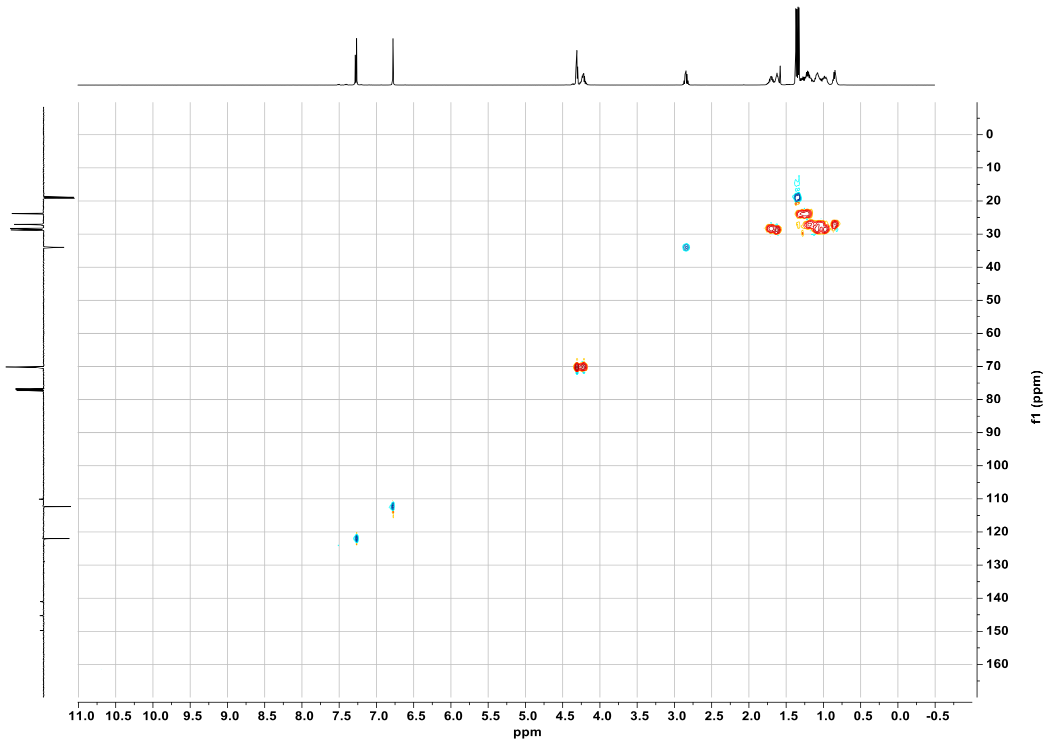

(*Rp*)-1⁵-phenyl-2,14-dioxa-1(1,4)-benzenacyclotetradecaphane-1²-yl isobutyrate 9

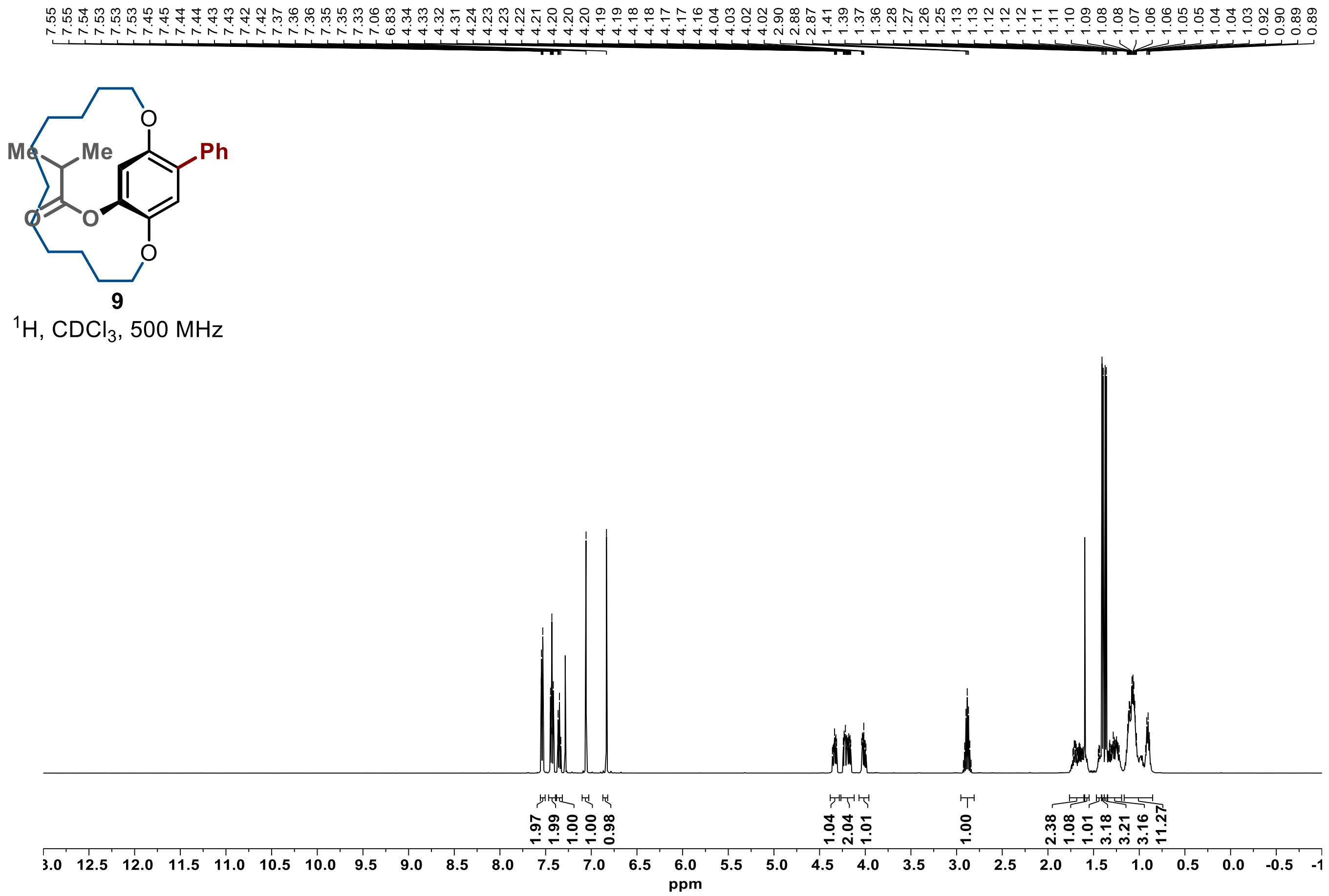

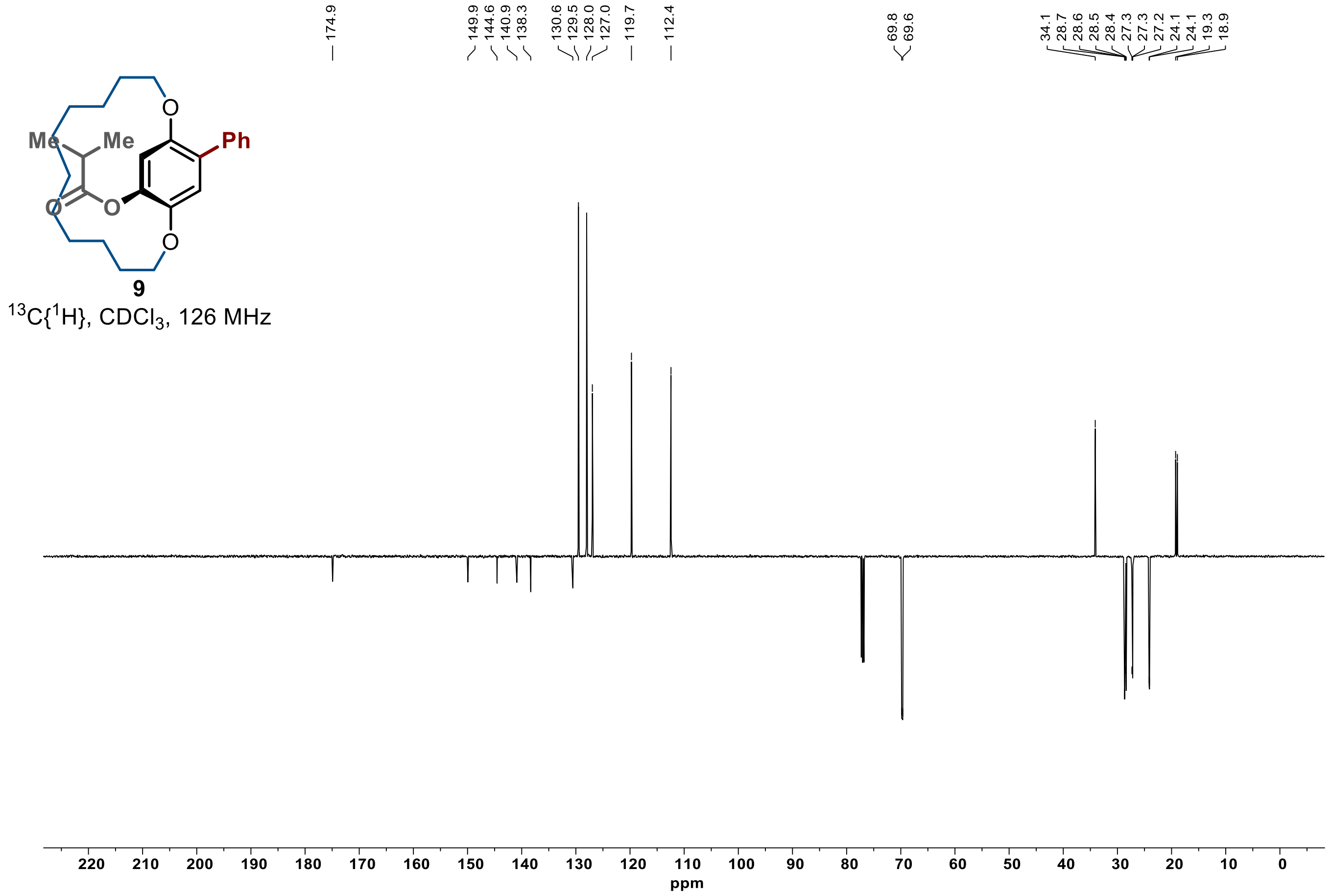

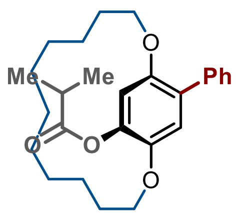

9

2D ^{1}H, $CDCl_3$, COSY

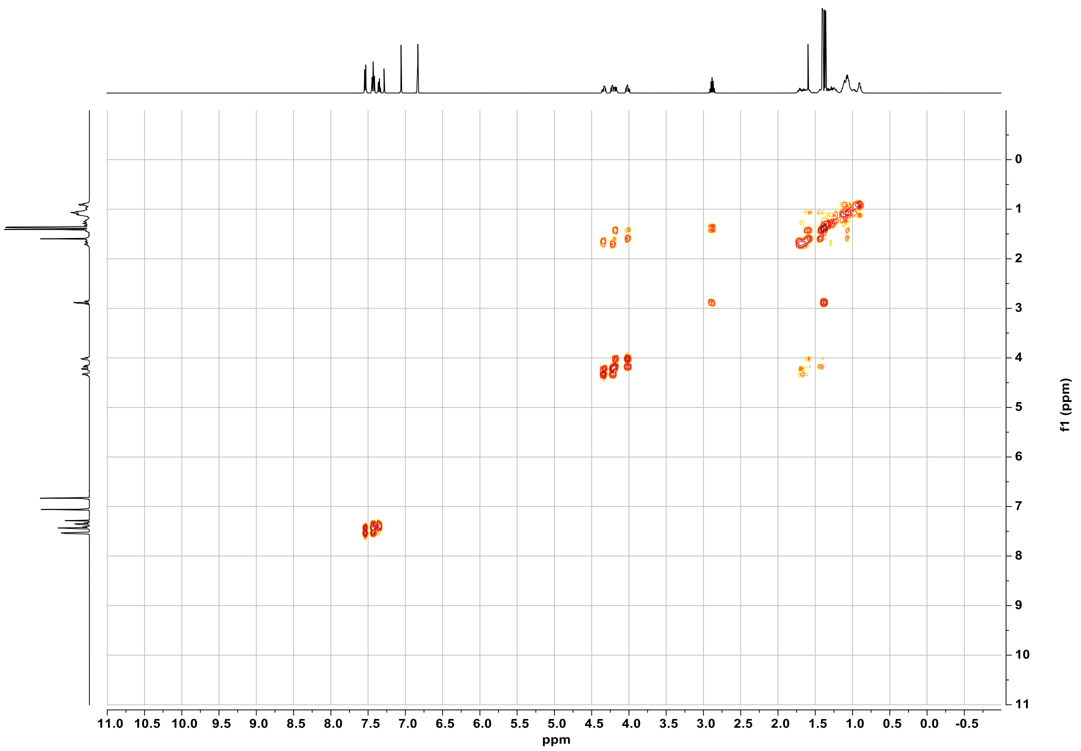

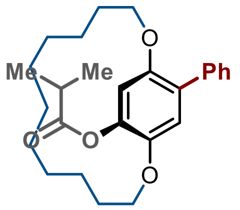

9

2D ^{1}H-^{13}C, $CDCl_3$, HSQC

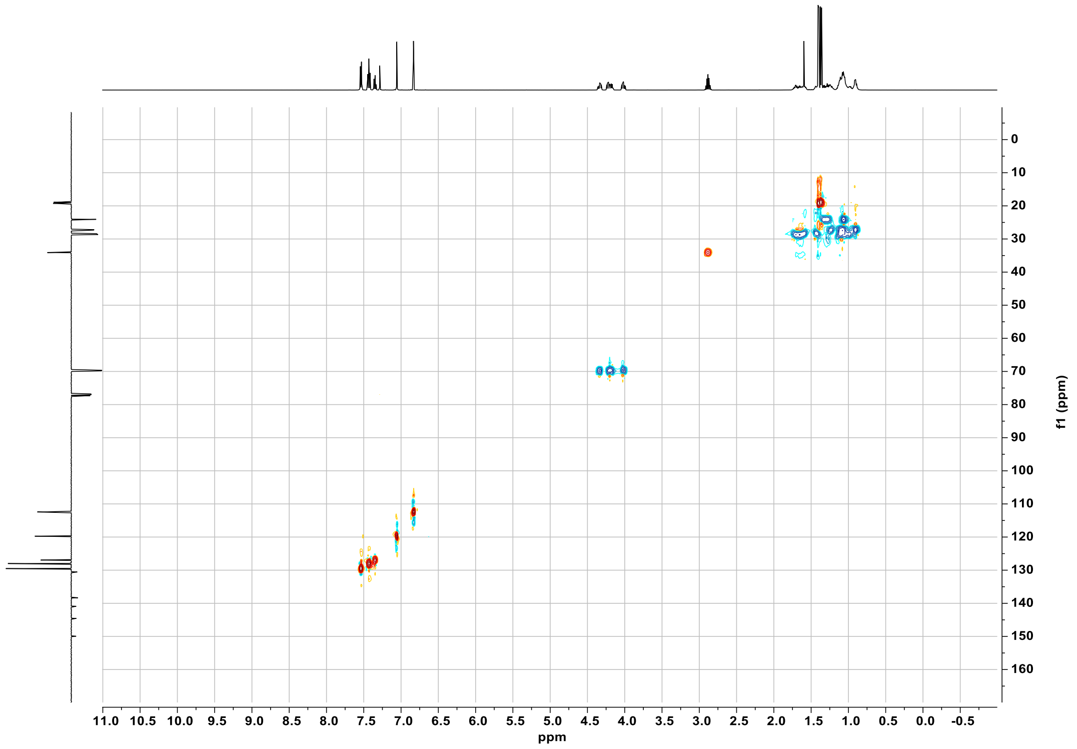

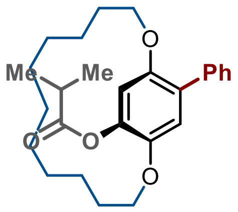

9

2D ^{1}H-^{13}C, $CDCl_3$, HMBC

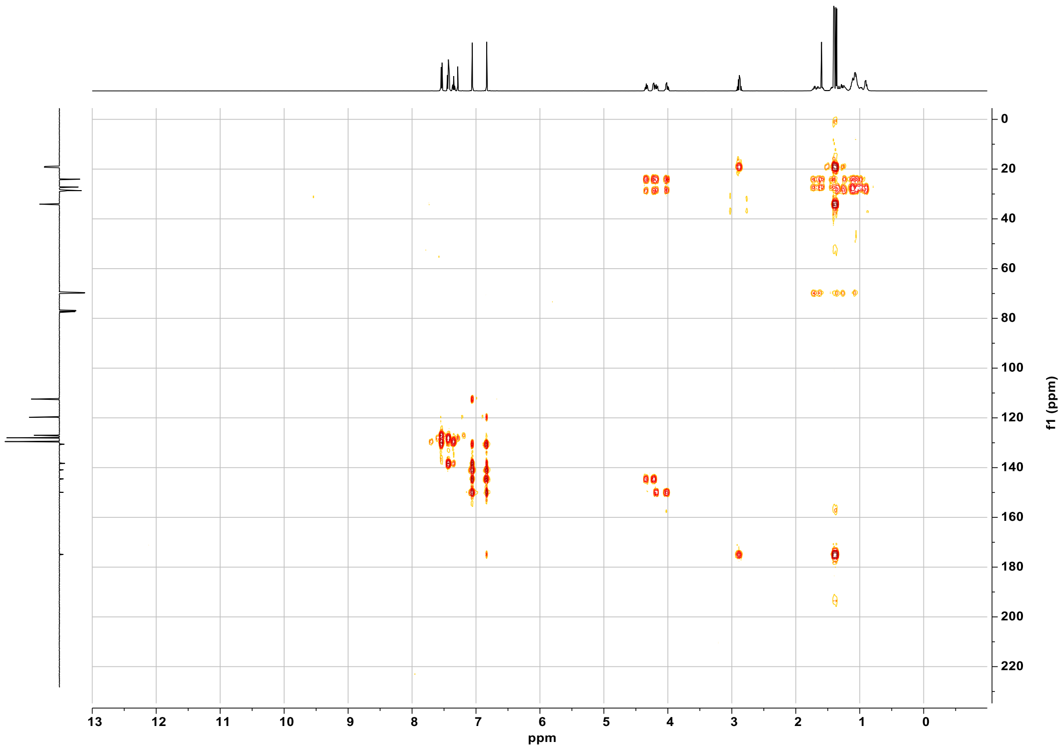

(*Rp*)-1⁵-phenyl-2,15-dioxa-1(1,4)-benzenacyclopentadecaphane-1²-yl isobutyrate 11

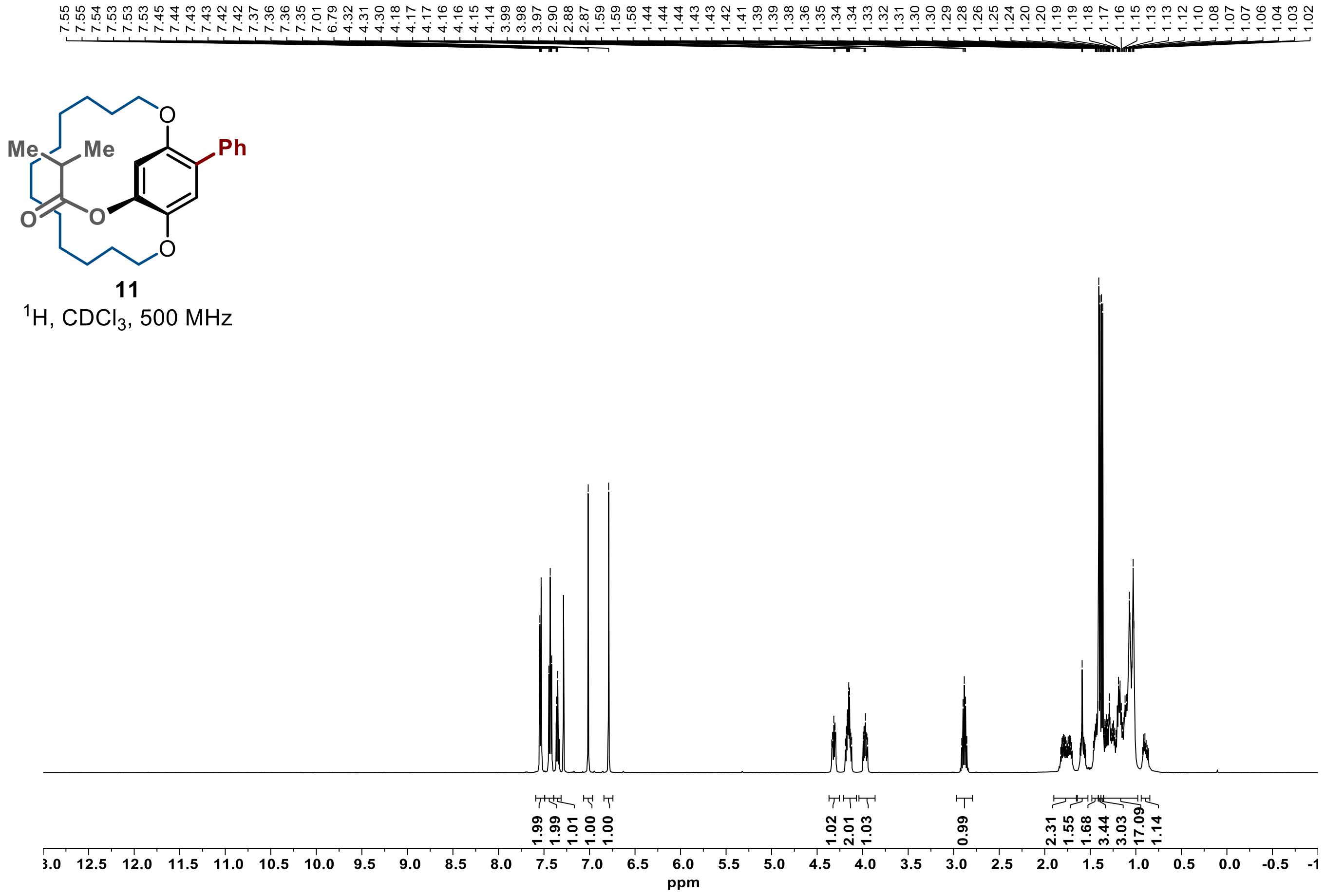

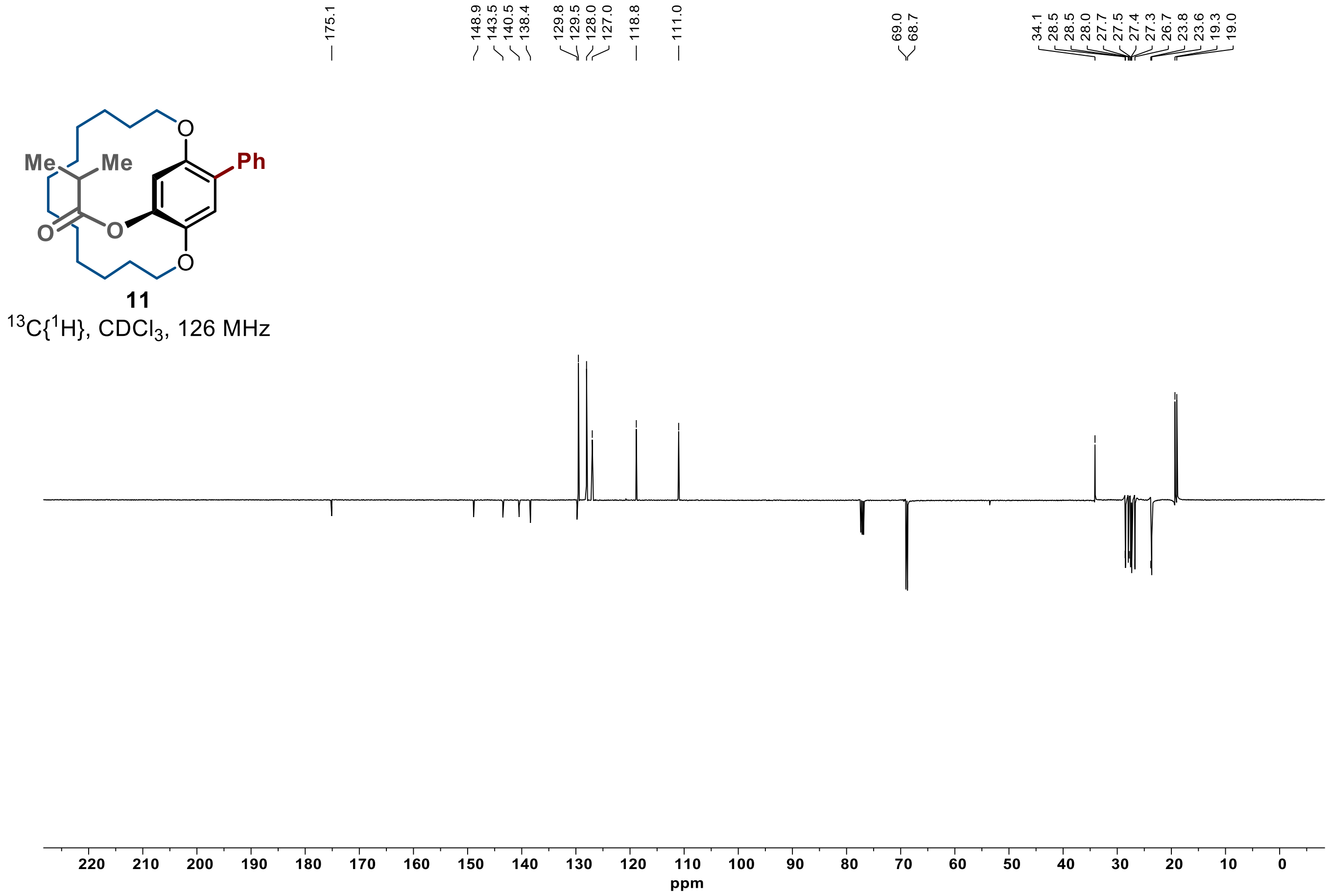

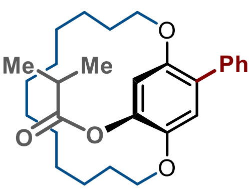

11

2D ^{1}H, $CDCl_3$, COSY

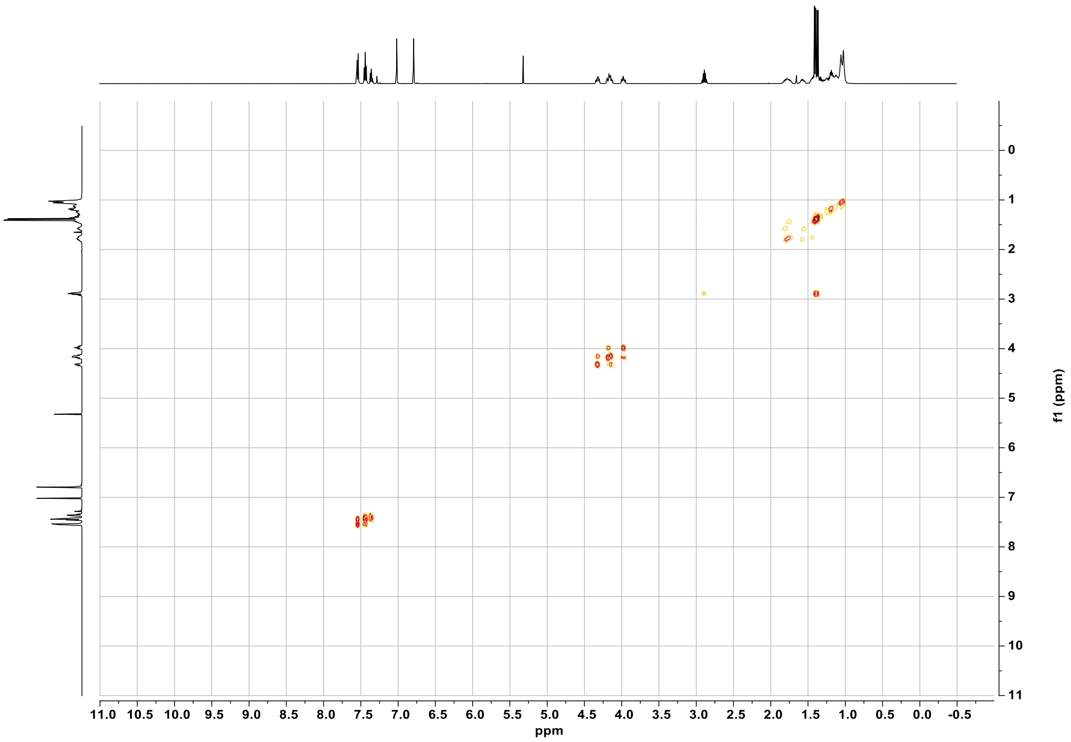

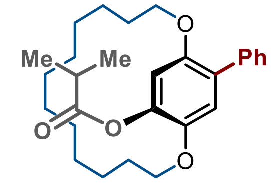

11

2D ^{1}H-^{13}C, $CDCl_3$, HSQC

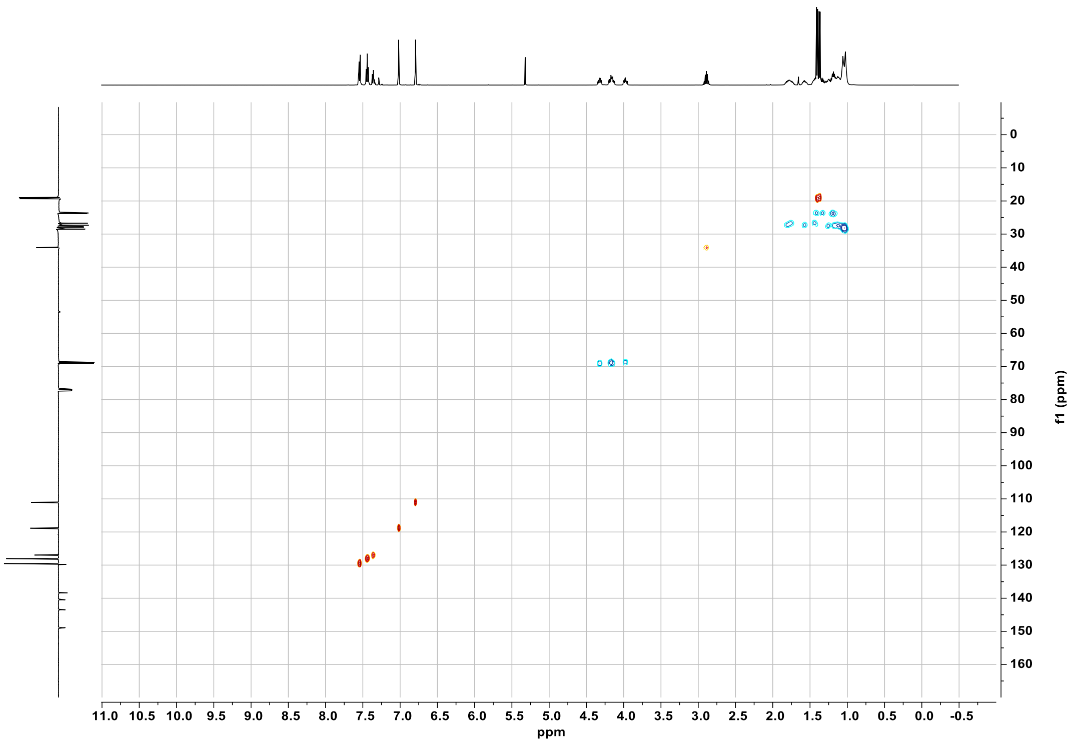

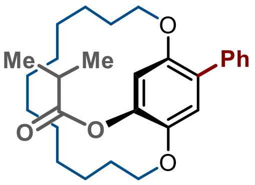

11

2D ^{1}H-^{13}C, $CDCl_3$, HMBC

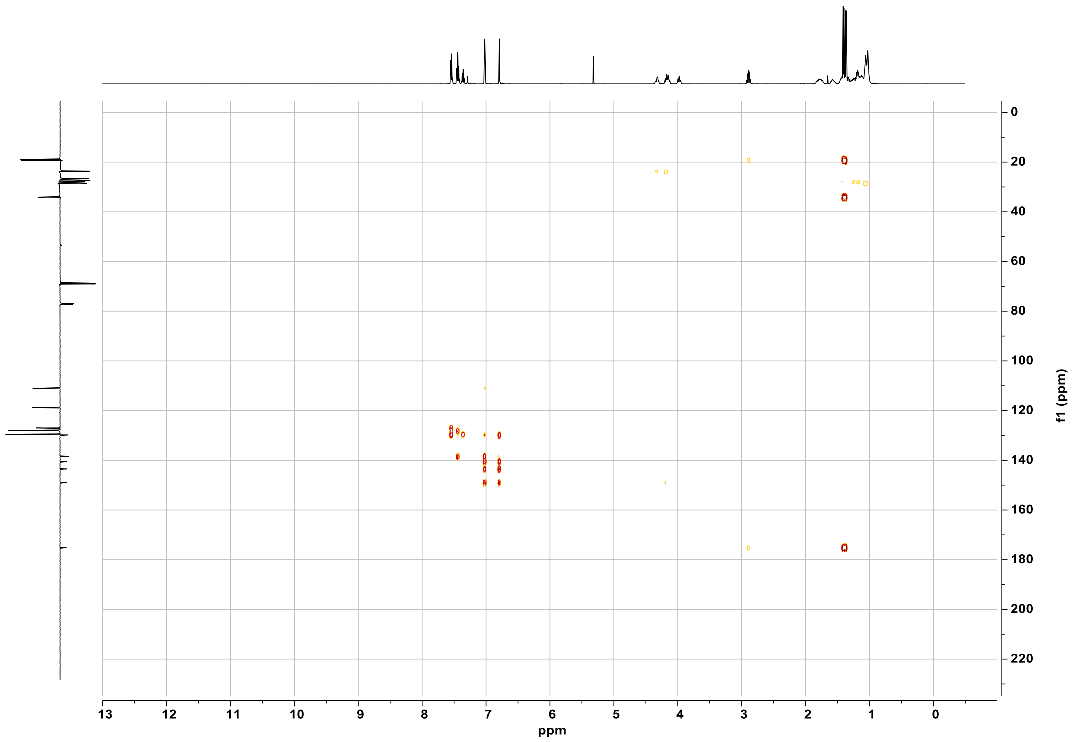

(*Rp*)-1⁵-phenyl-2,15-dioxa-1(1,4)-benzenacyclopentadecaphane-1²-yl propionate S70

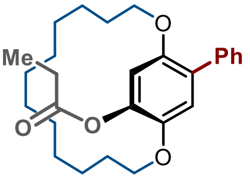

S70
^{1}H, $CDCl_3$, 500 MHz

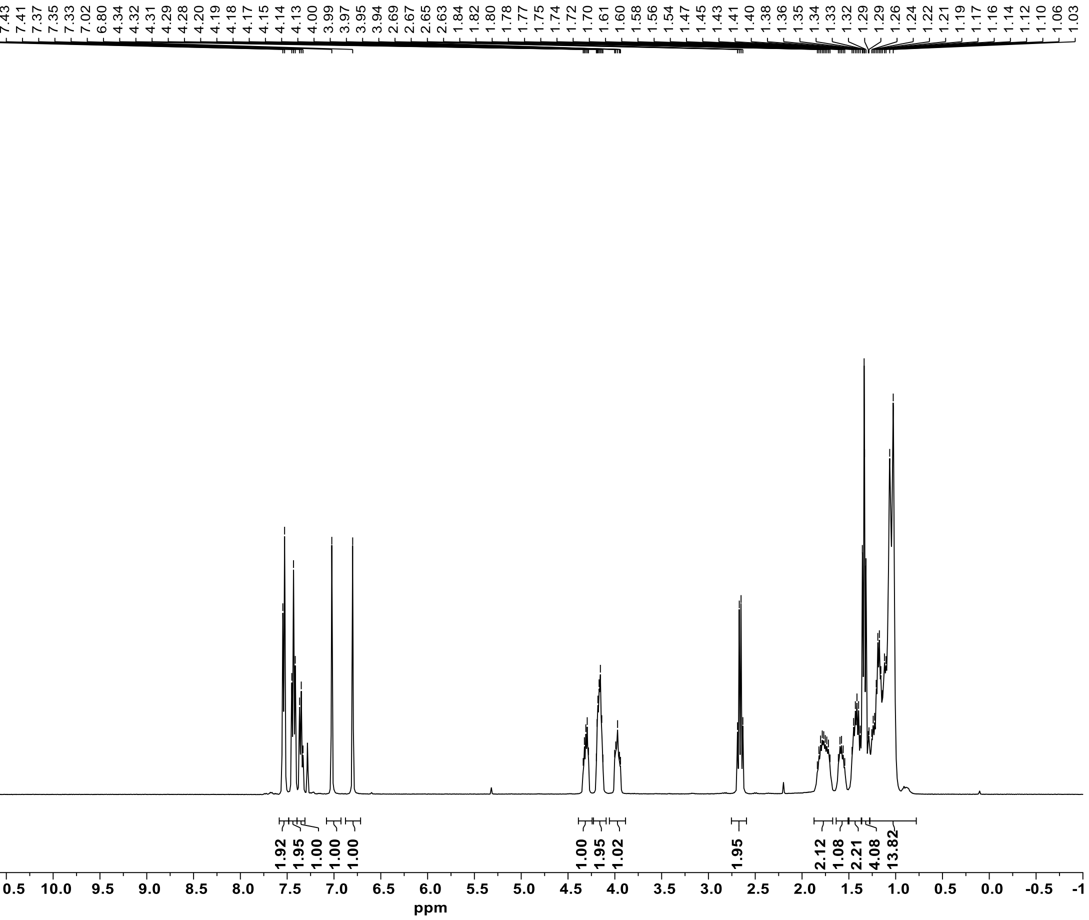

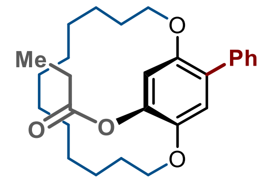

S70
^{13}C{^{1}H}, $CDCl_3$, 126 MHz

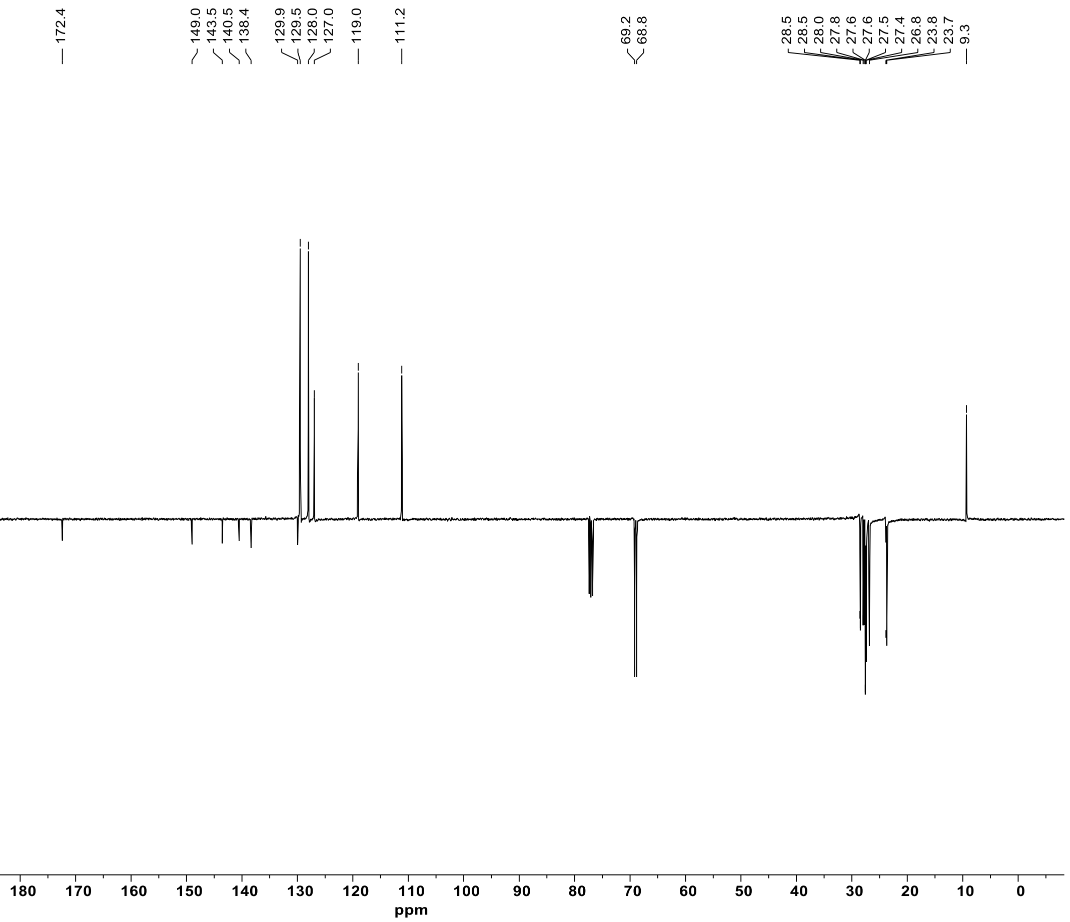

(*Rp*)-1⁵-phenyl-2,15-dioxa-1(1,4)-benzenacyclopentadecaphane-1²-yl benzoate S71

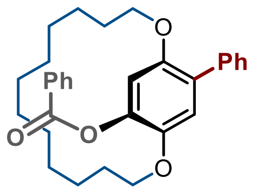

S71

^{1}H, $CDCl_3$, 500 MHz

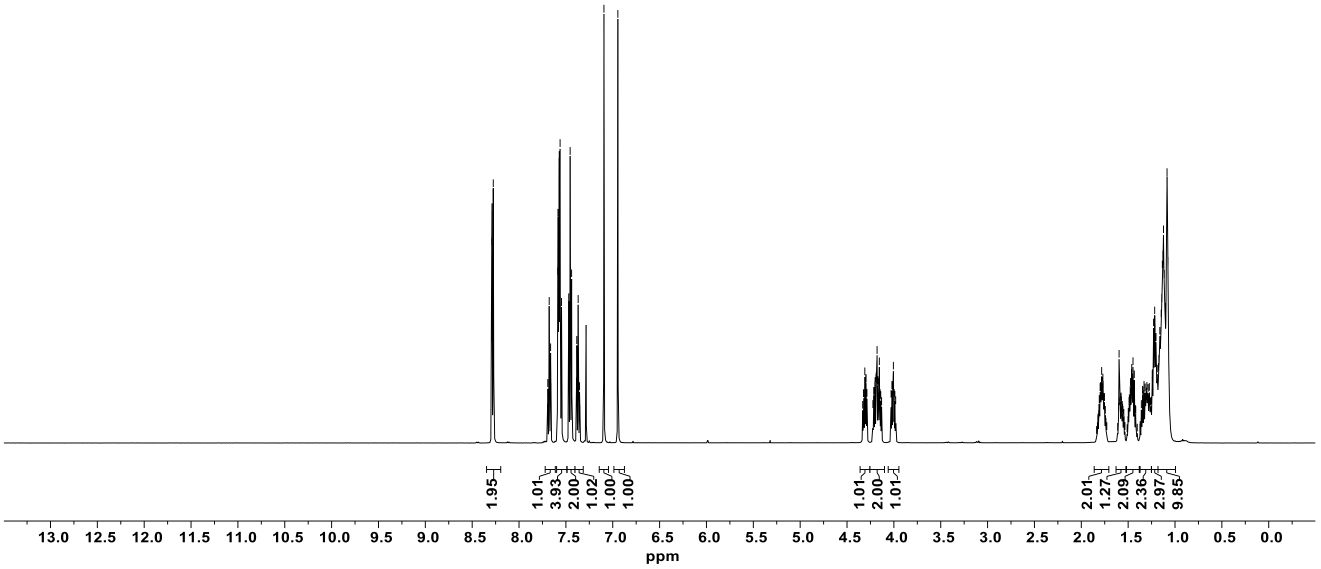

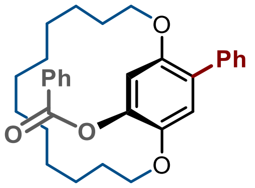

S71

$^{13}C\{^{1}H\}$, $CDCl_3$, 126 MHz

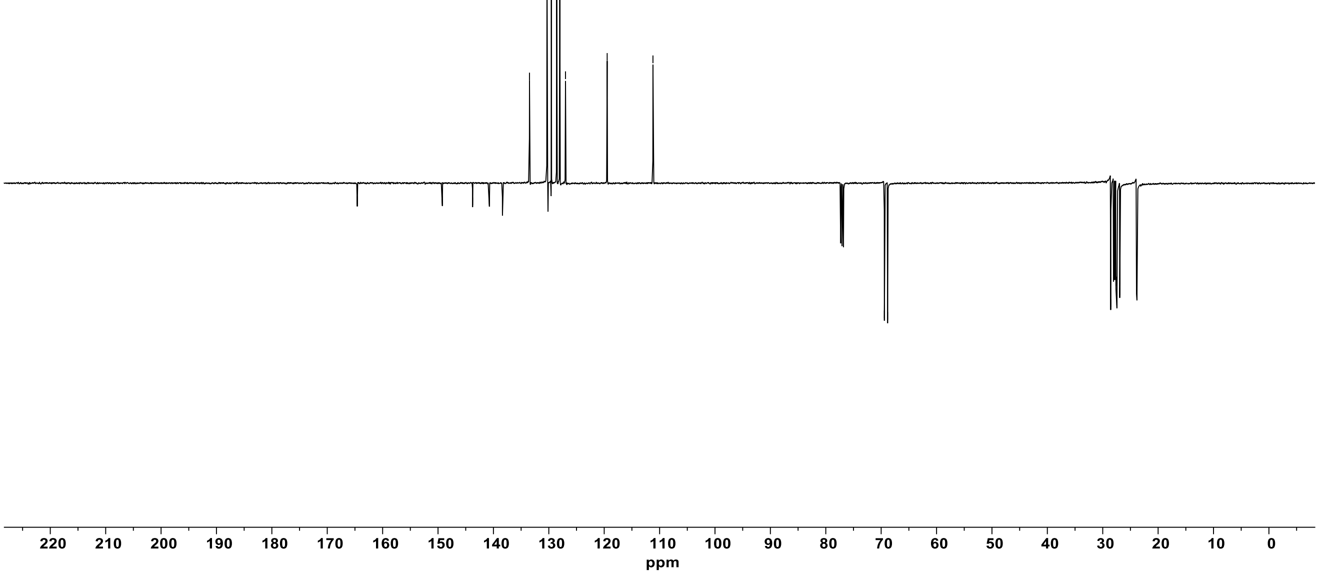

(*Rp*)-1[5]-bromo-2,15-dioxa-1(1,4)-benzenacyclopentadecaphane-1[2]-yl isobutyrate 12

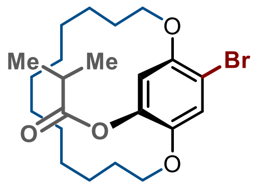

12
^{1}H, $CDCl_3$, 500 MHz

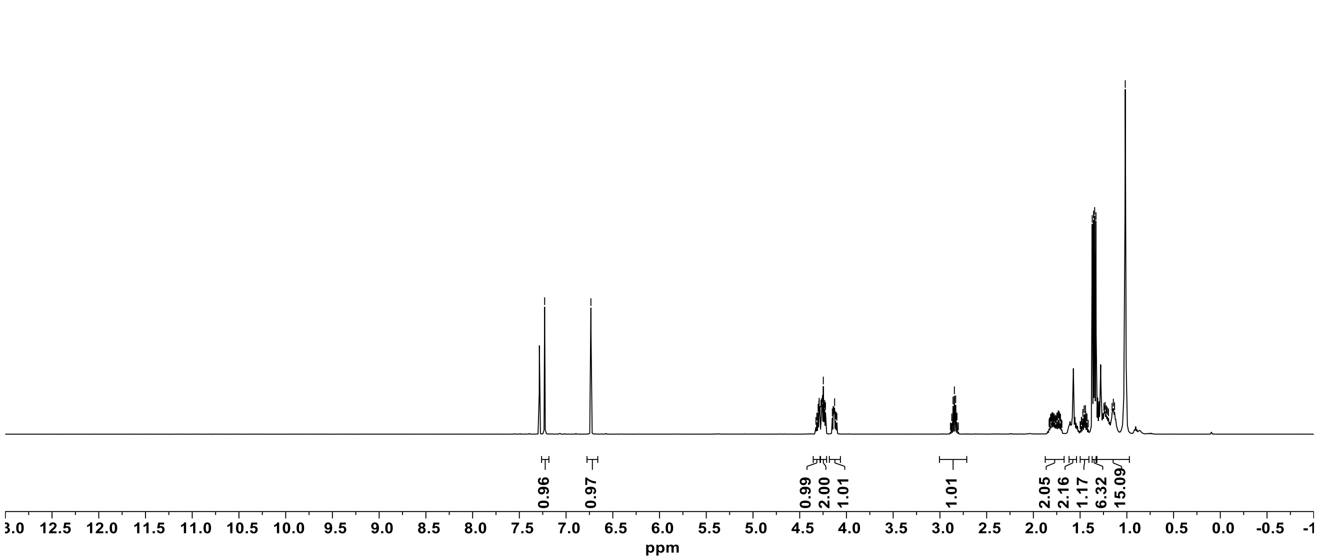

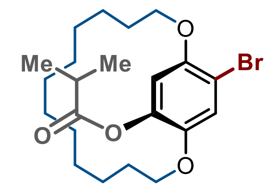

12
$^{13}C\{^1H\}$, $CDCl_3$, 126 MHz

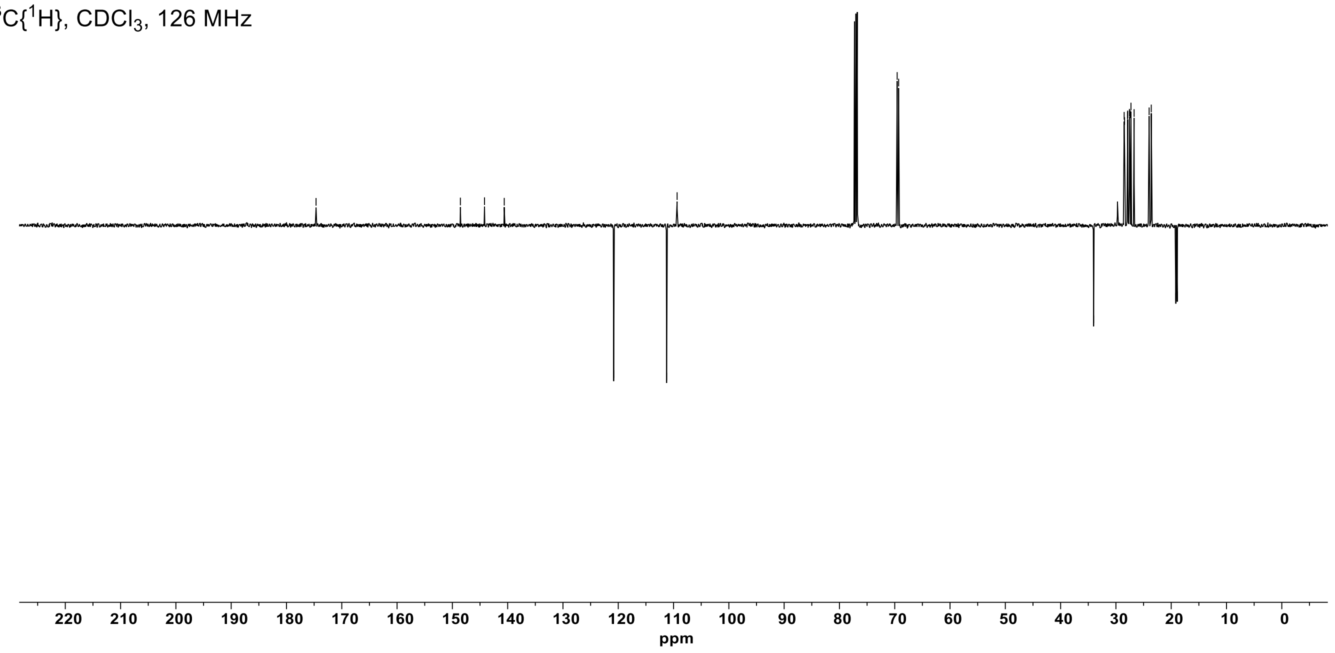

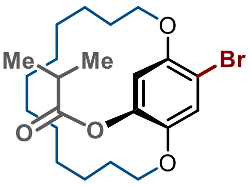

12

2D ^{1}H, $CDCl_3$, COSY

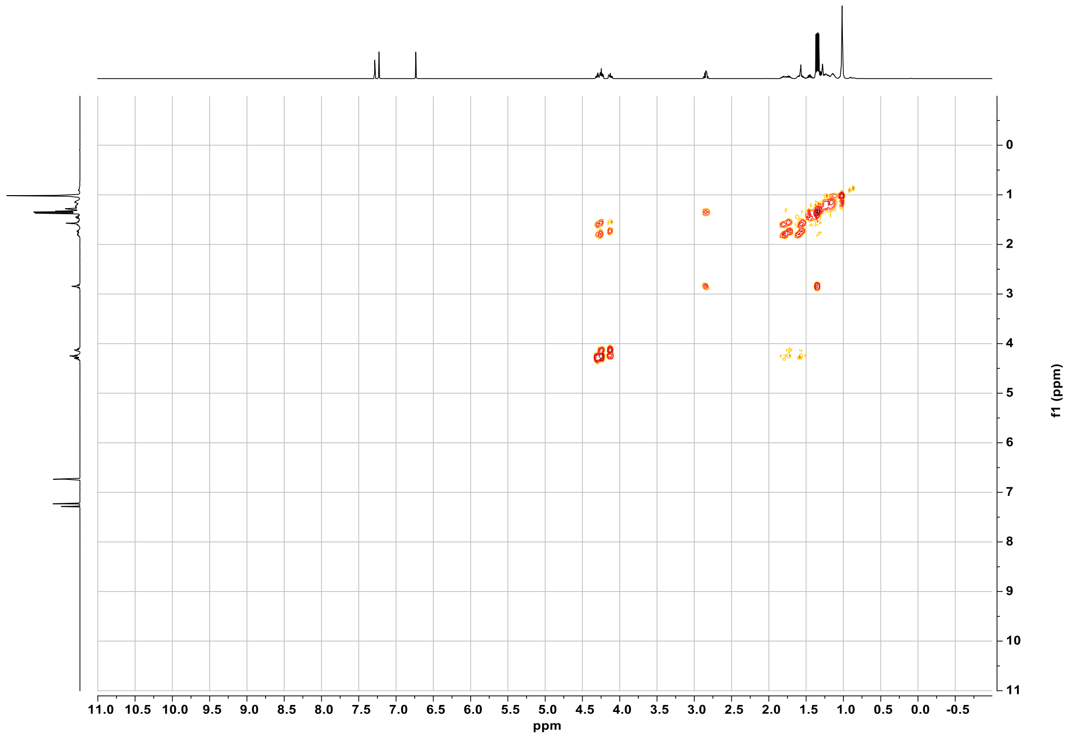

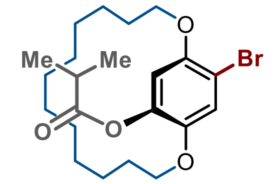

12

2D ^{1}H-^{13}C, $CDCl_3$, HSQC

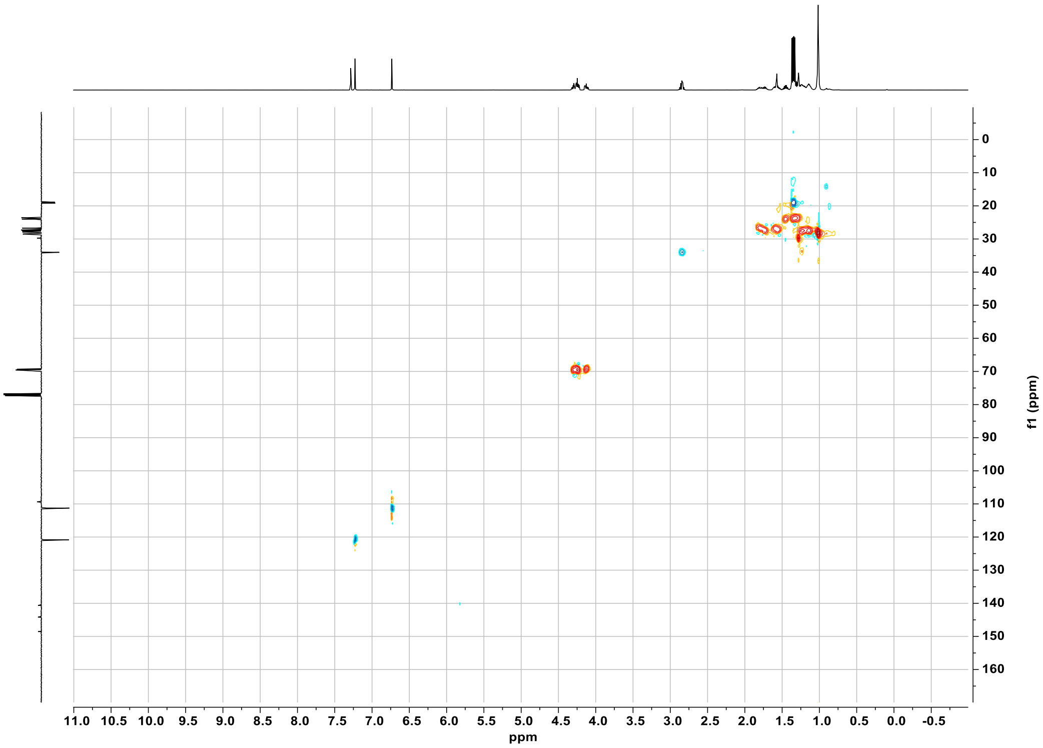

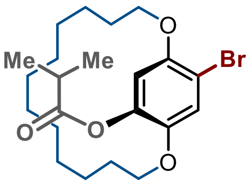

12

2D ^{1}H-^{13}C, $CDCl_3$, HMBC

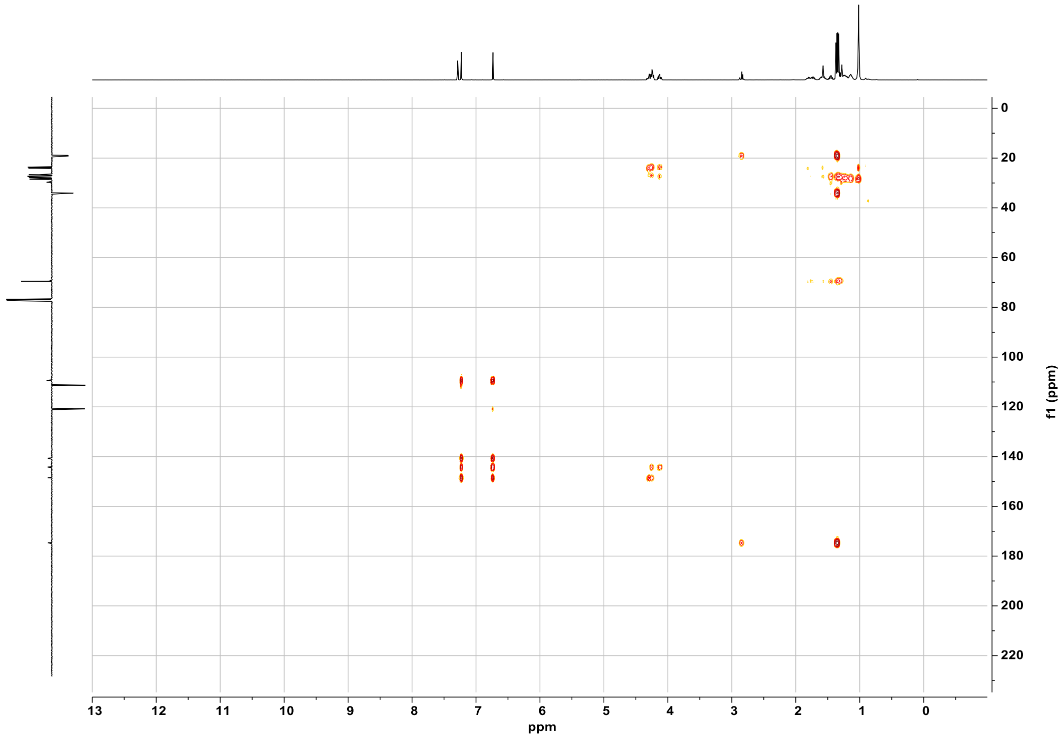

(*Rp*)-1^5-vinyl-2,15-dioxa-1(1,4)-benzenacyclopentadecaphane-1^2-yl isobutyrate 13

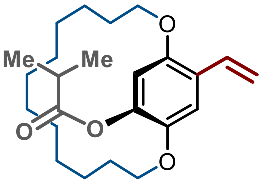

13

^{1}H, CDCl$_3$, 500 MHz

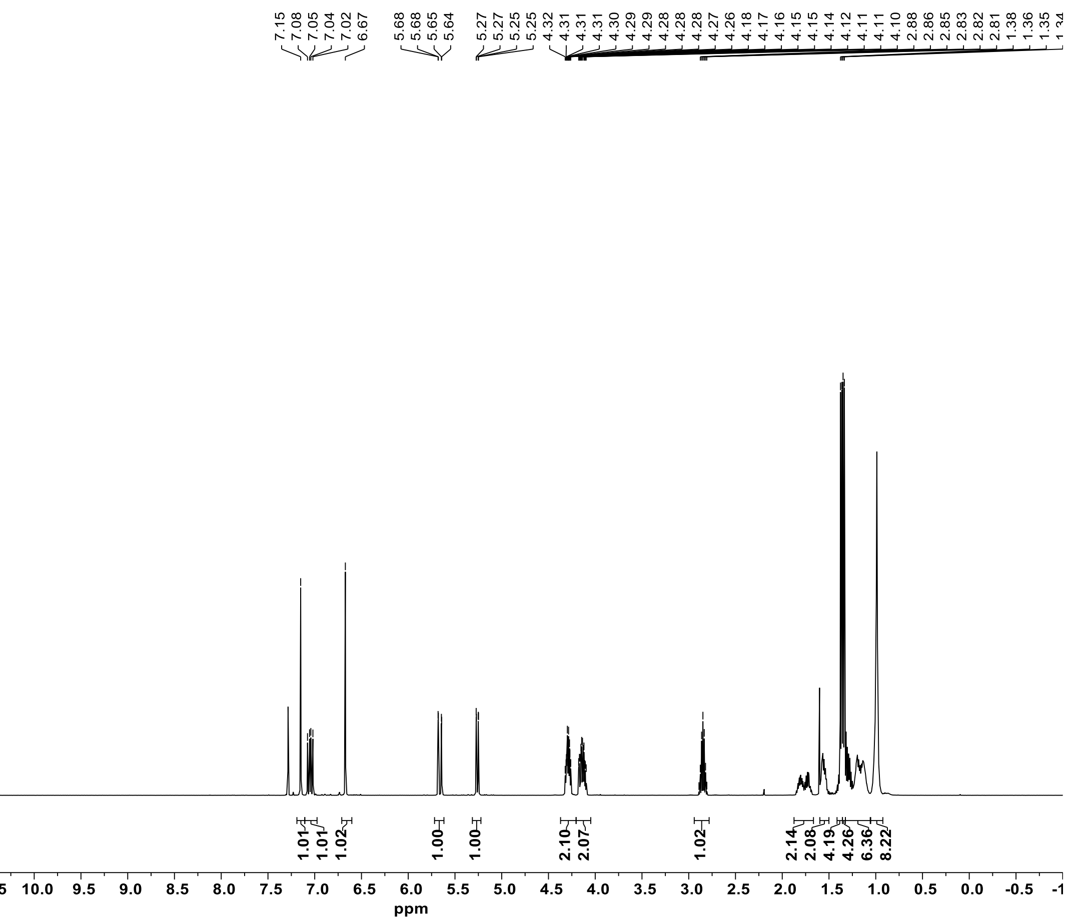

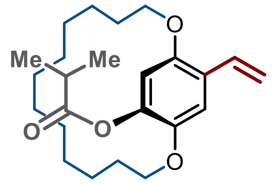

13

^{13}C{^{1}H}, CDCl$_3$, 126 MHz

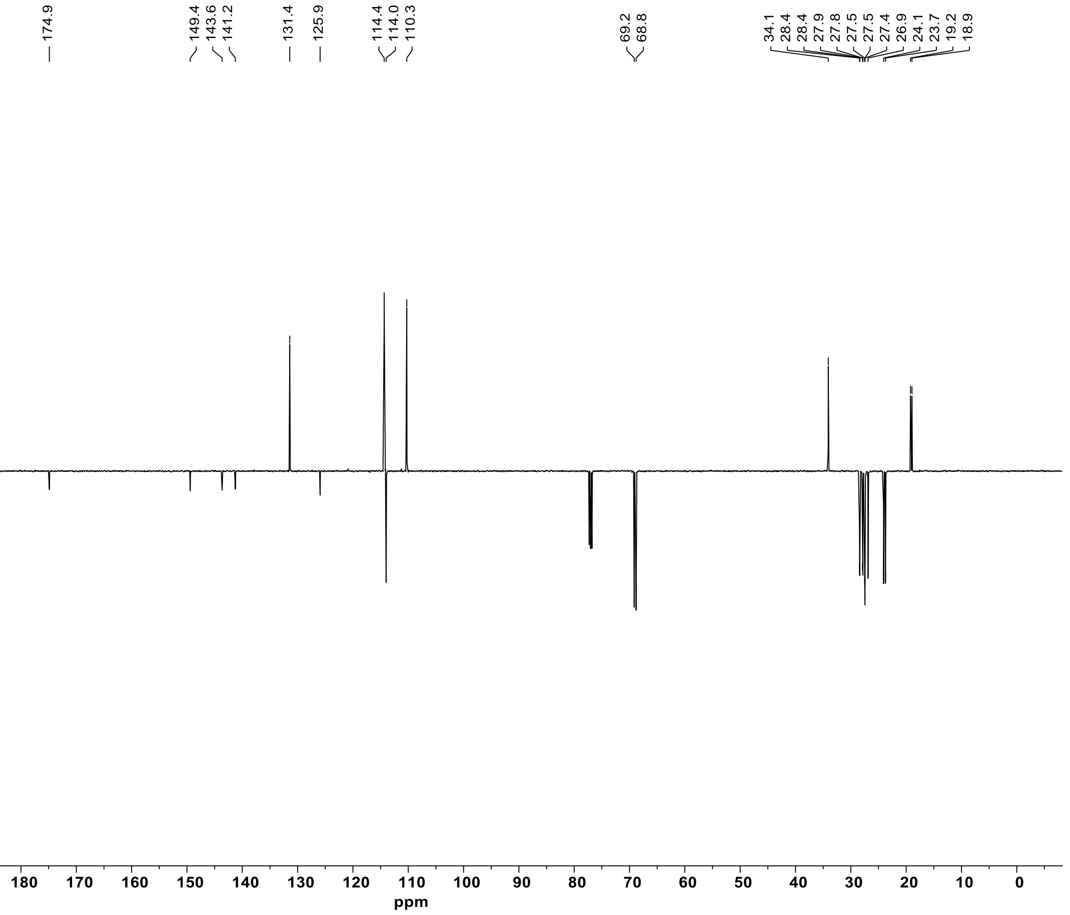

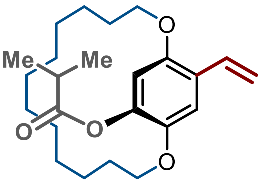

13

2D ^{1}H, $CDCl_3$, COSY

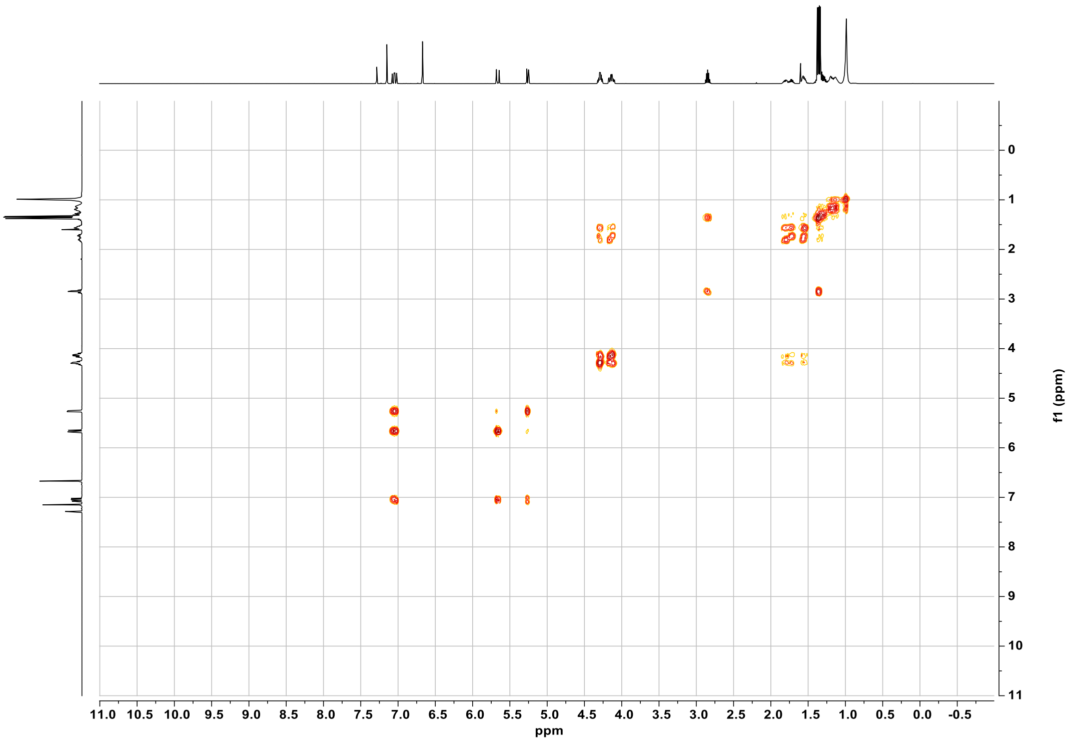

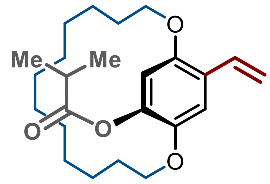

13

2D ^{1}H-^{13}C, $CDCl_3$, HSQC

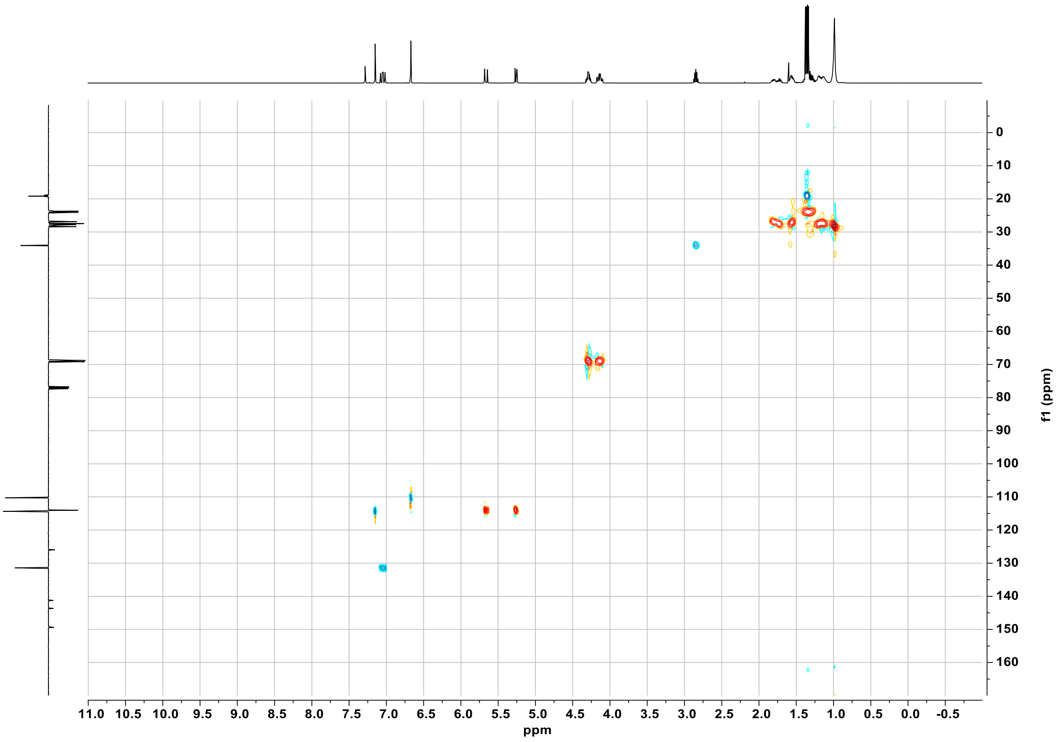

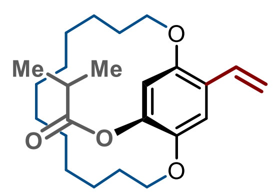

13

2D ^{1}H-^{13}C, $CDCl_3$, HMBC

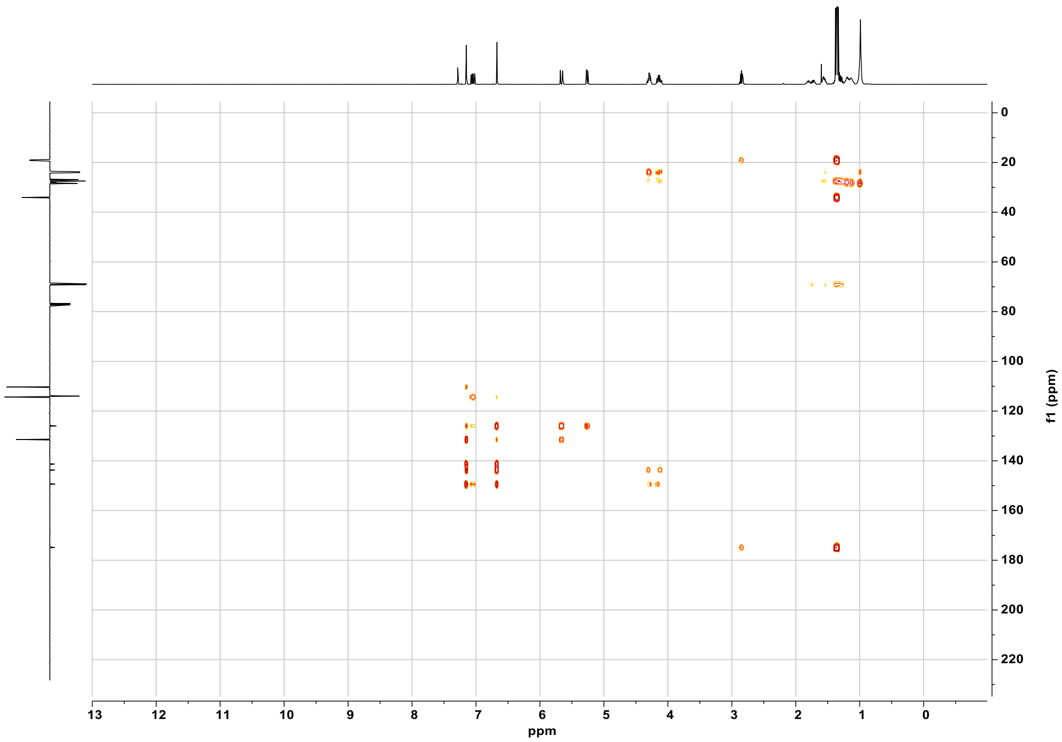

(Rp)-1⁵-(4-methoxyphenyl)-2,15-dioxa-1(1,4)-benzenacyclopentadecaphane-1²-yl isobutyrate 14

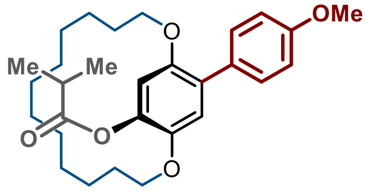

14
^{1}H, $CDCl_3$, 500 MHz

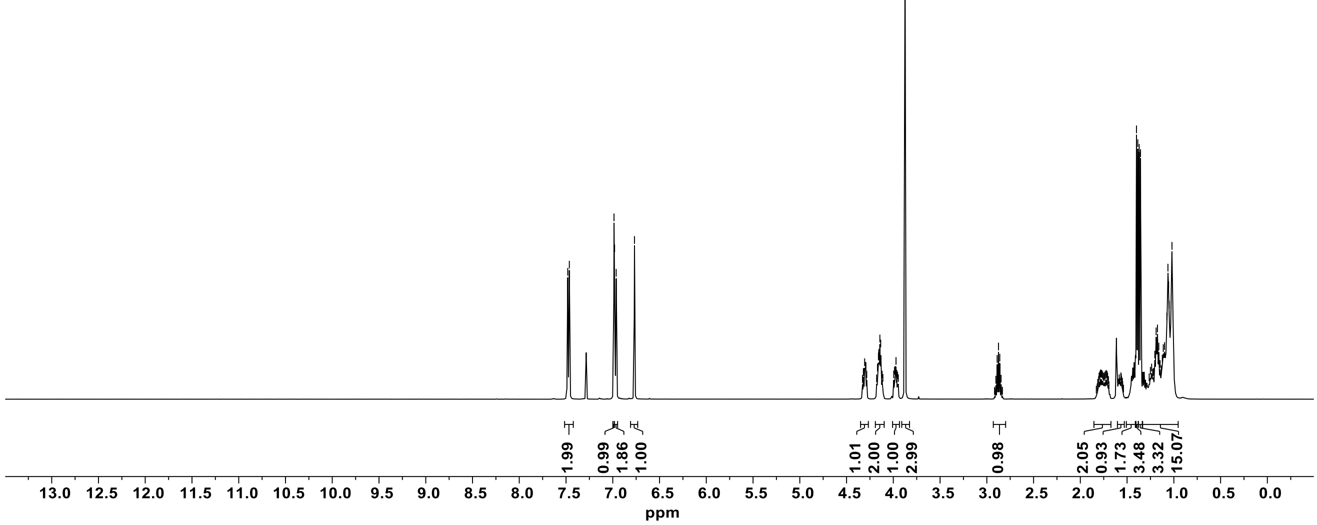

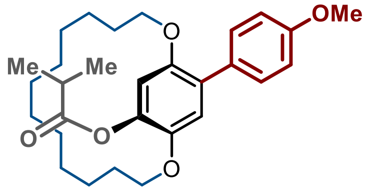

14
^{13}C{^{1}H}, $CDCl_3$, 126 MHz

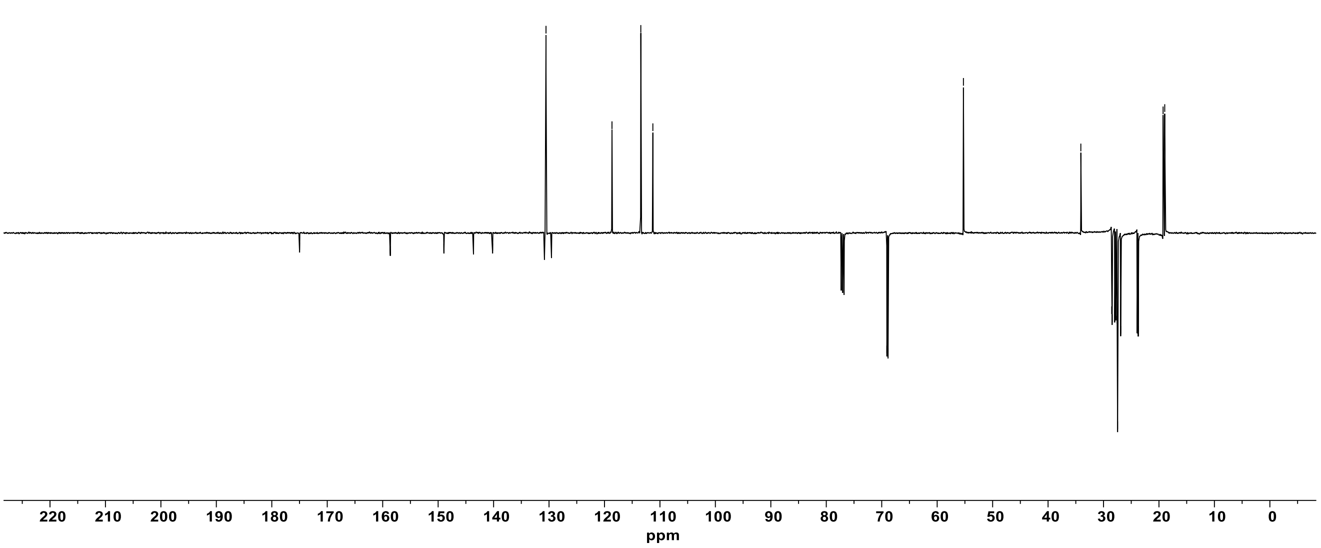

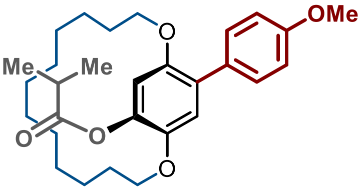

14

2D ^{1}H, $CDCl_3$, COSY

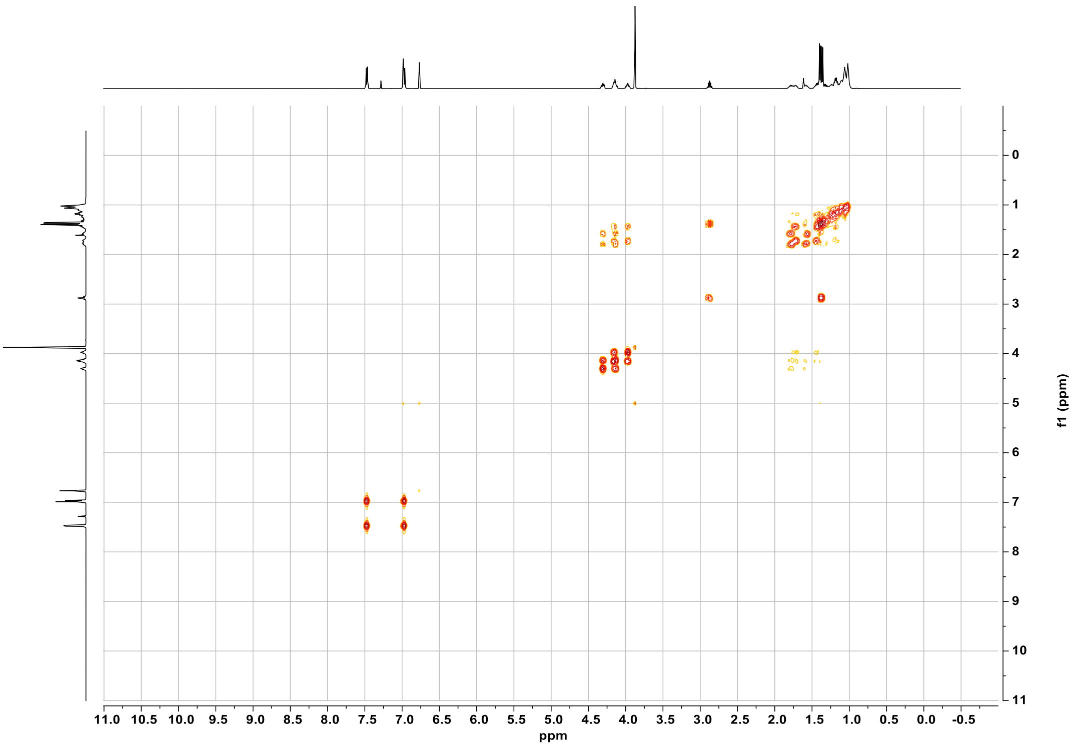

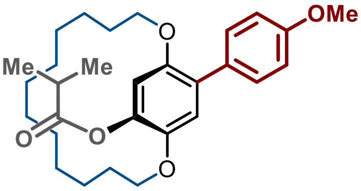

14

2D ^{1}H-^{13}C, $CDCl_3$, HSQC

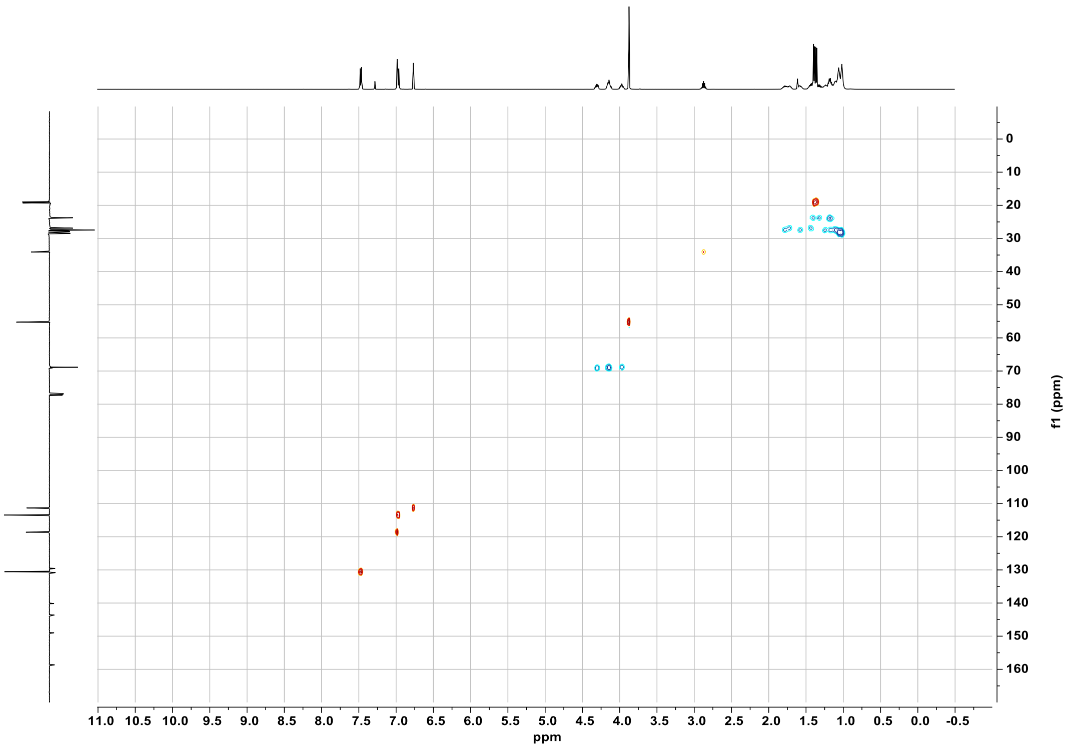

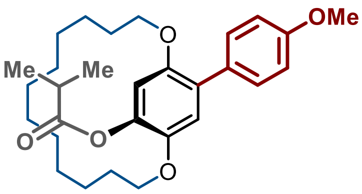

14

2D ^{1}H-^{13}C, $CDCl_3$, HMBC

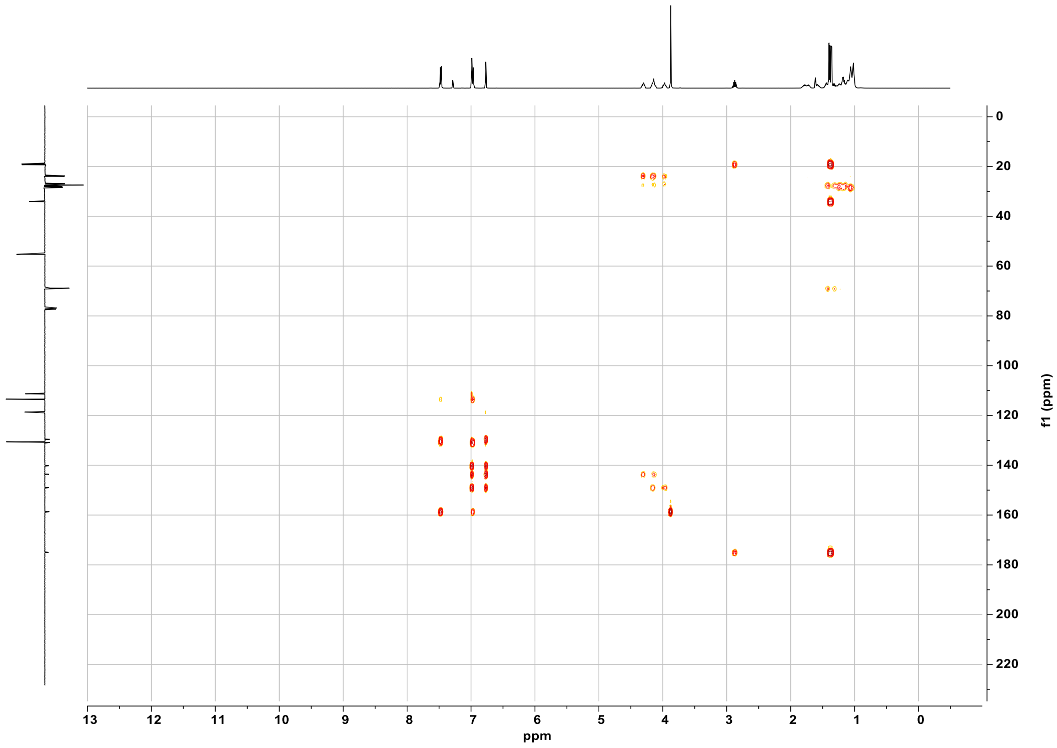

(*Rp*)-1⁵-(4-(trifluoromethyl)phenyl)-2,15-dioxa-1(1,4)-benzenacyclopentadecaphane-1²-yl isobutyrate 15

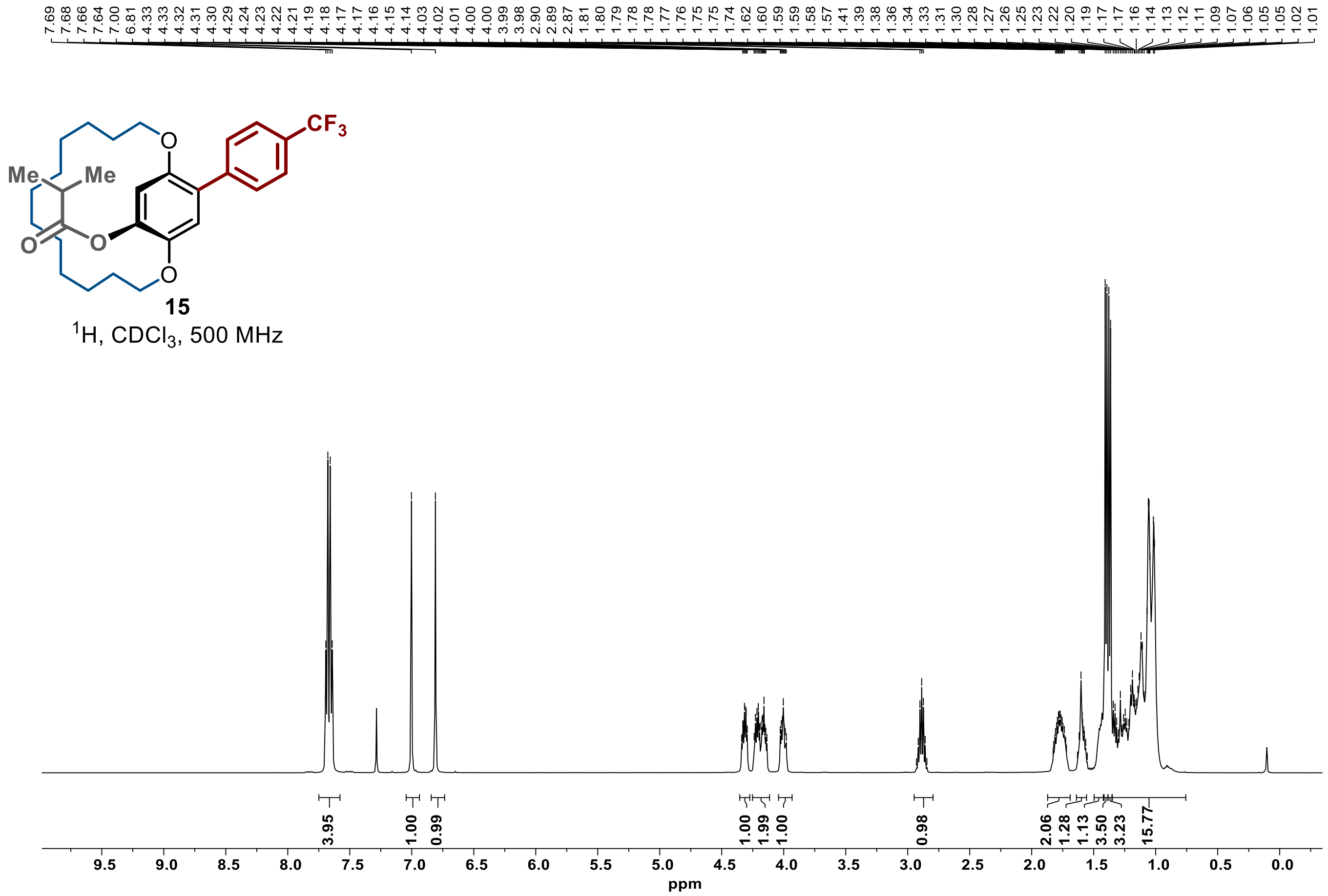

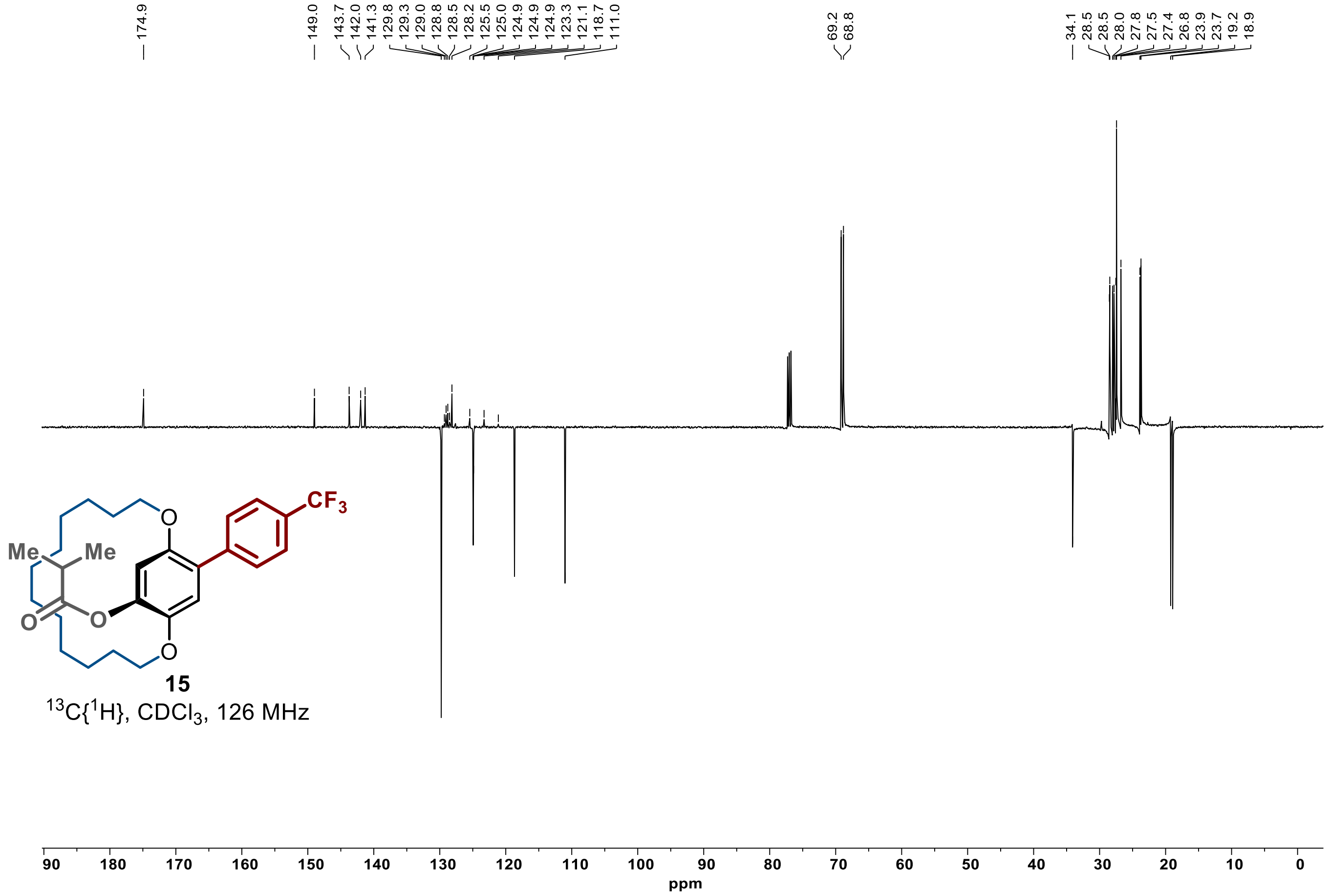

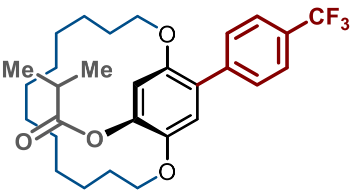

15

2D ^{1}H, $CDCl_3$, COSY

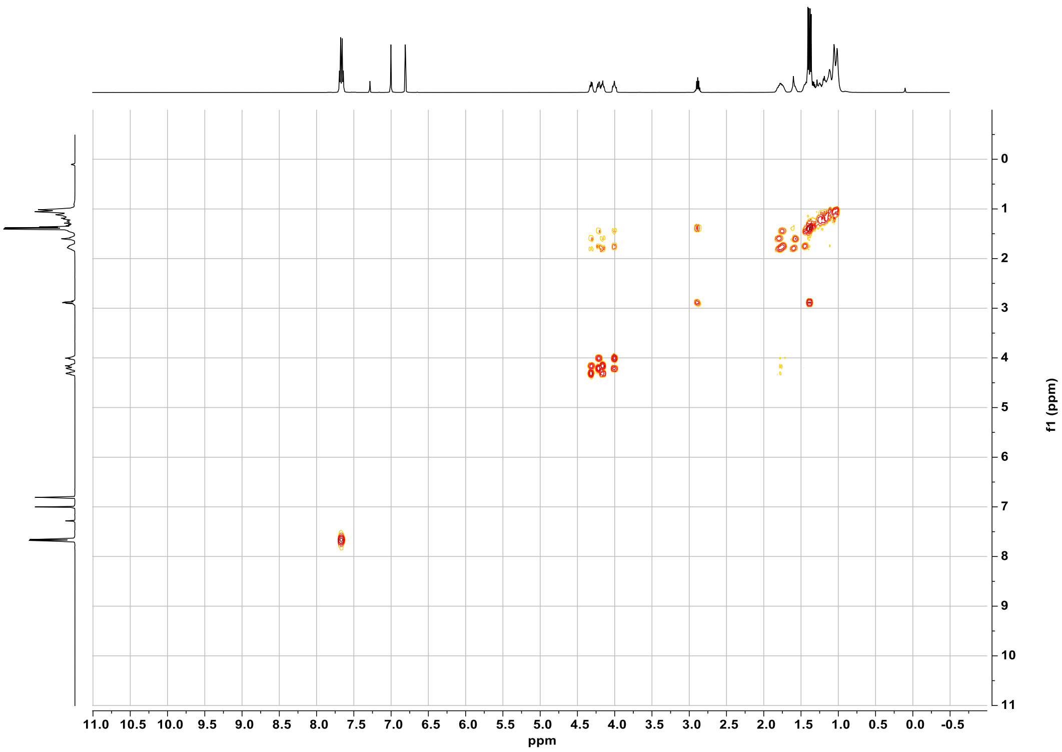

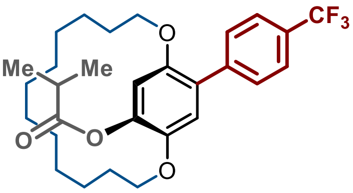

15

2D ^{1}H-^{13}C, $CDCl_3$, HSQC

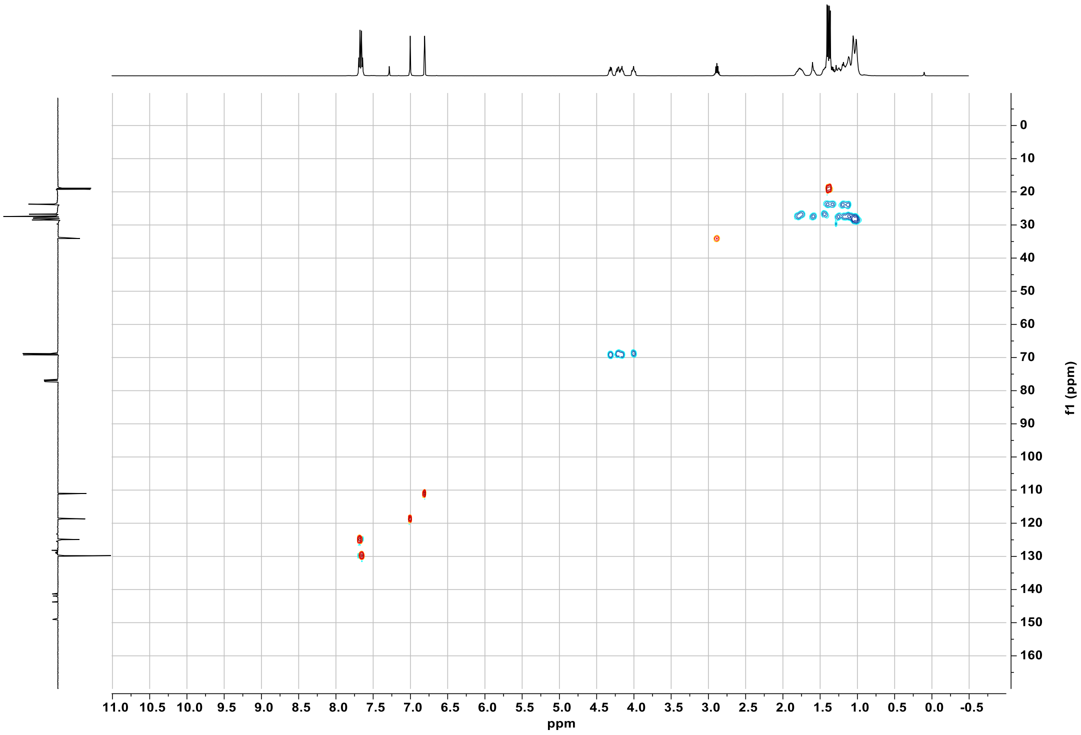

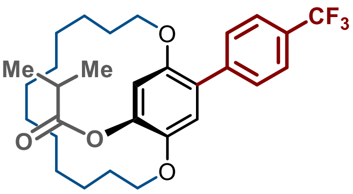

15

2D ^1H-^{13}C, $CDCl_3$, HMBC

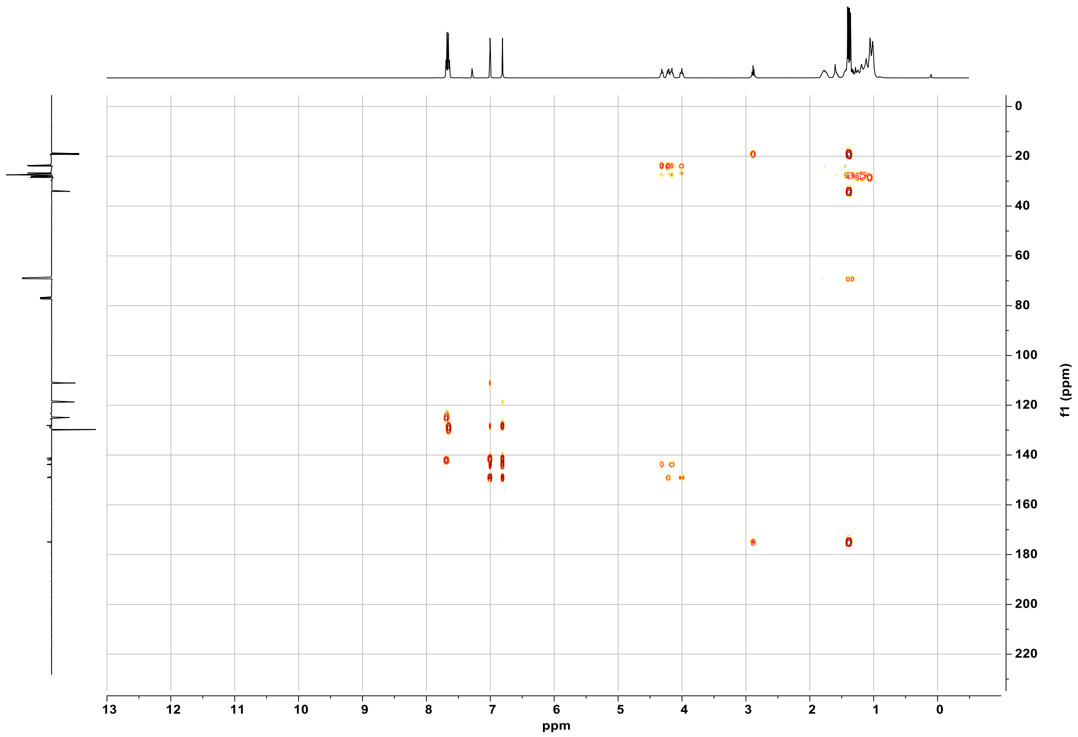

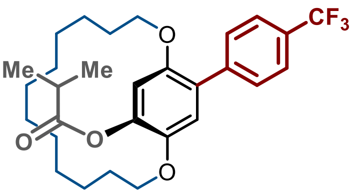

15

$^{19}F\{^1H\}$, $CDCl_3$, 470 MHz

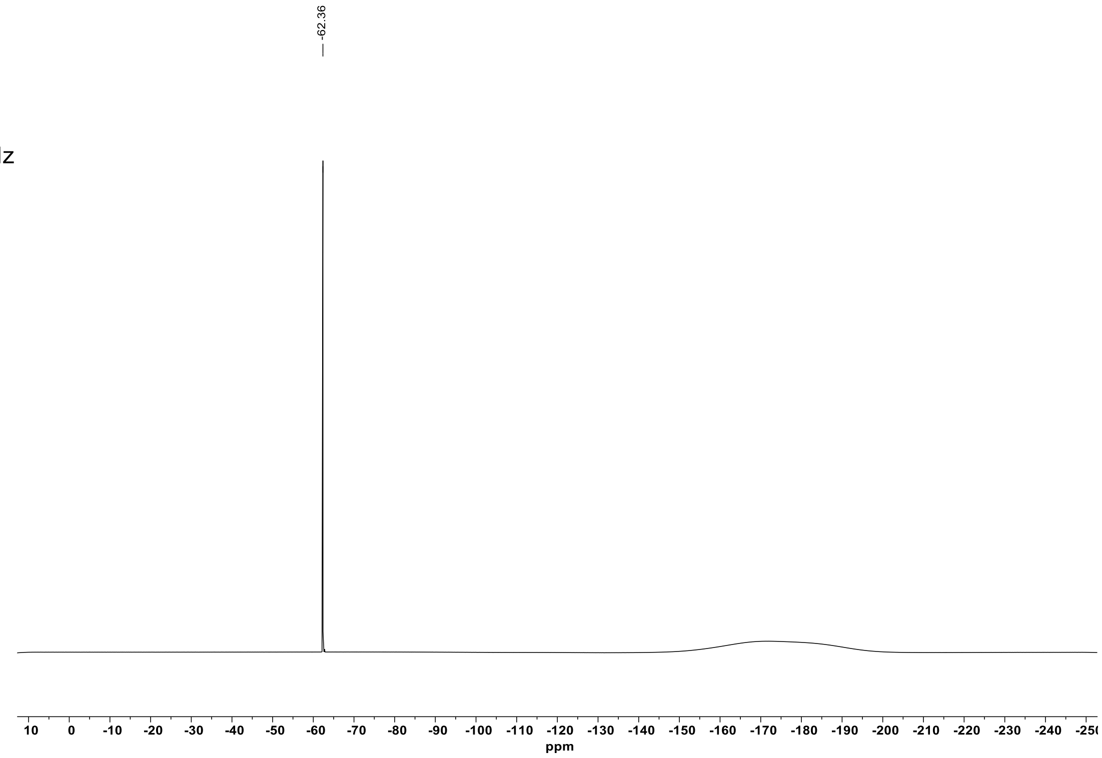

(*Rp*)-1⁵-(2-methoxyphenyl)-2,15-dioxa-1(1,4)-benzenacyclopentadecaphane-1²-yl isobutyrate 16

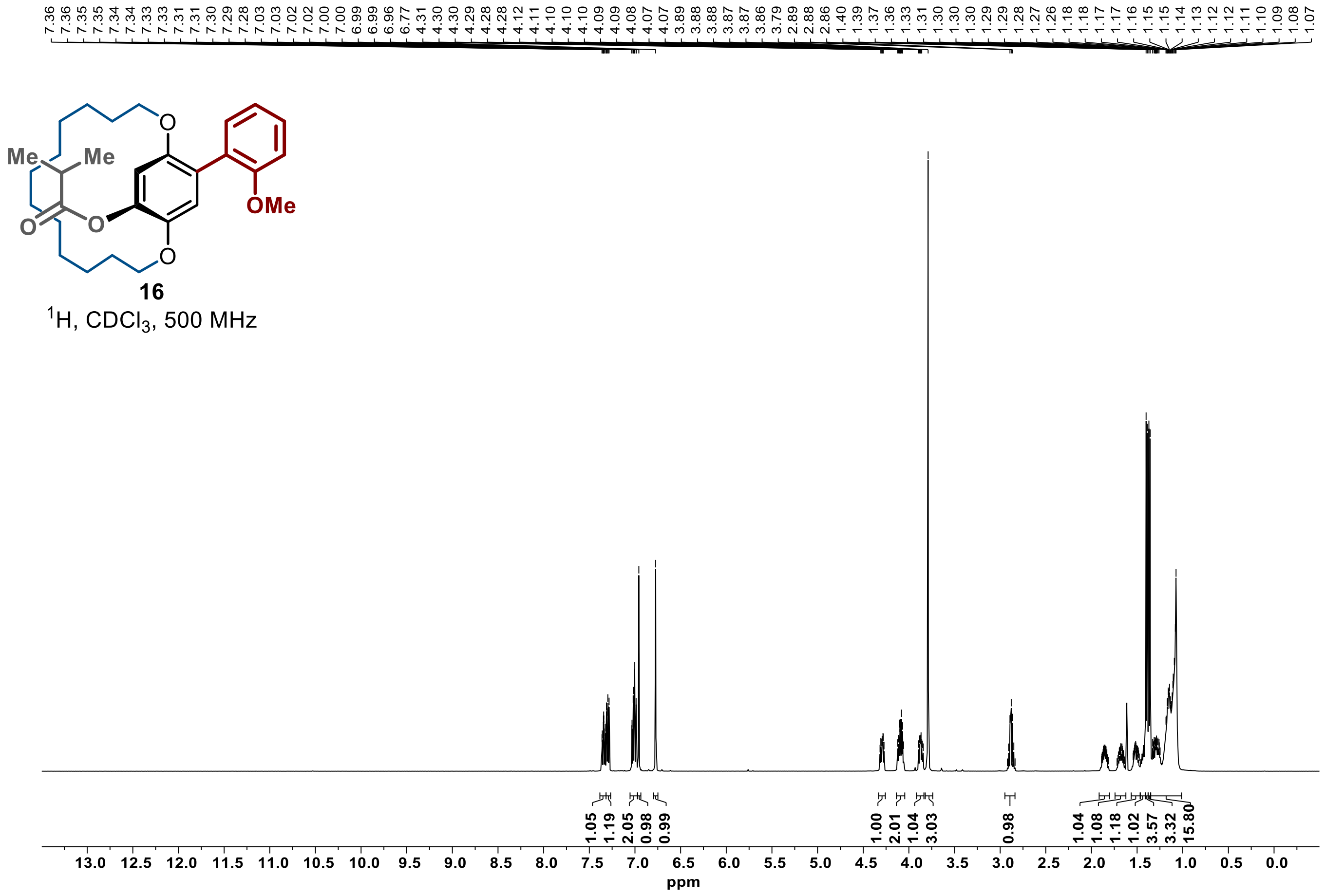

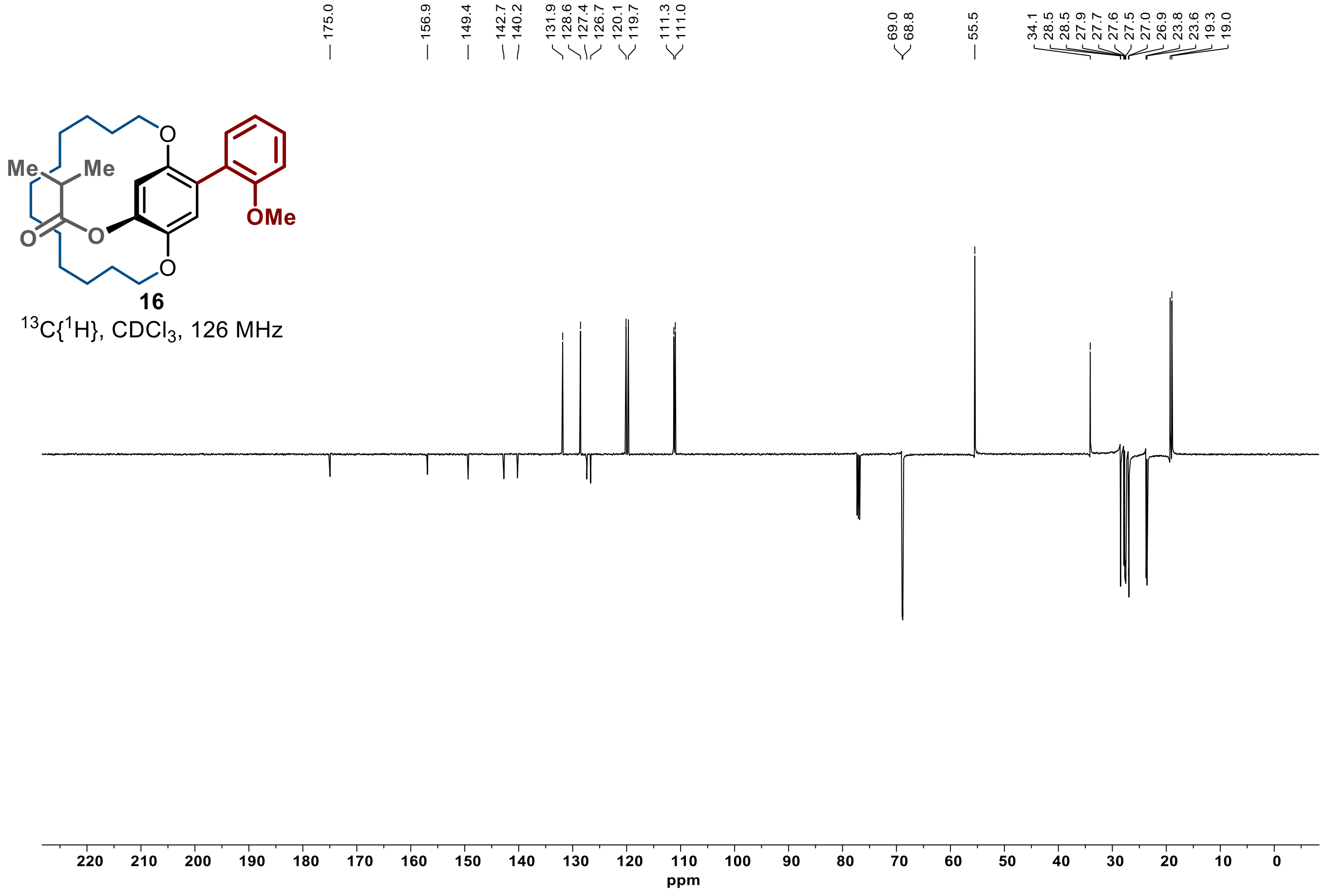

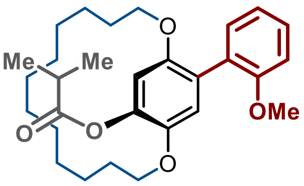

16

2D ^{1}H, $CDCl_3$, COSY

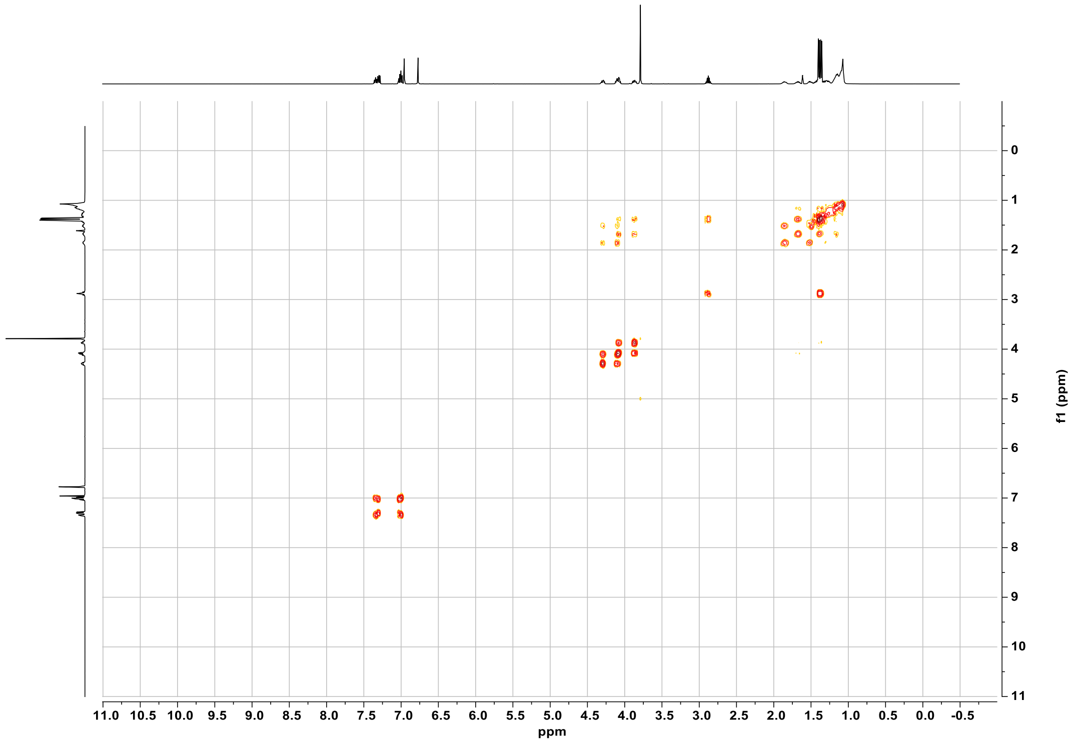

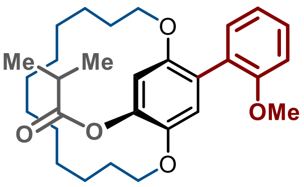

16

2D ^{1}H-^{13}C, $CDCl_3$, HSQC

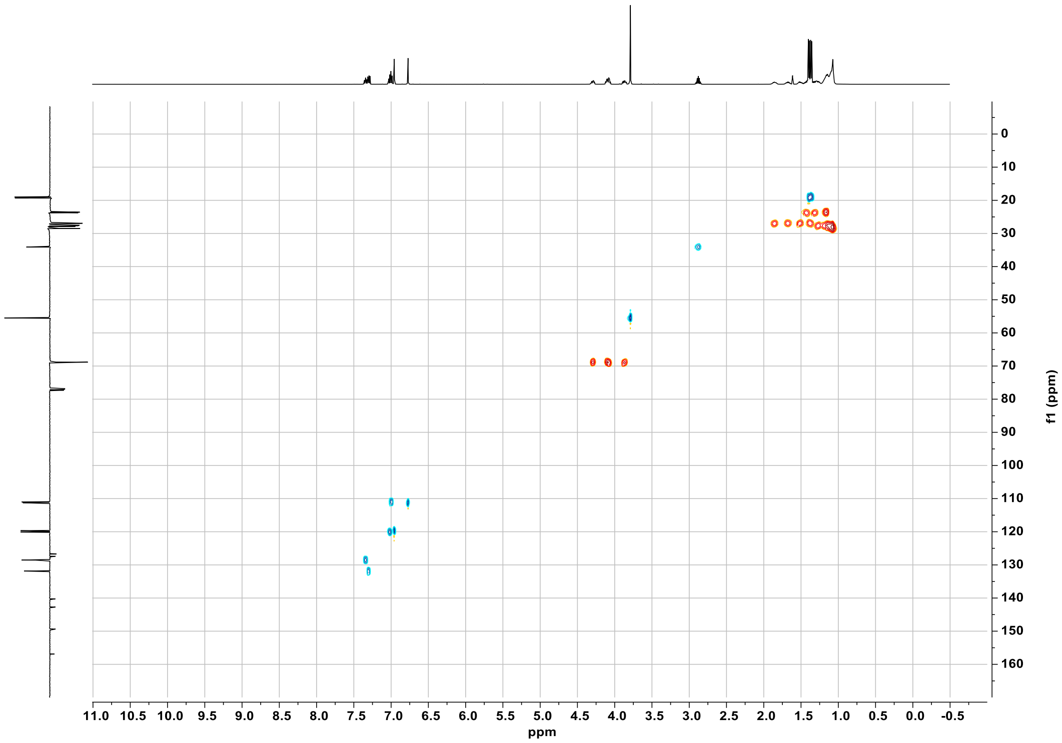

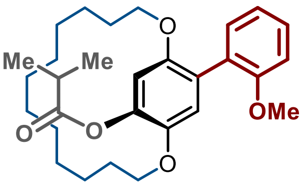

16

2D ^{1}H-^{13}C, $CDCl_3$, HMBC

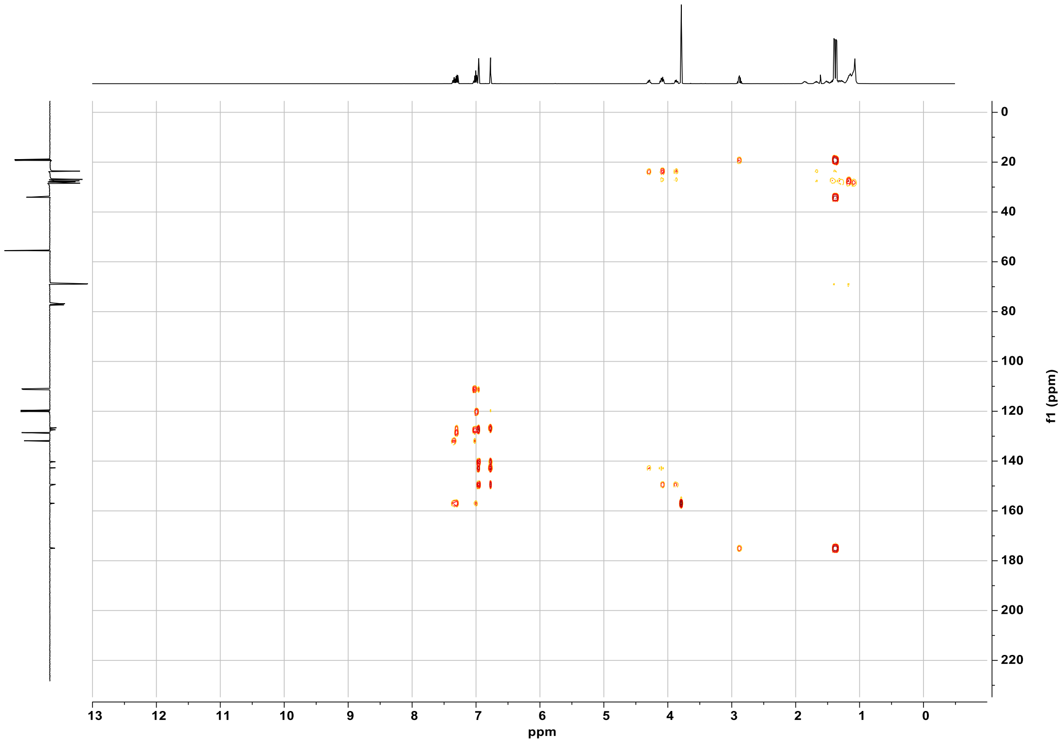

(*Rp*)-1⁵-(naphthalen-1-yl)-2,15-dioxa-1(1,4)-benzenacyclopentadecaphane-1²-yl isobutyrate 17

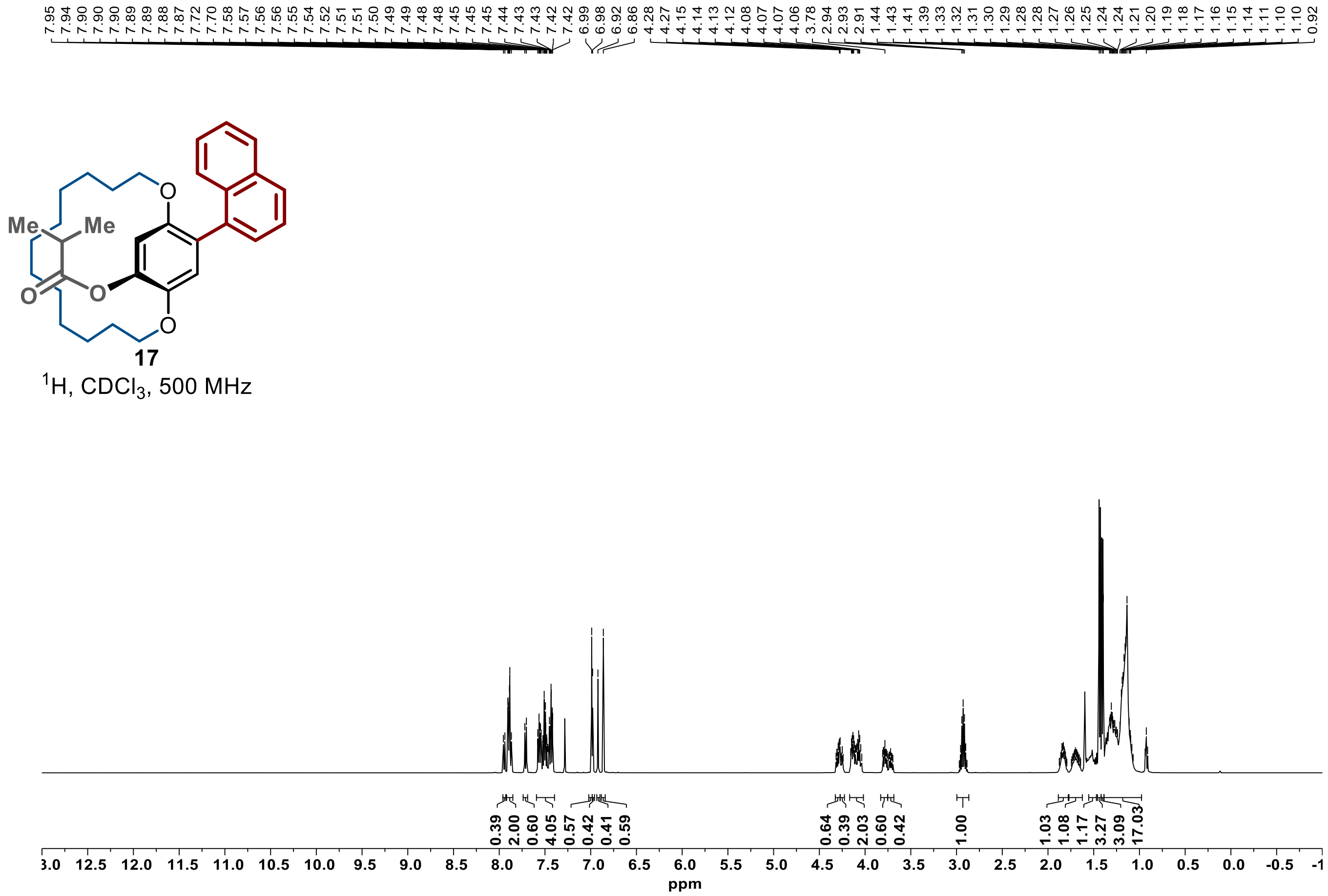

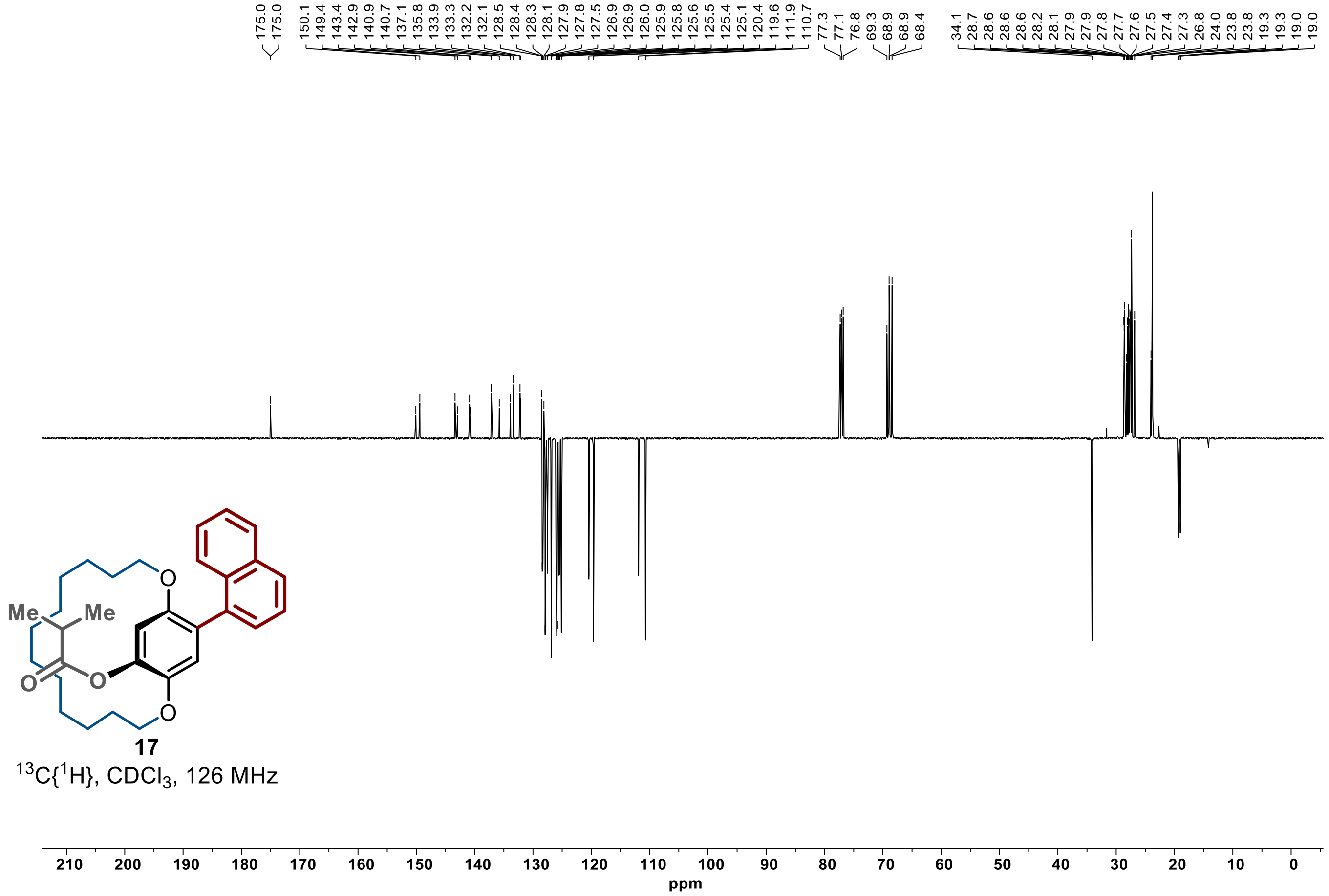

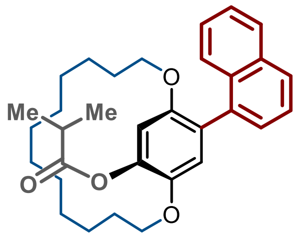

17

2D ^{1}H, $CDCl_3$, COSY

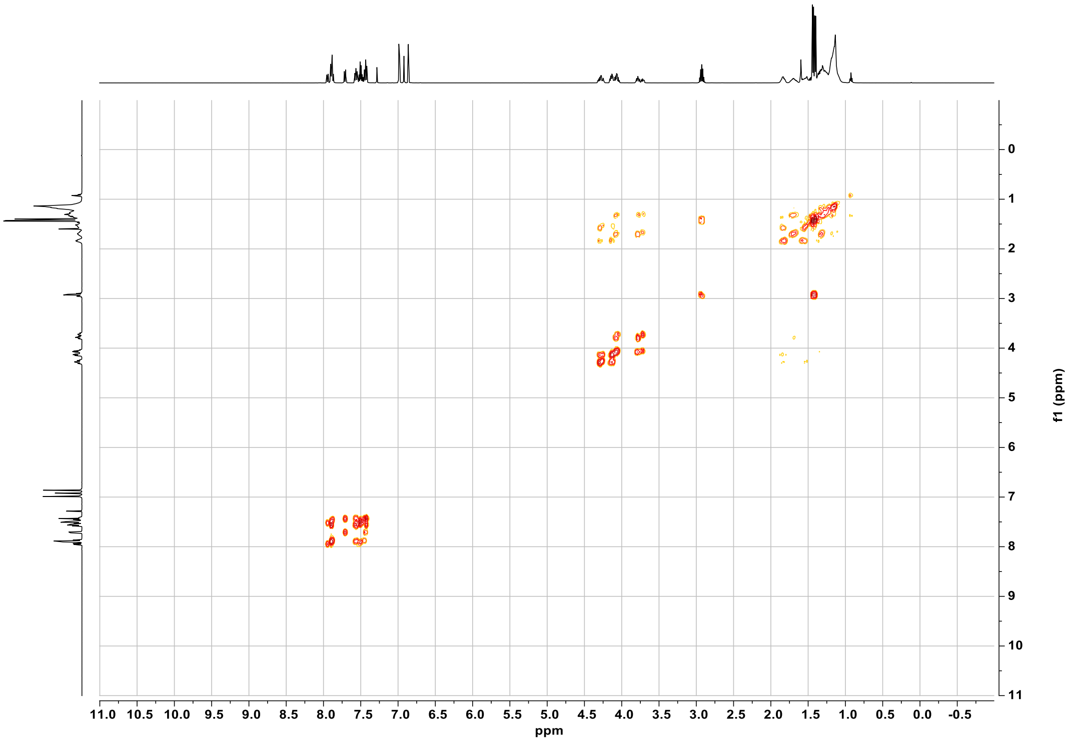

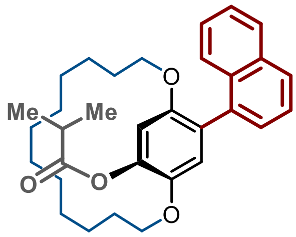

17

2D ^{1}H-^{13}C, $CDCl_3$, HSQC

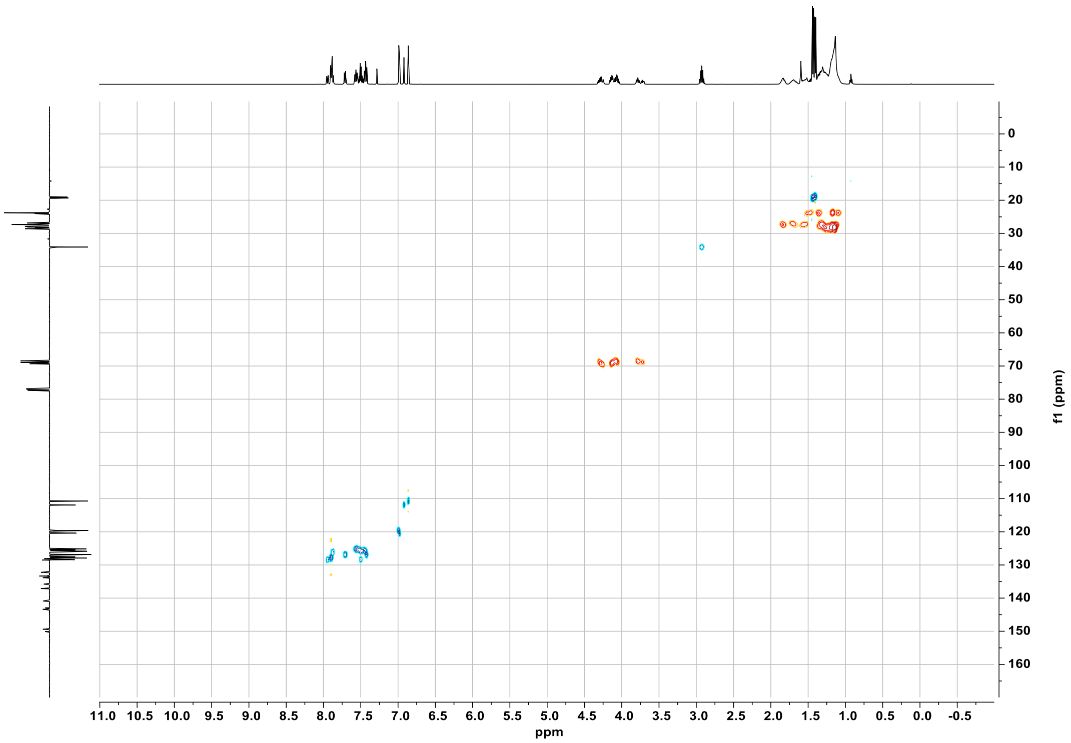

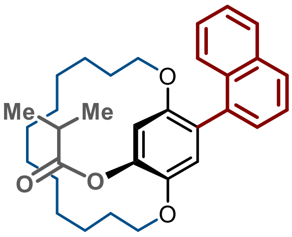

17

2D ^{1}H-^{13}C, $CDCl_3$, HMBC

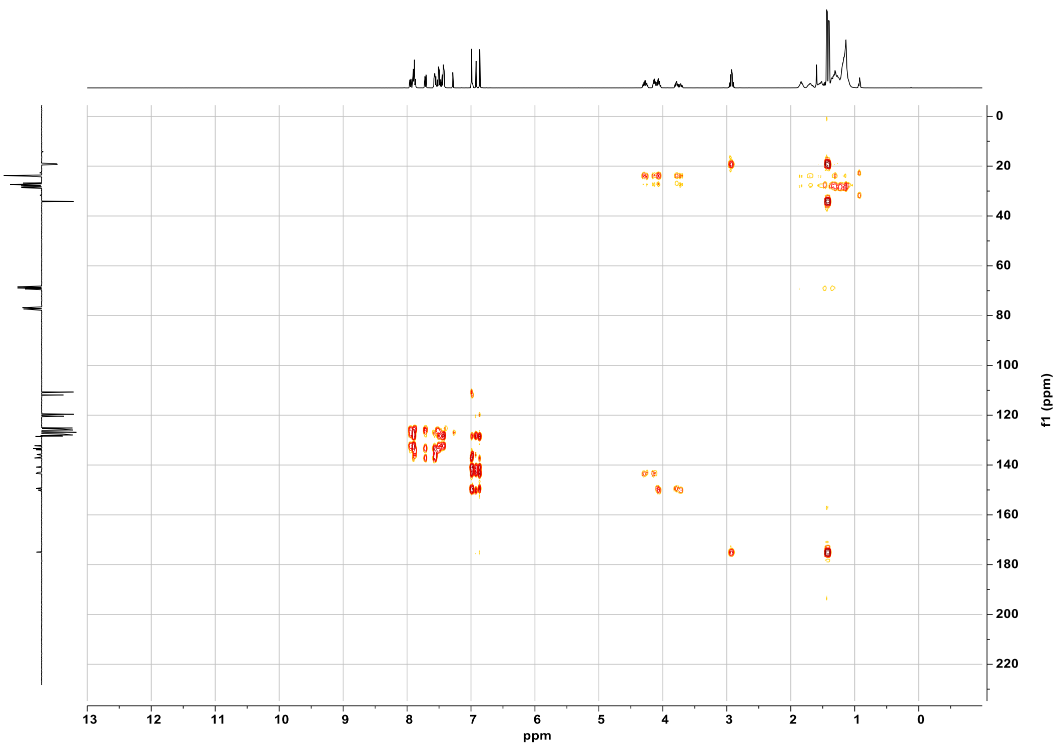

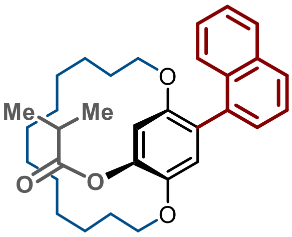

17

2D ^{1}H, $CDCl_3$, NOESY

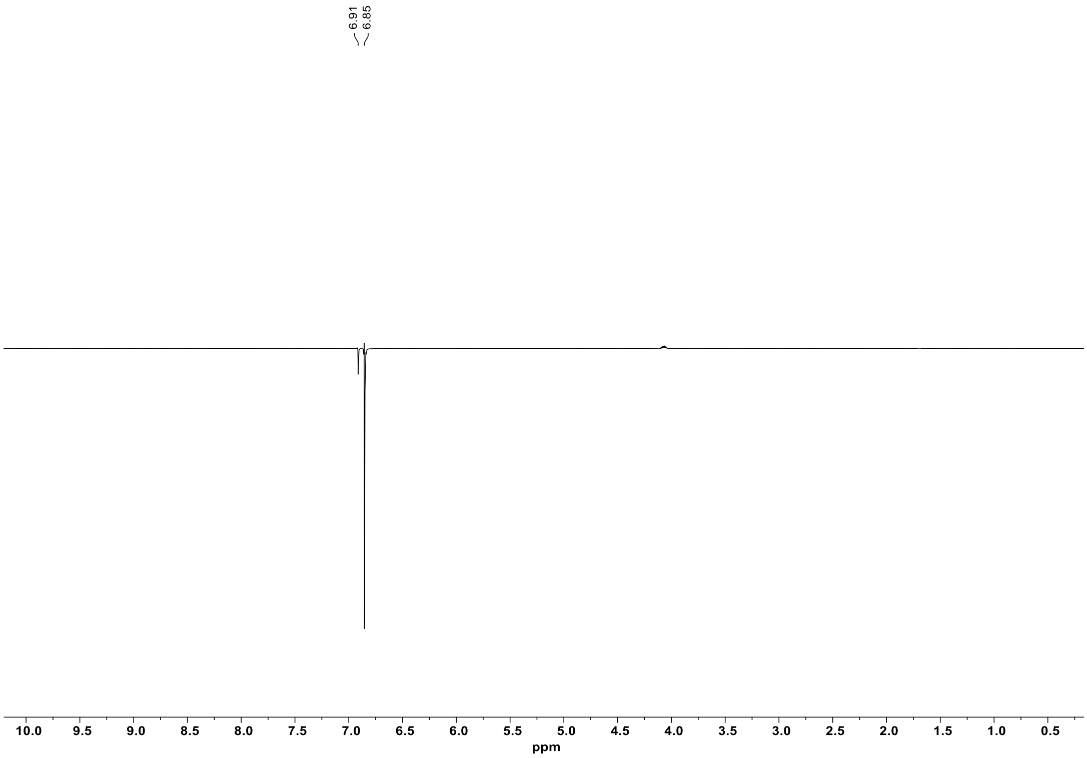

(*Rp*)-1⁵-(naphthalen-2-yl)-2,15-dioxa-1(1,4)-benzenacyclopentadecaphane-1²-yl isobutyrate 18

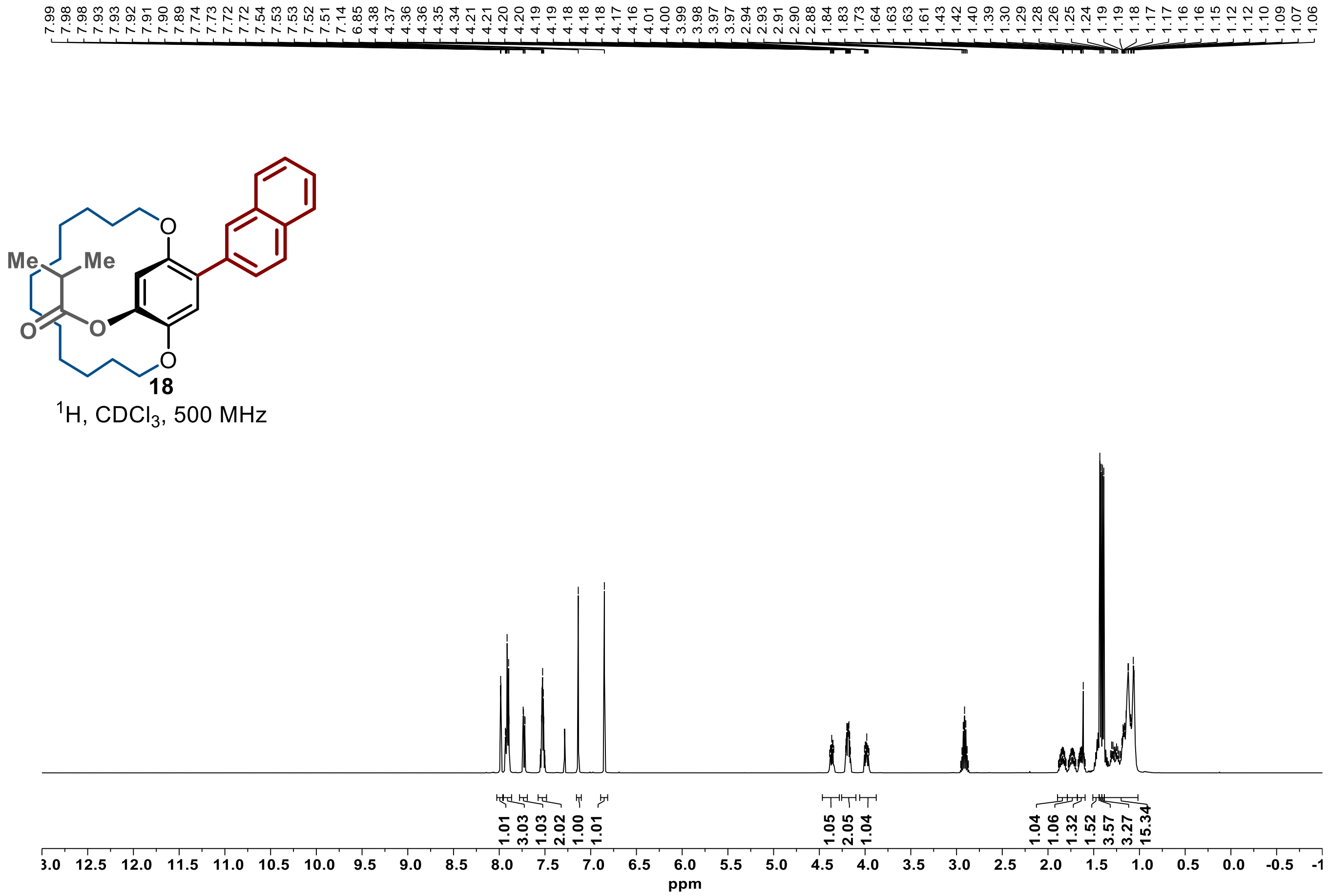

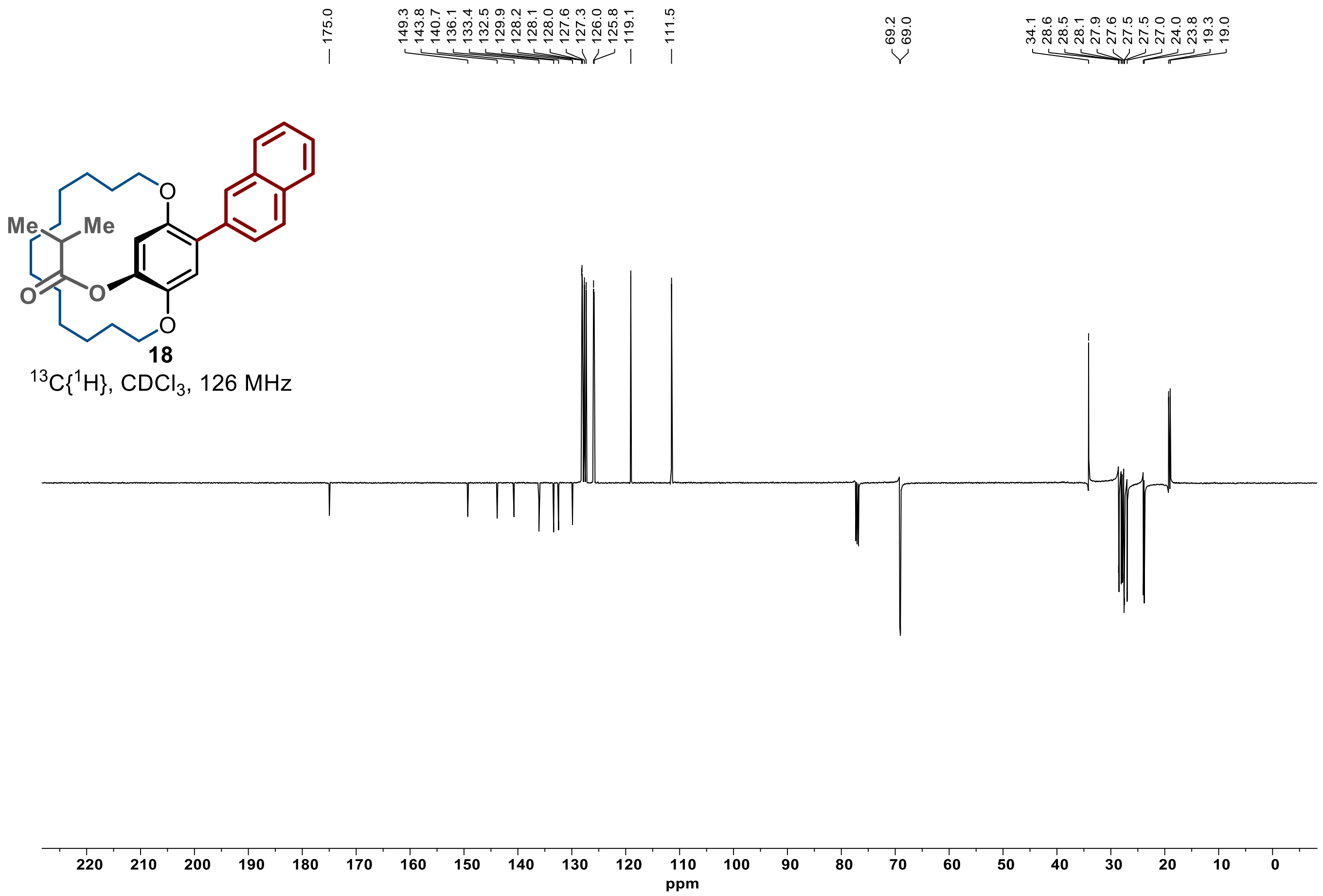

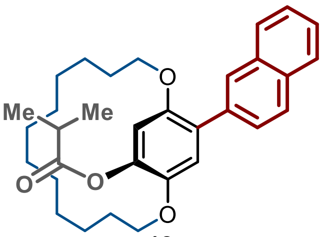

18

2D ^{1}H, $CDCl_3$, COSY

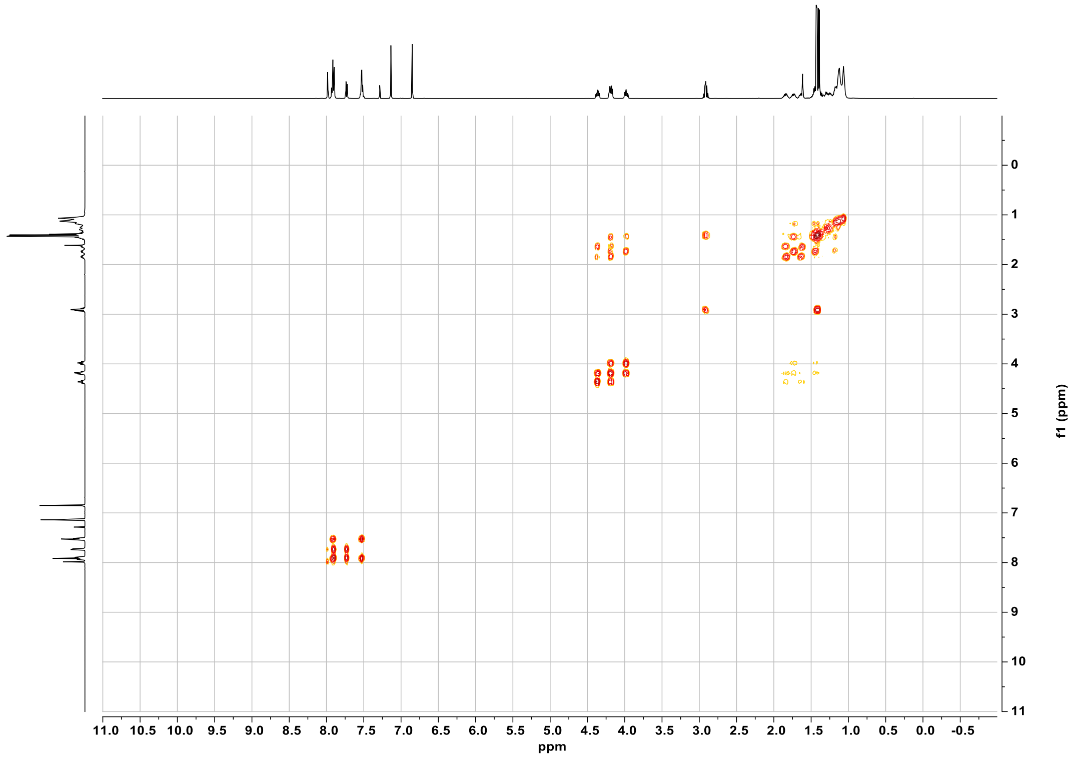

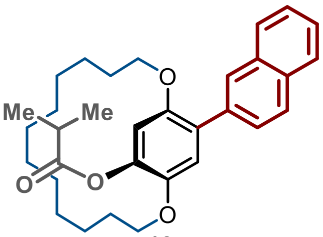

18

2D ^{1}H-^{13}C, $CDCl_3$, HSQC

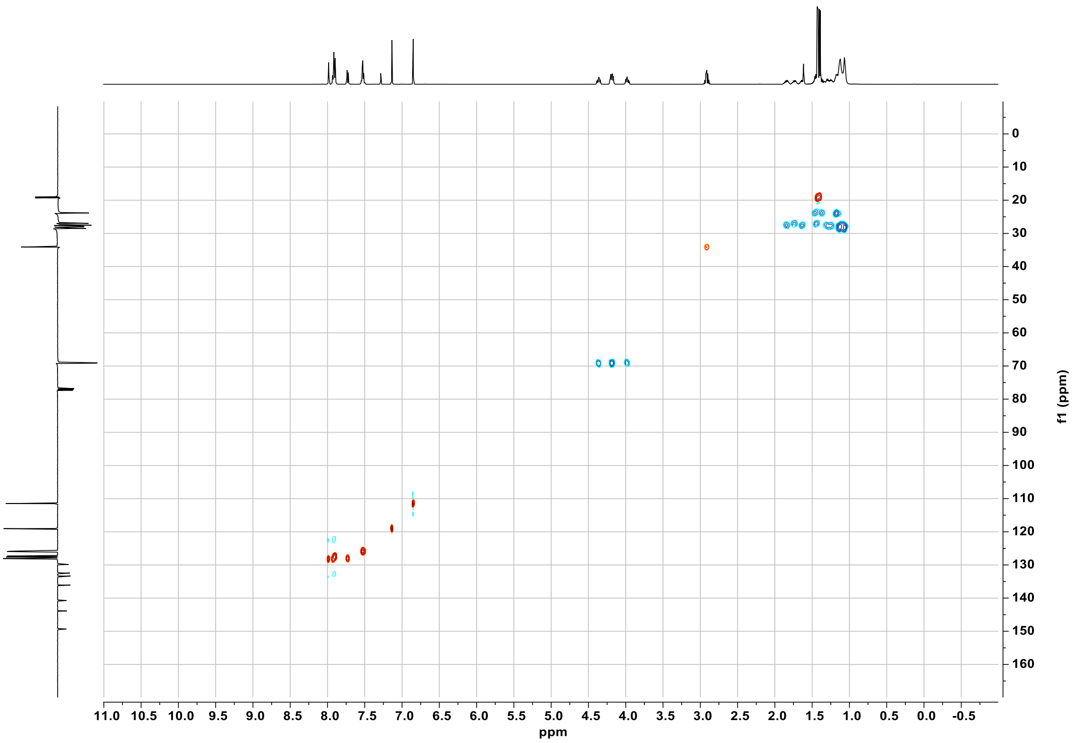

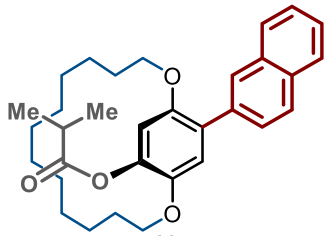

18

2D ^{1}H-^{13}C, $CDCl_3$, HMBC

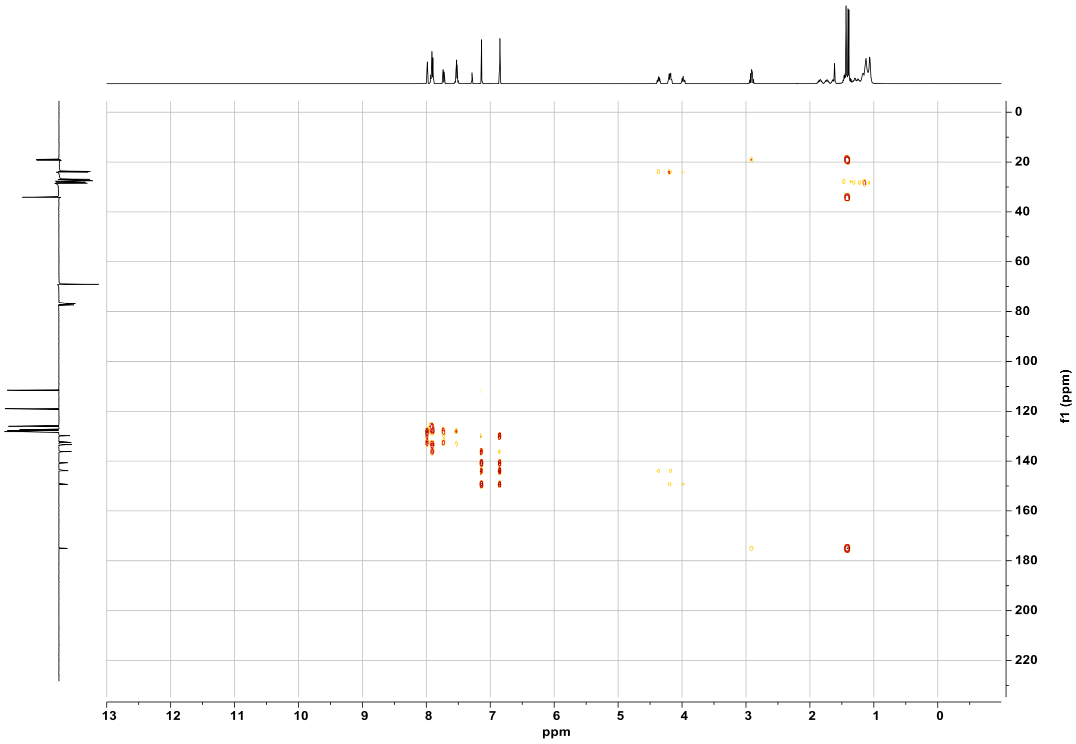

(*Rp*)-1⁵-(pyridin-3-yl)-2,15-dioxa-1(1,4)-benzenacyclopentadecaphane-1²-yl isobutyrate 19

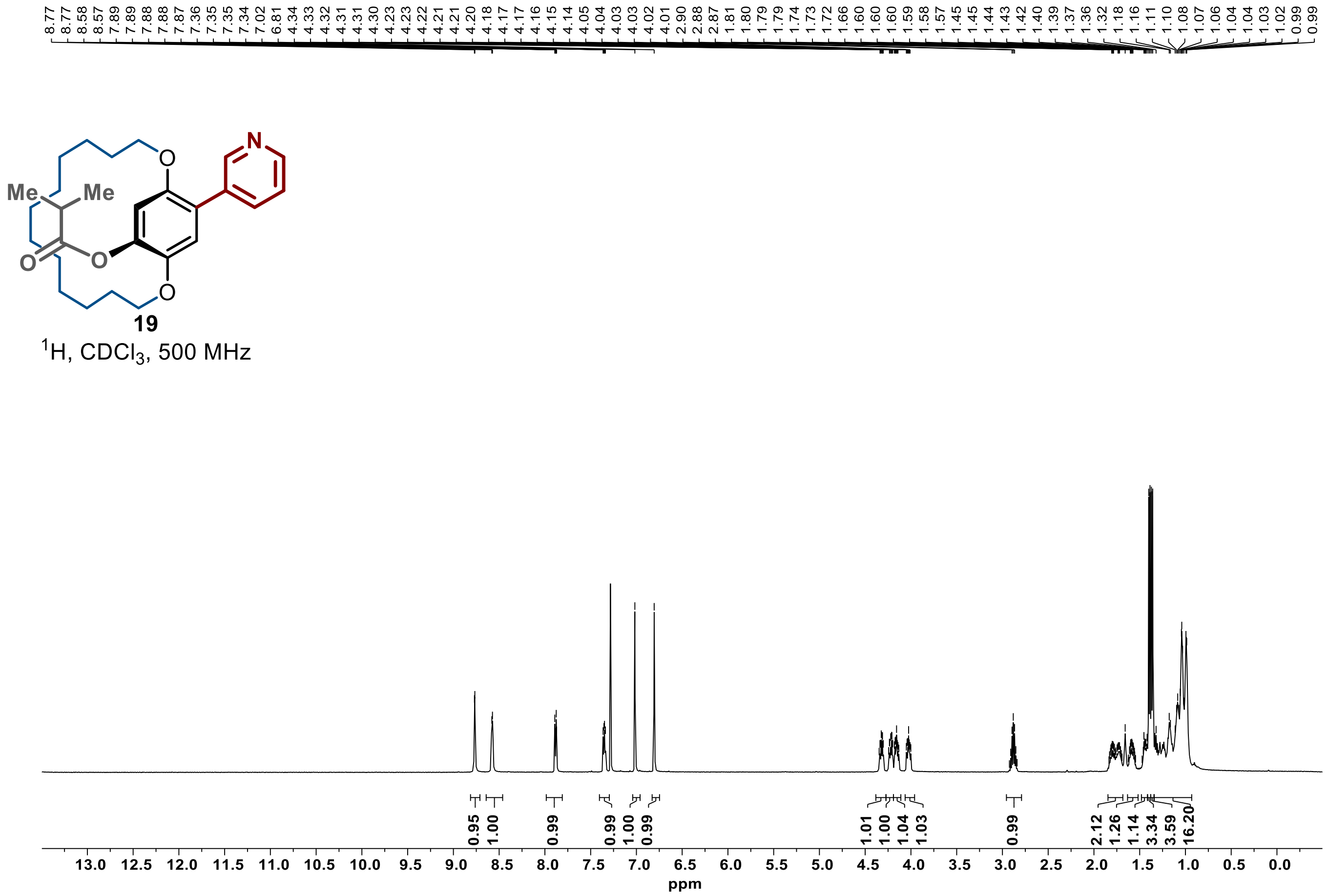

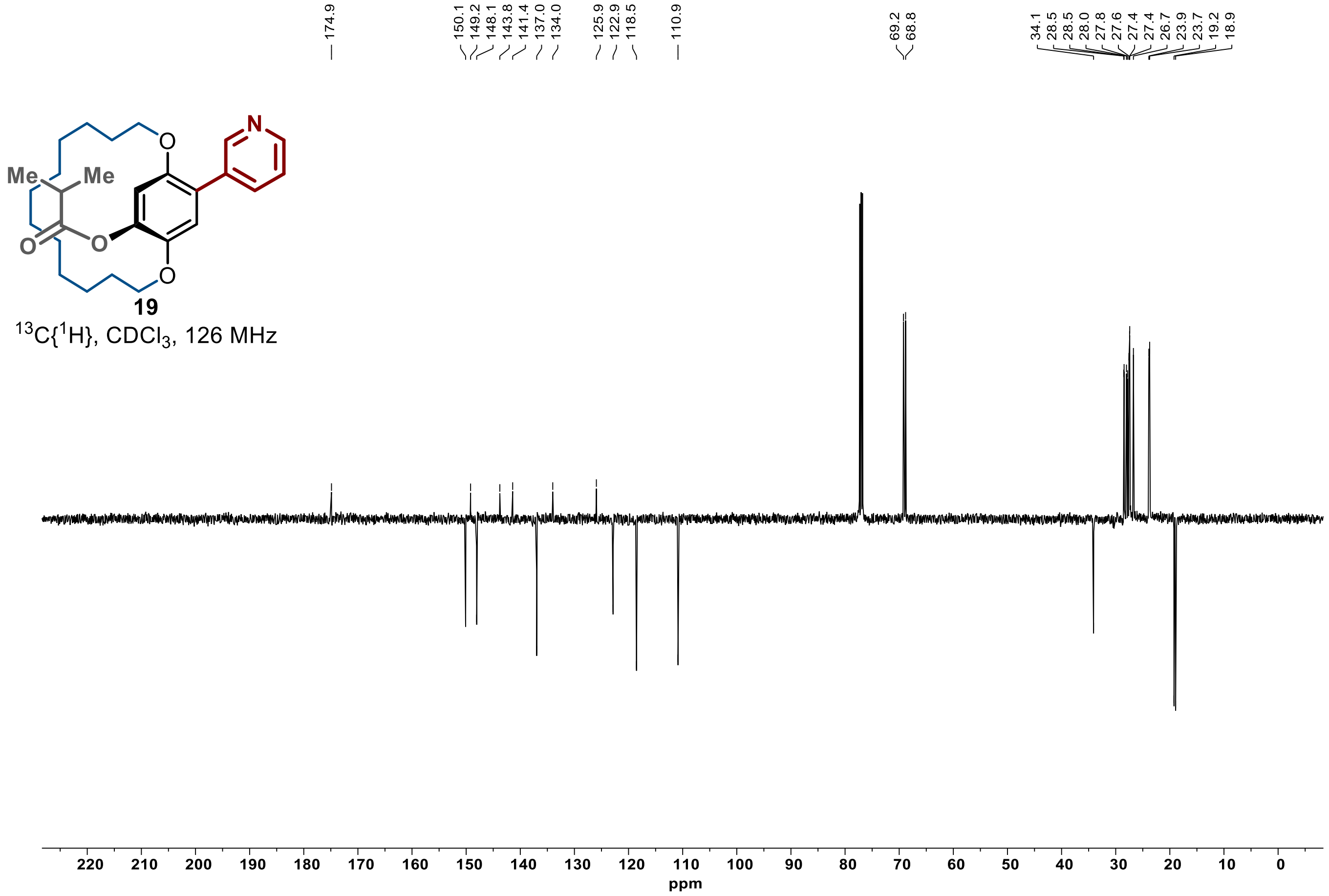

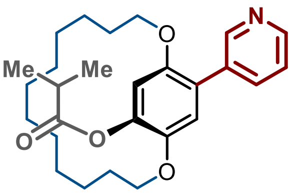

19

2D ^{1}H, $CDCl_3$, COSY

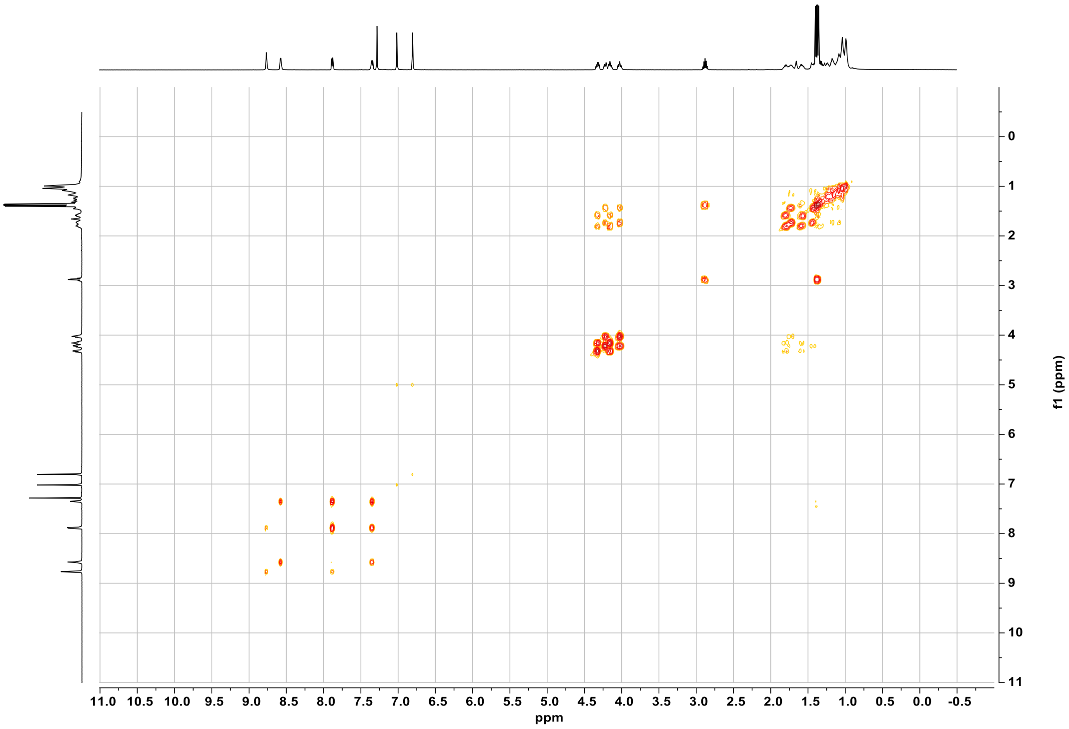

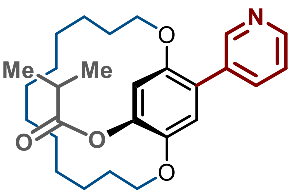

19

2D ^{1}H-^{13}C, $CDCl_3$, HSQC

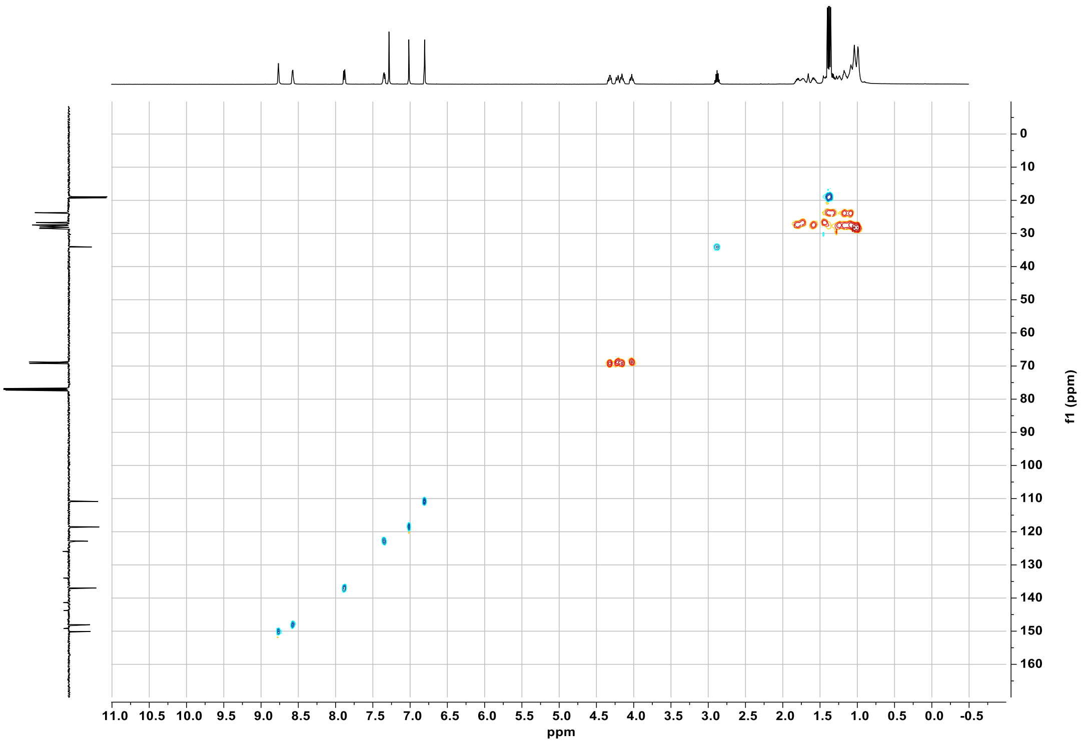

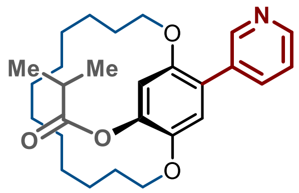

19

2D ^{1}H-^{13}C, $CDCl_3$, HMBC

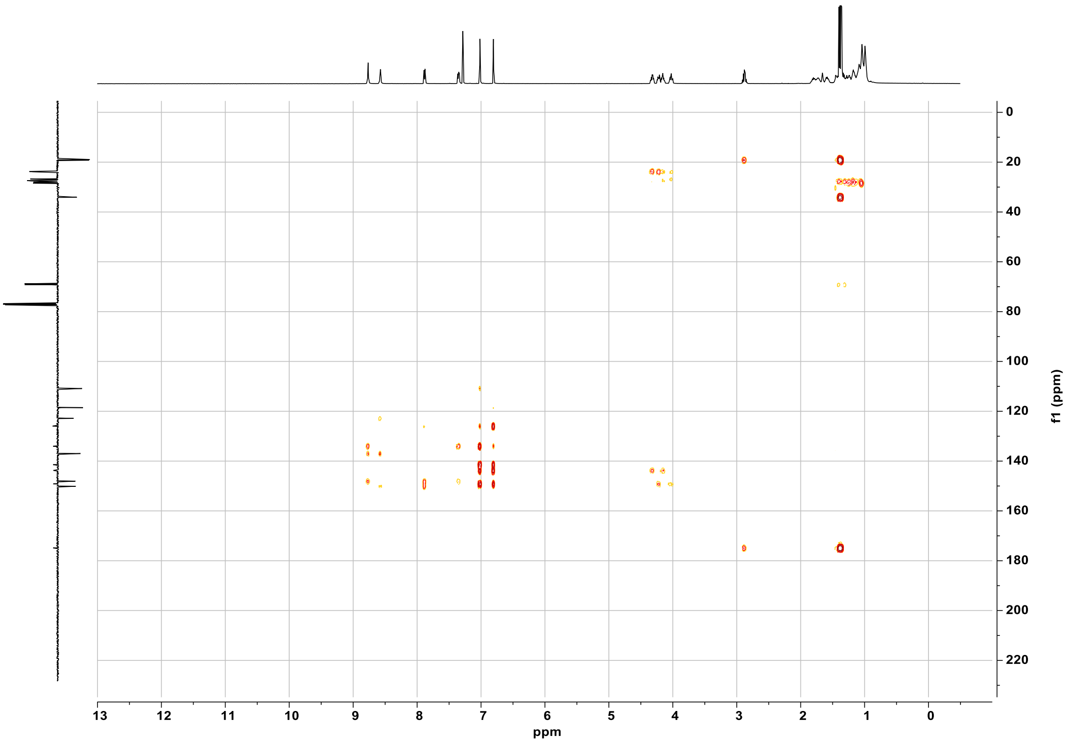

(*Rp*)-1^5-(furan-2-yl)-2,15-dioxa-1(1,4)-benzenacyclopentadecaphane-1^2-yl isobutyrate 20

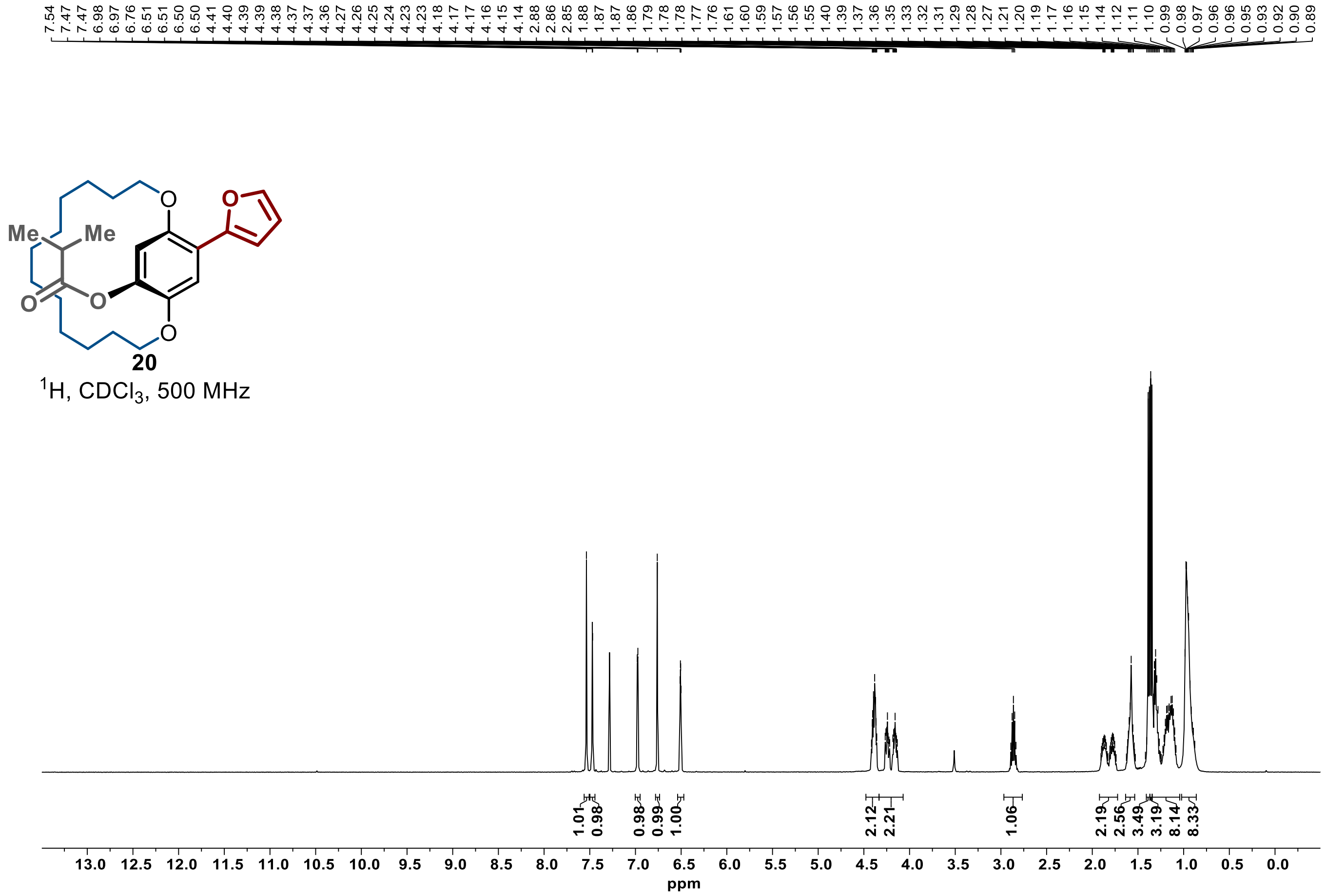

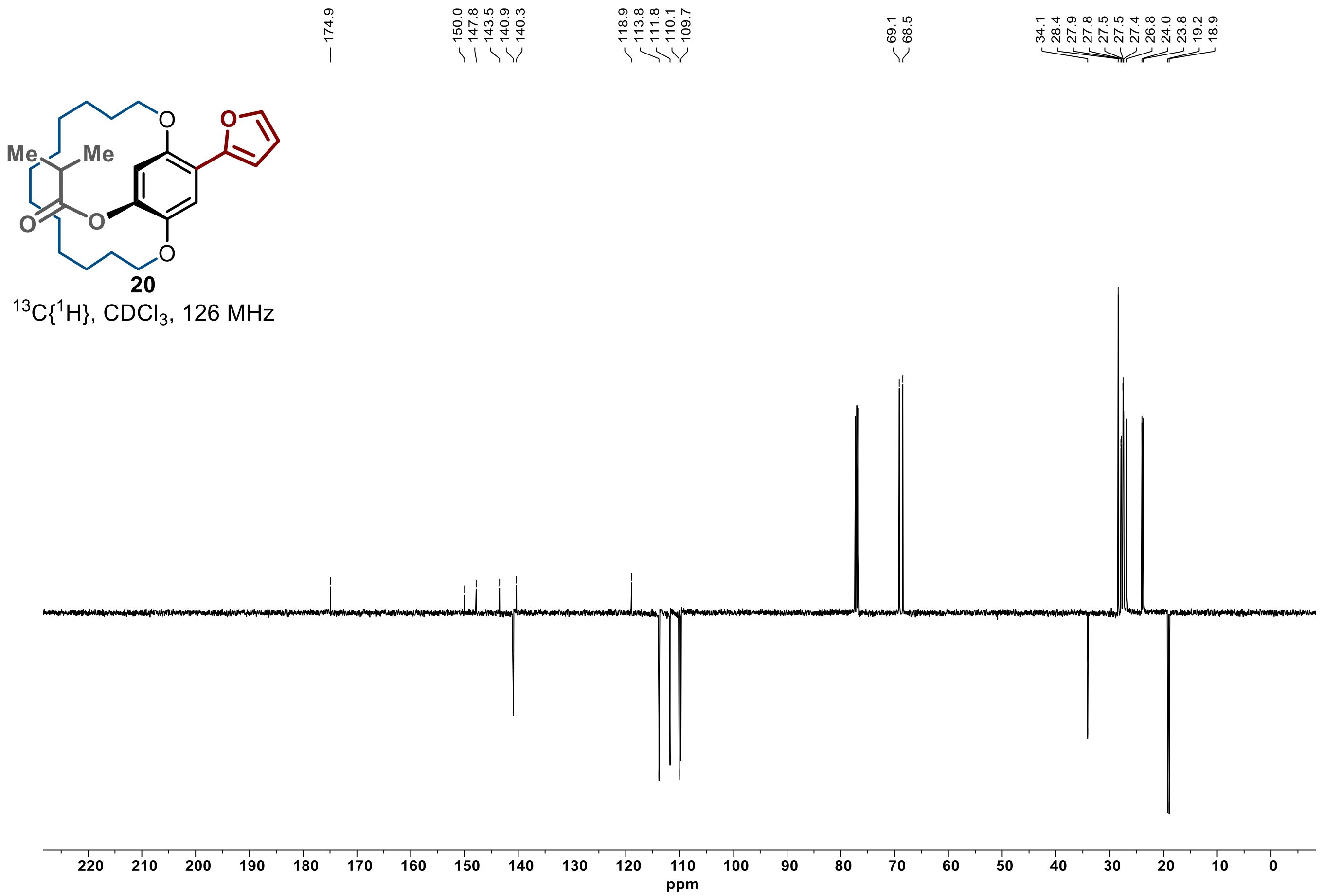

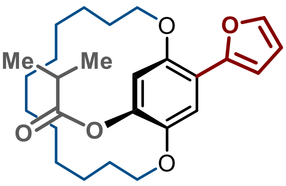

20

2D ^{1}H, $CDCl_3$, COSY

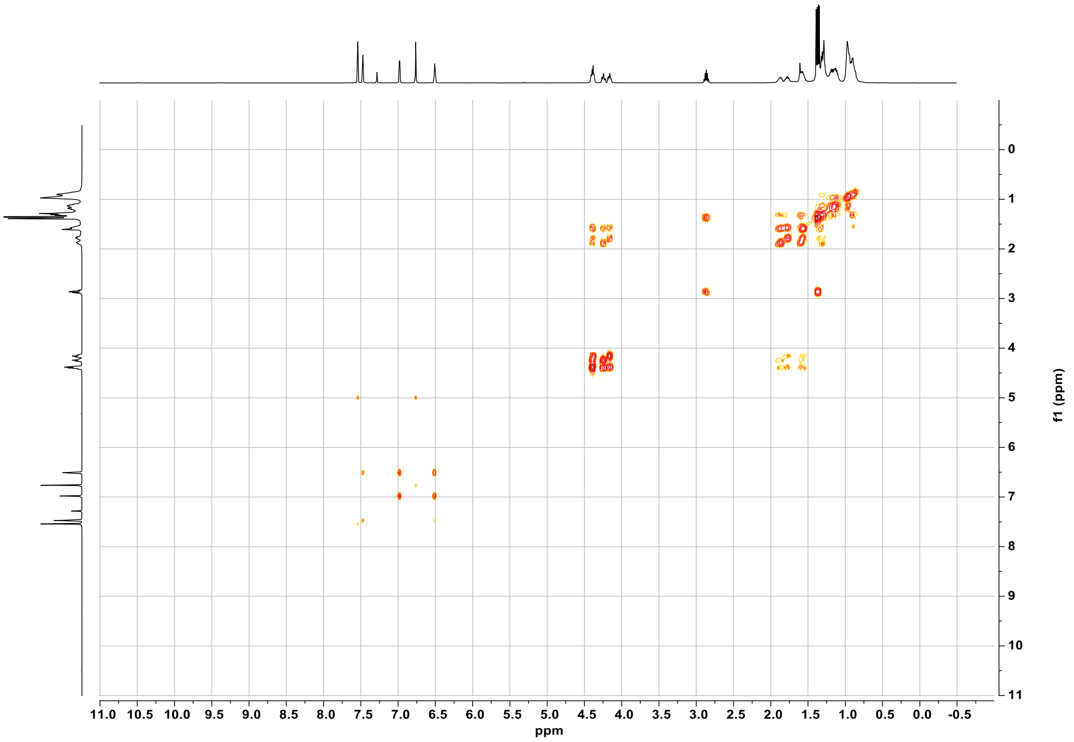

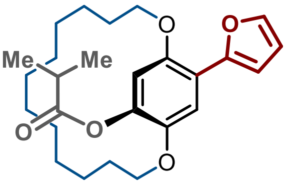

20

2D ^{1}H-^{13}C, $CDCl_3$, HSQC

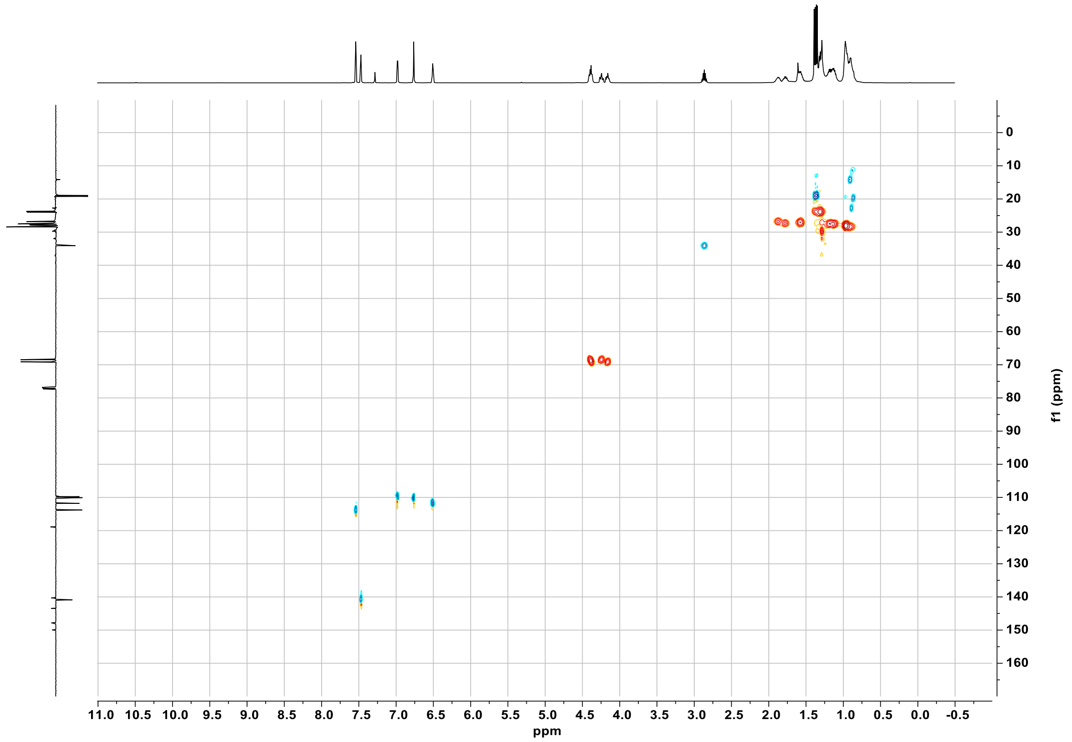

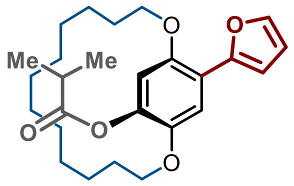

20

2D ^{1}H-^{13}C, $CDCl_3$, HMBC

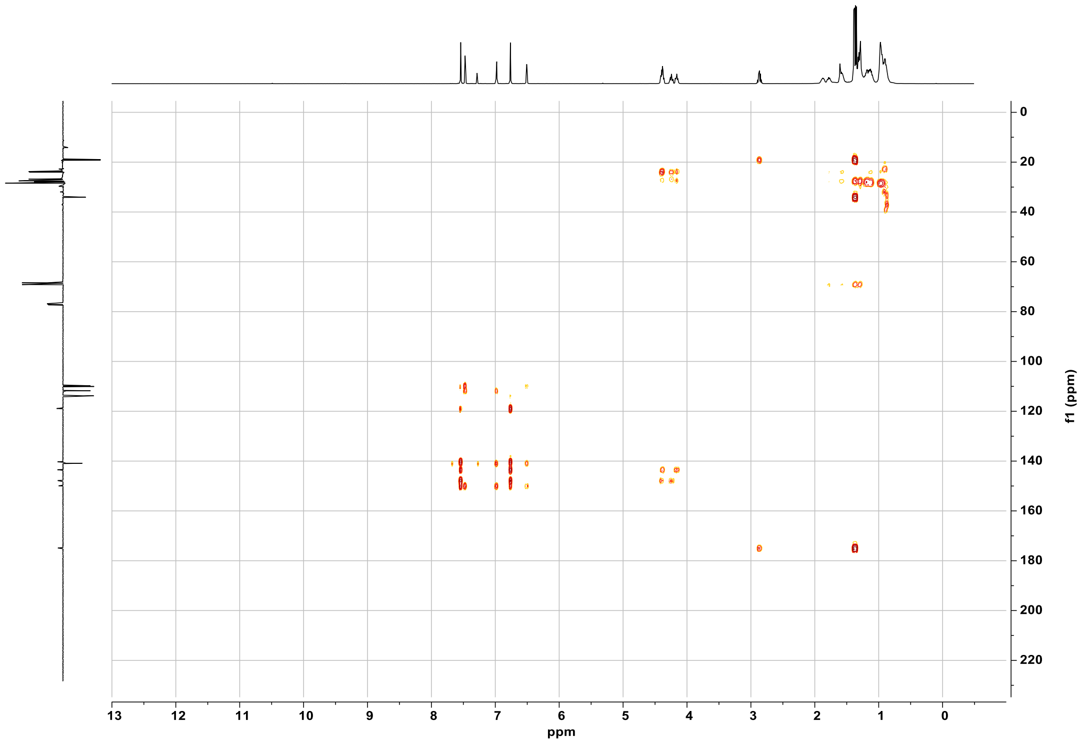

(*Rp*)-1⁵-(thiophen-3-yl)-2,15-dioxa-1(1,4)-benzenacyclopentadecaphane-1²-yl isobutyrate 21

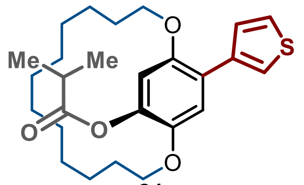

21

^{1}H, $CDCl_3$, 500 MHz

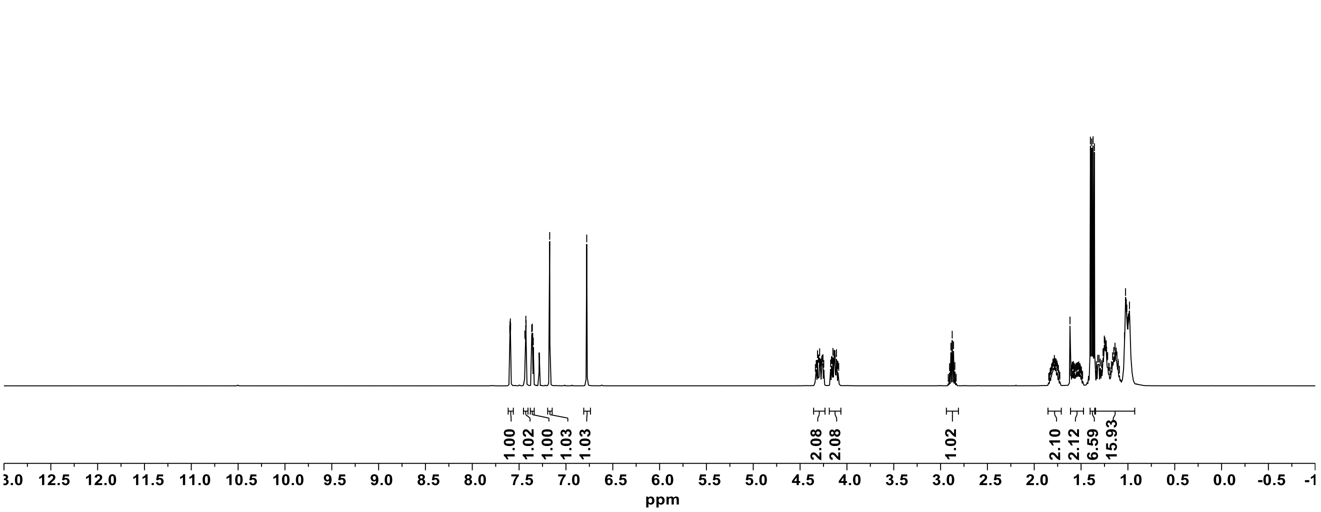

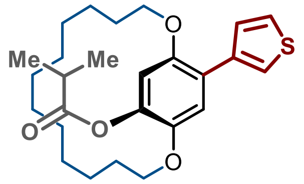

21

$^{13}C\{^{1}H\}$, $CDCl_3$, 126 MHz

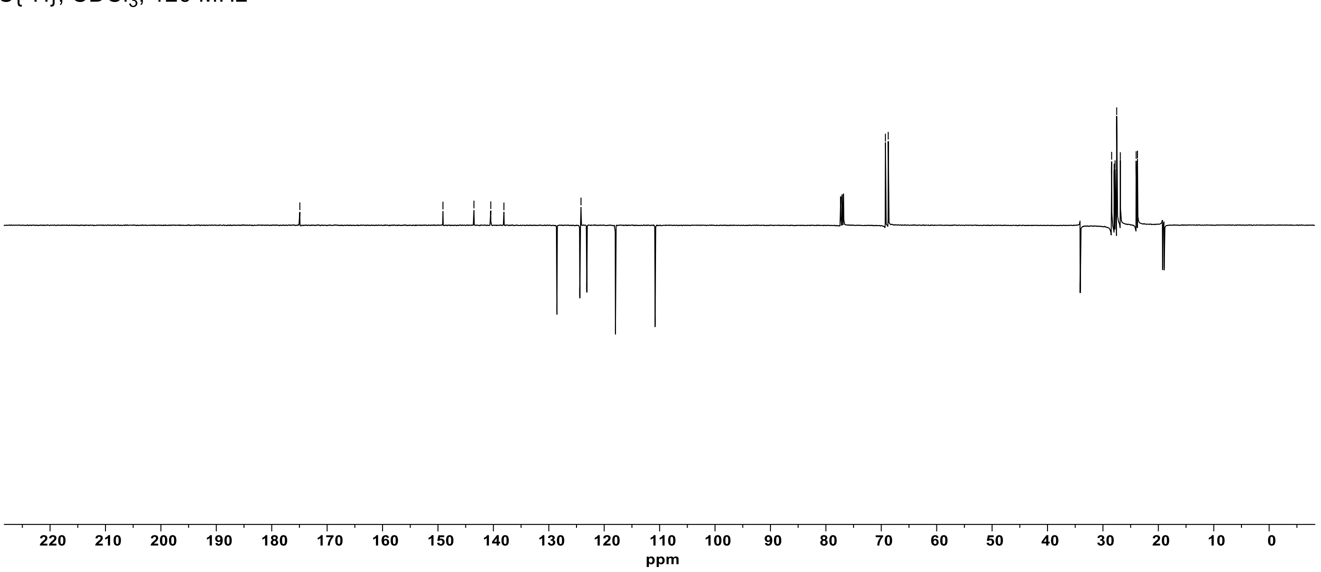

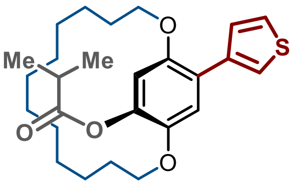

21

2D ^{1}H, $CDCl_3$, COSY

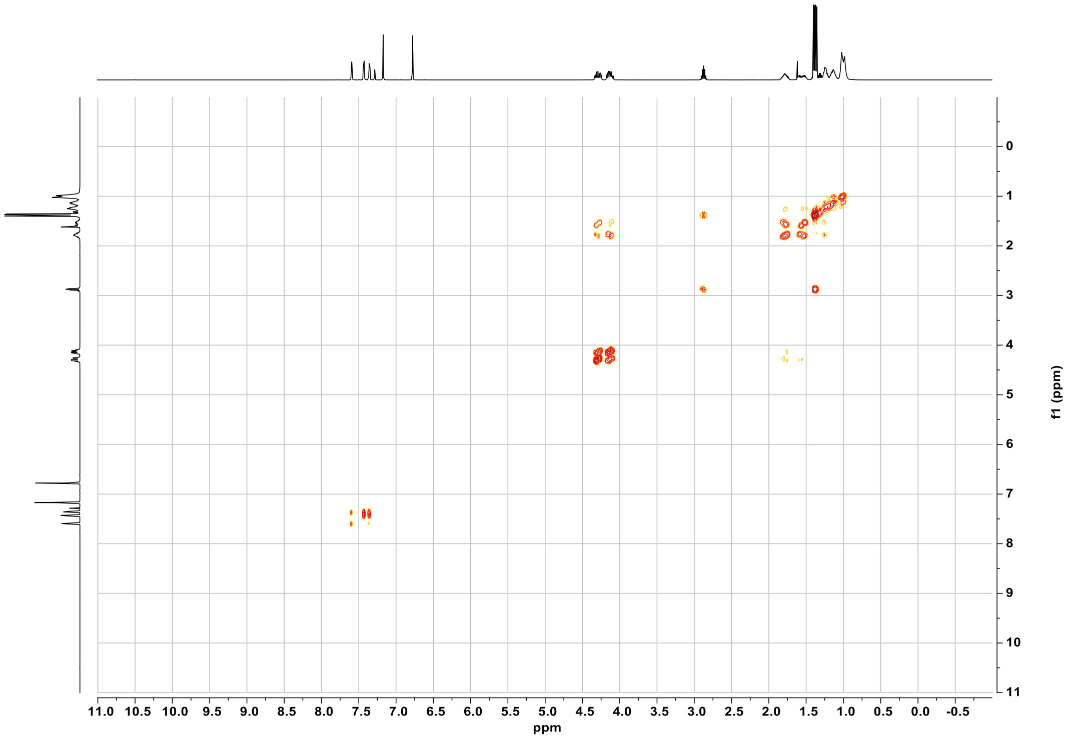

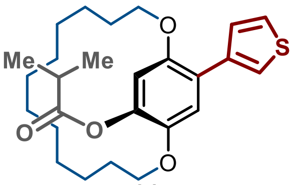

21

2D ^{1}H-^{13}C, $CDCl_3$, HSQC

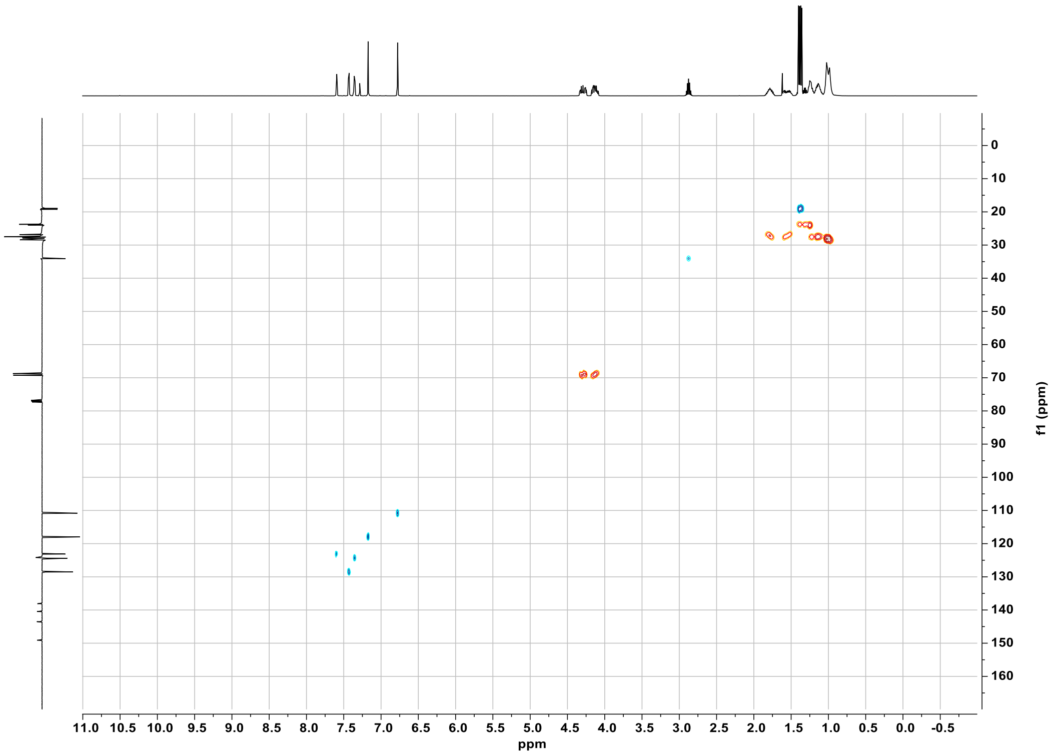

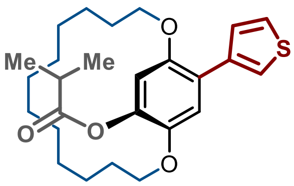

21

2D ^{1}H-^{13}C, $CDCl_3$, HMBC

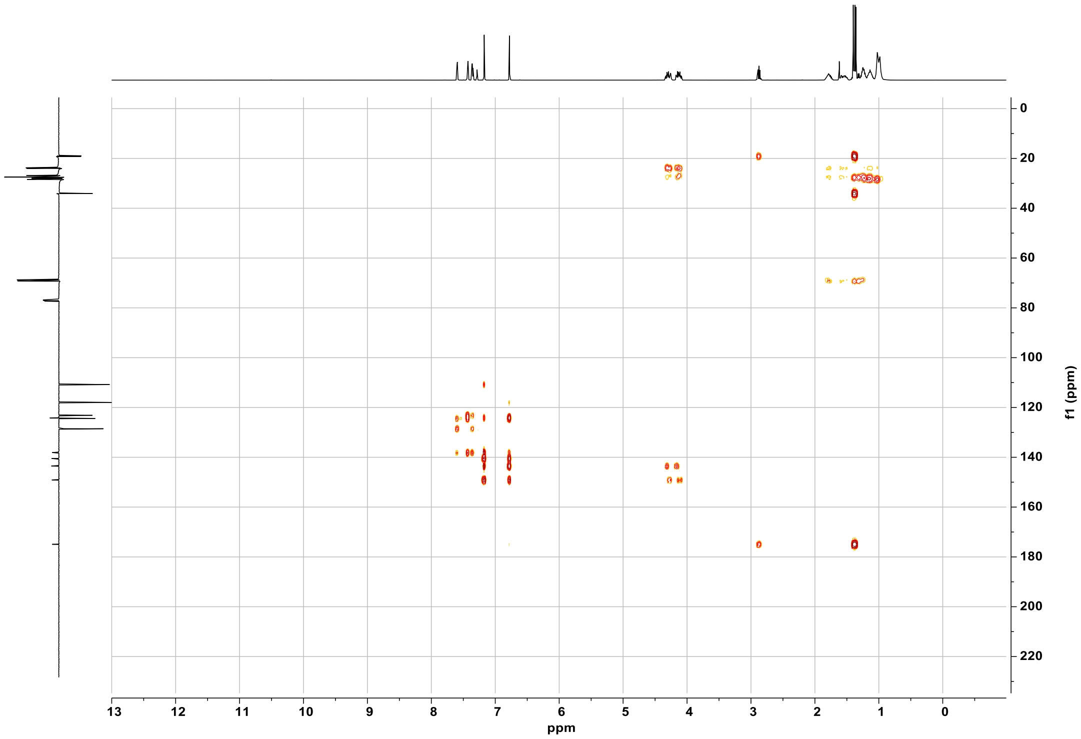

(*Rp*)-*tert*-butyl 3-(1⁵-(isobutyryloxy)-2,15-dioxa-1(1,4)-benzenacyclopentadecaphane-1²-yl)-5-methoxy-1H-indole-1-carboxylate 22

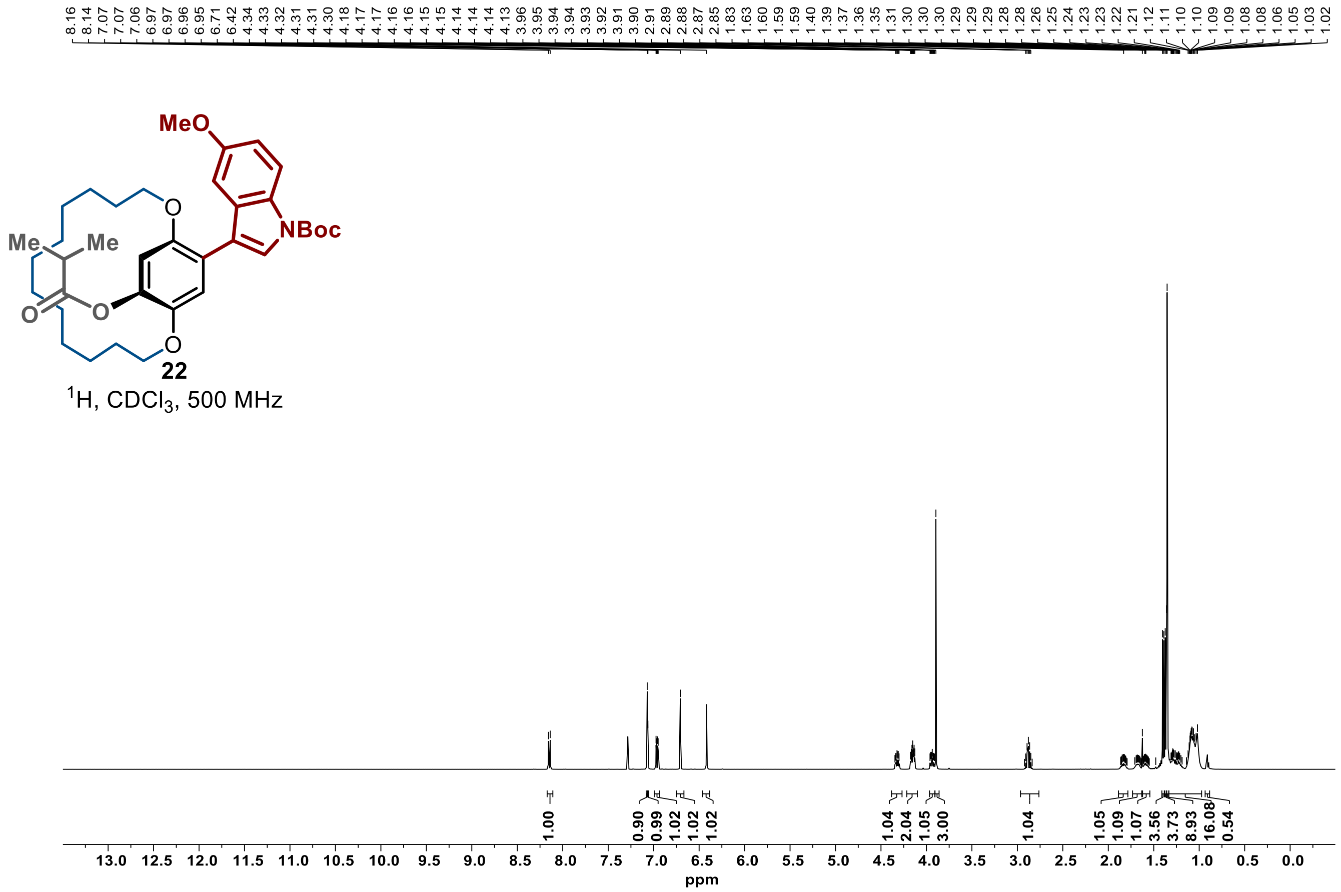

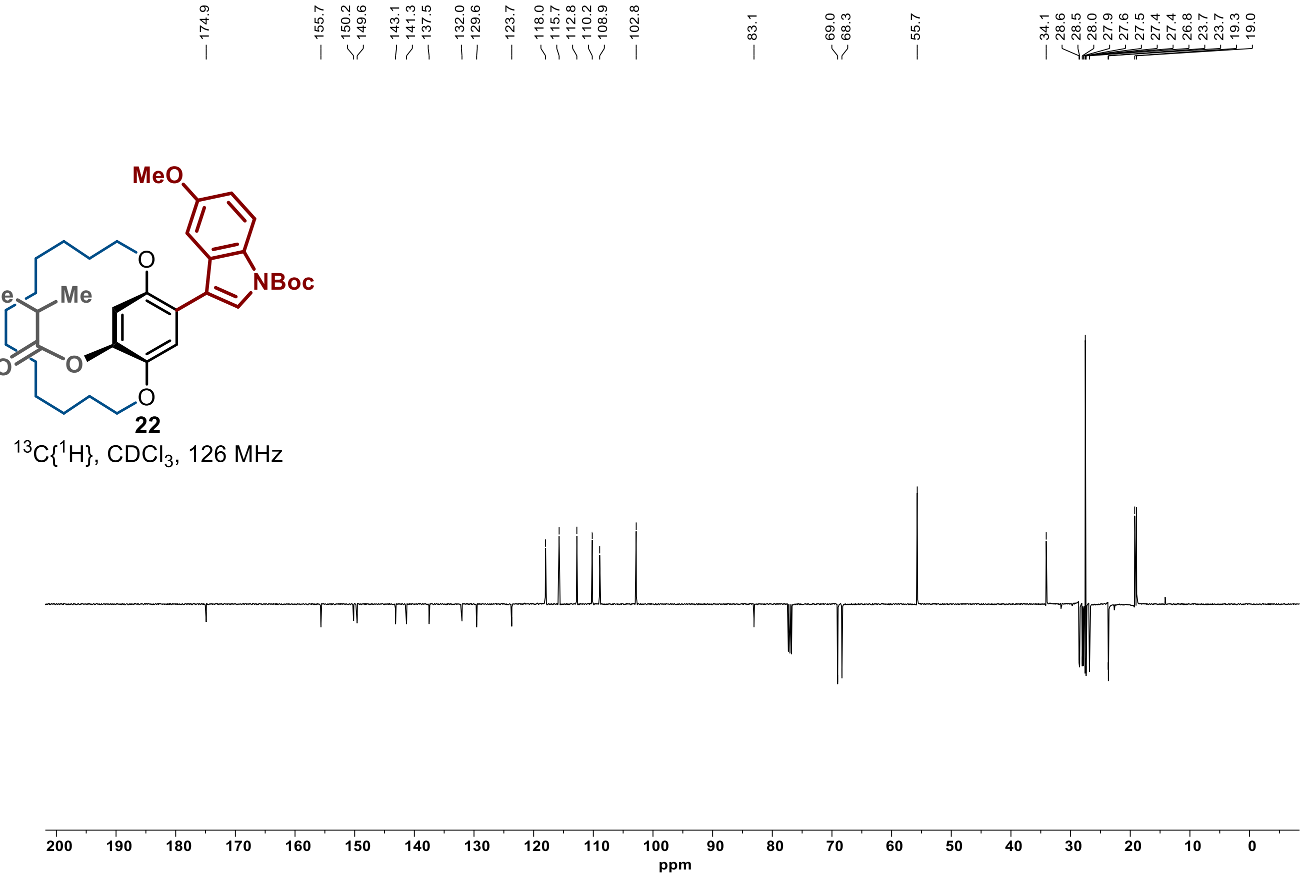

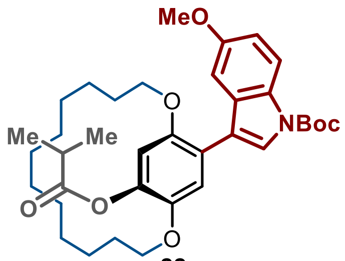

22

2D ^{1}H, $CDCl_3$, COSY

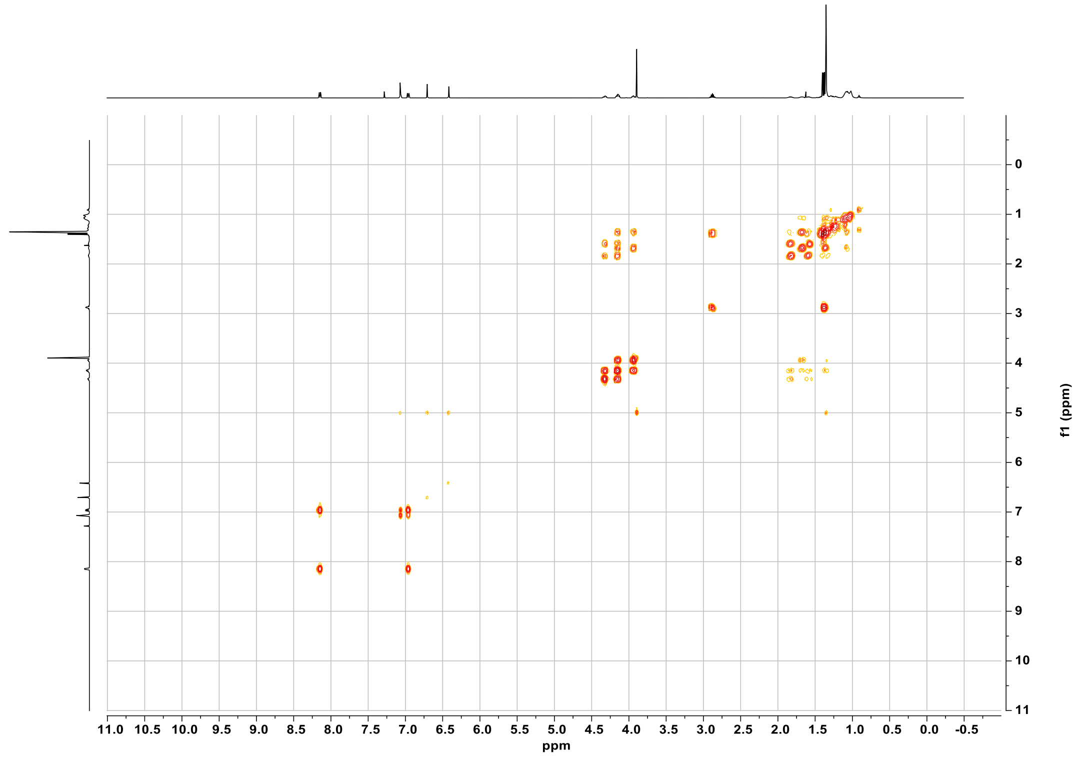

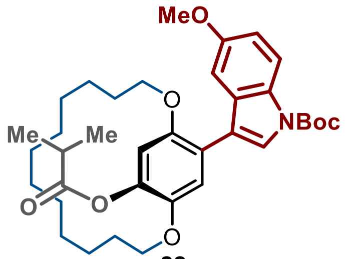

22

2D ^{1}H-^{13}C, $CDCl_3$, HSQC

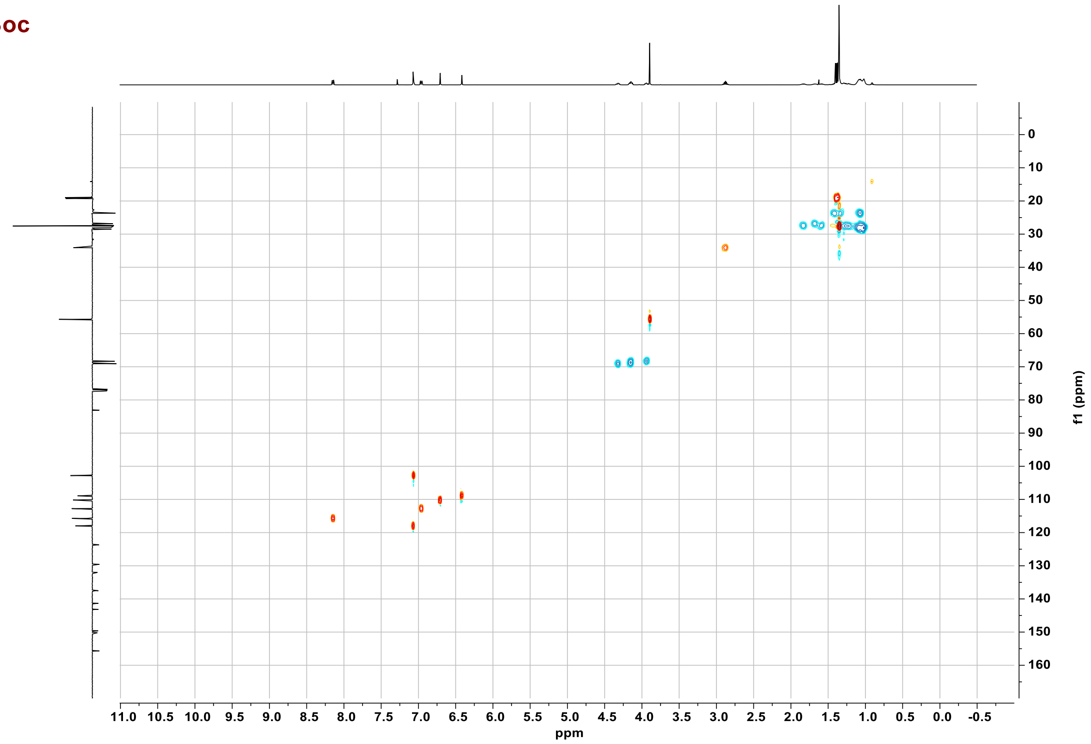

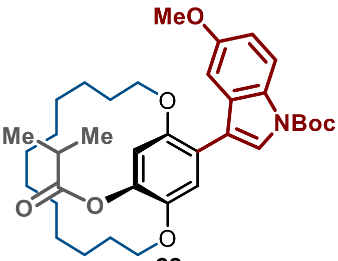

22

2D ^{1}H-^{13}C, $CDCl_3$, HMBC

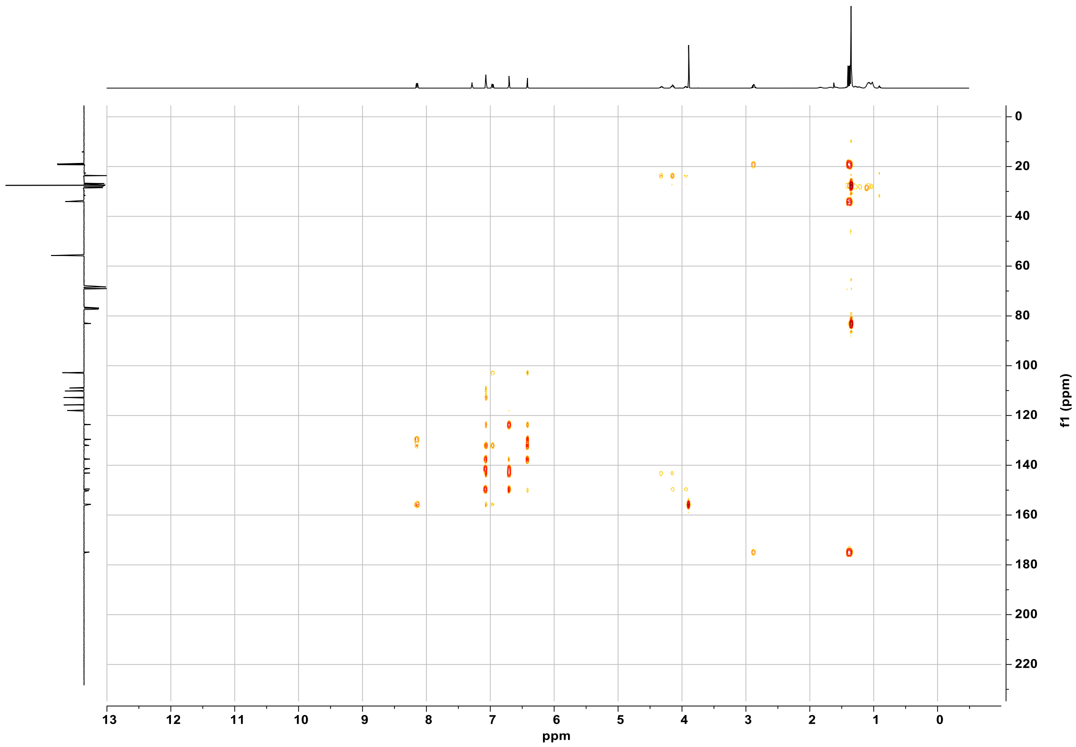

(*Rp*)-1^5-(pyren-1-yl)-2,15-dioxa-1(1,4)-benzenacyclopentadecaphane-1^2-yl isobutyrate 23

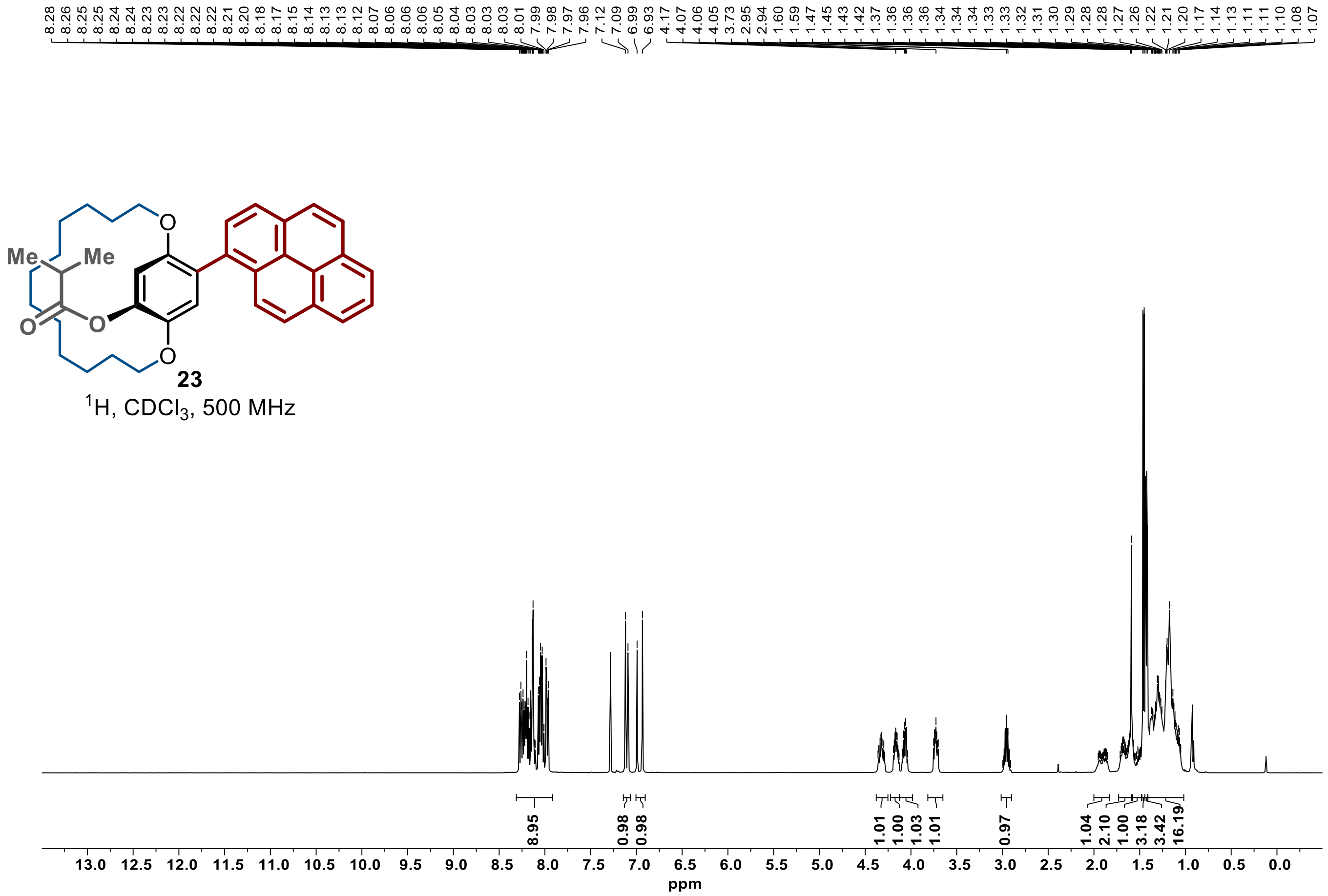

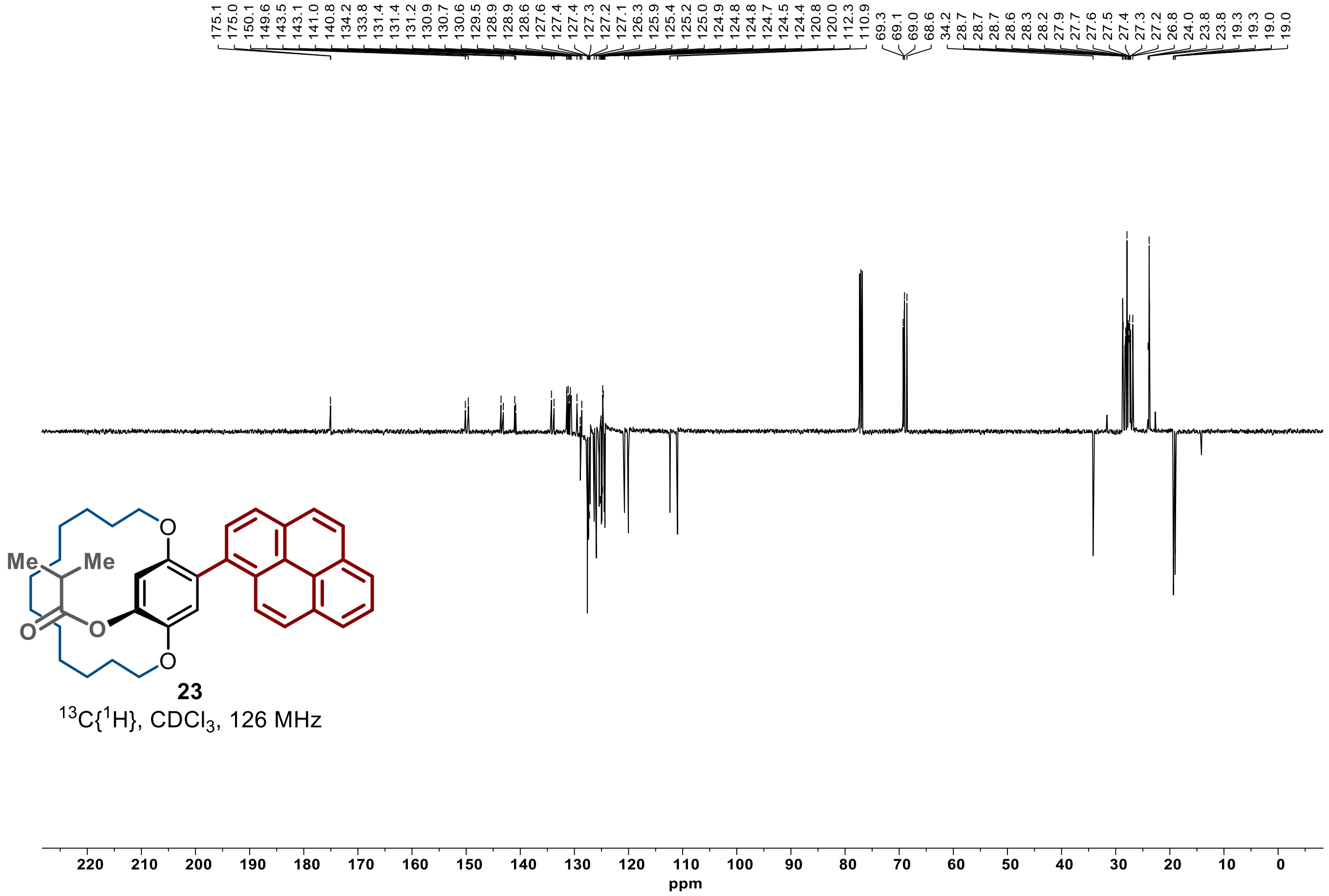

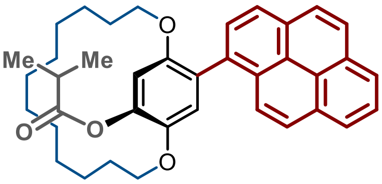

23

2D ^{1}H, $CDCl_3$, COSY

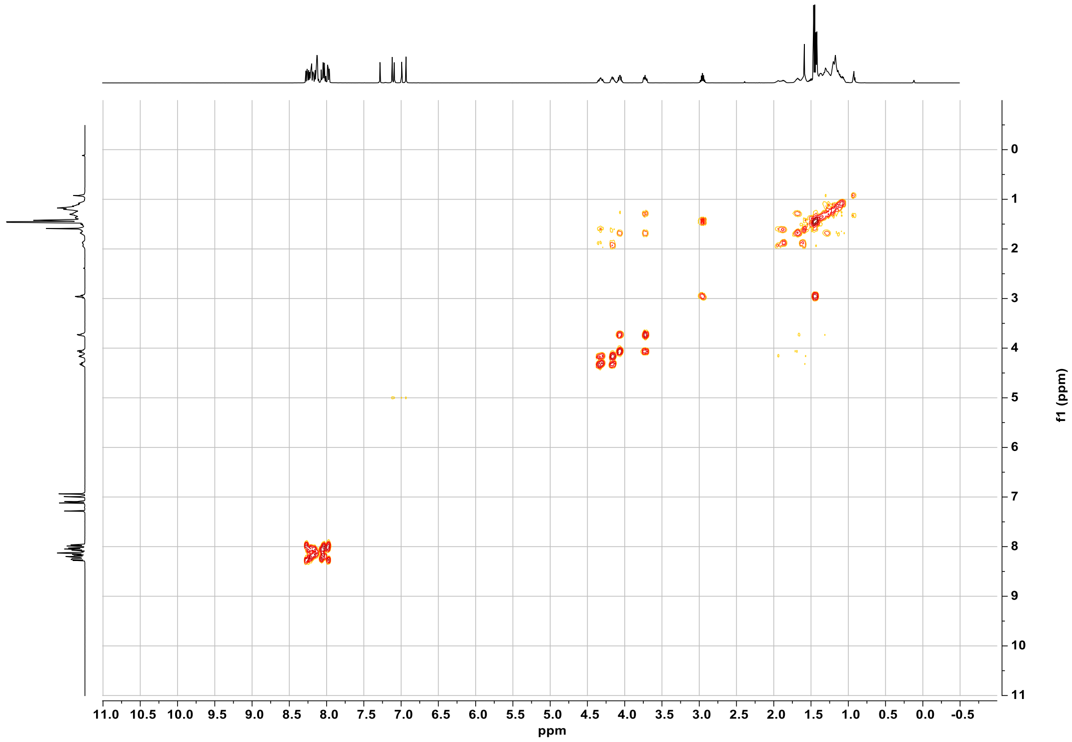

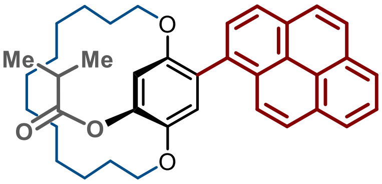

23

2D ^{1}H-^{13}C, $CDCl_3$, HSQC

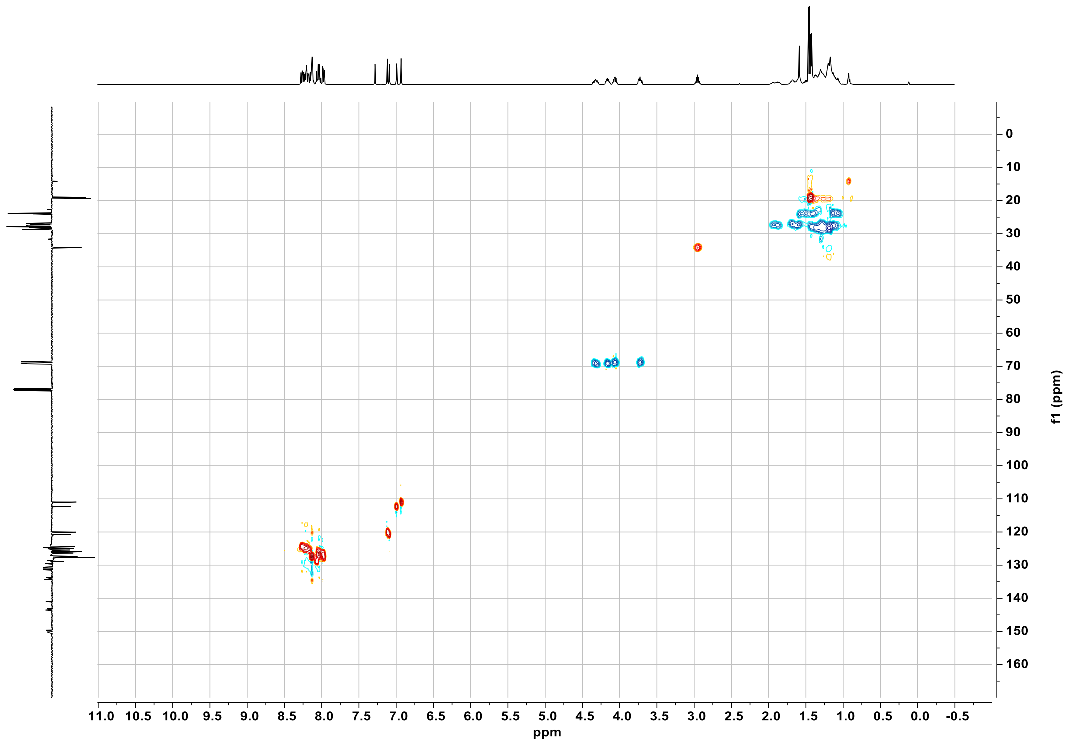

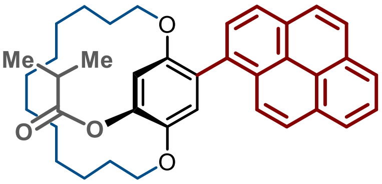

23

2D ^{1}H-^{13}C, $CDCl_3$, HMBC

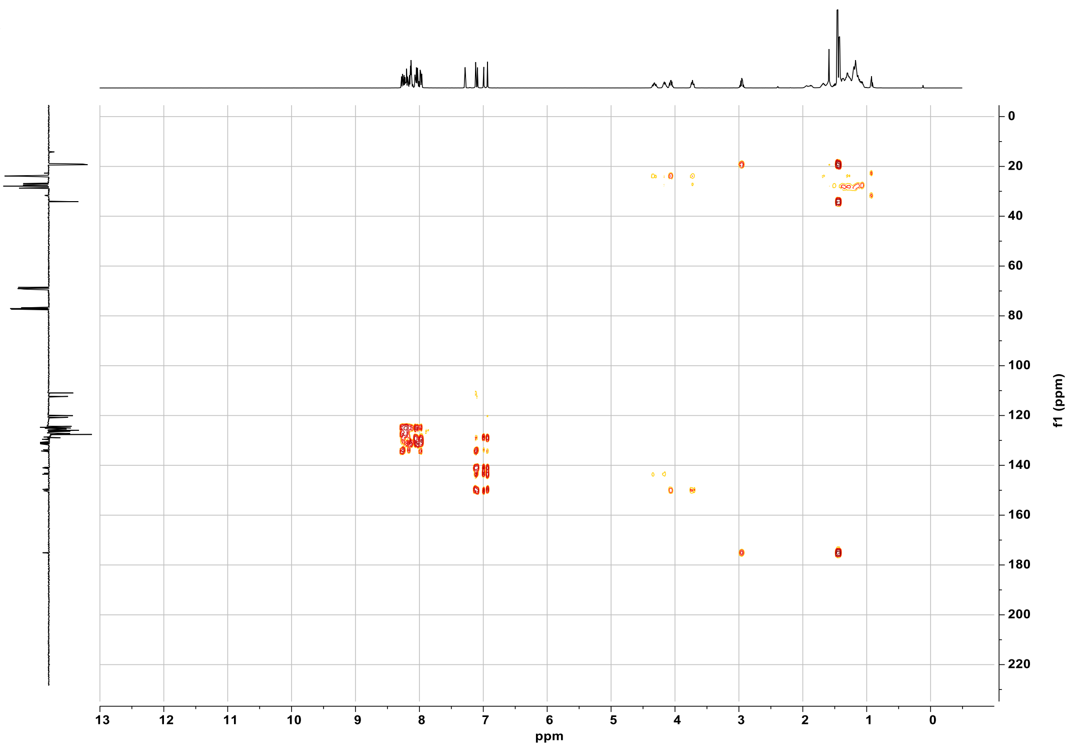

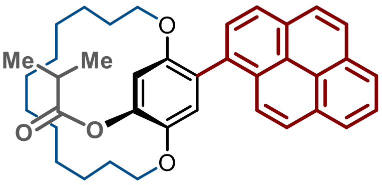

23

2D ^{1}H, $CDCl_3$, NOESY

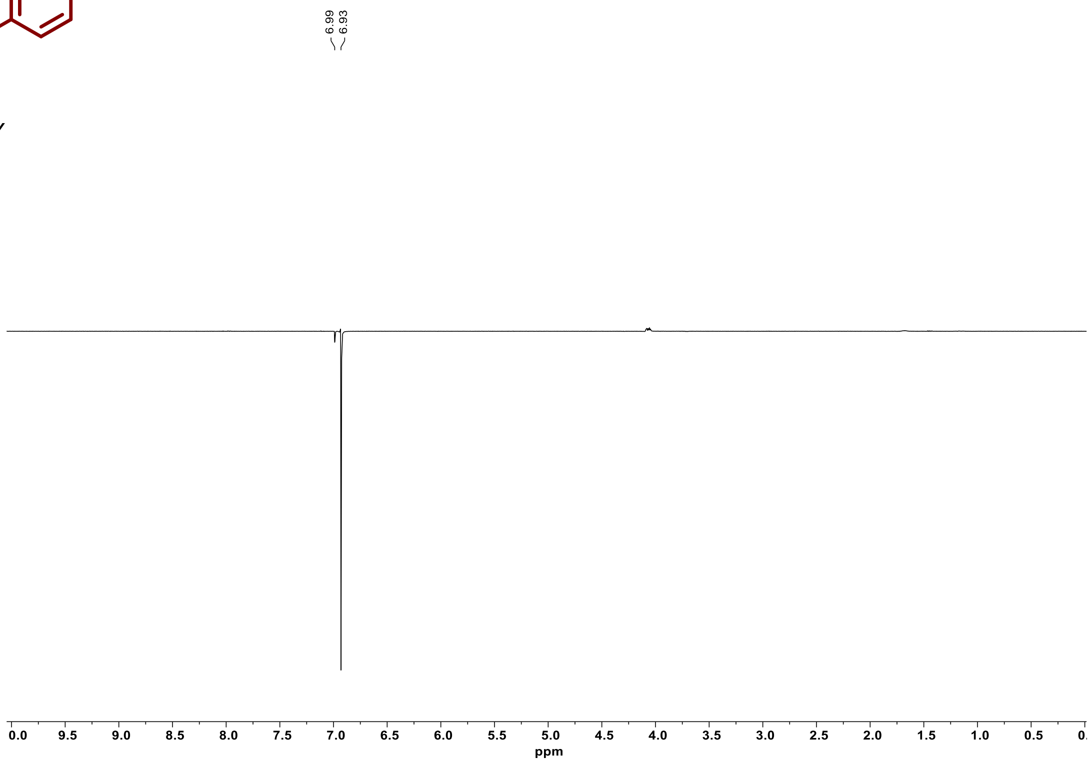

(*Rp*)-2,15-dioxa-1(1,4)-naphthalenacyclopentadecaphane-1²-yl isobutyrate 24

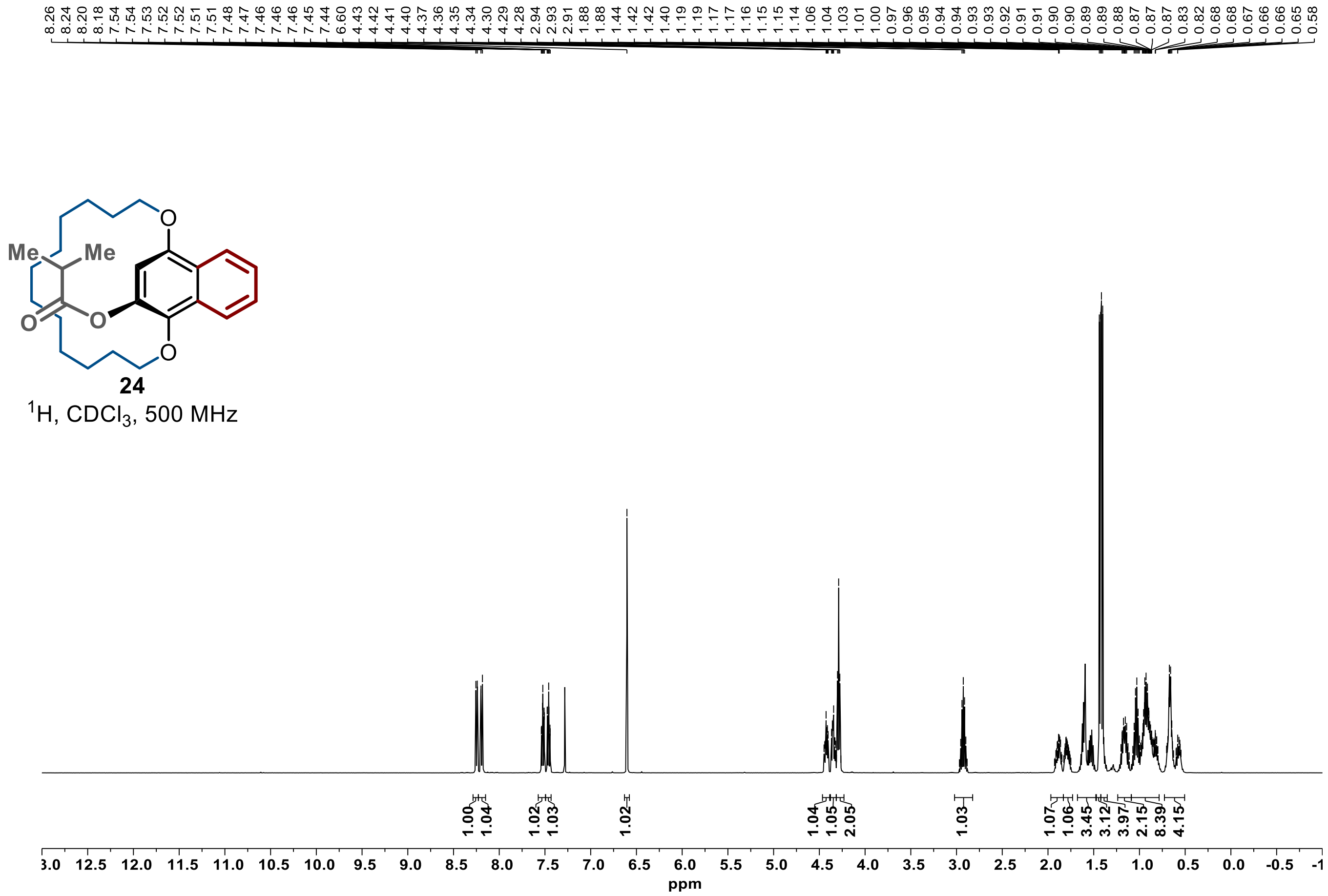

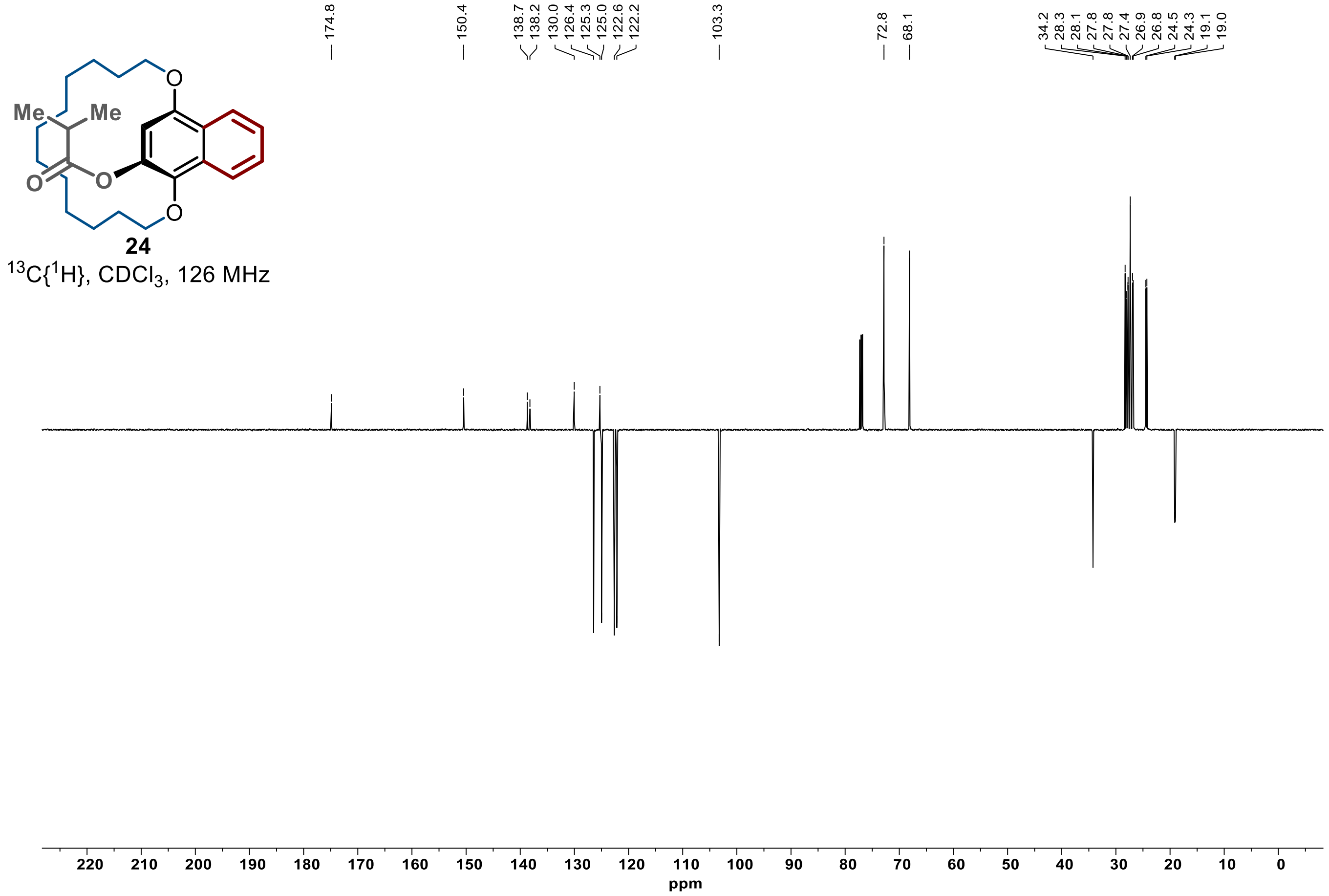

(*Rp*)-1⁵-butyl-2,15-dioxa-1(1,4)-benzenacyclopentadecaphane-1²-yl isobutyrate 25

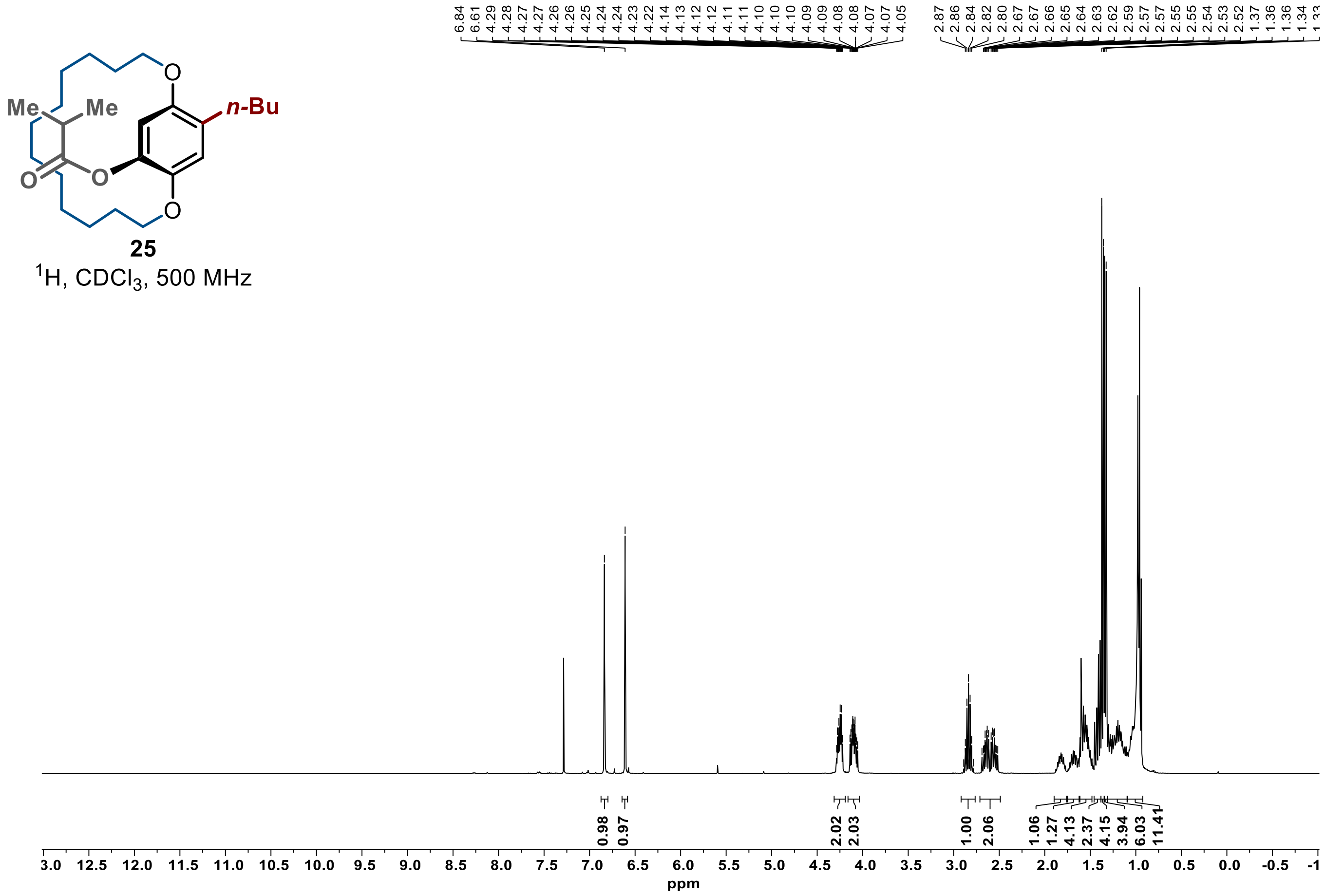

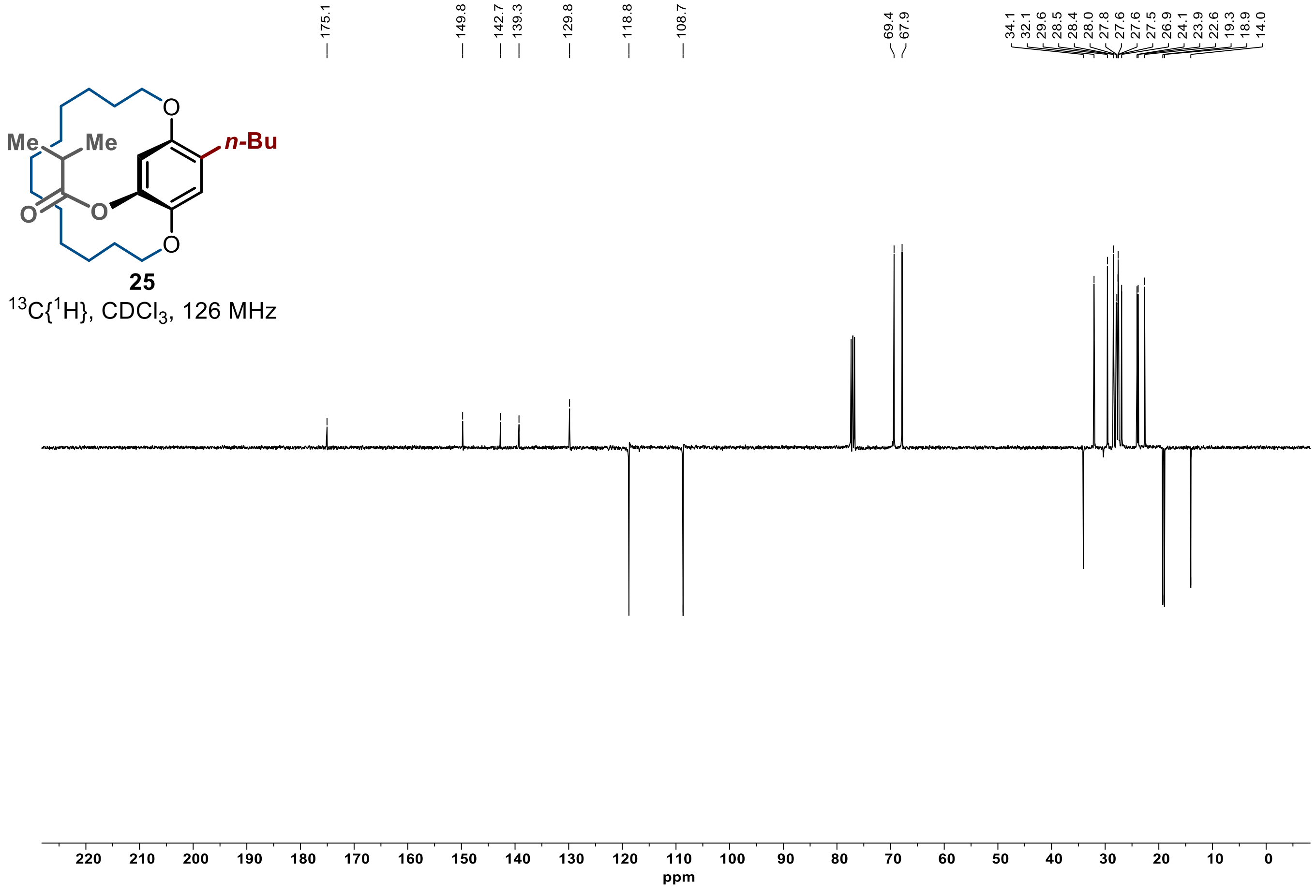

(Rp)-1^5-bromo-2,6,11,15-tetraoxa-1(1,4)-benzenacyclopentadecaphane-1^2-yl isobutyrate 26

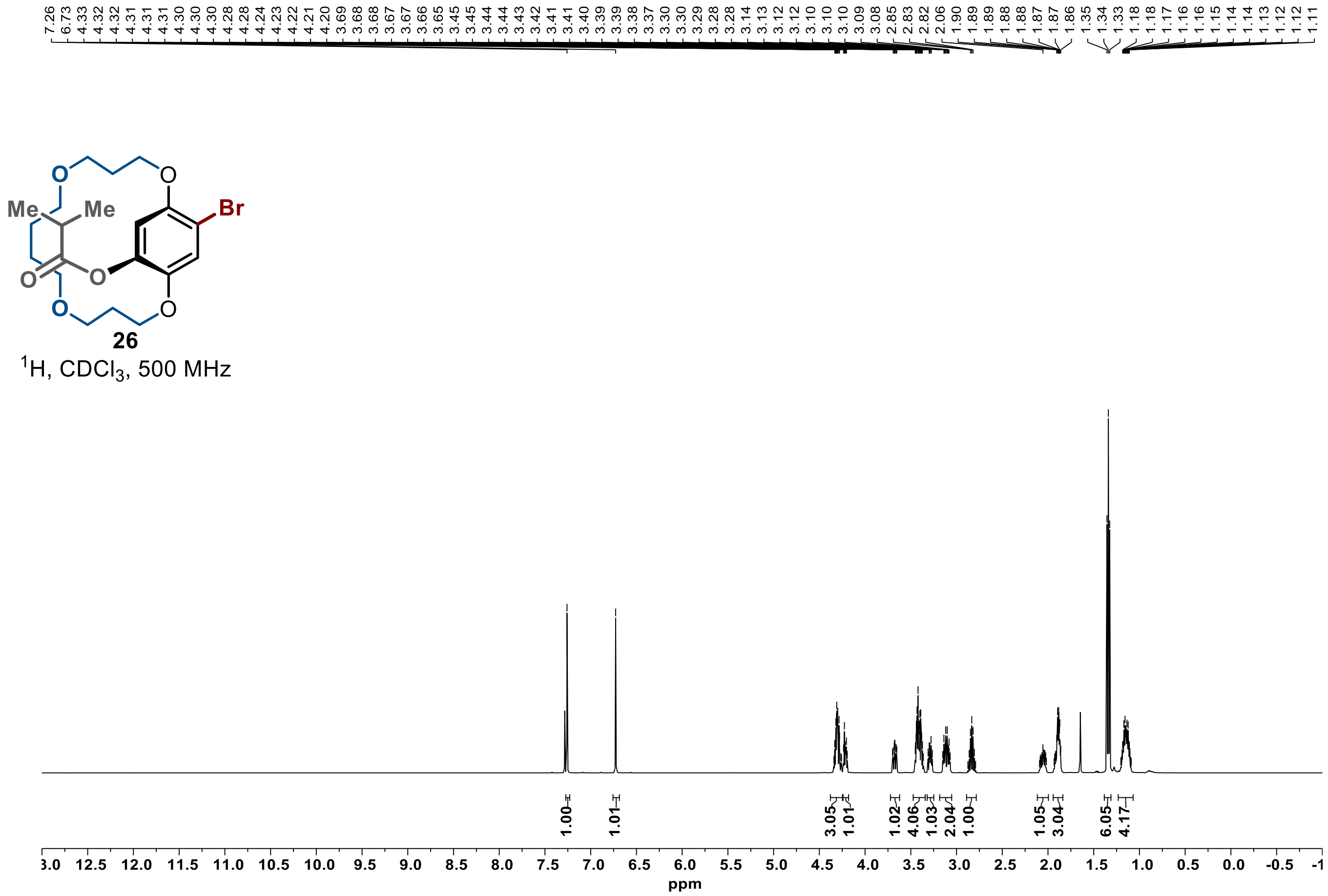

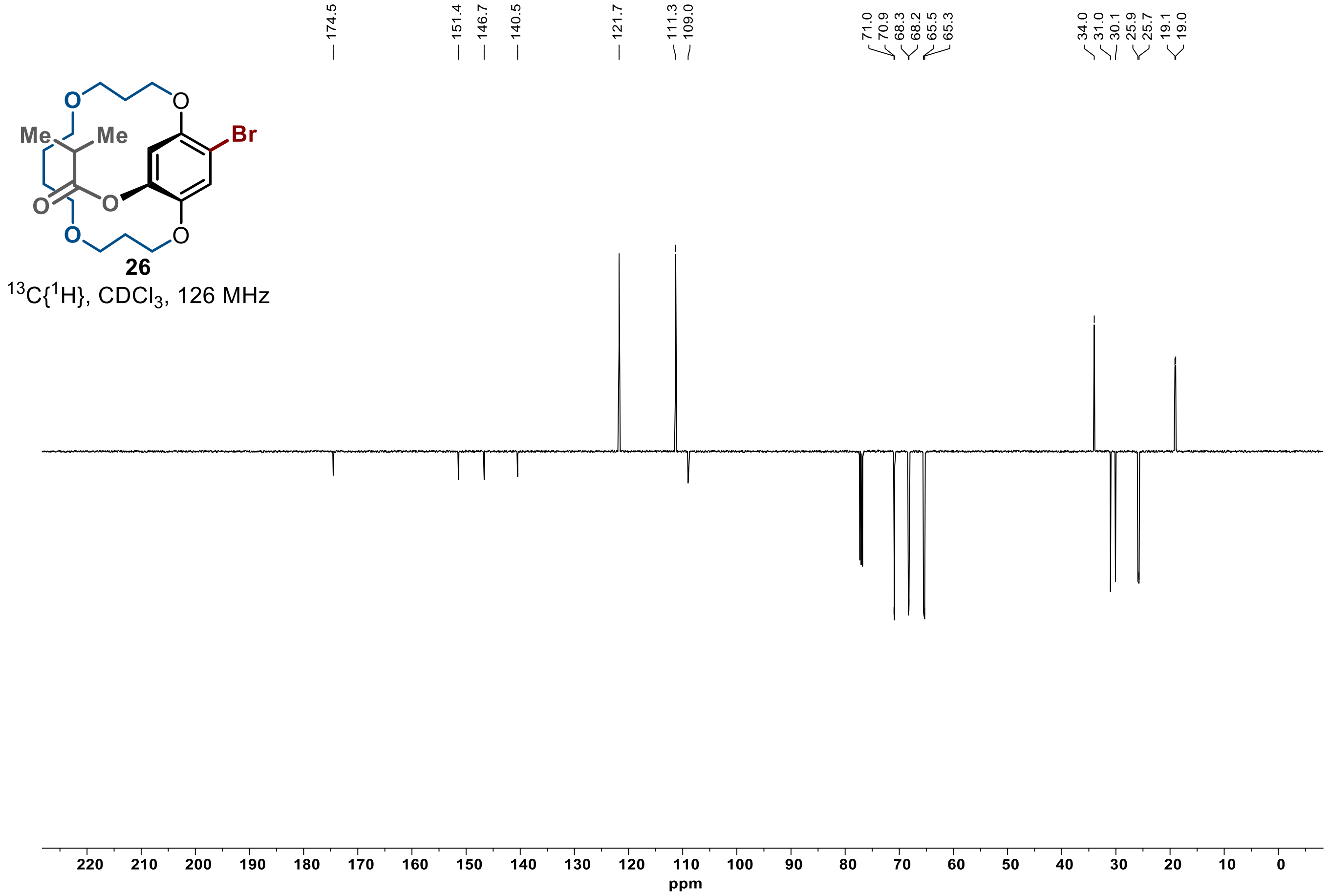

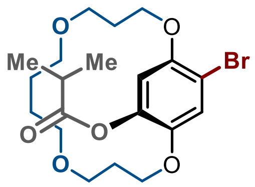

26

2D ^{1}H, $CDCl_3$, COSY

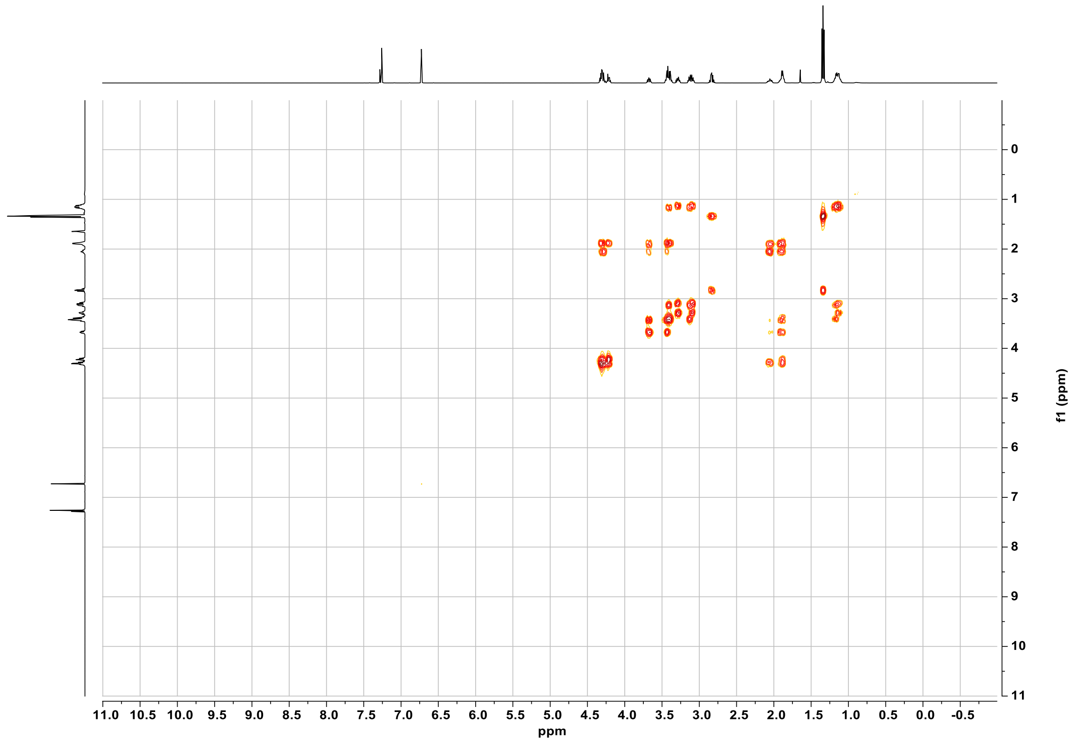

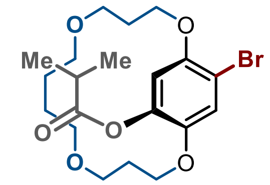

26

2D ^{1}H-^{13}C, $CDCl_3$, HSQC

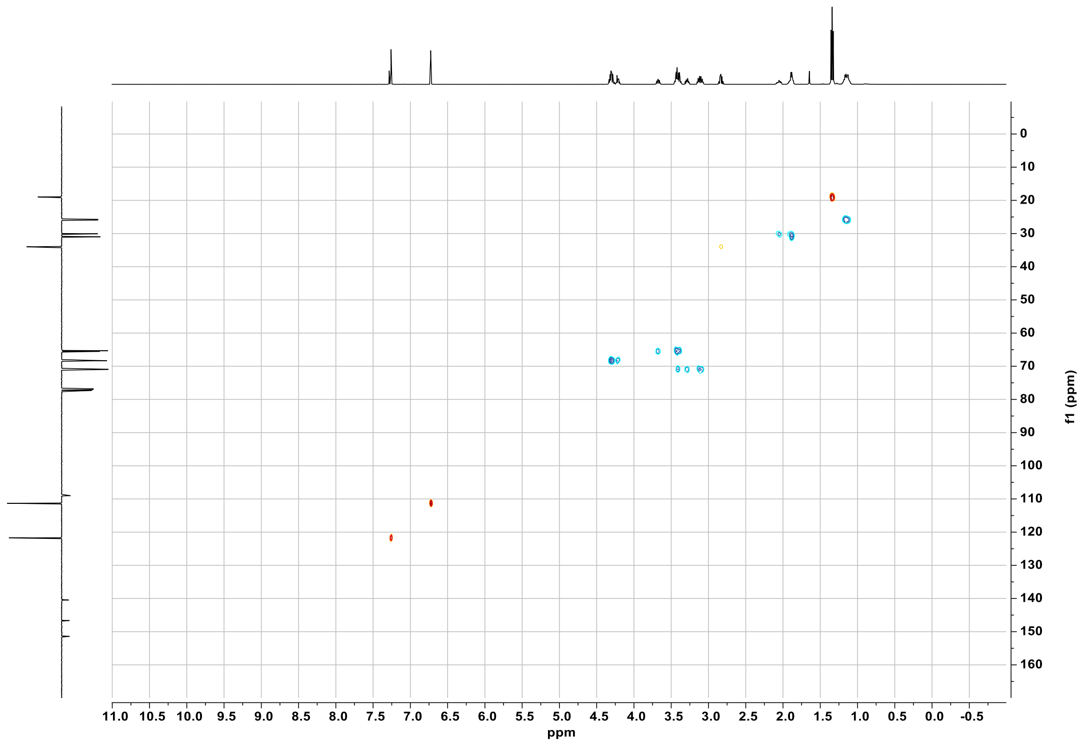

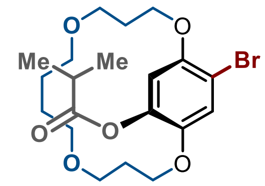

26

2D ^{1}H-^{13}C, $CDCl_3$, HMBC

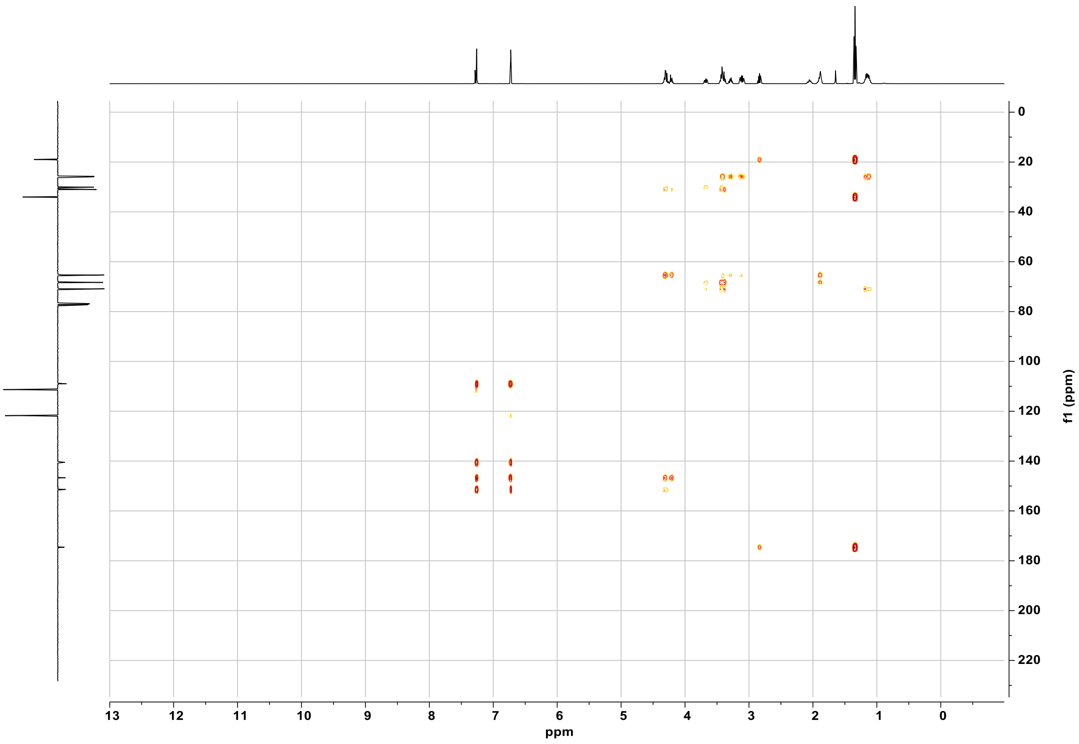

(*Rp*)-1⁵-bromo-2,16-dioxa-1(1,4)-benzenacyclohexadecaphane-1²-yl isobutyrate 27

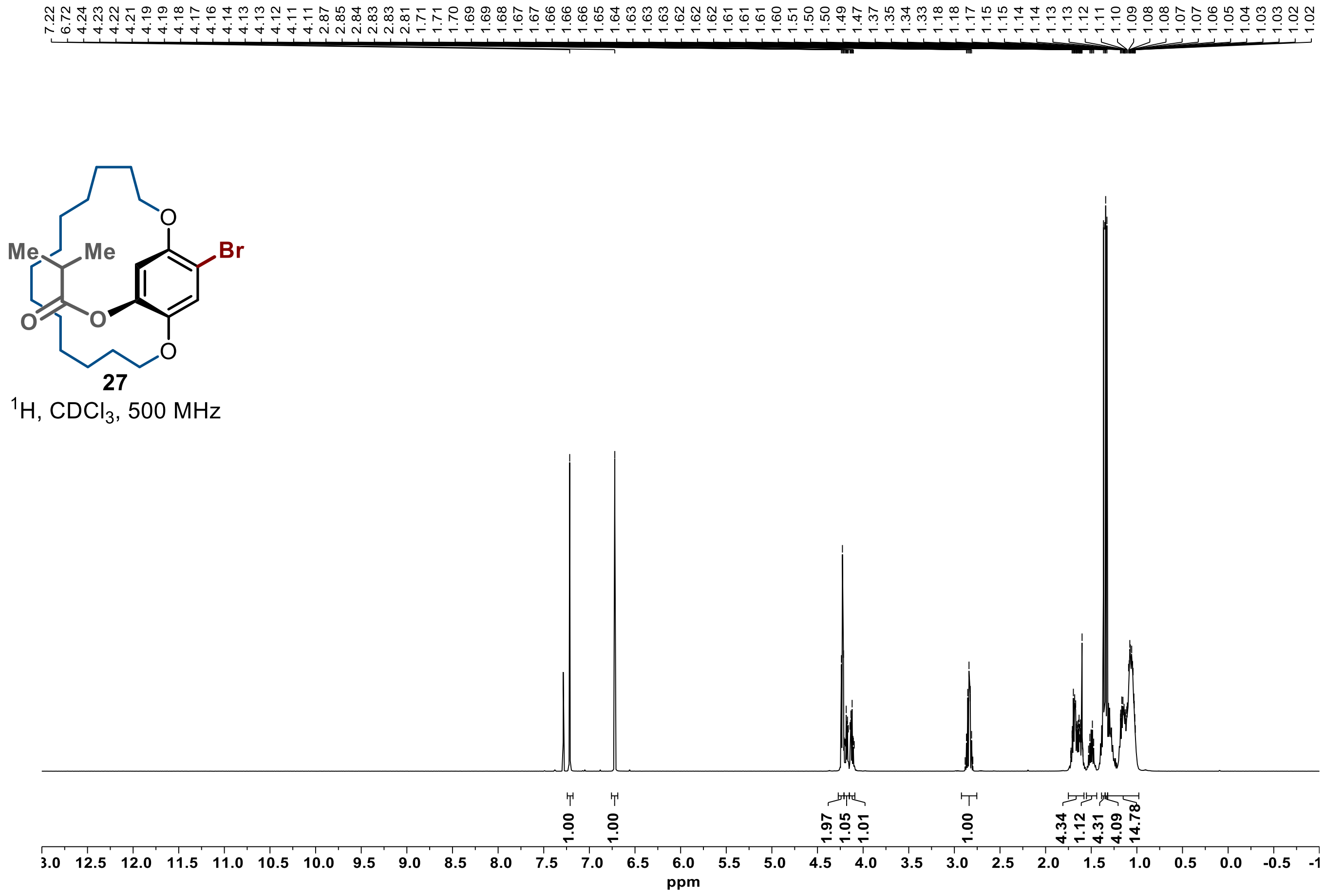

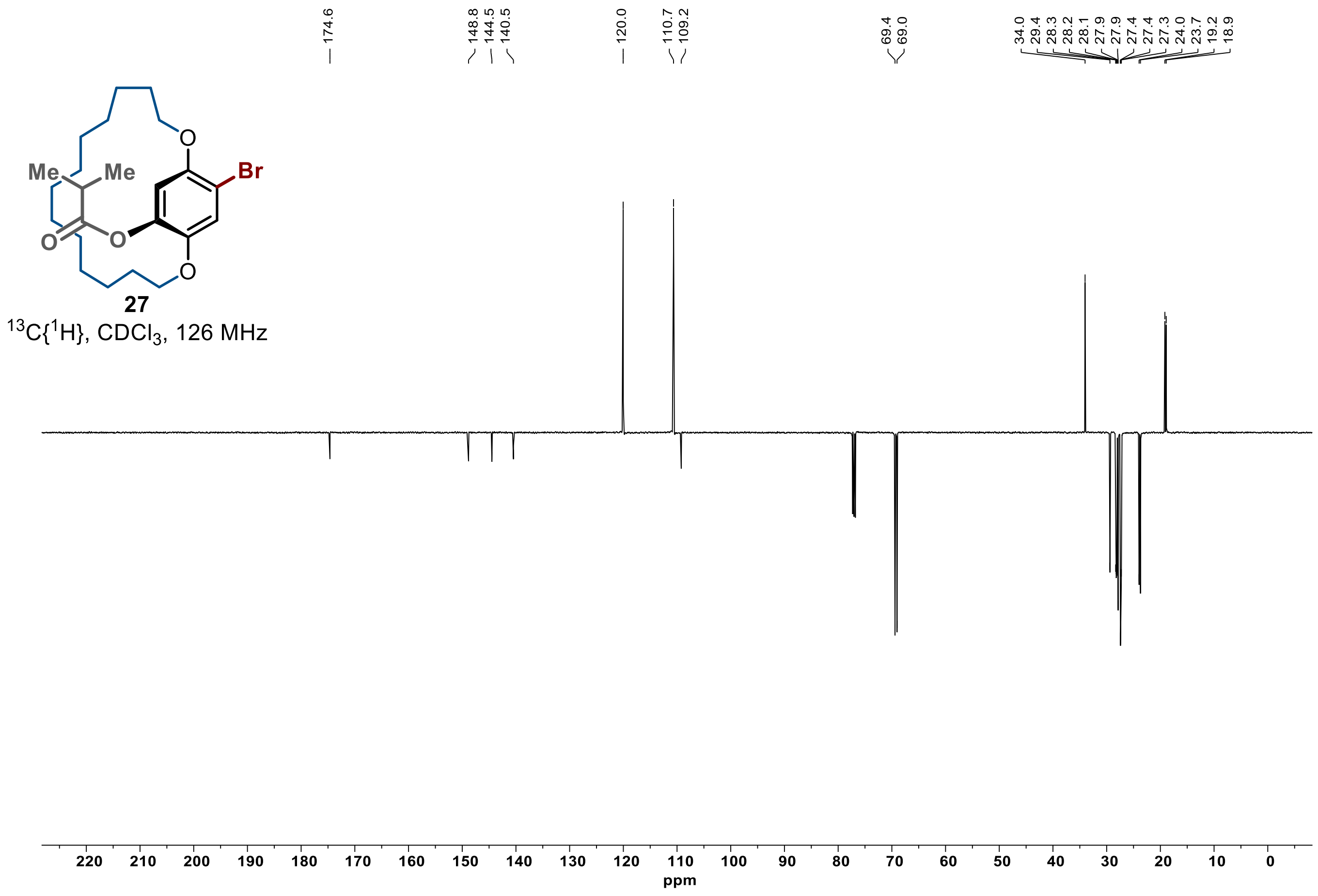

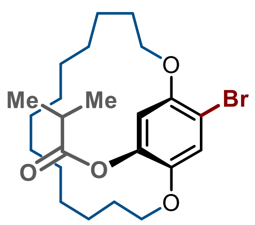

27

2D ^{1}H, $CDCl_3$, COSY

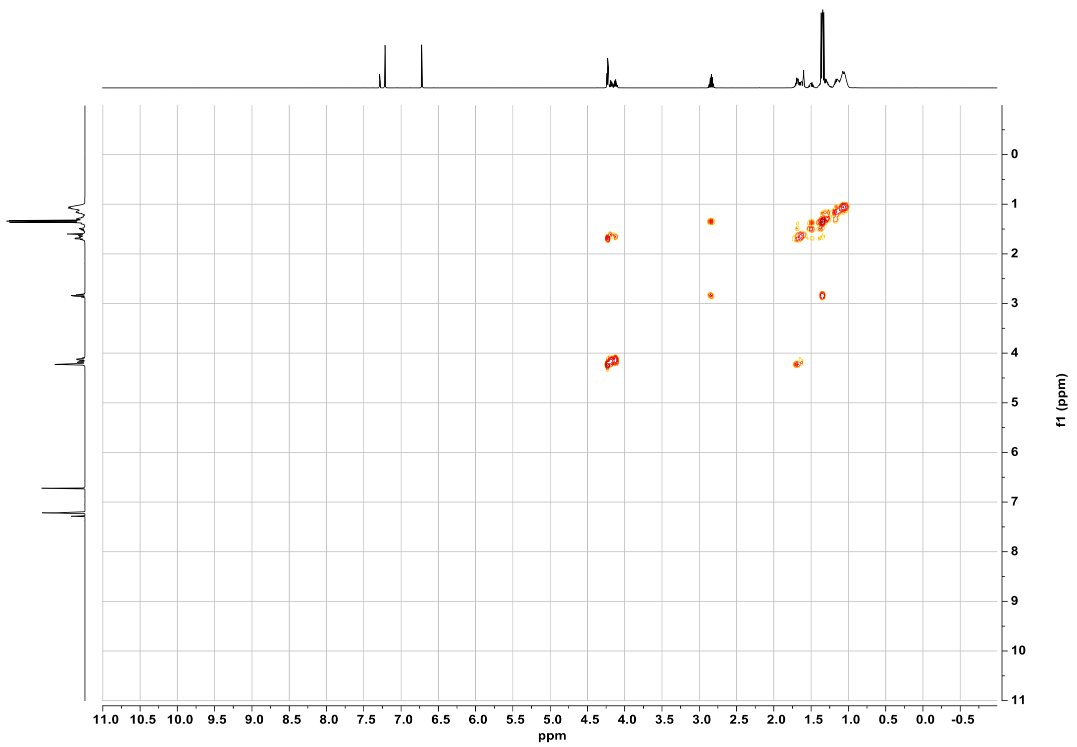

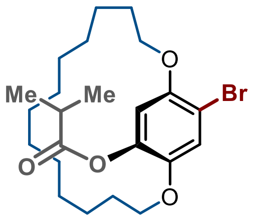

27

2D ^{1}H-^{13}C, $CDCl_3$, HSQC

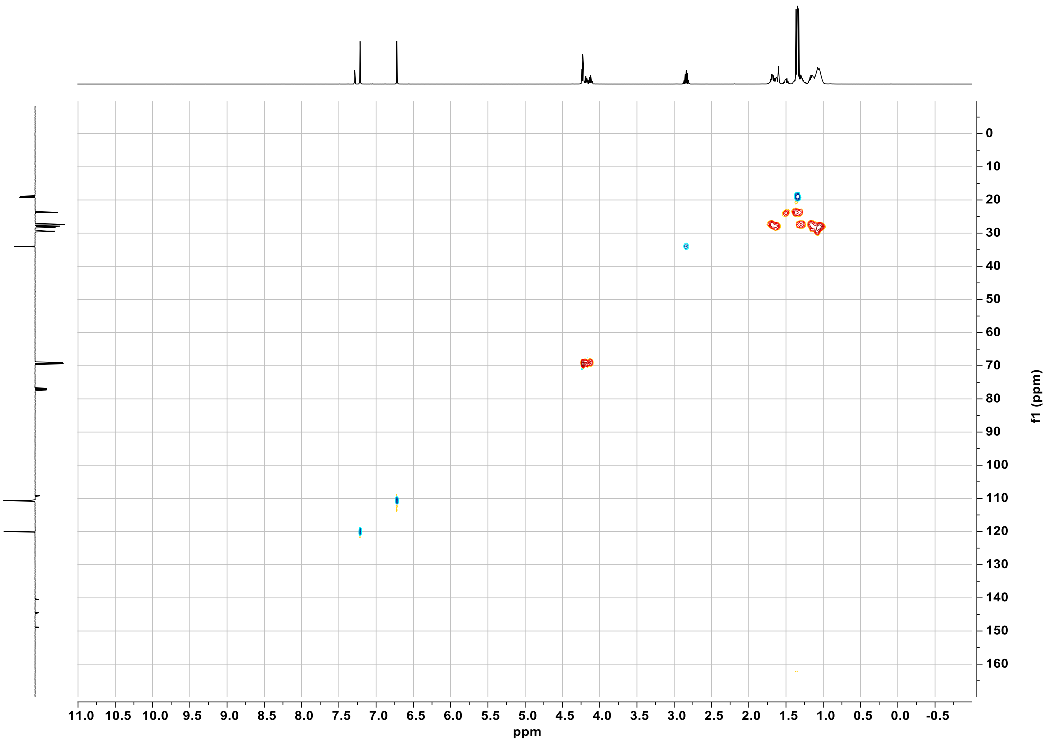

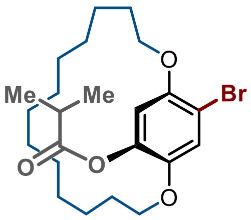

27

2D ^{1}H-^{13}C, $CDCl_3$, HMBC

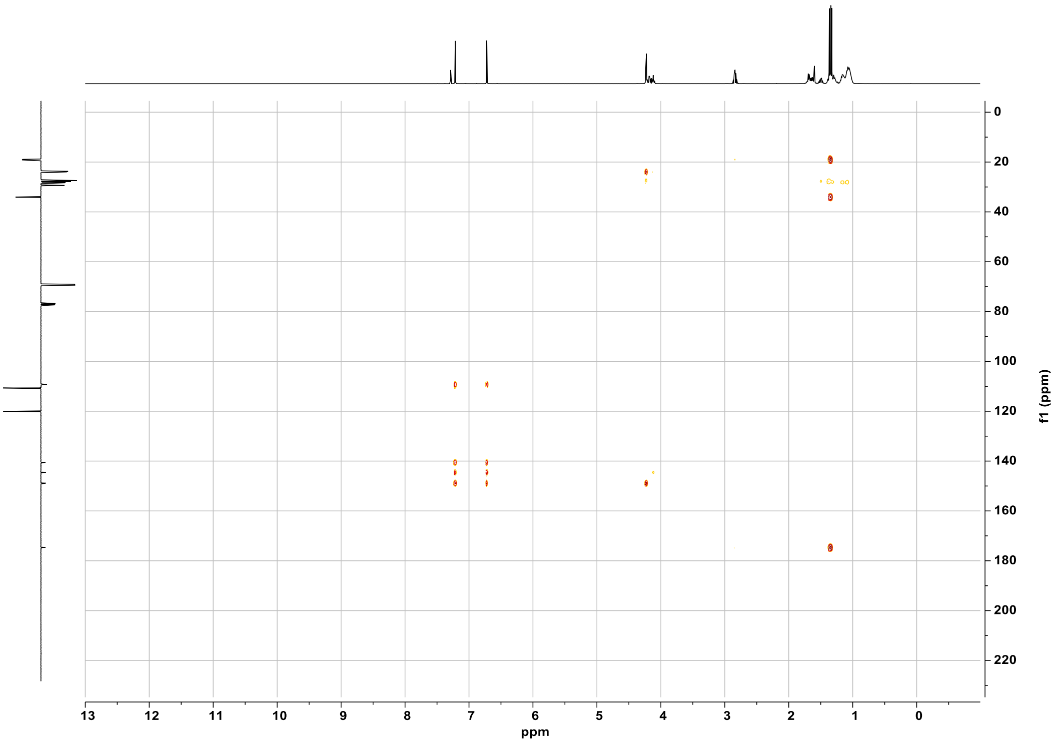

(*Rp*)-1⁵-phenyl-2,16-dioxa-1(1,4)-benzenacyclohexadecaphane-1²-yl isobutyrate 28

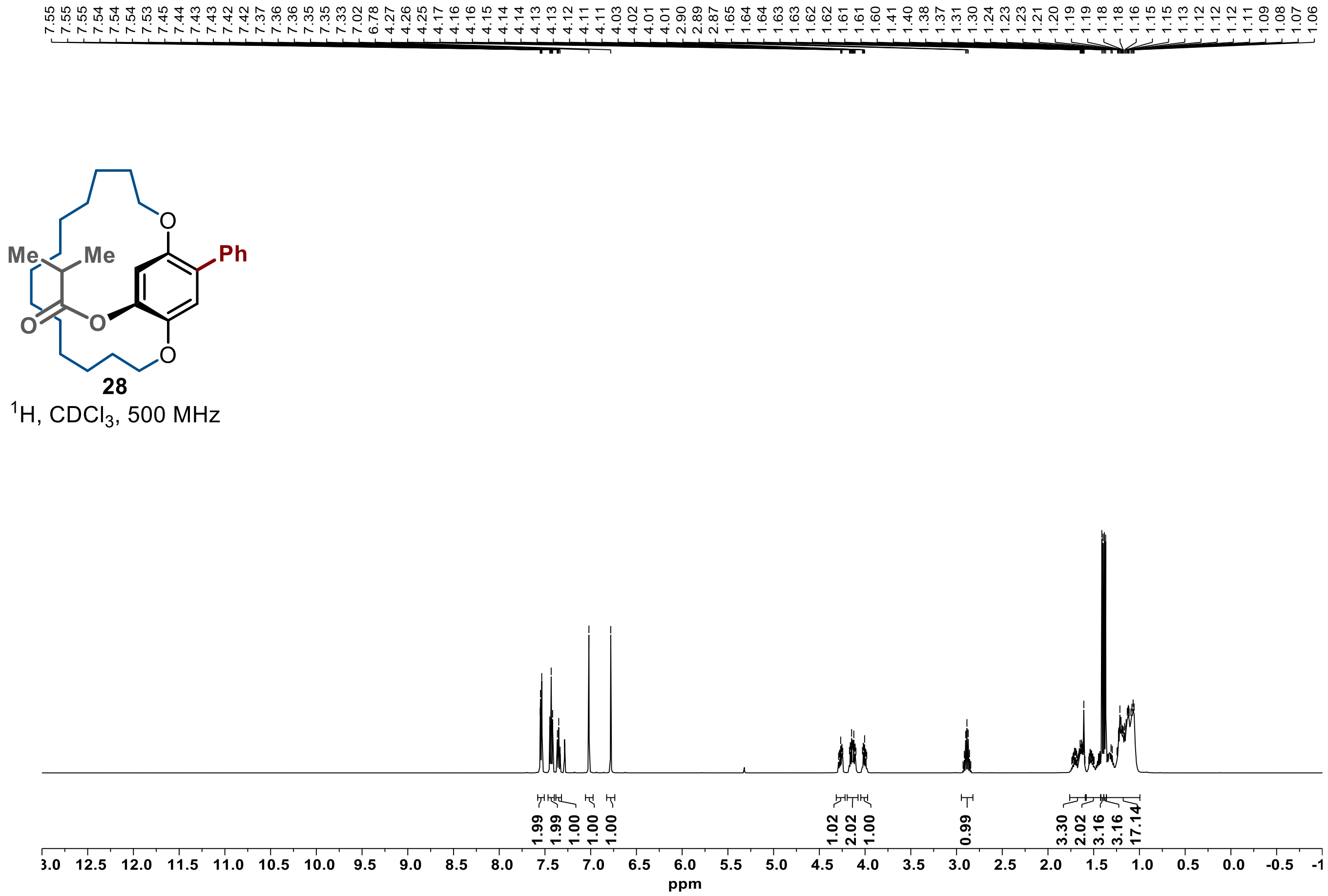

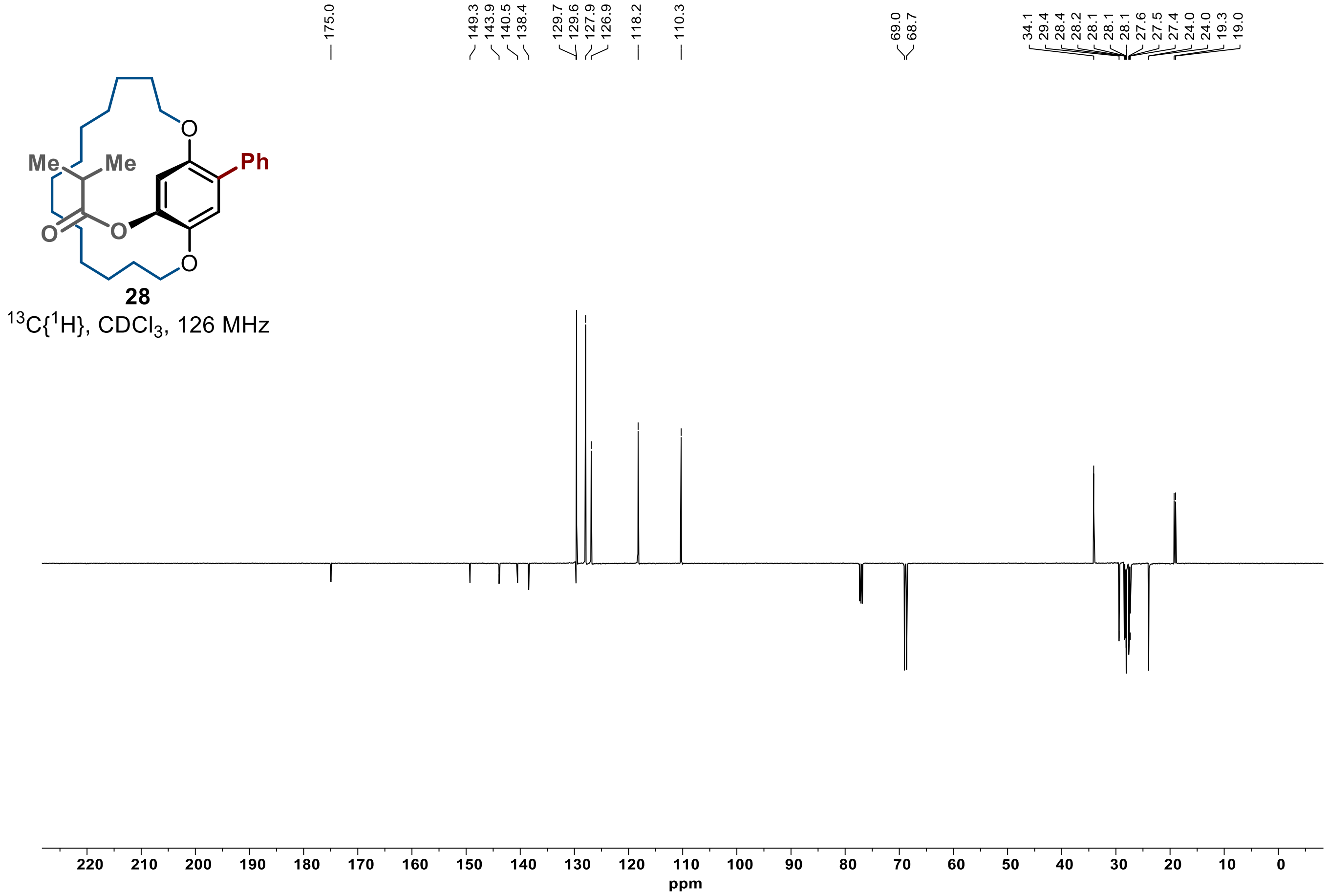

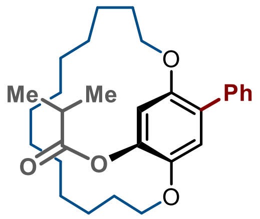

28

2D ^{1}H, $CDCl_3$, COSY

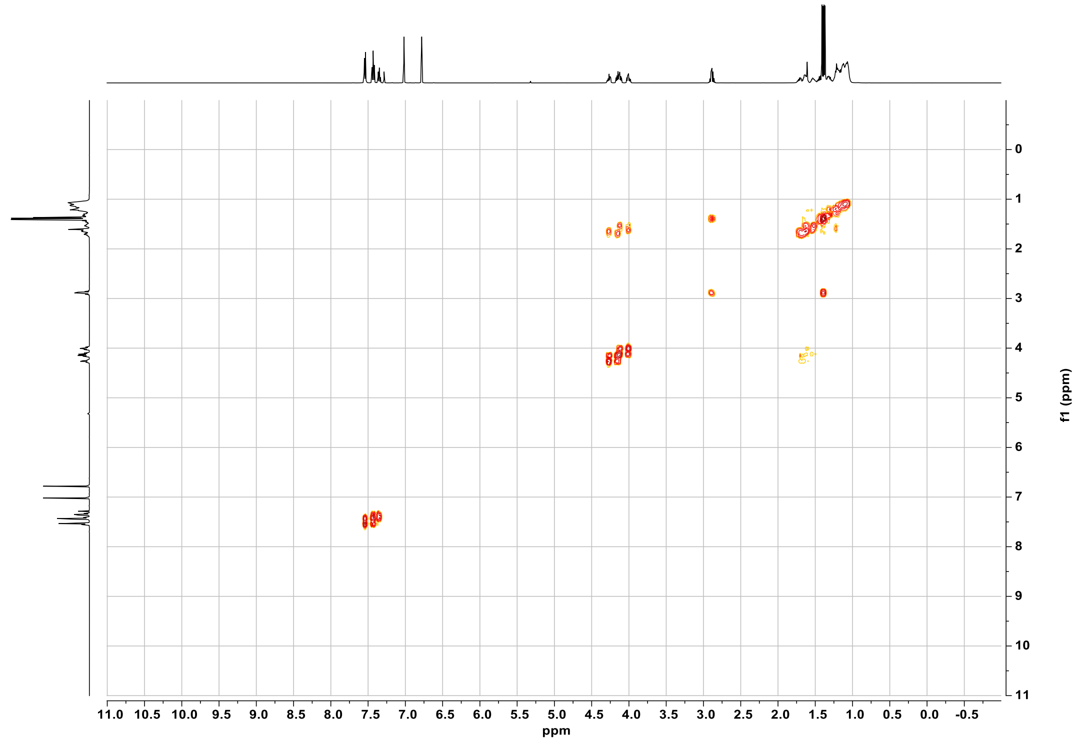

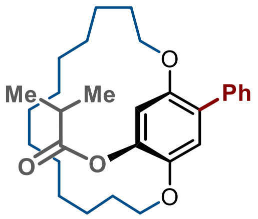

28

2D ^{1}H-^{13}C, $CDCl_3$, HSQC

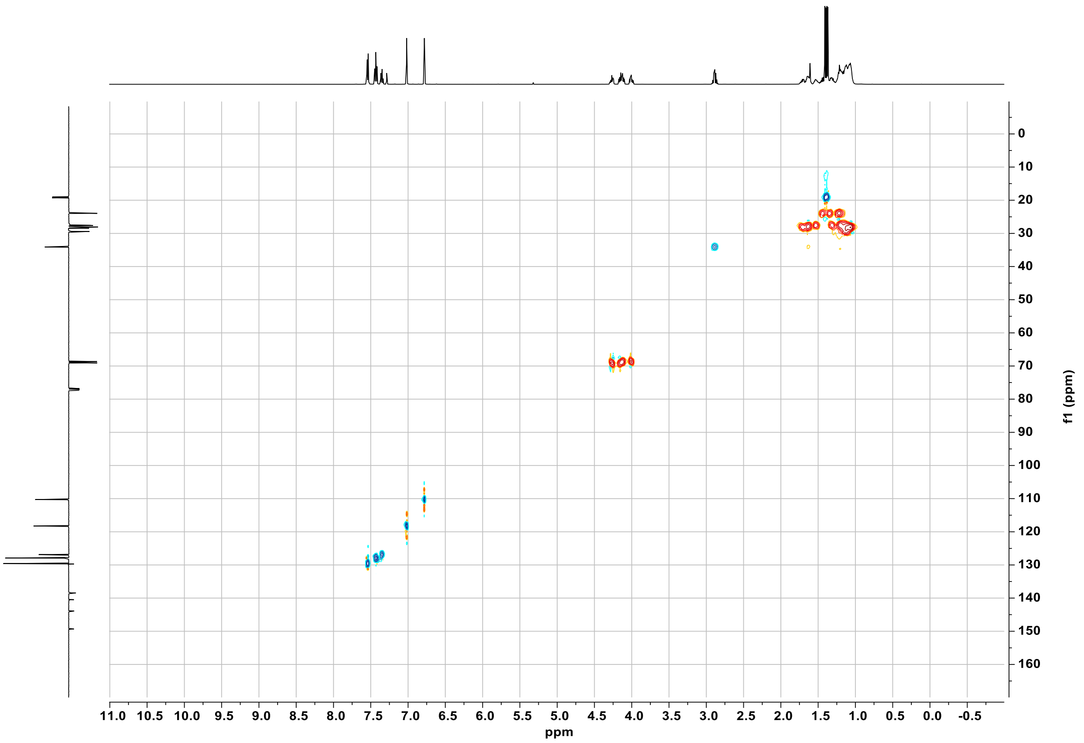

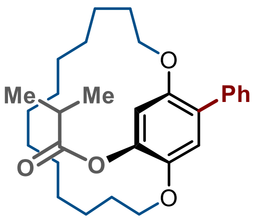

28

2D ^{1}H-^{13}C, $CDCl_3$, HMBC

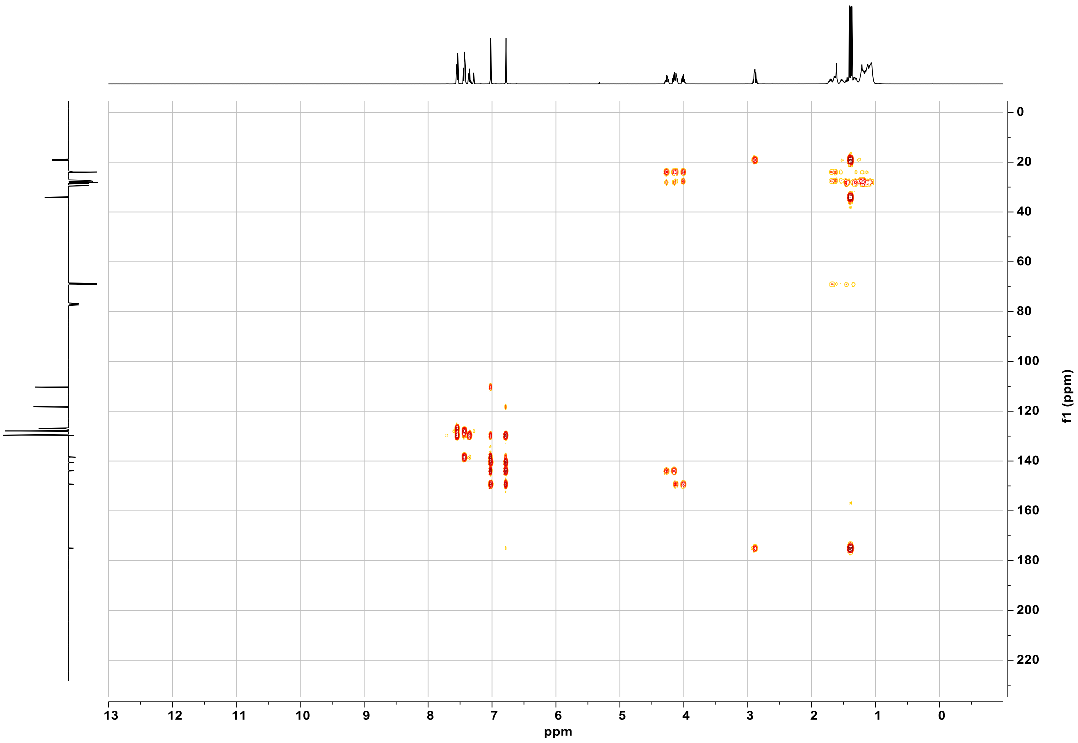

(*Rp*)-1⁵-bromo-2,17-dioxa-1(1,4)-benzenacyclohexadecaphane-1²-yl isobutyrate 29

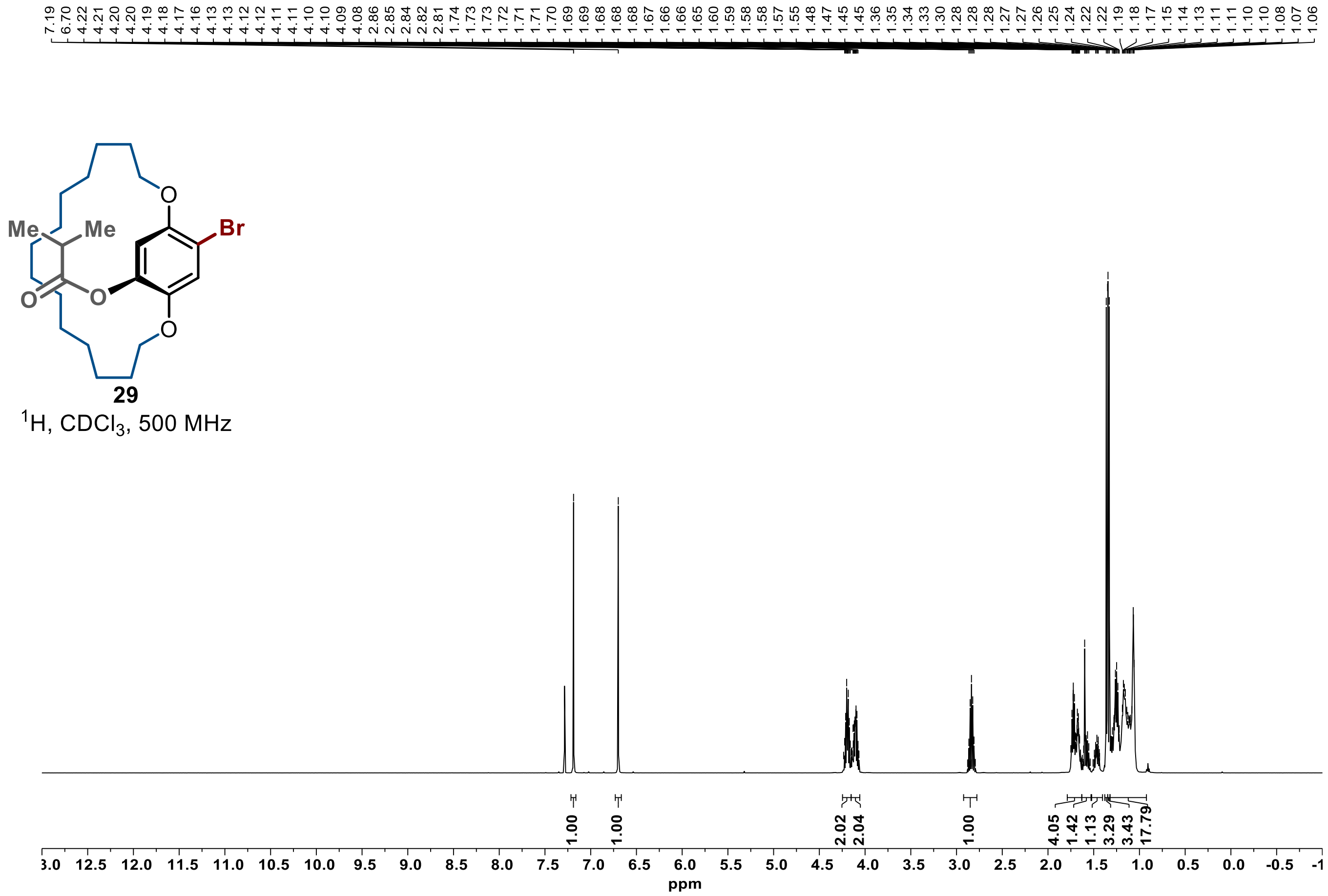

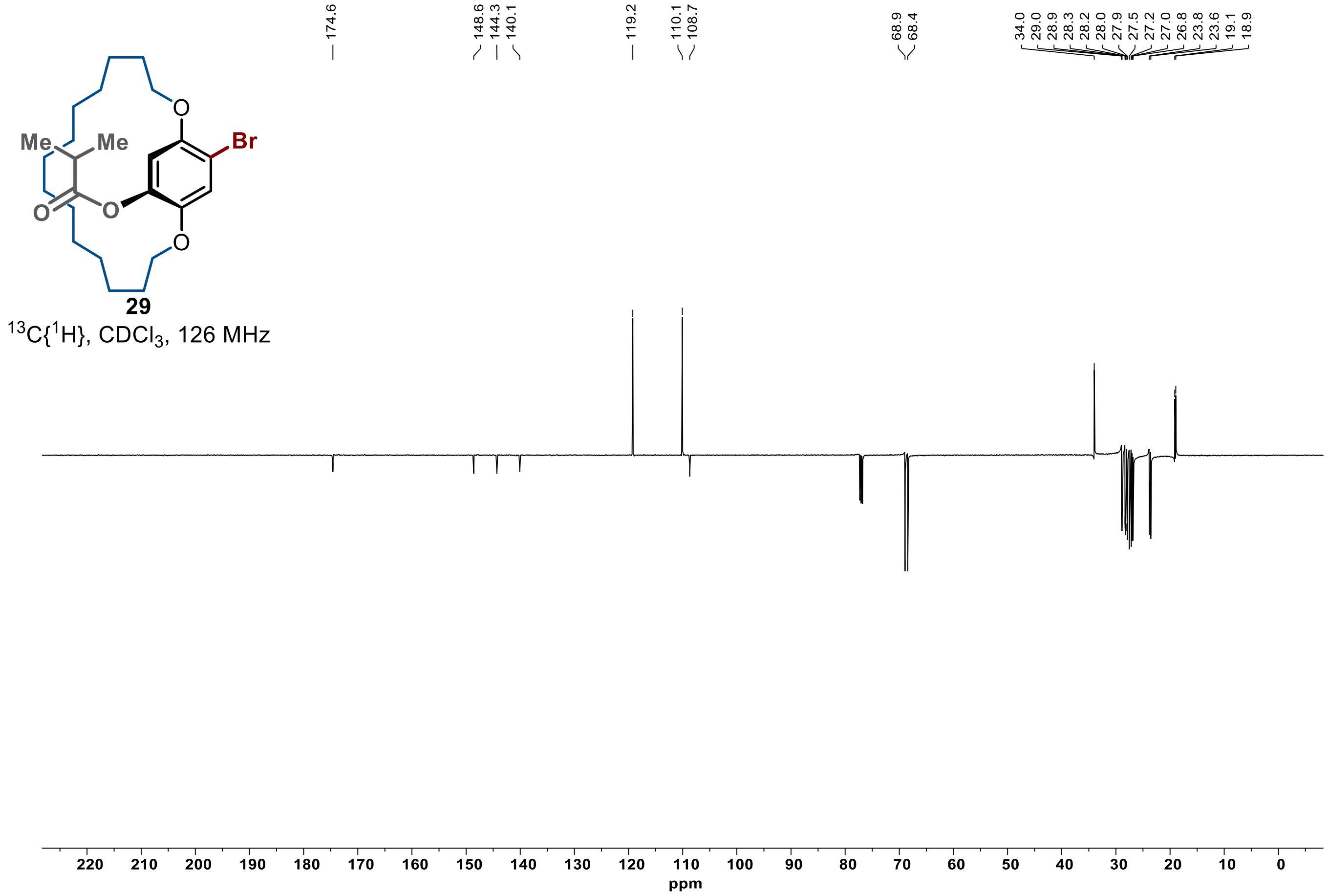

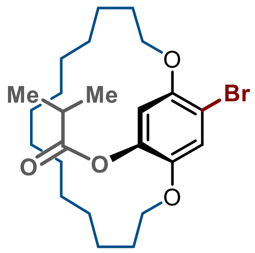

29

2D ^{1}H, $CDCl_3$, COSY

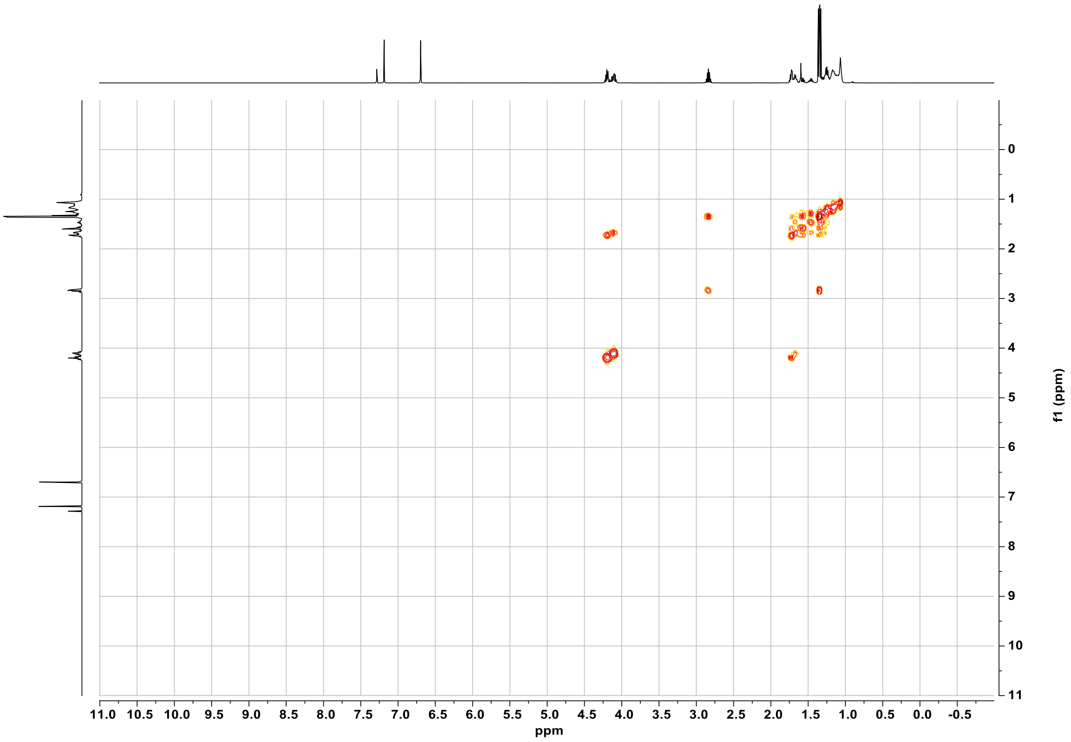

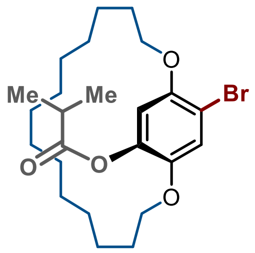

29

2D ^{1}H-^{13}C, $CDCl_3$, HSQC

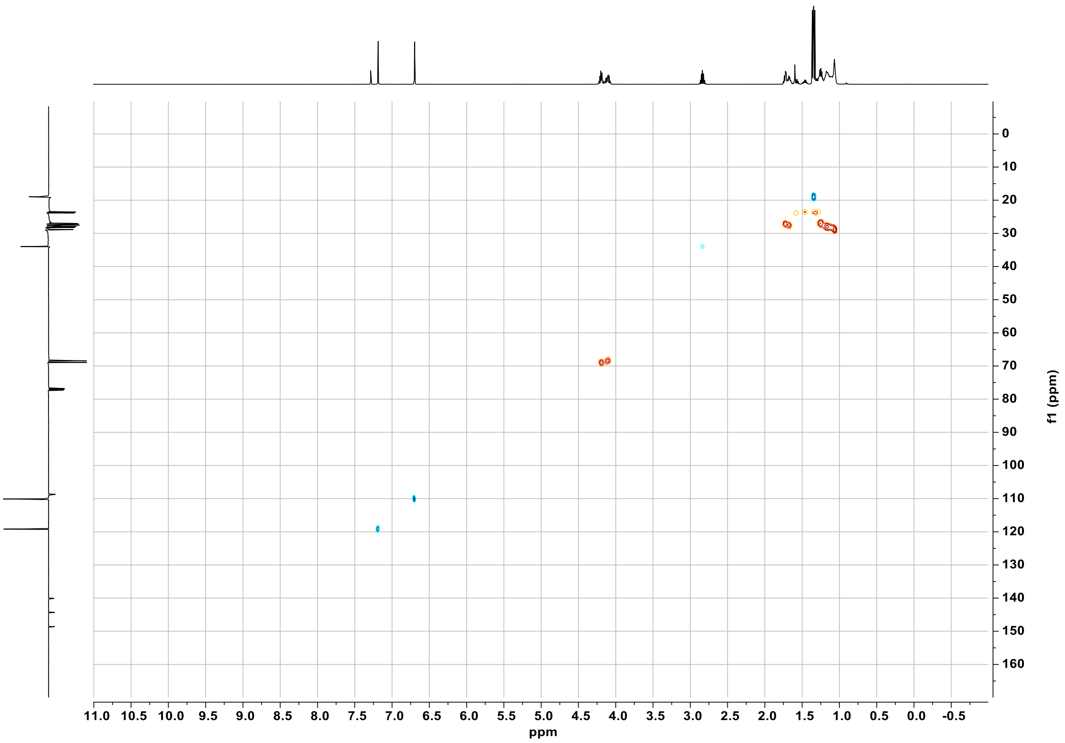

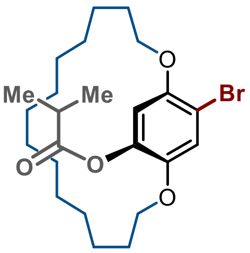

29

2D ^1H-^{13}C, $CDCl_3$, HMBC

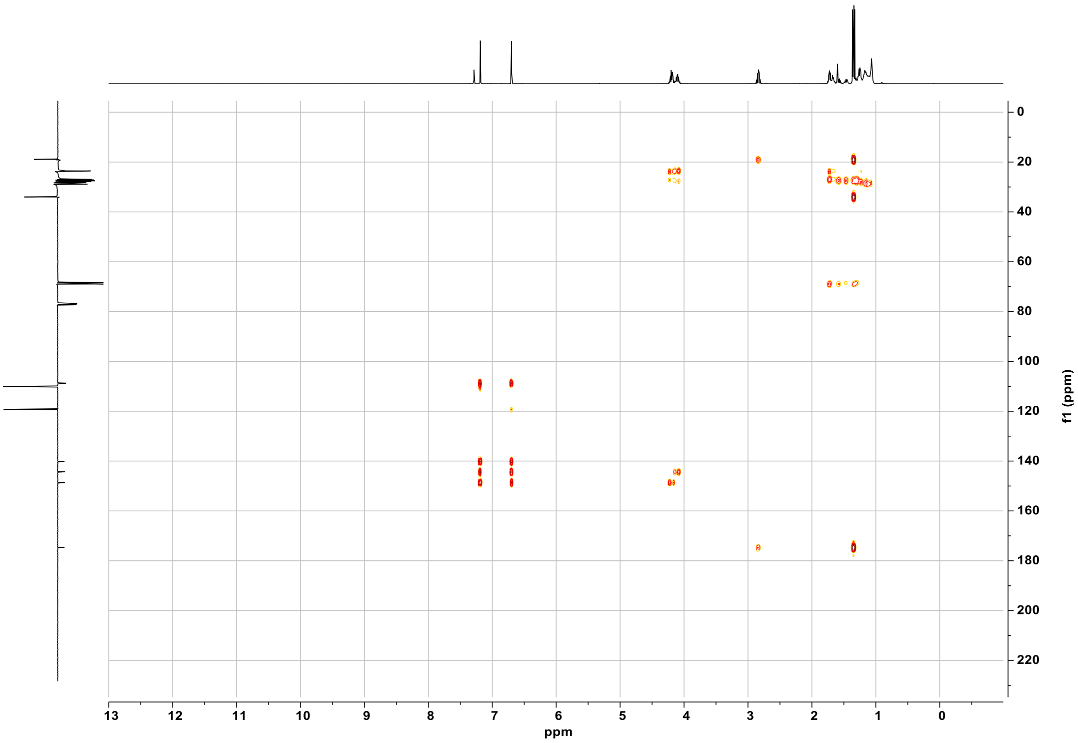

(*Rp*)-1⁵-phenyl-2,17-dioxa-1(1,4)-benzenacyclohexadecaphane-1²-yl isobutyrate 30

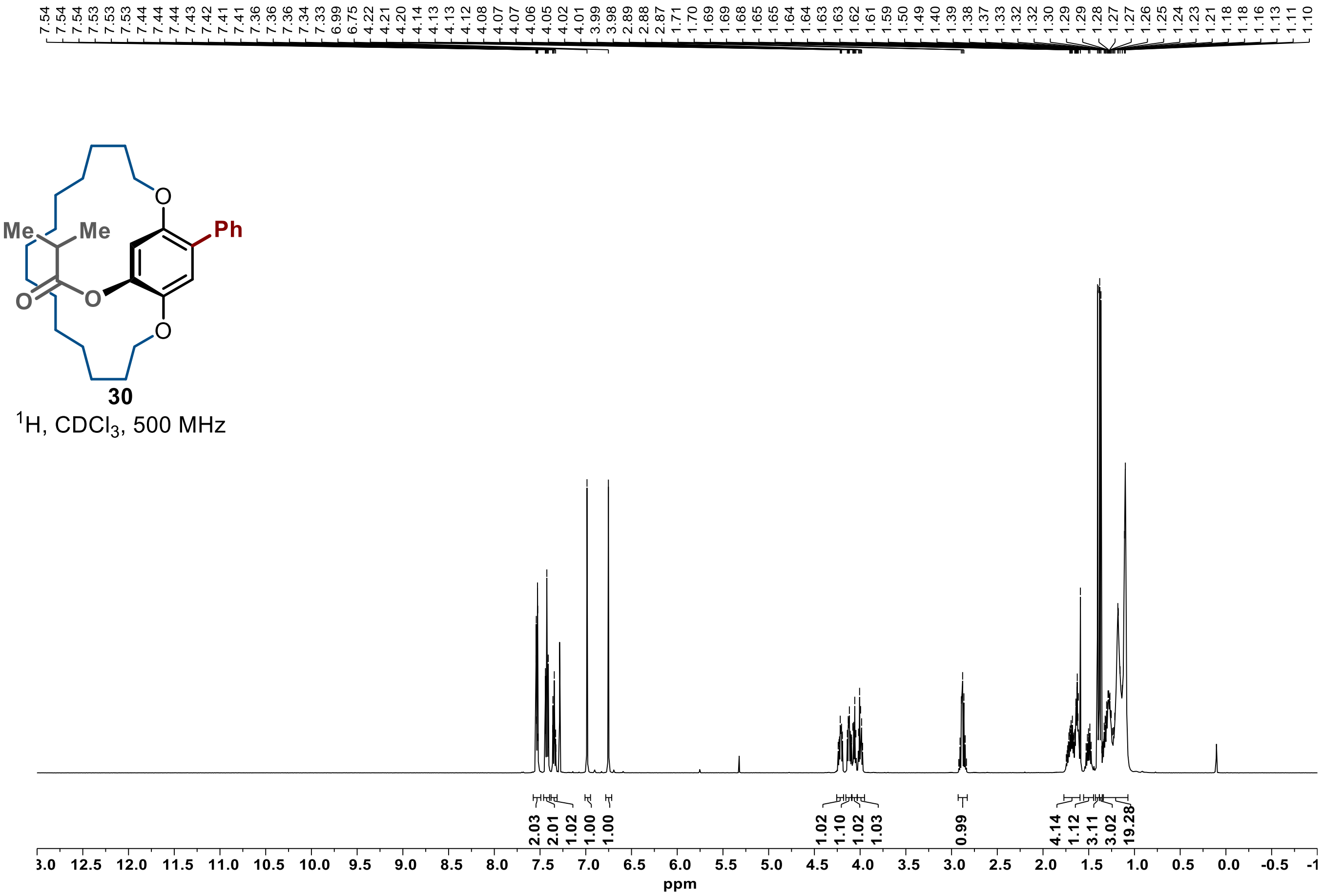

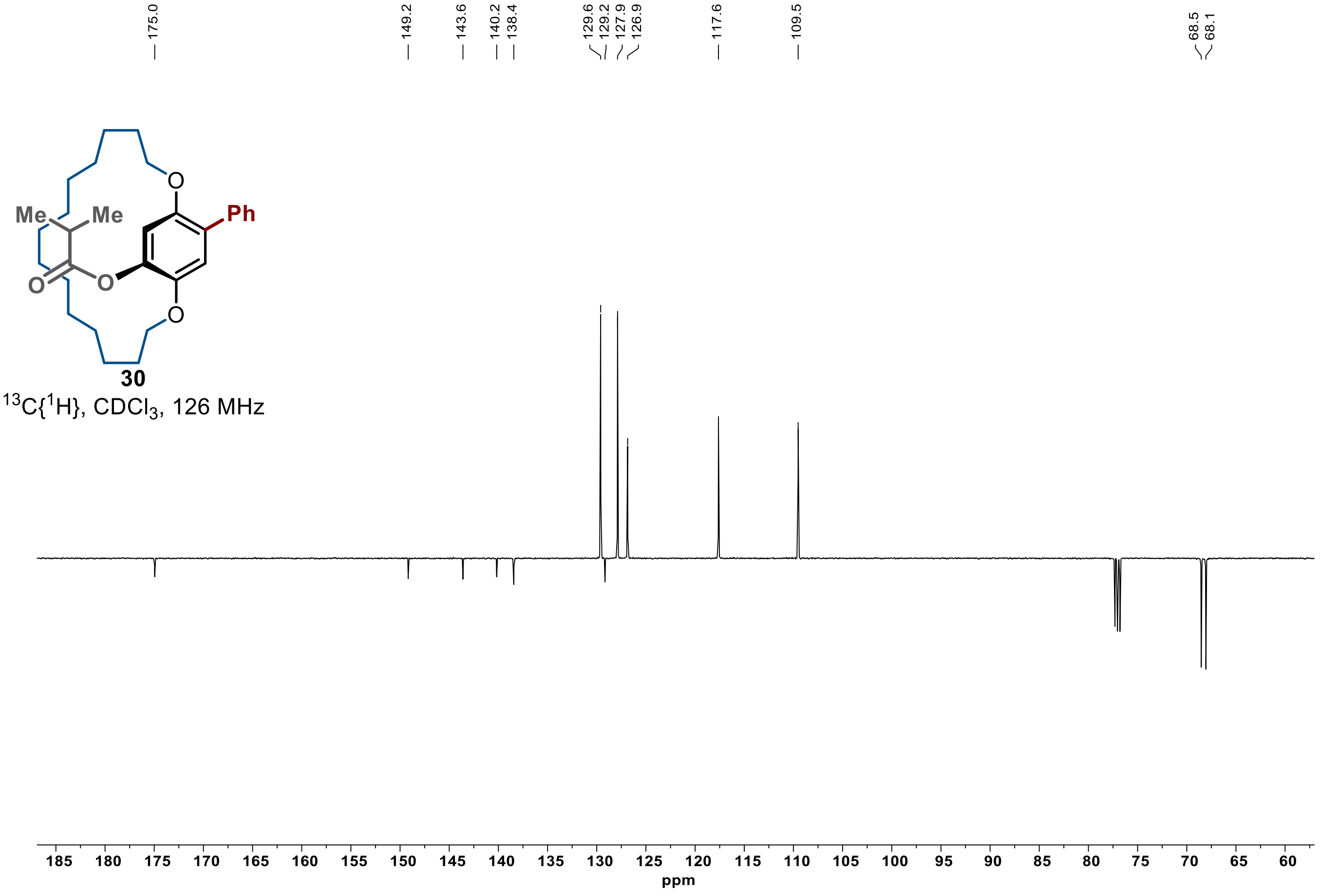

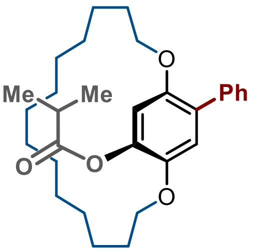

30

2D ^{1}H, $CDCl_3$, COSY

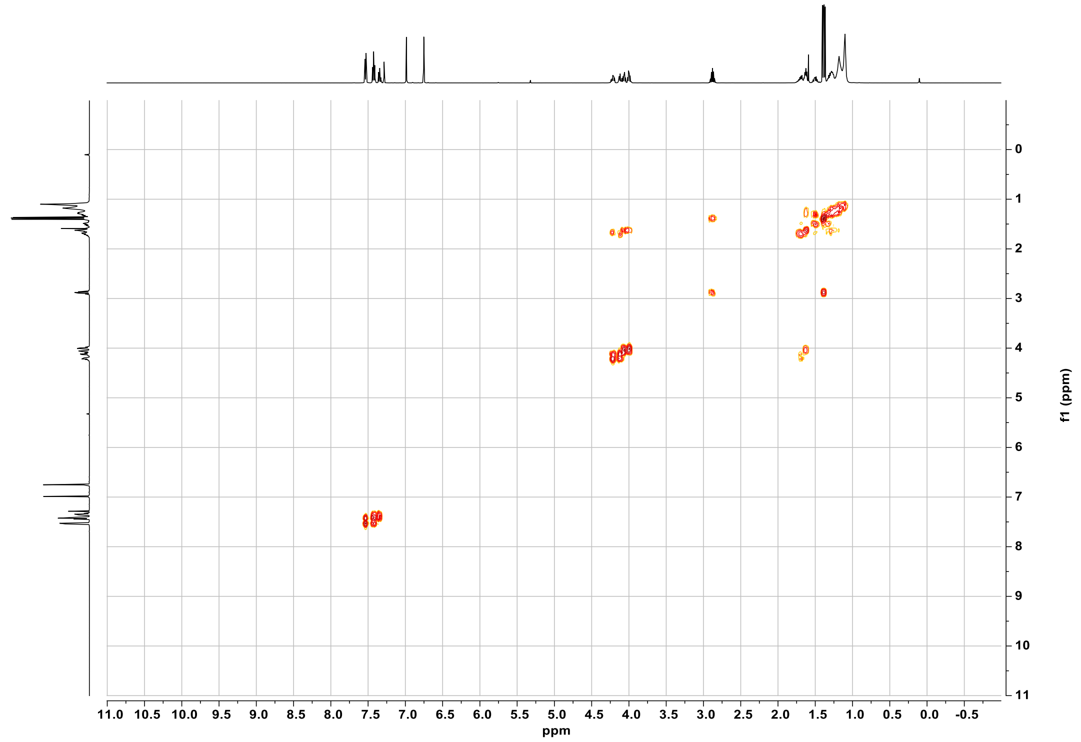

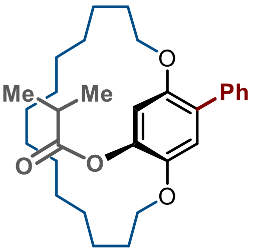

30

2D ^{1}H-^{13}C, $CDCl_3$, HSQC

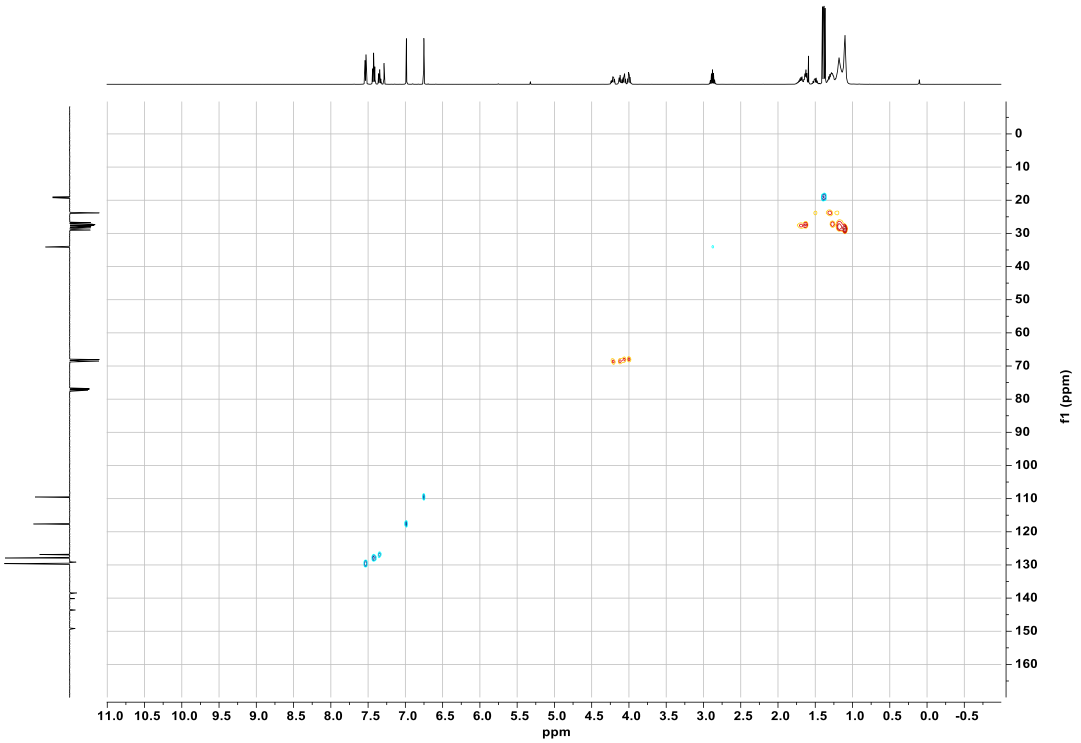

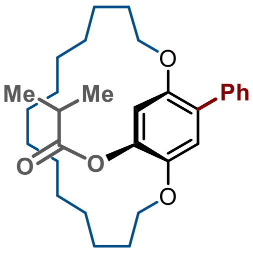

30

2D ^{1}H-^{13}C, $CDCl_3$, HMBC

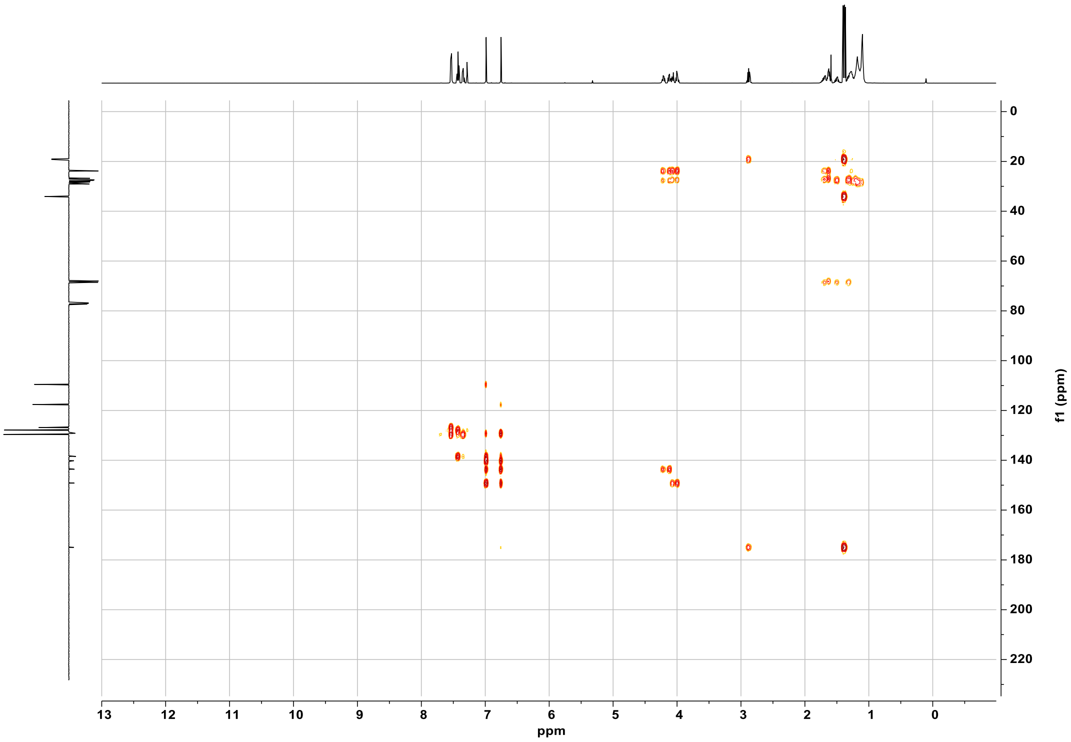

(*Rp*)-1⁵-bromo-4,15-dioxo-2,17-dioxa-5,14-diaza-1(1,4)-benzenacycloheptadecaphane-1²-yl isobutyrate 31

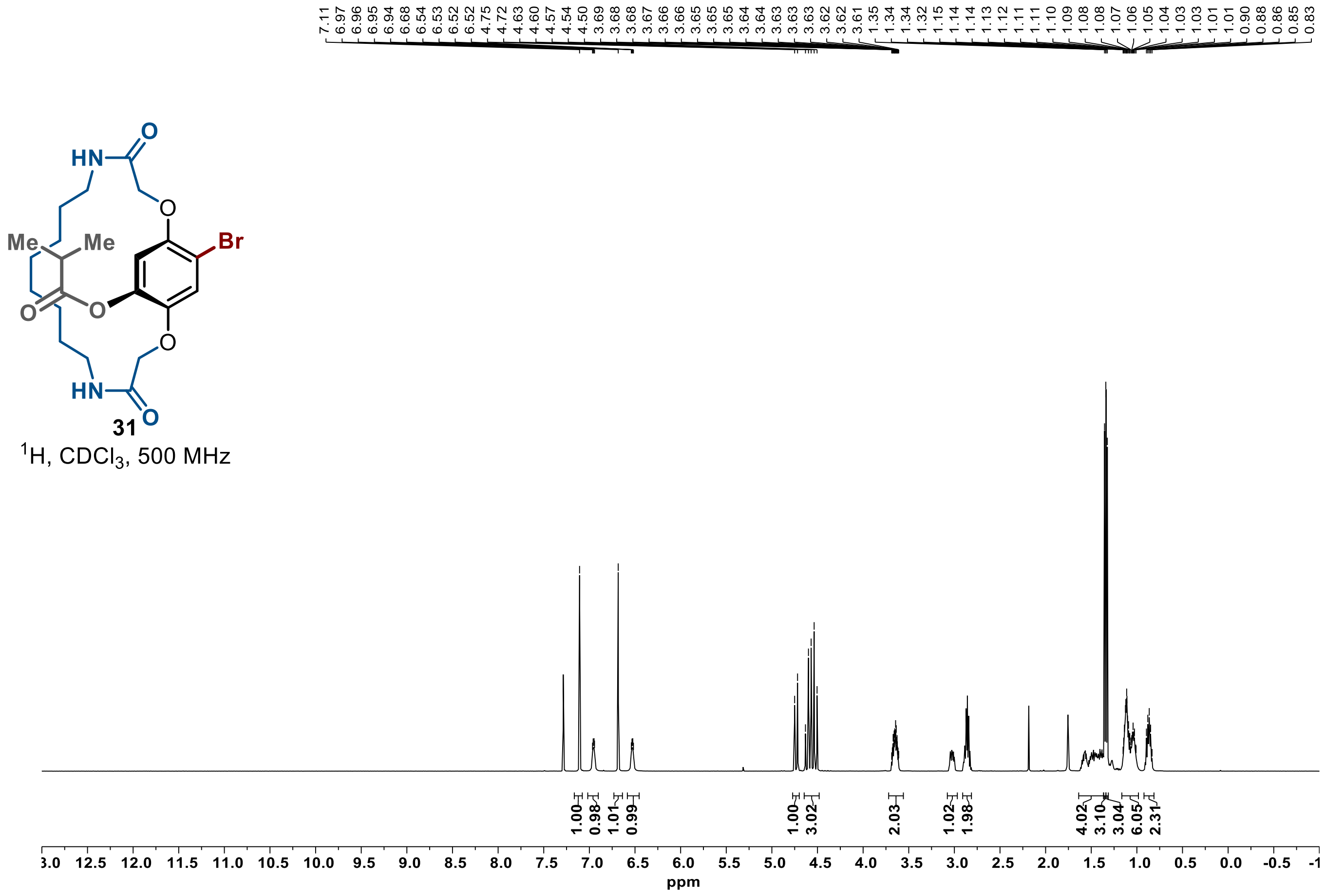

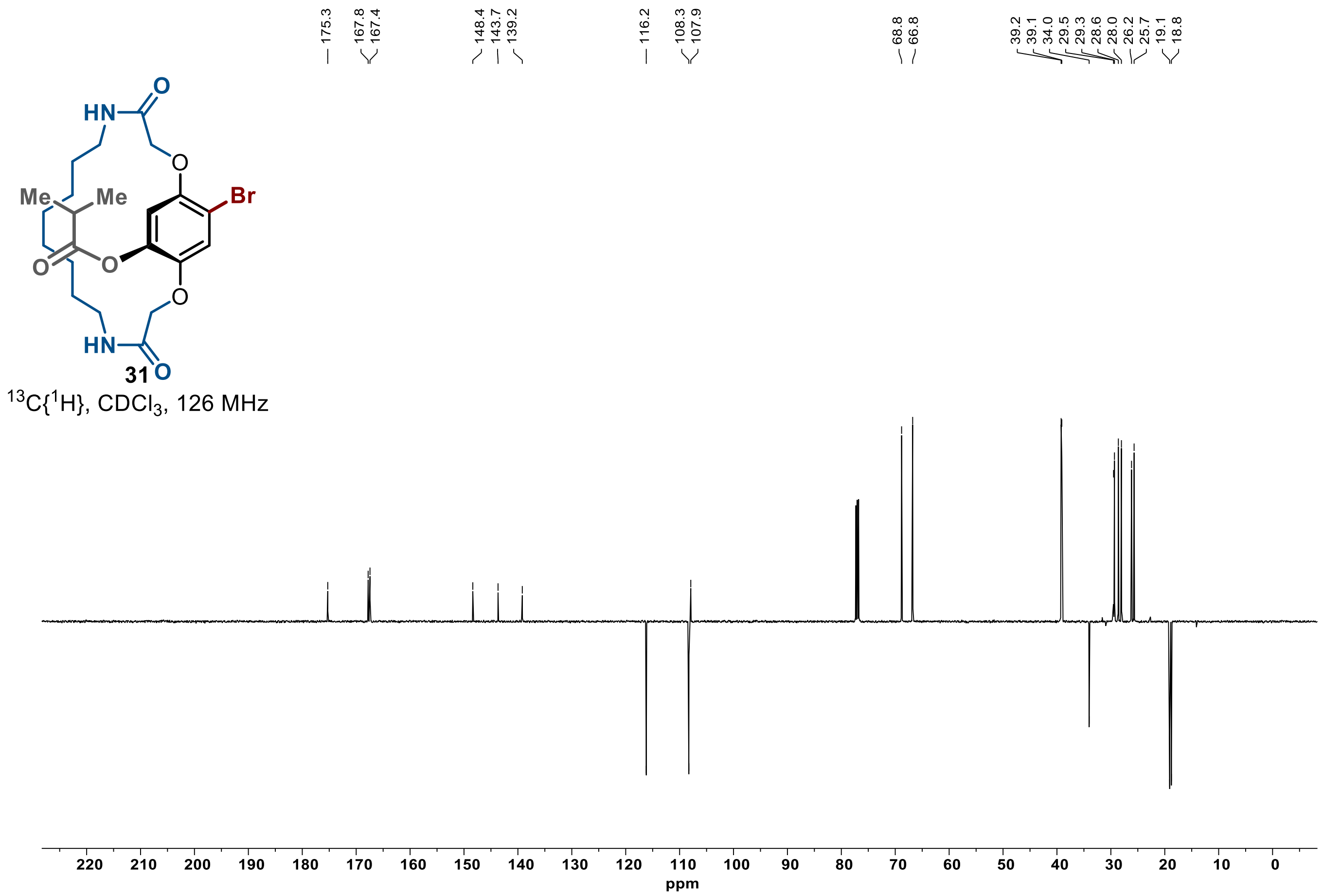

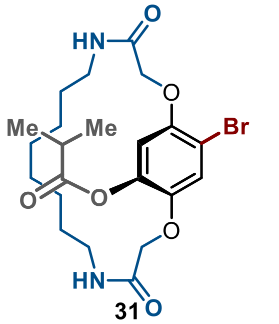

31

2D ^{1}H, $CDCl_3$, COSY

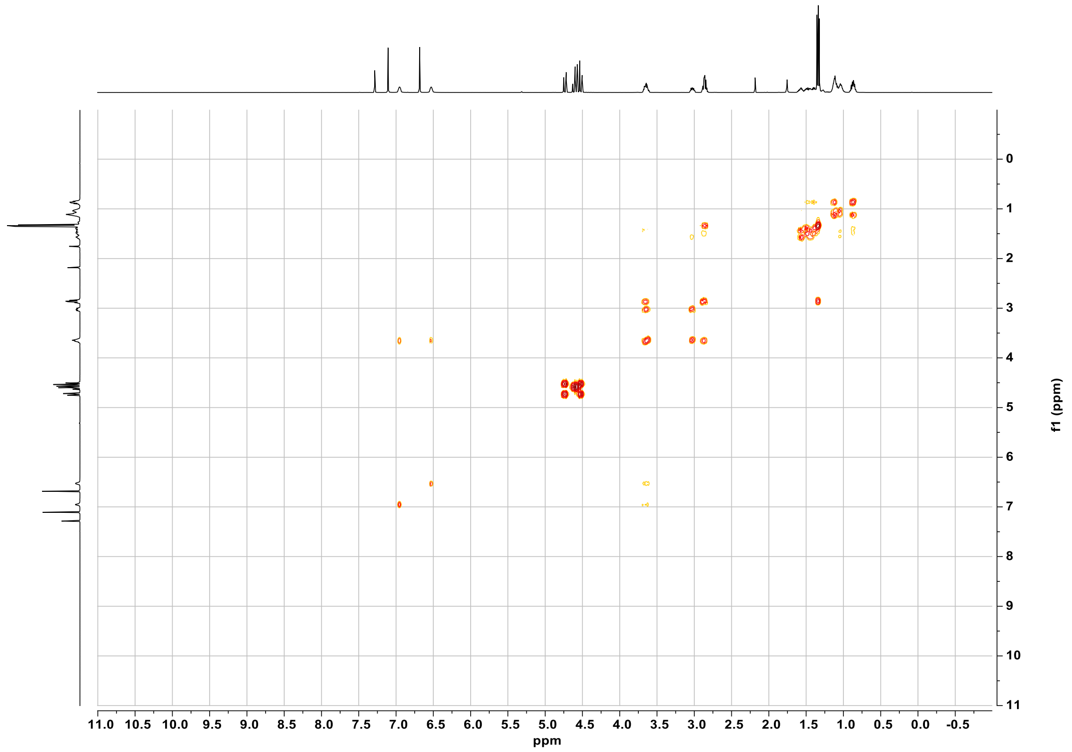

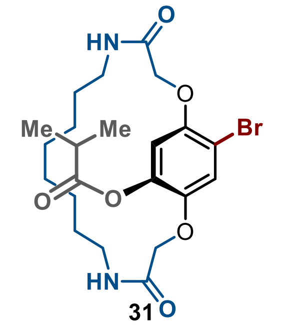

31

2D ^{1}H-^{13}C, $CDCl_3$, HSQC

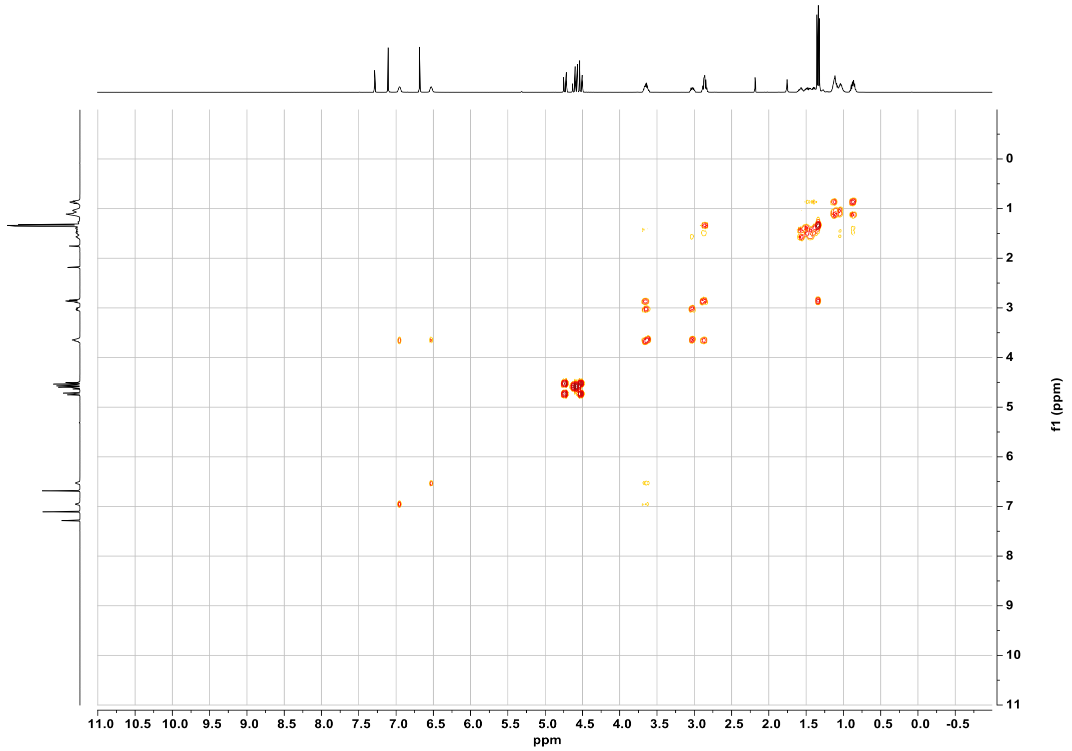

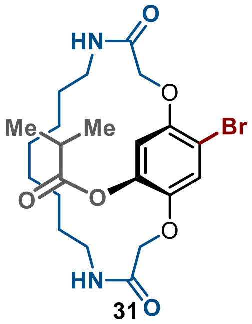

31

2D ^{1}H-^{13}C, $CDCl_3$, HMBC

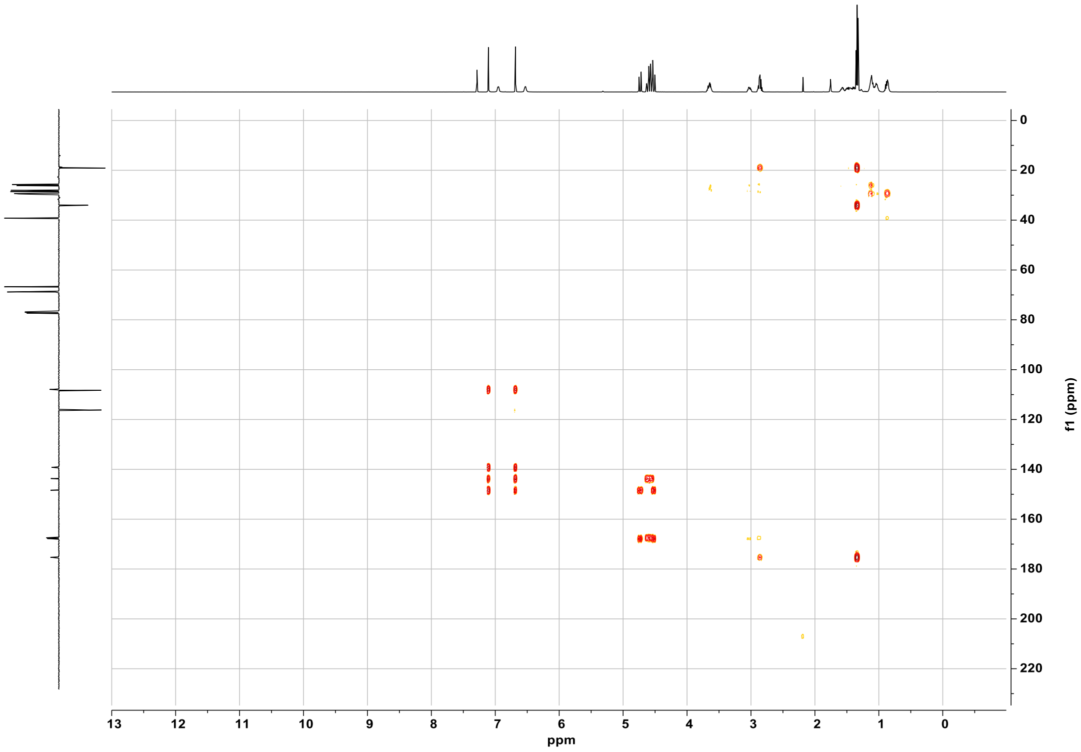

(*Rp*)-4,15-dioxo-1⁵-phenyl-2,17-dioxa-5,14-diaza-1(1,4)-benzenacycloheptadecaphane-1²-yl isobutyrate 32

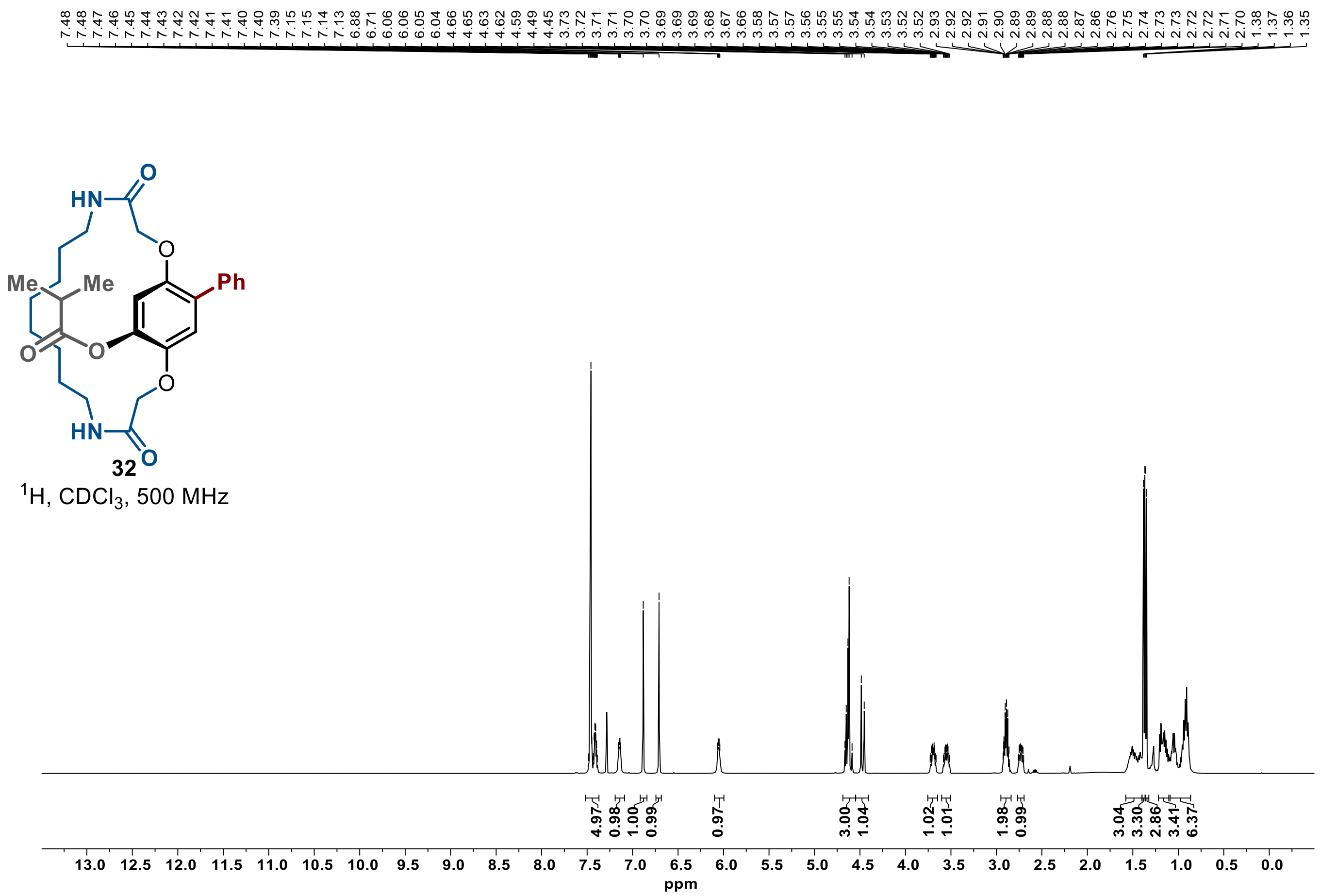

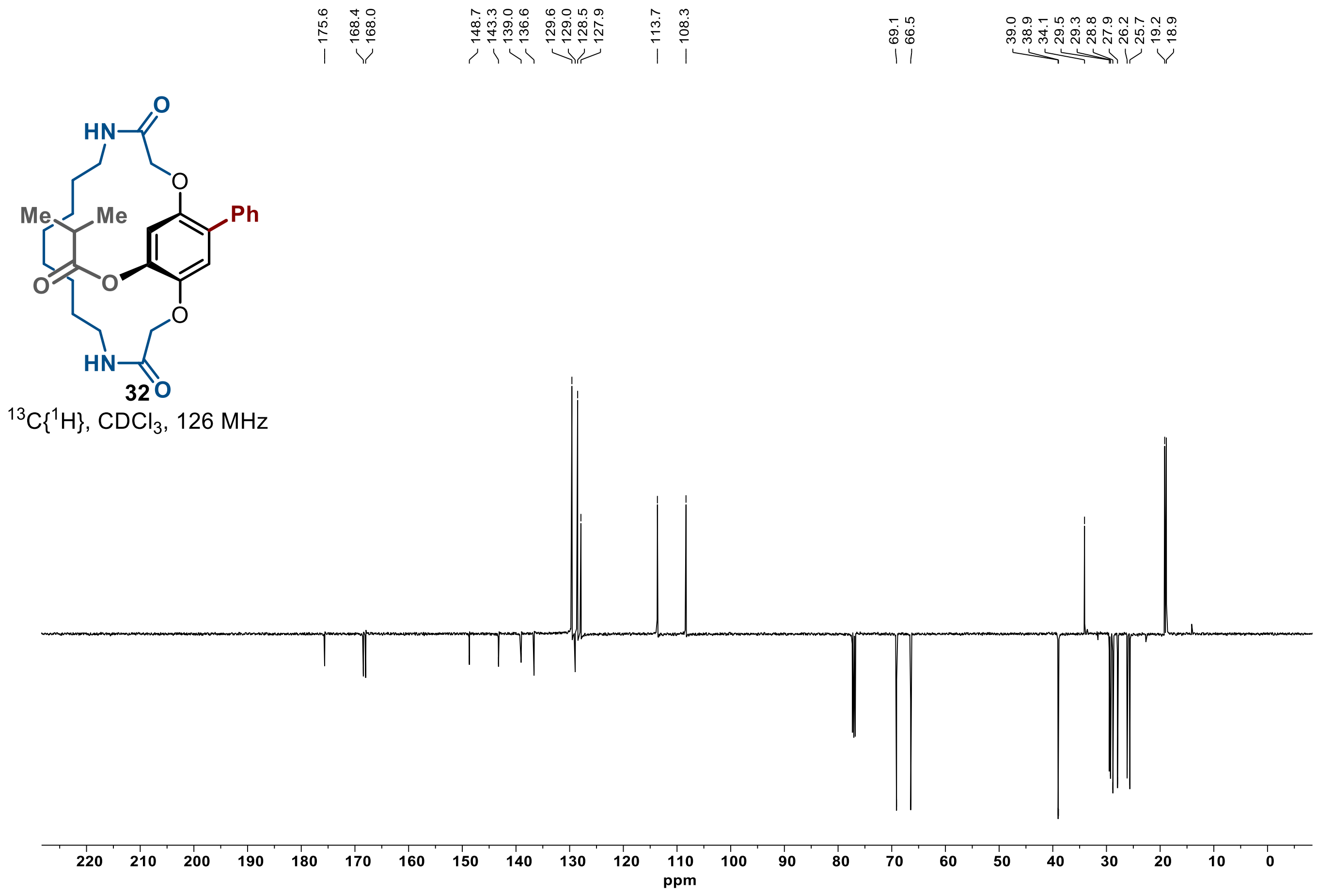

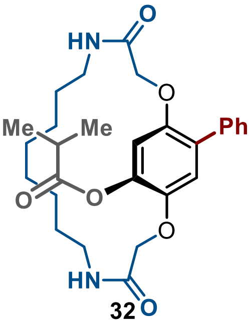

2D ^{1}H, $CDCl_3$, COSY

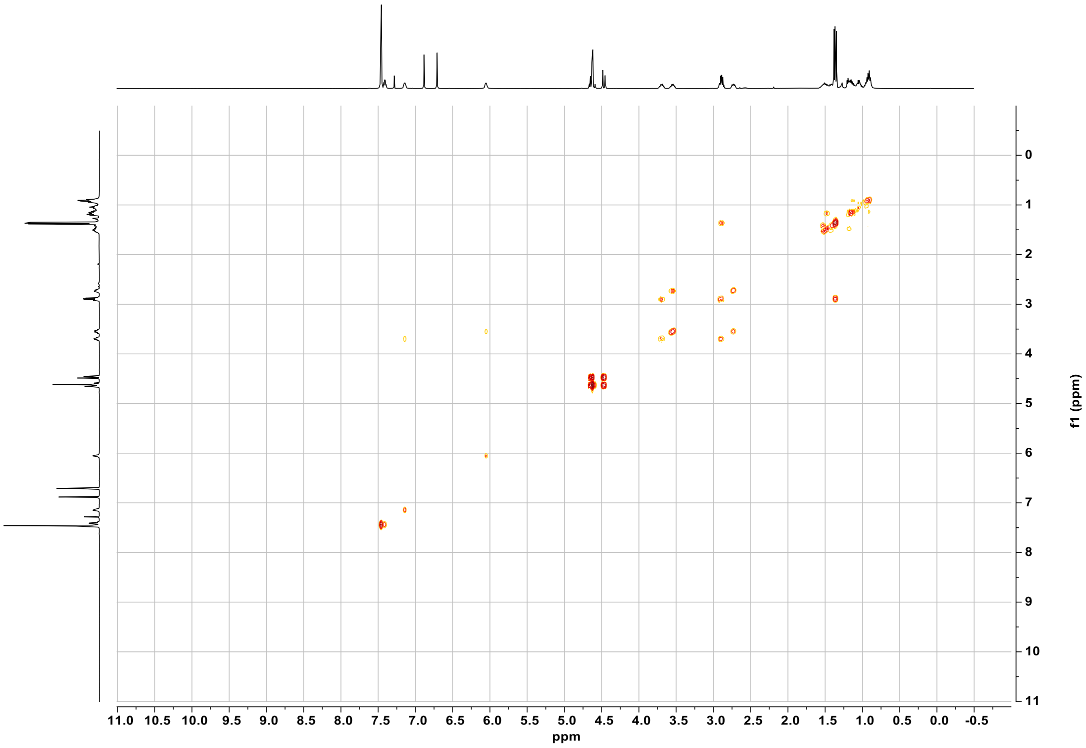

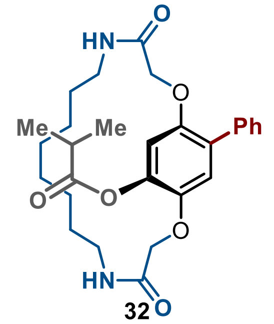

2D ^{1}H-^{13}C, $CDCl_3$, HSQC

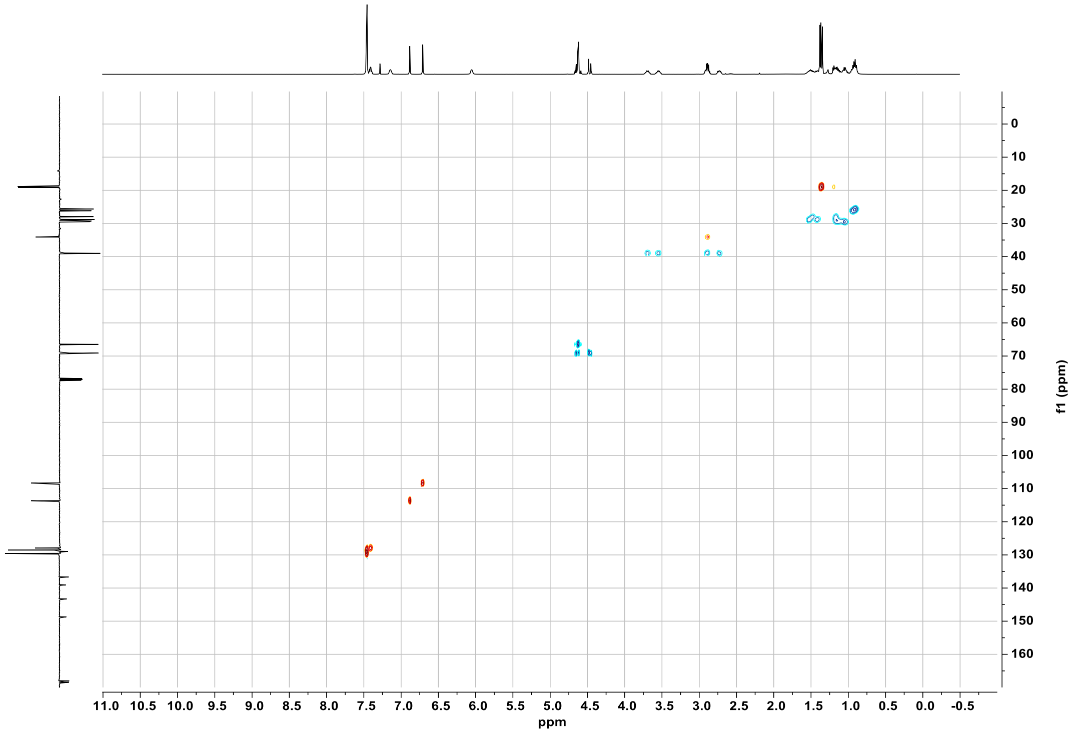

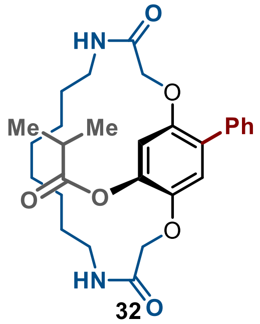

32

2D ^{1}H-^{13}C, $CDCl_3$, HMBC

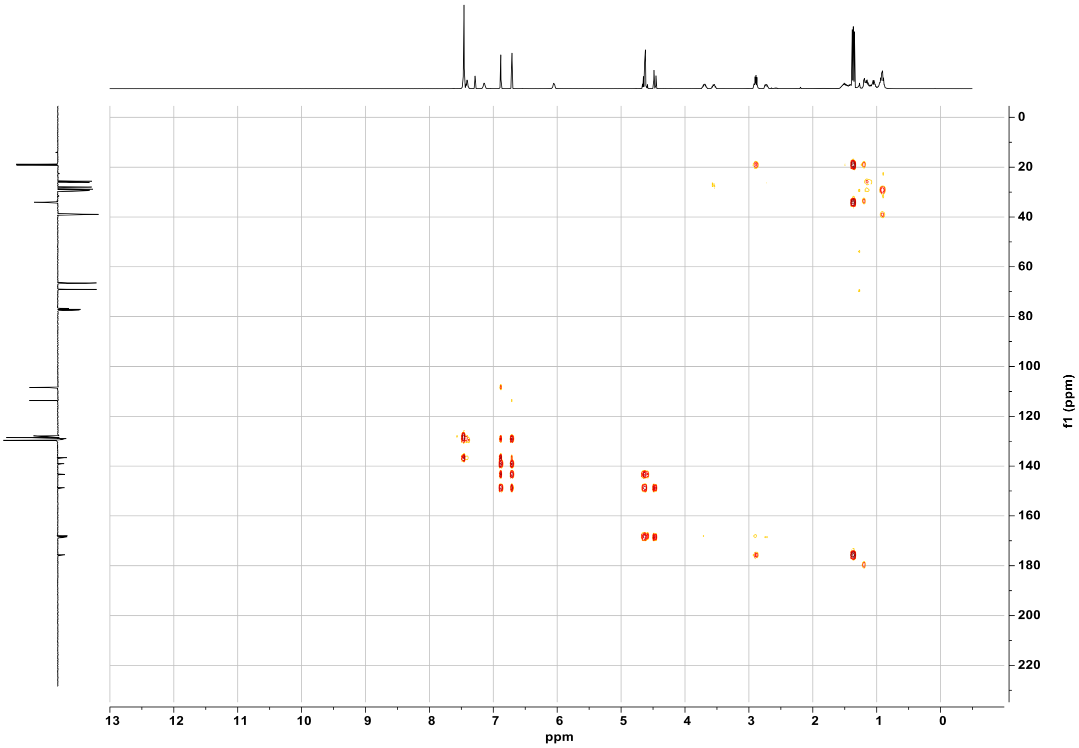

(*Rp*)-4,14-dioxo-1[5]-phenyl-2,8,10,16-tetraoxa-5,13-diaza-1(1,2),9(1,4)-dibenzenacyclohexadecaphane-1[2]-yl isobutyrate 33

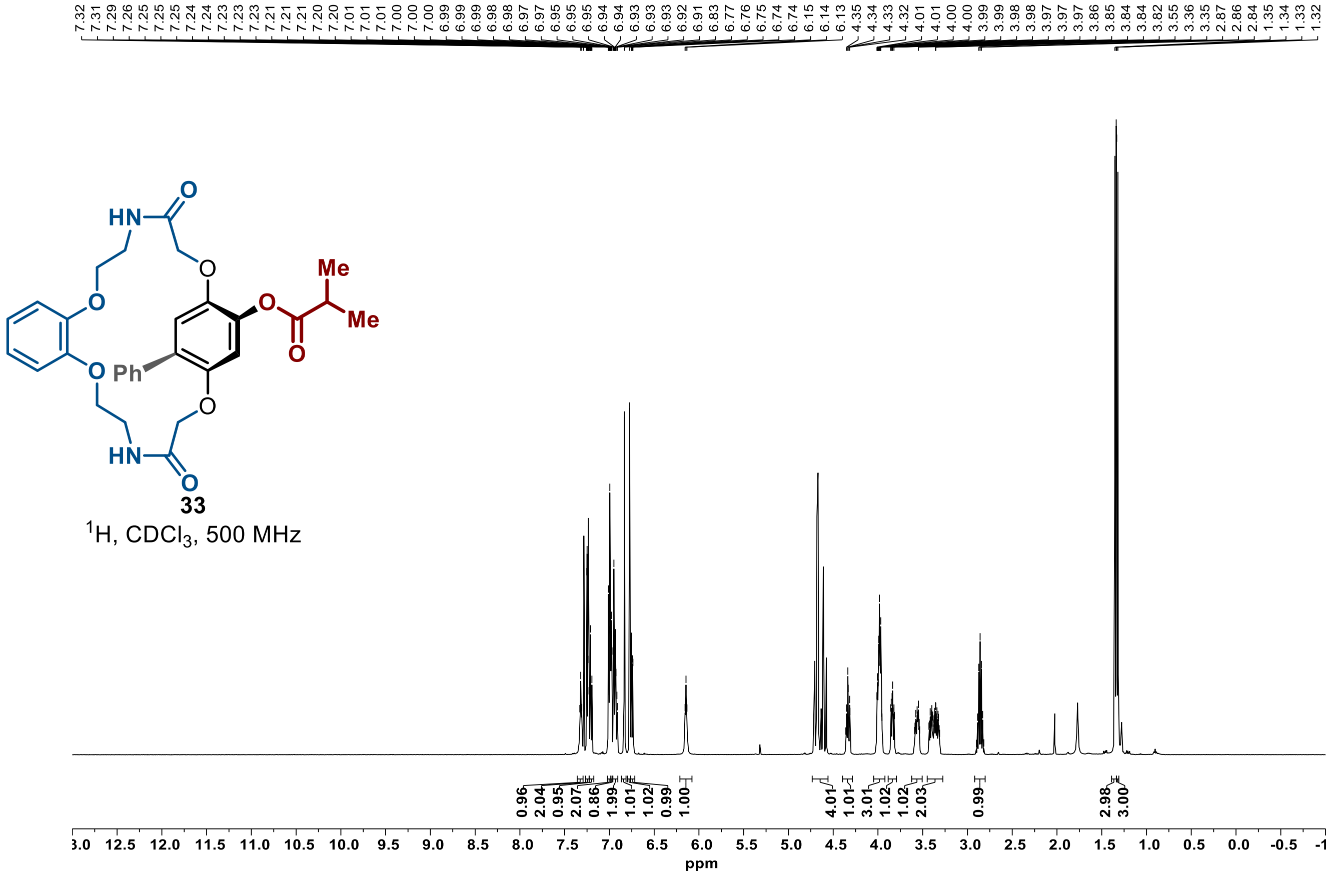

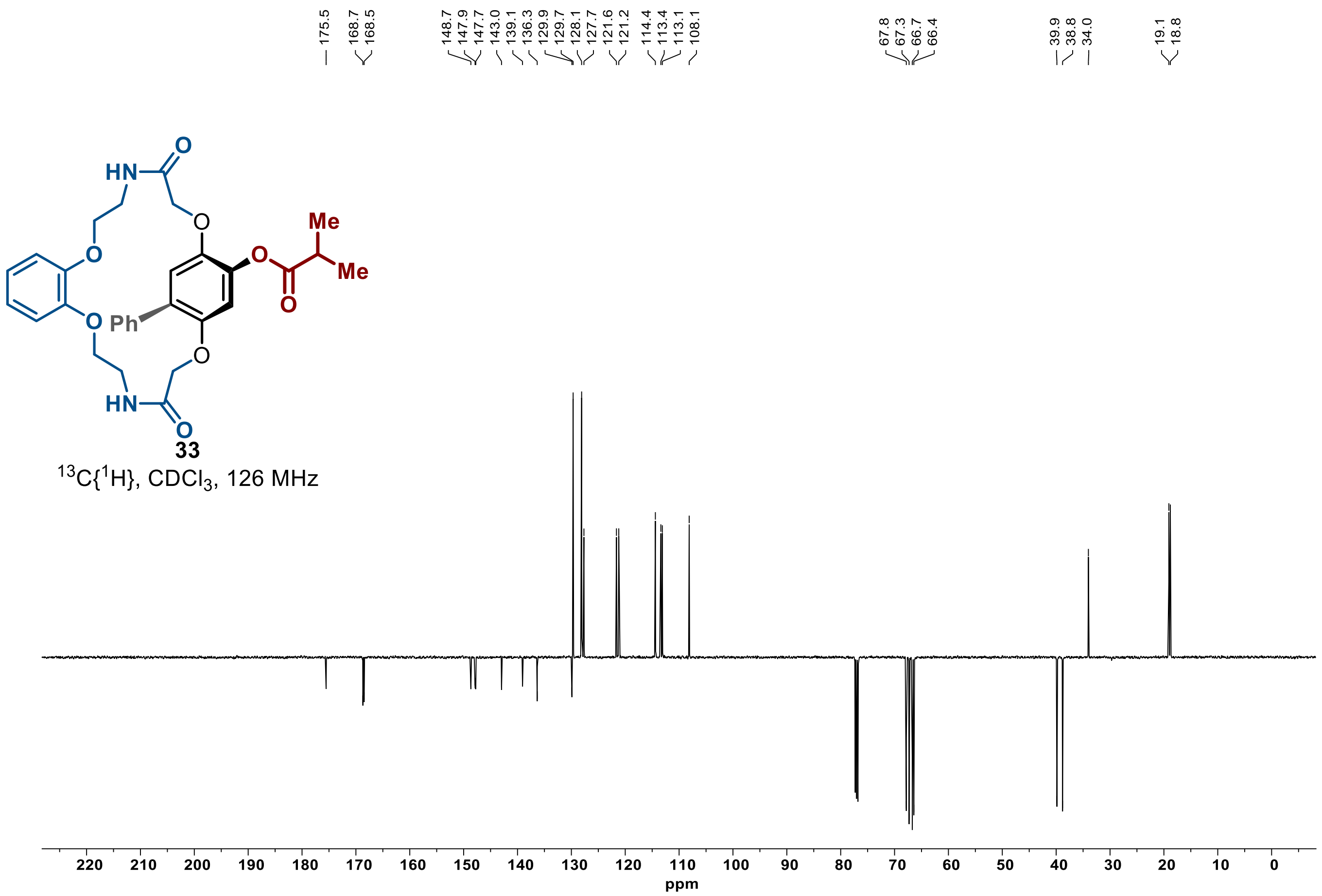

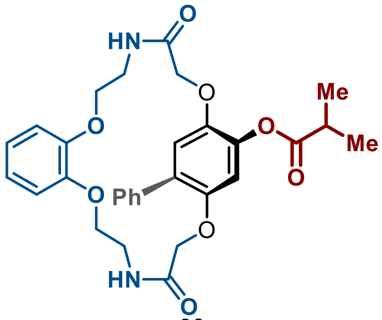

33

2D ^{1}H, $CDCl_3$, COSY

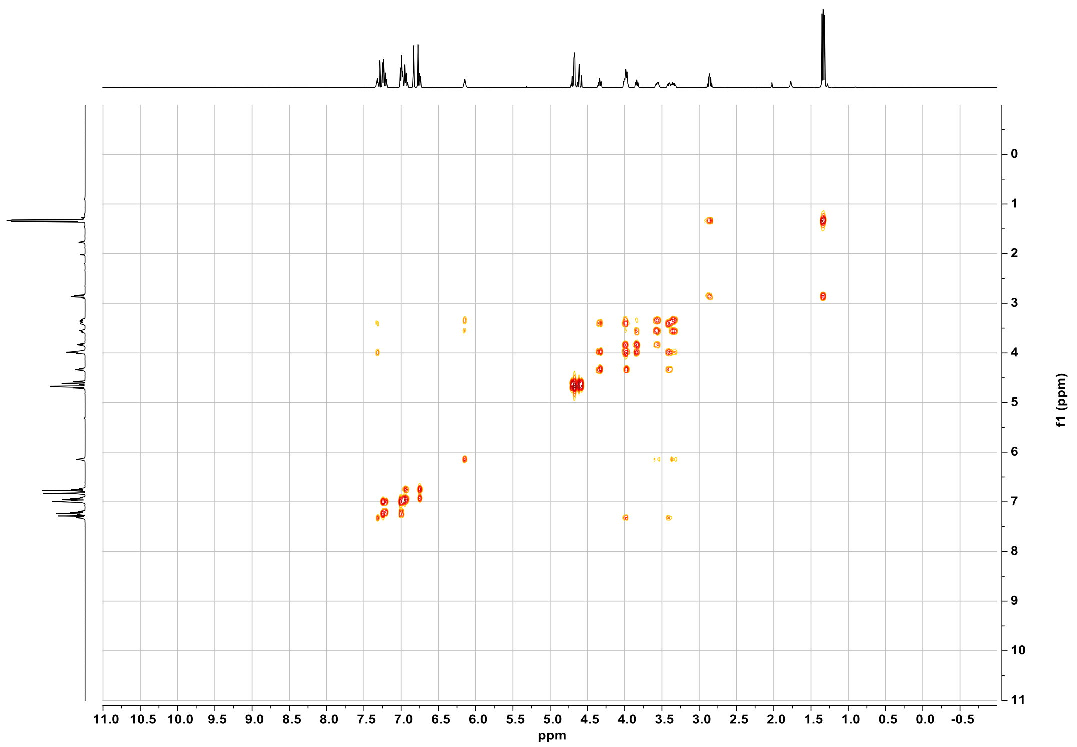

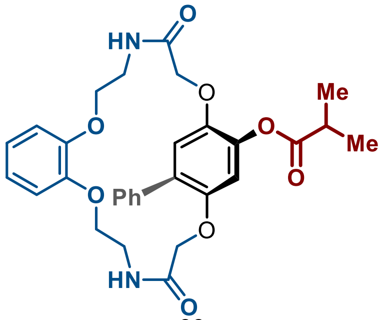

33

2D ^{1}H-^{13}C, $CDCl_3$, HSQC

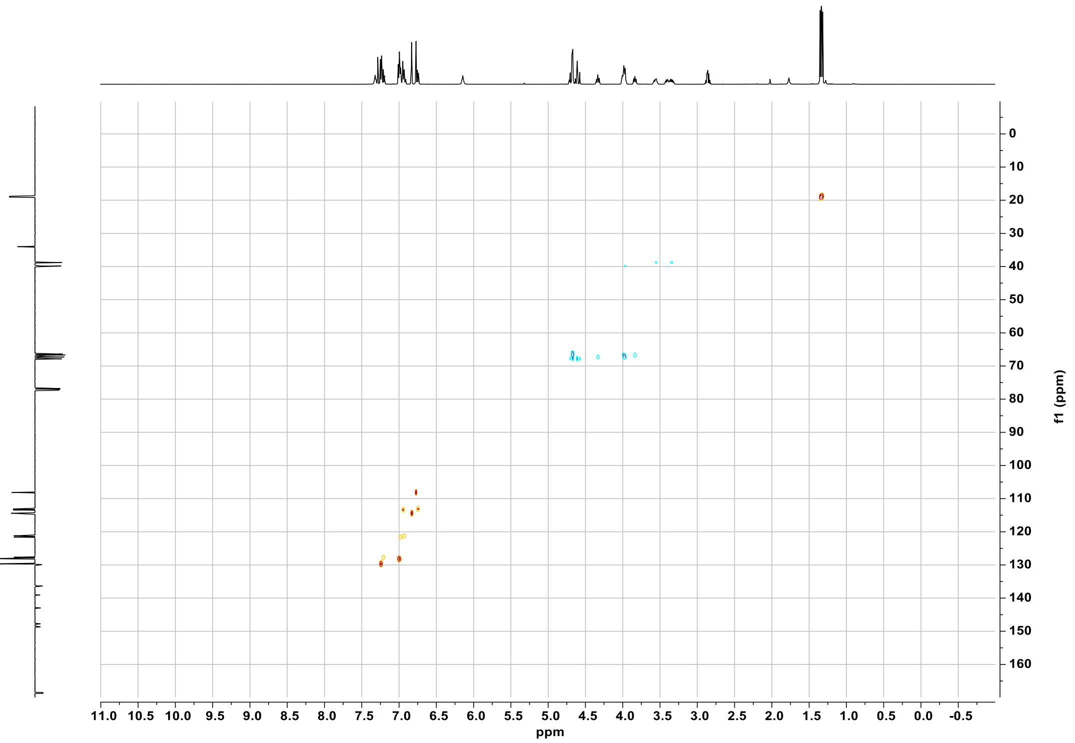

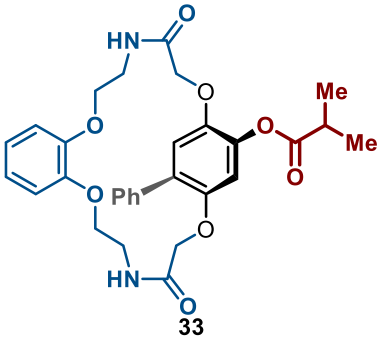

33

2D ^{1}H-^{13}C, $CDCl_3$, HMBC

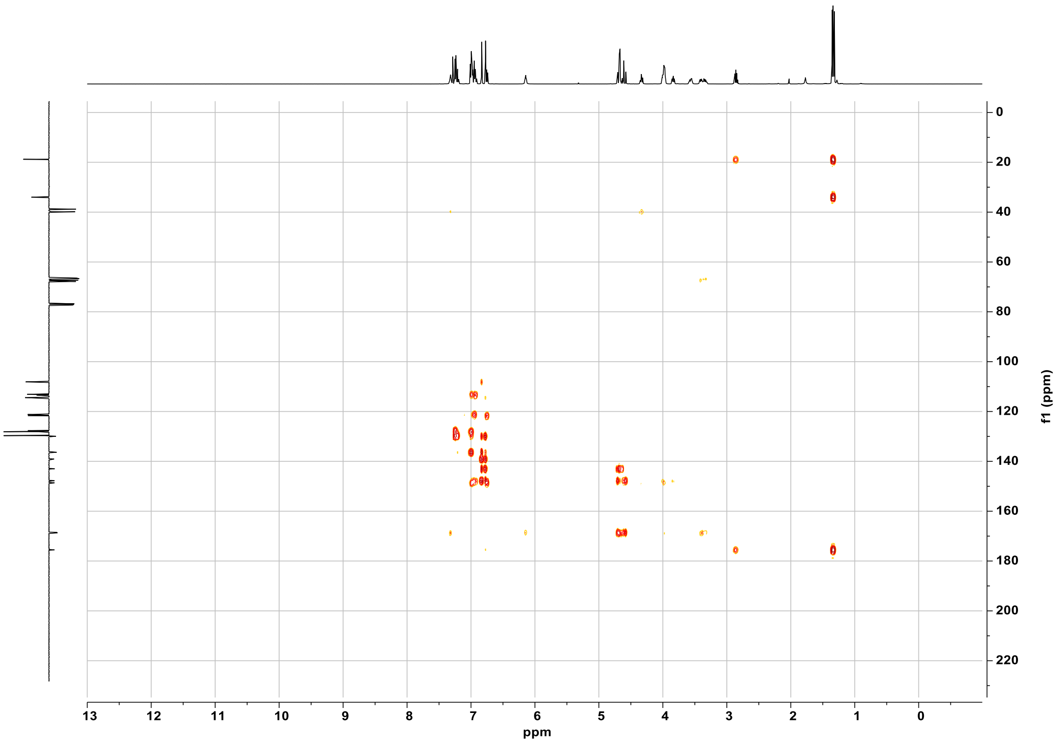

(*Rp*)-1⁵-phenyl-2,18-dioxa-1(1,4)-benzenacyclohexadecaphane-1²-yl isobutyrate 34

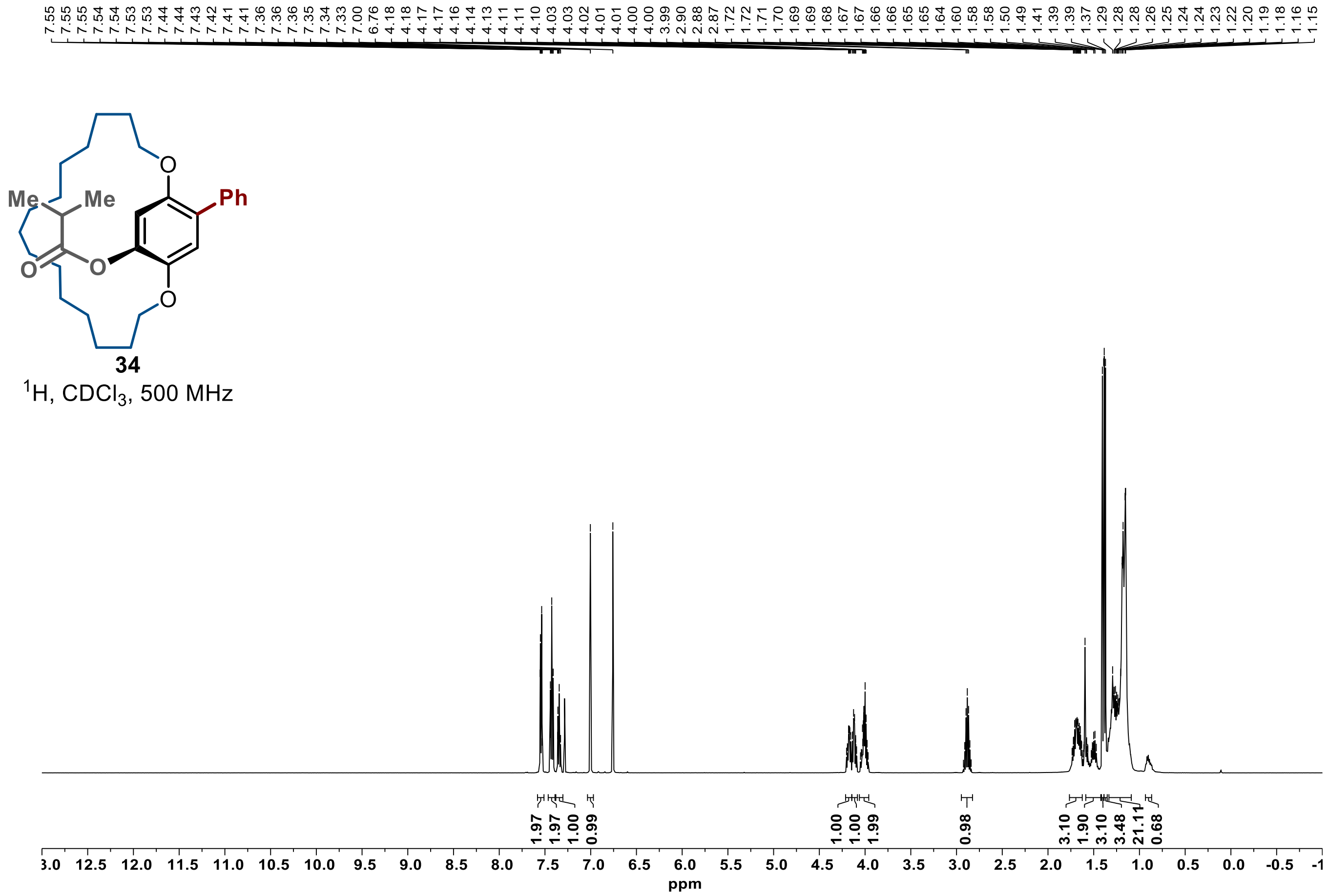

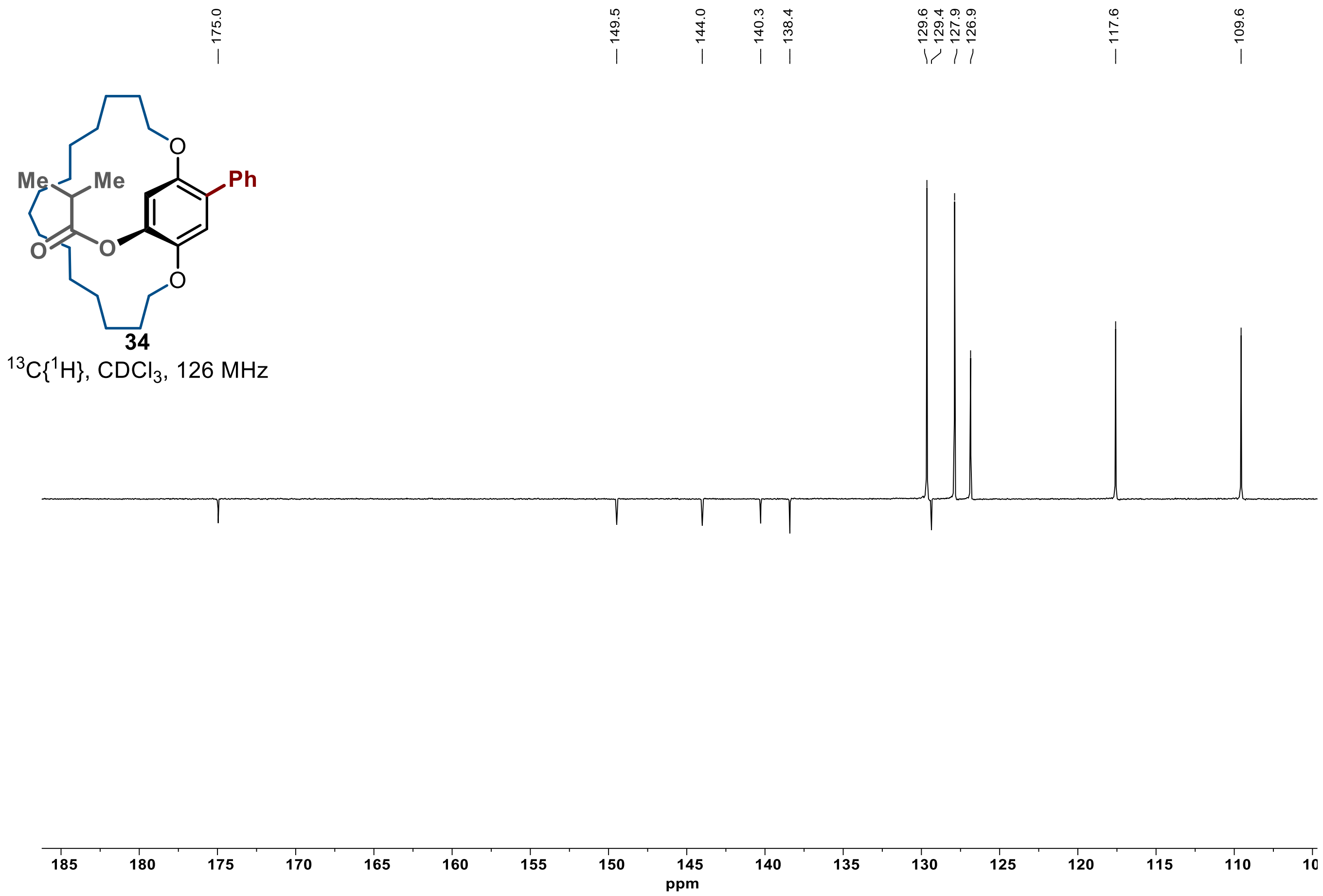

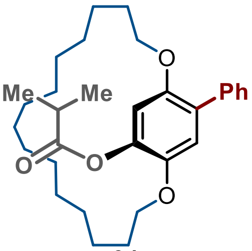

34

2D ^{1}H, $CDCl_3$, COSY

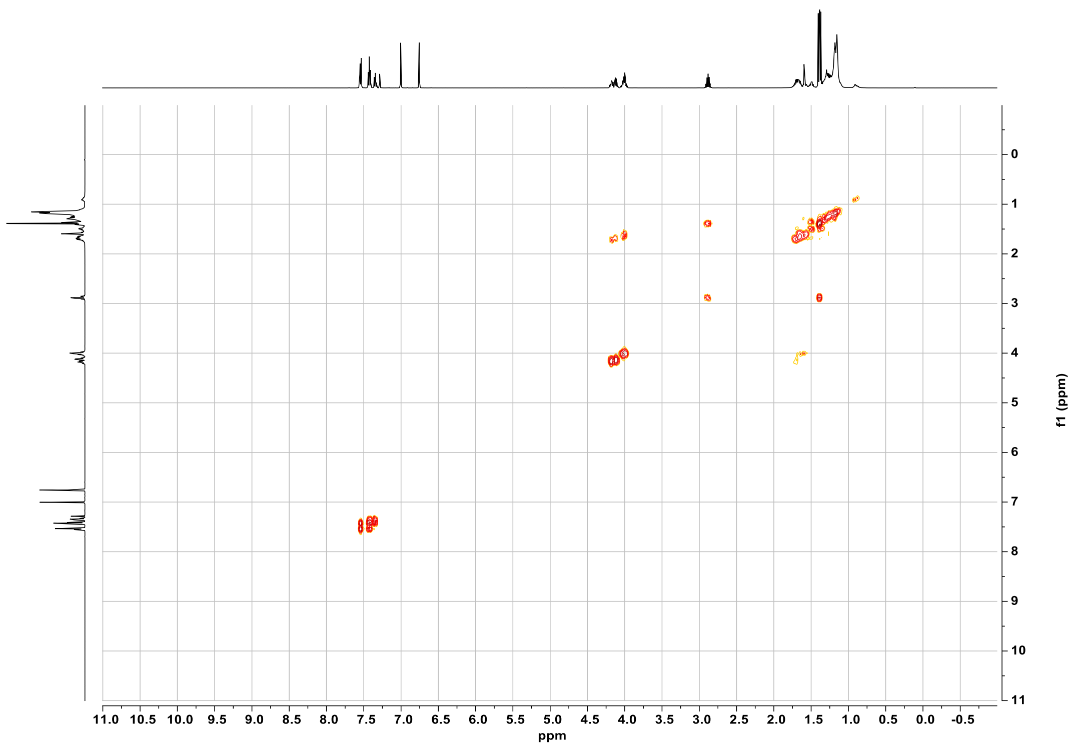

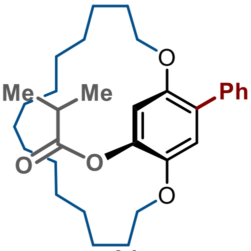

34

2D ^{1}H-^{13}C, $CDCl_3$, HSQC

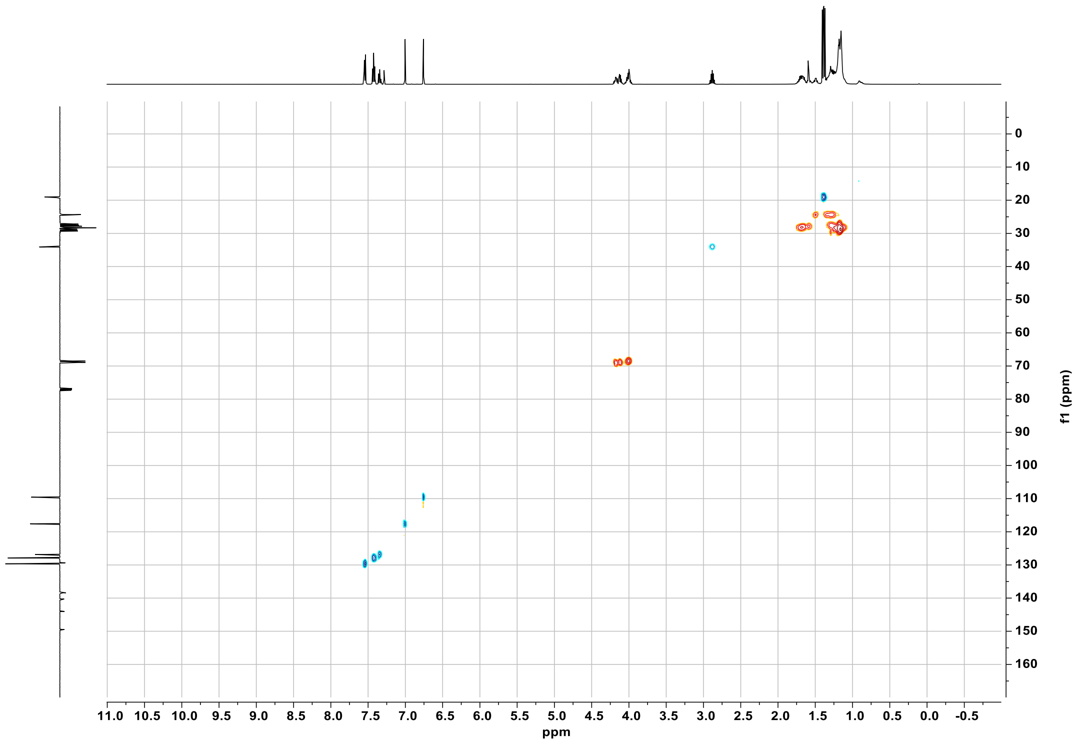

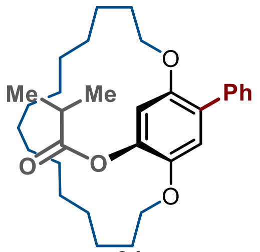

34

2D ^1H-^{13}C, $CDCl_3$, HMBC

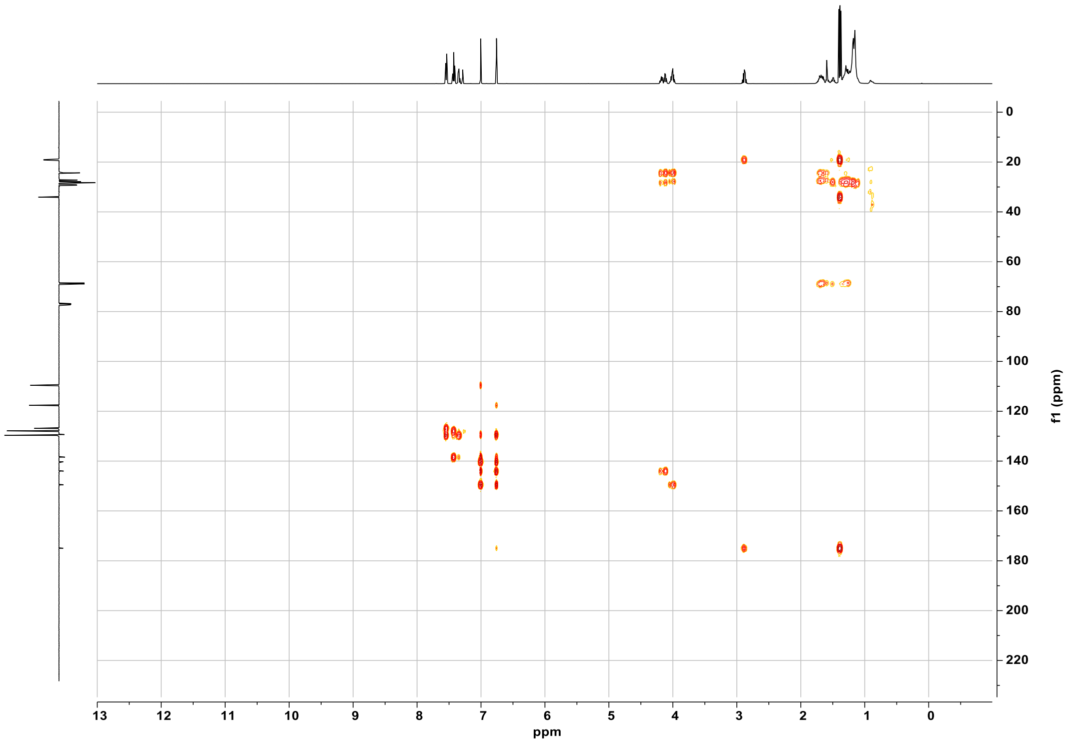

(*Rp*)-1⁵-phenyl-2,19-dioxa-1(1,4)-benzenacyclohexadecaphane-1²-yl isobutyrate 35

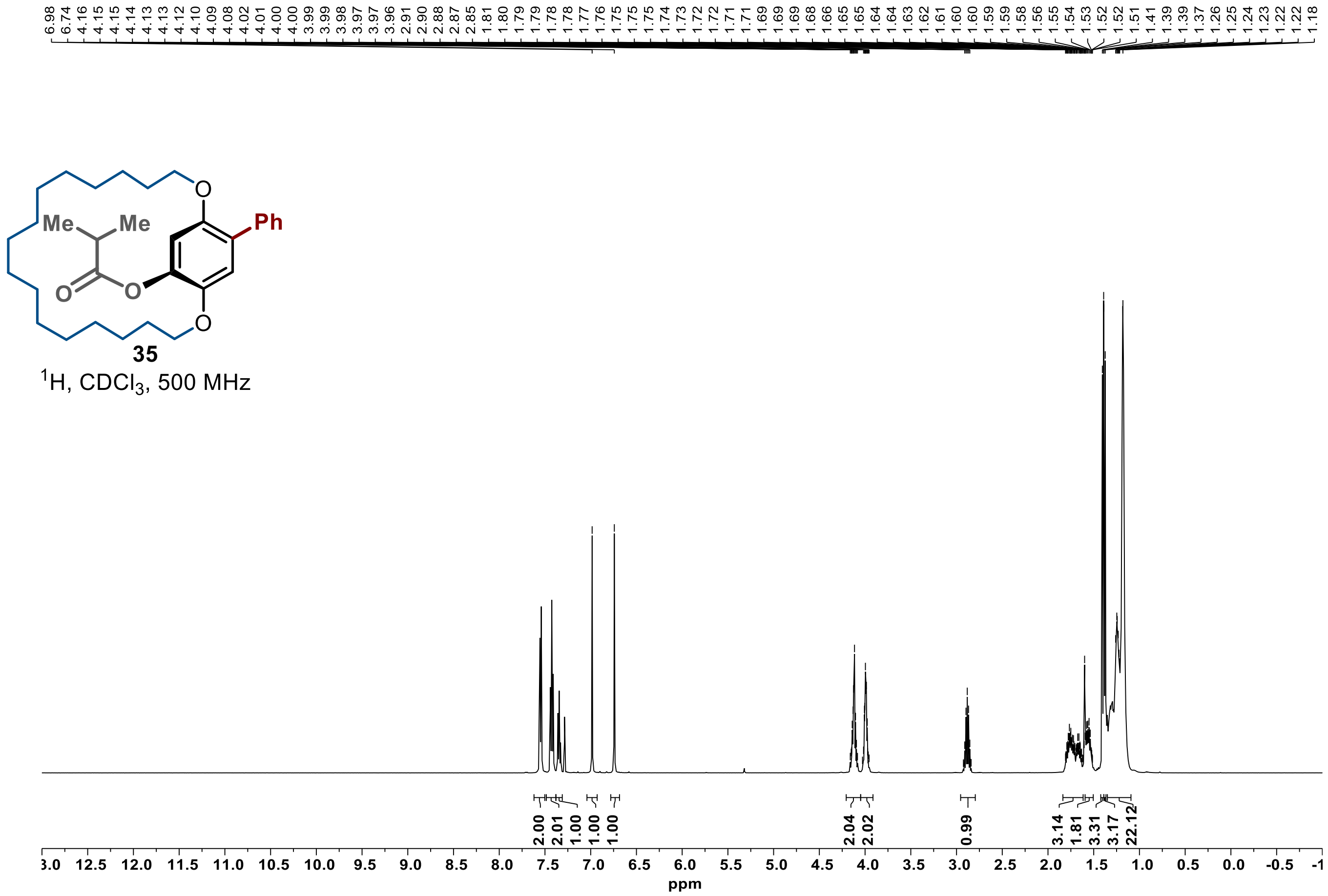

35

^{1}H, $CDCl_3$, 500 MHz

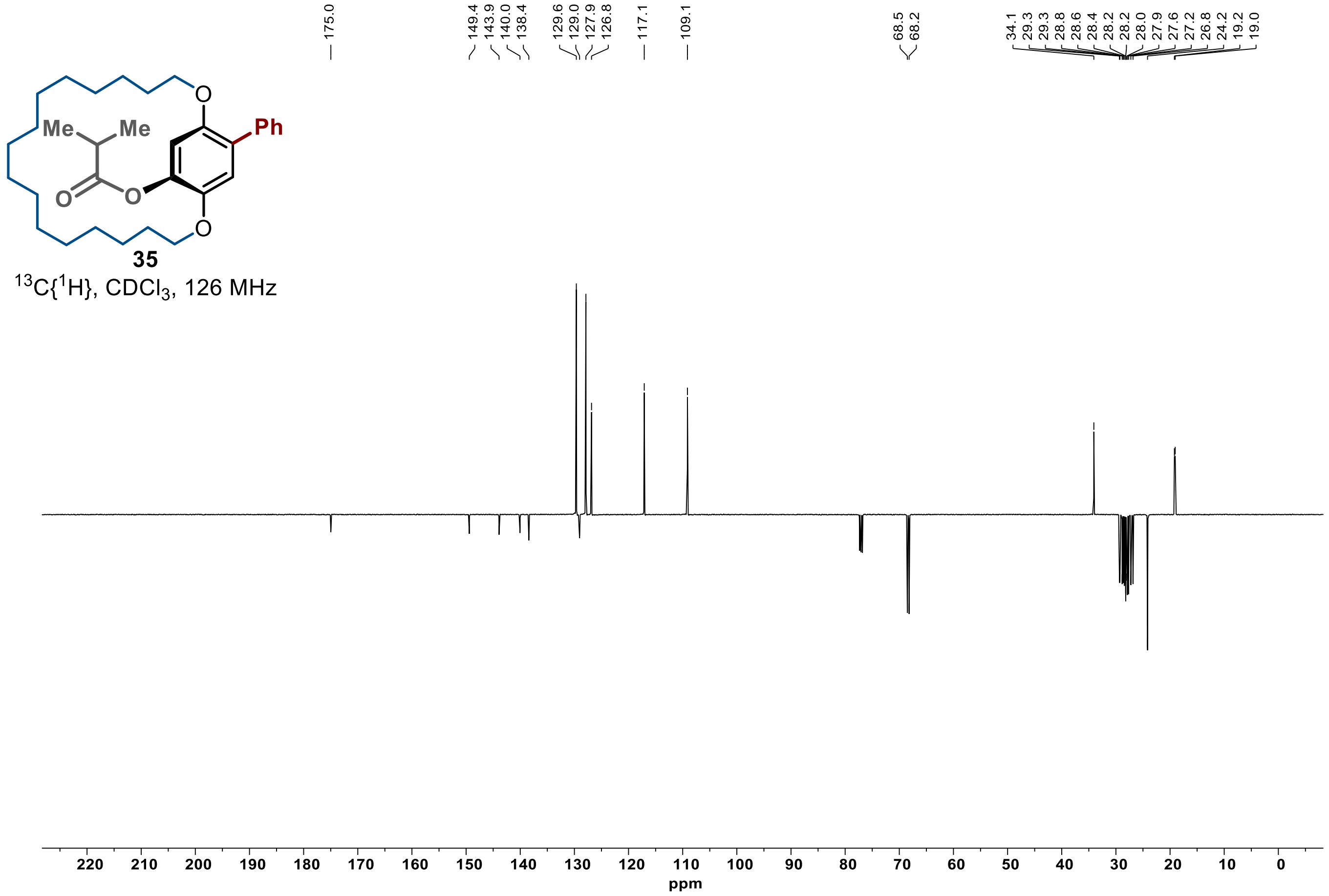

35

$^{13}C\{^1H\}$, $CDCl_3$, 126 MHz

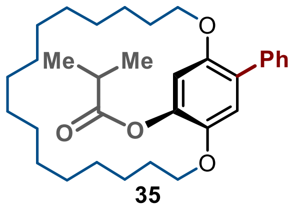

35

2D ^{1}H, $CDCl_3$, COSY

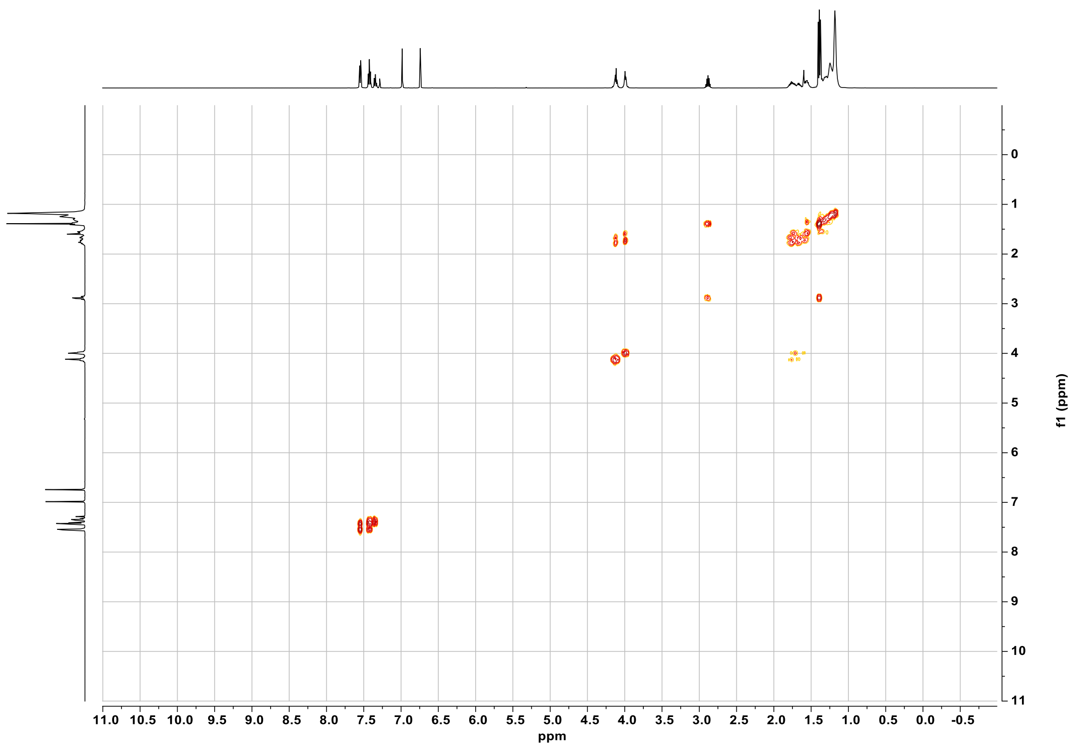

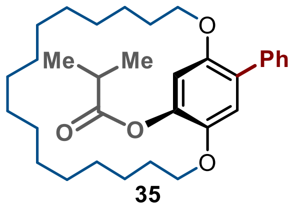

35

2D ^{1}H-^{13}C, $CDCl_3$, HSQC

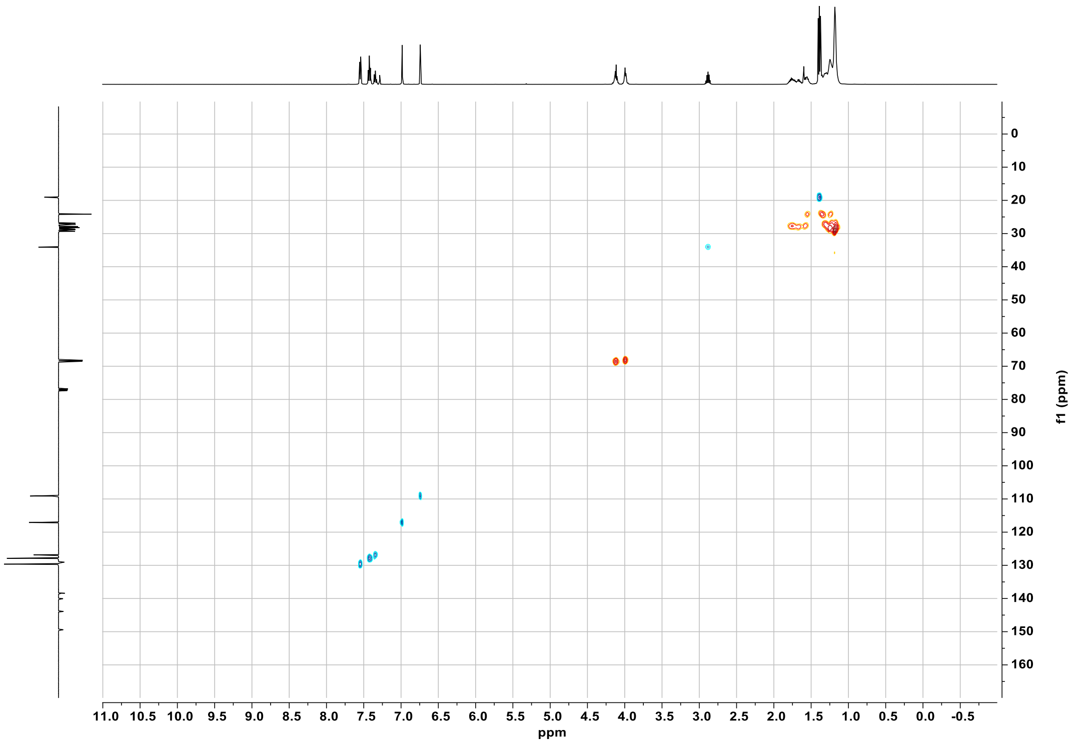

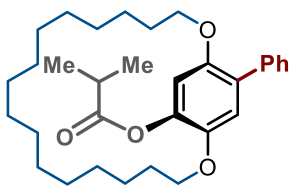

35

2D ^{1}H-^{13}C, $CDCl_3$, HMBC

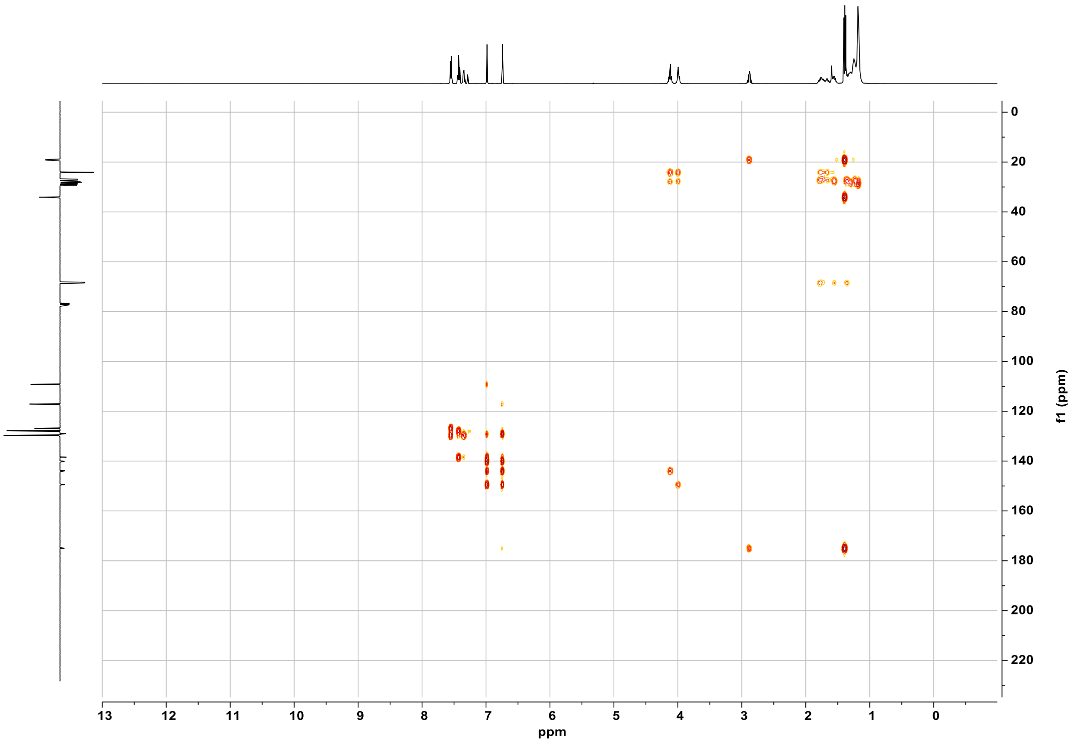

Supplement: Supplementary file 1 — Supporting Information [file ANIE-64-e202507126-s002.pdf]
